# Acetylome of *Acinetobacter baumannii* SK17 Reveals a Highly-Conserved Modification of Histone-Like Protein HU

**DOI:** 10.3389/fmolb.2017.00077

**Published:** 2017-11-27

**Authors:** Jiahn-Haur Liao, Cheng-Han Tsai, Sanjay G. Patel, Jhih-Tian Yang, I-Fan Tu, Matteo Lo Cicero, Magdalena Lipka-Lloyd, Wan-Ling Wu, Wen-Jie Shen, Meng-Ru Ho, Chi-Chi Chou, Garima R. Sharma, Hiroki Okanishi, Louis Y. P. Luk, Yu-Hsuan Tsai, Shih-Hsiung Wu

**Affiliations:** ^1^Institute of Biological Chemistry, Academia Sinica, Taipei, Taiwan; ^2^Institute of Biochemical Sciences, National Taiwan University, Taipei, Taiwan; ^3^School of Chemistry, Cardiff University, Cardiff, United Kingdom; ^4^Ph.D. Program in Microbial Genomics, National Chung Hsing University, Academia Sinica, Taipei, Taiwan; ^5^Republic Polytechnic, Singapore, Singapore; ^6^Department of Tumor Genetics and Biology, Kumamoto University, Kumamoto, Japan; ^7^Department of Chemistry, National Taiwan University, Taipei, Taiwan

**Keywords:** acetylome, *Acinetobacter baumannii*, DNA-binding proteins, genetic code expansion, HU

## Abstract

Lysine acetylation is a prevalent post-translational modification in both eukaryotes and prokaryotes. Whereas this modification is known to play pivotal roles in eukaryotes, the function and extent of this modification in prokaryotic cells remain largely unexplored. Here we report the acetylome of a pair of antibiotic-sensitive and -resistant nosocomial pathogen *Acinetobacter baumannii* SK17-S and SK17-R. A total of 145 lysine acetylation sites on 125 proteins was identified, and there are 23 acetylated proteins found in both strains, including histone-like protein HU which was found to be acetylated at Lys13. HU is a dimeric DNA-binding protein critical for maintaining chromosomal architecture and other DNA-dependent functions. To analyze the effects of site-specific acetylation, homogenously Lys13-acetylated HU protein, HU(K13ac) was prepared by genetic code expansion. Whilst not exerting an obvious effect on the oligomeric state, Lys13 acetylation alters both the thermal stability and DNA binding kinetics of HU. Accordingly, this modification likely destabilizes the chromosome structure and regulates bacterial gene transcription. This work indicates that acetyllysine plays an important role in bacterial epigenetics.

## Introduction

Post-translational modifications can confer novel properties to the modified proteins, including changes in enzymatic activity, subcellular localization, interaction partners, protein stability, and DNA-binding ability. In particular, acetylation of the ε-amino group of lysine residues is a widespread dynamic post-translational modification in eukaryotes and prokaryotes (Drazic et al., 2016). First discovered in eukaryotic histone proteins (Allfrey et al., 1964), lysine acetylation neutralizes the positive charge of the modified residues and weakens the interaction between histone and the negatively charged DNA backbone, leading to a structurally relaxed chromatin. Lysine acetylation of histones also serves as a molecular marker by many transcription factors for gene activation. Over the last few decades, lysine acetylation in eukaryotes has been clearly shown to play major roles in many essential cellular processes, and dysregulation of lysine acetylation is linked to several diseases, including cancer (Gil et al., 2017), cardiovascular diseases (Thiagarajan et al., 2016), and neurodegenerative disorders (Graff and Tsai, 2013). Likewise, lysine acetylation seems to be a general phenomenon in prokaryotes (Ouidir et al., 2016). The global lysine acetylation profiles have been brought out in many prokaryotes including *Bacillus subtilis* (Kim et al., 2013), *Cyanobacterium Synechocystis* (Mo et al., 2015; Chen et al., 2017), *Escherichia coli* (Zhang et al., 2013; Castano-Cerezo et al., 2014), *Mycobacterium tuberculosis* (Xie et al., 2015; Ghosh et al., 2016), *Salmonella enterica* (Wang et al., 2010), *Streptomyces roseosporus* (Liao et al., 2014), *Thermus thermophilus* (Okanishi et al., 2013), *Vibrio parahemolyticus* (Pan et al., 2014), etc. Based on these studies, the acetylated proteins appeared to be widespread in prokaryotes and participated in nearly all classes of cellular processes including adaptation and virulence (Ouidir et al., 2016). Nevertheless, the molecular mechanism and physiological importance of prokaryotic lysine acetylation remain largely elusive, though it has been suggested that regulating this type of protein modification may be a new avenue for developing antimicrobial agents (Ouidir et al., 2016). Accordingly, understanding the effects of site-specific lysine acetylation of prokaryotic proteins becomes an essential task in medicinal microbiology research.

Bacterial DNA-binding proteins, especially those involved in nucleoid compaction, represent promising targets against microbial infections because they often play key roles in bacterial survival and virulence. Histone-like protein, HU, is perhaps the most common bacterial DNA-binding proteins responsible for nucleoid compaction. This protein is ubiquitous and highly-conserved in all bacterial species. It has been shown to participate in all DNA-dependent functions. For example, HU knockout in Gram-positive bacteria is lethal due to disruption in genome integrity (Micka and Marahiel, 1992; Bartels et al., 2001; Liu et al., 2008; Nguyen et al., 2009). HU also modulates the pathogenicity of different bacteria mainly by transcriptional regulation (Alberti-Segui et al., 2010; Koli et al., 2011; Mangan et al., 2011; Wang et al., 2014; Phan et al., 2015). In addition, HU from *E. coli* is able to recognize specific mRNA and plays a role in regulation at a post-transcriptional level (Balandina et al., 2001). Interestingly, recent progresses in bacterial acetylome studies revealed that HU is subjected to post-translational acetylation in several microbes (Lee et al., 2013; Okanishi et al., 2013; Zhang et al., 2013; Castano-Cerezo et al., 2014; Pan et al., 2014; Kosono et al., 2015; Ouidir et al., 2015; Xie et al., 2015; Ghosh et al., 2016; Kentache et al., 2016). Nevertheless, the biophysical importance of HU acetylation has not yet been investigated.

To understand the biological significance of lysine acetylation in HU, a proteomics approach was used to reveal the acetylomes of *Acinetobacter baumannii* strains, SK17-S and SK17-R, which are sensitive and resistant to a carbapenem class antibiotic, imipenem, respectively (Chen et al., 2010). We found lysine acetylation in nearly all classes of proteins. Notably, a conserved lysine acetylation site on HU protein was found in both strains. To analyze its function, recombinant HU acetylated at Lys13, HU(K13ac), was prepared using genetic code expansion (Neumann et al., 2009). Acetylation of Lys13 affected the stability and DNA-binding kinetics of HU, indicating that this post-translational modification may regulate transcription and lead to epigenetic traits.

## Materials and methods

### Proteomic analysis of lysine acetylation

Using the published protocol (Lai et al., 2016), *A. baumannii* SK17-S and SK17-R were grown to the mid-exponential phase (OD_600nm_ = 0.8) before extraction of proteins. Extracted proteins were then subjected to in-solution tryptic digestion to yield dried peptide fragments as described before (Lai et al., 2016). Peptides were re-dissolved in NETN buffer (100 mM NaCl, 50 mM Tris-HCl, 1 mM EDTA, 0.5% w/v NP-40, pH 8.0). In parallel, 100 μl of the anti-acetyllysine antibody (Okanishi et al., 2013) at the concentration of 1 mg/ml were conjugated to 100 μg of protein G beads. The conjugated protein G beads were added into the peptide solution and incubated overnight at 4°C with gentle shaking. The beads were washed once with 1 ml ETN buffer (100 mM NaCl, 50 mM Tris-HCl, 1 mM EDTA, pH 8.0) and twice with 1 ml NETN buffer. The enriched peptides were eluted with 0.1% trifluoroacetic acid solution (400 μl × 3), and the eluent were passed through C18 Sep-Pak column before vacuum drying. Finally, ZipTip containing C18 reversed-phase media were used to desalt and concentrate peptides, which were then subjected to nano-scale liquid chromatography with tandem MS (nanoLC-MS/MS) analysis as described before (Lai et al., 2016).

All MS and MS/MS raw data were processed with Proteome Discoverer version 1.4 (Thermo Scientific), and the peptides were identified from the MS/MS data searched using the MASCOT search engine version 2.4.1 (Matrix Science) in combination with Sequest HT to search against the *A. baumannii* SK17 database (Lai et al., 2016). For each experiment, two raw files of MS/MS spectra generated from analyses of each protein fraction were merged into a single contiguous input file to facilitate bioinformatics analysis. The combined data files were searched by using the protein databases with the following parameters: a precursor mass within ±5 ppm of the theoretical mass, a fragment ion tolerance of <0.6 Da, and allowed a maximum of two missed cleavages sites for trypsin. Carbamidomethylation on cysteine was specified as a fixed modification, whereas oxidation on methionine and acetylation on lysine were set as variable modifications. The maximum false discovery rate (FDR) threshold of peptides and proteins was set to 0.05, for those FDR <0.01 were indicated with additional marks. Charge states of +2, +3, and +4 were considered for parent ions. Only rank-one peptides were counted for filtering the output data set. Peptides containing acetyllysine residue(s) with MASCOT score >20.0 were manually verified as described previously (Chen et al., 2005). Bioinformatics analysis of the identified peptides was carried out as described before (Lai et al., 2016).

### Molecular phylogenetic analysis

The evolutionary history was inferred by using the Maximum Likelihood method based on the James-Taylor-Thornton matrix-based model. The percentage of trees in which the associated taxa clustered together is shown next to the branches. Initial tree(s) for the heuristic search were obtained automatically by applying Neighbor-Join (NJ) and BioNJ algorithms to a matrix of pairwise distances estimated using a James-Taylor-Thornton model, and then selecting the topology with superior log likelihood value. The tree is drawn to scale, with branch lengths measured in the number of substitutions per site. The analysis involved 33 amino acid sequences. All positions containing gaps and missing data were eliminated. There was a total of 90 positions in the final dataset. Evolutionary analysis was conducted in MEGA7 (Kumar et al., 2016).

### Homology modeling

Amino acid sequence of *A. baumannii* HU (Swiss-Prot/TrEMBL accession number B0VNW6) was used to build all homology models by SWISS-MODEL (Biasini et al., 2014; Bienert et al., 2017) or I-TASSER (Yang et al., 2015). For SWISS-MODEL, pdb 1P51 (*Anabaena* HU), and 4P3V (*E. coli* Huβ) were used as the templates to generate homology models with and without DNA bound to the protein, respectively. For I-TASSER, default setting was applied, and the generated model has C-score = 0.74, estimated TM-score = 0.81 ± 0.09, and estimated RMSD = 2.4 ± 1.8 Å. The models were visualized with PyMOL (Schrödinger).

### Plasmid construction

Chromosomal DNA from both *A. baumannii* SK17-S and SK17-R were isolated by phenol extraction (Chan and Goodwin, 1995). The *hupB* gene was PCR amplified from the chromosome DNA. Sanger sequencing confirmed that the *hupB* gene in both SK17-S and SK17-R strains has identical sequence. The *hupB* gene was then cloned into pET-21a(+) vector (Novagen) between the NdeI and XhoI sites to provide a plasmid that expresses wild-type HU with a C-terminal His-tag. The plasmid, pET-21a hupB, was then PCR amplified using the two primers: TTCAGCAATTGCATCGATTAATTCTGATTTATTCATATG and TAATCGATGCAATTGCTGAAtagGGGGGAGTATCTAAGACTGATGCAG. These primers replace the original lysine codon (AAA) with an amber stop codon (TAG) for site-specific incorporation of acetyllysine. The PCR product (5,657 bp) was transformed into chemically competent *E. coli* cells to afford plasmid expressing HU(K13ac).

To construct a plasmid that expresses wild-type HU without a His-tag, a 342 bp PCR amplimer generated using primers TGAGCGATTACGACATCCCCACTACTGAGAATCTTTATTTTCAGGGCGCCATGAATAAATCAGAATTAATCGATGC and GCTTTGTTAGCAGCCGGATCTCAAGCAACTGAATCTTTAAGACC from pET-21a hupB was joined by Gibson assembly to a 5,397 bp PCR amplimer generated using primers AGTGGGGATGTCGTAATCGCTCATGGGGTGATGGTGATGGTGATGTTTCATATGTATATCTCCTTCTTAAAGTTAAAC and GCTTGAGATCCGGCTGCTAACAAAGCCCGAAAGGAAG from pET-21a hupB. This construct contains a N-terminal His-tag, followed by a PMSDYDIPTT linker and a TEV protease cleavage sequence (ENLYFQG).

To construct a plasmid that expresses acetyllysyl-tRNA synthetase/tRNA_CUA_ pair, the plasmids pCDF PylST (Neumann et al., 2010) and pBK AcKRS (Neumann et al., 2009), gifts from Jason Chin, were digested with BamHI and Eco147I, and the digested vector (4,630 bp) from pCDF PylST and the digested insert (1,382 bp) from pBK AcKRS were purified by gel extraction, followed by ligation to afford the plasmid pCDF AcKST.

### Recombinant protein production and purification

To generate the wild-type protein with or without His-tag, *E. coli* BL21(DE3) cells containing the appropriate plasmid were grown at 37°C in terrific broth (TB) medium containing ampicillin (100 μg/ml). Fresh TB medium containing ampicillin (100 μg/ml) was inoculated with 2% v/v overnight culture and was incubated at 37°C. At OD_600_ 0.6, isopropyl β-D-thiogalactopyranoside (IPTG) was added into the culture to a final concentration of 0.5 mM to induce gene expression at 20°C for 18 h.

To express the acetylated protein, *E. coli* BL21(DE3) cells containing pET21a hupB(K13TAG) and pCDF AcKST were grown at 37°C in TB medium containing ampicillin (50 μg/ml) and spectinomycin (25 μg/ml). Fresh TB medium containing ampicillin (50 μg/ml) and spectinomycin (25 μg/ml) was inoculated with 2% v/v overnight culture and was incubated at 37°C. At OD_600_ 0.5, the culture was supplemented with nicotinamide (final concentration = 20 mM) and *N*_ε_-acetyl-L-lysine (final concentration = 5 mM). Gene expression was induced 30 min later by the addition of IPTG and incubation at 20°C for 18 h.

Cells were harvested by centrifugation at 6,000 × *g* at 4°C for 30 min, re-suspended in lysis buffer (500 mM NaCl, 25 mM Tris-HCl, 20 mM imidazole, pH 7.5) containing lysozyme (1 mg/ml) and phenylmethanesulfonyl fluoride (100 μM), and lysed by sonication. Cell debris was removed by centrifugation at 39,000 × *g* for 30 min. The clear supernatant containing the hexahistidine-tagged proteins was purified using a Ni-NTA column, washed with 50 mM immidazole and eluted in 250 mM imidazole. Fractions containing the desired protein were identified by SDS-PAGE analysis and pooled for further treatment.

For wild-type and K13ac HU proteins containing a C-terminus His-tag, the pooled fractions were subjected to cation-exchange chromatography on a NGC Chromatography System (Bio-Rad). Resource S column (GE Healthcare, #17-1180-01) was equilibrated with potassium phosphate buffer (10 mM, pH 7.0, 5% v/v gycerol). Sample was loaded on the column and eluted using the same buffer with an increasing amount of NaCl (0–500 mM).

To generate wild-type HU without the His-tag, the protein purified by Ni-NTA column was exchanged into Tris buffer (25 mM, pH 8.0) to a concentration of 1–2 mg/ml. The protein was incubated with recombinant His-tagged TEV protease (10% w/w) in the presence of 5 mM DTT for 18 h at 20°C. The sample was loaded onto a Ni-NTA column, and the flow through was collected.

After purification, proteins were concentrated by Amicon stirred cell using 3 kDa cutoff membrane (Millipore, #PLBC04310). Protein concentration was determined by BCA assay. Protein yields were 15–20 mg/l for wild-type HU with a C-terminal His-tag, 1–1.5 mg/l for K13ac HU with a C-terminal His-tag, and 12–15 mg/l for wild-type HU without His-tag. For storage, 20% v/v glycerol was added into the buffer, and proteins were kept at −80°C.

### Analytical size-exclusion chromatography

Size-exclusion chromatography was performed on a NGC Chromatography System (Bio-Rad). Superdex 75 10/300 GL (24 ml, GE Healthcare) was equilibrated with PBS. Sample was then loaded on the column, followed by elution with PBS at a flow rate of 0.5 ml/min. Fractions were collected and analyzed by SDS-PAGE to confirm the peak corresponding to HU protein.

### CD spectroscopy

CD experiments were performed on an Applied PhotoPhysics Chirascan spectrometer using 11 μM protein in deoxygenated potassium phosphate buffer (10 mM, pH 7.0). Spectra were measured between 200 and 400 nm in 1 mm quartz cuvettes under N_2_ with a 50 nm/min scan speed, 0.5 nm data pitch, 1 nm bandwidth, and 0.5 s response time. To measure the thermal melting point, CD spectra were collected every 2°C as temperature increased from 5 to 85°C. The rate of temperature increase was 0.5°C/min with 300 s equilibration time at each temperature.

### Electrophoretic mobility shift assay

DNA (250 ng) was incubated with varied concentrations of protein at 37°C for 1 h in binding buffer in a 10 μl reaction volume (Ghosh et al., 2016). Following incubation, the samples were mixed with 5x GelPilot DNA Loading Dye (Qiagen) and loaded on 1% agarose gel containing SYBR Safe (Thermo Fisher) in 1x TAE buffer (40 mM Tris-acetate and 1 mM EDTA pH 8.0). The gel was electrophoresed at 130 V at 20°C for 45 min, visualized and analyzed using a ChemiDoc XRS+ System (Bio-Rad).

Plasmids pBK AcKRS (3,219 bp) and pCX eGFP (5,514 bp) were chosen due to their small size, and they only have identical sequence around the origin of replication (725 bp). Restriction enzyme BamHI (for pBK AcKRS) or BcuI (for pCX eGFP) was used to generate linear DNA. The plasmids were gifts from Jason Chin (Neumann et al., 2009) and Anthony Perry (Perry et al., 1999), respectively.

### Biolayer interferometry analysis

All the biolayer interferometry experiments were performed on an Octet RED96 System (Pall ForteBio). Samples in buffer were dispensed into polypropylene 96-well black flat-bottom plates (#655209, Greiner Bio-One) at a total volume of 200 μl per well, and all measurements were performed at 25°C with agitation at 1,000 rpm. Streptavidin-coated biosensor tips (#18-5019, Pall ForteBio) were pre-wetted with assay buffer (30 mM Tris, pH 7.5, 100 mM NaCl, 0.02% v/v Tween20, 0.5 mg/ml BSA). Biosensors were then loaded with biotin-labeled dsDNA (5′-biotin-ATTAATATTTTCTTAAACTAATTTAAAAT) by incubation at the concentration of 0.1 μM for 300 s, and excess DNA were removed by incubation with assay buffer for 300 s. To measure association and dissociation kinetics, biosensors were transferred into fresh assay buffer for 300 s to collect a baseline read, followed by incubation with the protein at a specific concentration for 300 s to record protein association and incubation with fresh assay buffer for 300 s to record protein dissociation. Measurements were performed from low to high protein concentration. Tips were regenerated between each measurement by repeatedly incubation in regeneration buffer (10 mM glycine, pH 2.0, 20 seconds) and fresh assay buffer (20 s) for three times.

Data were analyzed using ForteBio Data Analysis Software version 8.1. The binding profile of each sample was recorded as an “nm shift” (the wavelength or spectral shift in nanometers), which represented the difference between the start and end of the kinetic cycle. All sensorgrams were referenced for buffer effects, and the Y axes were aligned to the association step with an inter-step correction of aligning to the dissociation step. Kinetic responses were fitted to a 1:1 Langmuir binding model to obtain values for association (*k*_on_), dissociation (*k*_off_) rate constants, and the equilibrium dissociation constant (*K*_D_). All fitted curves have *R*^2^ > 0.90.

### Atomic force microscopy

Sample solutions contained linear pCX eGFP DNA (by treatment with BcuI) at 10 ng/μl and/or the designated HU protein (3 μM) in buffer (10 mM HEPES, pH 7.5, 4 mM MgCl_2_). This protein: DNA ratio equals to the presence of 1 HU homodimer for every 10 bp DNA, if all proteins bind. The solution was either incubated at 37°C for 1 h or without incubation. Ten microliters of sample solution was applied to freshly cleaved mica by spin coating (15 s ramp 0–2,000 rpm; 30 s at 2,000 rpm), followed by washing with water (2 ml, full wetting with intervals at 3,000 rpm) and drying (4 min at 3,000 rpm).

Atomic force microscopy measurements were carried out in air at 293 K, using a Nanoscope V instrument (Veeco), type Multimode 8. Probes PPP-NCHR POINTPROBE-PLUS® Silicon-SPM-Sensor were used (resonance frequency = 330 kHz; force constant = 42 N/m, length = 125 μm, NanosensorsTM) operating in tapping mode at a frequency around 321.5 kHz to image the surfaces through ScanAsyst®-air mode. The data were processed using the WS × M software (Horcas et al., 2007).

### Experimental design and statistical rationale

Tier 3 measurements, as defined by the National Cancer Institute's Clinical Proteomic Tumor Analysis Consortium (CPTAC) Program, were performed here. Acetylated peptides from whole cell lysates of *A. baumannii* SK17-S and SK17-R were fractionated and enriched using immunoaffinity enrichment strategies (Okanishi et al., 2013). Each strain was analyzed by high-accuracy nanoflow LC-MS/MS technology. Yields for the recombinant protein expression were based on three independent experiments. The same pools of proteins were subjected to biochemical characterizations. For analytical size-exclusion chromatography, identical samples were run twice on the instrument. For electrophoretic mobility shift assay, a representative gel from duplicates was shown for each condition. For biolayer interferometry analysis, three independent measurements were carried out for each protein. Mean and standard deviation calculated from the three experiments are presented.

## Results

### Acetylome of *A. baumannii* SK17-S and SK17-R

Two clinical isolates of *A. baumannii*, SK17-S and SK17-R (Chen et al., 2010), were used in this study. Strain SK17-S isolated first from a male hospitalized patient is susceptible to antibiotic imipenem, whereas strain SK17-R isolated later from the same patient is resistant to this antibiotic. To investigate the acetylomes of these two strains, cells were harvested at mid-exponential phase, and a polyclonal anti-acetyllysine antibody (Okanishi et al., 2013) was employed to enrich acetylated peptides from the whole-cell protein tryptic digests before analysis by high-resolution mass spectrometry (MS).

**Figure 1:**
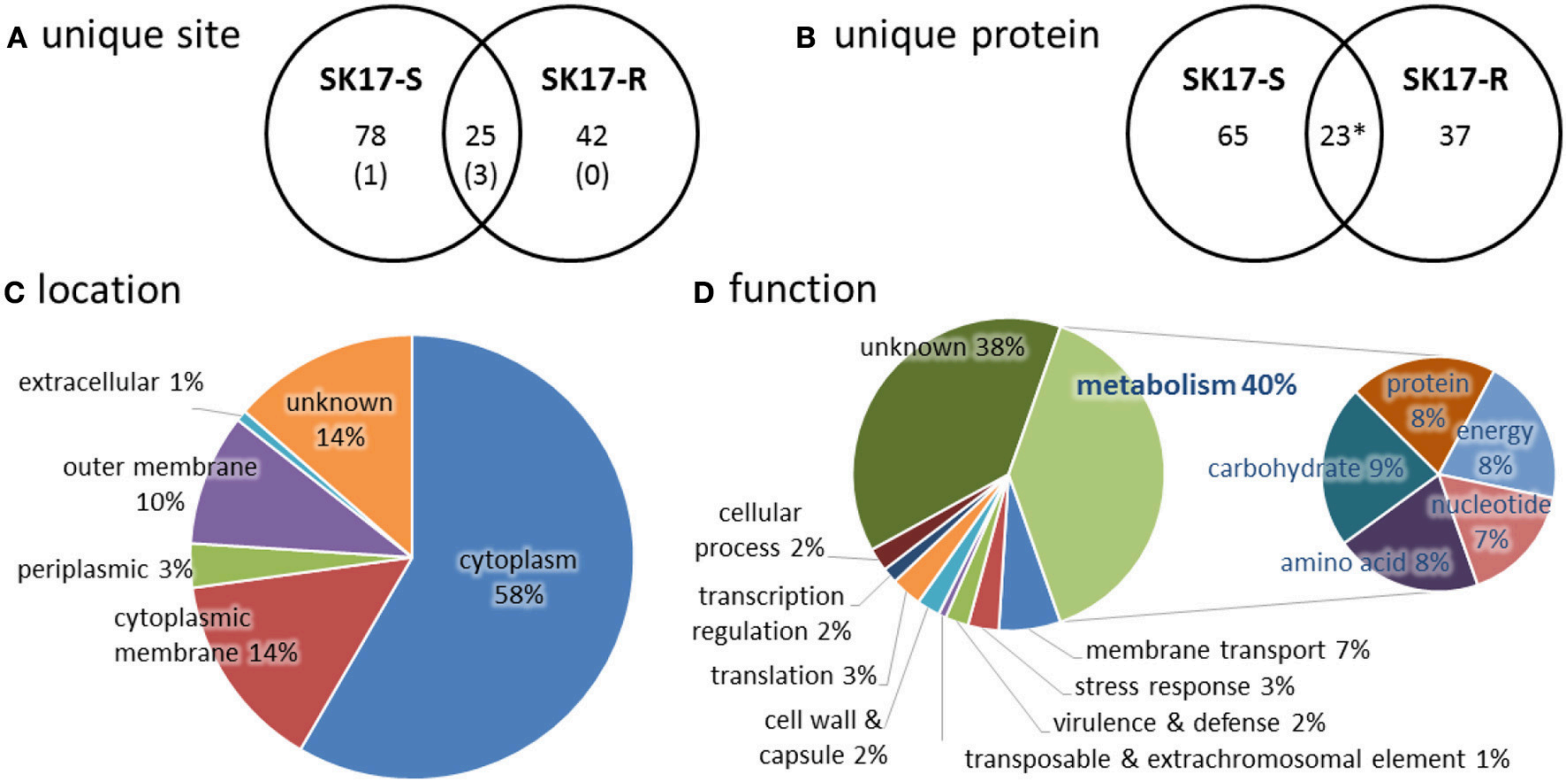
Statistics of acetylome in *A. baumannii* SK17-S and SK17-R at mid-exponential phase. **(A)** Unique acetylation site found in SK17-S and SK17-R. Number in bracket shows the identical acetylation site reported in *A. baumannii* ATCC19606 harvested at stationary phase (Kentache et al., 2016). **(B)** Unique acetylated protein found in SK17-S and SK17-R. ^*^Acetylation of SSU ribosomal protein S1p was found in both SK17-S and SK17-R but at different positions (K479 for SK17-S and K528 for SK17-R). **(C)** Classification of the identified acetylated proteins according to cellular localization. **(D)** Classification of the identified acetylated proteins according to putative function.

In total, we identified 145 unique lysine acetylation sites in 132 unique acetylated peptides from 125 proteins that have diverse functions and localize in different part of the cells (Tables S1–S4). Among these, there are 103 sites on 88 proteins in SK17-S and 67 sites on 60 proteins in SK17-R, including 25 sites on 22 proteins in both strains (Figures 1A,B). It is noteworthy that only four identical acetylation sites were previously reported in *A. baumannii* ATCC19606 harvested at stationary phase (Kentache et al., 2016), including DNA-binding protein HU (Table S1). Furthermore, most of the identified proteins locate in cytoplasm (Figure 1C). From the functional perspective, 77 of the 125 identified proteins could be functionally annotated to metabolism of nucleotide, amino acid, protein, carbohydrate, or energy (Figure 1D). This finding is consistent with the previous studies of bacterial acetylome (Ouidir et al., 2016), suggesting a close link between protein acetylation and metabolic processes.

### DNA-binding protein HU

**Figure 2:**
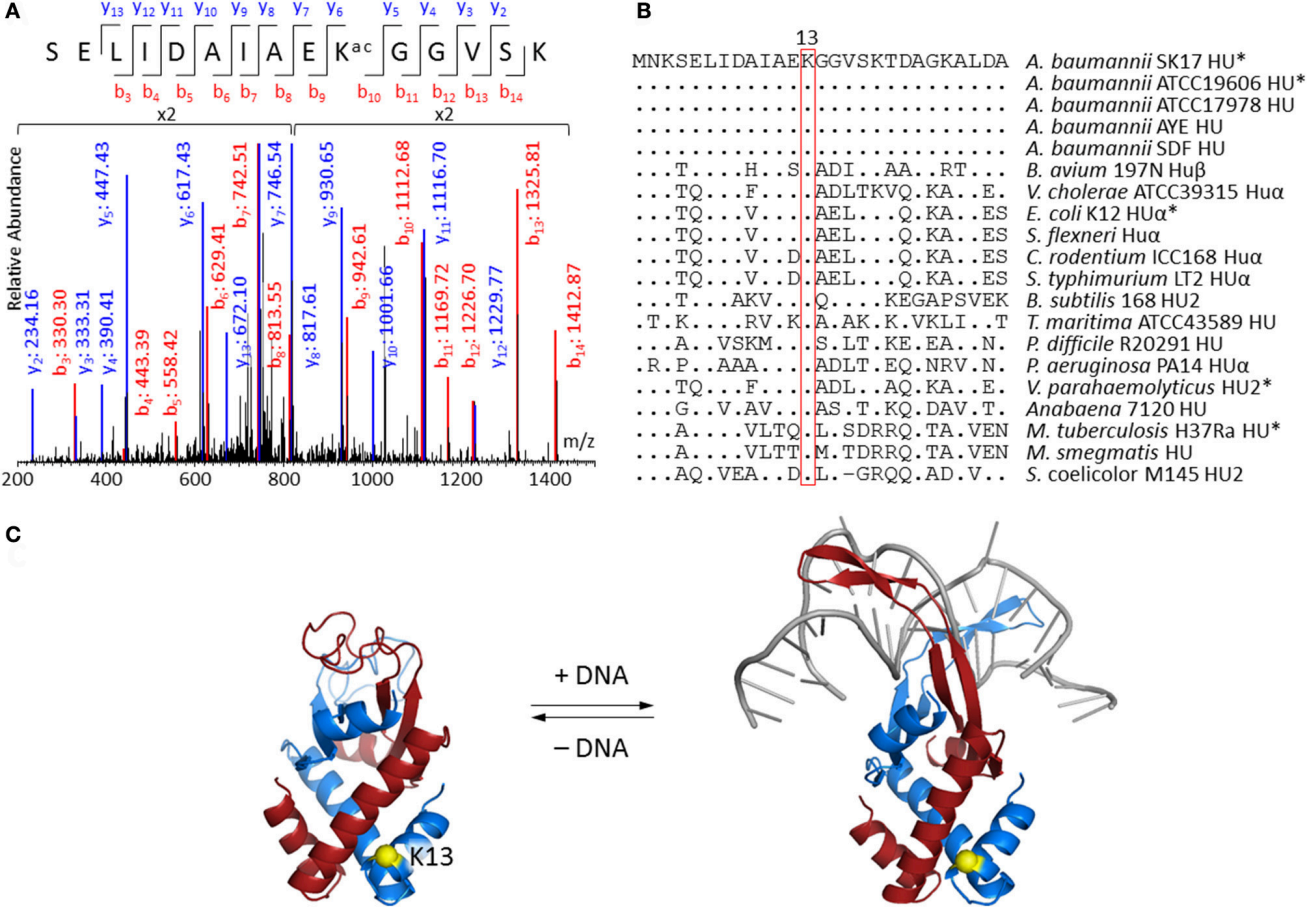
Acetylation at Lys13 of HU. **(A)** MS/MS spectrum showing Lys13 acetylation in *A. baumannii* SK17 HU. MS/MS spectra of all identified acetylated peptides are shown in Supplemental Data. **(B)** Sequence alignment of bacterial HU homologs. Sequences were aligned to amino acid residues 1–27 of *A. baumannii* SK17 HU. Dot (.) and hyphen (−) indicates identical amino acid residues and gap, respectively, in comparison to *A. baumannii* SK17 HU sequence. Proteins known to be acetylated at Lys13 are shown with asterisk (^*^). Full sequence alignment with all known acetylation sites is shown in Figure S2. **(C)** Homology models of *A. baumannii* HU. Homology model without or with DNA was generated by SWISS-MODEL using pdb 4P3V or 1P51 as the template. DNA is shown in gray. Two subunits of the homodimer are shown in blue and red. Lys13 of one subunit is shown in yellow. Alignment of the homology models is shown in Figure S3.

Lysine acetylation on DNA-binding protein HU is of particular interest because of its potential to change the biophysical properties and consequently the function of this protein which is known to regulate cell growth and pathogenicity (Lee et al., 2013; Okanishi et al., 2013; Zhang et al., 2013; Castano-Cerezo et al., 2014; Pan et al., 2014; Kosono et al., 2015; Ouidir et al., 2015; Xie et al., 2015; Ghosh et al., 2016; Kentache et al., 2016). MS/MS spectrum of peptide fragment SELIDAIAEKacGGVSK (corresponding to residues 4–18 of HU) clearly showed acetylation at Lys13 (Figure 2A). This acetylated peptide was found in both *A. baumannii* SK17-S and SK17-R, indicating that Lys13 acetylation is a common phenomenon in this organism. Molecular phylogenetic analysis showed that *A. baumannii* HU protein is closely related to homologs in other bacteria (Figure S1), and sequence alignment revealed that Lys13 is conserved in many HU homologs (Figure 2B). In fact, Lys13 acetylation (K13ac) was found in *A. baumannii* ATCC19606 (Kentache et al., 2016), *E. coli* K12 (Zhang et al., 2013) and BW25113 (Castano-Cerezo et al., 2014), *M. tuberculosis* H37Ra (Xie et al., 2015; Ghosh et al., 2016), and *Vibrio parahaemolyticus* (Pan et al., 2014; Figure 2B and Figure S2). The presence of K13ac in both SK17-S and SK17-R as well as other pathogenic bacteria indicates this site-specific modification may play critical role in bacterial growth or infection. In addition, bacterial HU has analogous function to eukaryotic histones on DNA compaction (Grove, 2011), so HU acetylation may affect DNA binding and serve as epigenetic modulator.

Since no crystal structure of *A. baumannii* HU is available, SWISS-MODEL (Biasini et al., 2014; Bienert et al., 2017) and I-TASSER (Yang et al., 2015) were used to generate homology models. SWISS-MODEL was used to build the homodimeric structure of the 90-amino-acid *A. baumannii* HU with or without DNA (Figure 2C). The DNA-bound structure is based on the 94-amino-acid *Anabaena* HU that shares 56% sequence identity and 72% similarity, whereas the DNA-free structure is based on the 90-amino-acid *E. coli* HUβ that shares 70% sequence identity and 83% similarity. On the other hand, I-TASSER generated a monomeric structure that aligned well to the subunit of DNA-bound structure produced by SWISS-MODEL (Figure S3). In fact, the only discrepancy among the three models is between the amino acid residue 55 and 73, the region involving in DNA binding. The homology model indicated that Lys13 does not directly interact with the DNA. Such a phenomenon is commonly observed in eukaryotic histone proteins (Shahbazian and Grunstein, 2007), where most histone acetylation sites locate in the N-terminal tails that do not directly interact with DNA and are accessible in nucleosome complexes. Acetylation at those sites can regulate nucleosome assembly, DNA accessibility by other proteins and gene transcription in eukaryotes (Shahbazian and Grunstein, 2007). Therefore, we decided to investigate if Lys13 acetylation changes the properties and/or function of *A. baumannii* HU.

### Physical properties of recombinant HU and HU(K13ac)

To pinpoint the effect of Lys13 acetylation, the technique of genetic code expansion was used to produce homogenously acetylated protein, HU(K13ac) that has acetyllysine at the amino acid residue 13. Site-specific incorporation of acetyllysine was achieved through an orthogonal acetyllysyl-tRNA synthetase/tRNA_CUA_ pair (Neumann et al., 2009). A His tag was introduced at the C-terminus to facilitate purification (Figure S4). HU and HU(K13ac) were overexpressed in *E. coli* BL21(DE3), purified by Ni-NTA column and cation-exchange chromatography. Yield of 15–20 and 1–1.5 mg/l was obtained for the wild-type and K13ac HU, respectively.

**Figure 3:**
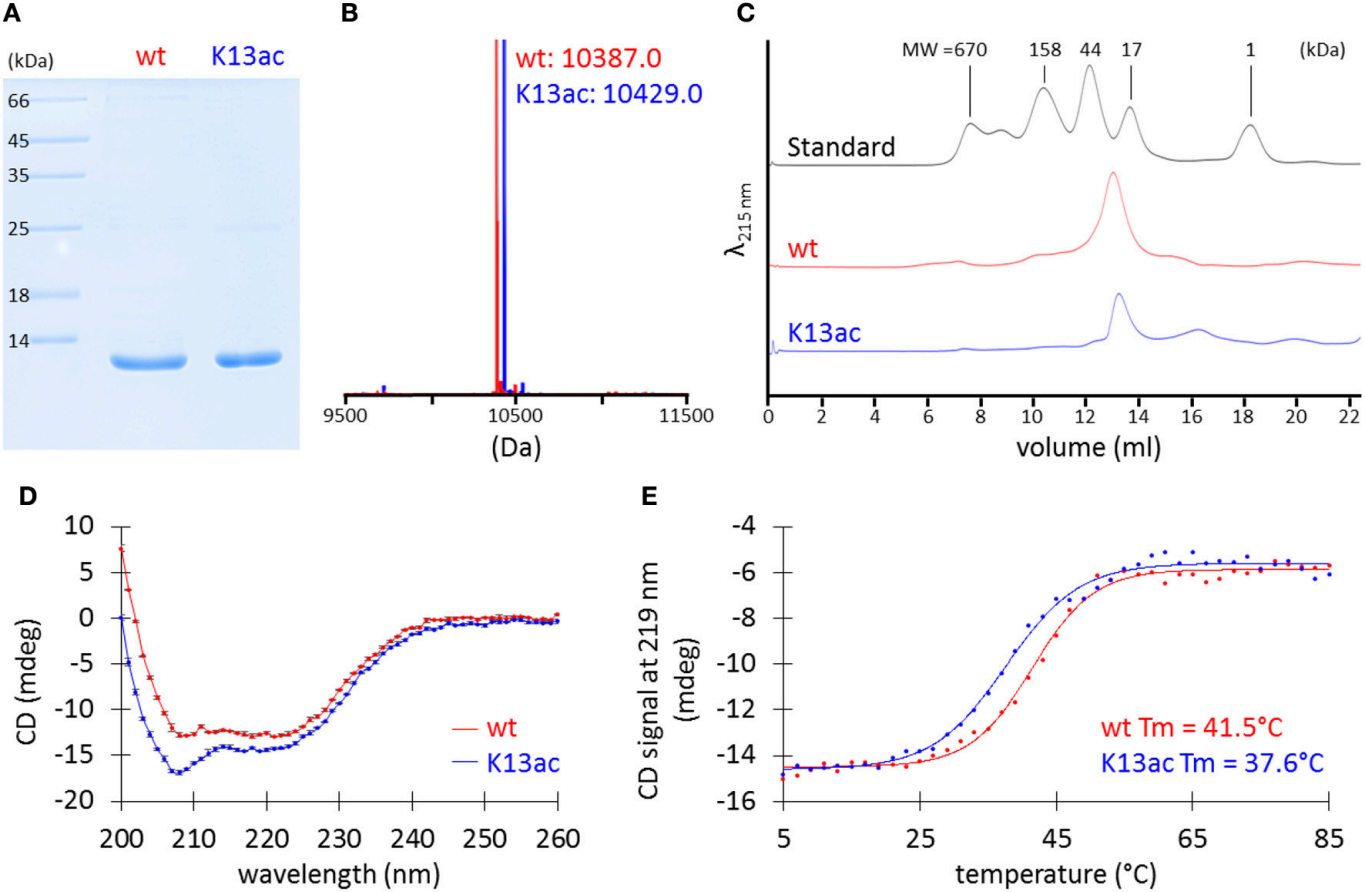
Characterization of purified recombinant wt and K13ac HU proteins. **(A)** SDS-PAGE. The gel was stained by Coomassie Brilliant Blue to visualize proteins. **(B)** MS. Theoretical MW of wt and K13ac HU is 10386.8 and 10428.9, respectively. Amino acid sequence and full mass spectrum of wt and K13ac HU are shown in Figure S4. **(C)** Analytical size-exclusion chromatography. Duplicate of size-exclusion chromatography runs and molecular weight estimation are shown in Figure S5. **(D)** CD of wt and K13ac HU at 11 μM and 20°C in potassium phosphate buffer (10 mM, pH 7.0). Dots show the average value measured from the same sample for three times, and error bars show the standard deviation of the three values. **(E)** Melting temperature (Tm) based on CD signal at 219 nm.

SDS-PAGE analysis of the purified proteins showed bands at the desired size (Figure 3A), and MS spectra confirmed the incorporation of acetyllysine (Figure 3B and Figure S4). Although there has been no prior experimental study of *A. baumannii* HU, bacterial HU proteins characterized to date normally exist in a dimeric form (Grove, 2011). However, it is also known that two point mutations of *E. coli* Huα can transform the dimeric protein into an octamer (Kar et al., 2006). Thus, we applied analytical size-exclusion chromatography to determine the oligomeric states of *A. baumannii* HU. Using Superdex 75 10/300 GL column, both wild-type and K13ac HU migrate between the 44-kDa ovalbumin and 17-kDa myoglobin (Figure 3C) marker with the estimated molecular weight of about 25 kDa (Figure S5), indicating that *A. baumannii* HU exists as a dimer and Lys13 acetylation does not change the oligomeric state. Lastly, the effect of Lys13 acetylation on protein secondary structure and thermal stability was investigated by circular dichroism (CD). At 20°C, there is a small change of the CD spectra around 209 nm (Figure 3D) likely due to acetylation at Lys13 that localizes on an α-helix (Figure 2C). Indeed, the overall CD spectra indicate that both proteins exist as mainly α-helix, which is in accordance with the homology models (Figure 2C). On the other hand, by measuring CD spectra at different temperature, we were able to calculate the melting temperature and found that Lys13 acetylation lowers the melting temperature by 4°C (Figure 3E). It is likely that either acetylation renders the protein intrinsically less stable or destabilizes the dimeric complex of HU, consequently decrease the protein thermal stability.

### Effect of Lys13 acetylation on protein-DNA interaction

**Figure 4:**
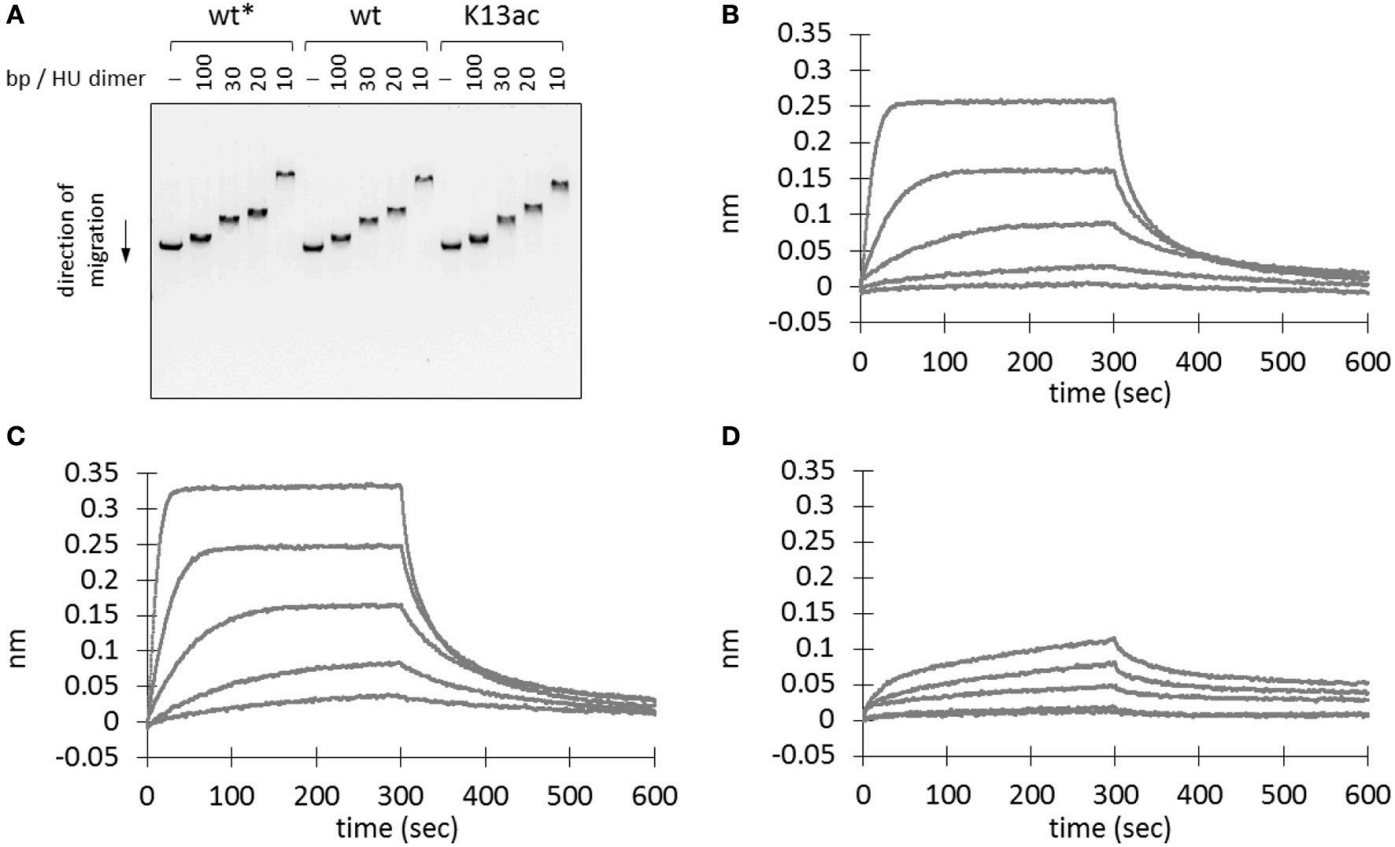
Effect of Lys13 acetylation on protein-DNA interaction. **(A)** Electrophoretic mobility shift assay. BamHI-treated linearized plasmid DNA, pBK AcKRS, (250 ng) was incubated with different amount proteins as indicated in binding buffer (30 mM Tris pH 7.5, 100 mM NaCl, 0.02% v/v Tween20, 0.5 mg/ml BSA) at 37°C for 1 h before electrophoresed on a 1% agarose gel. Electrophoretic mobility shift assay results under other conditions are shown in Figure S6. **(B–D)** Biolayer interferometry analysis of wt^*^ **(B)**, wt **(C)**, and K13ac **(D)** HU proteins. A representative sensorgram from triplicates is displayed here for each protein at 480, 192, 76.8, 30.7, and 12.3 nM. Asterisk (^*^) denotes protein without His-tag. The exact amino acid sequence of each protein construct is shown in Figure S4.

HU homologs are known to undergo sequence-independent DNA binding and regulation (Grove, 2011). Thus, electrophoretic mobility shift assay was performed to investigate if Lys13 acetylation affects DNA binding. A linear double-stranded DNA (dsDNA) from BamHI-digested plasmid, pBK AcKRS (Neumann et al., 2009), was employed as the binding partner. Mixture containing different amount of recombinant proteins was subjected to agarose gel electrophoresis. The binding of protein to DNA leads to a complex with larger molecular weight than that of DNA itself, hence a slower shift in mobility. Decrease of DNA mobility was observed in samples with higher amount of protein (Figure 4A). However, both wild-type and K13ac proteins showed similar degree of change in DNA mobility. In addition, this phenomenon is independent of DNA sequence or shape and composition of binding buffer (Figure S6). Notably, whereas high salt level (>300 mM NaCl) abolishes binding of *E. coli* HU to DNA (Xiao et al., 2010), the binding of *A. baumannii* HU to DNA remained up to 800 mM NaCl (Figure S6F).

To rule out the possibility that the observed DNA binding is due to the presence of C-terminal His-tag (Ceci et al., 2005), wild-type HU protein without a His-tag was also prepared. This protein was expressed with a TEV cleavage sequence between the HU sequence and N-terminal His-tag. SDS-PAGE and MS were used to monitor and confirm the TEV cleavage (Figure S4). This protein showed comparable DNA-binding ability to that of the C-terminally His-tagged wild-type HU (Figure 4A); accordingly, we concluded that binding of DNA by wt and K13ac HU is unlikely due to the His-tag.

To quantify the protein-DNA interaction, we used biolayer interferometry to study the binding kinetics (i.e. association and dissociation rate constants, *k*_on_ and *k*_off_) and the dissociation equilibrium constant *K*_D_. In the sensorgrams (Figures 4B–D), we noticed profound difference in the signal intensity between wild-type and K13ac proteins. Nevertheless, the *K*_D_ values calculated from experimental triplicate showed negligible difference among the modified and unmodified proteins (Table S5). However, both *k*_on_ and *k*_off_ values of HU(K13ac) are close to an order of magnitude smaller than those of the wild-type. Such difference in *k*_on_ likely explains the slow signal increase for the HU(K13ac) sample in comparison to the wild-type protein in the sensorgrams (Figures 4C,D). Thus, it is likely that Lys13 acetylation of free HU slows the step of DNA complexation, whereas acetylation of HU-DNA complex decreases the dissociation of DNA from the complex.

To examine the effect of acetylation on DNA architectural organization, atomic force microscopy was applied to visualize DNA-protein complexes at the stoichiometry of 10 bp/HU dimer. At this ratio, if all proteins bind to DNA, there will be one HU homodimer for every 10 bp DNA. This is the ratio we observed most profound interaction between DNA and HU proteins on electrophoretic mobility shift assay (Figure 4A and Figure S6). Samples in buffer solution (Ghosh et al., 2016) with or without pre-incubation at 37°C were deposited on freshly cleaved mica by spin coating, followed by imaging with the tapping mode (Figures S7–S15). Without pre-incubation, DNA seems to form bundles (Figure S8), and there is no significant morphology difference in samples containing HU proteins (Figures S9–S11). On the other hand, pre-incubating the sample solution at 37°C before depositing on mica resulted in well-separated DNA strands (Figure S12), and DNA strands appear significantly higher and wider in samples containing either wild-type or K13ac HU (Figures S13–S15), indicating binding of HU proteins to DNA strands. Nevertheless, DNA does not form compact aggregates with HU proteins, suggesting limited compaction ability of HU proteins under the experimental conditions.

## Discussion

*A. baumannii* is an opportunistic pathogen responsible for a majority of nosocomial infections. Symptoms include pneumonia, bloodstream infection and meningitis, and mortality rate can be as high as 70% (Antunes et al., 2014). Outbreaks of infection caused by multidrug-resistant *A. baumannii* strains in intensive care units have become a threat to the health care system. In fact, the World Health Organization recently ranked *A. baumannii* as one of the most threatening bacteria to human health (Willyard, 2017). Accordingly, protein post-translational modifications in *A. buamannii* warrant extensive investigations as better mechanistic understanding of bacterial physiology will shed light onto formatting new therapeutic strategies.

Lysine acetylation is a dynamic protein post-translational modification (Drazic et al., 2016). Recent progresses in proteomic research have revealed the acetylome of several bacterial species (Ouidir et al., 2016). In particular, acetylome of *A. baumannii* was recently reported in strain ATCC19606 at stationary phase, showing 551 lysine acetylation sites on 411 proteins (Kentache et al., 2016). In comparison, here we found 103 sites on 88 proteins in SK17-S and 67 sites on 60 proteins in SK17-R, including 25 sites on 22 proteins in both strains (Figures 1A,B). In both studies, a wide range of proteins was found to be acetylated, consistent with the argument that acetylation is an abundant post-translational modification. However, strikingly, there are only four identical sites among the previous and current studies. The drastic difference in the extent and substrate of acetylation can be attributed to (i) the different harvested stage as it was stationary phase in the previous study and mid-exponential phase in the current study, (ii) different *A. baumannii* strains investigated, and/or (iii) different anti-acetyllysine antibodies used for enrichment. Indeed, the use of different bacterial strains likely has a significant effect, as the two closely related strains, SK17-S and SK17-R, shared only <40% acetylation sites.

Consistent with other bacterial acetylome studies (Ouidir et al., 2016), the majority of acetylated proteins locate in the cytoplasm with putative function in metabolism (Figures 1C,D). Generally, acetylation of lysine changes the electrostatic state of a protein and increases the size of amino acid side chain. Such changes affect the biophysical properties of proteins and may serve as a regulatory mechanism for different biological processes. This has been well exemplified in the studies of eukaryotic histone proteins, where acetylation regulates gene transcription (Drazic et al., 2016). It is noteworthy that Lys13 of histone-like protein HU was found to be acetylated in both SK17-S and SK17-R as well as ATCC19606 (Kentache et al., 2016). Such a modification of HU has also been discovered in other bacteria (Figure 2B), indicating the prevalence and biological significance of this site-specific modification. However, there has been no characterization of the effect of site-specific acetylation in bacteria. In fact, the understanding of *A. baumannii* HU was limited to its amino acid sequence. Given that HU is an ubiquitous bacterial DNA-binding protein with essential function in several microbes, the role of Lys13 acetylation was thoroughly investigated in this work.

In general, HU homologs are typically homodimers, although heterodimers are common in enterobacteria (e.g. *E. coli*) that encode two homologs (Grove, 2011) and higher oligomers have been observed by mutation of HU (Kar et al., 2006). *A. baumannii* HU exists as a homodimer in solution and appears to be independent of its post-translational acetylation status (Figure 3C). The stability of HU has been assessed by the measurement of thermal melting temperature. HU from *B. subtilis* shows a melting temperature ranging from 33 to 48°C, depending on the salt, buffer, protein solutions, and other experimental conditions (Wilson et al., 1990; Welfle et al., 1992; Christodoulou and Vorgias, 2002). In this work, the K13ac variant was found to be less thermally stable than that of the unmodified HU under identical condition (Figure 3E). The effect of Lys13 acetylation on DNA binding appeared to significantly lower the association and dissociation rates toward DNA (Table S5). Similar to certain lysine residues found in eukaryotic histones (Shahbazian and Grunstein, 2007), Lys13 does not directly interact with DNA but its acetylation status can modulate DNA binding. Hence, it is likely that acetylation serves as an epigenetic regulator in bacteria by controlling the availability of free HU protein for DNA binding and the dissociation of DNA from HU-DNA complexes (Figure S16). The fast DNA-association kinetics of the unmodified HU protein indicates that the unmodified HU protein is required to form HU-DNA complexes. If DNA needs to dissociate from the complexes, HU proteins in the complexes have also to be in the non-acetylated state because complexes with acetylated HU have significantly lower DNA dissociation kinetics. On the other hand, acetylation of free HU proteins will preserve the pool of free HU protein due to the slow DNA-binding kinetics of acetylated HU. Similarly, due to the slow DNA-dissociation kinetics, acetylated HU-DNA complexes would remain in the DNA-bound state and preserve HU-DNA complexes. Given that acetylation is a dynamic and reversible process, cells can finely regulate the DNA-binding event by protein acetylation. Lastly, either wild-type or K13ac HU does not seem to compact DNA under our experimental conditions (Figures S7–S15), in contrast to previous studies of HU proteins in *E. coli* (Kar et al., 2006) and *M. tuberculosis* (Ghosh et al., 2016).

HU protein and its post-translational modification possess several characteristics as attractive targets for novel antimicrobial treatments. HU binds to DNA in a sequence-independent manner and plays critical roles in cell survival and pathogenicity (Micka and Marahiel, 1992; Bartels et al., 2001; Liu et al., 2008; Nguyen et al., 2009; Alberti-Segui et al., 2010; Koli et al., 2011; Mangan et al., 2011; Wang et al., 2014; Phan et al., 2015). Introducing two mutations into *E. coli* HUα converts a commensal strain into an invasive form (Koli et al., 2011), so it is likely that post-translational modification of HU may exert similar effect. In addition, HU and its homologs are only found in bacteria but not in mammalian cells, so molecules targeting HU will likely have little off-target interactions in human, because the closest HU homologs in eukaryotes are only found in organelles such as chloroplast and plastid of plant cells but not mitochondria (Grove, 2011). This principle can be applied to design molecules that bind to HU which prevent either its interaction with DNA (Bhowmick et al., 2014) or its post-translational modification. Such molecules may modulate the transcription-activation profile of pathogen and eliminate the virulence without killing the bacteria, thereby preventing the emergence of drug resistance (Dickey et al., 2017). With the ever-growing problems in antimicrobial resistance, new therapeutics is urgently needed. We believe that further investigation of *A. baumannii* HU and its post-translational modifications would represent a promising solution to address the problem.

## Data availability

The LC-MS/MS data in this manuscript is deposited at the PRIDE proteomics data repository (http://www.ebi.ac.uk/pride/archive/, European Bioinformatics Institute, Cambridge, UK) with the data set identifier PXD007289.

## Author contributions

Conceptualization: S-HW, Y-HT; Data curation: S-HW, Y-HT, J-HL, J-TY; Formal analysis: J-HL, C-HT, SP, J-TY, ML; Funding acquisition: S-HW, Y-HT, LL; Investigation: J-HL, C-HT, SP, J-TY, ML, ML-L, W-LW, I-FT, W-JS, M-RH, C-CC, MLC, GS, HO; Methodology: J-HL, C-HT, SP, J-TY, HO; Project administration: S-HW, Y-HT; resources: S-HW, Y-HT, LL, HO; Supervision: S-HW, Y-HT; Manuscript preparation: Y-HT, LL, J-HL, S-HW.

### Conflict of interest statement

The authors declare that the research was conducted in the absence of any commercial or financial relationships that could be construed as a potential conflict of interest. The reviewer XDL and handling Editor declared their shared affiliation.

## Abbreviations

BSA: bovine serum albumin
CD: circular dichroism
dsDNA: double-stranded DNA
FDR: false discovery rate
IPTG: isopropyl β-D-thiogalactopyranoside
MS: mass spectrometry
NJ: neighbor join
TB: terrific broth.
