# Supplementary material for: Acetylome of Acinetobacter baumannii SK17 Reveals a Highly-Conserved Modification of Histone-Like Protein HU
Source: Front Mol Biosci. 2017 Nov 27;4:77. doi: 10.3389/fmolb.2017.00077 (PMC5711770; doi:10.3389/fmolb.2017.00077)

Table S1. Lysine acetylation site found in both *A. baumannii* SK17-S and SK17-R.

Protein Group Accessions	Protein Description	Sequence	Confidence	ATCC 17978
470.191.peg.1037	Contig157_24634_25878 3-carboxymuconate cyclase	HMVFSPNG**kac**QAYITAEMR	High	
470.191.peg.1176	Contig168_1593_2234 Fumarylacetoacetate hydrolase family protein	DLQNEL**kac**DK	High	Y
470.191.peg.13	Contig1_8229_8996 FIG00350940: hypothetical protein	GGKGTGGGFQGGG**kac**GGAPTGMK	Medium	
470.191.peg.1307	Contig175_18025_19329 DcaP	LGLDFNTPVGDD**kac**VGGK	Medium	
470.191.peg.1331	Contig18_12932_14128 Dihydrolipoamide succinyltransferase component (E2) of 2-oxoglutarate dehydrogenase complex (EC 2.3.1.61)	KQY**kac**DAFEK	High	
470.191.peg.1479	Contig193_2500_4002 filamentation induced by cAMP protein Fic	KGGHDVDED**kac**VK*	High	
470.191.peg.1865	Contig232_10208_10654 LSU ribosomal protein L9p	AGDEG**kac**LFGSIGTR	High	
470.191.peg.1976	Contig251_22308_24452 Phosphate acetyltransferase (EC 2.3.1.8)	**kac**GKLNDLQAK	High	Y
470.191.peg.2241	Contig41_9570_8092 D-serine/D-alanine/glycine transporter	EDQGP**kac**AFGKLNR	Medium	
470.191.peg.227	Contig112_8198_8962 Beta 1,4 glucosyltransferase	NNQYSNL**kac**ALEK	Medium	
470.191.peg.2279	Contig42_11710_12591 FIG00351805: hypothetical protein	**kac**RGSVSEKPK	Medium	
470.191.peg.2396	Contig47_8217_8489 DNA-binding protein HU	SELIDAIAE**kac**GGVSK	High	Y
470.191.peg.252	Contig112_35507_34794 Glycerophosphoryl diester phosphodiesterase (EC 3.1.4.46)	AVRDMALAE**kac**LIQK	Medium	
470.191.peg.2561	Contig54_6359_8554 TRAP-type C4-dicarboxylate transport system, large permease component	HPNIDAQV**kac**EQMK	High	
470.191.peg.2616	Contig6_23804_22917 Chromosome (plasmid) partitioning protein ParB / Stage 0 sporulation protein J	DWSEP**kac**QE**kac**EK	High	
470.191.peg.2692	Contig67_3550_3050 FIG00351518: hypothetical protein	VQGE**kac**ALEDLK	High	
470.191.peg.2836	Contig73_16322_17938 Probable conserved transmembrane protein	FMPA**kac**T**kac**ENSK	High	
470.191.peg.2868	Contig75_21794_22951 2-methylcitrate synthase (EC 2.3.3.5)	A**kac**LKALR	High	
470.191.peg.2991	Contig85_8947_7877 Outer membrane protein A precursor	VAE**kac**LSEYPNATAR	High	
		LSTQGFAWDQPIADN**kac**TK	High	
470.191.peg.300	Contig114_13490_14449 Phenylacetic acid degradation operon negative regulatory protein PaaX	NDWLCSD**kac**IGR	Medium	
470.191.peg.723	Contig139_20080_19049 Arginine N-succinyltransferase (EC 2.3.1.109)	RENQNG**kac**FLSK	Medium	
470.191.peg.852	Contig148_548_78 FIG00351502: hypothetical protein	LDMSLMP**kac**YKNK	High	

* Peptide GGHDVDEDkacVK was identified in SK17-R with high confidence.

Table S2. Lysine acetylation site found only in *A. baumannii* SK17-S.

Protein Group Accessions	Protein Description	Sequence	Confidence	ATCC 17978
470.191.peg.1002	Contig156_23370_24188 FIG00350748: hypothetical protein	LSVYSEYMQTLLQSQHSLHK**kac**QIA	Medium	
470.191.peg.1007	Contig156_29632_28895 FIG00351138: hypothetical protein	S**kac**DSSRFSINPVNADDSVPQGTNTK	Medium	
470.191.peg.1063	Contig158_18927_18631 LSU ribosomal protein L25p	AEDKQG**kac**GASR	Medium	
470.191.peg.113	Contig104_5919_5050 FIG00349932: hypothetical protein	MELQGICH**kac**MHAGLK	Medium	
470.191.peg.1132	Contig163_29742_30359 Predicted transcriptional regulator LiuX of leucine degradation pathway, AcrR family	FDHAH**kac**DYFS**kac**QIADFCVDAVQK	Medium	
470.191.peg.1211	Contig170_6767_6204 Cyclohexanone monooxygenase (EC 1.14.13.22)	SDSYYTALQRANC**kac**LQTWPIVK	High	
470.191.peg.1225	Contig170_21037_20459 NAD(P)H-flavin oxidoreductase	LDDPQ**kac**LEHCGVELFYQKDLTSPLVSGCIAWLVCK	High	
470.191.peg.1263	Contig173_6283_7617 GTP-binding protein HflX	QAVQECLMGQLQHFSLVL**kac**PAYGKLR	Medium	
470.191.peg.1324	Contig18_5227_3953 Citrate synthase (si) (EC 2.3.3.1)	MLDEIGSVENVAEFME**kac**VK	High	
470.191.peg.1331	Contig18_12932_14128 Dihydrolipoamide succinyltransferase component (E2) of 2-oxoglutarate dehydrogenase complex (EC 2.3.1.61)	MIDG**kac**EAVGFLVAIK	High	
470.191.peg.1342	Contig180_5378_4476 putative ATP/GTP-binding protein	FGLQINFYGFSQQEYLKTVQYWLNEY**kac**WQQR	Medium	
470.191.peg.1381	Contig185_3192_1714 Aspartyl-tRNA(Asn) amidotransferase subunit A (EC 6.3.5.6) @ Glutamyl-tRNA(Gln) amidotransferase subunit A (EC 6.3.5.7)	QPASFCGLTGL**kac**PTYGR	Medium	
470.191.peg.1410	Contig186_21638_22459 Dihydrodipicolinate reductase (EC 1.3.1.26)	YDM**kac**DVLGLNDVQV	Medium	
470.191.peg.1437	Contig188_2299_35 TonB-dependent receptor; Outer membrane receptor for ferrienterochelin and colicins	GMGPENTLILVDG**kac**PINSRNSVR	Medium	
470.191.peg.1448	Contig19_9429_11063 Heat shock protein 60 family chaperone GroEL	SIAKPADDF**kac**AIEQVGSISANSDTTVGK	High	
470.191.peg.1479	Contig193_2500_4002 filamentation induced by cAMP protein Fic	MTQ**kac**NELDTK	High	Y
470.191.peg.1551	Contig201_639_1595 DnaJ-class molecular chaperone CbpA	E**kac**DAEE**kac**MQAINVAYDTLSNPEK	Medium	
470.191.peg.1558	Contig201_13879_10181 Exodeoxyribonuclease V beta chain (EC 3.1.11.5)	HVSGTIFRNGAFN**kac**LFSECLPQLLQVLK	Medium	
470.191.peg.1615	Contig21_4982_4602 putative signal peptide	GNGYKVDY**kac**DNNLP**kac**PDR	Medium	
470.191.peg.1707	Contig219_16579_14054 Outer membrane protein assembly factor YaeT precursor	E**kac**MAASLEALR	Medium	
470.191.peg.1712	Contig219_21307_20753 Ribosome recycling factor	E**kac**EISEDDERR	Medium	
470.191.peg.1713	Contig219_22121_21393 Uridylate kinase (EC 2.7.4.-)	LGVMDLTAICLCRDHNVPLQVFDMN**kac**SGALLSVVMGEK	Medium	
470.191.peg.1714	Contig219_23516_22173 Ribosomal protein S12p Asp88 (E. coli) methylthiotransferase	LI**kac**IK	High	
470.191.peg.1733	Contig22_9195_8263 4-hydroxyproline epimerase (EC 5.1.1.8)	SPCGTGTSA**kac**IACLAADGK	Medium	
470.191.peg.1734	Contig22_10008_9364 Predicted regulator PutR for proline utilization, GntR family	QDEIA**kac**LFNVSKIPVR	Medium	
470.191.peg.1776	Contig221_44818_43409 FIG00608533: hypothetical protein	S**kac**TAETSSTENQIR	High	

Protein Group Accessions	Protein Description	Sequence	Confidence	ATCC 17978
470.191.peg.1803	Contig224_13401_11638 Phosphodiesterase/alkaline phosphatase D	FGNKVSPVGQT**kac**TLPTSTNK	Medium	
470.191.peg.1811	Contig224_19937_19350 FIG00350330: hypothetical protein	AVSTKATQV**kac**EVVAEK	Medium	
470.191.peg.1833	Contig23_2901_3539 TniB NTP-binding protein	ASDPEA**kac**LRNQAVHLMR	Medium	
470.191.peg.1836	Contig23_5576_4725 Universal stress protein	MSKIIACIDGSLVTNTVCDYAAWFSD**kac**LNSPIK	Medium	
470.191.peg.1837	Contig23_7076_5589 Sulfate permease	EC**kac**GQGVANVASGFLGGMAGCAMIGQSIINV**kac**SGGR	Medium	
470.191.peg.1846	Contig230_864_124 hypothetical protein	LFMLF**kac**TDEFDIE**kac**QEFFK	Medium	
470.191.peg.1914	Contig25_2928_2011 Permease of the drug/metabolite transporter (DMT) superfamily	LFSSQ**kac**NCEKQA	High	
470.191.peg.1982	Contig251_31338_30004 Cell division trigger factor (EC 5.2.1.8)	QVEKP**kac**LPEIDAEFLK	Medium	
470.191.peg.2053	Contig3_18190_18882 Hypothetical protein YggS, proline synthase co-transcribed bacterial homolog PROSC	DGCAPEDVAELVAQMSQLP**kac**IK	Medium	
470.191.peg.2065	Contig3_32907_34892 Gamma-glutamyltranspeptidase (EC 2.3.2.2)	YMVVANTPLAV**kac**AGCDVLK	High	
470.191.peg.2101	Contig32_18364_16538 FIG00351490: hypothetical protein	MGQL**kac**ILELR	Medium	
470.191.peg.2119	Contig34_5891_6109 FIG00352022: hypothetical protein	NLVEHENEPL**kac**LSQR	High	
470.191.peg.218	Contig111_34274_36511 Isocitrate dehydrogenase [NADP] (EC 1.1.1.42); Monomeric isocitrate dehydrogenase [NADP] (EC 1.1.1.42)	VALQEGEIIDSMFMS**kackac**ALCDFYEK	High	
470.191.peg.2230	Contig40_80_1147 DUF1176 domain-containing protein	**kac**GSANENQVLMPEPK	High	
470.191.peg.2241	Contig41_9570_8092 D-serine/D-alanine/glycine transporter	EDQGPKAFG**kac**LNR	Medium	
470.191.peg.229	Contig112_10895_10005 FIG00350869: hypothetical protein	YQQAK**kac**AK	High	
		IDSTWYMIPESSAN**kac**TIELWKCTDFPMK	High	
470.191.peg.2332	Contig45_73_3858 Phage tail length tape-measure protein 1	RADNDIAIA**kac**QALR	Medium	
470.191.peg.2484	Contig52_10670_9786 Ribose-phosphate pyrophosphokinase (EC 2.7.6.1)	ICAVGQAFSLDVMT**kac**LLNINADK	Medium	
470.191.peg.2596	Contig6_8370_6697 SSU ribosomal protein S1p	LADEVEATL**kac**ASEINR	High	
470.191.peg.2693	Contig67_3837_5900 Methionyl-tRNA synthetase (EC 6.1.1.10)	EAGNIAVRPVTQLFDPE**kac**GMFLSDR	High	
470.191.peg.2836	Contig73_16322_17938 PROBABLE CONSERVED TRANSMEMBRANE PROTEIN	VCEQGYERPDIMFSH**kac**HNGTICSLCK	High	
470.191.peg.2920	Contig79_20208_19204 Dihydroorotate dehydrogenase (EC 1.3.3.1)	MGFNNDGVD**kac**LIENVKASK	High	
470.191.peg.2988	Contig85_4648_6108 Lysine-specific permease	NKT**kac**LIPYDQMDVKPEQD	Medium	
470.191.peg.2989	Contig85_6385_7590 Protein RtcB	GATVGSVIPT**kac**HAIIPAAVGVDIGCGMNAIRLSLK	Medium	
470.191.peg.3015	Contig91_103_660 hypothetical protein	NSISN**kac**SINK	Medium	
470.191.peg.3021	Contig91_5379_4519 Short-chain dehydrogenase/reductase SDR	KPISPEYMAQAIV**kac**GVQR	Medium	
470.191.peg.3078	Contig99_3760_4611 transcriptional regulator, LysR family	FGRP**kac**DDYLIAR	Medium	
470.191.peg.312	Contig116_1189_98 ABC-type multidrug transport system, permease component	LIFLATIGVIFLITAYSRL**kac**VFK	Medium	
470.191.peg.369	Contig119_6564_5869 ortholog of Bordetella pertussis (BX470248) BP2475	DSSLSKEQQ**kac**LLK	Medium	

Protein Group Accessions	Protein Description	Sequence	Confidence	ATCC 17978
470.191.peg.383	Contig119_25850_25224 Superoxide dismutase [Fe] (EC 1.15.1.1)	ETLEYHHD**kac**HHNTYVVNLNNLIK	High	
470.191.peg.412	Contig120_2240_102 Translation elongation factor G	EFFGAVD**kac**GIQER	High	
470.191.peg.451	Contig122_12416_13300 Aldehyde dehydrogenase (EC 1.2.1.3)	VVFALDT**kac**GQQWLLR	Medium	
470.191.peg.519	Contig127_7069_8442 UDP-N-acetylmuramate:L-alanyl-gamma-D-glutamyl-meso-diaminopimelate ligase (EC 6.3.2.-)	ACEALSNFGGV**kac**R	Medium	
470.191.peg.522	Contig127_10813_11769 Integral membrane protein TerC	FL**kac**GQEEETNIEDIKLLK	Medium	
470.191.peg.553	Contig128_903_496 Gfa-like protein	SDLEIVQGENLLQAYFASPN**kac**KR	Medium	
470.191.peg.564	Contig128_12431_13693 benzoate-specific porin	FTL**kac**NAYINRDFDNDALK	Medium	
470.191.peg.569	Contig129_5437_3209 Helicase PriA essential for oriC/DnaA-independent DNA replication	KGLVDCTLEPHDFSPSPVQLAQMPLTLNEDQ**kac**K	High	
470.191.peg.629	Contig132_11281_9203 type IV pilus biogenesis protein PilJ	LAERSASAT**kac**QIETLVK	Medium	
470.191.peg.635	Contig133_4973_3300 High-affinity choline uptake protein BetT	NDSLEESPPE**kac**SSLQVIIEK	High	
470.191.peg.657	Contig134_8982_9626 FIG00350418: hypothetical protein	YYSGADGT**kac**LLVLDDDENR	Medium	
470.191.peg.711	Contig139_6603_8672 Glycyl-tRNA synthetase beta chain (EC 6.1.1.14)	TLPEAAALAAAN**kac**R	Medium	
470.191.peg.843	Contig147_13098_12508 TolA protein	**kac**WMVQVALAPNQNK	High	
470.191.peg.893	Contig152_4020_6302 Ferrichrome-iron receptor	FDQGTFAHTLSLFEI**kackac**PSAYTDLVTNIYTSGGEQR	Medium	
470.191.peg.905	Contig152_22296_21049 PROTEIN ERFK/SRFK	GLYYTRVSEMLGE**kac**FHIDEAFLK	Medium	

Table S3. Lysine acetylation site found only in *A. baumannii* SK17-R.

Protein Group Accessions	Protein Description	Sequence	Confidence
470.191.peg.1003	Contig156_24307_25602 Acyl-CoA dehydrogenase; probable dibenzothiophene desulfurization enzyme	KYYTTGSLYADWVDVGVTDL**kac**GESGSVVVR	High
470.191.peg.1026	Contig157_11877_10309 3-ketoacyl-CoA thiolase (EC 2.3.1.16) @ Acetyl-CoA acetyltransferase (EC 2.3.1.9)	MS**kac**TTQENPAVENSAQEK	Medium
470.191.peg.1027	Contig157_12113_13504 3-oxoacyl-[acyl-carrier protein] reductase (EC 1.1.1.100)	IVCVSSISGIAGNLGQTNYAAS**kac**AGVIGLVKFTAPILK	Medium
470.191.peg.1038	Contig157_26179_26682 Low molecular weight protein tyrosine phosphatase (EC 3.1.3.48)	**kac**RGYDLSSLR	Medium
470.191.peg.1187	Contig168_12785_11334 tRNA-i(6)A37 methylthiotransferase	TDAMLG**kac**IER	Medium
470.191.peg.1241	Contig171_4318_5211 LysR-family transcriptional regulator clustered with PA0057	SNDVNALYDMCLHAQGVAMLPTLLVE**kac**DLK	Medium
470.191.peg.1286	Contig174_13273_10175 RND multidrug efflux transporter; Acriflavin resistance protein	MTVEEFPNIDFPFVVVTTQYAGASPEAVESDIT**kac**K	Medium
470.191.peg.1389	Contig185_9930_8545 Wax ester synthase/acyl-CoA:diacylglycerol acyltransferase	**kac**PLIAMVPASLRTDDSDVSNRITMILANLGTHK	Medium
470.191.peg.1408	Contig186_19876_20988 Chaperone protein DnaJ	**kac**TITFTAPAPCDVCDGK	Medium
470.191.peg.1543	Contig20_9673_10818 Aldose 1-epimerase (EC 5.1.3.3)	FSLDG**kac**TYNLEK	Medium
470.191.peg.1646	Contig212_61_345 hypothetical protein	SIAAMVNGMCKQCG**kac**EPLQAIVSDK	Medium
470.191.peg.2009	Contig263_318_40 FIG00350892: hypothetical protein	MLD**kac**LLLLQPCLY**kac**K	Medium
470.191.peg.2109	Contig33_443_2989 Nitrite reductase [NAD(P)H] large subunit (EC 1.7.1.4)	CT**kac**AATACGGCAPLVTQVLK	Medium
470.191.peg.2158	Contig35_4517_9214 FIG00350631: hypothetical protein	GYLFIDS**kac**GR	Medium
470.191.peg.2177	Contig38_9085_7736 Permeases of the major facilitator superfamily	GQQLNQSGQVDQVKFTQCLQFI**kac**K	High
470.191.peg.2229	Contig4_31958_30366 lipid A phosphoethanolamine transferase	QE**kac**ASRLLLK	Medium
470.191.peg.2410	Contig5_1566_2231 Ribosomal large subunit pseudouridine synthase A (EC 4.2.1.70)	HYIALVQGQV**kac**QEGSVEAPLITDWENRPR	Medium
470.191.peg.2423	Contig5_15931_16821 3-hydroxyisobutyrate dehydrogenase (EC 1.1.1.31)	NIVHCGDVGAGQIA**kac**ICNNLILGISMAAVAEGMALGVK	Medium
470.191.peg.25	Contig1_16328_16879 FIG00351865: hypothetical protein	LYHELLMDACAAHLIEDW**kac**GVVFAEIVDGK	Medium
470.191.peg.2546	Contig53_39489_41306 Translation elongation factor LepA	MAQA**kackac**SVDIK	Medium
470.191.peg.2596	Contig6_8370_6697 SSU ribosomal protein S1p	AKDEAEE**kac**EAVANLR	High
470.191.peg.2662	Contig62_11813_10323 FIG076926: outer membrane protein	AEWMHFISADKAF**kac**LEAESK	Medium
470.191.peg.2730	Contig68_306_1892 Cytochrome d ubiquinol oxidase subunit I (EC 1.10.3.-)	EVTGL**kac**DLMQQHEVR	Medium
470.191.peg.2792	Contig7_5508_5711 FIG00354137: hypothetical protein	MDNS**kac**LPINQIIARINDAAK	Medium
470.191.peg.2802	Contig70_8382_8921 FIG027190: Putative transmembrane protein	AELWSWR**kac**TPEGR	Medium
470.191.peg.2807	Contig70_14414_13581 hypothetical protein	QELDIE**kac**CGVSSLCVHPGGIR	Medium

Protein Group Accessions	Protein Description	Sequence	Confidence
470.191.peg.2845	Contig74_4385_3702 ATP phosphoribosyltransferase (EC 2.4.2.17)	C**kac**LMTAG**kac**VGMERPK	Medium
470.191.peg.2851	Contig75_1005_2108 Poly(glycerol-phosphate) alpha-glucosyltransferase (EC 2.4.1.52)	LEE**kac**VILLGR	Medium
470.191.peg.2897	Contig78_5828_4458 Biotin carboxylase of acetyl-CoA carboxylase (EC 6.3.4.14)	MLQ**kac**VLIANR	Medium
470.191.peg.2933	Contig8_3951_4577 merops peptidase family S24	QVDLALATG**kac**SK	Medium
470.191.peg.2992	Contig85_9428_9844 putative type 4 fimbrial biogenesis protein FimT	MASVEL**kac**NTPR	Medium
470.191.peg.3067	Contig98_286_1530 Beta-lactamase (EC 3.5.2.6)	IVDDL**kac**VSLGALK	Medium
470.191.peg.376	Contig119_18623_17082 Di-/tripeptide transporter	QEANSCS**kac**PVVEN**kac**NAPK	Medium
470.191.peg.452	Contig122_13616_13732 hypothetical protein	TGVLANHMEG**kac**SGLGSLSSSVLVQHAPG	Medium
470.191.peg.555	Contig128_3879_1408 Lead, cadmium, zinc and mercury transporting ATPase (EC 3.6.3.3) (EC 3.6.3.5); Copper-translocating P-type ATPase (EC 3.6.3.4)	VE**kac**ALK	Medium
470.191.peg.764	Contig141_11916_11380 nucleoprotein/polynucleotide-associated enzyme	ANIE**kac**AQK	Medium
470.191.peg.884	Contig151_6218_8974 Aconitate hydratase (EC 4.2.1.3)	LAIYDAAMRYQQEHTPLVIIAG**kac**EYGTGSSR	Medium
470.191.peg.964	Contig154_21357_22694 K(+)-uptake protein KtrB, integral membrane subunit; Potassium uptake protein TrkH	GITPDLHNGSLFILSLLMFAGRLGPLTLAYLIATP**kac**K	Medium

Table S4. Cellular localization and putative function of the acetylated proteins found in *A. baumannii* SK17-S and SK17-R.

Accession Number	Cellular Localization	Putative Function
470.191.peg.1002	cytoplasm	unknown
470.191.peg.1003	cytoplasm	unknown
470.191.peg.1007	outer membrane	unknown
470.191.peg.1026	cytoplasm	metabolism (amino acid)
470.191.peg.1027	cytoplasm	metabolism (energy)
470.191.peg.1037	unknown	unknown
470.191.peg.1038	cytoplasm	unknown
470.191.peg.1063	cytoplasm	translation
470.191.peg.113	cytoplasm	unknown
470.191.peg.1132	cytoplasm	transcription regulation
470.191.peg.1176	cytoplasm	metabolism (energy)
470.191.peg.1187	cytoplasm	metabolism (nucleotide)
470.191.peg.1211	cytoplasm	unknown
470.191.peg.1225	unknown	metabolism (energy)
470.191.peg.1241	cytoplasm	unknown
470.191.peg.1263	cytoplasm	unknown
470.191.peg.1286	cytoplasmic membrane	virulence & defense
470.191.peg.13	cytoplasm	unknown
470.191.peg.1307	outer membrane	unknown
470.191.peg.1324	cytoplasm	metabolism (carbohydrate)
470.191.peg.1331	cytoplasm	metabolism (carbohydrate)
470.191.peg.1342	cytoplasm	unknown
470.191.peg.1381	cytoplasm	metabolism (protein)
470.191.peg.1389	cytoplasm	metabolism (energy)
470.191.peg.1408	cytoplasm	metabolism (protein)
470.191.peg.1410	cytoplasm	metabolism (amino acid)
470.191.peg.1437	outer membrane	membrane transport
470.191.peg.1448	cytoplasm	metabolism (protein)
470.191.peg.1479	cytoplasm	unknown
470.191.peg.1543	periplasmic	metabolism (carbohydrate)
470.191.peg.1551	cytoplasm	metabolism (protein)
470.191.peg.1558	cytoplasm	metabolism (nucleotide)
470.191.peg.1615	unknown	unknown
470.191.peg.1646	unknown	unknown
470.191.peg.1707	outer membrane	unknown
470.191.peg.1712	cytoplasm	metabolism (protein)
470.191.peg.1713	cytoplasm	unknown
470.191.peg.1714	cytoplasm	metabolism (nucleotide)
470.191.peg.1733	cytoplasm	metabolism (amino acid)
470.191.peg.1734	cytoplasm	metabolism (amino acid)
470.191.peg.1776	outer membrane	unknown
470.191.peg.1803	extracellular	unknown
470.191.peg.1811	cytoplasm	unknown
470.191.peg.1833	cytoplasm	unknown
470.191.peg.1836	cytoplasm	stress response
470.191.peg.1837	cytoplasmic membrane	metabolism (amino acid)
470.191.peg.1846	cytoplasm	unknown
470.191.peg.1865	cytoplasm	translation
470.191.peg.1914	cytoplasmic membrane	unknown
470.191.peg.1976	cytoplasm	metabolism (carbohydrate)
470.191.peg.1982	cytoplasm	metabolism (protein)
470.191.peg.2009	unknown	unknown
470.191.peg.2053	cytoplasm	metabolism (amino acid)
470.191.peg.2065	periplasmic	metabolism (amino acid)

Accession Number	Cellular Localization	Putative Function
470.191.peg.2101	unknown	unknown
470.191.peg.2109	cytoplasm	metabolism (energy)
470.191.peg.2119	cytoplasm	unknown
470.191.peg.2158	outer membrane	unknown
470.191.peg.2177	cytoplasmic membrane	membrane transport
470.191.peg.218	cytoplasm	metabolism (carbohydrate)
470.191.peg.2229	cytoplasmic membrane	unknown
470.191.peg.2230	periplasmic	unknown
470.191.peg.2241	cytoplasmic membrane	metabolism (carbohydrate)
470.191.peg.227	cytoplasm	metabolism (energy)
470.191.peg.2279	unknown	unknown
470.191.peg.229	cytoplasm	unknown
470.191.peg.2332	outer membrane	transposable & extrachromosomal element
470.191.peg.2396	cytoplasm	metabolism (nucleotide)
470.191.peg.2410	cytoplasm	metabolism (nucleotide)
470.191.peg.2423	cytoplasm	metabolism (amino acid)
470.191.peg.2484	cytoplasm	metabolism (carbohydrate)
470.191.peg.25	cytoplasm	unknown
470.191.peg.252	cytoplasm	metabolism (carbohydrate)
470.191.peg.2546	cytoplasmic membrane	translation
470.191.peg.2561	cytoplasmic membrane	membrane transport
470.191.peg.2596	cytoplasm	translation
470.191.peg.2616	cytoplasm	cellular process
470.191.peg.2662	outer membrane	cellular process
470.191.peg.2692	unknown	unknown
470.191.peg.2693	cytoplasm	metabolism (protein)
470.191.peg.2730	cytoplasmic membrane	cellular process
470.191.peg.2792	unknown	unknown
470.191.peg.2802	unknown	unknown
470.191.peg.2807	cytoplasm	unknown
470.191.peg.2836	cytoplasmic membrane	unknown
470.191.peg.2845	cytoplasm	metabolism (amino acid)
470.191.peg.2851	cytoplasm	cell wall & capsule
470.191.peg.2868	cytoplasm	metabolism (carbohydrate)
470.191.peg.2897	cytoplasm	metabolism (energy)
470.191.peg.2920	cytoplasmic membrane	metabolism (nucleotide)
470.191.peg.2933	unknown	metabolism (protein)
470.191.peg.2988	cytoplasmic membrane	membrane transport
470.191.peg.2989	cytoplasm	metabolism (nucleotide)
470.191.peg.2991	outer membrane	stress response
470.191.peg.2992	cytoplasm	unknown
470.191.peg.300	unknown	metabolism (energy)
470.191.peg.3015	cytoplasm	unknown
470.191.peg.3021	cytoplasm	stress response
470.191.peg.3067	unknown	virulence & defense
470.191.peg.3078	cytoplasm	transcription regulation
470.191.peg.312	cytoplasmic membrane	membrane transport
470.191.peg.369	cytoplasm	unknown
470.191.peg.376	cytoplasmic membrane	membrane transport
470.191.peg.383	periplasmic	stress response
470.191.peg.412	cytoplasm	metabolism (protein)
470.191.peg.451	cytoplasm	metabolism (carbohydrate)
470.191.peg.452	unknown	unknown
470.191.peg.519	cytoplasm	cell wall & capsule
470.191.peg.522	cytoplasmic membrane	unknown
470.191.peg.553	cytoplasm	unknown
470.191.peg.555	cytoplasmic membrane	virulence & defense

Accession Number	Cellular Localization	Putative Function
470.191.peg.564	outer membrane	metabolism (energy)
470.191.peg.569	cytoplasm	metabolism (nucleotide)
470.191.peg.629	outer membrane	unknown
470.191.peg.635	cytoplasmic membrane	unknown
470.191.peg.657	unknown	unknown
470.191.peg.711	cytoplasm	metabolism (protein)
470.191.peg.723	cytoplasm	metabolism (amino acid)
470.191.peg.764	cytoplasm	unknown
470.191.peg.843	cytoplasm	membrane transport
470.191.peg.852	unknown	unknown
470.191.peg.884	cytoplasm	metabolism (carbohydrate)
470.191.peg.893	outer membrane	membrane transport
470.191.peg.905	unknown	cell wall & capsule
470.191.peg.964	cytoplasmic membrane	metabolism (energy)

Table S5. Lysine acetylation site found in both *A. baumannii* SK17-S and SK17-R.

Protein Sequence	Presence of His tag	k_{on} (× 10^4 M^{-1} s^{-1})	k_{off} (× 10^{-3} s^{-1})	K_D (nM)
wild-type	No	8.53 ± 2.39	22.2 ± 12.0	276 ± 143
wild-type	C terminus	11.7 ± 5.5	21.6 ± 9.2	349 ± 352
K13ac	C terminus	2.60 ± 1.43	3.92 ± 0.74	184 ± 9.3

Note: Data analysis were performed using ForteBio Data Analysis Software with a 1:1 Langmuir binding model. Values shown here are average ± standard deviation calculated from experimental triplicate.

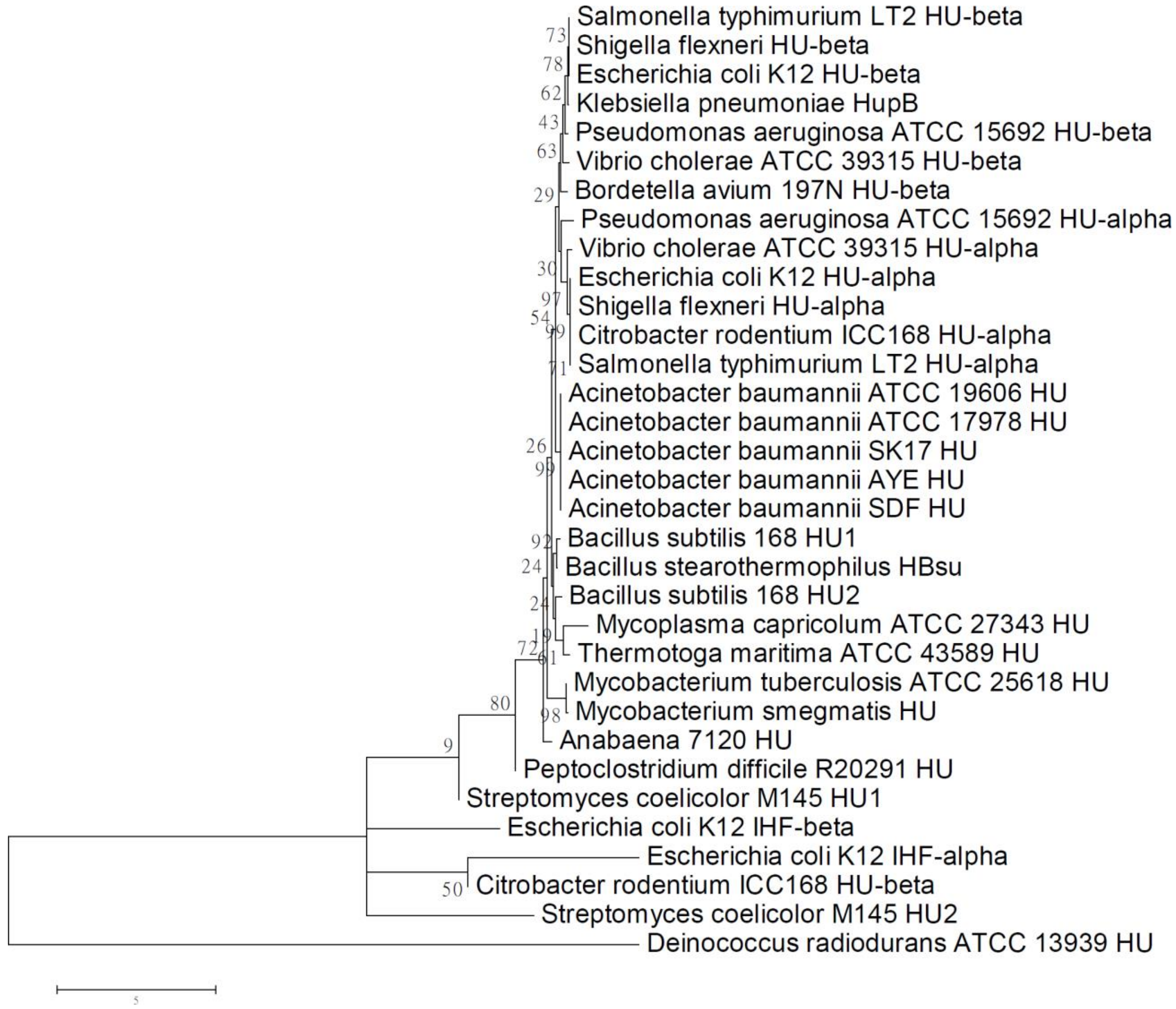

Figure S1. Phylogenetic tree of HU homologs.

```
A._baumannii_SK17_HU            ---------------------------------MNKSELIDAIAEKGGVSKTDAGKALDA
A._baumannii_ATCC19606_HU       ---------------------------------MNKSELIDAIAEKGGVSKTDAGKALDA
A._baumannii_ATCC17978_HU       ---------------------------------MNKSELIDAIAEKGGVSKTDAGKALDA
A._baumannii_AYE_HU             ---------------------------------MNKSELIDAIAEKGGVSKTDAGKALDA
A._baumannii_SDF_HU             ---------------------------------MNKSELIDAIAEKGGVSKTDAGKALDA
P._aeruginosa_PA14_HUβ          ---------------------------------MNKSELIDAIAASADIPKAVAGRALDA
E._coli_K12_HUβ                 ---------------------------------MNKSQLIDKIAAGADISKAAAGRALDA
S. flexneri Huβ                 ---------------------------------MNKSQLIDKIAAGADISKAAAGRALDA
S._typhimurium_LT2_Huβ          ---------------------------------MNKSQLIEKIAAGADISKAAAGRALDA
C. rodentium ICC168 HUβ         ----------------------------MRKRRVNKSQLIDKIAAGADISKAAAGRALDA
K. pneumoniae_HupB              ---------------------------------MNKSQLIDKIAAGADISKAAAGRALDA
V. cholerae_ATCC39315_Huβ       ---------------------------------MNKTQLVEQIAANADISKASAGRALDA
B. avium_197N_Huβ               ---------------------------------MNKTELIDHIASKADISKAAAGRTLDA
V. cholerae_ATCC39315_Huα       ---------------------------------MNKTQLIDFIAEKADLTKVQAKAALEA
E._coli_K12_HUα                 ---------------------------------MNKTQLIDVIAEKAELSKTQAKAALES
S. flexneri Huα                 ---------------------------------MNKTQLIDVIAEKAELSKTQAKAALES
C. rodentium ICC168 Huα         ---------------------------------MNKTQLIDVIADKAELSKTQAKAALES
S. typhimurium LT2 HUα          ---------------------------------MNKTQLIDVIADKAELSKTQAKAALES
B. stearothermophilus HBsu      ---------------------------------MNKTELINAVAETSGLSKKDATKAVDA
B. subtilis 168 HU1             ---------------------------------MNKTELINAVAEASELSKKDATKAVDS
B. subtilis 168 HU2             ---------------------------------MNKTELIAKVAEKQGVSKKEGAPSVEK
T. maritima ATCC43589 HU        ---------------------------------MTKKELIDRVAKKAGAKKKDVKLILDT
P. difficile R20291 HU          ---------------------------------MNKAELVSKMAEKSGLTKKEAEAALNA
P._aeruginosa_PA14_HUα          ---------------------------------MRKPELAAAIAEKADLTKEQANRVLNA
V. parahaemolyticus Huβ         ---------------------------------MNKTQLVEQIAENADISKASAGRALDA
V. parahaemolyticus HU2         ---------------------------------MNKTQLIDFIAEKADLSKAQAKAALEA
G. kaustophilus HU              ---------------------------------MNKTELINAVAETSGLSKKDATKAVDA
T. thermophilus HB8 HU          ---------------------------MAAKKTVTKADLVDQVAQATGLKKKDVKAMVDA
Anabaena_7120_HU                ---------------------------------MNKGELVDAVAEKASVTKKQADAVLTA
S. coelicolor M145 HU1          ---------------------------------MNRSELVAALADRAEVTRKDADAVLAA
M. tuberculosis_H37Ra_HU        ---------------------------------MNKAELIDVLTQKLGSDRRQATAAVEN
M. smegmatis HU                 ---------------------------------MNKAELIDVLTTKMGTDRRQATAAVEN
S. coelicolor M145 HU2          ---------------------------------MNKAQLVEAIADKLG-GRQQAADAVDA
M. capricolum ATCC27343 HU      ---------------------------------MTKKELINKIALETEVSKKEVNLIVKK
D. radiodurans ATCC13939 HU     MTKKSTKAPAKKAAPAAKAAPAAKRGAAADSGKVAKTQLVEMVADQTGLTKKQSEEAISS
E._coli_K12_IHFα                -------------------------------MALTKAEMSEYLFDKLGLSKRDAKELVEL

A._baumannii_SK17_HU            TIASITEALKKGD-TVTLVGFGTFSVKERAARTGRNPK-TGEELQIKATKVPSFKAGKGL
A._baumannii_ATCC19606_HU       TIASITEALKKGD-TVTLVGFGTFSVKERAARTGRNPK-TGEELQIKATKVPSFKAGKGL
A._baumannii_ATCC17978_HU       TIASITEALKKGD-TVTLVGFGTFSVKERAARTGRNPK-TGEELQIKATKVPSFKAGKGL
A._baumannii_AYE_HU             TIASITEALKKGD-TVTLVGFGTFSVKERAARTGRNPK-TGEELQIKATKVPSFKAGKGL
A._baumannii_SDF_HU             TIASITEALKKGD-TVTLVGFGTFSVKERAARTGRNPK-TGEELQIKATKVPSFKAGKGL
P._aeruginosa_PA14_HUβ          VIESVTGALKAGD-SVVLVGFGTFAVKERAARTGRNPQ-TGKPIKIAAAKIPGFKAGKAL
E._coli_K12_HUβ                 IIASVTESLKEGD-DVALVGFGTFAVKERAARTGRNPQ-TGKEITIAAAKVPSFRAGKAL
S. flexneri HUβ                 IIASVTESLKEGD-DVALVGFGTFAVKERAARTGRNPQ-TGKEITIAAAKVPSFRAGKAL
S._typhimurium_LT2_Huβ          IIASVTESLKEGD-DVALVGFGTFAVKERAARTGRNPQ-TGKEITIAAAKVPSFRAGKAL
C. rodentium ICC168 HUβ         IIASVTESLKEGD-DVALVGFGTFSVKERAARTGRNPQ-TGKEITIAAAKVPGFRAGKAL
K. pneumoniae_HupB              LIASVTESLQAGD-DVALVGFGTFAVKERAARTGRNPQ-TGKEITIAAAKVPGFRAGKAL
V. cholerae_ATCC39315_Huβ       FIEAVSGTLQSGD-QVALVGFGTFSVRTRAARTGRNPK-TGEEIKIAEAKVPSFKAGKAL
B. avium_197N_Huβ               LIGAVKTTLKKGG-TVTLVGFGTFAVSSRAARTGRNPR-TGETIKIKKAKVPKFRPGKAL
V. cholerae_ATCC39315_Huα       TLGAVEGALKDGD-QVQLIGFGTFKVNHRSARTGRNPK-TGEEIKIAAANVPAFVAGKAL
E._coli_K12_HUα                 TLAAITESLKEGD-AVQLVGFGTFKVNHRAERTGRNPQ-TGKEIKIAAANVPAFVSGKAL
S. flexneri HUα                 TLAAITESLKEGD-AVQLVGFGTFKVNHRAERTGRNPQ-TGKEIKIAAANVPAFVSGKAL
C. rodentium ICC168 HUα         TLAAITESLKEGD-AVQLVGFGTFKVNHRAERTGRNPQ-TGKEIKIAAANVPAFVSGKAL
S. typhimurium LT2 HUα          TLAAITESLKEGD-AVQLVGFGTFKVNHRAERTGRNPQ-TGKEIKIAAANVPAFVSGKAL
B. stearothermophilus HBsu      VFDSITEALRKGD-KVQLIGFGNFEVRERAARKGRNPQ-TGEEMEIPASKVPAFKPGKAL
B. subtilis 168 HU1             VFDTILDALKNGD-KIQLIGFGNFEVRERSARKGRNPQ-TGEEIEIPASKVPAFKPGKAL
B. subtilis 168 HU2             VFDTISEALKSGE-KVSIPGFGTFEVRERAARKGRNPQ-TGEEIDIPATKAPAFKPAKAL
T. maritima ATCC43589 HU        ILETITEALAKGE-KVQIVGFGSFEVRKAAARKGVNPQ-TRKPITIPERKVPKFKPGKAL
P. difficile R20291 HU          FMSSVQDALVNNE-KVQLVGFGTFETRERAARQGRNPRDPEQVIDIPASKAPVFKAGKGL
P._aeruginosa_PA14_HUα          LLDEITGALNRKD-SVTLVGFGTFLQRHRGARTGKNPQ-TGQPVKIKASNTVAFKPGKAL
V. parahaemolyticus Huβ         FIEAVSGTLQSGD-QVALVGFGTFSVRTRAARTGRNPK-TGEEIQIAEAKVPAFKAGKAL
V. parahaemolyticus HU2         TLEGVTGALKEGD-QVQLIGFGTFKVNHRAARTGRNPK-TGDEIQIAAANVPAFVAGKAL
G. kaustophilus HU              VFDSITEALRKGD-KVQLIGFGNFEVRERAARKGRNPQ-TGEEMEIPASKVPAFKPGKAL
T. thermophilus HB8 HU          LLAKVEEALANGS-KVQLTGFGTFEVRKRKARTGVKPG-TKEKIKIPATQYPAFKPGKAL
Anabaena_7120_HU                ALETIIEAVSSGD-KVTLVGFGSFESRERKAREGRNPK-TNEKMEIPATRVPAFSAGKLF
S. coelicolor M145 HU1          FAEVVGDIVSKGDEKVTIPGFLTFERTHRAARTARNPQ-TGEPIQIPAGYSVKVSAGSKL
M. tuberculosis_H37Ra_HU        VVDTIVRAVHKGD-SVTITGFGVFEQRRRAARVARNPR-TGETVKVKPTSVPAFRPGAQF
M. smegmatis HU                 VVDTIVRAVHKGD-SVTITGFGVFEQRRRAARVARNPR-TGETVKVKPTSVPAFRPGAQF
S. coelicolor M145 HU2          VLDALVRAVVAGD-RVSVTGFGSFEKVDRPARYARNPQ-TGERVRVKKTSVPRFRAGQGF
M. capricolum ATCC27343 HU      VFELISKNLLDKK-EVLISDFGKFTILQKVSRKGVNPI-TRQKMVIPASKTVKFKPSKQL
D. radiodurans ATCC13939 HU     MLECVVSALQSGK-SVGLPGLGTLSVKETAARTGVRPG-TSEKIQIPAGKKVAFKVASTL
E._coli_K12_IHFα                FFEEIRRALENGE-QVKLSGFGNFDLRDKNQRPGRNPK-TGEDIPITARRVVTFRPGQKL
```

```
A._baumannii_SK17_HU          KDSVA-------------------------------------------------------
A._baumannii_ATCC19606_HU     KDSVA-------------------------------------------------------
A._baumannii_ATCC17978_HU     KDSVA-------------------------------------------------------
A._baumannii_AYE_HU           KDSVA-------------------------------------------------------
A._baumannii_SDF_HU           KDSVA-------------------------------------------------------
P._aeruginosa_PA14_HUβ        KDAVN-------------------------------------------------------
E._coli_K12_HUβ               KDAVN-------------------------------------------------------
S._flexneri_HUβ               KDAVN-------------------------------------------------------
S._typhimurium_LT2_Huβ        KDAVN-------------------------------------------------------
C._rodentium_ICC168_HUβ       KDAVN-------------------------------------------------------
K._pneumoniae_HupB            KDAVN-------------------------------------------------------
V._cholerae_ATCC39315_Huβ     KDACN-------------------------------------------------------
B._avium_197N_Huβ             KDAVN-------------------------------------------------------
V._cholerae_ATCC39315_Huα     KDAIK-------------------------------------------------------
E._coli_K12_HUα               KDAVK-------------------------------------------------------
S._flexneri_HUα               KDAVK-------------------------------------------------------
C. rodentium ICC168 HUα       KDAVK-------------------------------------------------------
S. typhimurium LT2 HUα        KDAVK-------------------------------------------------------
B. stearothermophilus HBsu    KDAVK-------------------------------------------------------
B. subtilis 168 HU1           KDAVAGK-----------------------------------------------------
B. subtilis 168 HU2           KDAVKAK-----------------------------------------------------
T. maritima ATCC43589 HU      KEKVK-------------------------------------------------------
P. difficile R20291 HU        KDIING------------------------------------------------------
P._aeruginosa_PA14_HUα        RDAVN-------------------------------------------------------
V. parahaemolyticus Huβ       KDACN-------------------------------------------------------
V. parahaemolyticus HU2       KEACND------------------------------------------------------
G. kaustophilus HU            KDAVK-------------------------------------------------------
T. thermophilus HB8 HU        KDKVKK------------------------------------------------------
Anabaena_7120_HU              REKVAPPKA---------------------------------------------------
S. coelicolor M145 HU1        KEAAKGK-----------------------------------------------------
M. tuberculosis_H37Ra_HU      KAVVSGAQRLPAEGPAVKRGVGASAAKKVAKKAPAKKATKAAKKAA-TKAPARKAATKAP
M. smegmatis HU               KAVISGAQKLPADGPAVKRGVTAGPAKKAAKKAP------AKKAAA-KKTATKAAAKKAP
S. coelicolor M145 HU2        KDLVSGSKKLPKNDIAVKKAPKGSLSGPPPTISKAAGKKAAAKKATGAAKKTTGAAKKTS
M. capricolum ATCC27343 HU    KELLIKE-----------------------------------------------------
D. radiodurans ATCC13939 HU   KGNL--------------------------------------------------------
E._coli_K12_IHFα              KSRVENASPKDE------------------------------------------------

A._baumannii_SK17_HU          ------------------------------------------------------------
A._baumannii_ATCC19606_HU     ------------------------------------------------------------
A._baumannii_ATCC17978_HU     ------------------------------------------------------------
A._baumannii_AYE_HU           ------------------------------------------------------------
A._baumannii_SDF_HU           ------------------------------------------------------------
P._aeruginosa_PA14_HUβ        ------------------------------------------------------------
E._coli_K12_HUβ               ------------------------------------------------------------
S._flexneri_HUβ               ------------------------------------------------------------
S._typhimurium_LT2_Huβ        ------------------------------------------------------------
C._rodentium_ICC168_HUβ       ------------------------------------------------------------
K._pneumoniae_HupB            ------------------------------------------------------------
V._cholerae_ATCC39315_Huβ     ------------------------------------------------------------
B._avium_197N_Huβ             ------------------------------------------------------------
V._cholerae_ATCC39315_Huα     ------------------------------------------------------------
E._coli_K12_HUα               ------------------------------------------------------------
S._flexneri_HUα               ------------------------------------------------------------
C. rodentium ICC168 HUα       ------------------------------------------------------------
S. typhimurium LT2 HUα        ------------------------------------------------------------
B. stearothermophilus HBsu    ------------------------------------------------------------
B. subtilis 168 HU1           ------------------------------------------------------------
B. subtilis 168 HU2           ------------------------------------------------------------
T. maritima ATCC43589 HU      ------------------------------------------------------------
P. difficile R20291 HU        ------------------------------------------------------------
P._aeruginosa_PA14_HUα        ------------------------------------------------------------
V. parahaemolyticus Huβ       ------------------------------------------------------------
V. parahaemolyticus HU2       ------------------------------------------------------------
G. kaustophilus HU            ------------------------------------------------------------
T. thermophilus HB8 HU        ------------------------------------------------------------
Anabaena_7120_HU              ------------------------------------------------------------
S. coelicolor M145 HU1        ------------------------------------------------------------
M. tuberculosis_H37Ra_HU      AKKAATKAPAKKAVKATKSPAKKVTKAVKKTAVKASVRKAATKAPAKKA-AAKRPATKAP
M. smegmatis HU               AKKAATKAPAKKAATKAPAKKAATKAPAKKAATKAPAKKAAAKAPAKKA-ATKAPAKKAA
S. coelicolor M145 HU2        AAAKKTTAKKTTGAAKTTAKKTTAKKSAAKTTTAAAKKTAAKKAPAKKATAKKAPAKKST
M. capricolum ATCC27343 HU    ------------------------------------------------------------
D. radiodurans ATCC13939 HU   ------------------------------------------------------------
E._coli_K12_IHFα              ------------------------------------------------------------
```

```
A._baumannii_SK17_HU            --------------
A._baumannii_ATCC19606_HU       --------------
A._baumannii_ATCC17978_HU       --------------
A._baumannii_AYE_HU             --------------
A._baumannii_SDF_HU             --------------
P._aeruginosa_PA14_HUβ          --------------
E._coli_K12_HUβ                 --------------
S. flexneri HUβ                 --------------
S._typhimurium_LT2_Huβ          --------------
C. rodentium ICC168 HUβ         --------------
K. pneumoniae_HupB              --------------
V. cholerae_ATCC39315_Huβ       --------------
B. avium_197N_Huβ               --------------
V. cholerae_ATCC39315_Huα       --------------
E._coli_K12_HUα                 --------------
S. flexneri HUα                 --------------
C. rodentium ICC168 HUα         --------------
S. typhimurium LT2 HUα          --------------
B. stearothermophilus HBsu      --------------
B. subtilis 168 HU1             --------------
B. subtilis 168 HU2             --------------
T. maritima ATCC43589 HU        --------------
P. difficile R20291 HU          --------------
P._aeruginosa_PA14_HUα          --------------
V. parahaemolyticus Huβ         --------------
V. parahaemolyticus HU2         --------------
G. kaustophilus HU              --------------
T. thermophilus HB8 HU          --------------
Anabaena_7120_HU                --------------
S. coelicolor M145 HU1          --------------
M. tuberculosis_H37Ra_HU        AKKATARRGRK---
M. smegmatis HU                 AKKAPAKKGRR---
S. coelicolor M145 HU2          ARKTTAKKATARKK
M. capricolum ATCC27343 HU      --------------
D. radiodurans ATCC13939 HU     --------------
E._coli_K12_IHFα                --------------
```

Figure S2. Sequence alignment of different bacterial HU homologs to *A. baumannii* SK17 HU. Residues in red indicate acetylation sites found in *A. baumannii* ATCC19606 (1), *B. subtilis* 168 (2), *E. coli* K12 (3) and BW25113 (4), *G. kaustophilus* (5), *M. tuberculosis* H37Ra (6,7), *P. aeruginosa* PA14 (8), *T. thermophilus* HB8 (9) and *V. parahaemolyticus* (10).

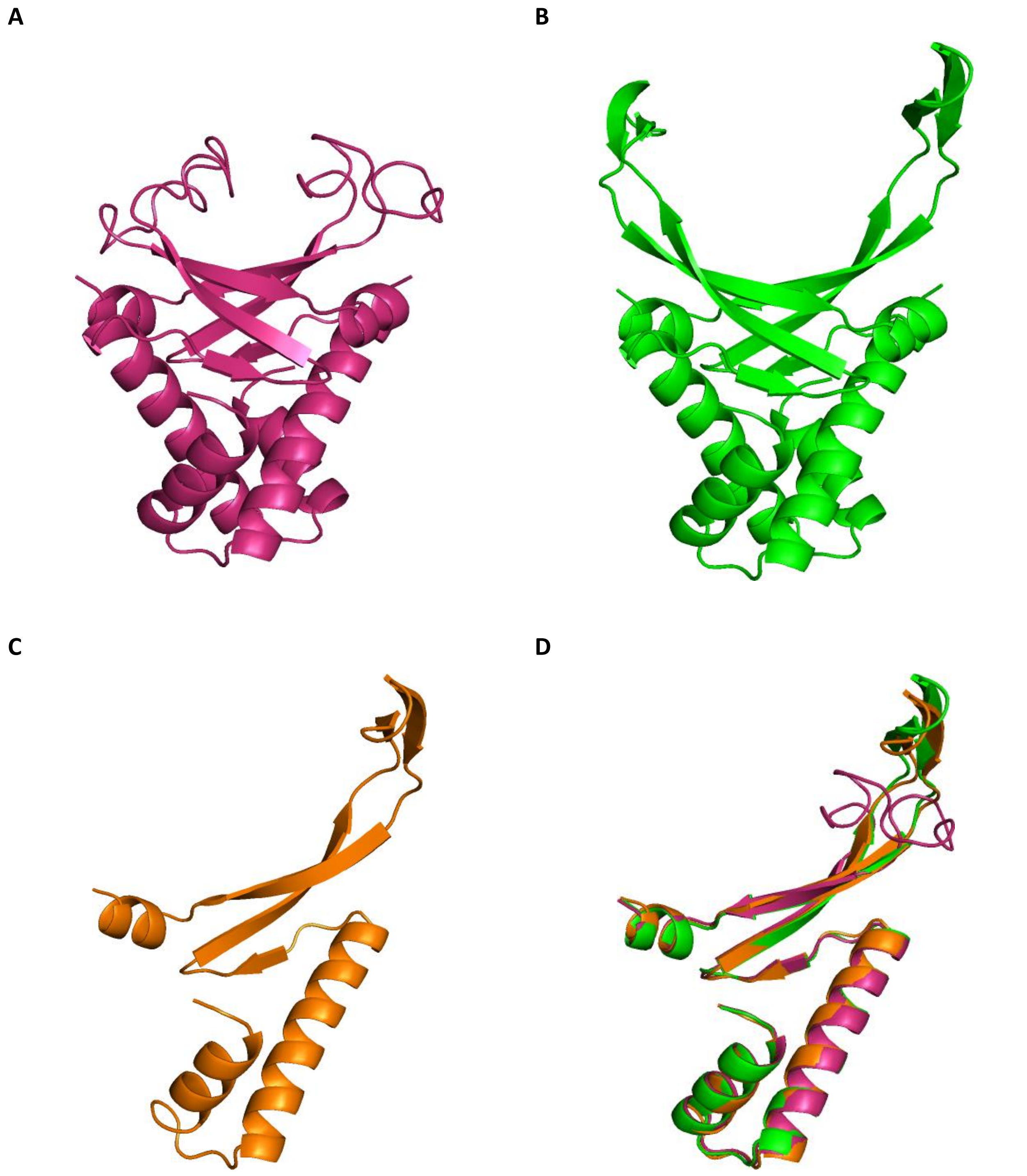

Figure S3. Homology models of *A. baumannii* HU. *A*. Homology model generated by SWISS-MODEL using pdb 4P3V as the template. *B*. Homology model generated by SWISS-MODEL using pdb 1P51 as the template. *C*. Homology model generated by I-TASSER. *D*. Alignment of one subunit of the three homology models.

A HU with C-terminal His tag

MNKSELIDAIAEKGGVSKTDAGKALDATIASITEALKKGDTVTLVGFGTFSVKERAARTGRNPKTGEELQIKATKVPSFKAGKGLKDSVALEHHHHHH (calculated MW = 10386.82)

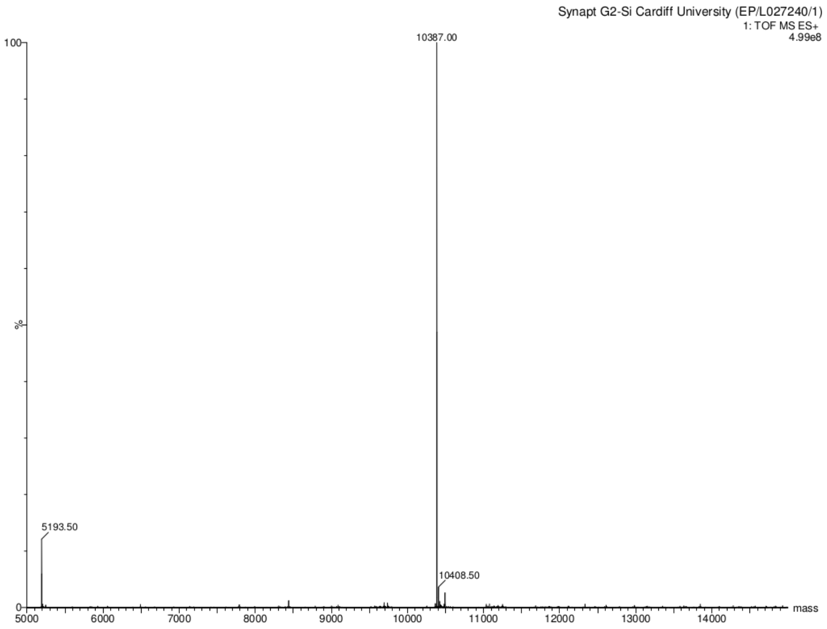

B HU(K13ac) with C-terminal His tag

MNKSELIDAIAE<u>Kac</u>GGVSKTDAGKALDATIASITEALKKGDTVTLVGFGTFSVKERAARTGRNPKTGEELQIKATKVPSFKAGKGLKDSVALEHHHHHH (calculated MW = 10428.86)

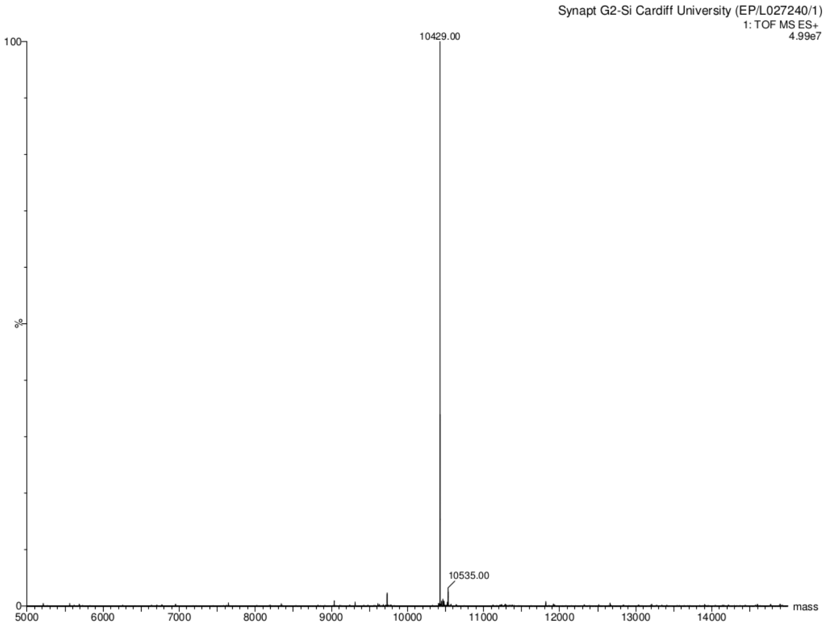

C LC chromatogram of peptides from tryptic digestion of HU and HU(K13ac)

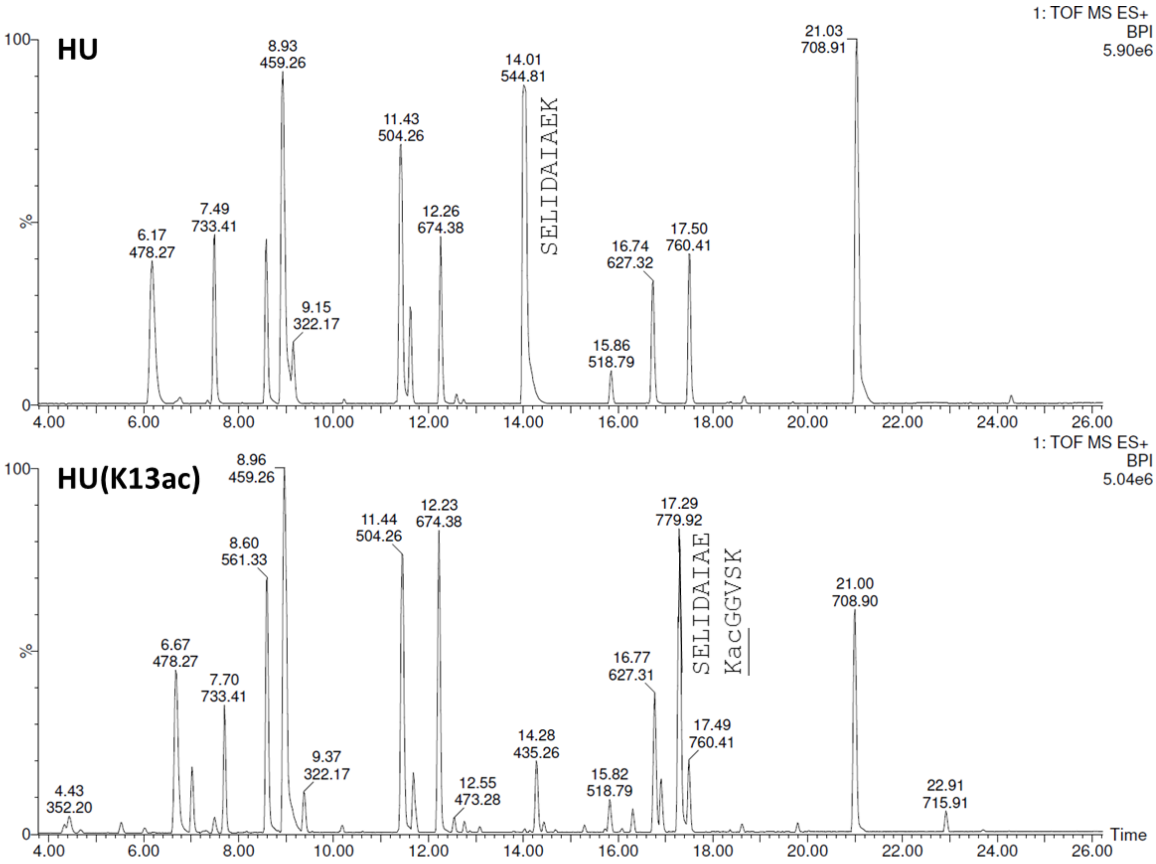

D HR-MS of SELIDAIAE<u>Kac</u>GGVSK peptide

Calculated mass for $C_{67}H_{116}N_{17}O_{25}$ = 1558.8328, observed mass = 1558.8398 (+ 4.5 ppm)

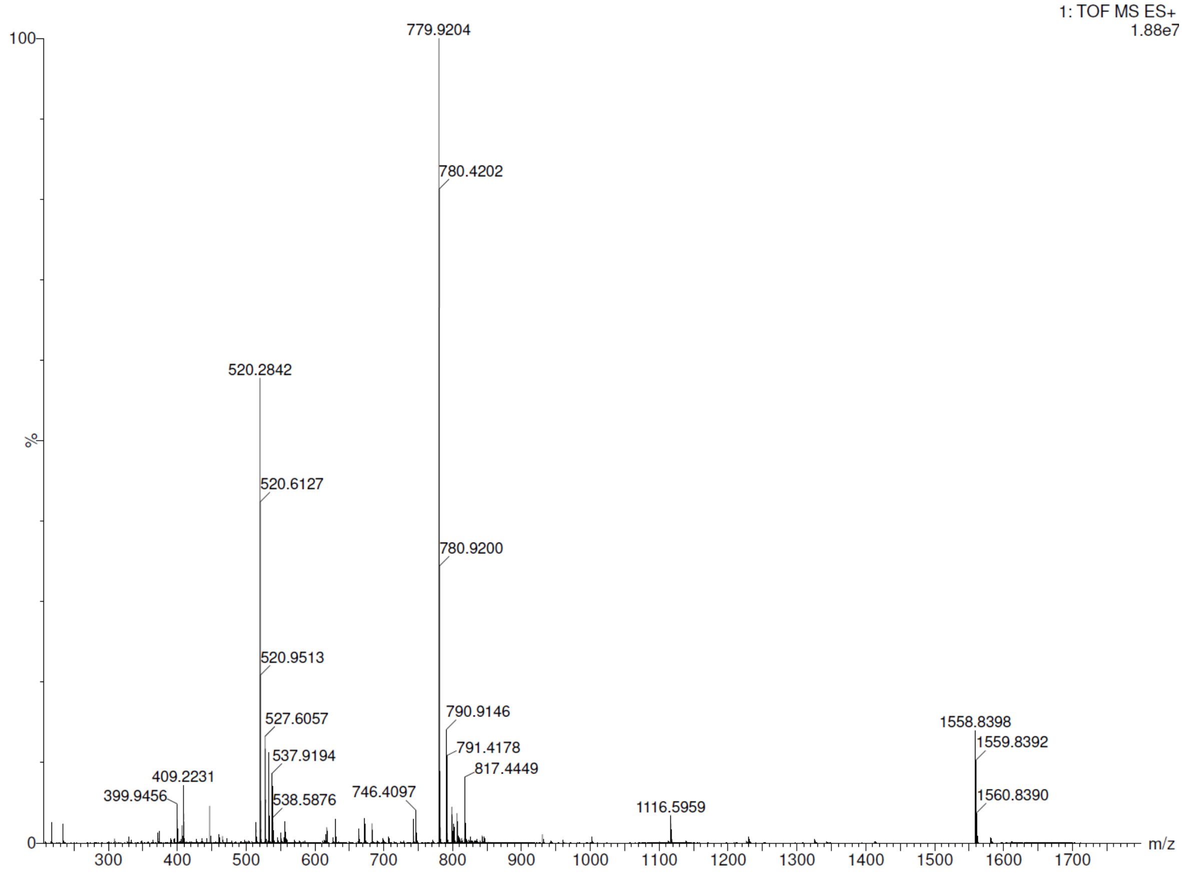

E MS/MS of SELIDAIAEKacGGVSK peptide

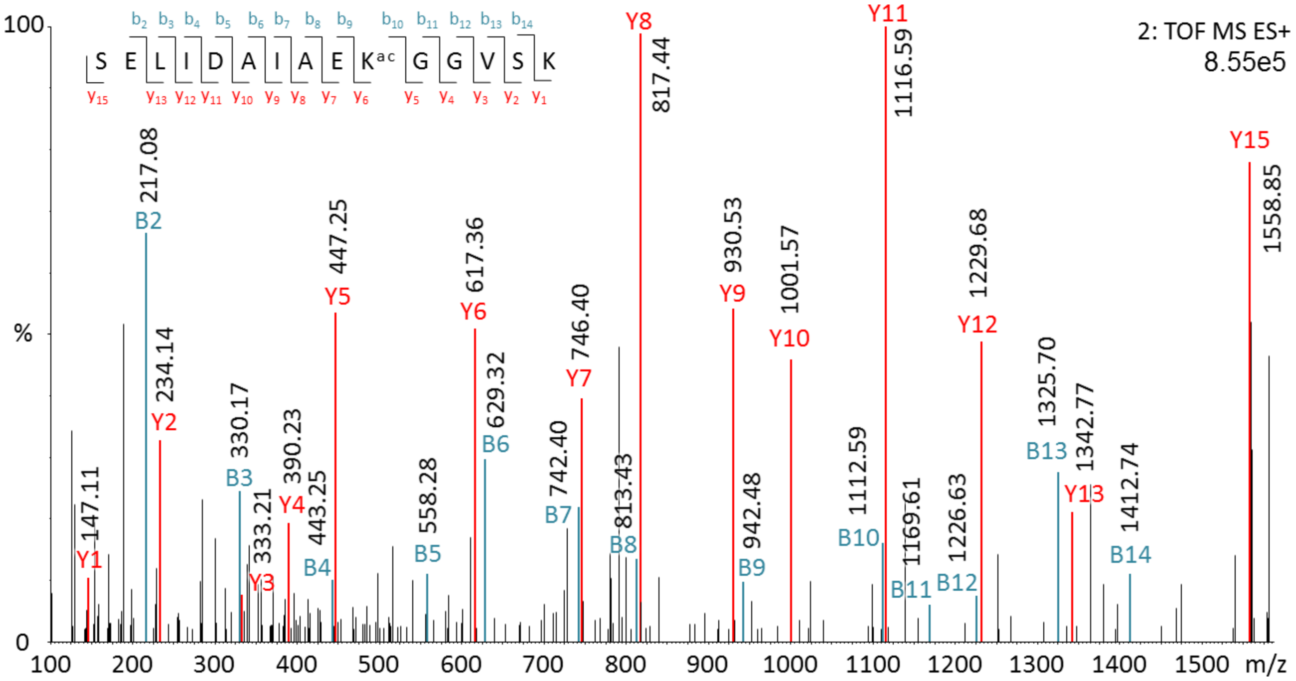

F HU with N-terminal His tag and TEV cleavage site

- Before TEV cleavage: MKHHHHHHPMSDYDIPTTENLYFQGAMNKSELIDAIAEKGGVSKTDAGKALD ATIASITEALKKGDTVTLVGFGTFSVKERAARTGRNPKTGEELQIKATKVPSFKAGKGLKDSVA (calculated MW = 12448.13)

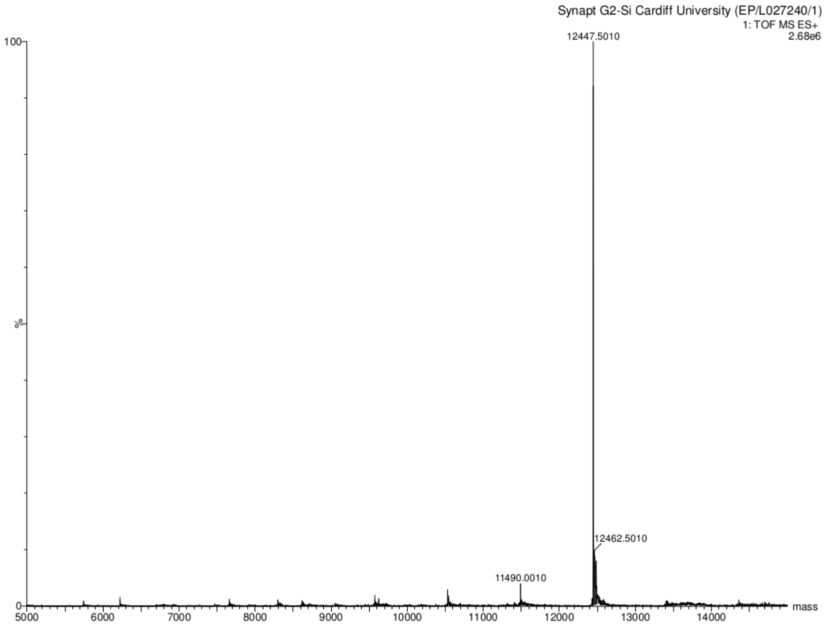

- After TEV cleavage GAMNKSELIDAIAEKGGVSKTDAGKALDATIASITEALKKGDTVTLVGFGTFSVKER AARTGRNPKTGEELQIKATKVPSFKAGKGLKDSVA (calculated MW = 9449.83)

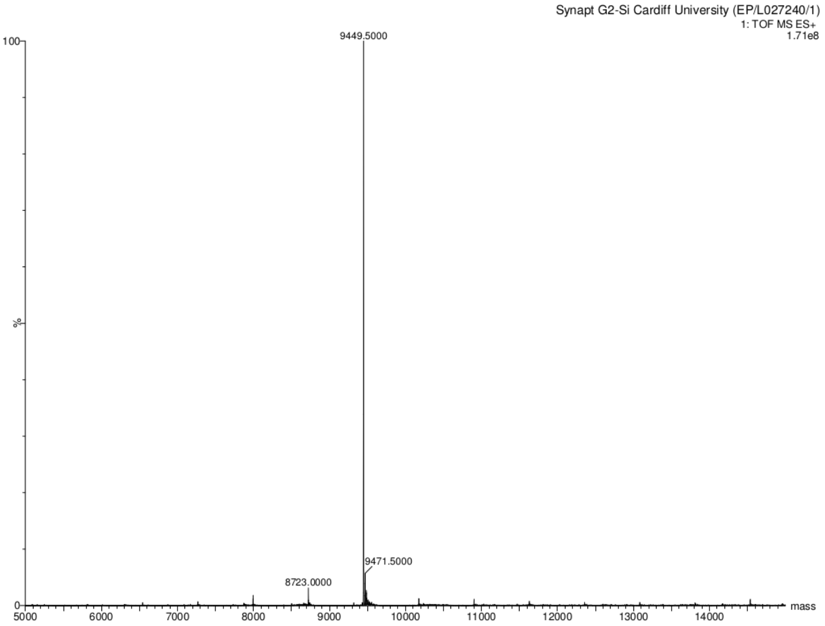

Figure S4. Amino acid sequence and mass spectrum of HU constructs. *A*, HU with C-terminal His tag. *B*, HU(K13ac) with C-terminal His tag. *C*, LC chromatogram of peptides from tryptic digestion of HU and HU(K13ac). *D*, HR-MS of SELIDAIAEKacGGVSK peptide. *E*, MS/MS of SELIDAIAEKacGGVSK peptide. *F*, HU with N-terminal His tag and TEV cleavage site. Amino acid sequence corresponds to HU, His-tag and TEV cleavage site is in blue, green and red, respectively. Acetylation site is underlined.

A Standard, run 1

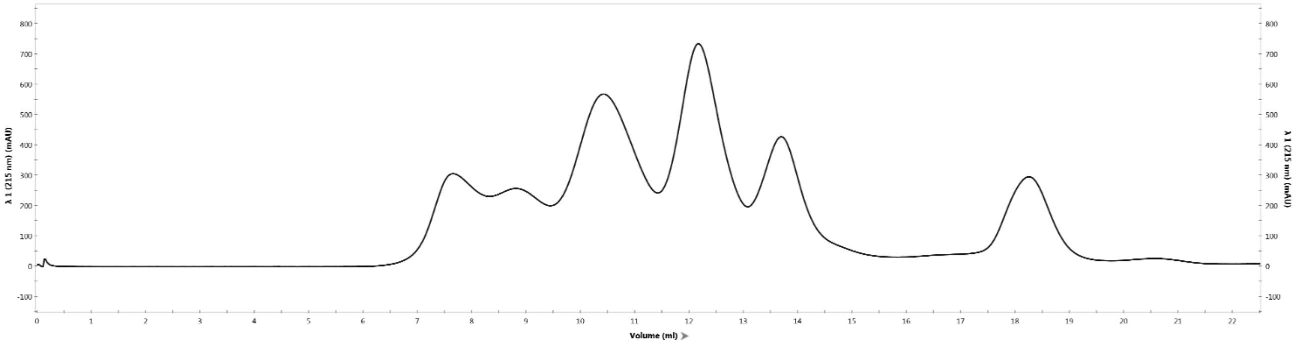

B Standard, run 2

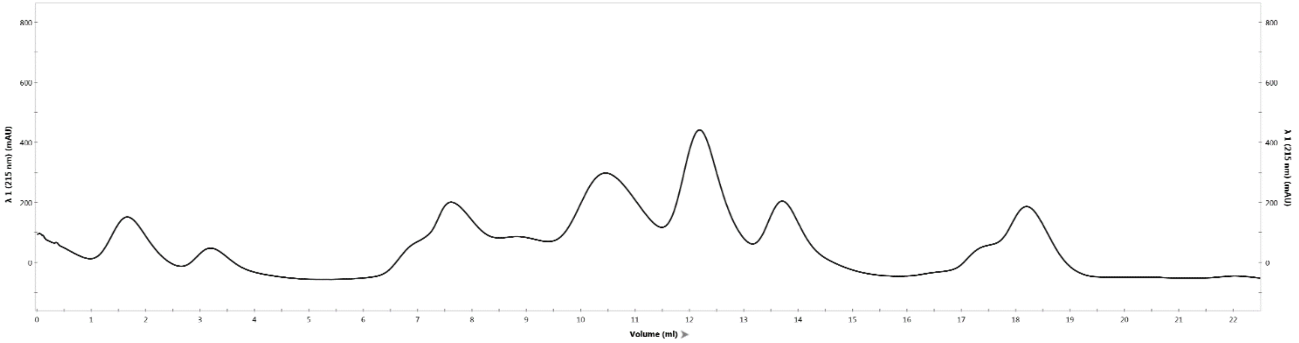

C HU, run 1

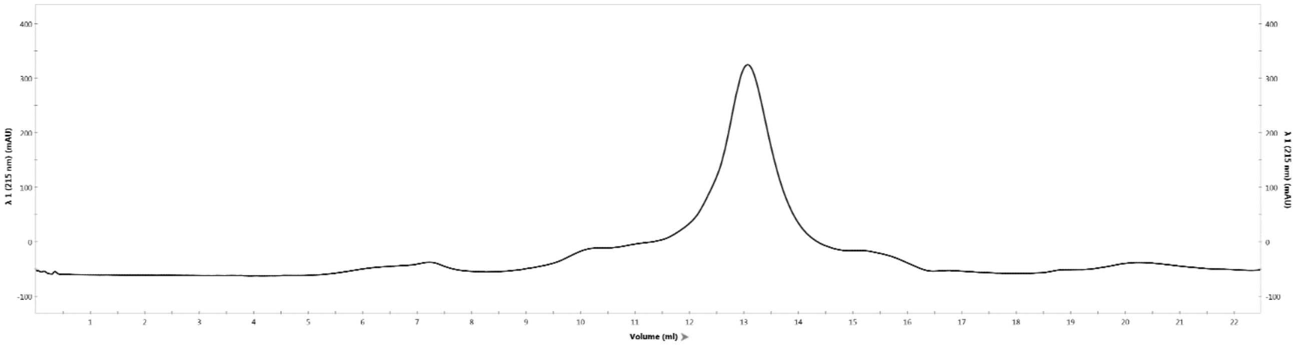

D HU, run 2

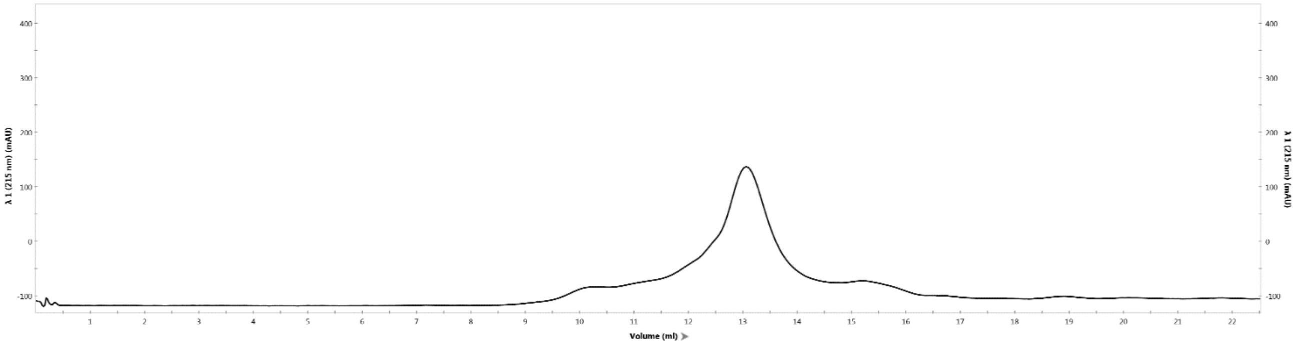

E HU(K13Ac), run 1

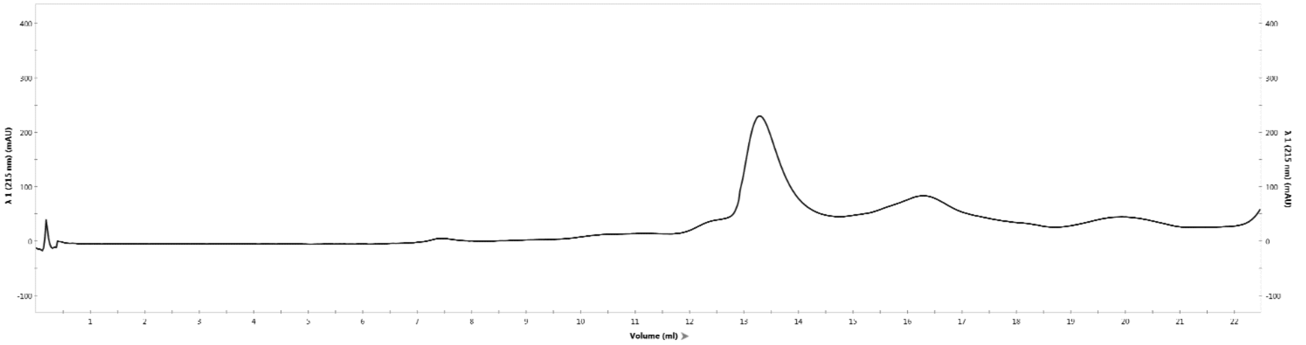

F HU(K13Ac), run 2

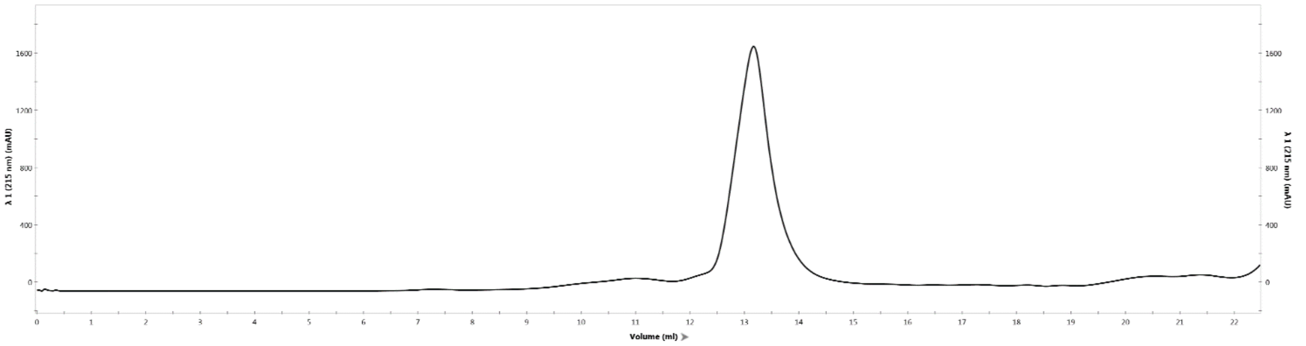

G Calibration curve

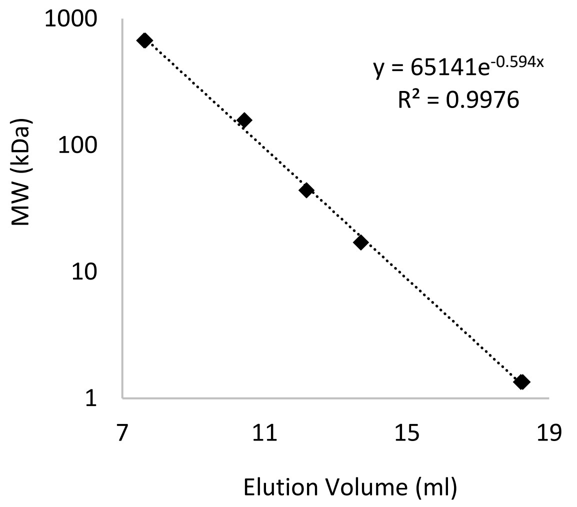

H

Protein	Elution Vol. (ml)	Estimated MW (kDa)	Theoretical MW (kDa)
HU	13.06 ± 0.01	27.8	10.4
HU(K13ac)	13.23 ± 0.06	25.2	10.4
BSA	11.53	69.1	66.5
DHFR	13.21	25.5	18.0
Myoglobin	13.86	17.3	17.0

Figure S5. Size exclusion chromatography indicated the existence of HU and HU(K13Ac) as homodimers. Duplicate runs of standard (*A*, *B*) wild-type HU (*C*, *D*) and acetylated HU (*E*, *F*) are shown. Calibration curve (*G*) was calculated from the two standard runs. MW of proteins was calculated using the calibration curve (*H*). Standard proteins (BSA, DHFR and myglobin) were analyzed under the same condition (spectra not shown) to validate the reliability of calibration curve.

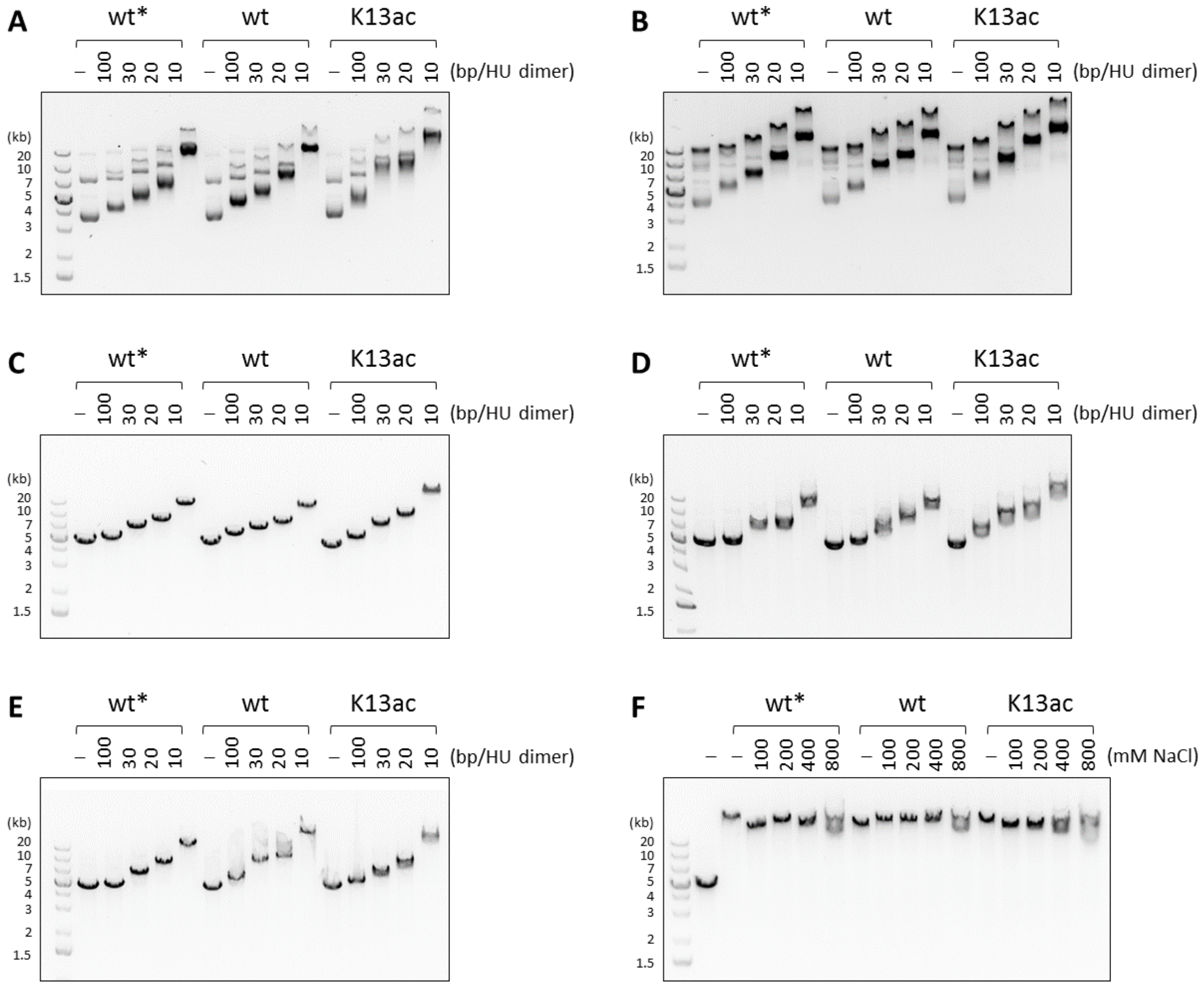

Figure S6. Electrophoretic mobility shift assay. DNA (250 ng) was incubated with the indicated amount of proteins in binding buffer at 37°C for 1 h before electrophoresed on a 1% agarose gel. Asterisk (*) denotes protein without His-tag. (*A* – *C*) Binding buffer: 30 mM Tris pH 7.5, 100 mM NaCl, 0.02% v/v Tween20, 0.5 mg/ml BSA. (*A*) DNA: pBK AckRS. (*B*) DNA: pCX GFP. (*C* – *F*) DNA: BcuI-treated linearized pCX GFP. (*D*) Binding buffer: 10 mM HEPES, pH 7.5, 4 mM $MgCl_2$. (*E*) Binding buffer: PBS pH 7.5. (*F*) Binding buffer: 30 mM Tris pH 7.5 with the indicated amount of NaCl.

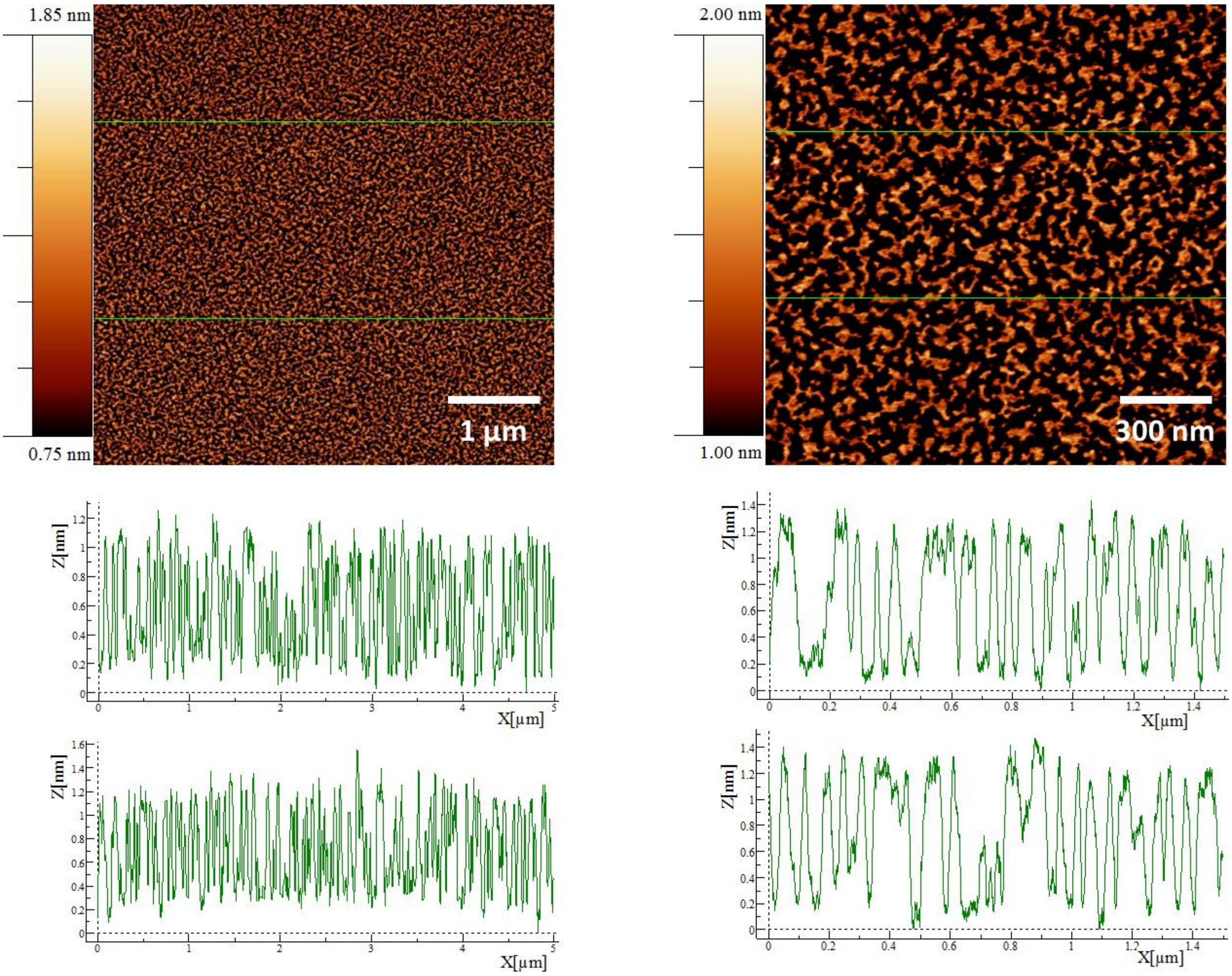

Figure S7. Atomic force microscopy images of wild-type HU protein with a C-terminal His tag without pre-incubation. HU protein (3 µM) in the buffer solution (10 mM HEPES, pH 7.5, 4 mM $MgCl_2$) without incubation at 37°C was deposited onto freshly cleaved mica by spin coating.

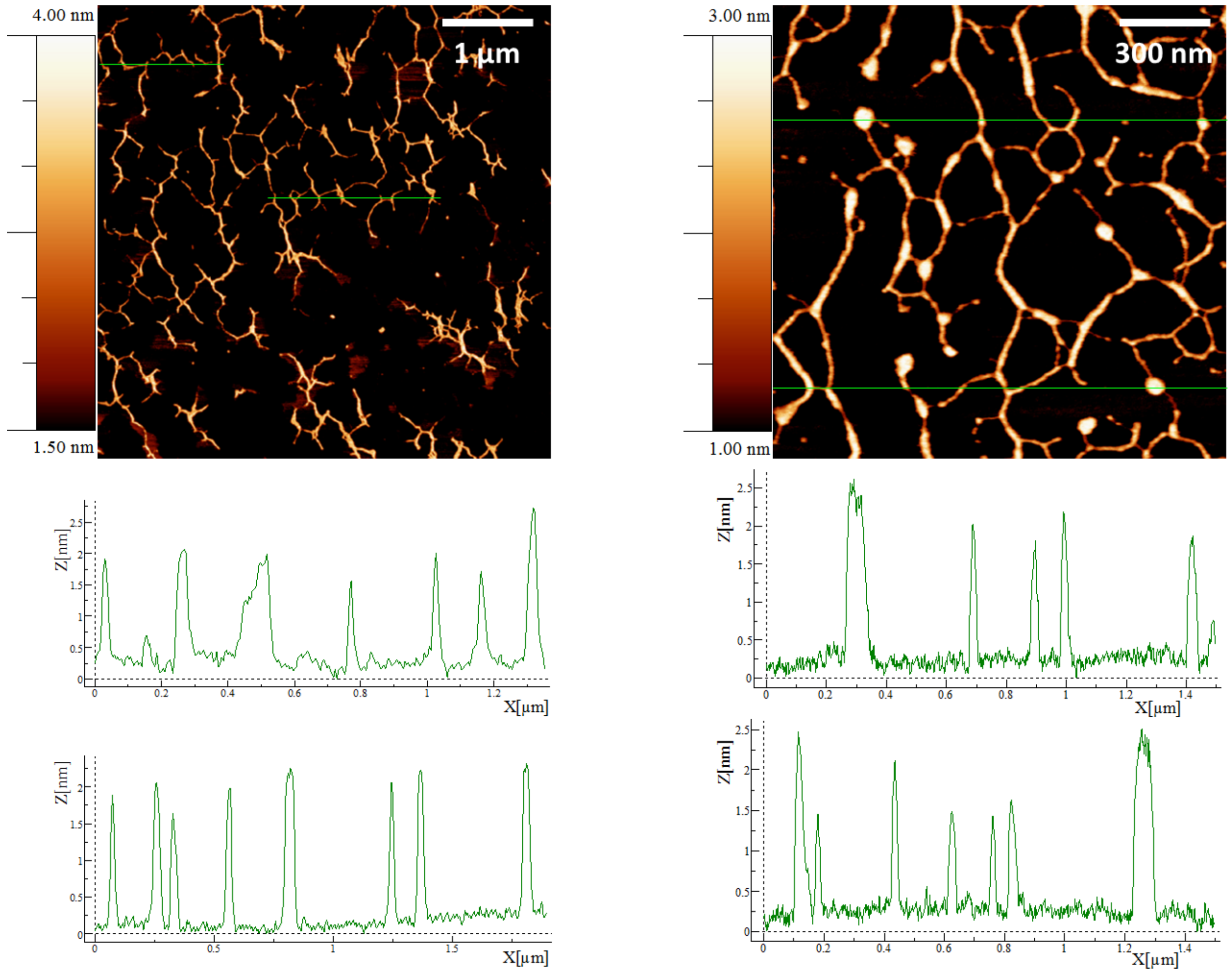

Figure S8. Atomic force microscopy images of DNA without pre-incubation. Linear DNA (BcuI-treated pCX eGFP at 10 ng/µl) in the buffer solution without incubation at 37°C was deposited onto freshly cleaved mica by spin coating.

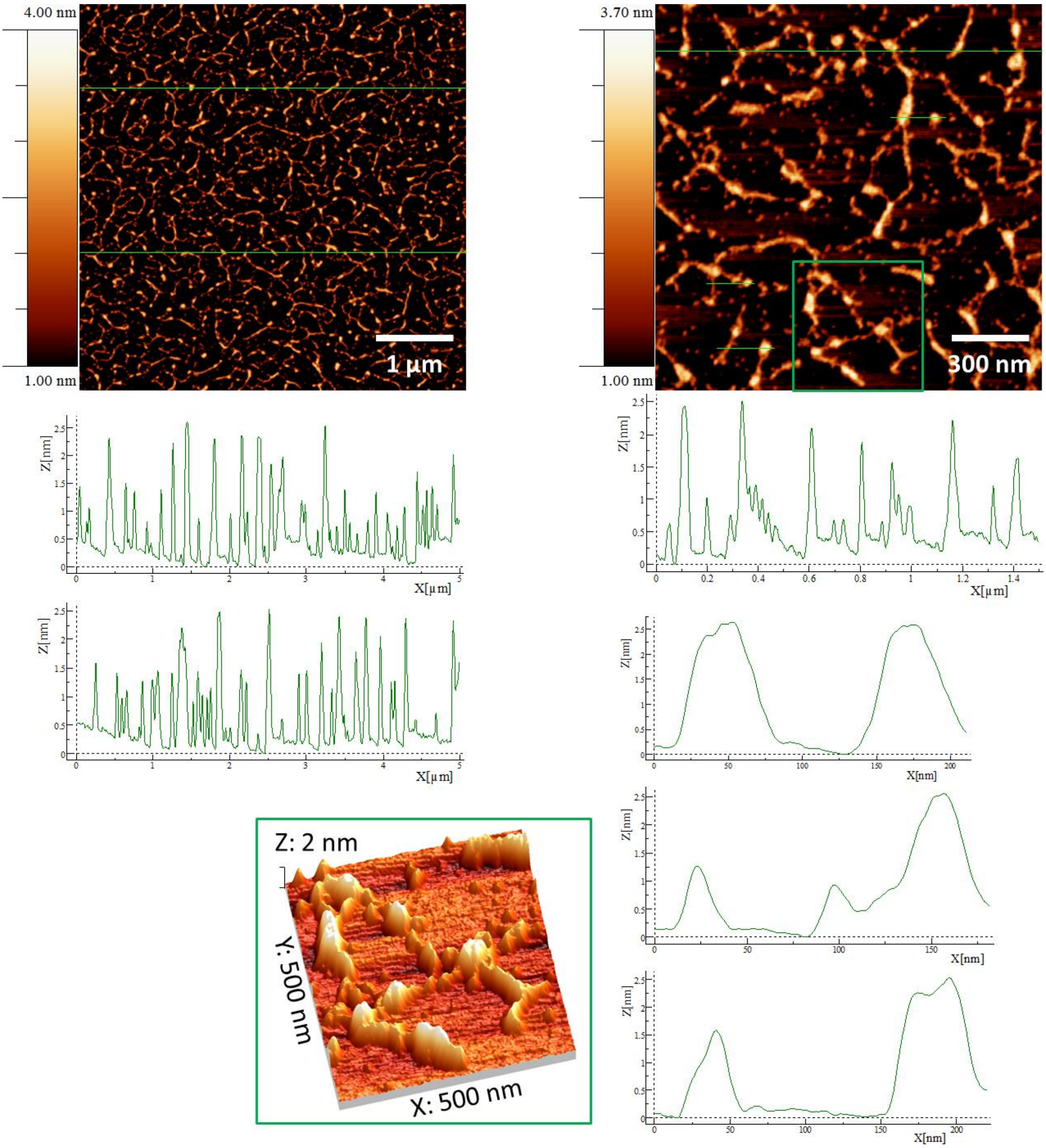

Figure S9. Atomic force microscopy images of DNA and wild-type HU protein without a C-terminal His tag without pre-incubation. Linear DNA (BcuI-treated pCX eGFP at 10 ng/µl) and HU protein (3 µM) in the buffer solution without incubation at 37°C was deposited onto freshly cleaved mica by spin coating. This protein: DNA ratio equals to the presence of 1 HU homodimer for every 10 bp DNA, if all proteins bind.

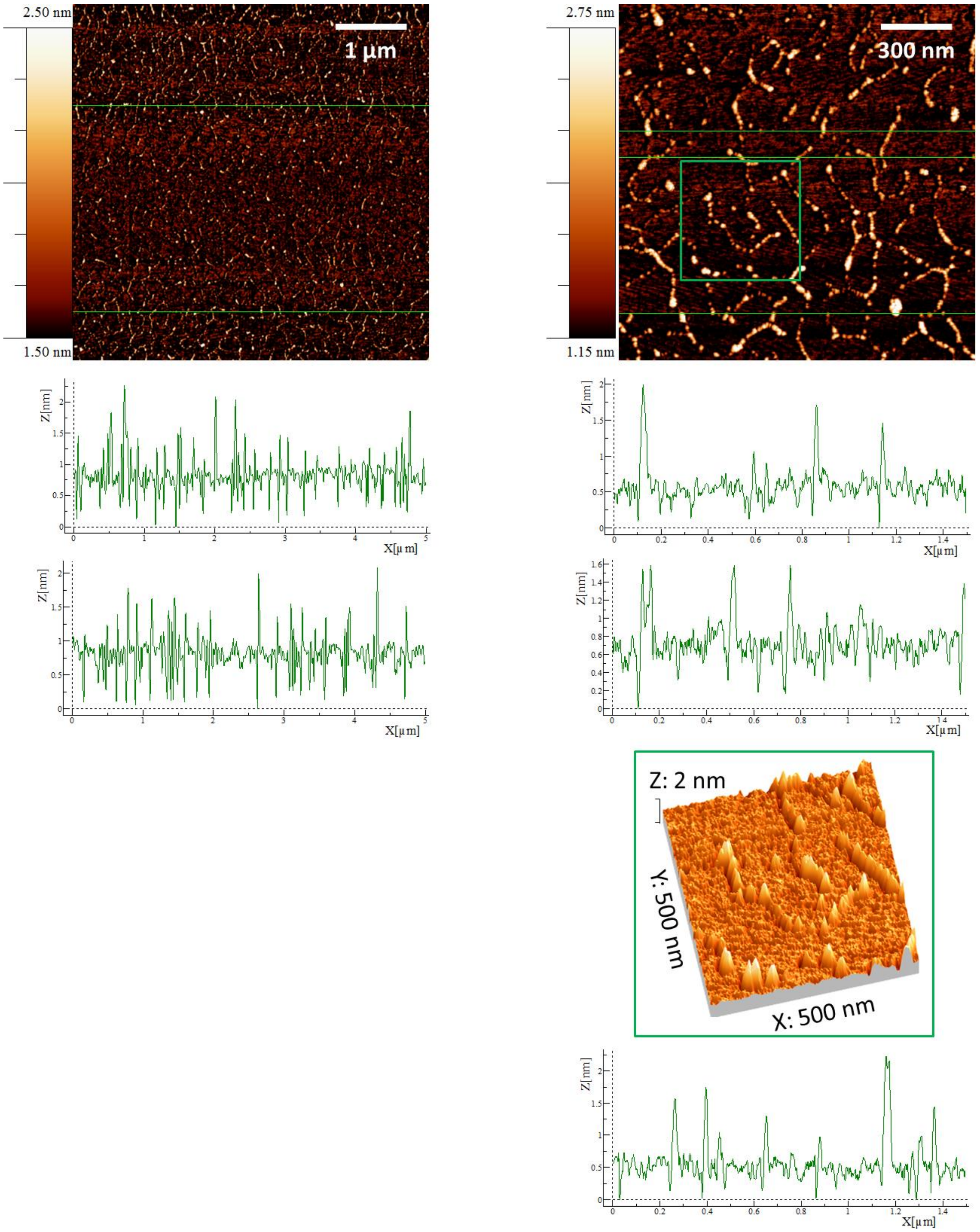

Figure S10. Atomic force microscopy images of DNA and wild-type HU protein with a C-terminal His tag without pre-incubation. Linear DNA (BcuI-treated pCX eGFP at 10 ng/µl) and HU protein (3 µM) in the buffer solution without incubation at 37°C was deposited onto freshly cleaved mica by spin coating. This protein: DNA ratio equals to the presence of 1 HU homodimer for every 10 bp DNA, if all proteins bind.

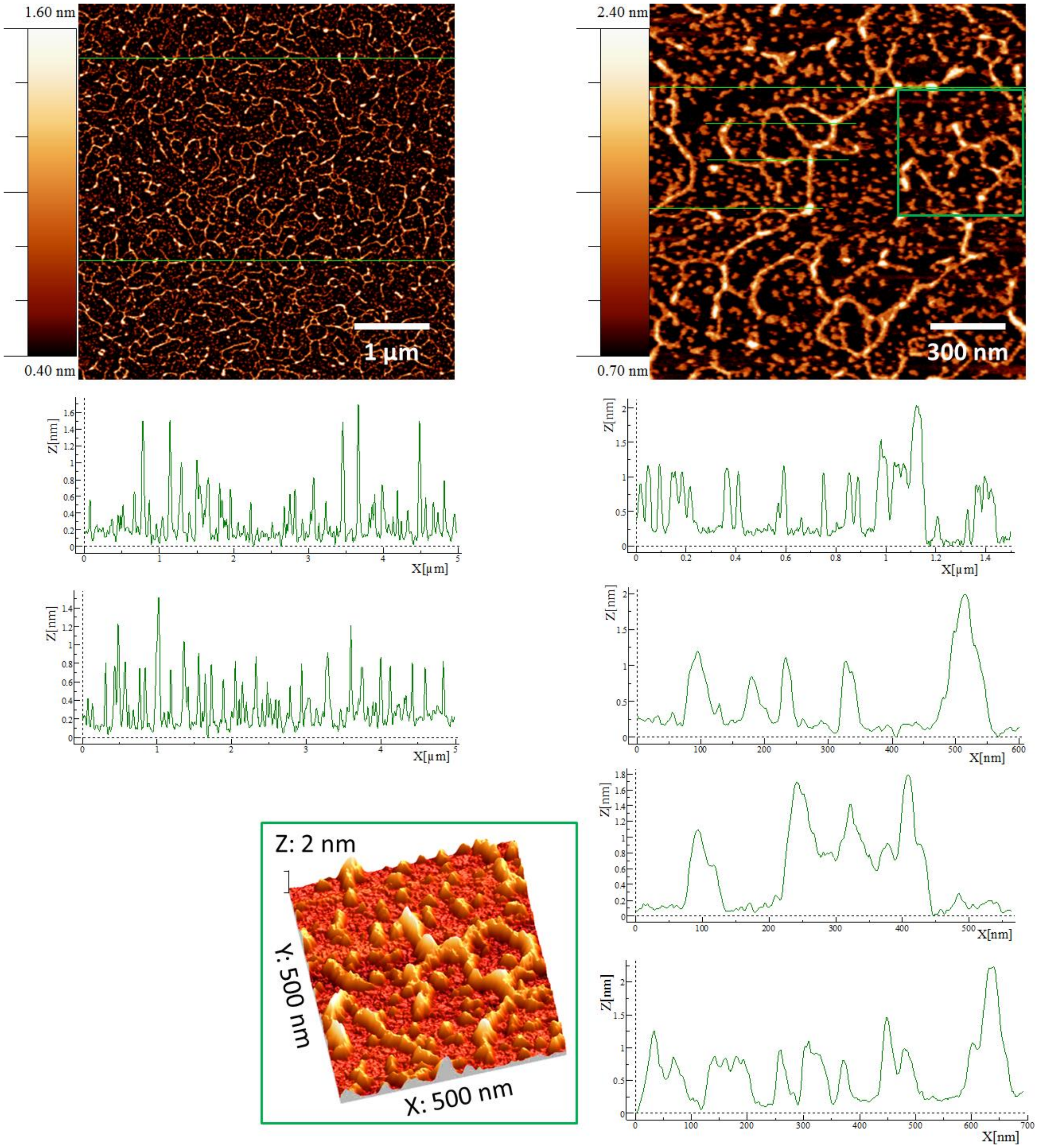

Figure S11. Atomic force microscopy images of DNA and K13ac HU protein with a C-terminal His tag without pre-incubation. Linear DNA (BcuI-treated pCX eGFP at 10 ng/µl) and HU protein (3 µM) in the buffer solution without incubation at 37°C was deposited onto freshly cleaved mica by spin coating. This protein: DNA ratio equals to the presence of 1 HU homodimer for every 10 bp DNA, if all proteins bind.

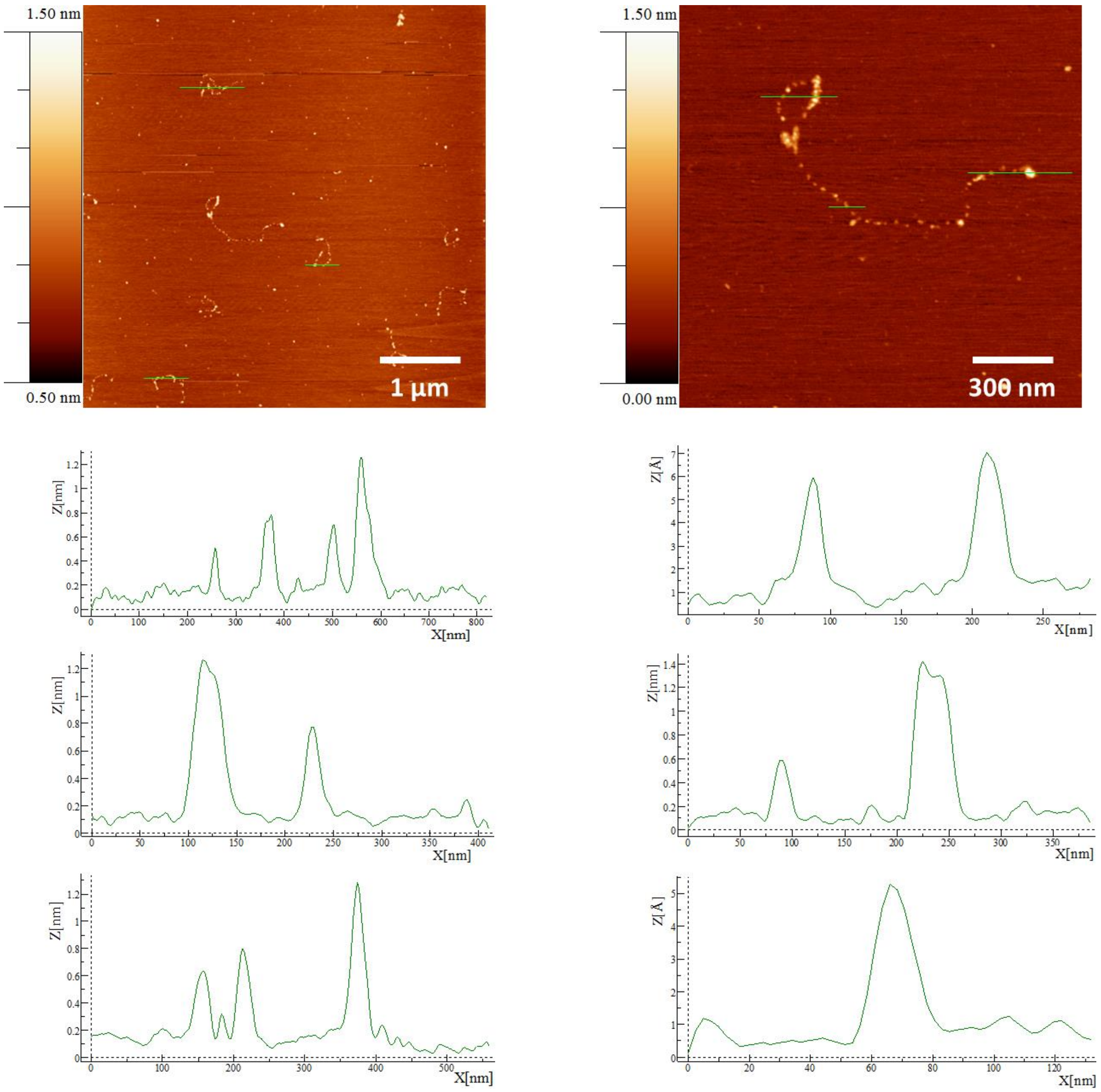

Figure S12. Atomic force microscopy images of DNA after incubation. Linear DNA (BcuI-treated pCX eGFP at 10 ng/µl) in the buffer solution was incubated at 37°C for 1 h before being deposited onto freshly cleaved mica by spin coating.

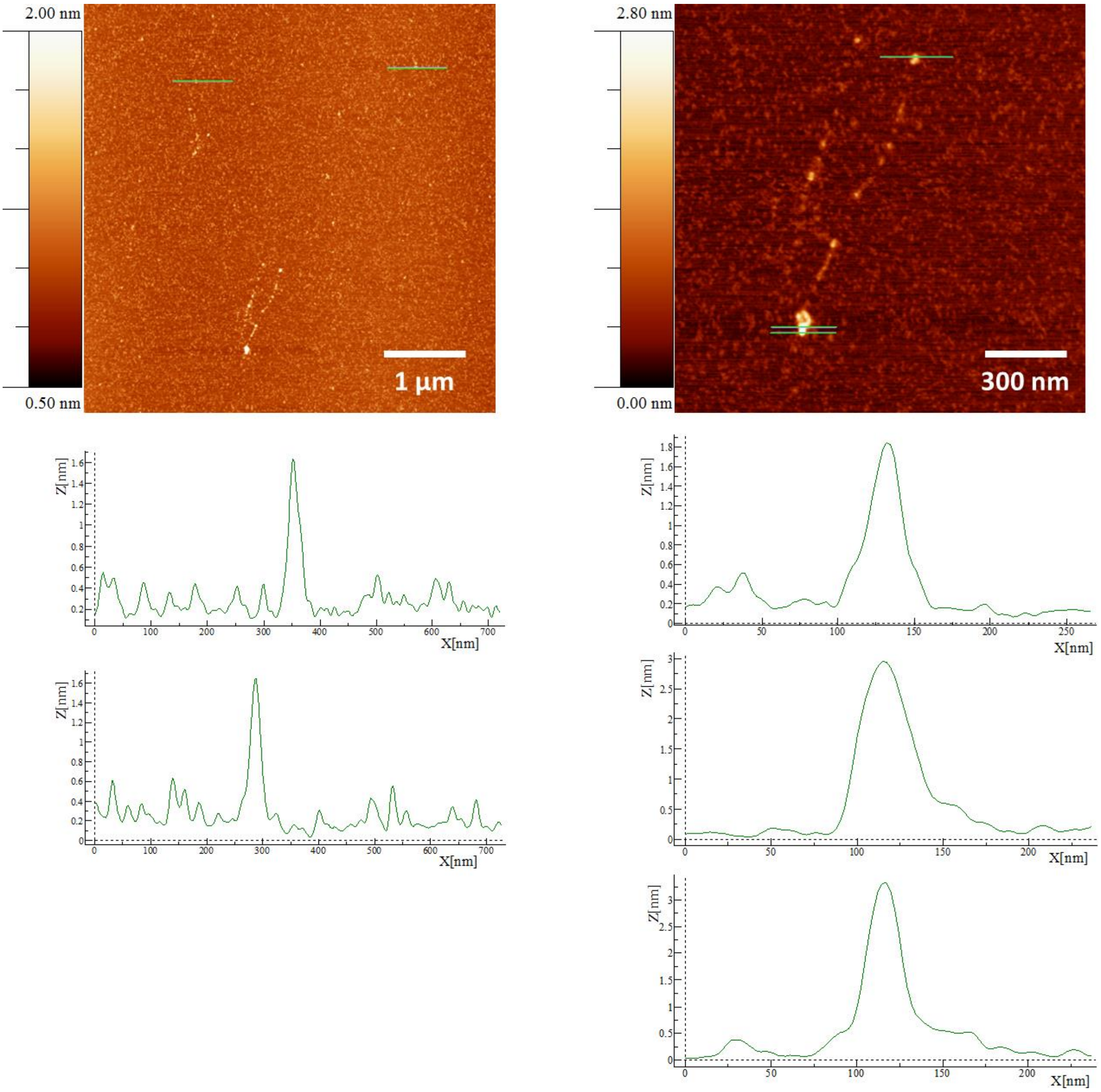

Figure S13. Atomic force microscopy images of DNA and wild-type HU protein without a C-terminal His tag after incubation. Linear DNA (BcuI-treated pCX eGFP at 10 ng/µl) and HU protein (3 µM) in the buffer solution were incubated at 37°C for 1 h before being deposited onto freshly cleaved mica by spin coating. This protein: DNA ratio equals to the presence of 1 HU homodimer for every 10 bp DNA, if all proteins bind.

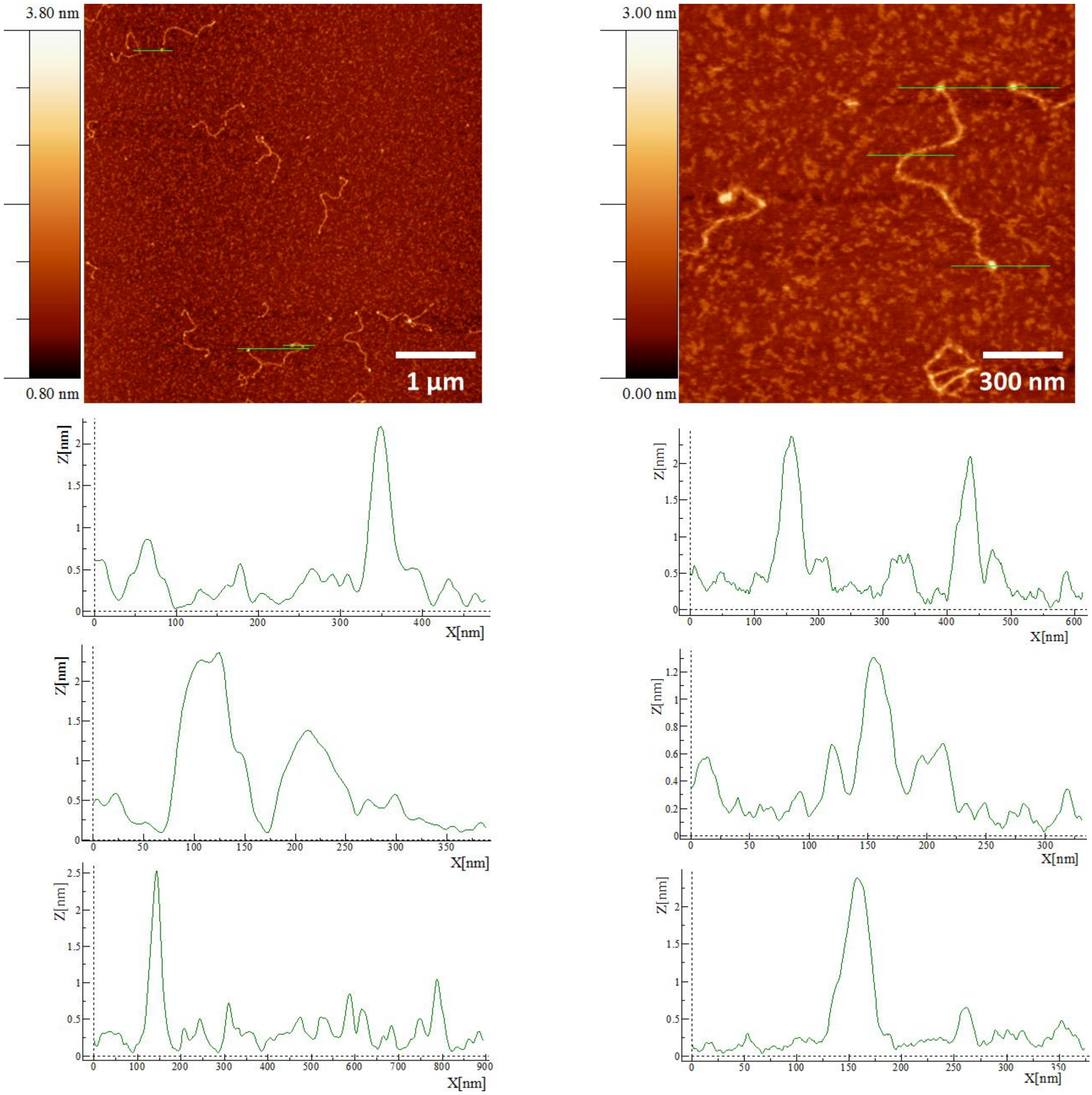

Figure S14. Atomic force microscopy images of DNA and wild-type HU protein with a C-terminal His tag after incubation. Linear DNA (BcuI-treated pCX eGFP at 10 ng/µl) and HU protein (3 µM) in the buffer solution were incubated at 37°C for 1 h before being deposited onto freshly cleaved mica by spin coating. This protein: DNA ratio equals to the presence of 1 HU homodimer for every 10 bp DNA, if all proteins bind.

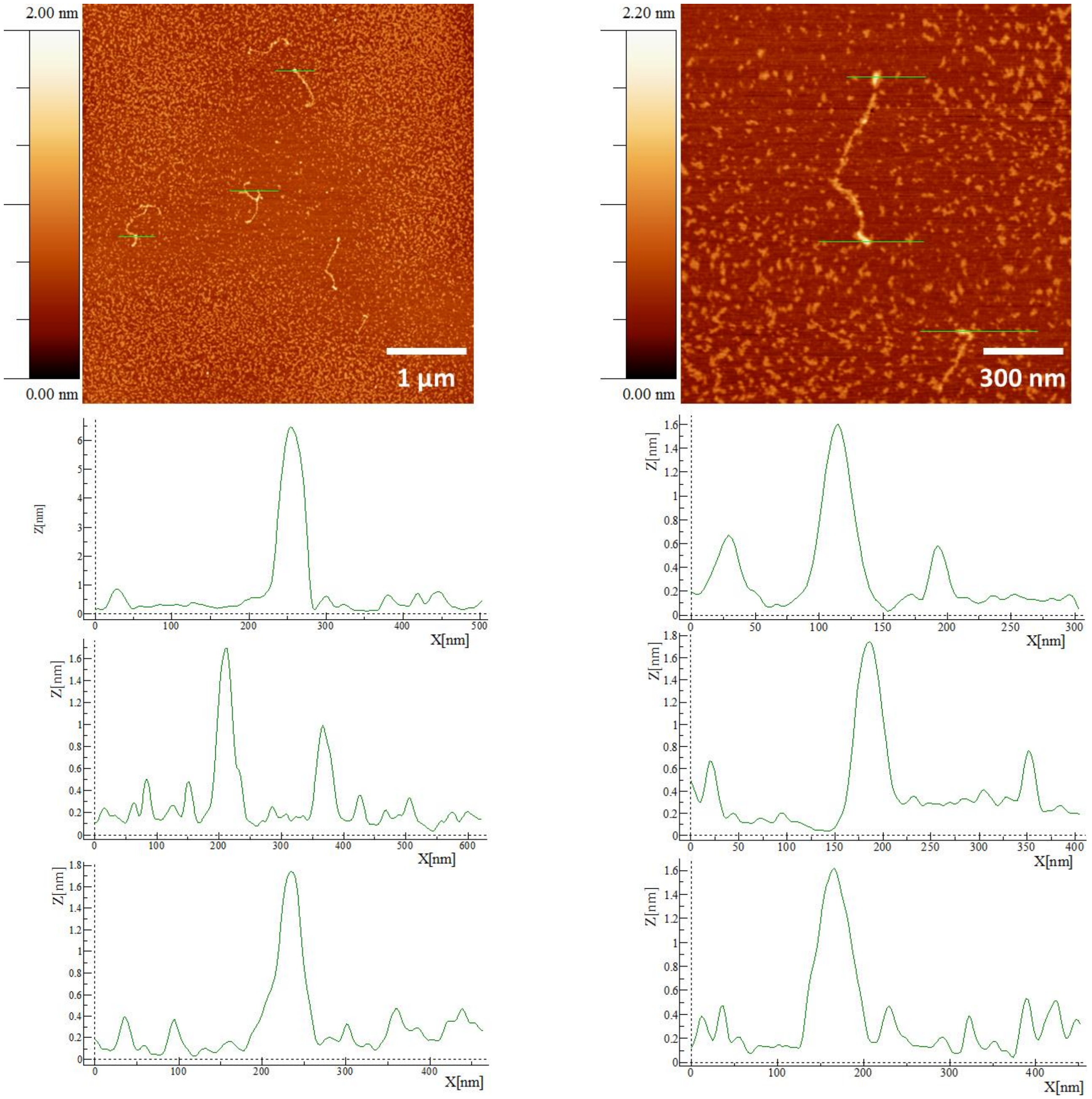

Figure S15. Atomic force microscopy images of DNA and K13ac HU protein with a C-terminal His tag after incubation. Linear DNA (BcuI-treated pCX eGFP at 10 ng/µl) and HU protein (3 µM) in the buffer solution were incubated at 37°C for 1 h before being deposited onto freshly cleaved mica by spin coating. This protein: DNA ratio equals to the presence of 1 HU homodimer for every 10 bp DNA, if all proteins bind.

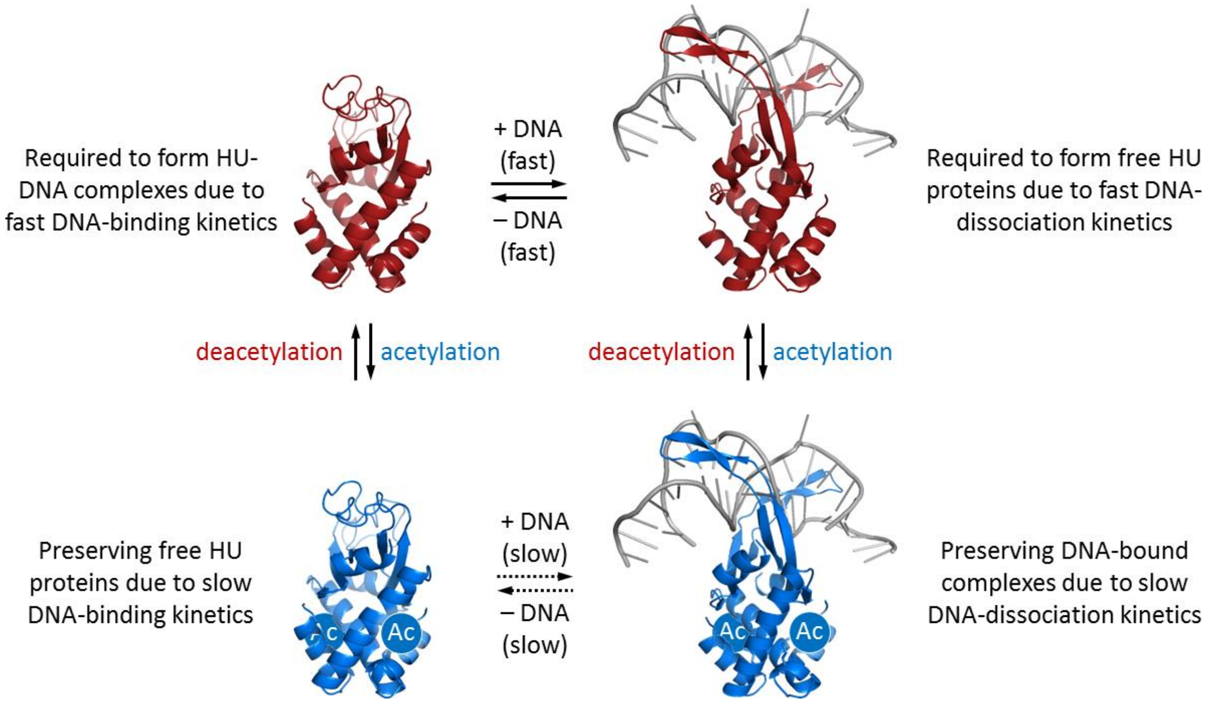

Figure S16. Model of Lys13 acetylation in regulating DNA binding of HU proteins. The fast DNA-association kinetics of the unmodified HU protein indicates that this species is required to form HU-DNA complexes. If DNA needs to dissociate from the complexes, HU proteins in the complexes have also in the non-acetylated state because acetylation significantly decreases the DNA dissociation kinetics. On the other hand, acetylation of free HU proteins will preserve the pool of free HU protein due to the slow DNA-binding kinetics of acetylated HU. Similarly, due to the slow DNA-dissociation kinetics, acetylated HU-DNA complexes would remain in the DNA-bound state and preserve HU-DNA complexes.

References

1. Kentache, T., Jouenne, T., De, E. and Hardouin, J. (2016) Proteomic characterization of N-alpha- and N-epsilon-acetylation in Acinetobacter baumannii. *J. Proteomics*, **144**, 148-158.
2. Kosono, S., Tamura, M., Suzuki, S., Kawamura, Y., Yoshida, A., Nishiyama, M. and Yoshida, M. (2015) Changes in the Acetylome and Succinylome of Bacillus subtilis in Response to Carbon Source. *Plos One*, **10**, e0131169.
3. Zhang, K., Zheng, S.Z., Yang, J.S., Chen, Y. and Cheng, Z.Y. (2013) Comprehensive Profiling of Protein Lysine Acetylation in Escherichia coli. *J. Proteome Res.*, **12**, 844-851.
4. Castano-Cerezo, S., Bernal, V., Post, H., Fuhrer, T., Cappadona, S., Sanchez-Diaz, N.C., Sauer, U., Heck, A.J.R., Altelaar, A.M.F. and Canovas, M. (2014) Protein acetylation affects acetate metabolism, motility and acid stress response in Escherichia coli. *Mol. Syst. Biol.*, **10**, 762.
5. Lee, D.W., Kim, D., Lee, Y.J., Kim, J.A., Choi, J.Y., Kang, S. and Pan, J.G. (2013) Proteomic analysis of acetylation in thermophilic Geobacillus kaustophilus. *Proteomics*, **13**, 2278-2282.
6. Ghosh, S., Padmanabhan, B., Anand, C. and Nagaraja, V. (2016) Lysine acetylation of the Mycobacterium tuberculosis HU protein modulates its DNA binding and genome organization. *Mol. Microbiol.*, **100**, 577-588.
7. Xie, L., Wang, X., Zeng, J., Zhou, M., Duan, X., Li, Q., Zhang, Z., Luo, H., Pang, L., Li, W. *et al.* (2015) Proteome-wide lysine acetylation profiling of the human pathogen Mycobacterium tuberculosis. *Int. J. Biochem. Cell Biol.*, **59**, 193-202.
8. Ouidir, T., Cosette, P., Jouenne, T. and Hardouin, J. (2015) Proteomic profiling of lysine acetylation in Pseudomonas aeruginosa reveals the diversity of acetylated proteins. *Proteomics*, **15**, 2152-2157.
9. Okanishi, H., Kim, K., Masui, R. and Kuramitsu, S. (2013) Acetylome with structural mapping reveals the significance of lysine acetylation in Thermus thermophilus. *J. Proteome Res.*, **12**, 3952-3968.
10. Pan, J.Y., Ye, Z.C., Cheng, Z.Y., Peng, X.J., Wen, L.Y. and Zhao, F.K. (2014) Systematic Analysis of the Lysine Acetylome in Vibrio parahemolyticus. *J. Proteome Res.*, **13**, 3294-3302.

peg.1002 29134

#1	b+	b2+	b3+	Seq.	y+	y2+	y3+	#2
1	114.09135	57.54931	38.70197	L				24
2	201.12338	101.06533	67.71264	S	2777.39276	1389.20002	926.46910	23
3	300.19180	150.59954	100.73545	V	2690.36073	1345.68400	897.45843	22
4	463.25512	232.13120	155.08989	Y	2591.29231	1296.14979	864.43562	21
5	550.28715	275.64721	184.10057	S	2428.22899	1214.61813	810.08118	20
6	679.32975	340.16851	227.11477	E	2341.19696	1171.10212	781.07050	19
7	842.39307	421.70017	281.46921	Y	2212.15436	1106.58082	738.05630	18
8	989.42848	495.21788	330.48101	M-Oxidation	2049.09104	1025.04916	683.70186	17
9	1117.48706	559.24717	373.16720	Q	1902.05563	951.53145	634.69006	16
10	1218.53474	609.77101	406.84976	T	1773.99705	887.50216	592.00387	15
11	1331.61881	666.31304	444.54445	L	1672.94937	836.97832	558.32131	14
12	1444.70288	722.85508	482.23914	L	1559.86530	780.43629	520.62662	13
13	1572.76146	786.88437	524.92534	Q	1446.78123	723.89425	482.93193	12
14	1659.79349	830.40038	553.93601	S	1318.72265	659.86496	440.24573	11
15	1787.85207	894.42967	596.62221	Q	1231.69062	616.34895	411.23506	10
16	1924.91098	962.95913	642.30851	H	1103.63204	552.31966	368.54886	9
17	2011.94301	1006.47514	671.31919	S	966.57313	483.79020	322.86256	8
18	2125.02708	1063.01718	709.01388	L	879.54110	440.27419	293.85188	7
19	2262.08599	1131.54663	754.70018	H	766.45703	383.73215	256.15719	6
20	2390.18096	1195.59412	797.39850	K	629.39812	315.20270	210.47089	5
21	2560.28650	1280.64689	854.10035	K-Acetyl	501.30315	251.15521	167.77257	4
22	2688.34508	1344.67618	896.78654	Q	331.19761	166.10244	111.07072	3
23	2801.42915	1401.21821	934.48123	I	203.13903	102.07315	68.38453	2
24				A	90.05496	45.53112	30.68984	1

Extracted from: Z:\712AB\SK17-S (Ac)\Chichi-Abaumannii-SK17-S-Acetyl_DrWu_20140703.raw #29134 RT: 80.85
ITMS, CID@35.00, z=+3, Mono m/z=964.16290 Da, MH+=2890.47416 Da, Match Tol.=0.6 Da

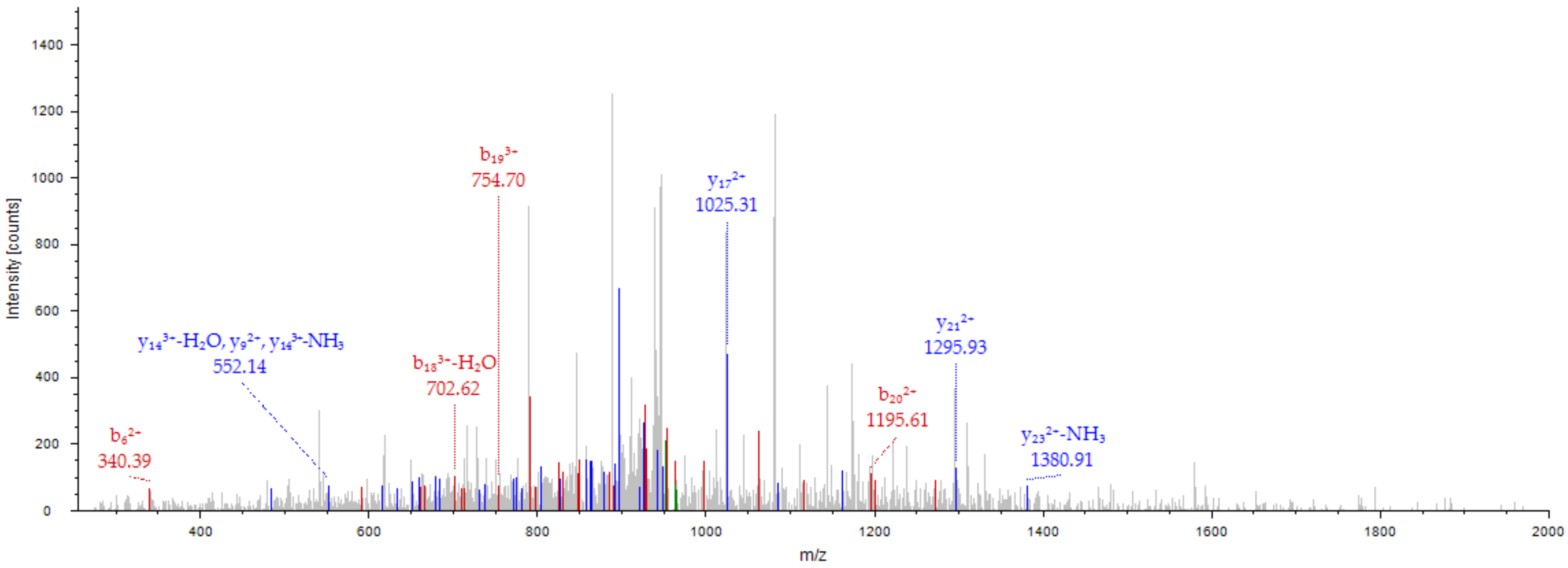

peg.1003   19195

#1	b⁺	b²⁺	b³⁺	Seq.	y⁺	y²⁺	y³⁺	#2
1	129.10225	65.05476	43.70560	K				30
2	292.16557	146.58642	98.06004	Y	3178.55802	1589.78265	1060.19086	29
3	455.22889	228.11808	152.41448	Y	3015.49470	1508.25099	1005.83642	28
4	556.27657	278.64192	186.09704	T	2852.43138	1426.71933	951.48198	27
5	657.32425	329.16576	219.77960	T	2751.38370	1376.19549	917.79942	26
6	714.34572	357.67650	238.78676	G	2650.33602	1325.67165	884.11686	25
7	801.37775	401.19251	267.79743	S	2593.31455	1297.16091	865.10970	24
8	914.46182	457.73455	305.49212	L	2506.28252	1253.64490	836.09902	23
9	1077.52514	539.26621	359.84656	Y	2393.19845	1197.10286	798.40433	22
10	1148.56226	574.78477	383.52560	A	2230.13513	1115.57120	744.04989	21
11	1263.58921	632.29824	421.86792	D	2159.09801	1080.05264	720.37085	20
12	1449.66853	725.33790	483.89436	W	2044.07106	1022.53917	682.02854	19
13	1548.73695	774.87211	516.91717	V	1857.99174	929.49951	620.00210	18
14	1663.76390	832.38559	555.25948	D	1758.92332	879.96530	586.97929	17
15	1762.83232	881.91980	588.28229	V	1643.89637	822.45182	548.63697	16
16	1819.85379	910.43053	607.28945	G	1544.82795	772.91761	515.61417	15
17	1918.92221	959.96474	640.31225	V	1487.80648	744.40688	496.60701	14
18	2019.96989	1010.48858	673.99481	T	1388.73806	694.87267	463.58420	13
19	2134.99684	1068.00206	712.33713	D	1287.69038	644.34883	429.90164	12
20	2248.08091	1124.54409	750.03182	L	1172.66343	586.83535	391.55933	11
21	2418.18644	1209.59686	806.73366	K-Acetyl	1059.57936	530.29332	353.86464	10
22	2475.20791	1238.10759	825.74082	G	889.47382	445.24055	297.16279	9
23	2604.25051	1302.62889	868.75502	E	832.45235	416.72981	278.15563	8
24	2691.28254	1346.14491	897.76570	S	703.40975	352.20851	235.14143	7
25	2748.30401	1374.65564	916.77285	G	616.37772	308.69250	206.13076	6
26	2835.33604	1418.17166	945.78353	S	559.35625	280.18176	187.12360	5
27	2934.40446	1467.70587	978.80634	V	472.32422	236.66575	158.11292	4
28	3033.47288	1517.24008	1011.82914	V	373.25580	187.13154	125.09012	3
29	3132.54130	1566.77429	1044.85195	V	274.18738	137.59733	92.06731	2
30				R	175.11896	88.06312	59.04450	1

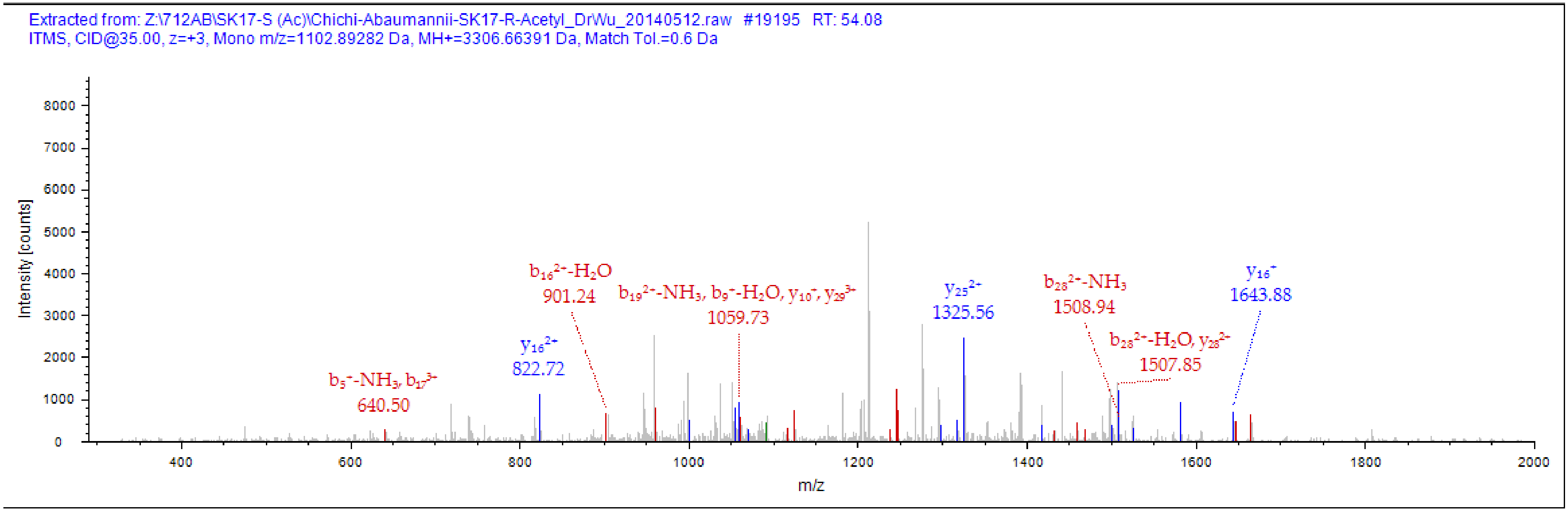

peg.1007 21648

#1	b⁺	b²⁺	b³⁺	Seq.	y⁺	y²⁺	y³⁺	#2
1	130.04987	65.52857	44.02147	S-Acetyl				25
2	300.15541	150.58134	100.72332	K-Acetyl	2661.27519	1331.14123	887.76325	24
3	415.18236	208.09482	139.06564	D	2491.16966	1246.08847	831.06140	23
4	502.21439	251.61083	168.07631	S	2376.14271	1188.57499	792.71909	22
5	589.24642	295.12685	197.08699	S	2289.11068	1145.05898	763.70841	21
6	745.34754	373.17741	249.12070	R	2202.07865	1101.54296	734.69773	20
7	892.41596	446.71162	298.14350	F	2045.97753	1023.49240	682.66403	19
8	979.44799	490.22763	327.15418	S	1898.90911	949.95819	633.64122	18
9	1092.53206	546.76967	364.84887	I	1811.87708	906.44218	604.63054	17
10	1206.57499	603.79113	402.86318	N	1698.79301	849.90014	566.93585	16
11	1303.62776	652.31752	435.21410	P	1584.75008	792.87868	528.92154	15
12	1402.69618	701.85173	468.23691	V	1487.69731	744.35229	496.57062	14
13	1516.73911	758.87319	506.25122	N	1388.62889	694.81808	463.54781	13
14	1587.77623	794.39175	529.93026	A	1274.58596	637.79662	425.53350	12
15	1702.80318	851.90523	568.27258	D	1203.54884	602.27806	401.85446	11
16	1817.83013	909.41870	606.61489	D	1088.52189	544.76458	363.51215	10
17	1904.86216	952.93472	635.62557	S	973.49494	487.25111	325.16983	9
18	2003.93058	1002.46893	668.64838	V	886.46291	443.73509	296.15915	8
19	2100.98335	1050.99531	700.99930	P	787.39449	394.20088	263.13635	7
20	2229.04193	1115.02460	743.68549	Q	690.34172	345.67450	230.78542	6
21	2286.06340	1143.53534	762.69265	G	562.28314	281.64521	188.09923	5
22	2387.11108	1194.05918	796.37521	T	505.26167	253.13447	169.09207	4
23	2501.15401	1251.08064	834.38952	N	404.21399	202.61063	135.40951	3
24	2602.20169	1301.60448	868.07208	T	290.17106	145.58917	97.39520	2
25				K-Acetyl	189.12338	95.06533	63.71264	1

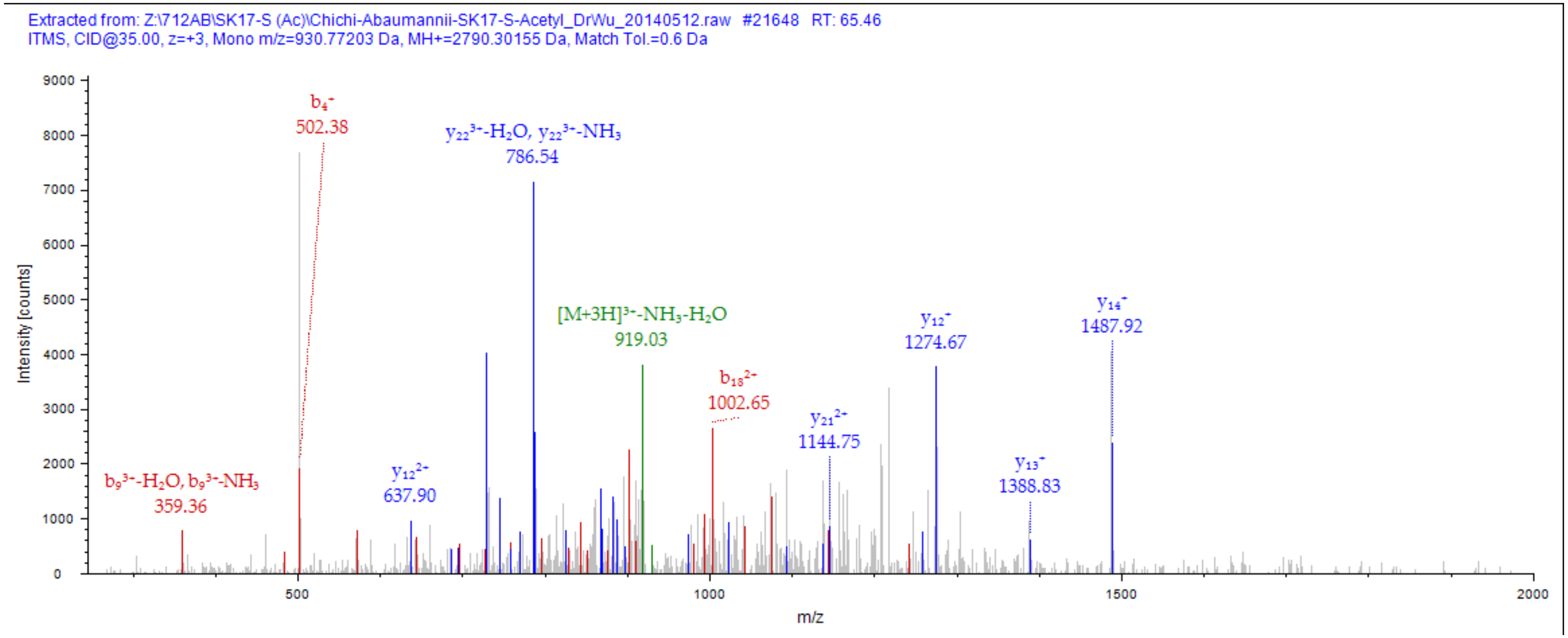

peg.1026 22581

#1	b⁺	b²⁺	b³⁺	Seq.	y⁺	y²⁺	y³⁺	#2
1	174.05834	87.53281	58.69096	M-Acetyl				18
2	261.09037	131.04882	87.70164	S	1902.90402	951.95565	634.97286	17
3	431.19591	216.10159	144.40349	K-Acetyl	1815.87199	908.43963	605.96218	16
4	532.24359	266.62543	178.08605	T	1645.76645	823.38686	549.26033	15
5	633.29127	317.14927	211.76861	T	1544.71877	772.86302	515.57777	14
6	761.34985	381.17856	254.45480	Q	1443.67109	722.33918	481.89521	13
7	890.39245	445.69986	297.46900	E	1315.61251	658.30989	439.20902	12
8	1004.43538	502.72133	335.48331	N	1186.56991	593.78859	396.19482	11
9	1101.48815	551.24771	367.83423	P	1072.52698	536.76713	358.18051	10
10	1172.52527	586.76627	391.51327	A	975.47421	488.24074	325.82959	9
11	1271.59369	636.30048	424.53608	V	904.43709	452.72218	302.15055	8
12	1400.63629	700.82178	467.55028	E	805.36867	403.18797	269.12774	7
13	1514.67922	757.84325	505.56459	N	676.32607	338.66667	226.11354	6
14	1601.71125	801.35926	534.57527	S	562.28314	281.64521	188.09923	5
15	1672.74837	836.87782	558.25431	A	475.25111	238.12919	159.08855	4
16	1800.80695	900.90711	600.94050	Q	404.21399	202.61063	135.40951	3
17	1929.84955	965.42841	643.95470	E	276.15541	138.58134	92.72332	2
18				K	147.11281	74.06004	49.70912	1

Extracted from: Z:\712AB\SK17-S (Ac)\Chichi-Abaumannii-SK17-R-Acetyl_DrWu_20140512.raw #22581 RT: 64.25
ITMS, CID@35.00, z=+3, Mono m/z=692.65155 Da, MH+=2075.94010 Da, Match Tol.=0.6 Da

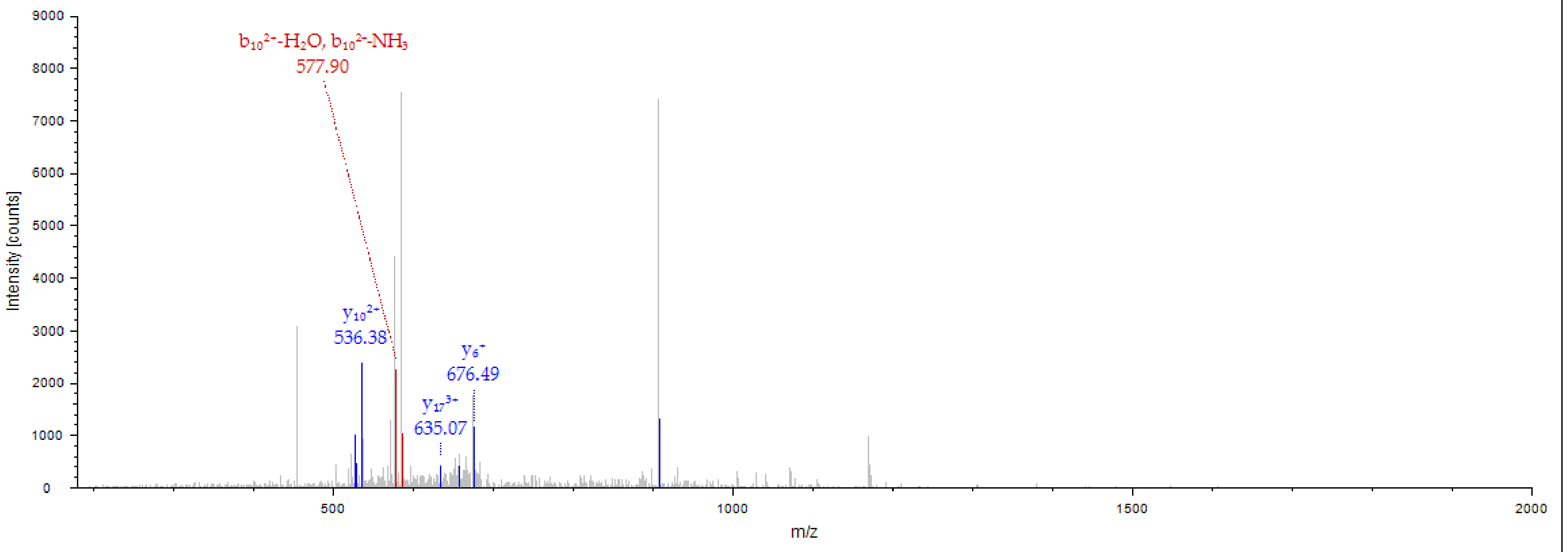

peg.1027 27375

peg.271 27375

#1	b^{+}	b^{2+}	b^{3+}	Seq.	y^{+}	y^{2+}	y^{3+}	#2
1	114.09135	57.54931	38.70197	I				38
2	213.15977	107.08352	71.72477	V	3690.03006	1845.51867	1230.68154	37
3	316.16896	158.58812	106.06117	C	3590.96164	1795.98446	1197.65873	36
4	415.23738	208.12233	139.08398	V	3487.95245	1744.47986	1163.32233	35
5	502.26941	251.63834	168.09465	S	3388.88403	1694.94565	1130.29953	34
6	589.30144	295.15436	197.10533	S	3301.85200	1651.42964	1101.28885	33
7	702.38551	351.69639	234.80002	I	3214.81997	1607.91362	1072.27817	32
8	789.41754	395.21241	263.81070	S	3101.73590	1551.37159	1034.58348	31
9	846.43901	423.72314	282.81785	G	3014.70387	1507.85557	1005.57281	30
10	959.52308	480.26518	320.51254	I	2957.68240	1479.34484	986.56565	29
11	1030.56020	515.78374	344.19158	A	2844.59833	1422.80280	948.87096	28
12	1087.58167	544.29447	363.19874	G	2773.56121	1387.28424	925.19192	27
13	1201.62460	601.31594	401.21305	N	2716.53974	1358.77351	906.18476	26
14	1314.70867	657.85797	438.90774	L	2602.49681	1301.75204	868.17045	25
15	1371.73014	686.36871	457.91490	G	2489.41274	1245.21001	830.47576	24
16	1499.78872	750.39800	500.60109	Q	2432.39127	1216.69927	811.46861	23
17	1600.83640	800.92184	534.28365	T	2304.33269	1152.66998	768.78241	22
18	1714.87933	857.94330	572.29796	N	2203.28501	1102.14614	735.09985	21
19	1877.94265	939.47496	626.65240	Y	2089.24208	1045.12468	697.08554	20
20	1948.97977	974.99352	650.33144	A	1926.17876	963.59302	642.73110	19
21	2020.01689	1010.51208	674.01048	A	1855.14164	928.07446	619.05206	18
22	2107.04892	1054.02810	703.02116	S	1784.10452	892.55590	595.37302	17
23	2277.15445	1139.08086	759.72300	K-Acetyl	1697.07249	849.03988	566.36235	16
24	2348.19157	1174.59942	783.40204	A	1526.96695	763.98711	509.66050	15
25	2405.21304	1203.11016	802.40920	G	1455.92983	728.46855	485.98146	14
26	2504.28146	1252.64437	835.43200	V	1398.90836	699.95782	466.97430	13
27	2617.36553	1309.18640	873.12669	I	1299.83994	650.42361	433.95150	12
28	2674.38700	1337.69714	892.13385	G	1186.75587	593.88157	396.25681	11
29	2787.47107	1394.23917	929.82854	L	1129.73440	565.37084	377.24965	10
30	2886.53949	1443.77338	962.85135	V	1016.65033	508.82880	339.55496	9
31	3014.63446	1507.82087	1005.54967	K	917.58191	459.29459	306.53215	8
32	3161.70288	1581.35508	1054.57248	F	789.48694	395.24711	263.83383	7
33	3262.75056	1631.87892	1088.25504	T	642.41852	321.71290	214.81102	6
34	3333.78768	1667.39748	1111.93408	A	541.37084	271.18906	181.12846	5
35	3430.84045	1715.92386	1144.28500	P	470.33372	235.67050	157.44942	4
36	3543.92452	1772.46590	1181.97969	I	373.28095	187.14411	125.09850	3
37	3657.00859	1829.00793	1219.67438	L	260.19688	130.60208	87.40381	2
38				K	147.11281	74.06004	49.70912	1

Extracted from: Z:\712AB\SK17-S (Ac)\Chichi-Abaumannii-SK17-R-Acetyl_DrWu_20140703.raw #27375 RT: 75.01
ITMS, CID@35.00, z=+3, Mono m/z=1268.37878 Da, MH+=3803.12180 Da, Match Tol.=0.6 Da

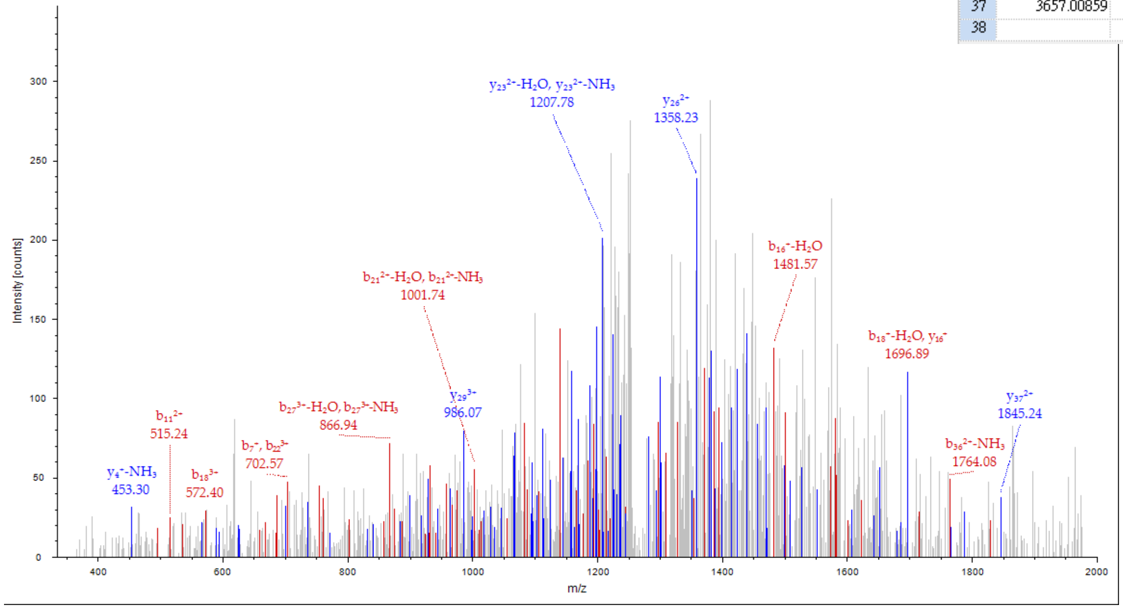

peg.1037 15752

#1	b^+	b^{2+}	Seq.	y^+	y^{2+}	#2
1	180.07675	90.54201	H-Acetyl			18
2	311.11725	156.06226	M	1984.96203	992.98465	17
3	410.18567	205.59647	V	1853.92153	927.46440	16
4	557.25409	279.13068	F	1754.85311	877.93019	15
5	644.28612	322.64670	S	1607.78469	804.39598	14
6	741.33889	371.17308	P	1520.75266	760.87997	13
7	855.38182	428.19455	N	1423.69989	712.35358	12
8	912.40329	456.70528	G	1309.65696	655.33212	11
9	1082.50883	541.75805	K-Acetyl	1252.63549	626.82138	10
10	1210.56741	605.78734	Q	1082.52995	541.76861	9
11	1281.60453	641.30590	A	954.47137	477.73932	8
12	1444.66785	722.83756	Y	883.43425	442.22076	7
13	1557.75192	779.37960	I	720.37093	360.68910	6
14	1658.79960	829.90344	T	607.28686	304.14707	5
15	1729.83672	865.42200	A	506.23918	253.62323	4
16	1858.87932	929.94330	E	435.20206	218.10467	3
17	1989.91982	995.46355	M	306.15946	153.58337	2
18			R	175.11896	88.06312	1

Extracted from: Z:\712AB\SK17-S (Ac)\Chichi-Abaumannii-SK17-R-Acetyl_DrWu_20140703.raw #15752 RT: 45.74
ITMS, CID@35.00, z=+2, Mono m/z=1082.52368 Da, MH+=2164.04009 Da, Match Tol.=0.6 Da

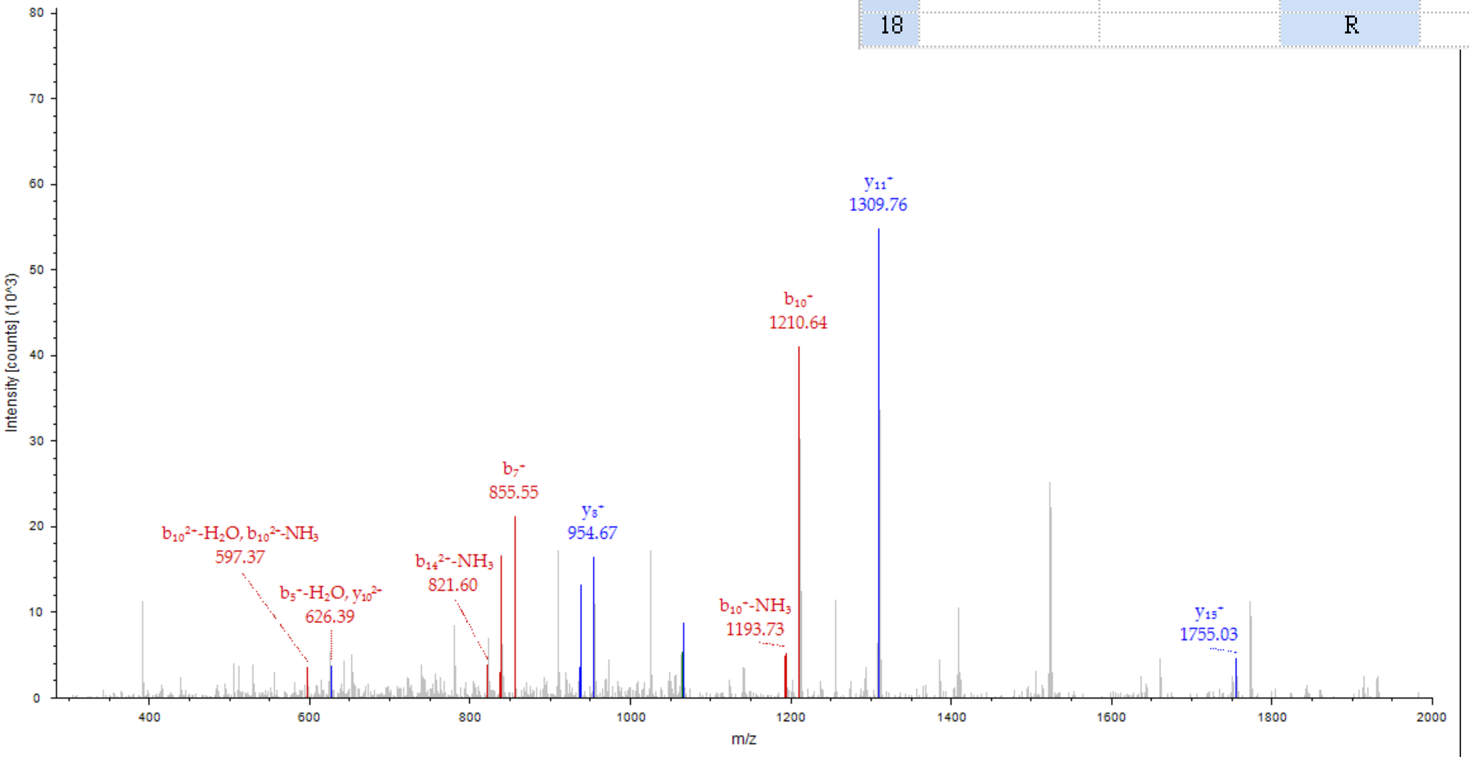

peg.1037 17517

#1	b+	b2+	Seq.	y+	y2+	#2
1	180.07675	90.54201	H-Acetyl			18
2	311.11725	156.06226	M	1984.96203	992.98465	17
3	410.18567	205.59647	V	1853.92153	927.46440	16
4	557.25409	279.13068	F	1754.85311	877.93019	15
5	644.28612	322.64670	S	1607.78469	804.39598	14
6	741.33889	371.17308	P	1520.75266	760.87997	13
7	855.38182	428.19455	N	1423.69989	712.35358	12
8	912.40329	456.70528	G	1309.65696	655.33212	11
9	1082.50883	541.75805	K-Acetyl	1252.63549	626.82138	10
10	1210.56741	605.78734	Q	1082.52995	541.76861	9
11	1281.60453	641.30590	A	954.47137	477.73932	8
12	1444.66785	722.83756	Y	883.43425	442.22076	7
13	1557.75192	779.37960	I	720.37093	360.68910	6
14	1658.79960	829.90344	T	607.28686	304.14707	5
15	1729.83672	865.42200	A	506.23918	253.62323	4
16	1858.87932	929.94330	E	435.20206	218.10467	3
17	1989.91982	995.46355	M	306.15946	153.58337	2
18			R	175.11896	88.06312	1

Extracted from: Z:\712AB\SK17-S (Ac)\Chichi-Abaumannii-SK17-S-Acetyl_DrWu_20140703.raw #17517 RT: 49.21
ITMS, CID@35.00, z=+2, Mono m/z=1082.52307 Da, MH+=2164.03887 Da, Match Tol.=0.6 Da

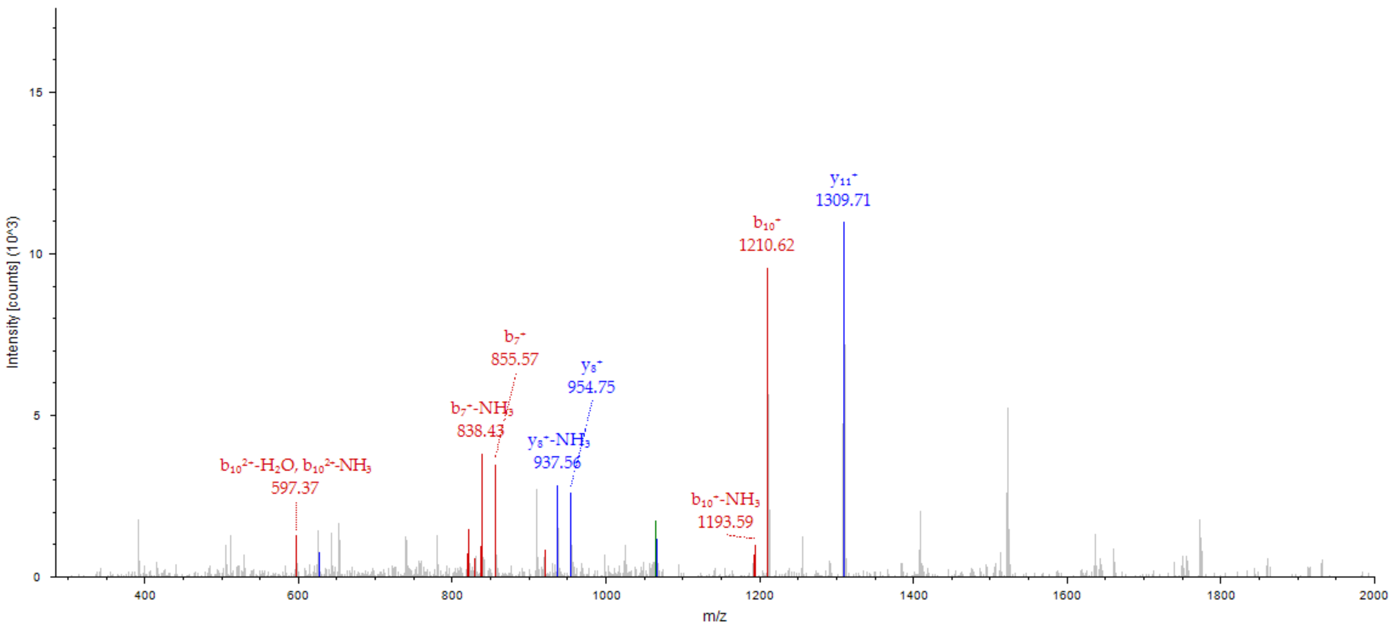

peg.1038 26132

#1	b⁺	b²⁺	Seq.	y⁺	y²⁺	#2
1	171.11281	86.06004	K-Acetyl			10
2	327.21393	164.11060	R	1066.56402	533.78565	9
3	384.23540	192.62134	G	910.46290	455.73509	8
4	547.29872	274.15300	Y	853.44143	427.22435	7
5	662.32567	331.66647	D	690.37811	345.69269	6
6	775.40974	388.20851	L	575.35116	288.17922	5
7	862.44177	431.72452	S	462.26709	231.63718	4
8	949.47380	475.24054	S	375.23506	188.12117	3
9	1062.55787	531.78257	L	288.20303	144.60515	2
10			R	175.11896	88.06312	1

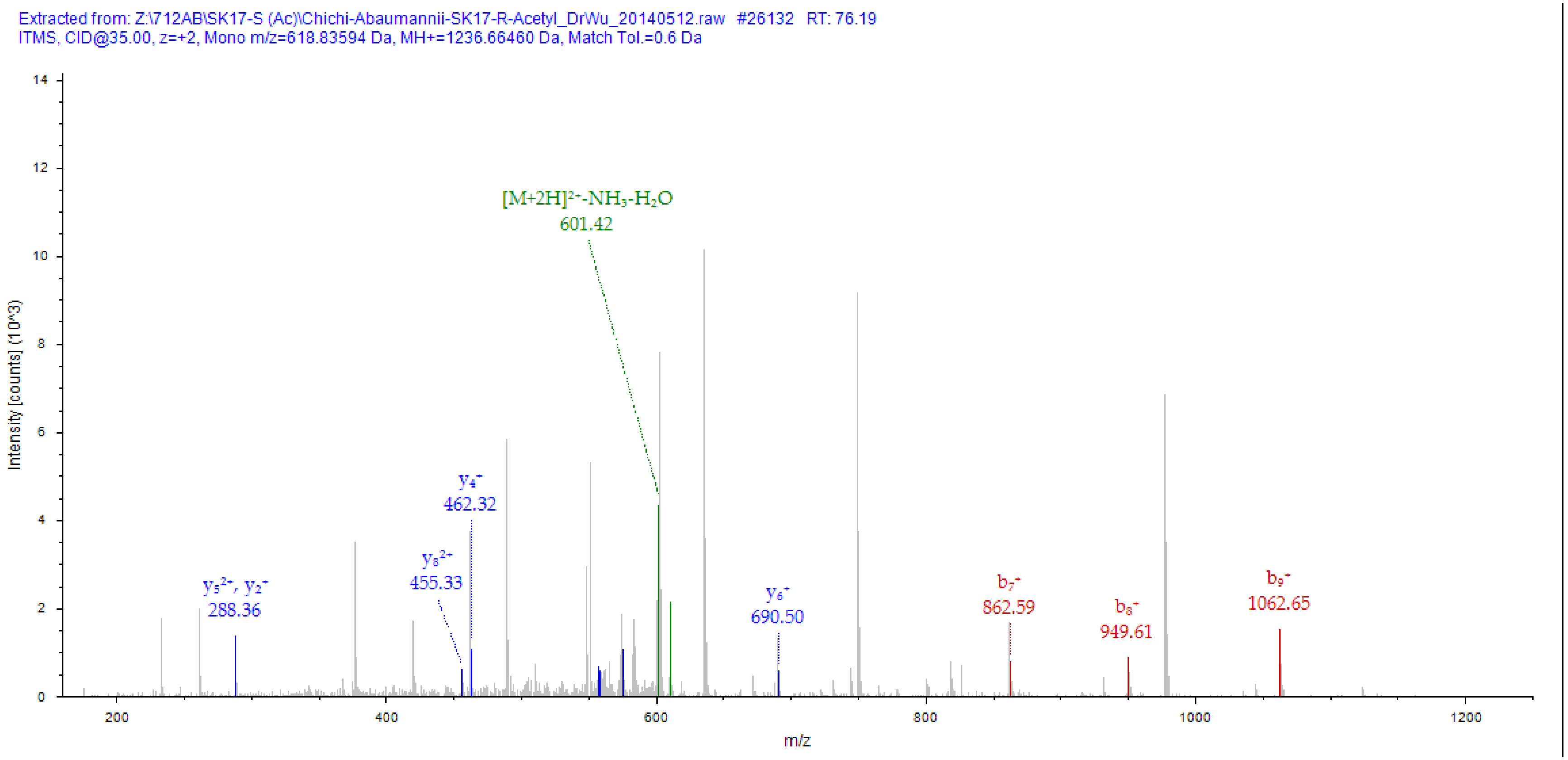

peg.1063 3522

#1	b+	b2+	Seq.	y+	y2+	#2
1	114.05496	57.53112	A-Acetyl			11
2	243.09756	122.05242	E	1117.55969	559.28348	10
3	358.12451	179.56589	D	988.51709	494.76218	9
4	486.21948	243.61338	K	873.49014	437.24871	8
5	614.27806	307.64267	Q	745.39517	373.20122	7
6	671.29953	336.15340	G	617.33659	309.17193	6
7	841.40507	421.20617	K-Acetyl	560.31512	280.66120	5
8	898.42654	449.71691	G	390.20958	195.60843	4
9	969.46366	485.23547	A	333.18811	167.09769	3
10	1056.49569	528.75148	S	262.15099	131.57913	2
11			R	175.11896	88.06312	1

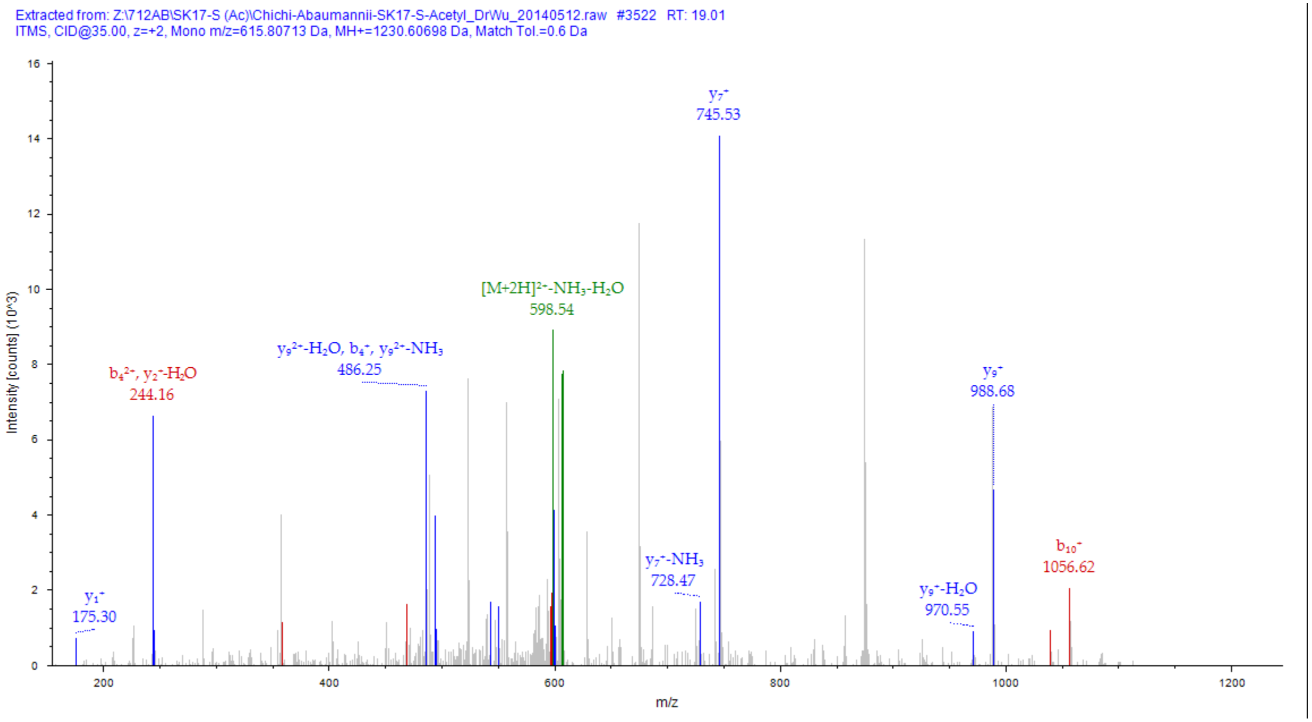

peg.113   22768

#1	b^+	b^{2+}	Seq.	y^+	y^{2+}	#2
1	148.04269	74.52498	M-Oxidation			15
2	277.08529	139.04628	E	1705.85134	853.42931	14
3	390.16936	195.58832	L	1576.80874	788.90801	13
4	518.22794	259.61761	Q	1463.72467	732.36597	12
5	575.24941	288.12834	G	1335.66609	668.33668	11
6	688.33348	344.67038	I	1278.64462	639.82595	10
7	848.36414	424.68571	C-Carbam…	1165.56055	583.28391	9
8	985.42305	493.21516	H	1005.52989	503.26858	8
9	1155.52858	578.26793	K-Acetyl	868.47098	434.73913	7
10	1286.56908	643.78818	M	698.36545	349.68636	6
11	1423.62799	712.31763	H	567.32495	284.16611	5
12	1494.66511	747.83619	A	430.26604	215.63666	4
13	1551.68658	776.34693	G	359.22892	180.11810	3
14	1664.77065	832.88896	L	302.20745	151.60736	2
15			K-Acetyl	189.12338	95.06533	1

Extracted from: Z:\712AB\SK17-S (Ac)\Chichi-Abaumannii-SK17-S-Acetyl_DrWu_20140512.raw #22768 RT: 68.72
ITMS, CID@35.00, z=+2, Mono m/z=926.94098 Da, MH+=1852.87468 Da, Match Tol.=0.6 Da

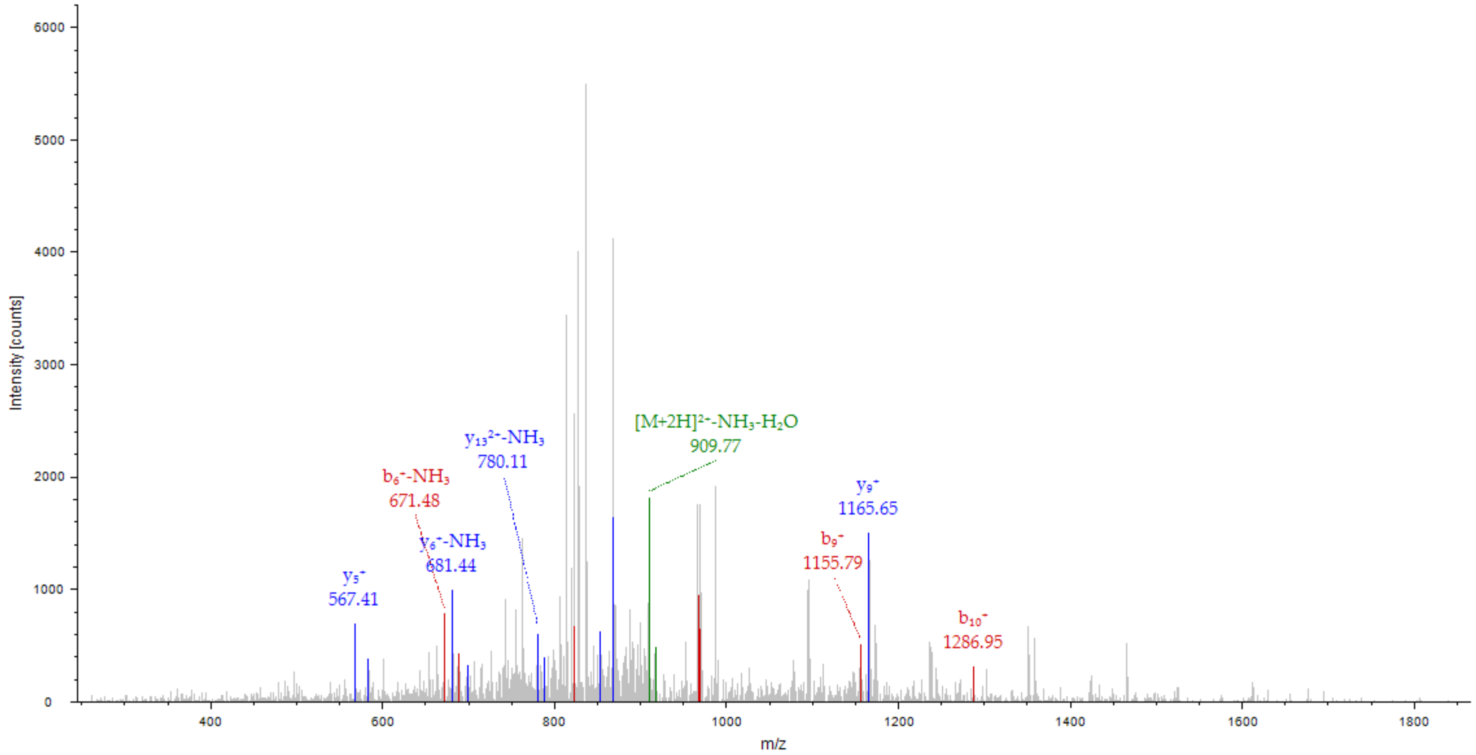

peg.1132 16322

#1	b⁺	b²⁺	b³⁺	Seq.	y⁺	y²⁺	y³⁺	#2
1	190.08626	95.54677	64.03360	F-Acetyl				23
2	305.11321	153.06024	102.37592	D	2649.24031	1325.12379	883.75162	22
3	442.17212	221.58970	148.06222	H	2534.21336	1267.61032	845.40930	21
4	513.20924	257.10826	171.74126	A	2397.15445	1199.08086	799.72300	20
5	650.26815	325.63771	217.42757	H	2326.11733	1163.56230	776.04396	19
6	820.37369	410.69048	274.12941	K-Acetyl	2189.05842	1095.03285	730.35766	18
7	935.40064	468.20396	312.47173	D	2018.95289	1009.98008	673.65581	17
8	1098.46396	549.73562	366.82617	Y	1903.92594	952.46661	635.31350	16
9	1245.53238	623.26983	415.84898	F	1740.86262	870.93495	580.95906	15
10	1332.56441	666.78584	444.85965	S	1593.79420	797.40074	531.93625	14
11	1502.66994	751.83861	501.56150	K-Acetyl	1506.76217	753.88472	502.92557	13
12	1630.72852	815.86790	544.24769	Q	1336.65663	668.83195	446.22373	12
13	1743.81259	872.40993	581.94238	I	1208.59805	604.80266	403.53753	11
14	1814.84971	907.92849	605.62142	A	1095.51398	548.26063	365.84284	10
15	1929.87666	965.44197	643.96374	D	1024.47686	512.74207	342.16380	9
16	2076.94508	1038.97618	692.98654	F	909.44991	455.22859	303.82149	8
17	2179.95427	1090.48077	727.32294	C	762.38149	381.69438	254.79868	7
18	2279.02269	1140.01498	760.34575	V	659.37230	330.18979	220.46228	6
19	2394.04964	1197.52846	798.68806	D	560.30388	280.65558	187.43948	5
20	2465.08676	1233.04702	822.36710	A	445.27693	223.14210	149.09716	4
21	2564.15518	1282.58123	855.38991	V	374.23981	187.62354	125.41812	3
22	2692.21376	1346.61052	898.07610	Q	275.17139	138.08933	92.39531	2
23				K	147.11281	74.06004	49.70912	1

Extracted from: Z:\712AB\SK17-S (Ac)\Chichi-Abaumannii-SK17-S-Acetyl_DrWu_20140512.raw #16322 RT: 51.02
ITMS, CID@35.00, z=+3, Mono m/z=946.77405 Da, MH+=2838.30759 Da, Match Tol.=0.6 Da

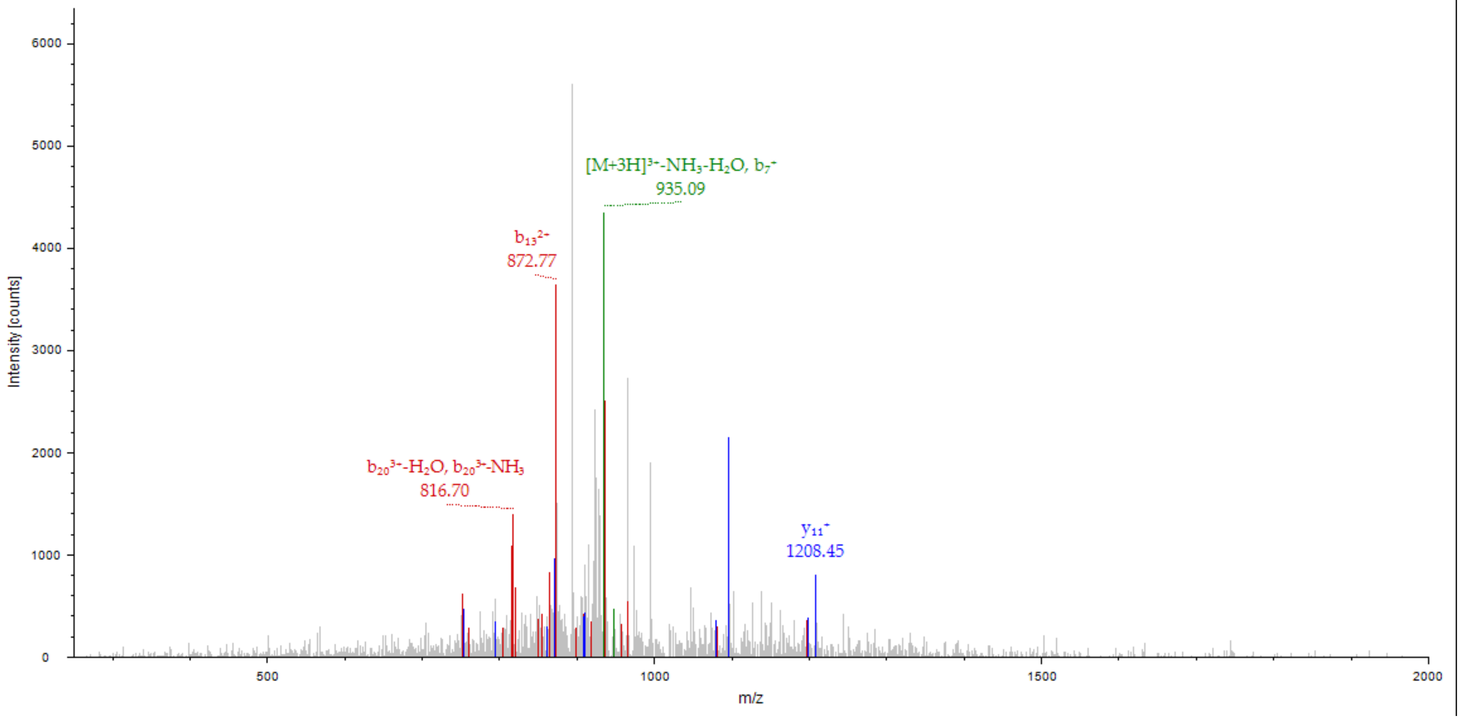

peg.1176 2951

#1	b+	b2+	Seq.	y+	y2+	#2
1	116.03423	58.52075	D			9
2	229.11830	115.06279	L	1029.55755	515.28241	8
3	357.17688	179.09208	Q	916.47348	458.74038	7
4	471.21981	236.11354	N	788.41490	394.71109	6
5	600.26241	300.63484	E	674.37197	337.68962	5
6	713.34648	357.17688	L	545.32937	273.16832	4
7	883.45201	442.22964	K-Acetyl	432.24530	216.62629	3
8	998.47896	499.74312	D	262.13976	131.57352	2
9			K	147.11281	74.06004	1

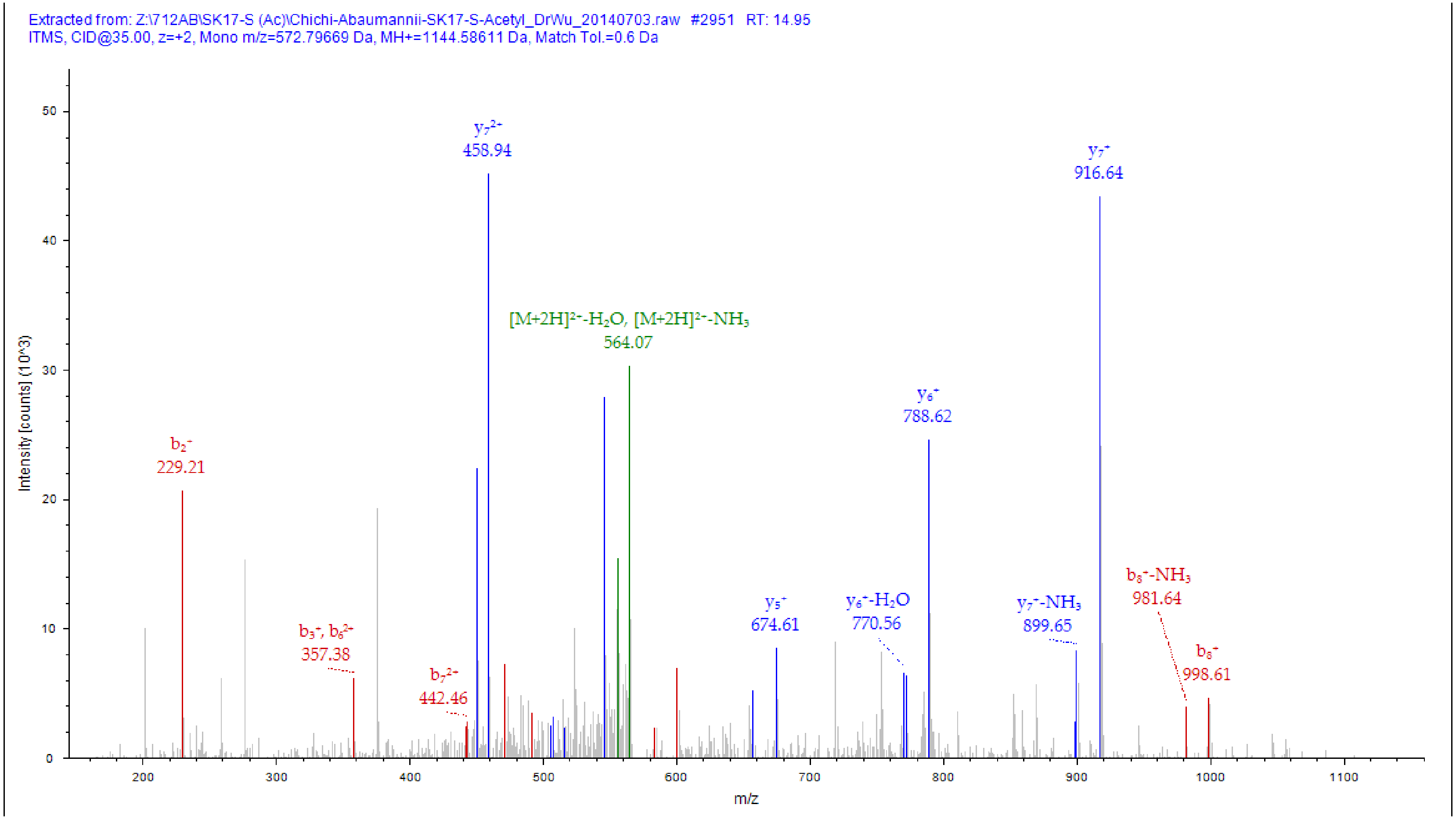

peg.1187   1744

#1	b⁺	b²⁺	b³⁺	Seq.	y⁺	y²⁺	y³⁺	#2
1	102.05496	51.53112	34.68984	T				10
2	217.08191	109.04459	73.03215	D	1090.55619	545.78173	364.19025	9
3	288.11903	144.56315	96.71119	A	975.52924	488.26826	325.84793	8
4	435.15444	218.08086	145.72300	M-Oxidation	904.49212	452.74970	302.16889	7
5	548.23851	274.62289	183.41769	L	757.45671	379.23199	253.15709	6
6	605.25998	303.13363	202.42484	G	644.37264	322.68996	215.46240	5
7	775.36552	388.18640	259.12669	K-Acetyl	587.35117	294.17922	196.45524	4
8	888.44959	444.72843	296.82138	I	417.24563	209.12645	139.75339	3
9	1017.49219	509.24973	339.83558	E	304.16156	152.58442	102.05870	2
10				R	175.11896	88.06312	59.04450	1

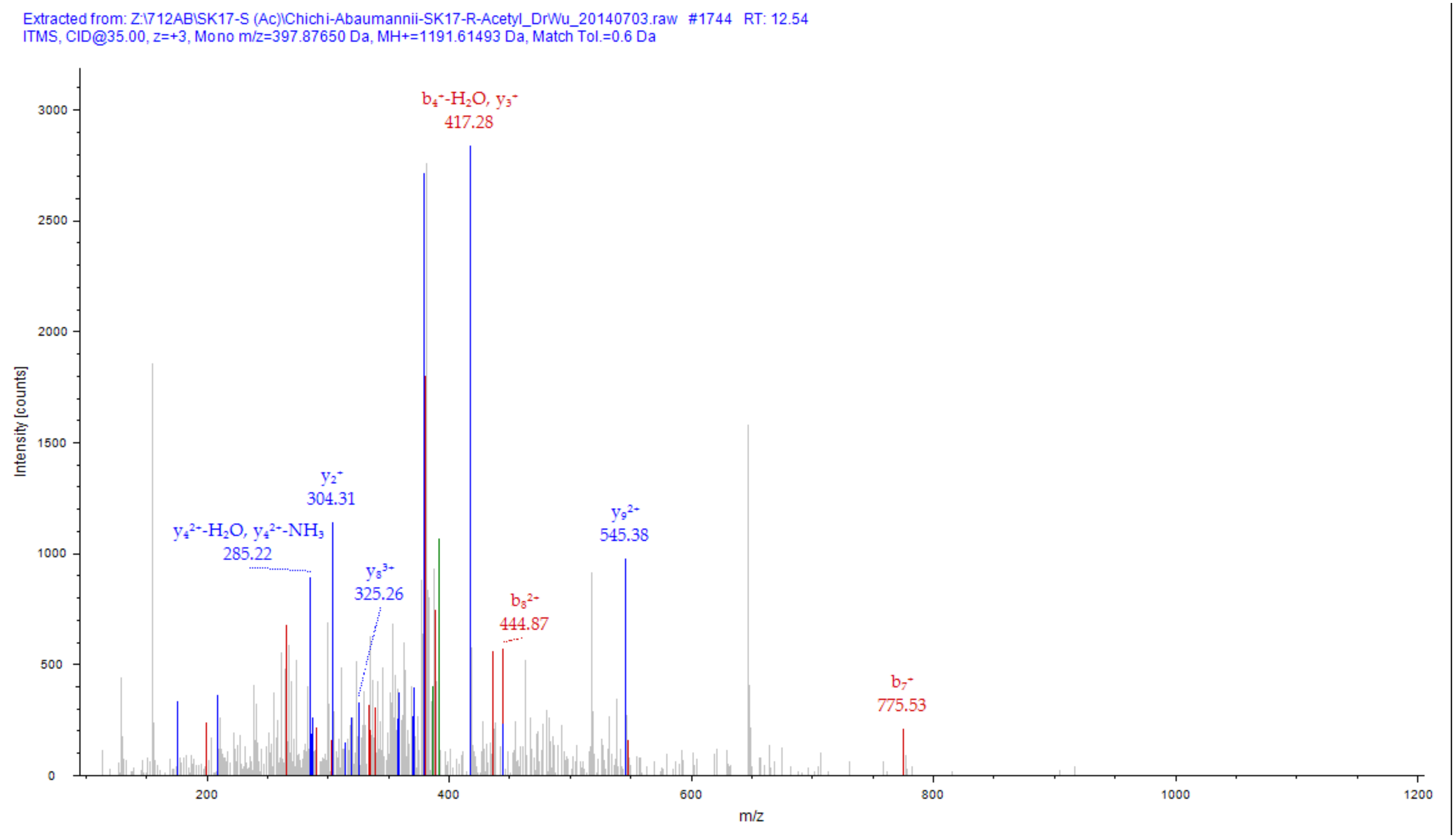

peg.1211   26137

#1	b⁺	b²⁺	b³⁺	Seq.	y⁺	y²⁺	y³⁺	#2
1	130.04987	65.52857	44.02147	S-Acetyl				22
2	245.07682	123.04205	82.36379	D	2639.32872	1320.16800	880.44776	21
3	332.10885	166.55806	111.37447	S	2524.30177	1262.65452	842.10544	20
4	495.17217	248.08972	165.72891	Y	2437.26974	1219.13851	813.09476	19
5	658.23549	329.62138	220.08335	Y	2274.20642	1137.60685	758.74032	18
6	759.28317	380.14522	253.76591	T	2111.14310	1056.07519	704.38588	17
7	830.32029	415.66378	277.44495	A	2010.09542	1005.55135	670.70332	16
8	943.40436	472.20582	315.13964	L	1939.05830	970.03279	647.02428	15
9	1071.46294	536.23511	357.82583	Q	1825.97423	913.49075	609.32959	14
10	1227.56406	614.28567	409.85954	R	1697.91565	849.46146	566.64340	13
11	1298.60118	649.80423	433.53858	A	1541.81453	771.41090	514.60969	12
12	1412.64411	706.82569	471.55289	N	1470.77741	735.89234	490.93065	11
13	1572.67477	786.84102	524.89644	C-Carbam···	1356.73448	678.87088	452.91634	10
14	1742.78030	871.89379	581.59828	K-Acetyl	1196.70382	598.85555	399.57279	9
15	1855.86437	928.43582	619.29297	L	1026.59829	513.80278	342.87095	8
16	1983.92295	992.46511	661.97917	Q	913.51422	457.26075	305.17626	7
17	2084.97063	1042.98895	695.66173	T	785.45564	393.23146	262.49006	6
18	2271.04995	1136.02861	757.68817	W	684.40796	342.70762	228.80750	5
19	2368.10272	1184.55500	790.03909	P	498.32864	249.66796	166.78106	4
20	2481.18679	1241.09703	827.73378	I	401.27587	201.14157	134.43014	3
21	2580.25521	1290.63124	860.75659	V	288.19180	144.59954	96.73545	2
22				K-Acetyl	189.12338	95.06533	63.71264	1

Extracted from: Z:\712AB\SK17-S (Ac)\Chichi-Abaumannii-SK17-S-Acetyl_DrWu_20140512.raw #26137 RT: 79.01
ITMS, CID@35.00, z=+3, Mono m/z=923.45593 Da, MH+=2768.35324 Da, Match Tol.=0.6 Da

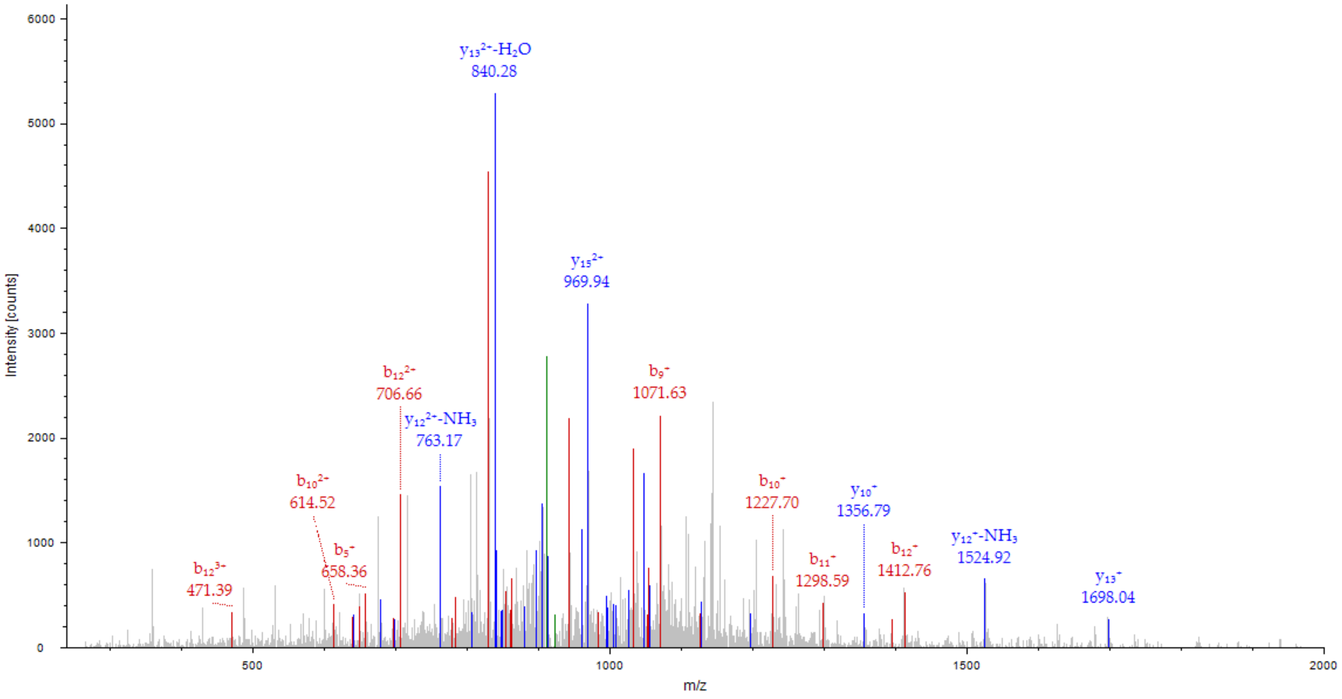

peg.1225 26260

#1	b^+	b^{2+}	b^{3+}	Seq.	y^+	y^{2+}	y^{3+}	#2
1	114.09135	57.54931	38.70197	L				35
2	229.11830	115.06279	77.04428	D	3933.92255	1967.46491	1311.97903	34
3	344.14525	172.57626	115.38660	D	3818.89560	1909.95144	1273.63672	33
4	441.19802	221.10265	147.73752	P	3703.86865	1852.43796	1235.29440	32
5	569.25660	285.13194	190.42372	Q	3606.81588	1803.91158	1202.94348	31
6	739.36213	370.18470	247.12556	K-Acetyl	3478.75730	1739.88229	1160.25728	30
7	852.44620	426.72674	284.82025	L	3308.65177	1654.82952	1103.55544	29
8	981.48880	491.24804	327.83445	E	3195.56770	1598.28749	1065.86075	28
9	1118.54771	559.77749	373.52075	H	3066.52510	1533.76619	1022.84655	27
10	1278.57837	639.79282	426.86431	C-Carbam···	2929.46619	1465.23673	977.16025	26
11	1335.59984	668.30356	445.87146	G	2769.43553	1385.22140	923.81669	25
12	1434.66826	717.83777	478.89427	V	2712.41406	1356.71067	904.80954	24
13	1563.71086	782.35907	521.90847	E	2613.34564	1307.17646	871.78673	23
14	1676.79493	838.90110	559.60316	L	2484.30304	1242.65516	828.77253	22
15	1823.86335	912.43531	608.62597	F	2371.21897	1186.11312	791.07784	21
16	1986.92667	993.96697	662.98041	Y	2224.15055	1112.57891	742.05503	20
17	2114.98525	1057.99626	705.66660	Q	2061.08723	1031.04725	687.70059	19
18	2243.08022	1122.04375	748.36492	K	1933.02865	967.01796	645.01440	18
19	2358.10717	1179.55722	786.70724	D	1804.93368	902.97048	602.31608	17
20	2471.19124	1236.09926	824.40193	L	1689.90673	845.45700	563.97376	16
21	2572.23892	1286.62310	858.08449	T	1576.82266	788.91497	526.27907	15
22	2659.27095	1330.13911	887.09517	S	1475.77498	738.39113	492.59651	14
23	2756.32372	1378.66550	919.44609	P	1388.74295	694.87511	463.58583	13
24	2869.40779	1435.20753	957.14078	L	1291.69018	646.34873	431.23491	12
25	2968.47621	1484.74174	990.16359	V	1178.60611	589.80669	393.54022	11
26	3055.50824	1528.25776	1019.17426	S	1079.53769	540.27248	360.51741	10
27	3112.52971	1556.76849	1038.18142	G	992.50566	496.75647	331.50674	9
28	3215.53890	1608.27309	1072.51782	C	935.48419	468.24573	312.49958	8
29	3328.62297	1664.81512	1110.21251	I	832.47500	416.74114	278.16318	7
30	3399.66009	1700.33368	1133.89155	A	719.39093	360.19910	240.46849	6
31	3585.73941	1793.37334	1195.91799	W	648.35381	324.68054	216.78945	5
32	3698.82348	1849.91538	1233.61268	L	462.27449	231.64088	154.76301	4
33	3797.89190	1899.44959	1266.63548	V	349.19042	175.09885	117.06832	3
34	3900.90109	1950.95418	1300.97188	C	250.12200	125.56464	84.04552	2
35				K	147.11281	74.06004	49.70912	1

Extracted from: Z:\712AB\SK17-S (Ac)\Chichi-Abaumannii-SK17-S-Acetyl_DrWu_20140703.raw #26260 RT: 71.90
ITMS, CID@35.00, z=+3, Mono m/z=1349.68164 Da, MH+=4047.03037 Da, Match Tol.=0.6 Da

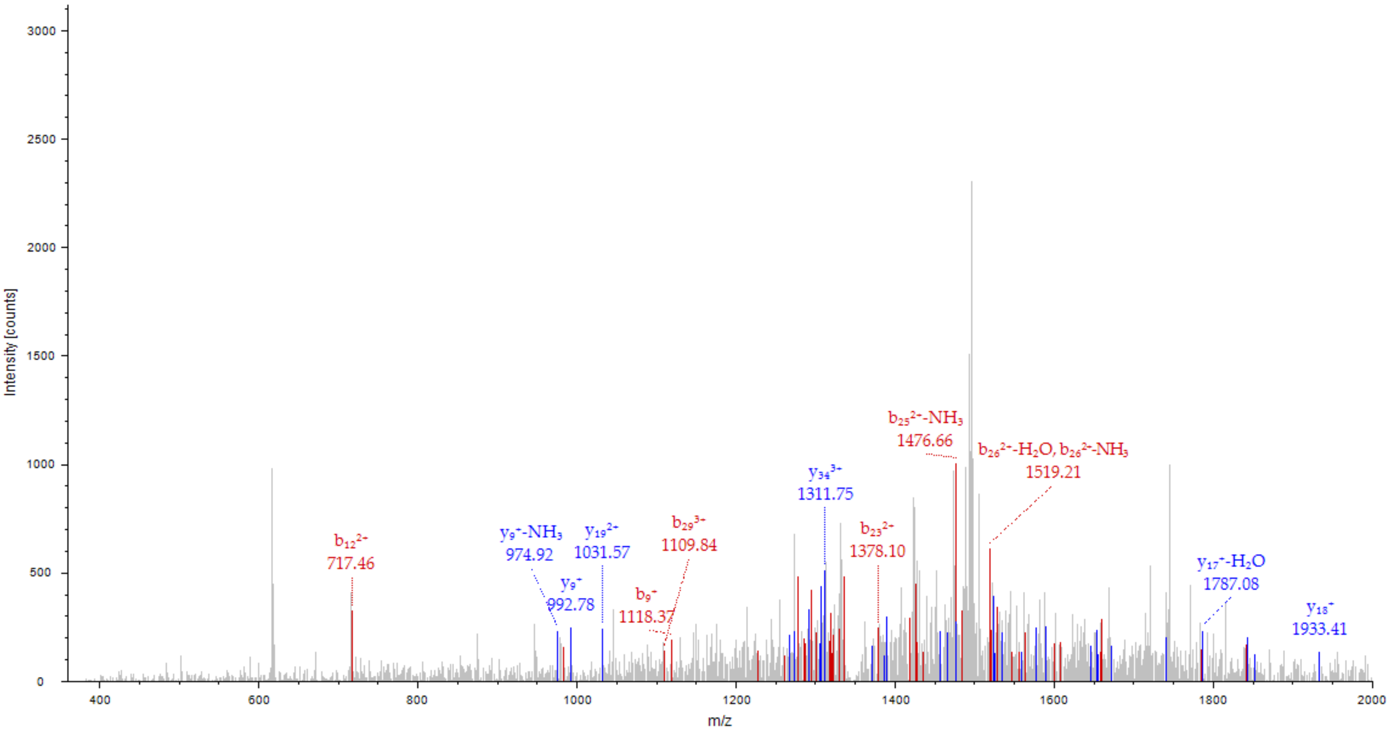

peg.1241   30175

#1	b+	b2+	b3+	Seq.	y+	y2+	y3+	#2
1	130.04987	65.52857	44.02147	S-Acetyl				30
2	244.09280	122.55004	82.03578	N	3256.64162	1628.82445	1086.21872	29
3	359.11975	180.06351	120.37810	D	3142.59869	1571.80298	1048.20441	28
4	458.18817	229.59772	153.40091	V	3027.57174	1514.28951	1009.86210	27
5	572.23110	286.61919	191.41522	N	2928.50332	1464.75530	976.83929	26
6	643.26822	322.13775	215.09426	A	2814.46039	1407.73383	938.82498	25
7	756.35229	378.67978	252.78895	L	2743.42327	1372.21527	915.14594	24
8	919.41561	460.21144	307.14339	Y	2630.33920	1315.67324	877.45125	23
9	1034.44256	517.72492	345.48570	D	2467.27588	1234.14158	823.09681	22
10	1165.48306	583.24517	389.16587	M	2352.24893	1176.62810	784.75449	21
11	1268.49225	634.74976	423.50227	C	2221.20843	1111.10785	741.07433	20
12	1381.57632	691.29180	461.19696	L	2118.19924	1059.60326	706.73793	19
13	1518.63523	759.82125	506.88326	H	2005.11517	1003.06122	669.04324	18
14	1589.67235	795.33981	530.56230	A	1868.05626	934.53177	623.35694	17
15	1717.73093	859.36910	573.24849	Q	1797.01914	899.01321	599.67790	16
16	1774.75240	887.87984	592.25565	G	1668.96056	834.98392	556.99170	15
17	1873.82082	937.41405	625.27846	V	1611.93909	806.47318	537.98455	14
18	1944.85794	972.93261	648.95750	A	1512.87067	756.93897	504.96174	13
19	2075.89844	1038.45286	692.63766	M	1441.83355	721.42041	481.28270	12
20	2188.98251	1094.99489	730.33235	L	1310.79305	655.90016	437.60253	11
21	2286.03528	1143.52128	762.68328	P	1197.70898	599.35813	399.90784	10
22	2387.08296	1194.04512	796.36584	T	1100.65621	550.83174	367.55692	9
23	2500.16703	1250.58715	834.06053	L	999.60853	500.30790	333.87436	8
24	2613.25110	1307.12919	871.75522	L	886.52446	443.76587	296.17967	7
25	2712.31952	1356.66340	904.77802	V	773.44039	387.22383	258.48498	6
26	2841.36212	1421.18470	947.79222	E	674.37197	337.68962	225.46217	5
27	3011.46766	1506.23747	1004.49407	K-Acetyl	545.32937	273.16832	182.44797	4
28	3126.49461	1563.75094	1042.83639	D	375.22383	188.11555	125.74613	3
29	3239.57868	1620.29298	1080.53108	L	260.19688	130.60208	87.40381	2
30				K	147.11281	74.06004	49.70912	1

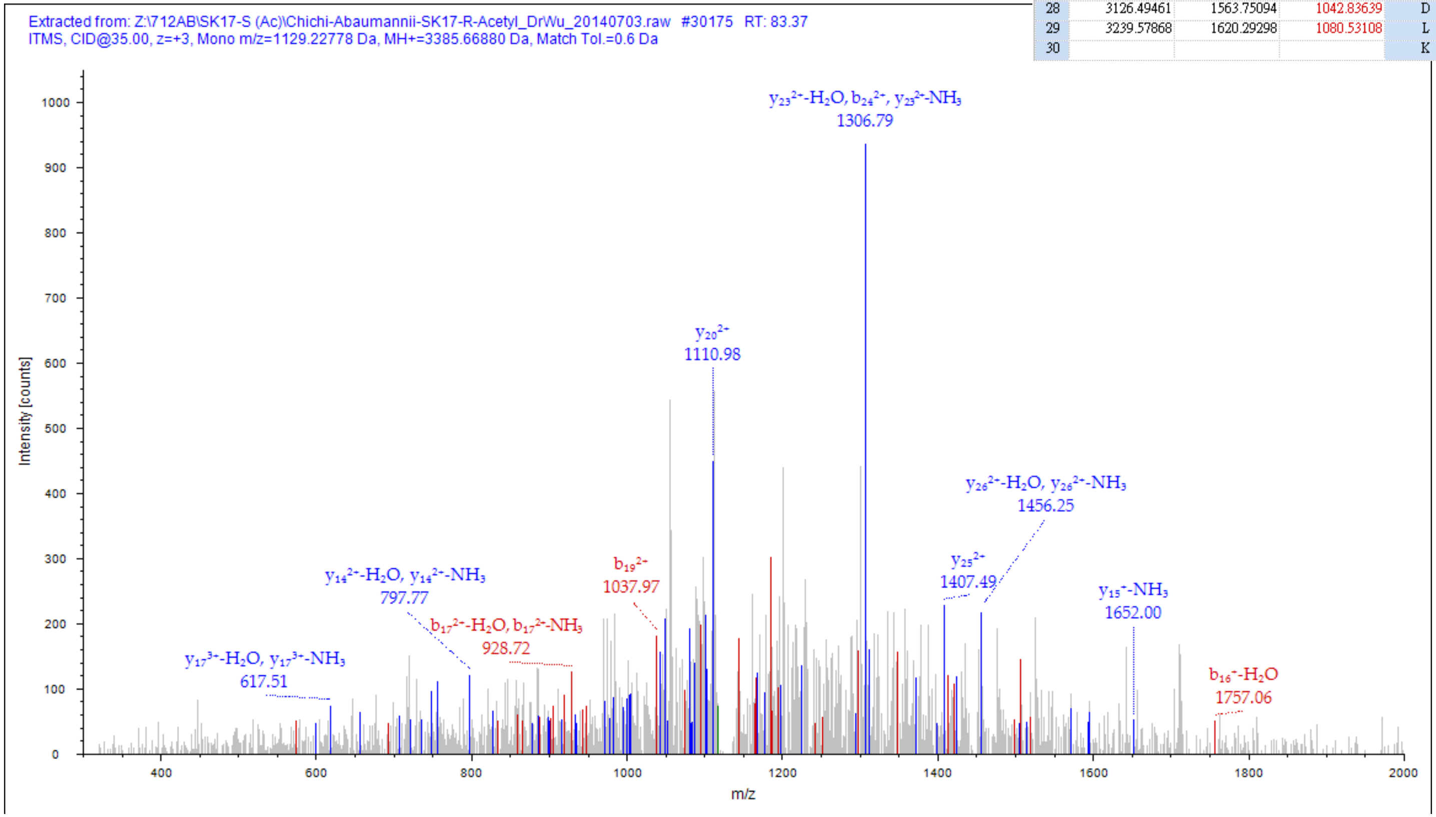

peg.1263 25699

#1	b^+	b^{2+}	b^{3+}	Seq.	y^+	y^{2+}	y^{3+}	#2
1	171.07642	86.04185	57.69699	Q-Acetyl				26
2	242.11354	121.56041	81.37603	A	2887.53223	1444.26975	963.18226	25
3	341.18196	171.09462	114.39884	V	2816.49511	1408.75119	939.50322	24
4	469.24054	235.12391	157.08503	Q	2717.42669	1359.21698	906.48041	23
5	598.28314	299.64521	200.09923	E	2589.36811	1295.18769	863.79422	22
6	701.29233	351.14980	234.43563	C	2460.32551	1230.66639	820.78002	21
7	814.37640	407.69184	272.13032	L	2357.31632	1179.16180	786.44362	20
8	961.41182	481.20955	321.14212	M-Oxidation	2244.23225	1122.61976	748.74893	19
9	1018.43329	509.72028	340.14928	G	2097.19684	1049.10206	699.73713	18
10	1146.49187	573.74957	382.83547	Q	2040.17537	1020.59132	680.72997	17
11	1259.57594	630.29161	420.53016	L	1912.11679	956.56203	638.04378	16
12	1387.63452	694.32090	463.21636	Q	1799.03272	900.02000	600.34909	15
13	1524.69343	762.85035	508.90266	H	1670.97414	835.99071	557.66290	14
14	1671.76185	836.38456	557.92547	F	1533.91523	767.46125	511.97659	13
15	1758.79388	879.90058	586.93614	S	1386.84681	693.92704	462.95379	12
16	1871.87795	936.44261	624.63083	L	1299.81478	650.41103	433.94311	11
17	1970.94637	985.97682	657.65364	V	1186.73071	593.86899	396.24842	10
18	2084.03044	1042.51886	695.34833	L	1087.66229	544.33478	363.22561	9
19	2254.13597	1127.57162	752.05017	K-Acetyl	974.57822	487.79275	325.53092	8
20	2351.18874	1176.09801	784.40110	P	804.47268	402.73998	268.82908	7
21	2422.22586	1211.61657	808.08014	A	707.41991	354.21359	236.47815	6
22	2585.28918	1293.14823	862.43458	Y	636.38279	318.69503	212.79911	5
23	2642.31065	1321.65896	881.44173	G	473.31947	237.16337	158.44467	4
24	2770.40562	1385.70645	924.14006	K	416.29800	208.65264	139.43752	3
25	2883.48969	1442.24848	961.83475	L	288.20303	144.60515	96.73919	2
26				R	175.11896	88.06312	59.04450	1

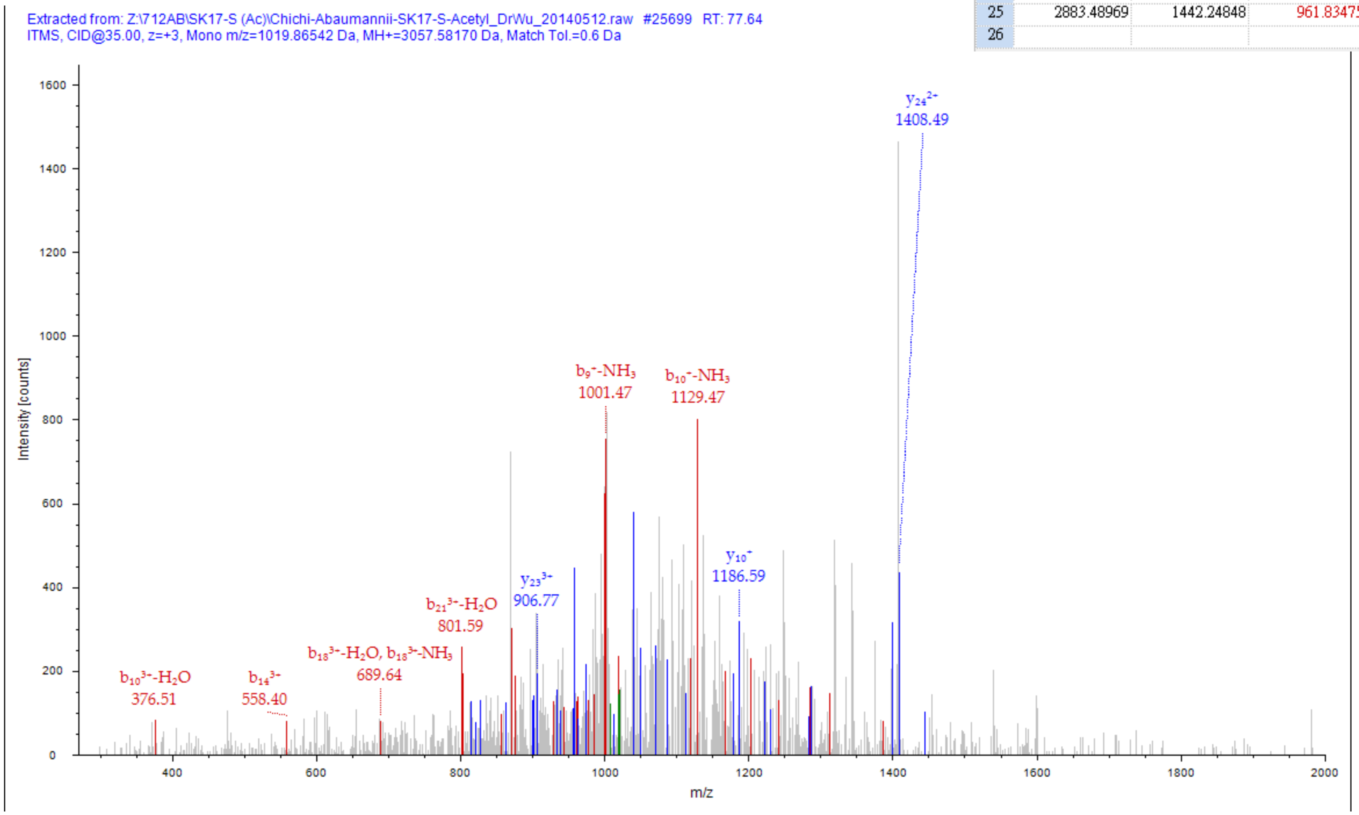

peg.1286 27953

#1	b⁺	b²⁺	b³⁺	Seq.	y⁺	y²⁺	y³⁺	#2
1	148.04269	74.52498	50.01908	M-Oxidation				35
2	249.09037	125.04882	83.70164	T	3812.87946	1906.94337	1271.63134	34
3	348.15879	174.58303	116.72445	V	3711.83178	1856.41953	1237.94878	33
4	477.20139	239.10433	159.73865	E	3612.76336	1806.88532	1204.92597	32
5	606.24399	303.62563	202.75285	E	3483.72076	1742.36402	1161.91177	31
6	753.31241	377.15984	251.77565	F	3354.67816	1677.84272	1118.89757	30
7	850.36518	425.68623	284.12658	P	3207.60974	1604.30851	1069.87476	29
8	964.40811	482.70769	322.14089	N	3110.55697	1555.78212	1037.52384	28
9	1077.49218	539.24973	359.83558	I	2996.51404	1498.76066	999.50953	27
10	1192.51913	596.76320	398.17789	D	2883.42997	1442.21862	961.81484	26
11	1339.58755	670.29741	447.20070	F	2768.40302	1384.70515	923.47252	25
12	1436.64032	718.82380	479.55162	P	2621.33460	1311.17094	874.44972	24
13	1583.70874	792.35801	528.57443	F	2524.28183	1262.64455	842.09879	23
14	1682.77716	841.89222	561.59724	V	2377.21341	1189.11034	793.07599	22
15	1781.84558	891.42643	594.62004	V	2278.14499	1139.57613	760.05318	21
16	1880.91400	940.96064	627.64285	V	2179.07657	1090.04192	727.03037	20
17	1981.96168	991.48448	661.32541	T	2080.00815	1040.50771	694.00757	19
18	2083.00936	1042.00832	695.00797	T	1978.96047	989.98387	660.32501	18
19	2211.06794	1106.03761	737.69416	Q	1877.91279	939.46003	626.64245	17
20	2374.13126	1187.56927	792.04860	Y	1749.85421	875.43074	583.95625	16
21	2445.16838	1223.08783	815.72764	A	1586.79089	793.89908	529.60181	15
22	2502.18985	1251.59856	834.73480	G	1515.75377	758.38052	505.92277	14
23	2573.22697	1287.11712	858.41384	A	1458.73230	729.86979	486.91562	13
24	2660.25900	1330.63314	887.42452	S	1387.69518	694.35123	463.23658	12
25	2757.31177	1379.15952	919.77544	P	1300.66315	650.83521	434.22590	11
26	2886.35437	1443.68082	962.78964	E	1203.61038	602.30883	401.87498	10
27	2957.39149	1479.19938	986.46868	A	1074.56778	537.78753	358.86078	9
28	3056.45991	1528.73359	1019.49149	V	1003.53066	502.26897	335.18174	8
29	3185.50251	1593.25489	1062.50569	E	904.46224	452.73476	302.15893	7
30	3272.53454	1636.77091	1091.51636	S	775.41964	388.21346	259.14473	6
31	3387.56149	1694.28438	1129.85868	D	688.38761	344.69744	230.13405	5
32	3500.64556	1750.82642	1167.55337	I	573.36066	287.18397	191.79174	4
33	3601.69324	1801.35026	1201.23593	T	460.27659	230.64193	154.09705	3
34	3771.79878	1886.40303	1257.93778	K-Acetyl	359.22891	180.11809	120.41449	2
35				K-Acetyl	189.12338	95.06533	63.71264	1

Extracted from: Z:\712AB\SK17-S (Ac)\Chichi-Abaumannii-SK17-R-Acetyl_DrWu_20140512.raw #27953 RT: 83.41
ITMS, CID@35.00, z=+3, Mono m/z=1320.64771 Da, MH+=3959.92856 Da, Match Tol.=0.6 Da

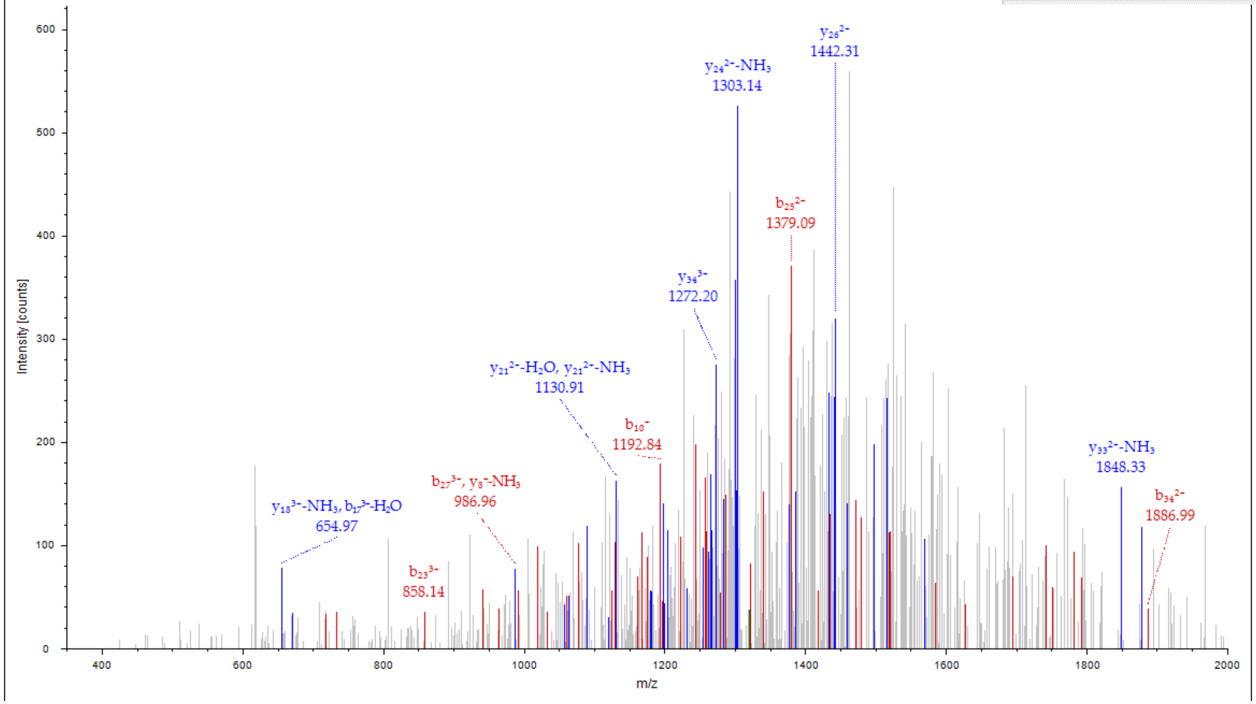

peg.13 16218

#1	b^+	b^{2+}	Seq.	y^+	y^{2+}	#2
1	58.02875	29.51801	G			22
2	115.05022	58.02875	G	1848.90224	924.95476	21
3	243.14519	122.07623	K	1791.88077	896.44402	20
4	300.16666	150.58697	G	1663.78580	832.39654	19
5	401.21434	201.11081	T	1606.76433	803.88580	18
6	458.23581	229.62154	G	1505.71665	753.36196	17
7	515.25728	258.13228	G	1448.69518	724.85123	16
8	572.27875	286.64301	G	1391.67371	696.34049	15
9	719.34717	360.17722	F	1334.65224	667.82976	14
10	847.40575	424.20651	Q	1187.58382	594.29555	13
11	904.42722	452.71725	G	1059.52524	530.26626	12
12	961.44869	481.22798	G	1002.50377	501.75552	11
13	1018.47016	509.73872	G	945.48230	473.24479	10
14	1188.57569	594.79148	K-Acetyl	888.46083	444.73405	9
15	1245.59716	623.30222	G	718.35529	359.68128	8
16	1302.61863	651.81295	G	661.33382	331.17055	7
17	1373.65575	687.33151	A	604.31235	302.65981	6
18	1470.70852	735.85790	P	533.27523	267.14125	5
19	1571.75620	786.38174	T	436.22246	218.61487	4
20	1628.77767	814.89247	G	335.17478	168.09103	3
21	1759.81817	880.41272	M	278.15331	139.58029	2
22			K	147.11281	74.06004	1

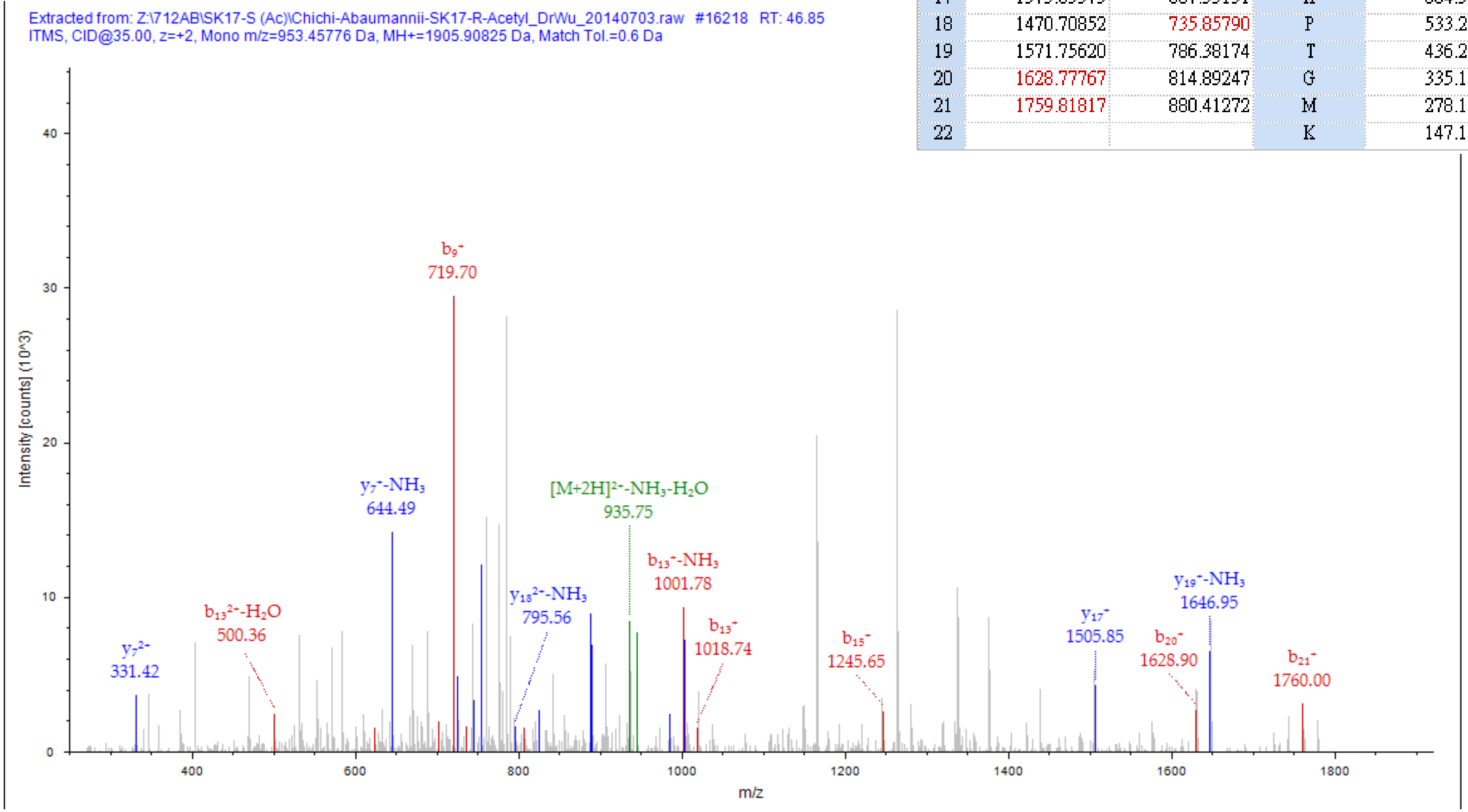

peg.13 14419

#1	b^+	b^{2+}	Seq.	y^+	y^{2+}	#2
1	58.02875	29.51801	G			22
2	115.05022	58.02875	G	1848.90224	924.95476	21
3	243.14519	122.07623	K	1791.88077	896.44402	20
4	300.16666	150.58697	G	1663.78580	832.39654	19
5	401.21434	201.11081	T	1606.76433	803.88580	18
6	458.23581	229.62154	G	1505.71665	753.36196	17
7	515.25728	258.13228	G	1448.69518	724.85123	16
8	572.27875	286.64301	G	1391.67371	696.34049	15
9	719.34717	360.17722	F	1334.65224	667.82976	14
10	847.40575	424.20651	Q	1187.58382	594.29555	13
11	904.42722	452.71725	G	1059.52524	530.26626	12
12	961.44869	481.22798	G	1002.50377	501.75552	11
13	1018.47016	509.73872	G	945.48230	473.24479	10
14	1188.57569	594.79148	K-Acetyl	888.46083	444.73405	9
15	1245.59716	623.30222	G	718.35529	359.68128	8
16	1302.61863	651.81295	G	661.33382	331.17055	7
17	1373.65575	687.33151	A	604.31235	302.65981	6
18	1470.70852	735.85790	P	533.27523	267.14125	5
19	1571.75620	786.38174	T	436.22246	218.61487	4
20	1628.77767	814.89247	G	335.17478	168.09103	3
21	1759.81817	880.41272	M	278.15331	139.58029	2
22			K	147.11281	74.06004	1

Extracted from: Z:\712AB\SK17-S (Ac)\Chichi-Abaumannii-SK17-S-Acetyl_DrWu_20140512.raw #14419 RT: 46.08
ITMS, CID@35.00, z=+2, Mono m/z=953.45630 Da, MH+=1905.90532 Da, Match Tol.=0.6 Da

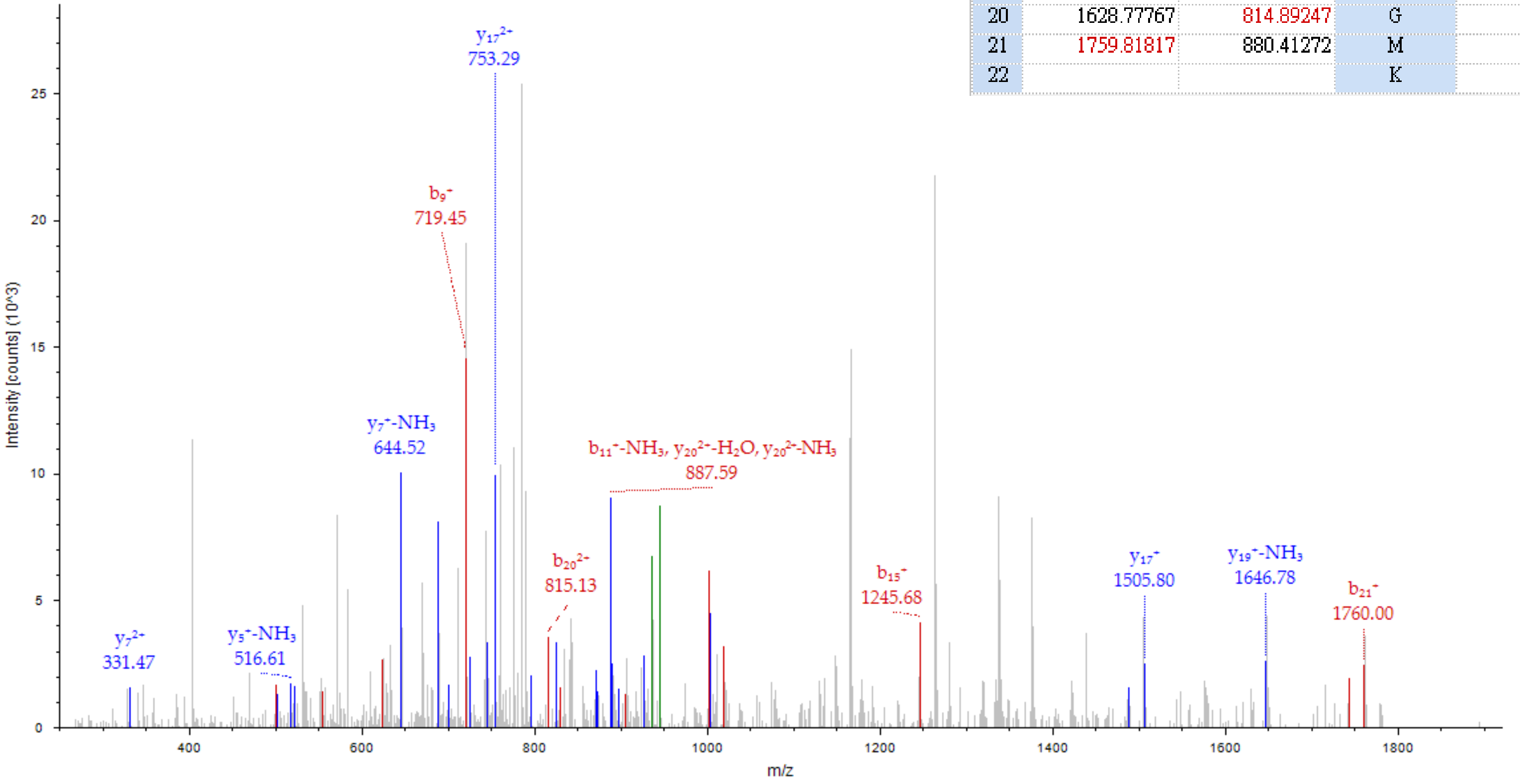

peg.1307   9072

#1	b⁺	b^{2+}	b^{3+}	Seq.	y⁺	y^{2+}	y^{3+}	#2
1	114.09135	57.54931	38.70197	L				17
2	171.11282	86.06005	57.70912	G	1660.81779	830.91253	554.27745	16
3	284.19689	142.60208	95.40381	L	1603.79632	802.40180	535.27029	15
4	399.22384	200.11556	133.74613	D	1490.71225	745.85976	497.57560	14
5	546.29226	273.64977	182.76894	F	1375.68530	688.34629	459.23328	13
6	660.33519	330.67123	220.78325	N	1228.61688	614.81208	410.21048	12
7	761.38287	381.19507	254.46581	T	1114.57395	557.79061	372.19617	11
8	858.43564	429.72146	286.81673	P	1013.52627	507.26677	338.51361	10
9	957.50406	479.25567	319.83954	V	916.47350	458.74039	306.16268	9
10	1014.52553	507.76640	338.84669	G	817.40508	409.20618	273.13988	8
11	1129.55248	565.27988	377.18901	D	760.38361	380.69544	254.13272	7
12	1244.57943	622.79335	415.53133	D	645.35666	323.18197	215.79040	6
13	1414.68496	707.84612	472.23317	K-Acetyl	530.32971	265.66849	177.44809	5
14	1513.75338	757.38033	505.25598	V	360.22417	180.61572	120.74624	4
15	1570.77485	785.89106	524.26313	G	261.15575	131.08151	87.72343	3
16	1627.79632	814.40180	543.27029	G	204.13428	102.57078	68.71628	2
17				K	147.11281	74.06004	49.70912	1

Extracted from: Z:\712AB\SK17-S (Ac)\Chichi-Abaumannii-SK17-R-Acetyl_DrWu_20140703.raw #9072 RT: 29.95
ITMS, CID@35.00, z=+3, Mono m/z=591.97070 Da, MH+=1773.89756 Da, Match Tol.=0.6 Da

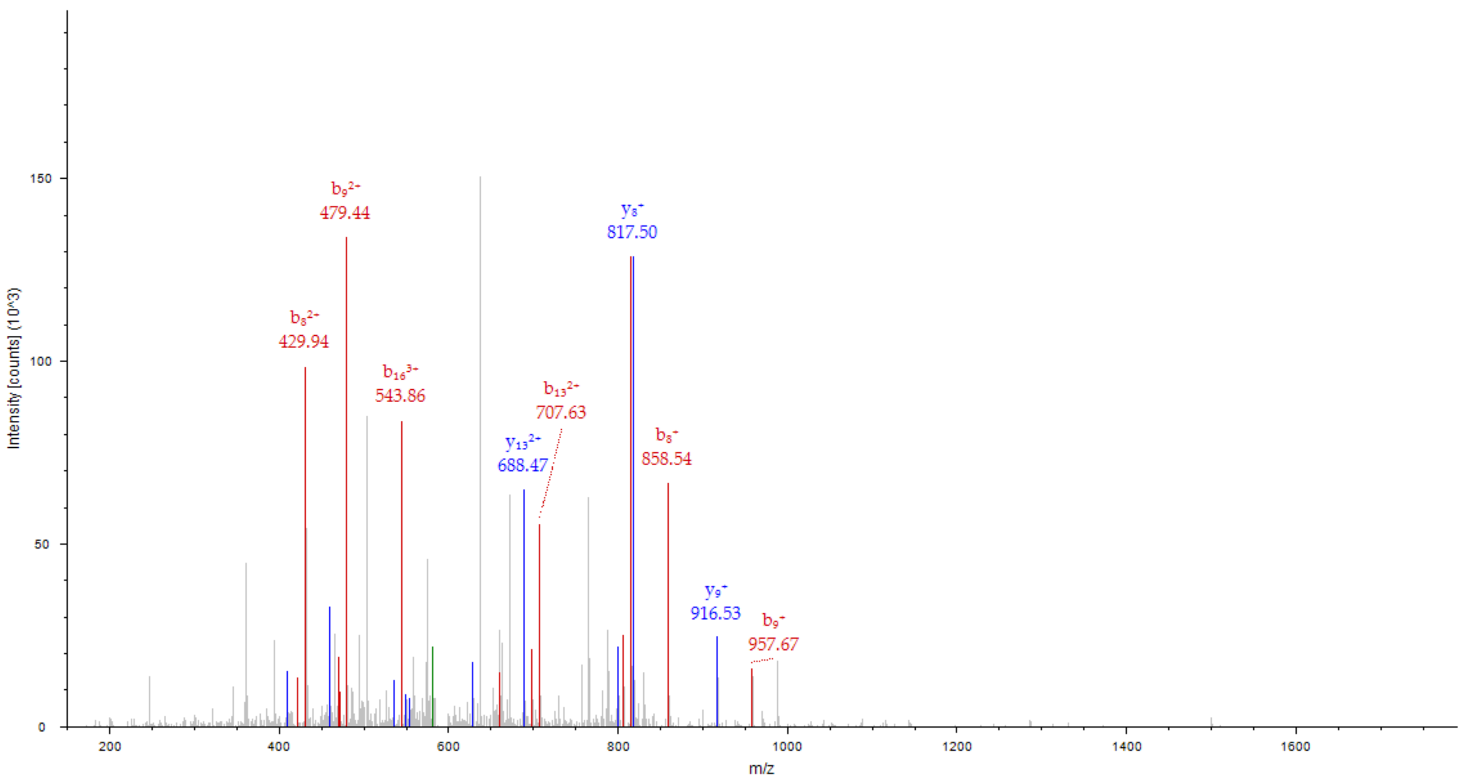

peg.1307 9541

#1	b+	b2+	b3+	Seq.	y+	y2+	y3+	#2
1	114.09135	57.54931	38.70197	L				17
2	171.11282	86.06005	57.70912	G	1660.81779	830.91253	554.27745	16
3	284.19689	142.60208	95.40381	L	1603.79632	802.40180	535.27029	15
4	399.22384	200.11556	133.74613	D	1490.71225	745.85976	497.57560	14
5	546.29226	273.64977	182.76894	F	1375.68530	688.34629	459.23328	13
6	660.33519	330.67123	220.78325	N	1228.61688	614.81208	410.21048	12
7	761.38287	381.19507	254.46581	T	1114.57395	557.79061	372.19617	11
8	858.43564	429.72146	286.81673	P	1013.52627	507.26677	338.51361	10
9	957.50406	479.25567	319.83954	V	916.47350	458.74039	306.16268	9
10	1014.52553	507.76640	338.84669	G	817.40508	409.20618	273.13988	8
11	1129.55248	565.27988	377.18901	D	760.38361	380.69544	254.13272	7
12	1244.57943	622.79335	415.53133	D	645.35666	323.18197	215.79040	6
13	1414.68496	707.84612	472.23317	K-Acetyl	530.32971	265.66849	177.44809	5
14	1513.75338	757.38033	505.25598	V	360.22417	180.61572	120.74624	4
15	1570.77485	785.89106	524.26313	G	261.15575	131.08151	87.72343	3
16	1627.79632	814.40180	543.27029	G	204.13428	102.57078	68.71628	2
17				K	147.11281	74.06004	49.70912	1

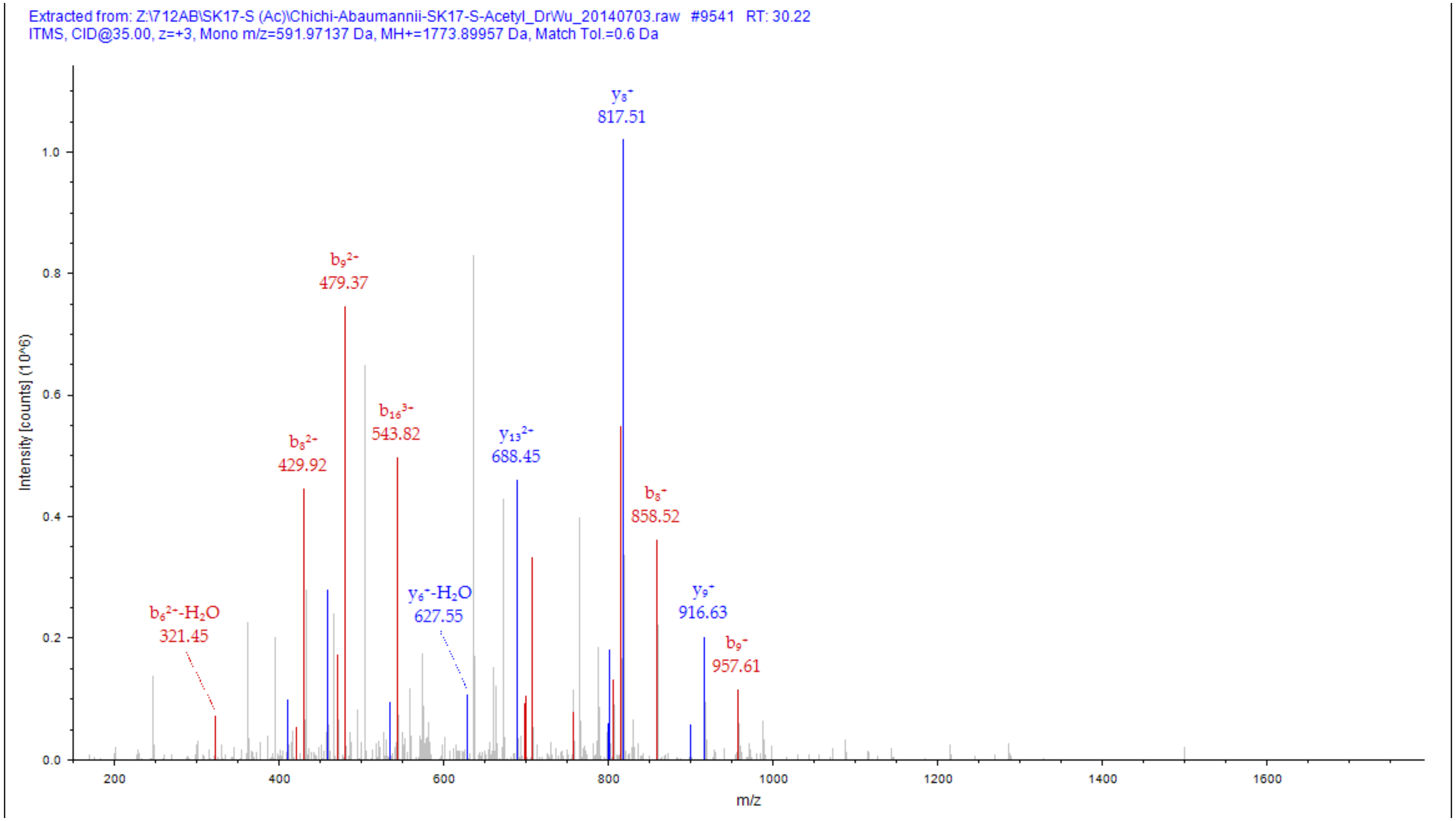

peg.1324   25617

#1	b+	b2+	b3+	Seq.	y+	y2+	y3+	#2
1	132.04778	66.52753	44.68744	M				19
2	245.13185	123.06956	82.38213	L	2079.03157	1040.01942	693.68204	18
3	360.15880	180.58304	120.72445	D	1965.94750	983.47739	655.98735	17
4	489.20140	245.10434	163.73865	E	1850.92055	925.96391	617.64503	16
5	602.28547	301.64637	201.43334	I	1721.87795	861.44261	574.63083	15
6	659.30694	330.15711	220.44050	G	1608.79388	804.90058	536.93614	14
7	746.33897	373.67312	249.45117	S	1551.77241	776.38984	517.92899	13
8	845.40739	423.20733	282.47398	V	1464.74038	732.87383	488.91831	12
9	974.44999	487.72863	325.48818	E	1365.67196	683.33962	455.89550	11
10	1088.49292	544.75010	363.50249	N	1236.62936	618.81832	412.88130	10
11	1187.56134	594.28431	396.52530	V	1122.58643	561.79685	374.86699	9
12	1258.59846	629.80287	420.20434	A	1023.51801	512.26264	341.84419	8
13	1387.64106	694.32417	463.21854	E	952.48089	476.74408	318.16515	7
14	1534.70948	767.85838	512.24134	F	823.43829	412.22278	275.15095	6
15	1665.74998	833.37863	555.92151	M	676.36987	338.68857	226.12814	5
16	1794.79258	897.89993	598.93571	E	545.32937	273.16832	182.44797	4
17	1964.89811	982.95269	655.63755	K-Acetyl	416.28677	208.64702	139.43377	3
18	2063.96653	1032.48690	688.66036	V	246.18123	123.59425	82.73193	2
19				K	147.11281	74.06004	49.70912	1

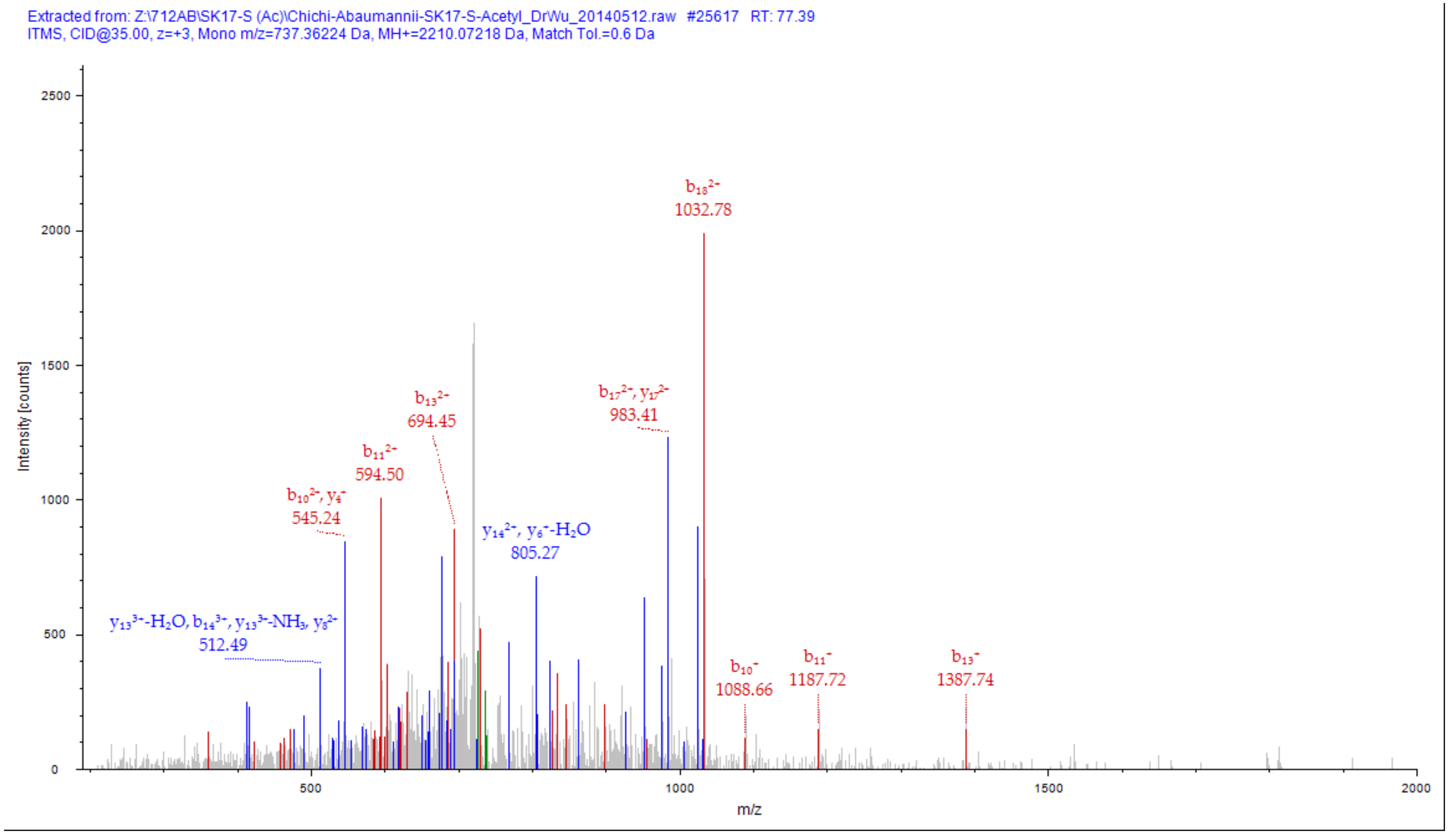

peg.1331 23320

#1	b+	b2+	Seq.	y+	y2+	#2
1	132.04778	66.52753	M			15
2	245.13185	123.06956	I	1501.86255	751.43491	14
3	360.15880	180.58304	D	1388.77848	694.89288	13
4	417.18027	209.09377	G	1273.75153	637.37940	12
5	587.28580	294.14654	K-Acetyl	1216.73006	608.86867	11
6	716.32840	358.66784	E	1046.62452	523.81590	10
7	787.36552	394.18640	A	917.58192	459.29460	9
8	886.43394	443.72061	V	846.54480	423.77604	8
9	943.45541	472.23134	G	747.47638	374.24183	7
10	1090.52383	545.76555	F	690.45491	345.73109	6
11	1203.60790	602.30759	L	543.38649	272.19688	5
12	1302.67632	651.84180	V	430.30242	215.65485	4
13	1373.71344	687.36036	A	331.23400	166.12064	3
14	1486.79751	743.90239	I	260.19688	130.60208	2
15			K	147.11281	74.06004	1

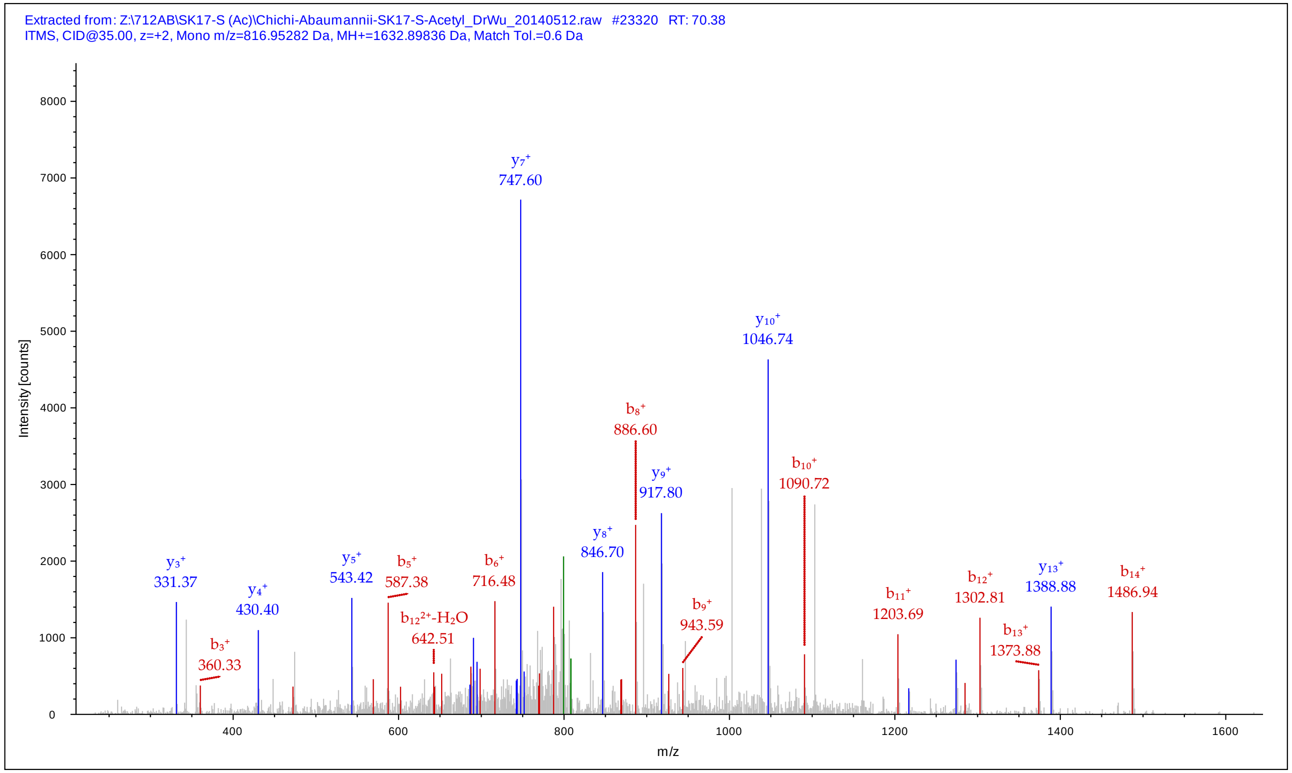

peg.1331 4726

#1	b^+	b^{2+}	Seq.	y^+	y^{2+}	#2
1	129.10225	65.05476	K			9
2	257.16083	129.08405	Q	1070.51534	535.76131	8
3	420.22415	210.61571	Y	942.45676	471.73202	7
4	590.32968	295.66848	K-Acetyl	779.39344	390.20036	6
5	705.35663	353.18195	D	609.28790	305.14759	5
6	776.39375	388.70051	A	494.26095	247.63411	4
7	923.46217	462.23472	F	423.22383	212.11555	3
8	1052.50477	526.75602	E	276.15541	138.58134	2
9			K	147.11281	74.06004	1

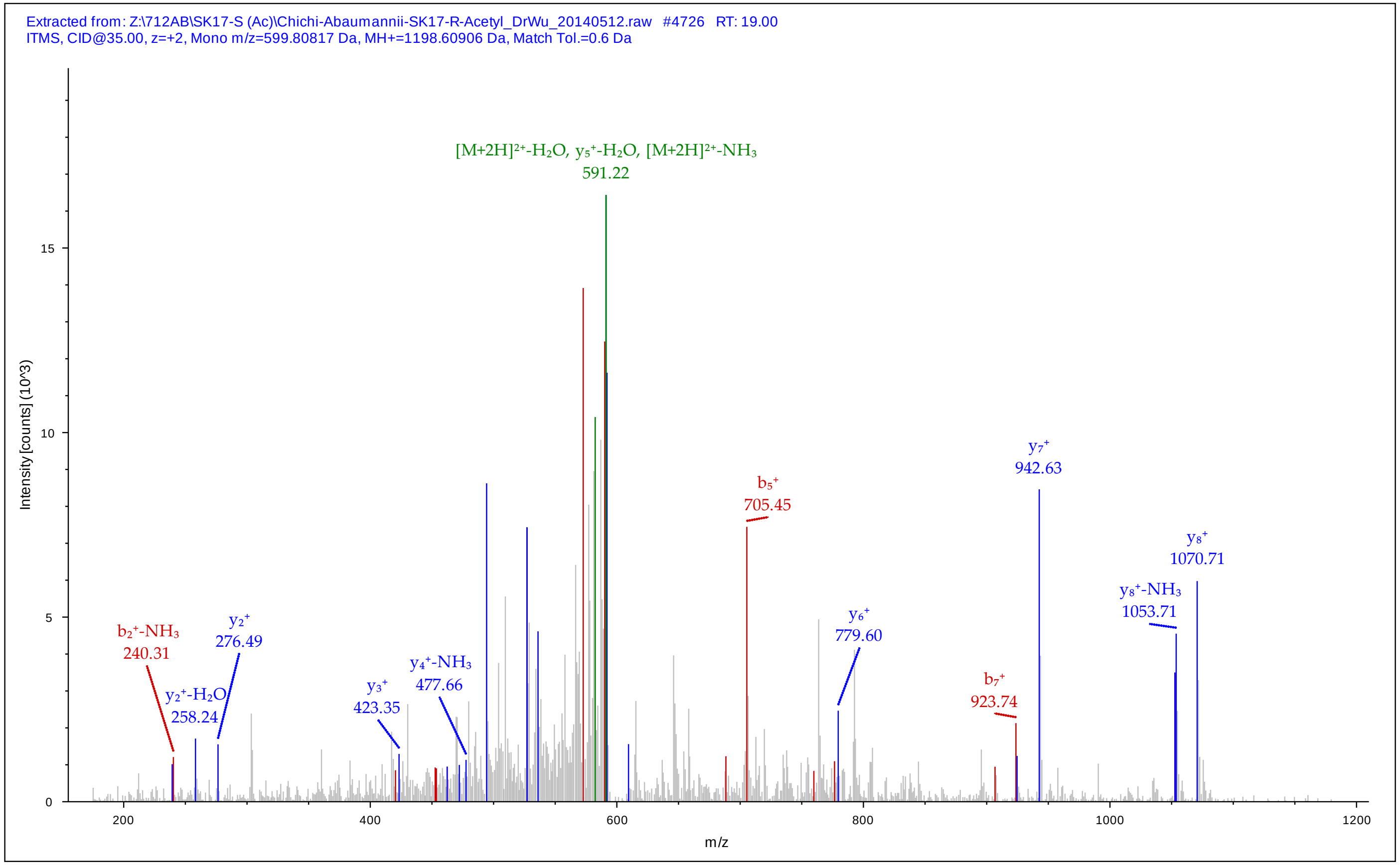

peg.1331 3652

#1	b+	b2+	Seq.	y+	y2+	#2
1	129.10225	65.05476	K			9
2	257.16083	129.08405	Q	1070.51534	535.76131	8
3	420.22415	210.61571	Y	942.45676	471.73202	7
4	590.32968	295.66848	K-Acetyl	779.39344	390.20036	6
5	705.35663	353.18195	D	609.28790	305.14759	5
6	776.39375	388.70051	A	494.26095	247.63411	4
7	923.46217	462.23472	F	423.22383	212.11555	3
8	1052.50477	526.75602	E	276.15541	138.58134	2
9			K	147.11281	74.06004	1

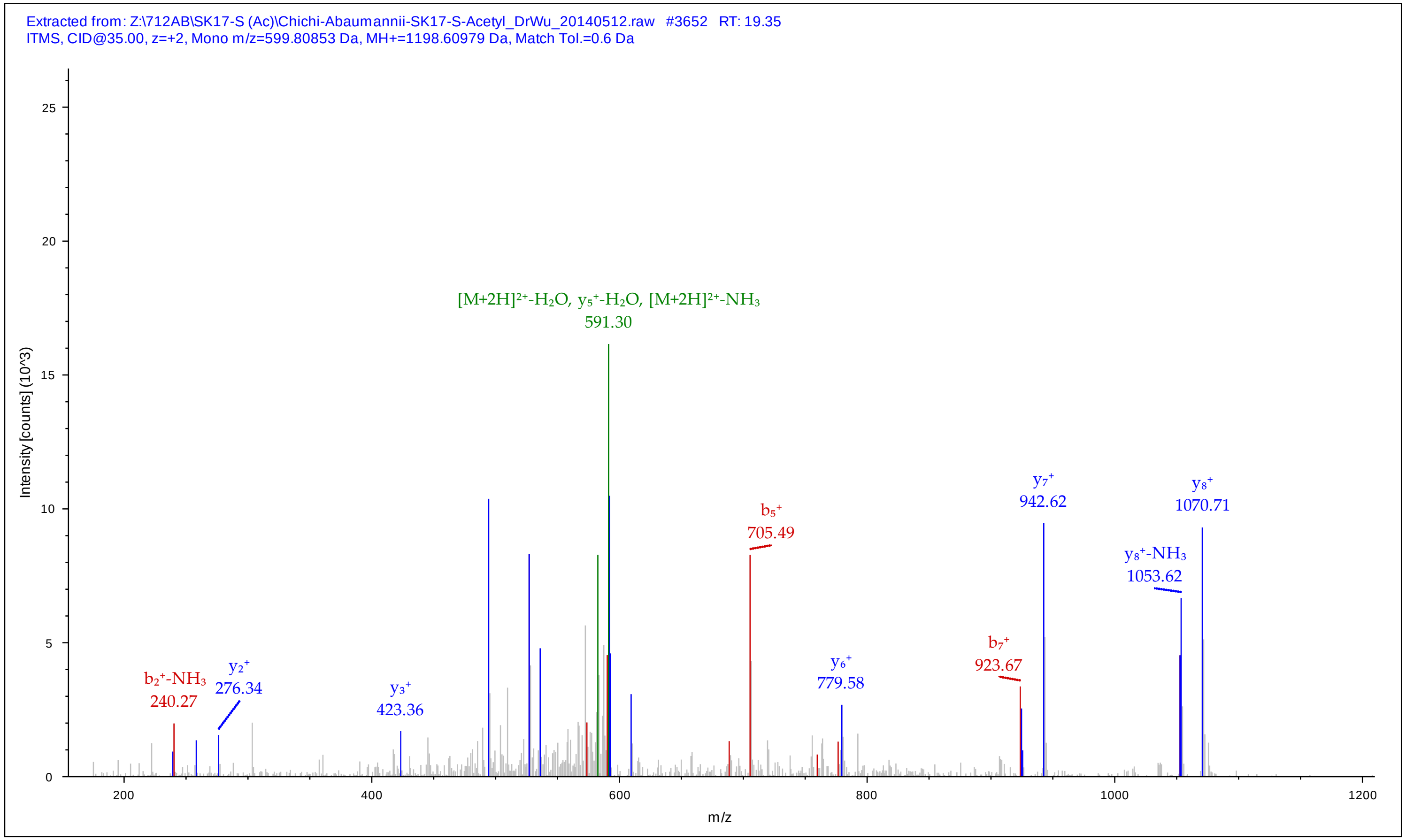

peg.1342 29761

#1	b^{+}	b^{2+}	b^{3+}	Seq.	y^{+}	y^{2+}	y^{3+}	#2
1	148.07570	74.54149	50.03008	F				31
2	205.09717	103.05222	69.03724	G	3899.91812	1950.46270	1300.64422	30
3	318.18124	159.59426	106.73193	L	3842.89665	1921.95196	1281.63707	29
4	446.23982	223.62355	149.41812	Q	3729.81258	1865.40993	1243.94238	28
5	559.32389	280.16558	187.11281	I	3601.75400	1801.38064	1201.25618	27
6	673.36682	337.18705	225.12712	N	3488.66993	1744.83860	1163.56149	26
7	820.43524	410.72126	274.14993	F	3374.62700	1687.81714	1125.54718	25
8	983.49856	492.25292	328.50437	Y	3227.55858	1614.28293	1076.52438	24
9	1040.52003	520.76365	347.51153	G	3064.49526	1532.75127	1022.16994	23
10	1187.58845	594.29786	396.53433	F	3007.47379	1504.24053	1003.16278	22
11	1274.62048	637.81388	425.54501	S	2860.40537	1430.70632	954.13997	21
12	1402.67906	701.84317	468.23120	Q	2773.37334	1387.19031	925.12930	20
13	1530.73764	765.87246	510.91740	Q	2645.31476	1323.16102	882.44310	19
14	1659.78024	830.39376	553.93160	E	2517.25618	1259.13173	839.75691	18
15	1822.84356	911.92542	608.28604	Y	2388.21358	1194.61043	796.74271	17
16	1935.92763	968.46745	645.98073	L	2225.15026	1113.07877	742.38827	16
17	2064.02260	1032.51494	688.67905	K	2112.06619	1056.53673	704.69358	15
18	2165.07028	1083.03878	722.36161	T	1983.97122	992.48925	661.99526	14
19	2264.13870	1132.57299	755.38442	V	1882.92354	941.96541	628.31270	13
20	2392.19728	1196.60228	798.07061	Q	1783.85512	892.43120	595.28989	12
21	2555.26060	1278.13394	852.42505	Y	1655.79654	828.40191	552.60370	11
22	2741.33992	1371.17360	914.45149	W	1492.73322	746.87025	498.24926	10
23	2854.42399	1427.71563	952.14618	L	1306.65390	653.83059	436.22282	9
24	2968.46692	1484.73710	990.16049	N	1193.56983	597.28855	398.52813	8
25	3097.50952	1549.25840	1033.17469	E	1079.52690	540.26709	360.51382	7
26	3260.57284	1630.79006	1087.52913	Y	950.48430	475.74579	317.49962	6
27	3430.67837	1715.84282	1144.23097	K-Acetyl	787.42098	394.21413	263.14518	5
28	3616.75769	1808.88248	1206.25741	W	617.31544	309.16136	206.44333	4
29	3744.81627	1872.91177	1248.94361	Q	431.23612	216.12170	144.41689	3
30	3872.87485	1936.94106	1291.62980	Q	303.17754	152.09241	101.73070	2
31				R	175.11896	88.06312	59.04450	1

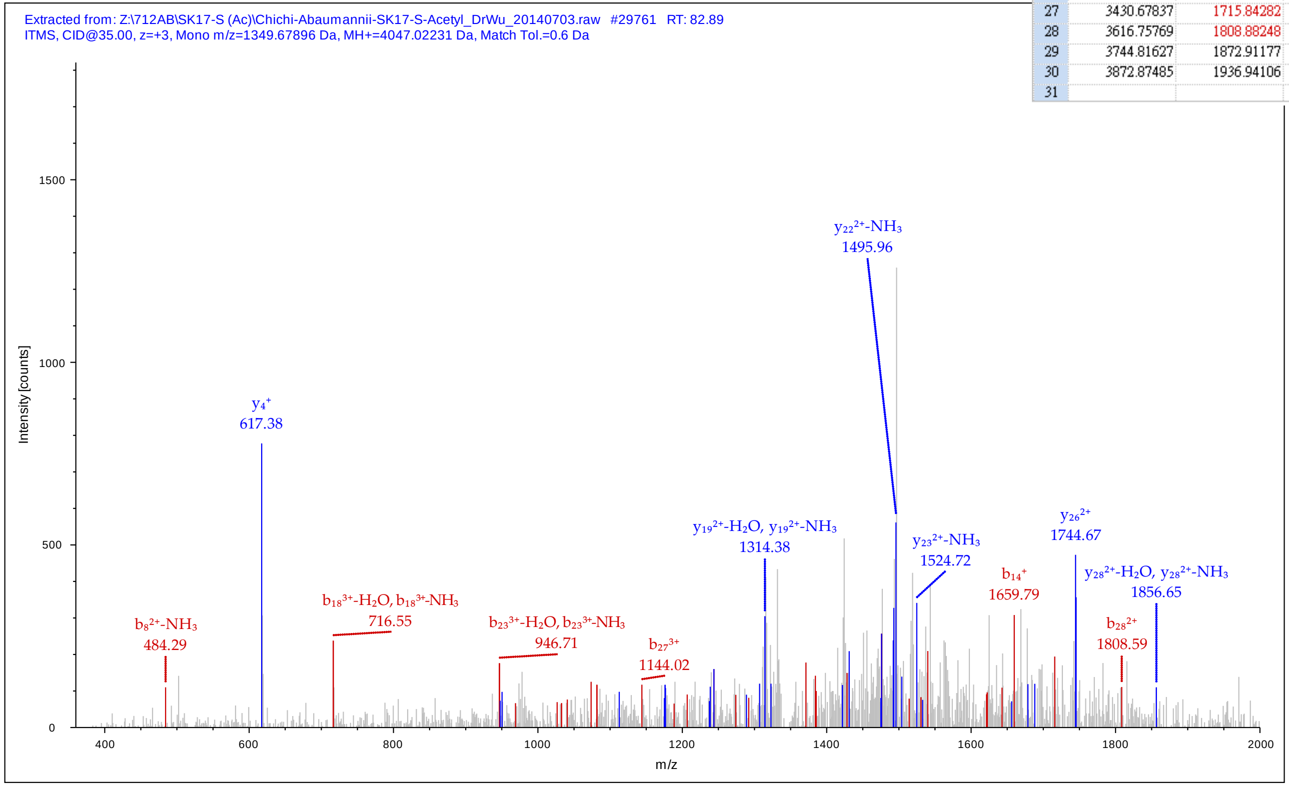

peg.1381   19691

#1	b⁺	b²⁺	Seq.	y⁺	y²⁺	#2
1	129.06586	65.03657	Q			17
2	226.11863	113.56295	P	1709.86803	855.43765	16
3	297.15575	149.08151	A	1612.81526	806.91127	15
4	384.18778	192.59753	S	1541.77814	771.39271	14
5	531.25620	266.13174	F	1454.74611	727.87669	13
6	634.26539	317.63633	C	1307.67769	654.34248	12
7	691.28686	346.14707	G	1204.66850	602.83789	11
8	804.37093	402.68910	L	1147.64703	574.32715	10
9	905.41861	453.21294	T	1034.56296	517.78512	9
10	962.44008	481.72368	G	933.51528	467.26128	8
11	1075.52415	538.26571	L	876.49381	438.75054	7
12	1245.62968	623.31848	K-Acetyl	763.40974	382.20851	6
13	1342.68245	671.84486	P	593.30420	297.15574	5
14	1443.73013	722.36870	T	496.25143	248.62935	4
15	1606.79345	803.90036	Y	395.20375	198.10551	3
16	1663.81492	832.41110	G	232.14043	116.57385	2
17			R	175.11896	88.06312	1

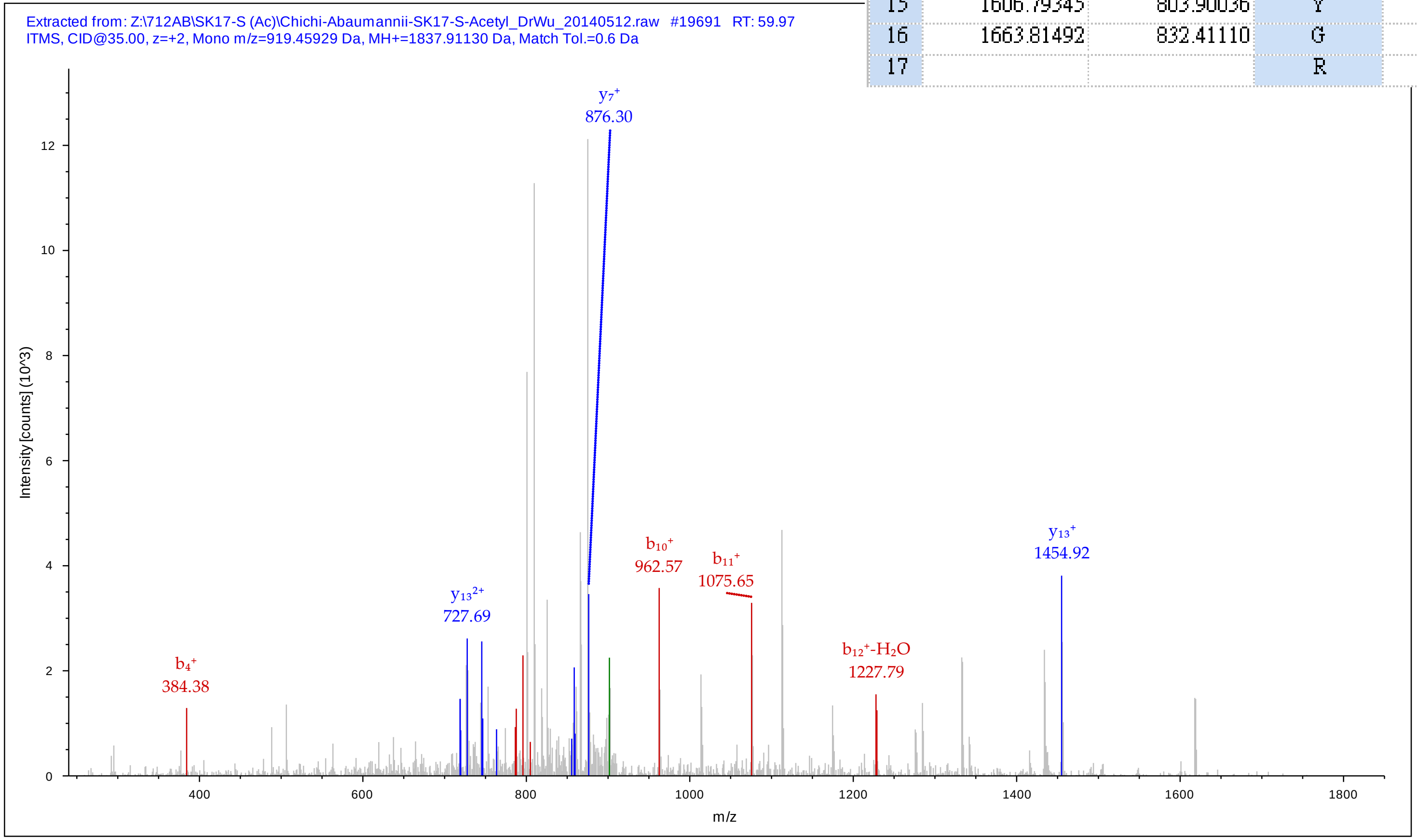

peg.1389 25722

1	171.11281	86.06004	57.70912	K-Acetyl				33
2	268.16558	134.58643	90.06004	P	3449.82450	1725.41589	1150.61302	32
3	381.24965	191.12846	127.75473	L	3352.77173	1676.88950	1118.26209	31
4	494.33372	247.67050	165.44942	I	3239.68766	1620.34747	1080.56740	30
5	565.37084	283.18906	189.12846	A	3126.60359	1563.80543	1042.87271	29
6	696.41134	348.70931	232.80863	M	3055.56647	1528.28687	1019.19367	28
7	795.47976	398.24352	265.83144	V	2924.52597	1462.76662	975.51351	27
8	892.53253	446.76990	298.18236	P	2825.45755	1413.23241	942.49070	26
9	963.56965	482.28846	321.86140	A	2728.40478	1364.70603	910.13978	25
10	1050.60168	525.80448	350.87208	S	2657.36766	1329.18747	886.46074	24
11	1163.68575	582.34651	388.56677	L	2570.33563	1285.67145	857.45006	23
12	1319.78687	660.39707	440.60047	R	2457.25156	1229.12942	819.75537	22
13	1420.83455	710.92091	474.28303	T	2301.15044	1151.07886	767.72166	21
14	1535.86150	768.43439	512.62535	D	2200.10276	1100.55502	734.03910	20
15	1650.88845	825.94786	550.96767	D	2085.07581	1043.04154	695.69679	19
16	1737.92048	869.46388	579.97834	S	1970.04886	985.52807	657.35447	18
17	1852.94743	926.97735	618.32066	D	1883.01683	942.01205	628.34379	17
18	1952.01585	976.51156	651.34347	V	1767.98988	884.49858	590.00148	16
19	2039.04788	1020.02758	680.35414	S	1668.92146	834.96437	556.97867	15
20	2153.09081	1077.04904	718.36845	N	1581.88943	791.44835	527.96799	14
21	2309.19193	1155.09960	770.40216	R	1467.84650	734.42689	489.95368	13
22	2422.27600	1211.64164	808.09685	I	1311.74538	656.37633	437.91998	12
23	2523.32368	1262.16548	841.77941	T	1198.66131	599.83429	400.22529	11
24	2654.36418	1327.68573	885.45958	M	1097.61363	549.31045	366.54273	10
25	2767.44825	1384.22776	923.15427	I	966.57313	483.79020	322.86256	9
26	2880.53232	1440.76980	960.84896	L	853.48906	427.24817	285.16787	8
27	2951.56944	1476.28836	984.52800	A	740.40499	370.70613	247.47318	7
28	3065.61237	1533.30982	1022.54231	N	669.36787	335.18757	223.79414	6
29	3178.69644	1589.85186	1060.23700	L	555.32494	278.16611	185.77983	5
30	3235.71791	1618.36259	1079.24415	G	442.24087	221.62407	148.08514	4
31	3336.76559	1668.88643	1112.92671	T	385.21940	193.11334	129.07798	3
32	3473.82450	1737.41589	1158.61302	H	284.17172	142.58950	95.39542	2
33				K	147.11281	74.06004	49.70912	1

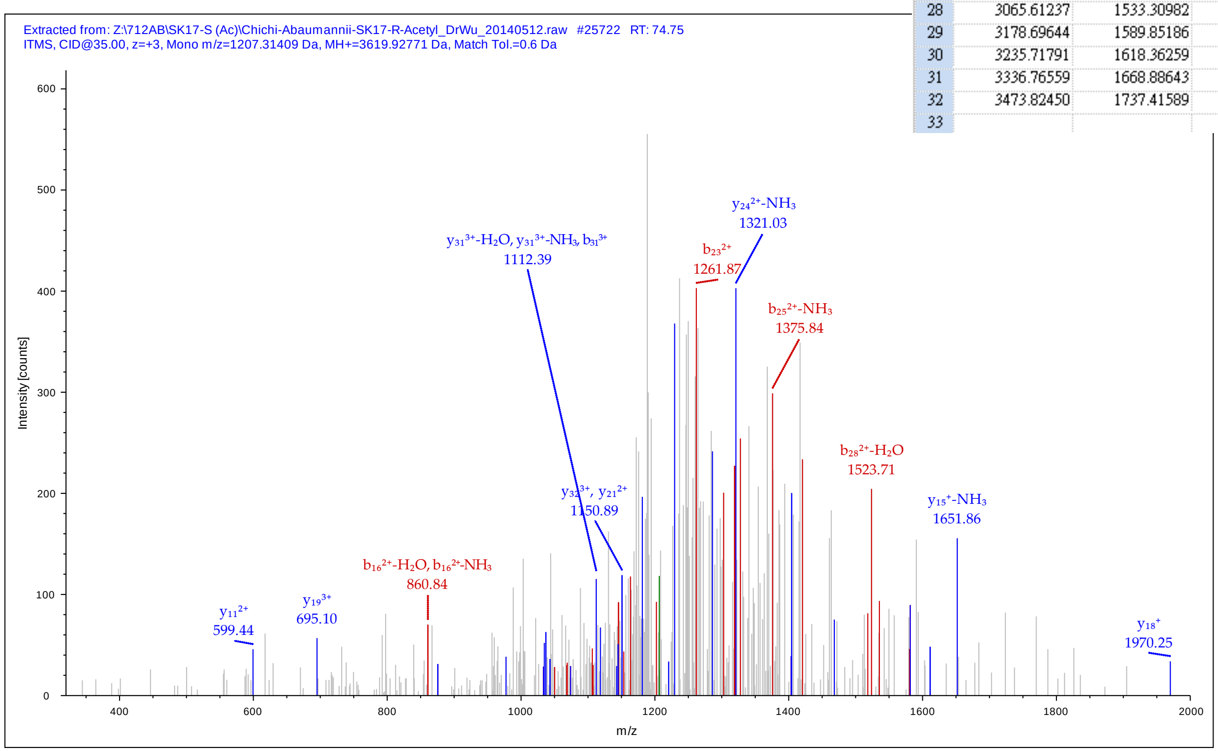

peg.1408 1866

#1	b^{+}	b^{2+}	b^{3+}	Seq.	y^{+}	y^{2+}	y^{3+}	#2
1	171.11281	86.06004	57.70912	K-Acetyl				17
2	272.16049	136.58388	91.39168	T	1638.75029	819.87878	546.92161	16
3	385.24456	193.12592	129.08637	I	1537.70261	769.35494	513.23905	15
4	486.29224	243.64976	162.76893	T	1424.61854	712.81291	475.54436	14
5	633.36066	317.18397	211.79174	F	1323.57086	662.28907	441.86180	13
6	734.40834	367.70781	245.47430	T	1176.50244	588.75486	392.83900	12
7	805.44546	403.22637	269.15334	A	1075.45476	538.23102	359.15644	11
8	902.49823	451.75275	301.50426	P	1004.41764	502.71246	335.47740	10
9	973.53535	487.27131	325.18330	A	907.36487	454.18607	303.12647	9
10	1070.58812	535.79770	357.53422	P	836.32775	418.66751	279.44743	8
11	1173.59731	587.30229	391.87062	C	739.27498	370.14113	247.09651	7
12	1288.62426	644.81577	430.21294	D	636.26579	318.63653	212.76011	6
13	1387.69268	694.34998	463.23574	V	521.23884	261.12306	174.41780	5
14	1490.70187	745.85457	497.57214	C	422.17042	211.58885	141.39499	4
15	1605.72882	803.36805	535.91446	D	319.16123	160.08425	107.05859	3
16	1662.75029	831.87878	554.92161	G	204.13428	102.57078	68.71628	2
17				K	147.11281	74.06004	49.70912	1

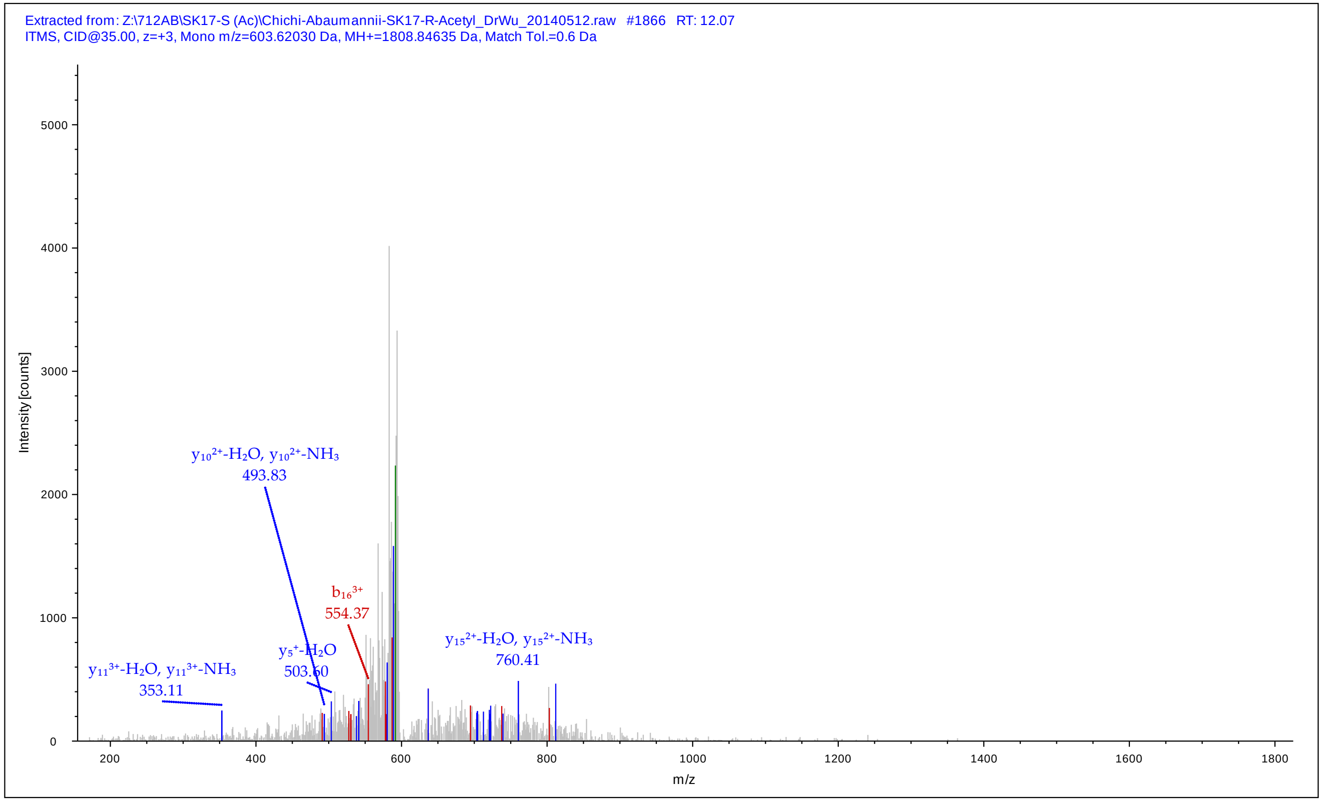

peg.1410 23079

#1	b+	b2+	Seq.	y+	y2+	#2
1	164.07060	82.53894	Y			14
2	279.09755	140.05241	D	1487.74111	744.37419	13
3	410.13805	205.57266	M	1372.71416	686.86072	12
4	580.24358	290.62543	K-Acetyl	1241.67366	621.34047	11
5	695.27053	348.13890	D	1071.56812	536.28770	10
6	794.33895	397.67311	V	956.54117	478.77422	9
7	907.42302	454.21515	L	857.47275	429.24001	8
8	964.44449	482.72588	G	744.38868	372.69798	7
9	1077.52856	539.26792	L	687.36721	344.18724	6
10	1191.57149	596.28938	N	574.28314	287.64521	5
11	1306.59844	653.80286	D	460.24021	230.62374	4
12	1405.66686	703.33707	V	345.21326	173.11027	3
13	1533.72544	767.36636	Q	246.14484	123.57606	2
14			V	118.08626	59.54677	1

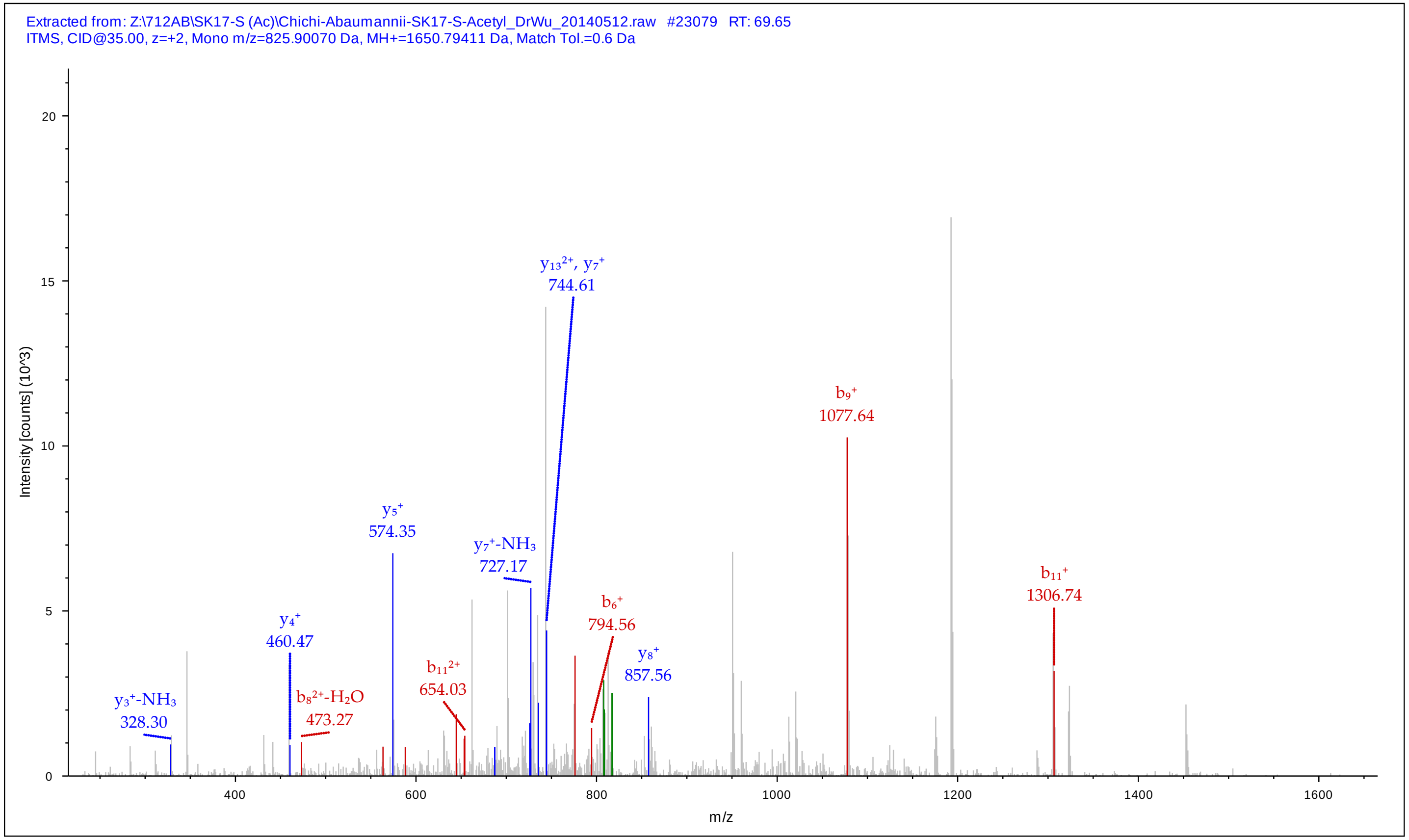

peg.1437 26881

#1	b^+	b^{2+}	Seq.	y^+	y^{2+}	#2
1	100.03931	50.52329	G-Acetyl			23
2	247.07473	124.04100	M-Oxidation	2468.29271	1234.64999	22
3	304.09620	152.55174	G	2321.25730	1161.13229	21
4	401.14897	201.07812	P	2264.23583	1132.62155	20
5	530.19157	265.59942	E	2167.18306	1084.09517	19
6	644.23450	322.62089	N	2038.14046	1019.57387	18
7	745.28218	373.14473	T	1924.09753	962.55240	17
8	858.36625	429.68676	L	1823.04985	912.02856	16
9	971.45032	486.22880	I	1709.96578	855.48653	15
10	1084.53439	542.77083	L	1596.88171	798.94449	14
11	1183.60281	592.30504	V	1483.79764	742.40246	13
12	1298.62976	649.81852	D	1384.72922	692.86825	12
13	1355.65123	678.32925	G	1269.70227	635.35477	11
14	1525.75676	763.38202	K-Acetyl	1212.68080	606.84404	10
15	1622.80953	811.90840	P	1042.57526	521.79127	9
16	1735.89360	868.45044	I	945.52249	473.26488	8
17	1849.93653	925.47190	N	832.43842	416.72285	7
18	1936.96856	968.98792	S	718.39549	359.70138	6
19	2093.06968	1047.03848	R	631.36346	316.18537	5
20	2207.11261	1104.05994	N	475.26234	238.13481	4
21	2294.14464	1147.57596	S	361.21941	181.11334	3
22	2393.21306	1197.11017	V	274.18738	137.59733	2
23			R	175.11896	88.06312	1

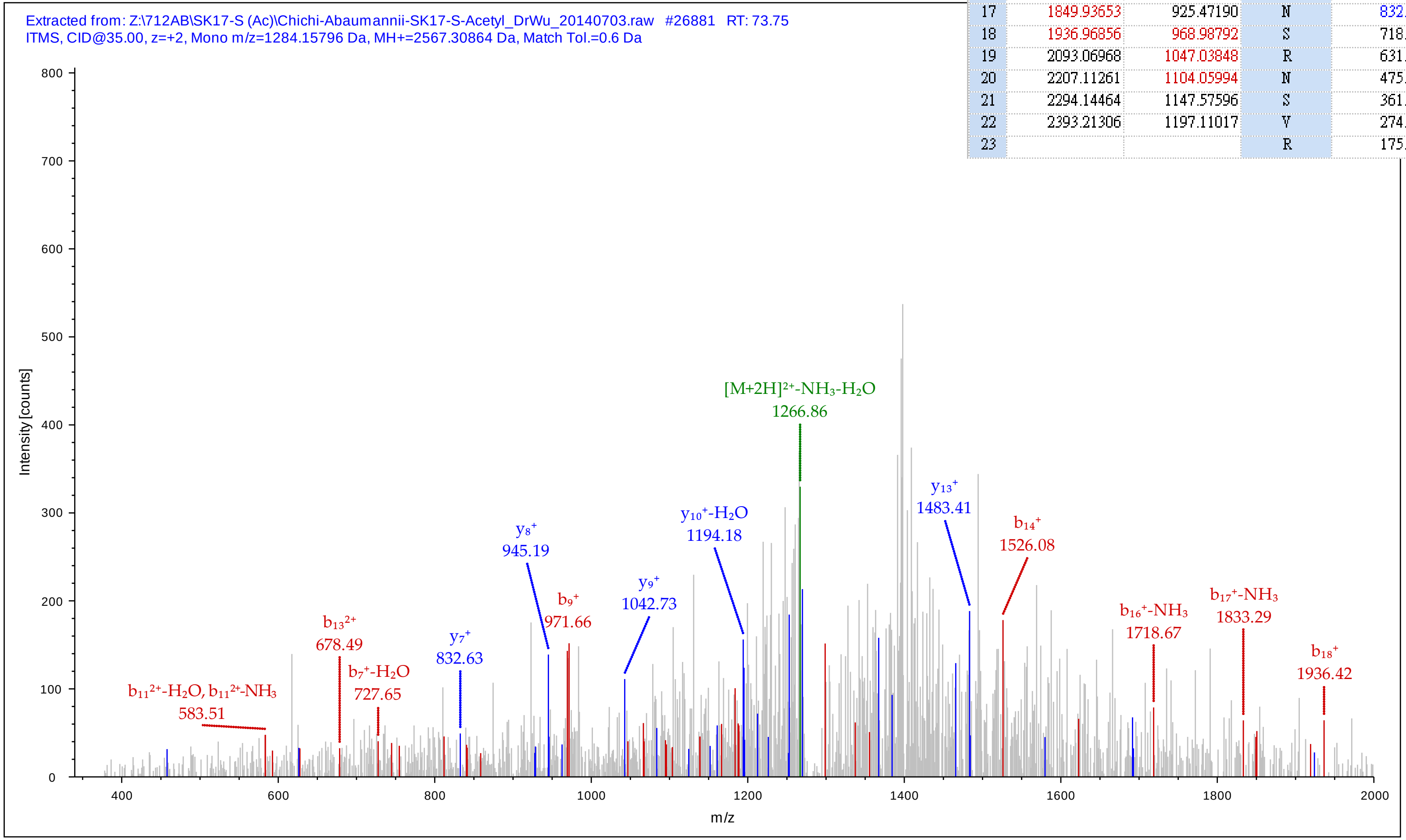

peg.1448   16835

#1	b⁺	b²⁺	b³⁺	Seq.	y⁺	y²⁺	y³⁺	#2
1	88.03931	44.52329	30.01795	S				28
2	201.12338	101.06533	67.71264	I	2804.43139	1402.71933	935.48198	27
3	272.16050	136.58389	91.39168	A	2691.34732	1346.17730	897.78729	26
4	400.25547	200.63137	134.09001	K	2620.31020	1310.65874	874.10825	25
5	497.30824	249.15776	166.44093	P	2492.21523	1246.61125	831.40993	24
6	568.34536	284.67632	190.11997	A	2395.16246	1198.08487	799.05900	23
7	683.37231	342.18979	228.46229	D	2324.12534	1162.56631	775.37996	22
8	798.39926	399.70327	266.80460	D	2209.09839	1105.05283	737.03765	21
9	945.46768	473.23748	315.82741	F	2094.07144	1047.53936	698.69533	20
10	1115.57321	558.29024	372.52925	K-Acetyl	1947.00302	974.00515	649.67252	19
11	1186.61033	593.80880	396.20829	A	1776.89748	888.95238	592.97068	18
12	1299.69440	650.35084	433.90298	I	1705.86036	853.43382	569.29164	17
13	1428.73700	714.87214	476.91718	E	1592.77629	796.89178	531.59695	16
14	1556.79558	778.90143	519.60338	Q	1463.73369	732.37048	488.58275	15
15	1655.86400	828.43564	552.62618	V	1335.67511	668.34119	445.89655	14
16	1712.88547	856.94637	571.63334	G	1236.60669	618.80698	412.87375	13
17	1799.91750	900.46239	600.64402	S	1179.58522	590.29625	393.86659	12
18	1913.00157	957.00442	638.33871	I	1092.55319	546.78023	364.85591	11
19	2000.03360	1000.52044	667.34938	S	979.46912	490.23820	327.16122	10
20	2071.07072	1036.03900	691.02842	A	892.43709	446.72218	298.15055	9
21	2185.11365	1093.06046	729.04273	N	821.39997	411.20362	274.47151	8
22	2272.14568	1136.57648	758.05341	S	707.35704	354.18216	236.45720	7
23	2387.17263	1194.08995	796.39573	D	620.32501	310.66614	207.44652	6
24	2488.22031	1244.61379	830.07829	T	505.29806	253.15267	169.10420	5
25	2589.26799	1295.13763	863.76085	T	404.25038	202.62883	135.42164	4
26	2688.33641	1344.67184	896.78365	V	303.20270	152.10499	101.73908	3
27	2745.35788	1373.18258	915.79081	G	204.13428	102.57078	68.71628	2
28				K	147.11281	74.06004	49.70912	1

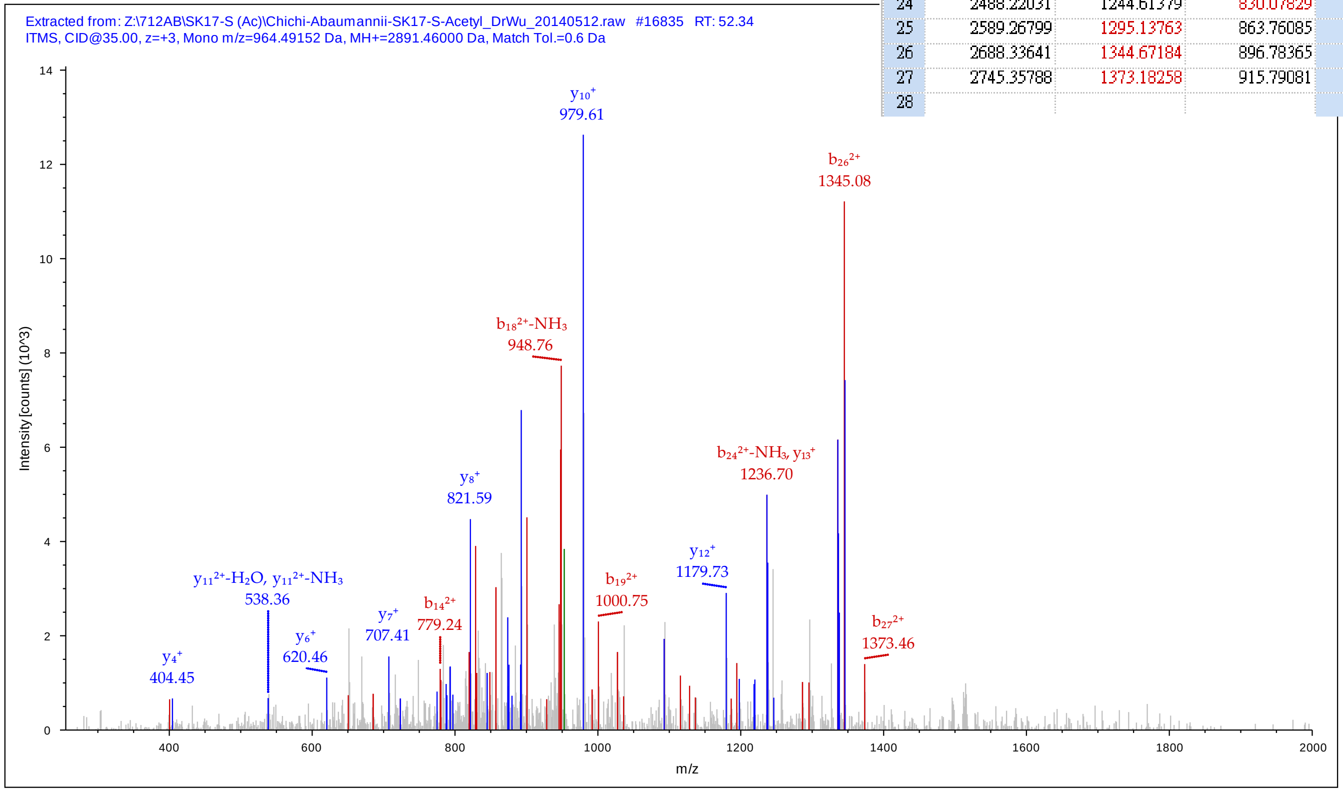

peg.1479 2281

#1	b^+	b^{2+}	b^{3+}	Seq.	y^+	y^{2+}	y^{3+}	#2
1	58.02875	29.51801	20.01443	G				11
2	115.05022	58.02875	39.02159	G	1183.55902	592.28315	395.19119	10
3	252.10913	126.55820	84.70789	H	1126.53755	563.77241	376.18403	9
4	367.13608	184.07168	123.05021	D	989.47864	495.24296	330.49773	8
5	466.20450	233.60589	156.07302	V	874.45169	437.72948	292.15541	7
6	581.23145	291.11936	194.41533	D	775.38327	388.19527	259.13261	6
7	710.27405	355.64066	237.42953	E	660.35632	330.68180	220.79029	5
8	825.30100	413.15414	275.77185	D	531.31372	266.16050	177.77609	4
9	995.40653	498.20690	332.47369	K-Acetyl	416.28677	208.64702	139.43377	3
10	1094.47495	547.74111	365.49650	V	246.18123	123.59425	82.73193	2
11				K	147.11281	74.06004	49.70912	1

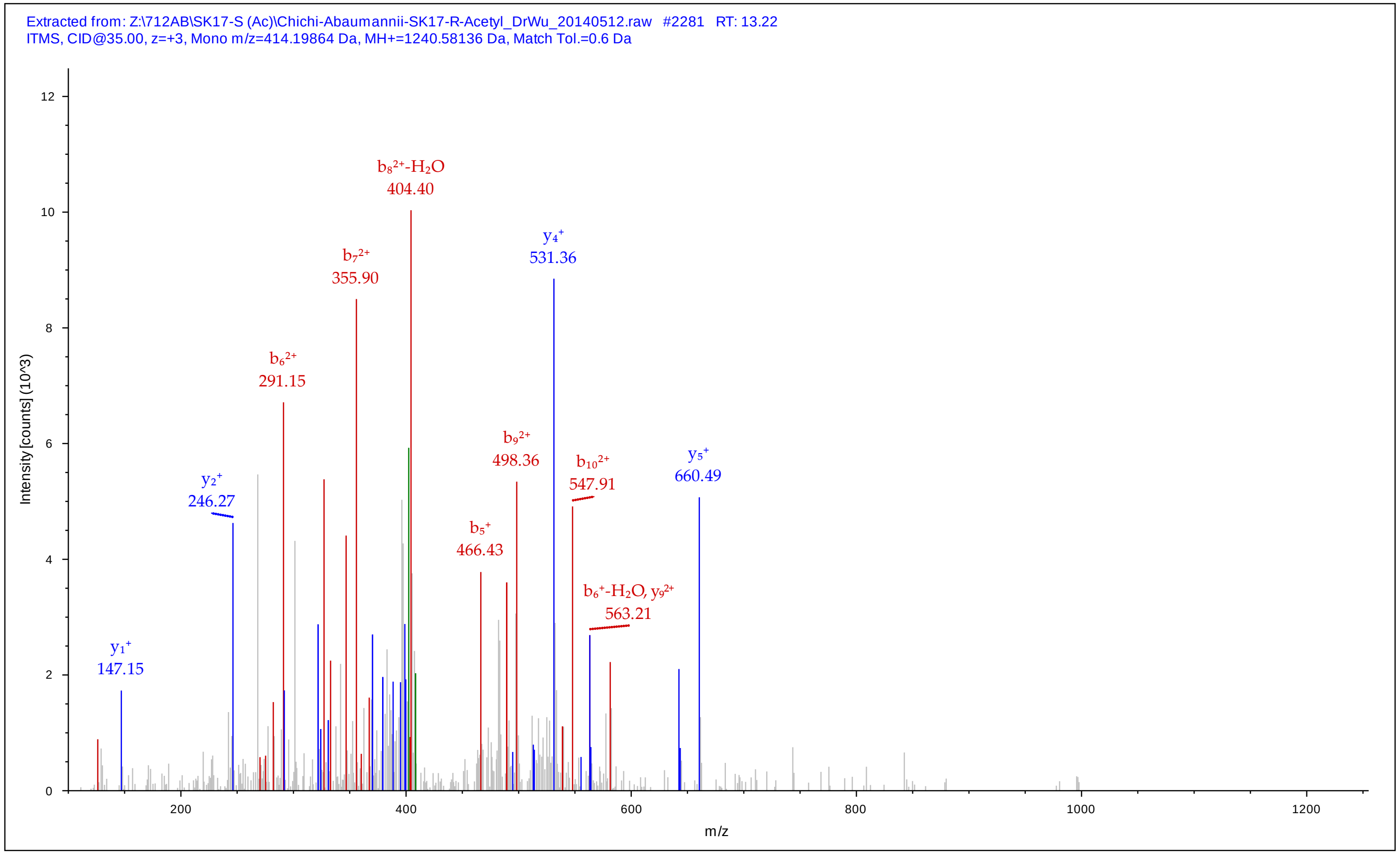

peg.1479 1711

#1	b^+	b^{2+}	b^{3+}	Seq.	y^+	y^{2+}	y^{3+}	#2
1	129.10225	65.05476	43.70560	K				12
2	186.12372	93.56550	62.71276	G	1240.58049	620.79388	414.19835	11
3	243.14519	122.07623	81.71991	G	1183.55902	592.28315	395.19119	10
4	380.20410	190.60569	127.40622	H	1126.53755	563.77241	376.18403	9
5	495.23105	248.11916	165.74853	D	989.47864	495.24296	330.49773	8
6	594.29947	297.65337	198.77134	V	874.45169	437.72948	292.15541	7
7	709.32642	355.16685	237.11366	D	775.38327	388.19527	259.13261	6
8	838.36902	419.68815	280.12786	E	660.35632	330.68180	220.79029	5
9	953.39597	477.20162	318.47017	D	531.31372	266.16050	177.77609	4
10	1123.50150	562.25439	375.17202	K-Acetyl	416.28677	208.64702	139.43377	3
11	1222.56992	611.78860	408.19482	V	246.18123	123.59425	82.73193	2
12				K	147.11281	74.06004	49.70912	1

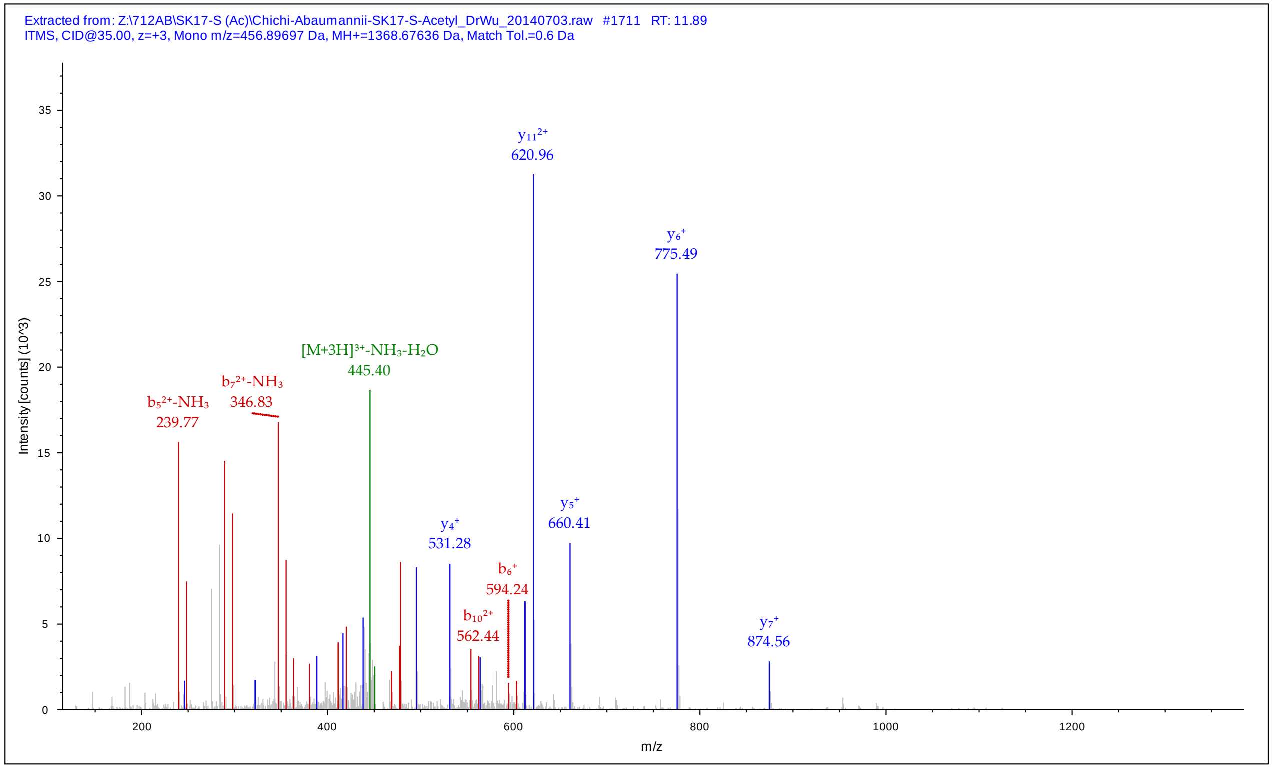

peg.1479 6344

#1	b+	b2+	Seq.	y+	y2+	#2
1	148.04269	74.52498	M-Oxidation			10
2	249.09037	125.04882	T	1118.56884	559.78806	9
3	377.14895	189.07811	Q	1017.52116	509.26422	8
4	547.25449	274.13088	K-Acetyl	889.46258	445.23493	7
5	661.29742	331.15235	N	719.35704	360.18216	6
6	790.34002	395.67365	E	605.31411	303.16069	5
7	903.42409	452.21568	L	476.27151	238.63939	4
8	1018.45104	509.72916	D	363.18744	182.09736	3
9	1119.49872	560.25300	T	248.16049	124.58388	2
10			K	147.11281	74.06004	1

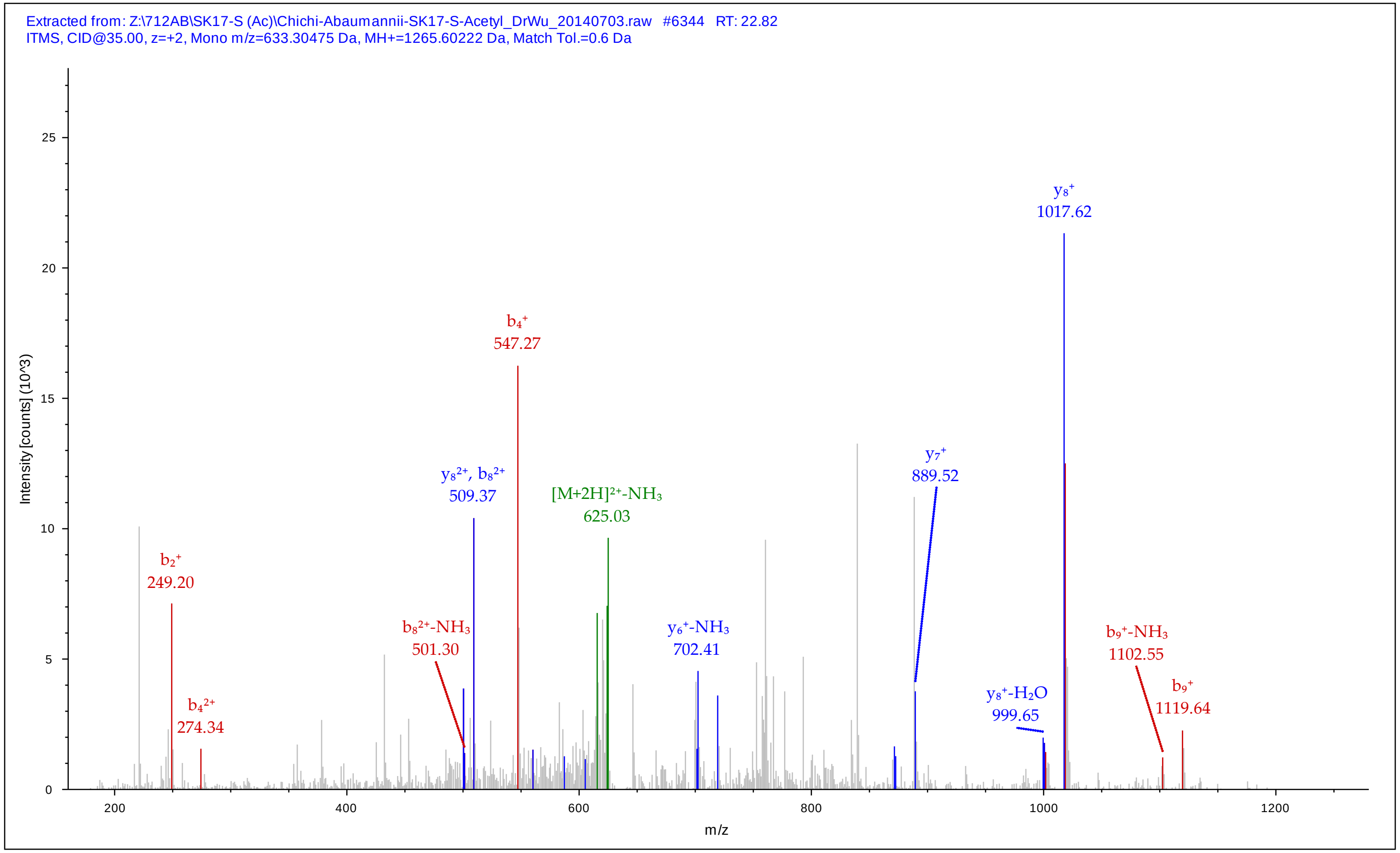

peg.1543 14248

#1	b⁺	b²⁺	Seq.	y⁺	y²⁺	#2
1	148.07570	74.54149	F			12
2	235.10773	118.05750	S	1309.66347	655.33537	11
3	348.19180	174.59954	L	1222.63144	611.81936	10
4	463.21875	232.11301	D	1109.54737	555.27732	9
5	520.24022	260.62375	G	994.52042	497.76385	8
6	690.34575	345.67651	K-Acetyl	937.49895	469.25311	7
7	791.39343	396.20035	T	767.39341	384.20034	6
8	954.45675	477.73201	Y	666.34573	333.67650	5
9	1068.49968	534.75348	N	503.28241	252.14484	4
10	1181.58375	591.29551	L	389.23948	195.12338	3
11	1310.62635	655.81681	E	276.15541	138.58134	2
12			K	147.11281	74.06004	1

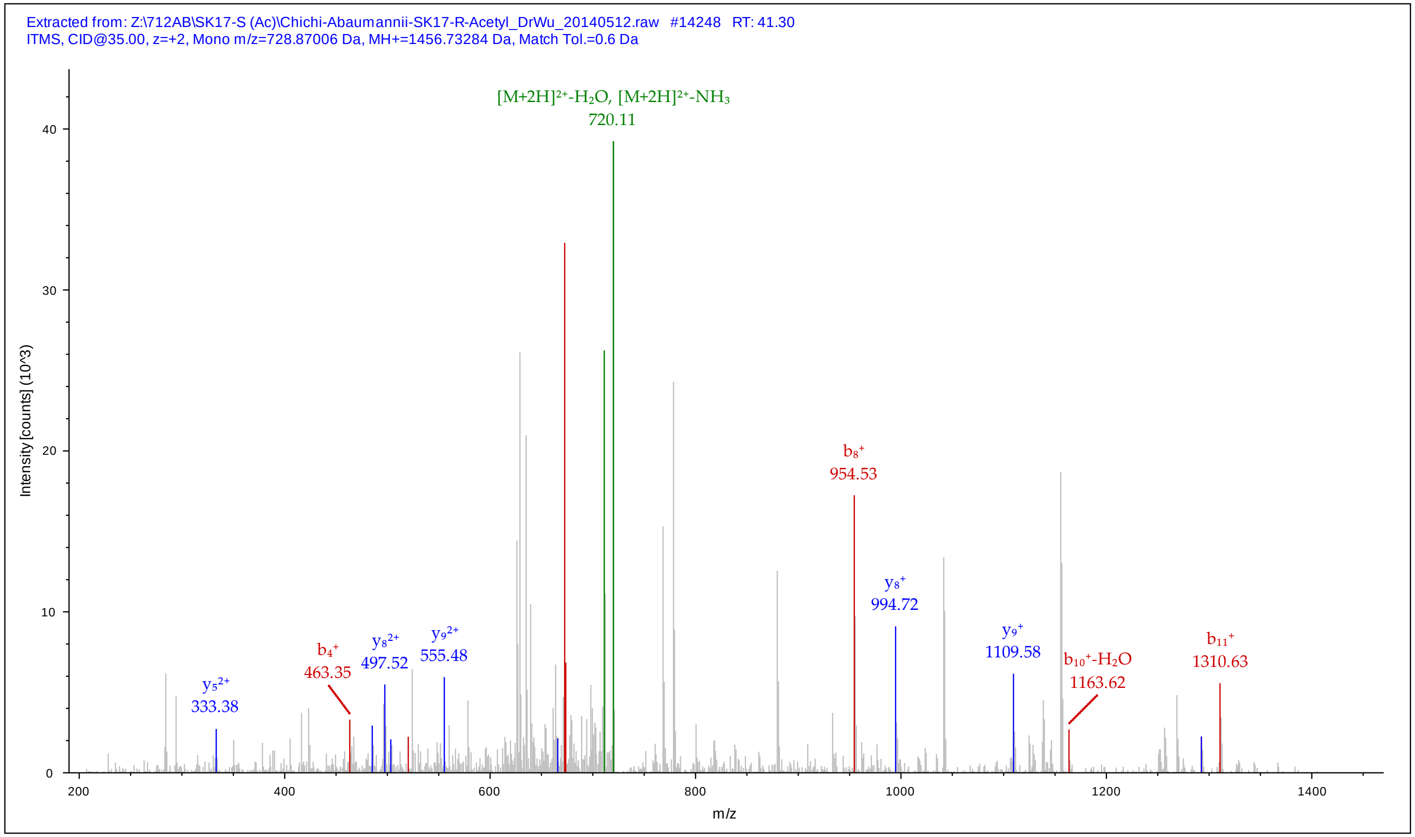

peg.1551 27629

#1	b⁺	b²⁺	b³⁺	Seq.	y⁺	y²⁺	y³⁺	#2
1	172.06044	86.53386	58.02500	E-Acetyl				23
2	342.16598	171.58663	114.72684	K-Acetyl	2620.24481	1310.62604	874.08645	22
3	457.19293	229.10010	153.06916	D	2450.13927	1225.57327	817.38461	21
4	528.23005	264.61866	176.74820	A	2335.11232	1168.05980	779.04229	20
5	657.27265	329.13996	219.76240	E	2264.07520	1132.54124	755.36325	19
6	786.31525	393.66126	262.77660	E	2135.03260	1068.01994	712.34905	18
7	956.42078	478.71403	319.47844	K-Acetyl	2005.99000	1003.49864	669.33485	17
8	1087.46128	544.23428	363.15861	M	1835.88447	918.44587	612.63301	16
9	1215.51986	608.26357	405.84480	Q	1704.84397	852.92562	568.95284	15
10	1286.55698	643.78213	429.52384	A	1576.78539	788.89633	526.26665	14
11	1399.64105	700.32416	467.21853	I	1505.74827	753.37777	502.58761	13
12	1513.68398	757.34563	505.23284	N	1392.66420	696.83574	464.89292	12
13	1612.75240	806.87984	538.25565	V	1278.62127	639.81427	426.87861	11
14	1683.78952	842.39840	561.93469	A	1179.55285	590.28006	393.85580	10
15	1846.85284	923.93006	616.28913	Y	1108.51573	554.76150	370.17676	9
16	1961.87979	981.44353	654.63145	D	945.45241	473.22984	315.82232	8
17	2062.92747	1031.96737	688.31401	T	830.42546	415.71637	277.48000	7
18	2176.01154	1088.50941	726.00870	L	729.37778	365.19253	243.79744	6
19	2263.04357	1132.02542	755.01937	S	616.29371	308.65049	206.10275	5
20	2377.08650	1189.04689	793.03368	N	529.26168	265.13448	177.09208	4
21	2474.13927	1237.57327	825.38461	P	415.21875	208.11301	139.07777	3
22	2603.18187	1302.09457	868.39881	E	318.16598	159.58663	106.72684	2
23				K-Acetyl	189.12338	95.06533	63.71264	1

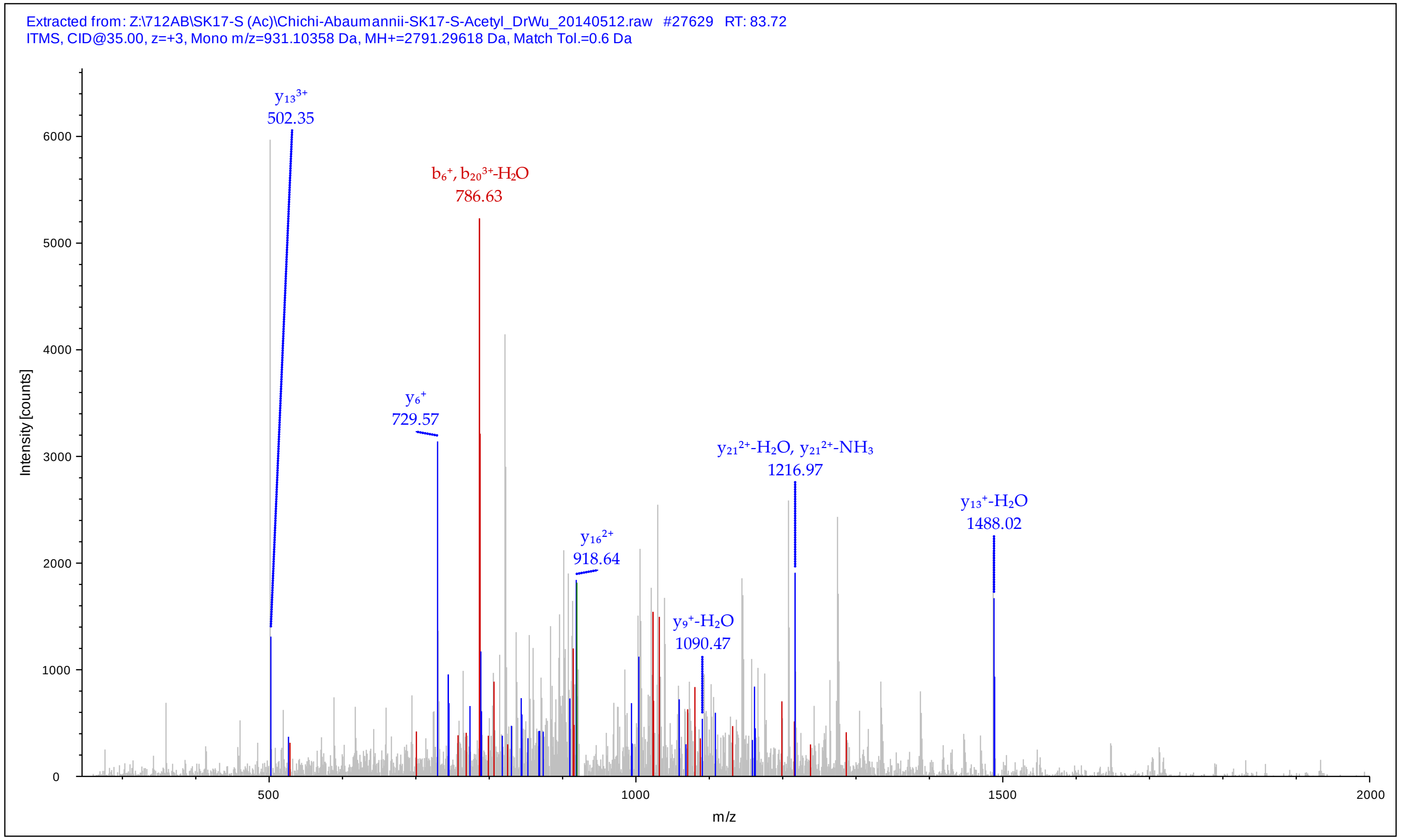

peg.1558 27715

#1	b^{+}	b^{2+}	b^{3+}	b^{4+}	Seq.	y^{+}	y^{2+}	y^{3+}	y^{4+}	#2
1	138.06619	69.53673	46.69358	35.27200	H					28
2	237.13461	119.07094	79.71639	60.03911	V	3121.68683	1561.34705	1041.23379	781.17717	27
3	324.16664	162.58696	108.72706	81.79712	S	3022.61841	1511.81284	1008.21099	756.41006	26
4	381.18811	191.09769	127.73422	96.05248	G	2935.58638	1468.29683	979.20031	734.65205	25
5	482.23579	241.62153	161.41678	121.31440	T	2878.56491	1439.78609	960.19315	720.39669	24
6	595.31986	298.16357	199.11147	149.58542	I	2777.51723	1389.26225	926.51059	695.13477	23
7	742.38828	371.69778	248.13428	186.35253	F	2664.43316	1332.72022	888.81590	666.86375	22
8	898.48940	449.74834	300.16798	225.37781	R	2517.36474	1259.18601	839.79310	630.09664	21
9	1012.53233	506.76980	338.18229	253.88854	N	2361.26362	1181.13545	787.75939	591.07136	20
10	1069.55380	535.28054	357.18945	268.14391	G	2247.22069	1124.11398	749.74508	562.56063	19
11	1140.59092	570.79910	380.86849	285.90319	A	2190.19922	1095.60325	730.73792	548.30526	18
12	1287.65934	644.33331	429.89130	322.67029	F	2119.16210	1060.08469	707.05888	530.54598	17
13	1401.70227	701.35477	467.90561	351.18102	N	1972.09368	986.55048	658.03608	493.77888	16
14	1571.80780	786.40754	524.60745	393.70741	K-Acetyl	1858.05075	929.52901	620.02177	465.26815	15
15	1684.89187	842.94957	562.30214	421.97843	L	1687.94522	844.47625	563.31992	422.74176	14
16	1831.96029	916.48378	611.32495	458.74553	F	1574.86115	787.93421	525.62523	394.47074	13
17	1918.99232	959.99980	640.33562	480.50354	S	1427.79273	714.40000	476.60243	357.70364	12
18	2048.03492	1024.52110	683.34982	512.76419	E	1340.76070	670.88399	447.59175	335.94563	11
19	2208.06558	1104.53643	736.69338	552.77185	C-Carbam···	1211.71810	606.36269	404.57755	303.68498	10
20	2321.14965	1161.07846	774.38807	581.04287	L	1051.68744	526.34736	351.23400	263.67732	9
21	2418.20242	1209.60485	806.73899	605.30606	P	938.60337	469.80532	313.53931	235.40630	8
22	2546.26100	1273.63414	849.42518	637.32071	Q	841.55060	421.27894	281.18838	211.14311	7
23	2659.34507	1330.17617	887.11987	665.59172	L	713.49202	357.24965	238.50219	179.12846	6
24	2772.42914	1386.71821	924.81456	693.86274	L	600.40795	300.70761	200.80750	150.85745	5
25	2900.48772	1450.74750	967.50076	725.87739	Q	487.32388	244.16558	163.11281	122.58643	4
26	2999.55614	1500.28171	1000.52356	750.64449	V	359.26530	180.13629	120.42662	90.57178	3
27	3112.64021	1556.82374	1038.21825	778.91551	L	260.19688	130.60208	87.40381	65.80468	2
28					K	147.11281	74.06004	49.70912	37.53366	1

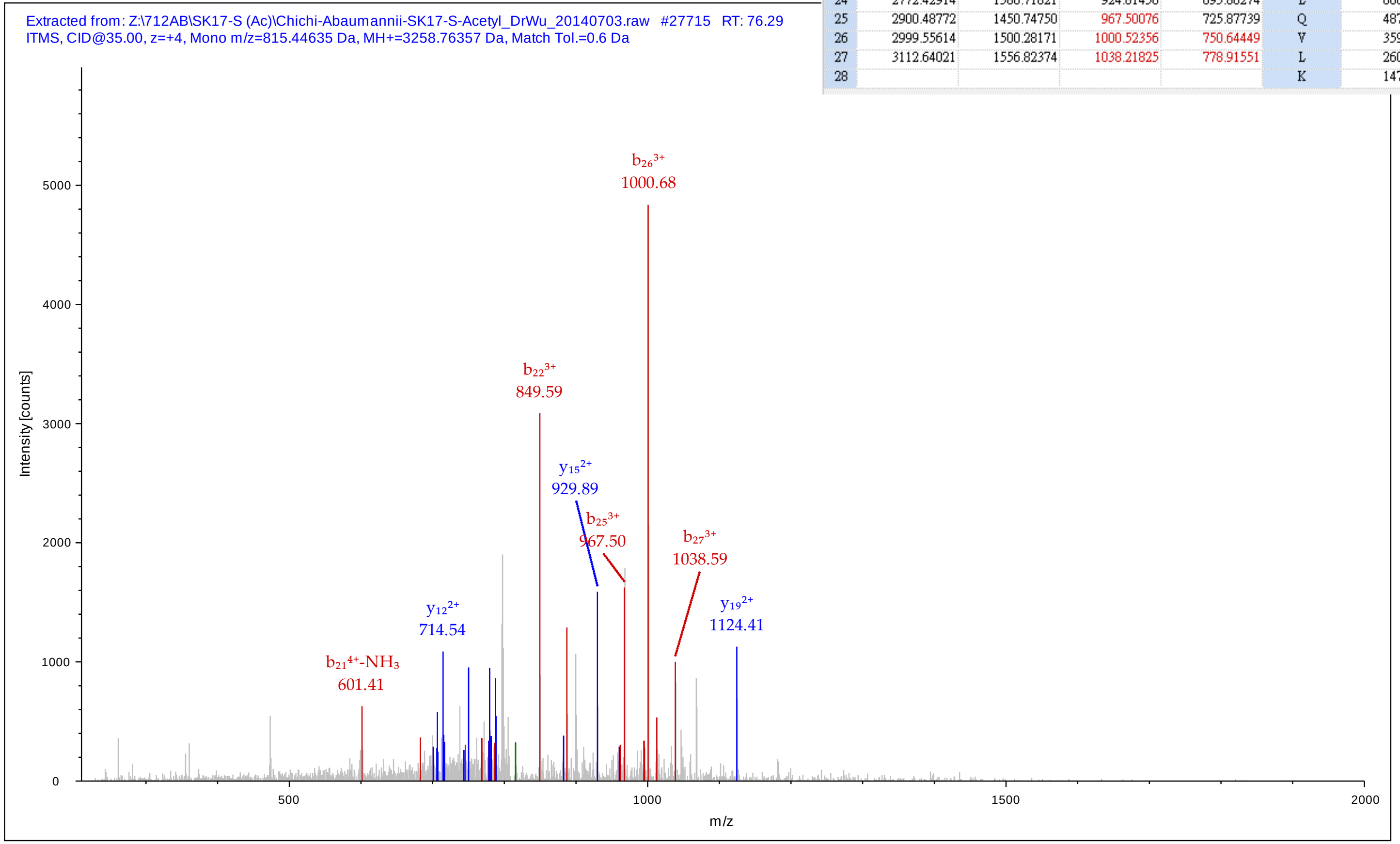

peg.1615 27340

peg.2943 27340

#1	b⁺	b²⁺	Seq.	y⁺	y²⁺	#2
1	58.02875	29.51801	G			18
2	172.07168	86.53948	N	2120.04078	1060.52403	17
3	229.09315	115.05021	G	2005.99785	1003.50256	16
4	392.15647	196.58187	Y	1948.97638	974.99183	15
5	520.25144	260.62936	K	1785.91306	893.46017	14
6	619.31986	310.16357	V	1657.81809	829.41268	13
7	734.34681	367.67704	D	1558.74967	779.87847	12
8	897.41013	449.20870	Y	1443.72272	722.36500	11
9	1067.51566	534.26147	K-Acetyl	1280.65940	640.83334	10
10	1182.54261	591.77494	D	1110.55387	555.78057	9
11	1296.58554	648.79641	N	995.52692	498.26710	8
12	1410.62847	705.81787	N	881.48399	441.24563	7
13	1523.71254	762.35991	L	767.44106	384.22417	6
14	1620.76531	810.88629	P	654.35699	327.68213	5
15	1790.87085	895.93906	K-Acetyl	557.30422	279.15575	4
16	1887.92362	944.46545	P	387.19868	194.10298	3
17	2002.95057	1001.97892	D	290.14591	145.57659	2
18			R	175.11896	88.06312	1

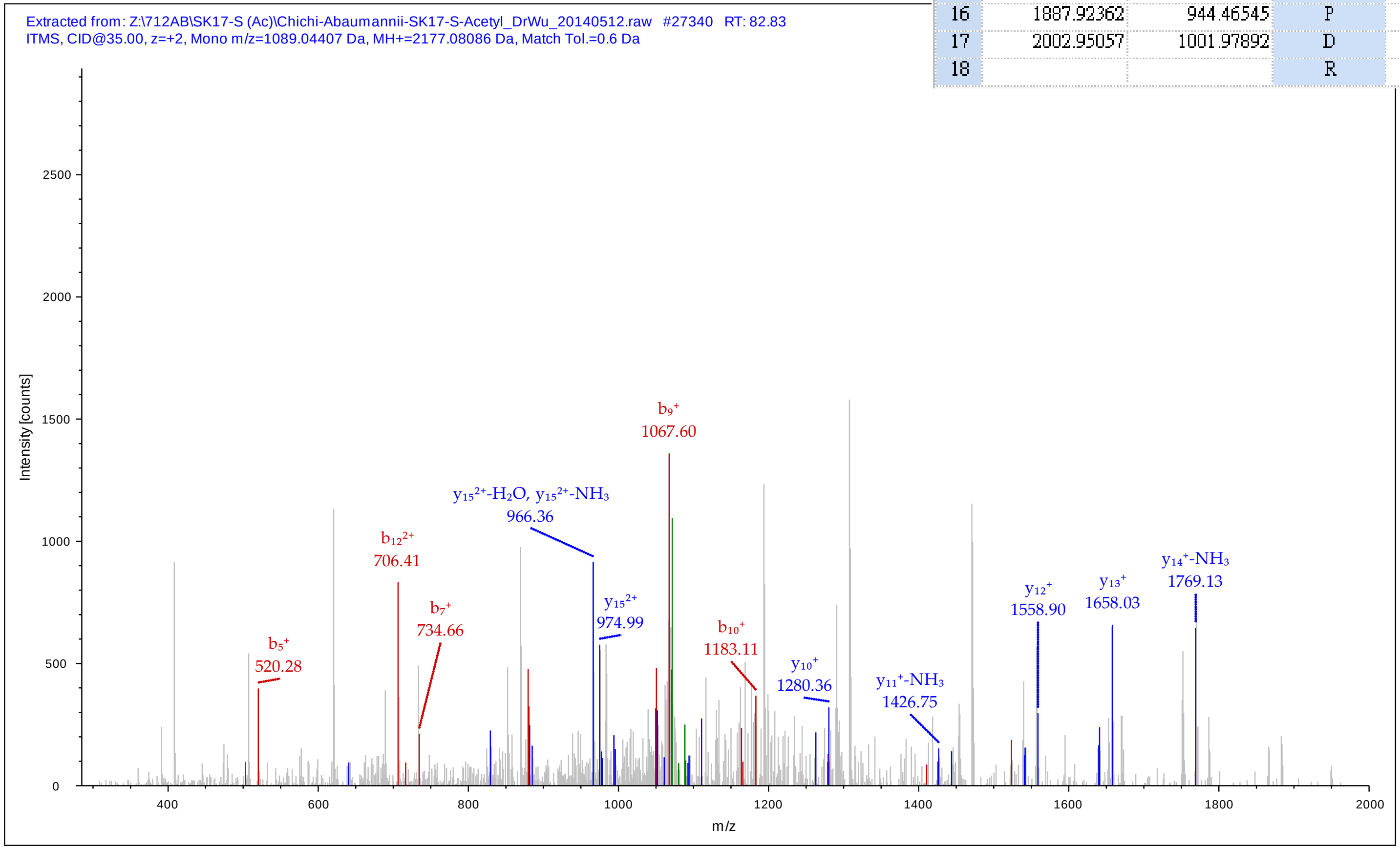

peg.1646 19843

#1	b^+	b^{2+}	b^{3+}	b^{4+}	Seq.	y^+	y^{2+}	y^{3+}	y^{4+}	#2
1	130.04987	65.52857	44.02147	33.26793	S-Acetyl					25
2	243.13394	122.07061	81.71616	61.53894	I	2592.26540	1296.63634	864.75998	648.82181	24
3	314.17106	157.58917	105.39520	79.29822	A	2479.18133	1240.09430	827.06529	620.55079	23
4	385.20818	193.10773	129.07424	97.05750	A	2408.14421	1204.57574	803.38625	602.79151	22
5	532.24360	266.62544	178.08605	133.81636	M-Oxidation	2337.10709	1169.05718	779.70721	585.03223	21
6	631.31202	316.15965	211.10886	158.58346	V	2190.07168	1095.53948	730.69541	548.27338	20
7	745.35495	373.18111	249.12317	187.09419	N	2091.00326	1046.00527	697.67260	523.50627	19
8	802.37642	401.69185	268.13032	201.34956	G	1976.96033	988.98380	659.65829	494.99554	18
9	933.41692	467.21210	311.81049	234.10969	M	1919.93886	960.47307	640.65114	480.74017	17
10	1036.42611	518.71669	346.14689	259.86198	C	1788.89836	894.95282	596.97097	447.98005	16
11	1164.52108	582.76418	388.84521	291.88573	K	1685.88917	843.44822	562.63457	422.22775	15
12	1292.57966	646.79347	431.53140	323.90037	Q	1557.79420	779.40074	519.93625	390.20401	14
13	1395.58885	698.29806	465.86780	349.65267	C	1429.73562	715.37145	477.25006	358.18936	13
14	1452.61032	726.80880	484.87496	363.90804	G	1326.72643	663.86685	442.91366	332.43706	12
15	1622.71585	811.86156	541.57680	406.43442	K-Acetyl	1269.70496	635.35612	423.90650	318.18170	11
16	1751.75845	876.38286	584.59100	438.69507	E	1099.59942	550.30335	367.20466	275.65531	10
17	1848.81122	924.90925	616.94192	462.95826	P	970.55682	485.78205	324.19046	243.39466	9
18	1961.89529	981.45128	654.63661	491.22928	L	873.50405	437.25566	291.83953	219.13147	8
19	2089.95387	1045.48057	697.32281	523.24393	Q	760.41998	380.71363	254.14484	190.86045	7
20	2160.99099	1080.99913	721.00185	541.00321	A	632.36140	316.68434	211.45865	158.84581	6
21	2274.07506	1137.54117	758.69654	569.27422	I	561.32428	281.16578	187.77961	141.08653	5
22	2373.14348	1187.07538	791.71934	594.04133	V	448.24021	224.62374	150.08492	112.81551	4
23	2460.17551	1230.59139	820.73002	615.79934	S	349.17179	175.08953	117.06211	88.04841	3
24	2575.20246	1288.10487	859.07234	644.55607	D	262.13976	131.57352	88.05144	66.29040	2
25					K	147.11281	74.06004	49.70912	37.53366	1

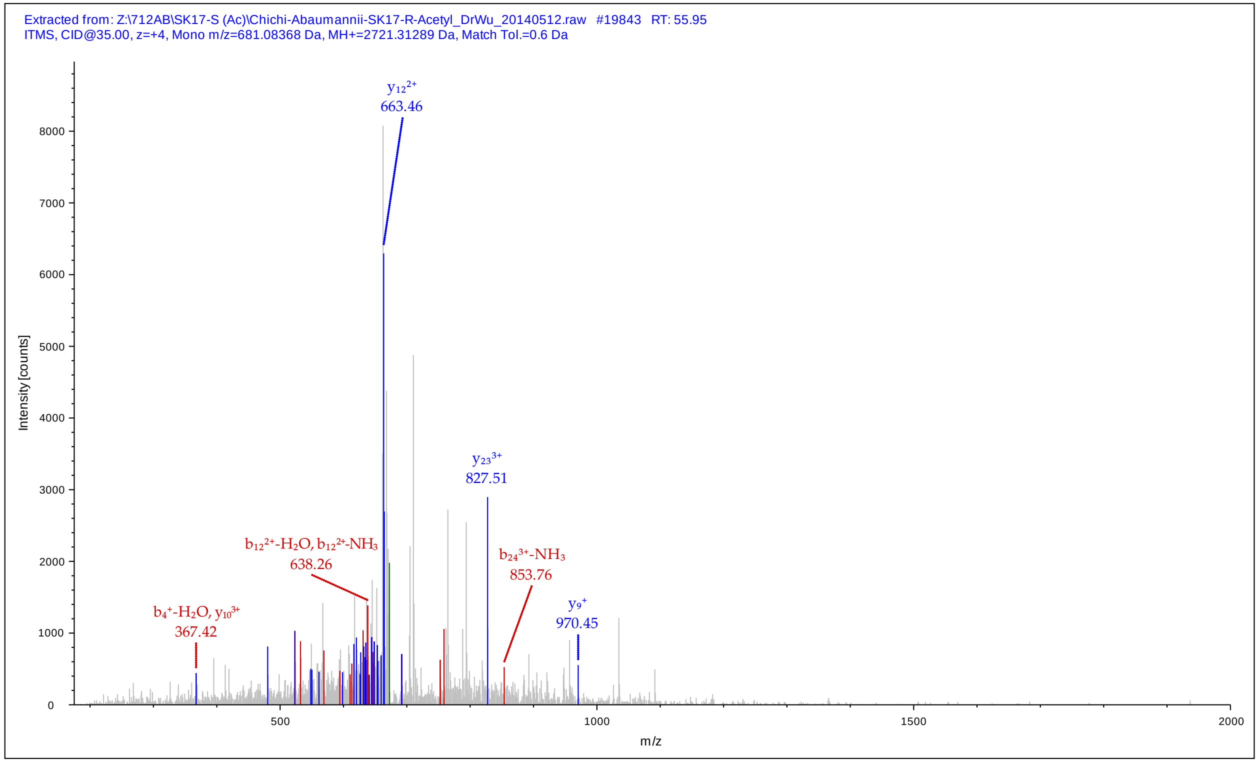

peg.1707 9382

#1	b⁺	b²⁺	Seq.	y⁺	y²⁺	#2
1	172.06044	86.53386	E-Acetyl			11
2	342.16598	171.58663	K-Acetyl	1147.61404	574.31066	10
3	489.20139	245.10433	M-Oxidation	977.50851	489.25789	9
4	560.23851	280.62289	A	830.47309	415.74018	8
5	631.27563	316.14145	A	759.43597	380.22162	7
6	718.30766	359.65747	S	688.39885	344.70306	6
7	831.39173	416.19950	L	601.36682	301.18705	5
8	960.43433	480.72080	E	488.28275	244.64501	4
9	1031.47145	516.23936	A	359.24015	180.12371	3
10	1144.55552	572.78140	L	288.20303	144.60515	2
11			R	175.11896	88.06312	1

Extracted from: Z:\712AB\SK17-S (Ac)\Chichi-Abaumannii-SK17-S-Acetyl_DrWu_20140703.raw #9382 RT: 29.85
ITMS, CID@35.00, z=+2, Mono m/z=659.83527 Da, MH+=1318.66326 Da, Match Tol.=0.6 Da

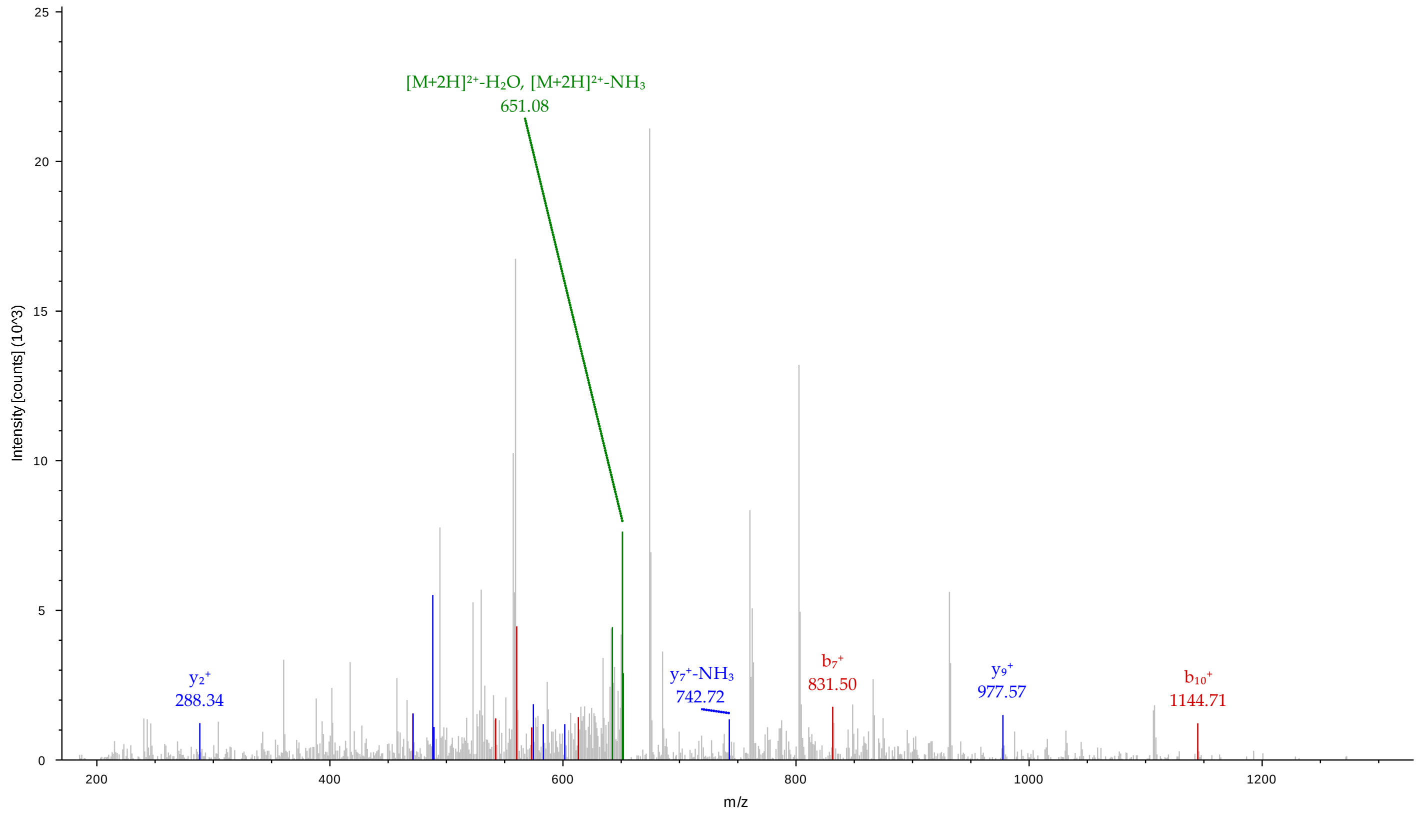

peg.1712 13076

#1	b⁺	b²⁺	Seq.	y⁺	y²⁺	#2
1	130.04988	65.52858	E			11
2	300.15541	150.58134	K-Acetyl	1318.62342	659.81535	10
3	429.19801	215.10264	E	1148.51788	574.76258	9
4	542.28208	271.64468	I	1019.47528	510.24128	8
5	629.31411	315.16069	S	906.39121	453.69924	7
6	758.35671	379.68199	E	819.35918	410.18323	6
7	873.38366	437.19547	D	690.31658	345.66193	5
8	988.41061	494.70894	D	575.28963	288.14845	4
9	1117.45321	559.23024	E	460.26268	230.63498	3
10	1273.55433	637.28080	R	331.22008	166.11368	2
11			R	175.11896	88.06312	1

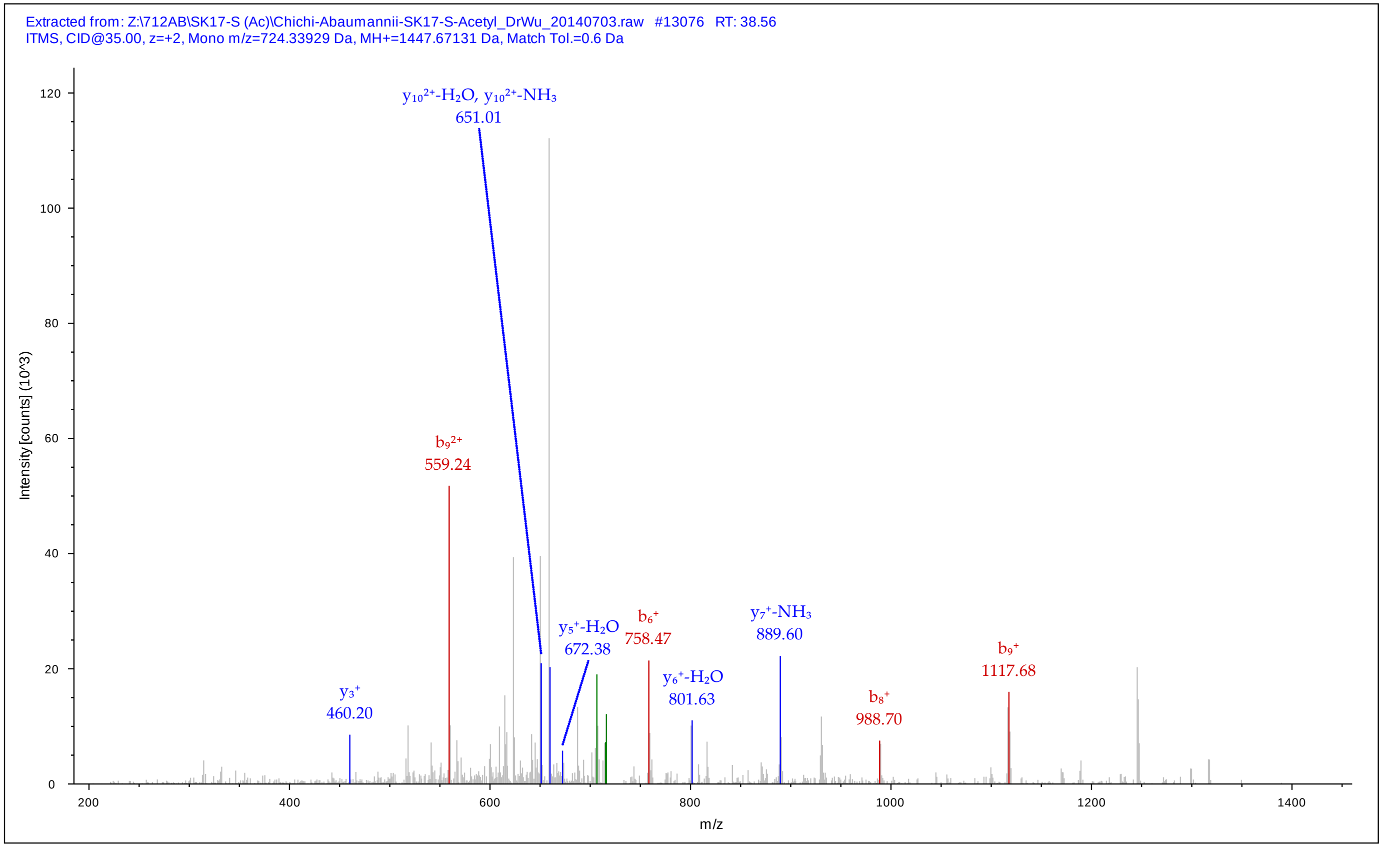

peg.1713 23766

#1	b^{+}	b^{2+}	b^{3+}	Seq.	y^{+}	y^{2+}	y^{3+}	#2
1	156.10191	78.55459	52.70549	L-Acetyl				38
2	213.12338	107.06533	71.71264	G	4119.02063	2060.01395	1373.67839	37
3	312.19180	156.59954	104.73545	V	4061.99916	2031.50322	1354.67124	36
4	459.22722	230.11725	153.74726	M-Oxidation	3962.93074	1981.96901	1321.64843	35
5	574.25417	287.63072	192.08957	D	3815.89532	1908.45130	1272.63662	34
6	687.33824	344.17276	229.78426	L	3700.86837	1850.93782	1234.29431	33
7	788.38592	394.69660	263.46682	T	3587.78430	1794.39579	1196.59962	32
8	859.42304	430.21516	287.14586	A	3486.73662	1743.87195	1162.91706	31
9	972.50711	486.75719	324.84055	I	3415.69950	1708.35339	1139.23802	30
10	1075.51630	538.26179	359.17695	C	3302.61543	1651.81135	1101.54333	29
11	1188.60037	594.80382	396.87164	L	3199.60624	1600.30676	1067.20693	28
12	1348.63102	674.81915	450.21519	C-Carbam…	3086.52217	1543.76472	1029.51224	27
13	1504.73214	752.86971	502.24890	R	2926.49152	1463.74940	976.16869	26
14	1619.75909	810.38318	540.59121	D	2770.39040	1385.69884	924.13498	25
15	1756.81800	878.91264	586.27752	H	2655.36345	1328.18536	885.79267	24
16	1870.86093	935.93410	624.29183	N	2518.30454	1259.65591	840.10636	23
17	1969.92935	985.46831	657.31463	V	2404.26161	1202.63444	802.09205	22
18	2066.98212	1033.99470	689.66556	P	2305.19319	1153.10023	769.06925	21
19	2180.06619	1090.53673	727.36025	L	2208.14042	1104.57385	736.71832	20
20	2308.12477	1154.56602	770.04644	Q	2095.05635	1048.03181	699.02363	19
21	2407.19319	1204.10023	803.06925	V	1966.99777	984.00252	656.33744	18
22	2554.26161	1277.63444	852.09205	F	1867.92935	934.46831	623.31463	17
23	2669.28856	1335.14792	890.43437	D	1720.86093	860.93410	574.29183	16
24	2800.32906	1400.66817	934.11454	M	1605.83398	803.42063	535.94951	15
25	2914.37199	1457.68963	972.12885	N	1474.79348	737.90038	492.26934	14
26	3084.47753	1542.74240	1028.83069	K-Acetyl	1360.75055	680.87891	454.25503	13
27	3171.50956	1586.25842	1057.84137	S	1190.64501	595.82614	397.55319	12
28	3228.53103	1614.76915	1076.84853	G	1103.61298	552.31013	368.54251	11
29	3299.56815	1650.28771	1100.52757	A	1046.59151	523.79939	349.53535	10
30	3412.65222	1706.82975	1138.22226	L	975.55439	488.28083	325.85631	9
31	3525.73629	1763.37178	1175.91695	L	862.47032	431.73880	288.16162	8
32	3612.76832	1806.88780	1204.92762	S	749.38625	375.19676	250.46693	7
33	3711.83674	1856.42201	1237.95043	V	662.35422	331.68075	221.45626	6
34	3810.90516	1905.95622	1270.97324	V	563.28580	282.14654	188.43345	5
35	3941.94566	1971.47647	1314.65340	M	464.21738	232.61233	155.41064	4
36	3998.96713	1999.98720	1333.66056	G	333.17688	167.09208	111.73048	3
37	4128.00973	2064.50850	1376.67476	E	276.15541	138.58134	92.72332	2
38				K	147.11281	74.06004	49.70912	1

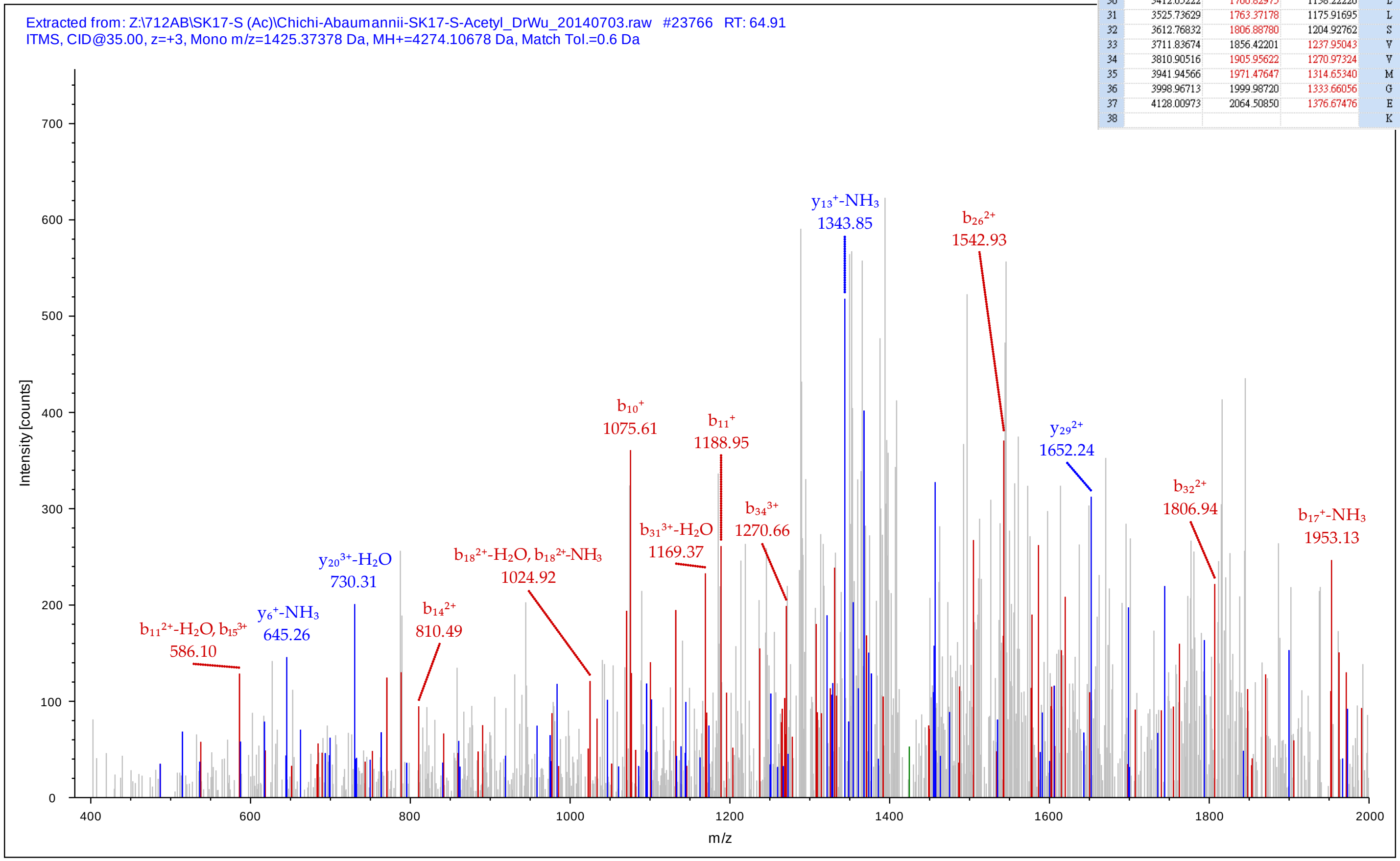

peg.1714   12465

#1	b+	b2+	Seq.	y+	y2+	#2
1	114.09135	57.54931	L			5
2	227.17542	114.09135	I	543.38649	272.19688	4
3	397.28095	199.14411	K-Acetyl	430.30242	215.65485	3
4	510.36502	255.68615	I	260.19688	130.60208	2
5			K	147.11281	74.06004	1

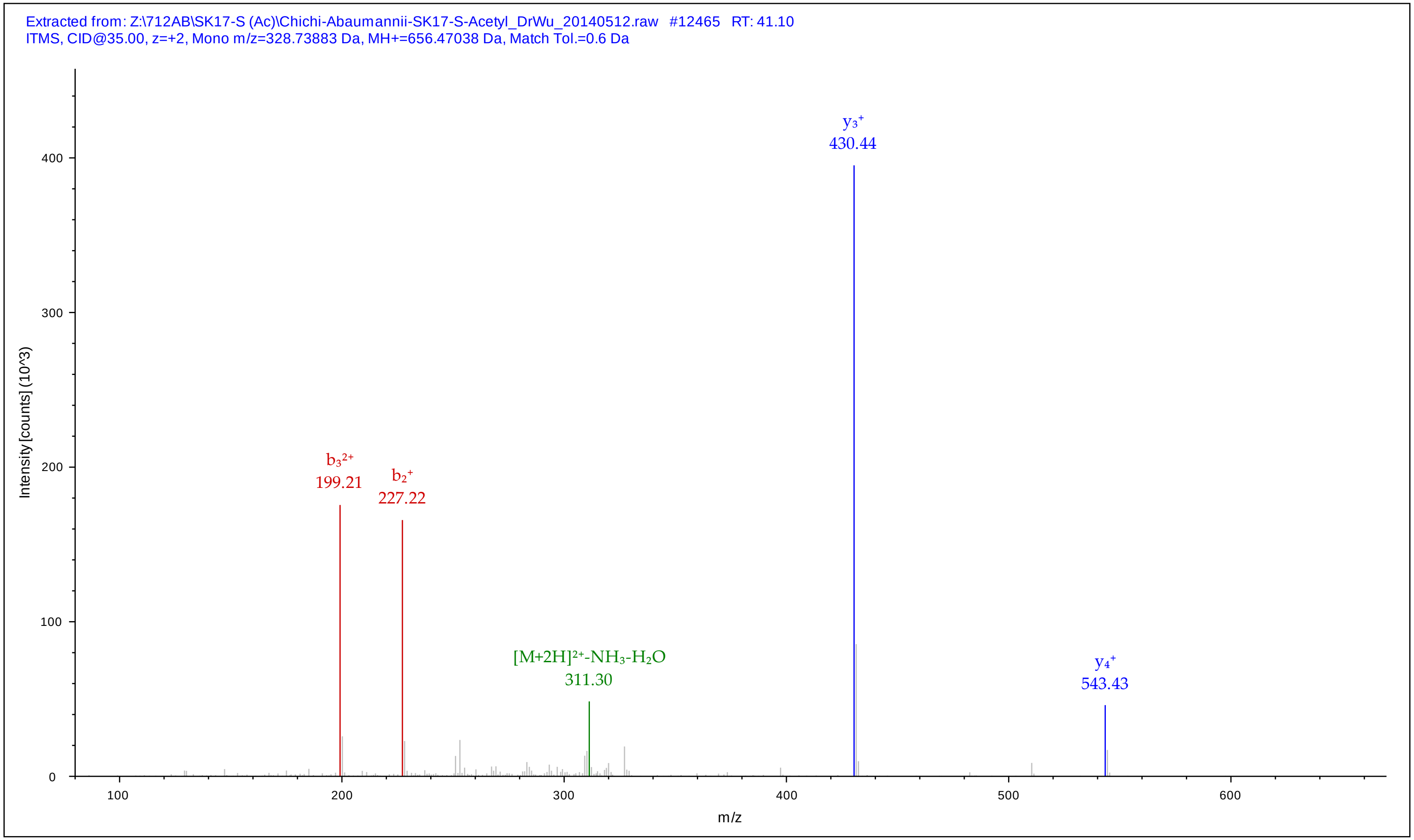

peg.1733   19215

#1	b+	b2+	Seq.	y+	y2+	#2
1	130.04987	65.52857	S-Acetyl			19
2	227.10264	114.05496	P	1762.84633	881.92680	18
3	330.11183	165.55955	C	1665.79356	833.40042	17
4	387.13330	194.07029	G	1562.78437	781.89582	16
5	488.18098	244.59413	T	1505.76290	753.38509	15
6	545.20245	273.10486	G	1404.71522	702.86125	14
7	646.25013	323.62870	T	1347.69375	674.35051	13
8	733.28216	367.14472	S	1246.64607	623.82667	12
9	804.31928	402.66328	A	1159.61404	580.31066	11
10	974.42482	487.71605	K-Acetyl	1088.57692	544.79210	10
11	1087.50889	544.25808	I	918.47139	459.73933	9
12	1158.54601	579.77664	A	805.38732	403.19730	8
13	1318.57666	659.79197	C-Carbam···	734.35020	367.67874	7
14	1431.66073	716.33400	L	574.31954	287.66341	6
15	1502.69785	751.85256	A	461.23547	231.12137	5
16	1573.73497	787.37112	A	390.19835	195.60281	4
17	1688.76192	844.88460	D	319.16123	160.08425	3
18	1745.78339	873.39533	G	204.13428	102.57078	2
19			K	147.11281	74.06004	1

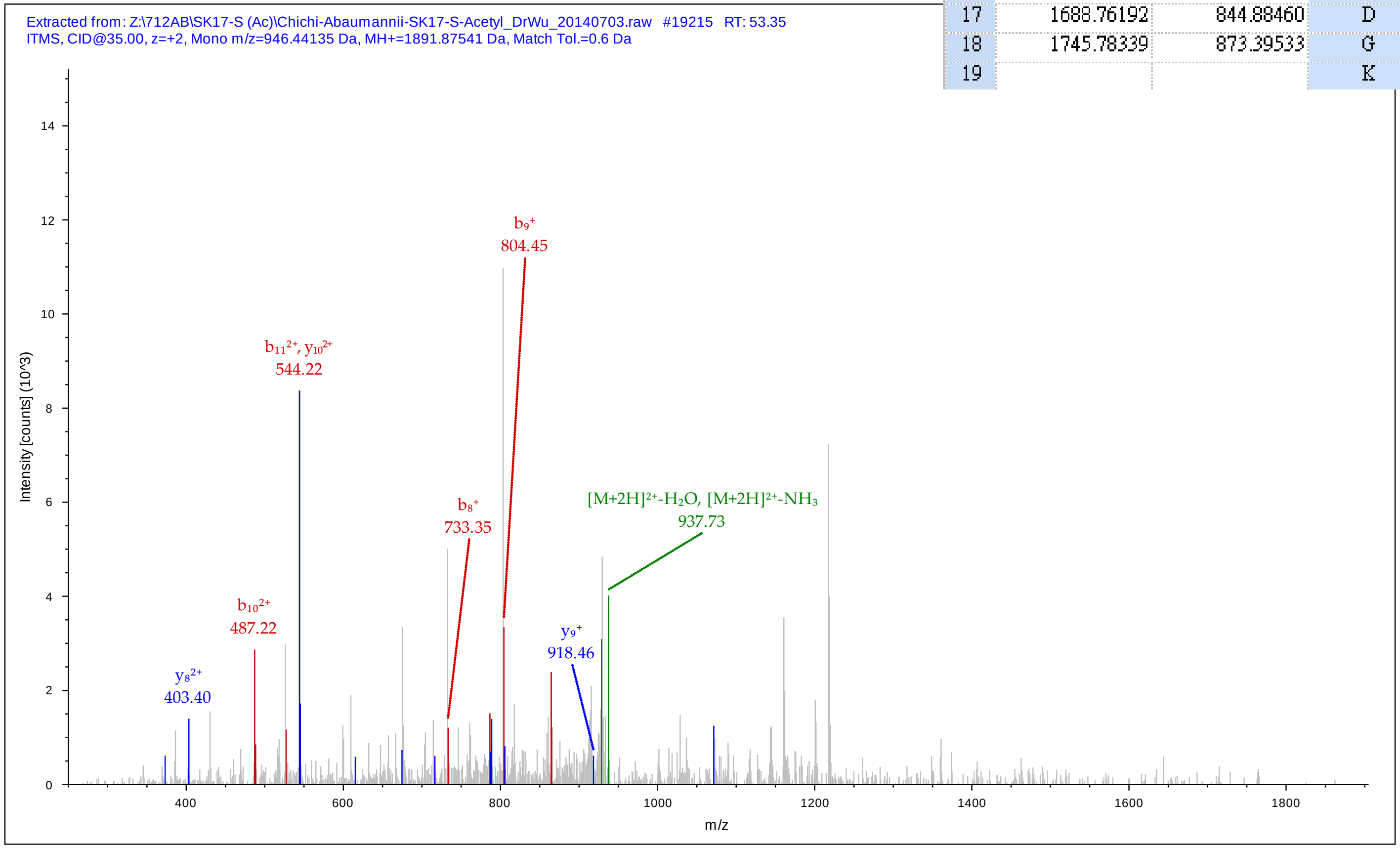

peg.1734 24326

#1	b⁺	b²⁺	Seq.	y⁺	y²⁺	#2
1	129.06586	65.03657	Q			16
2	244.09281	122.55004	D	1771.01134	886.00931	15
3	373.13541	187.07134	E	1655.98439	828.49583	14
4	486.21948	243.61338	I	1526.94179	763.97453	13
5	557.25660	279.13194	A	1413.85772	707.43250	12
6	727.36213	364.18470	K-Acetyl	1342.82060	671.91394	11
7	840.44620	420.72674	L	1172.71506	586.86117	10
8	987.51462	494.26095	F	1059.63099	530.31913	9
9	1101.55755	551.28241	N	912.56257	456.78492	8
10	1200.62597	600.81662	Y	798.51964	399.76346	7
11	1287.65800	644.33264	S	699.45122	350.22925	6
12	1415.75297	708.38012	K	612.41919	306.71323	5
13	1528.83704	764.92216	I	484.32422	242.66575	4
14	1625.88981	813.44854	P	371.24015	186.12371	3
15	1724.95823	862.98275	Y	274.18738	137.59733	2
16			R	175.11896	88.06312	1

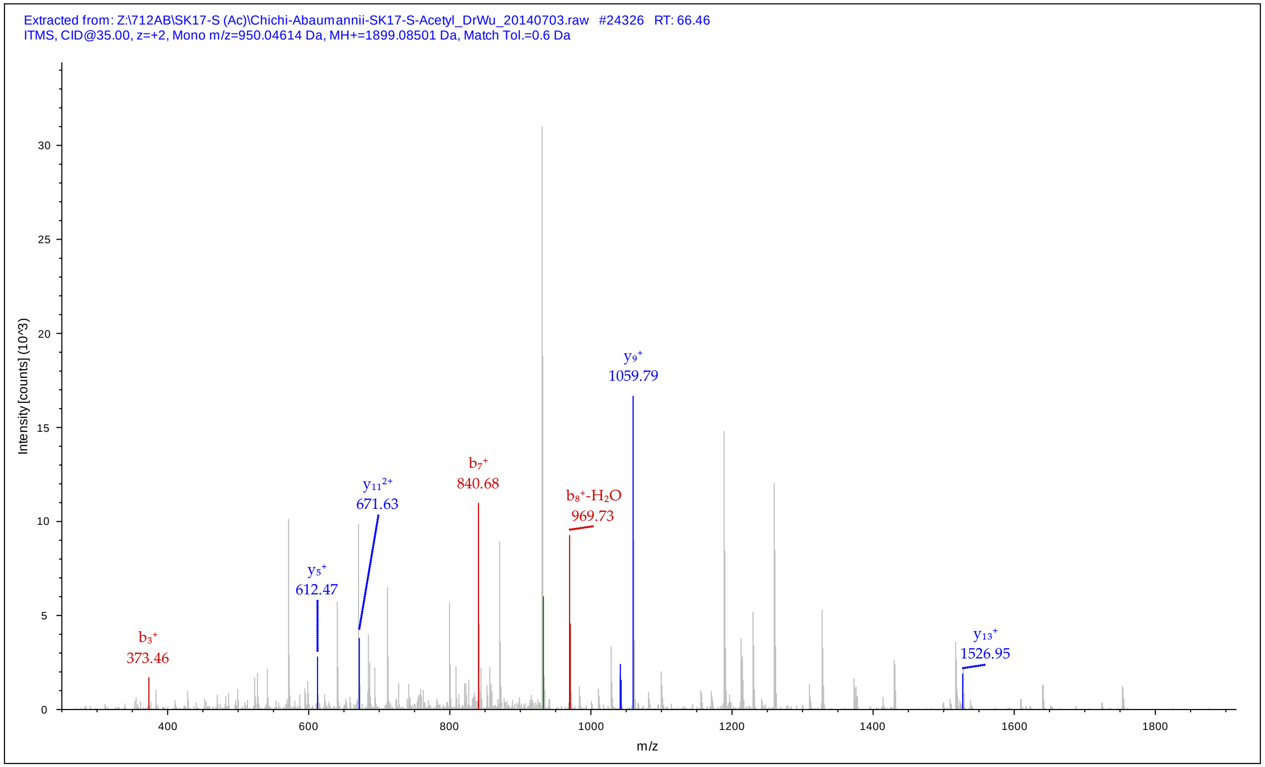

peg.1776 13276

#1	b⁺	b²⁺	Seq.	y⁺	y²⁺	#2
1	130.04987	65.52857	S-Acetyl			14
2	300.15541	150.58134	K-Acetyl	1506.73950	753.87339	13
3	401.20309	201.10518	T	1336.63396	668.82062	12
4	472.24021	236.62374	A	1235.58628	618.29678	11
5	601.28281	301.14504	E	1164.54916	582.77822	10
6	702.33049	351.66888	T	1035.50656	518.25692	9
7	789.36252	395.18490	S	934.45888	467.73308	8
8	876.39455	438.70091	S	847.42685	424.21706	7
9	977.44223	489.22475	T	760.39482	380.70105	6
10	1106.48483	553.74605	E	659.34714	330.17721	5
11	1220.52776	610.76752	N	530.30454	265.65591	4
12	1348.58634	674.79681	Q	416.26161	208.63444	3
13	1461.67041	731.33884	I	288.20303	144.60515	2
14			R	175.11896	88.06312	1

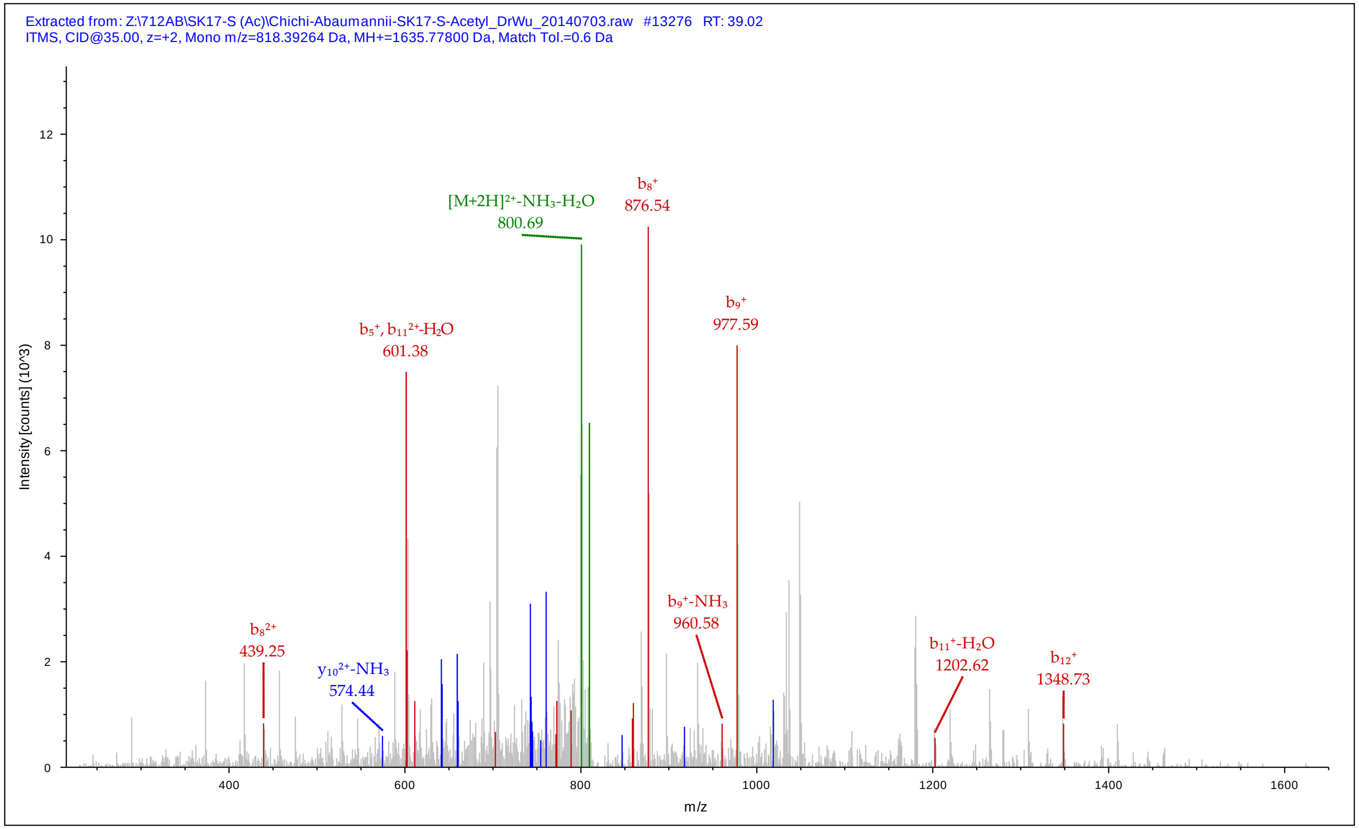

peg.1803 26971

#1	b+	b2+	b3+	Seq.	y+	y2+	y3+	#2
1	190.08626	95.54677	64.03360	F-Acetyl				20
2	247.10773	124.05750	83.04076	G	1999.08193	1000.04460	667.03216	19
3	361.15066	181.07897	121.05507	N	1942.06046	971.53387	648.02500	18
4	489.24563	245.12645	163.75339	K	1828.01753	914.51240	610.01069	17
5	588.31405	294.66066	196.77620	V	1699.92256	850.46492	567.31237	16
6	675.34608	338.17668	225.78688	S	1600.85414	800.93071	534.28956	15
7	772.39885	386.70306	258.13780	P	1513.82211	757.41469	505.27889	14
8	871.46727	436.23727	291.16061	V	1416.76934	708.88831	472.92796	13
9	928.48874	464.74801	310.16776	G	1317.70092	659.35410	439.90516	12
10	1056.54732	528.77730	352.85396	Q	1260.67945	630.84336	420.89800	11
11	1157.59500	579.30114	386.53652	T	1132.62087	566.81407	378.21181	10
12	1327.70054	664.35391	443.23836	K-Acetyl	1031.57319	516.29023	344.52925	9
13	1428.74822	714.87775	476.92092	T	861.46765	431.23746	287.82740	8
14	1541.83229	771.41978	514.61561	L	760.41997	380.71362	254.14484	7
15	1638.88506	819.94617	546.96654	P	647.33590	324.17159	216.45015	6
16	1739.93274	870.47001	580.64910	T	550.28313	275.64520	184.09923	5
17	1826.96477	913.98602	609.65977	S	449.23545	225.12136	150.41667	4
18	1928.01245	964.50986	643.34233	T	362.20342	181.60535	121.40599	3
19	2042.05538	1021.53133	681.35664	N	261.15574	131.08151	87.72343	2
20				K	147.11281	74.06004	49.70912	1

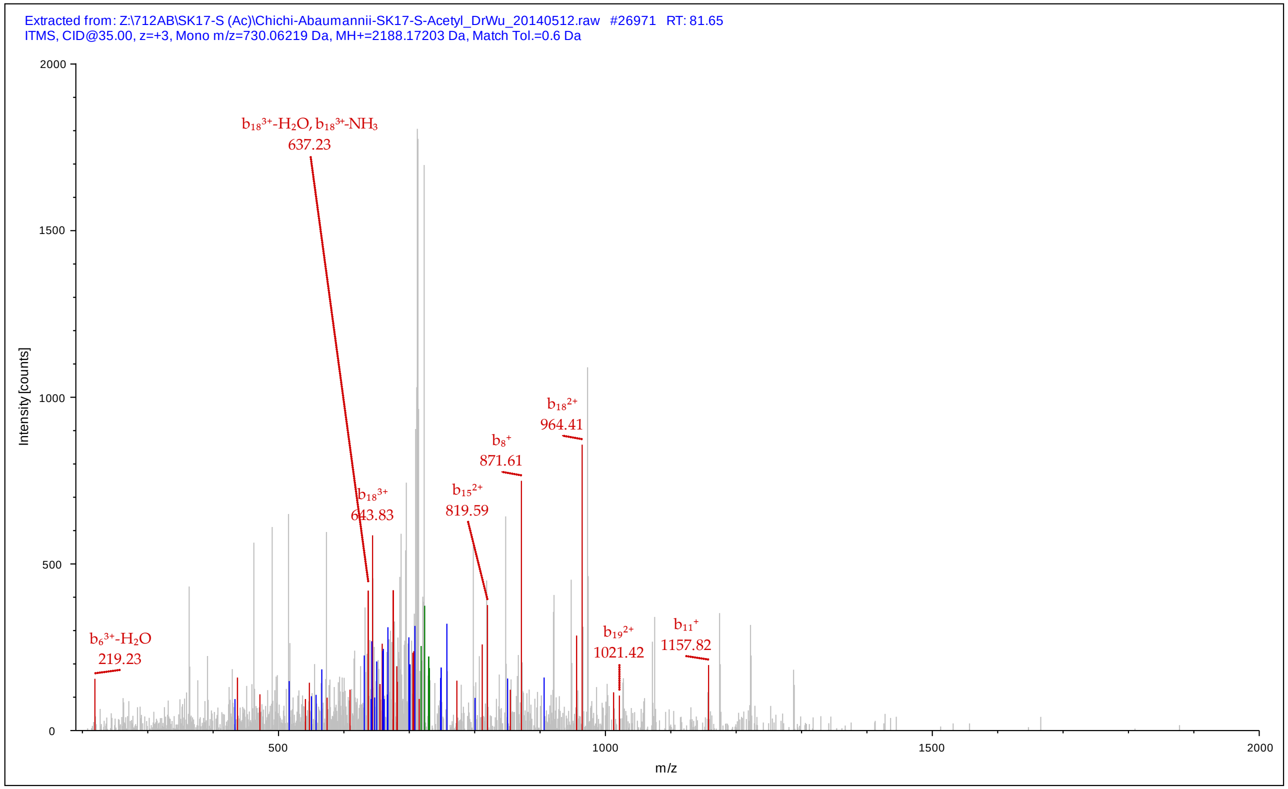

peg.1811 24884

#1	b⁺	b²⁺	Seq.	y⁺	y²⁺	#2
1	114.05496	57.53112	A-Acetyl			16
2	213.12338	107.06533	V	1658.93241	829.96984	15
3	300.15541	150.58134	S	1559.86399	780.43563	14
4	401.20309	201.10518	T	1472.83196	736.91962	13
5	529.29806	265.15267	K	1371.78428	686.39578	12
6	600.33518	300.67123	A	1243.68931	622.34829	11
7	701.38286	351.19507	T	1172.65219	586.82973	10
8	829.44144	415.22436	Q	1071.60451	536.30589	9
9	928.50986	464.75857	V	943.54593	472.27660	8
10	1098.61540	549.81134	K-Acetyl	844.47751	422.74239	7
11	1227.65800	614.33264	E	674.37197	337.68962	6
12	1326.72642	663.86685	V	545.32937	273.16832	5
13	1425.79484	713.40106	V	446.26095	223.63411	4
14	1496.83196	748.91962	A	347.19253	174.09990	3
15	1625.87456	813.44092	E	276.15541	138.58134	2
16			K	147.11281	74.06004	1

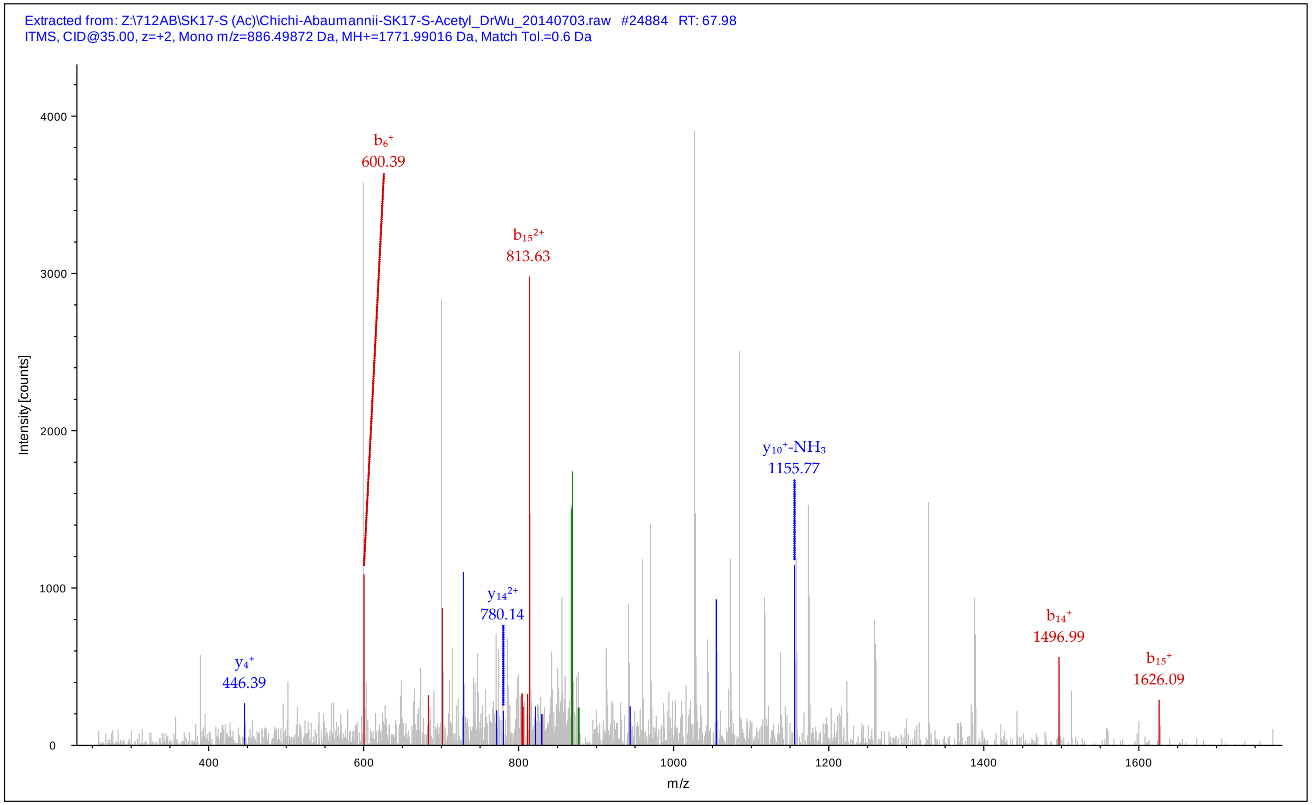

peg.1833 23498

#1	b⁺	b²⁺	Seq.	y⁺	y²⁺	#2
1	114.05496	57.53112	A-Acetyl			17
2	201.08699	101.04713	S	1906.99169	953.99948	16
3	316.11394	158.56061	D	1819.95966	910.48347	15
4	413.16671	207.08699	P	1704.93271	852.96999	14
5	542.20931	271.60829	E	1607.87994	804.44361	13
6	613.24643	307.12685	A	1478.83734	739.92231	12
7	783.35197	392.17962	K-Acetyl	1407.80022	704.40375	11
8	896.43604	448.72166	L	1237.69468	619.35098	10
9	1052.53716	526.77222	R	1124.61061	562.80894	9
10	1166.58009	583.79368	N	968.50949	484.75838	8
11	1294.63867	647.82297	Q	854.46656	427.73692	7
12	1365.67579	683.34153	A	726.40798	363.70763	6
13	1464.74421	732.87574	V	655.37086	328.18907	5
14	1601.80312	801.40520	H	556.30244	278.65486	4
15	1714.88719	857.94723	L	419.24353	210.12540	3
16	1845.92769	923.46748	M	306.15946	153.58337	2
17			R	175.11896	88.06312	1

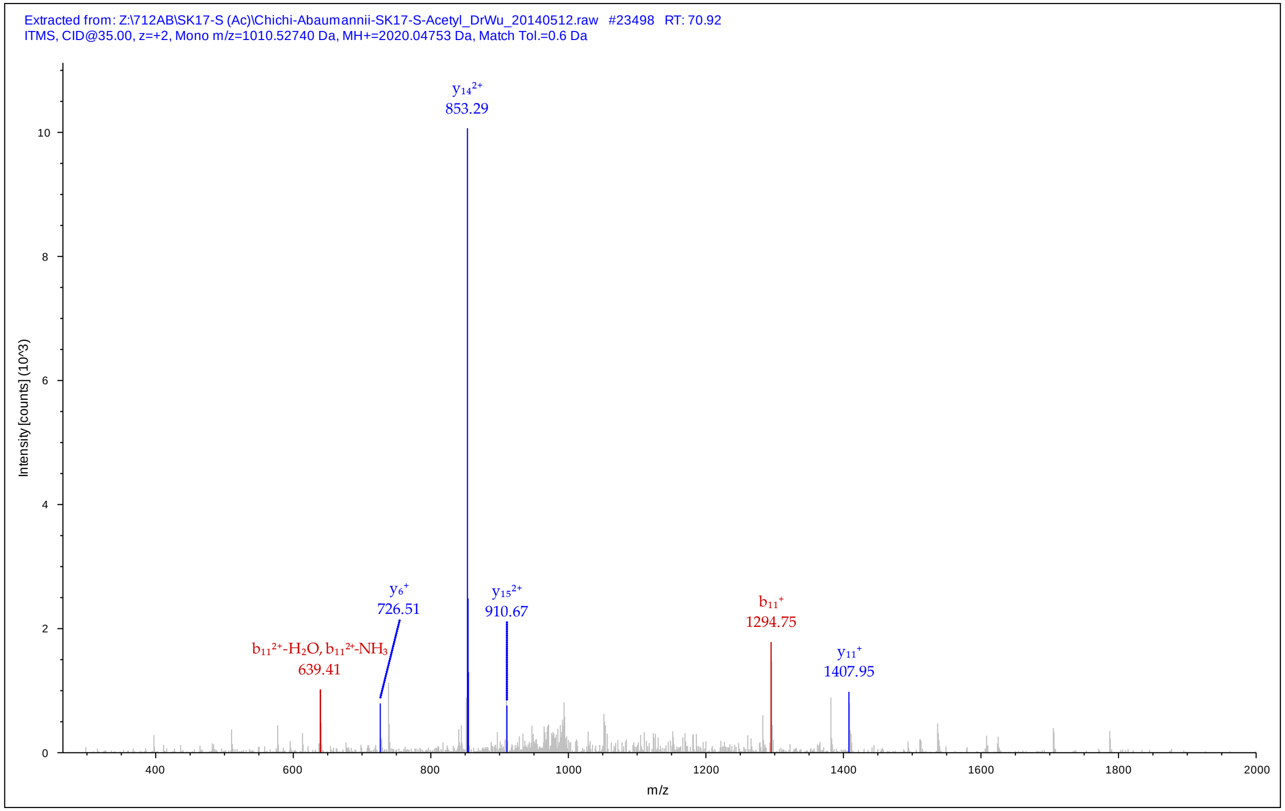

peg.1836 25733

#1	b+	b2+	b3+	Seq.	y+	y2+	y3+	#2
1	174.05834	87.53281	58.69096	M-Acetyl				33
2	261.09037	131.04882	87.70164	S	3556.77037	1778.88882	1186.26164	32
3	389.18534	195.09631	130.39996	K	3469.73834	1735.37281	1157.25096	31
4	502.26941	251.63834	168.09465	I	3341.64337	1671.32532	1114.55264	30
5	615.35348	308.18038	205.78934	I	3228.55930	1614.78329	1076.85795	29
6	686.39060	343.69894	229.46838	A	3115.47523	1558.24125	1039.16326	28
7	789.39979	395.20353	263.80478	C	3044.43811	1522.72269	1015.48422	27
8	902.48386	451.74557	301.49947	I	2941.42892	1471.21810	981.14782	26
9	1017.51081	509.25904	339.84179	D	2828.34485	1414.67606	943.45313	25
10	1074.53228	537.76978	358.84894	G	2713.31790	1357.16259	905.11082	24
11	1161.56431	581.28579	387.85962	S	2656.29643	1328.65185	886.10366	23
12	1274.64838	637.82783	425.55431	L	2569.26440	1285.13584	857.09298	22
13	1373.71680	687.36204	458.57712	V	2456.18033	1228.59380	819.39829	21
14	1474.76448	737.88588	492.25968	T	2357.11191	1179.05959	786.37549	20
15	1588.80741	794.90734	530.27399	N	2256.06423	1128.53575	752.69293	19
16	1689.85509	845.43118	563.95655	T	2142.02130	1071.51429	714.67862	18
17	1788.92351	894.96539	596.97935	V	2040.97362	1020.99045	680.99606	17
18	1891.93270	946.46999	631.31575	C	1941.90520	971.45624	647.97325	16
19	2006.95965	1003.98346	669.65807	D	1838.89601	919.95164	613.63685	15
20	2170.02297	1085.51512	724.01251	Y	1723.86906	862.43817	575.29454	14
21	2241.06009	1121.03368	747.69155	A	1560.80574	780.90651	520.94010	13
22	2312.09721	1156.55224	771.37059	A	1489.76862	745.38795	497.26106	12
23	2498.17653	1249.59190	833.39703	W	1418.73150	709.86939	473.58202	11
24	2645.24495	1323.12611	882.41983	F	1232.65218	616.82973	411.55558	10
25	2732.27698	1366.64213	911.43051	S	1085.58376	543.29552	362.53277	9
26	2847.30393	1424.15560	949.77283	D	998.55173	499.77950	333.52209	8
27	3017.40947	1509.20837	1006.47467	K-Acetyl	883.52478	442.26603	295.17978	7
28	3130.49354	1565.75041	1044.16936	L	713.41925	357.21326	238.47793	6
29	3244.53647	1622.77187	1082.18367	N	600.33518	300.67123	200.78324	5
30	3331.56850	1666.28789	1111.19435	S	486.29225	243.64976	162.76893	4
31	3428.62127	1714.81427	1143.54527	P	399.26022	200.13375	133.75826	3
32	3541.70534	1771.35631	1181.23996	I	302.20745	151.60736	101.40733	2
33				K-Acetyl	189.12338	95.06533	63.71264	1

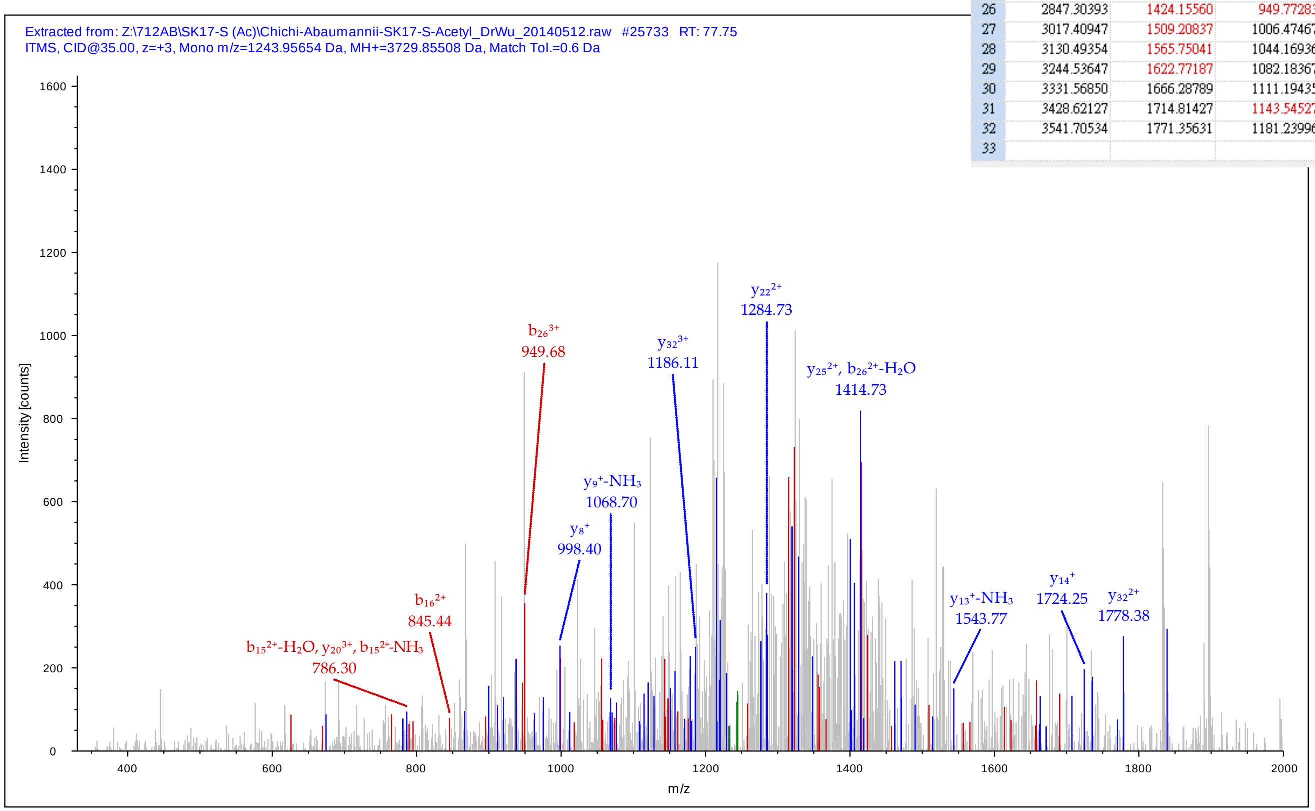

peg.1837 21969

#1	b^+	b^{2+}	b^{3+}	Seq.	y^+	y^{2+}	y^{3+}	#2
1	172.06044	86.53386	58.02500	E-Acetyl				36
2	275.06963	138.03845	92.36139	C	3422.68019	1711.84373	1141.56491	35
3	445.17517	223.09122	149.06324	K-Acetyl	3319.67100	1660.33914	1107.22852	34
4	502.19664	251.60196	168.07040	G	3149.56547	1575.28637	1050.52667	33
5	630.25522	315.63125	210.75659	Q	3092.54400	1546.77564	1031.51952	32
6	687.27669	344.14198	229.76375	G	2964.48542	1482.74635	988.83332	31
7	786.34511	393.67619	262.78655	V	2907.46395	1454.23561	969.82617	30
8	857.38223	429.19475	286.46559	A	2808.39553	1404.70140	936.80336	29
9	971.42516	486.21622	324.47990	N	2737.35841	1369.18284	913.12432	28
10	1070.49358	535.75043	357.50271	V	2623.31548	1312.16138	875.11001	27
11	1141.53070	571.26899	381.18175	A	2524.24706	1262.62717	842.08720	26
12	1228.56273	614.78500	410.19243	S	2453.20994	1227.10861	818.40816	25
13	1285.58420	643.29574	429.19958	G	2366.17791	1183.59259	789.39749	24
14	1432.65262	716.82995	478.22239	F	2309.15644	1155.08186	770.39033	23
15	1545.73669	773.37198	515.91708	L	2162.08802	1081.54765	721.36752	22
16	1602.75816	801.88272	534.92424	G	2049.00395	1025.00561	683.67283	21
17	1659.77963	830.39345	553.93139	G	1991.98248	996.49488	664.66568	20
18	1790.82013	895.91370	597.61156	M	1934.96101	967.98414	645.65852	19
19	1861.85725	931.43226	621.29060	A	1803.92051	902.46389	601.97835	18
20	1918.87872	959.94300	640.29776	G	1732.88339	866.94533	578.29931	17
21	2021.88791	1011.44759	674.63415	C	1675.86192	838.43460	559.29216	16
22	2092.92503	1046.96615	698.31319	A	1572.85273	786.93000	524.95576	15
23	2223.96553	1112.48640	741.99336	M	1501.81561	751.41144	501.27672	14
24	2337.04960	1169.02844	779.68805	I	1370.77511	685.89119	457.59655	13
25	2394.07107	1197.53917	798.69521	G	1257.69104	629.34916	419.90186	12
26	2522.12965	1261.56846	841.38140	Q	1200.66957	600.83842	400.89471	11
27	2609.16168	1305.08448	870.39208	S	1072.61099	536.80913	358.20851	10
28	2722.24575	1361.62651	908.08677	I	985.57896	493.29312	329.19784	9
29	2835.32982	1418.16855	945.78146	I	872.49489	436.75108	291.50315	8
30	2949.37275	1475.19001	983.79577	N	759.41082	380.20905	253.80846	7
31	3048.44117	1524.72422	1016.81857	V	645.36789	323.18758	215.79415	6
32	3218.54670	1609.77699	1073.52042	K-Acetyl	546.29947	273.65337	182.77134	5
33	3305.57873	1653.29300	1102.53109	S	376.19393	188.60060	126.06949	4
34	3362.60020	1681.80374	1121.53825	G	289.16190	145.08459	97.05882	3
35	3419.62167	1710.31447	1140.54541	G	232.14043	116.57385	78.05166	2
36				R	175.11896	88.06312	59.04450	1

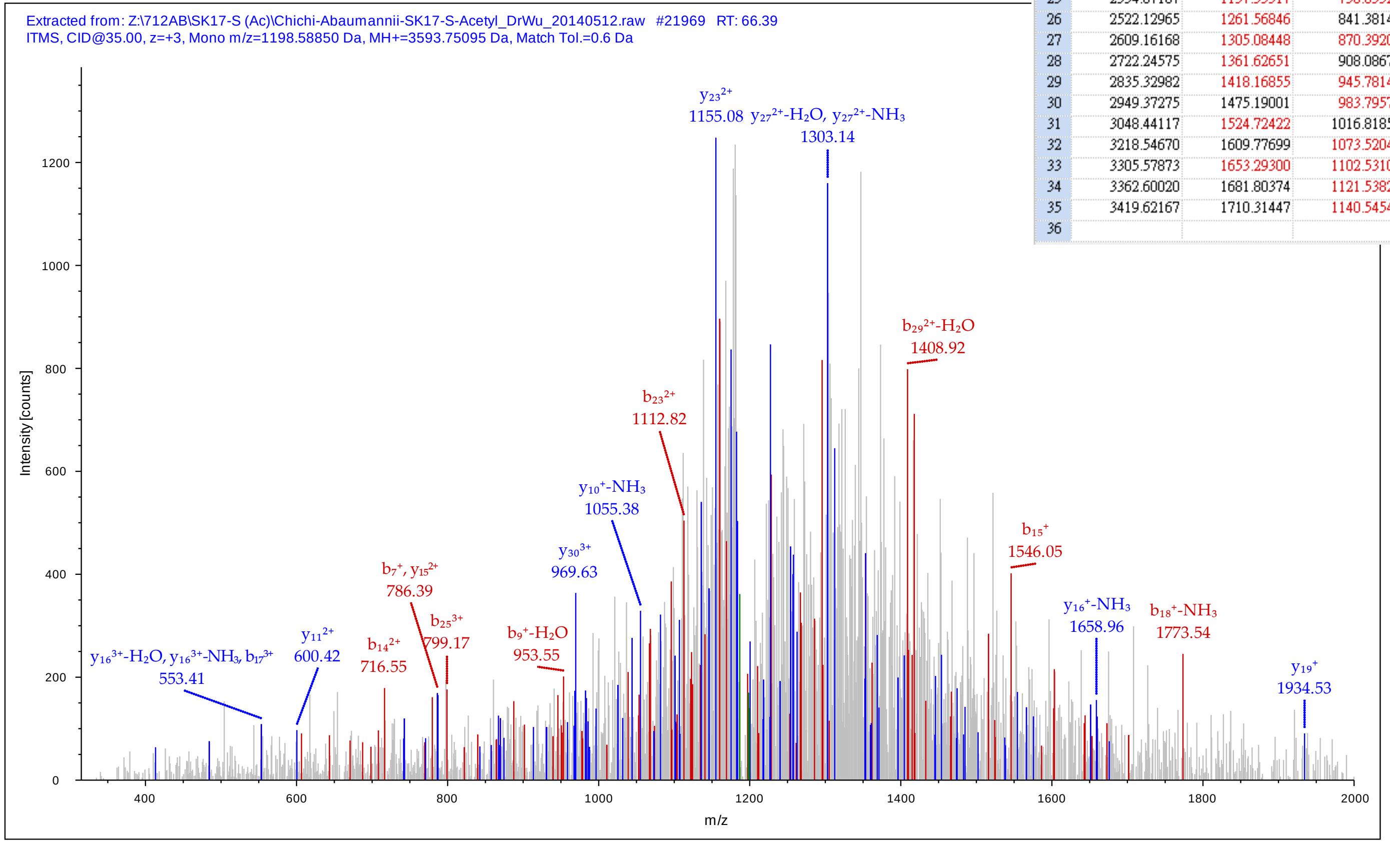

peg.1846 19805

#1	b⁺	b²⁺	b³⁺	Seq.	y⁺	y²⁺	y³⁺	#2
1	156.10191	78.55459	52.70549	L-Acetyl				19
2	303.17033	152.08880	101.72829	F	2468.17315	1234.59021	823.39590	18
3	434.21083	217.60905	145.40846	M	2321.10473	1161.05600	774.37309	17
4	547.29490	274.15109	183.10315	L	2190.06423	1095.53575	730.69293	16
5	694.36332	347.68530	232.12596	F	2076.98016	1038.99372	692.99824	15
6	864.46886	432.73807	288.82780	K-Acetyl	1929.91174	965.45951	643.97543	14
7	965.51654	483.26191	322.51036	T	1759.80620	880.40674	587.27358	13
8	1080.54349	540.77538	360.85268	D	1658.75852	829.88290	553.59102	12
9	1209.58609	605.29668	403.86688	E	1543.73157	772.36942	515.24871	11
10	1356.65451	678.83089	452.88969	F	1414.68897	707.84812	472.23451	10
11	1471.68146	736.34437	491.23200	D	1267.62055	634.31391	423.21170	9
12	1584.76553	792.88640	528.92669	I	1152.59360	576.80044	384.86938	8
13	1713.80813	857.40770	571.94089	E	1039.50953	520.25840	347.17469	7
14	1883.91366	942.46047	628.64274	K-Acetyl	910.46693	455.73710	304.16049	6
15	2011.97224	1006.48976	671.32893	Q	740.36140	370.68434	247.45865	5
16	2141.01484	1071.01106	714.34313	E	612.30282	306.65505	204.77246	4
17	2288.08326	1144.54527	763.36594	F	483.26022	242.13375	161.75826	3
18	2435.15168	1218.07948	812.38874	F	336.19180	168.59954	112.73545	2
19				K-Acetyl	189.12338	95.06533	63.71264	1

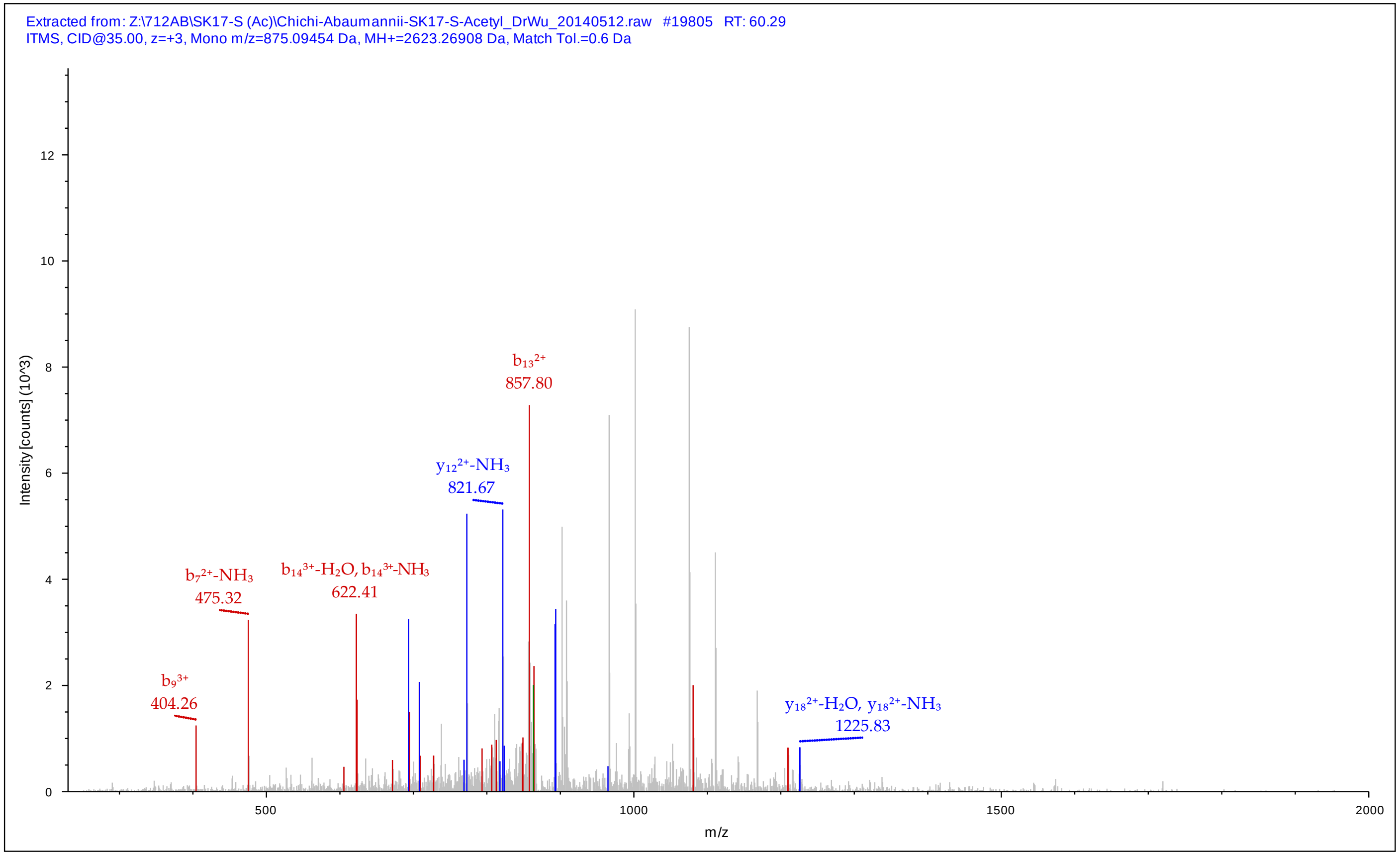

peg.1865 15269

#1	b⁺	b²⁺	Seq.	y⁺	y²⁺	#2
1	72.04440	36.52584	A			14
2	129.06587	65.03657	G	1378.69620	689.85174	13
3	244.09282	122.55005	D	1321.67473	661.34100	12
4	373.13542	187.07135	E	1206.64778	603.82753	11
5	430.15689	215.58208	G	1077.60518	539.30623	10
6	600.26242	300.63485	K-Acetyl	1020.58371	510.79549	9
7	713.34649	357.17688	L	850.47817	425.74272	8
8	860.41491	430.71109	F	737.39410	369.20069	7
9	917.43638	459.22183	G	590.32568	295.66648	6
10	1004.46841	502.73784	S	533.30421	267.15574	5
11	1117.55248	559.27988	I	446.27218	223.63973	4
12	1174.57395	587.79061	G	333.18811	167.09769	3
13	1275.62163	638.31445	T	276.16664	138.58696	2
14			R	175.11896	88.06312	1

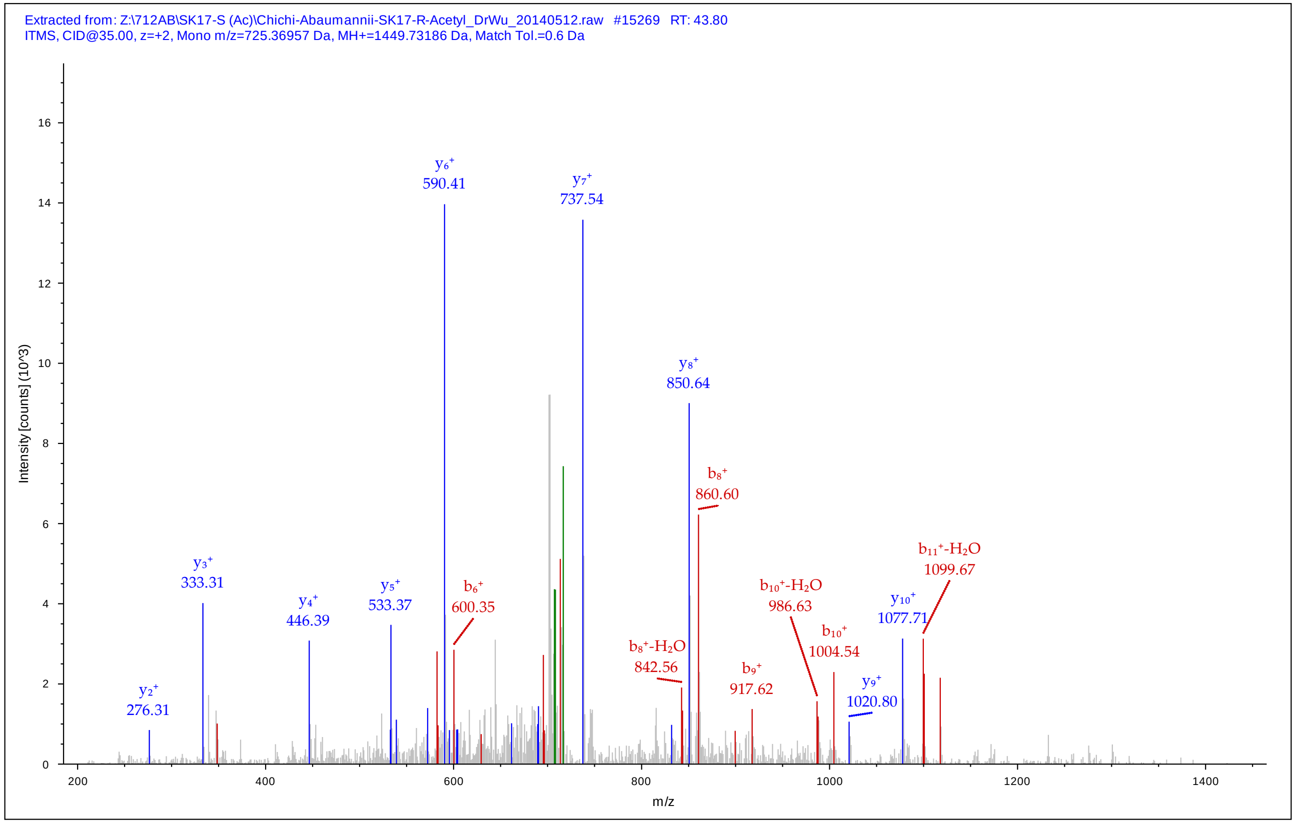

peg.1865 13528

#1	b+	b2+	Seq.	y+	y2+	#2
1	72.04440	36.52584	A			14
2	129.06587	65.03657	G	1378.69620	689.85174	13
3	244.09282	122.55005	D	1321.67473	661.34100	12
4	373.13542	187.07135	E	1206.64778	603.82753	11
5	430.15689	215.58208	G	1077.60518	539.30623	10
6	600.26242	300.63485	K-Acetyl	1020.58371	510.79549	9
7	713.34649	357.17688	L	850.47817	425.74272	8
8	860.41491	430.71109	F	737.39410	369.20069	7
9	917.43638	459.22183	G	590.32568	295.66648	6
10	1004.46841	502.73784	S	533.30421	267.15574	5
11	1117.55248	559.27988	I	446.27218	223.63973	4
12	1174.57395	587.79061	G	333.18811	167.09769	3
13	1275.62163	638.31445	T	276.16664	138.58696	2
14			R	175.11896	88.06312	1

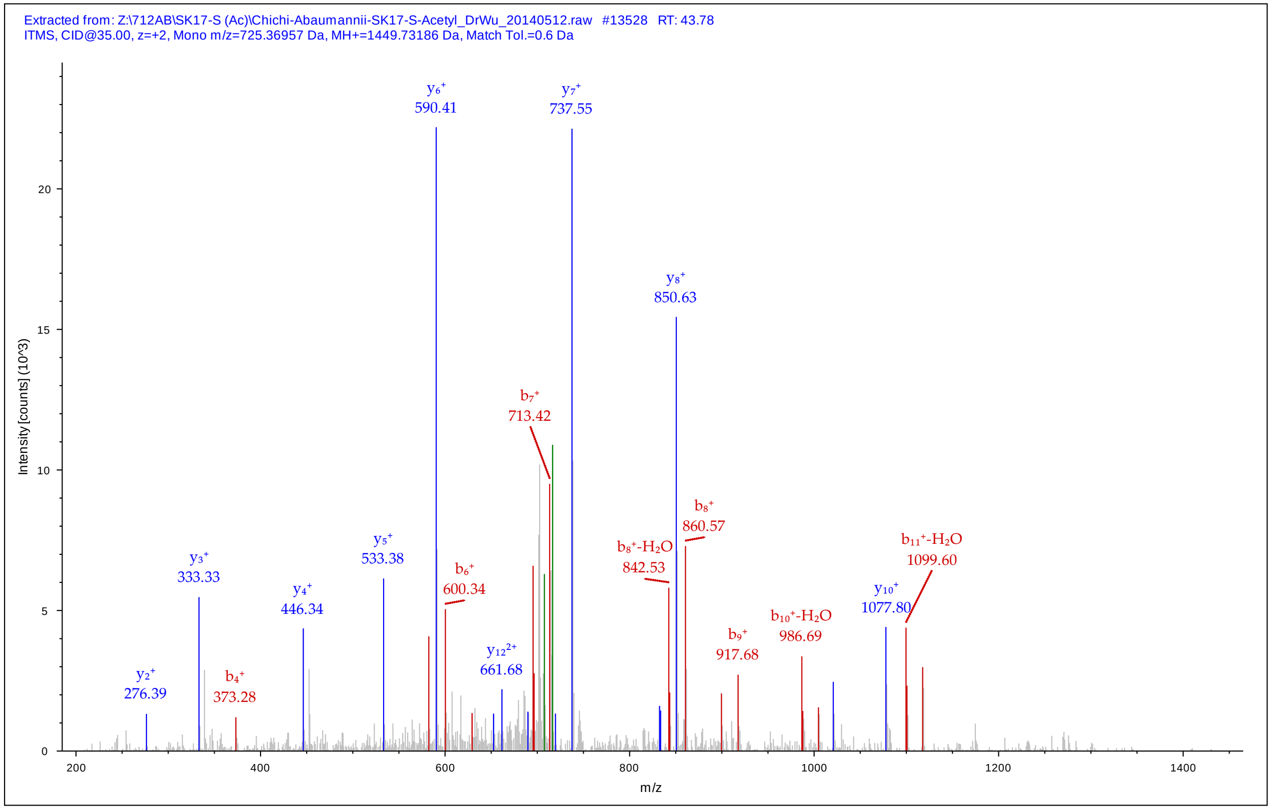

peg.1914   4087

#1	b⁺	b²⁺	Seq.	y⁺	y²⁺	#2
1	114.09135	57.54931	L			12
2	261.15977	131.08352	F	1368.62129	684.81428	11
3	348.19180	174.59954	S	1221.55287	611.28007	10
4	435.22383	218.11555	S	1134.52084	567.76406	9
5	563.28241	282.14484	Q	1047.48881	524.24804	8
6	733.38794	367.19761	K-Acetyl	919.43023	460.21875	7
7	847.43087	424.21907	N	749.32470	375.16599	6
8	1007.46153	504.23440	C-Carbam···	635.28177	318.14452	5
9	1136.50413	568.75570	E	475.25111	238.12919	4
10	1264.59910	632.80319	K	346.20851	173.60789	3
11	1392.65768	696.83248	Q	218.11354	109.56041	2
12			A	90.05496	45.53112	1

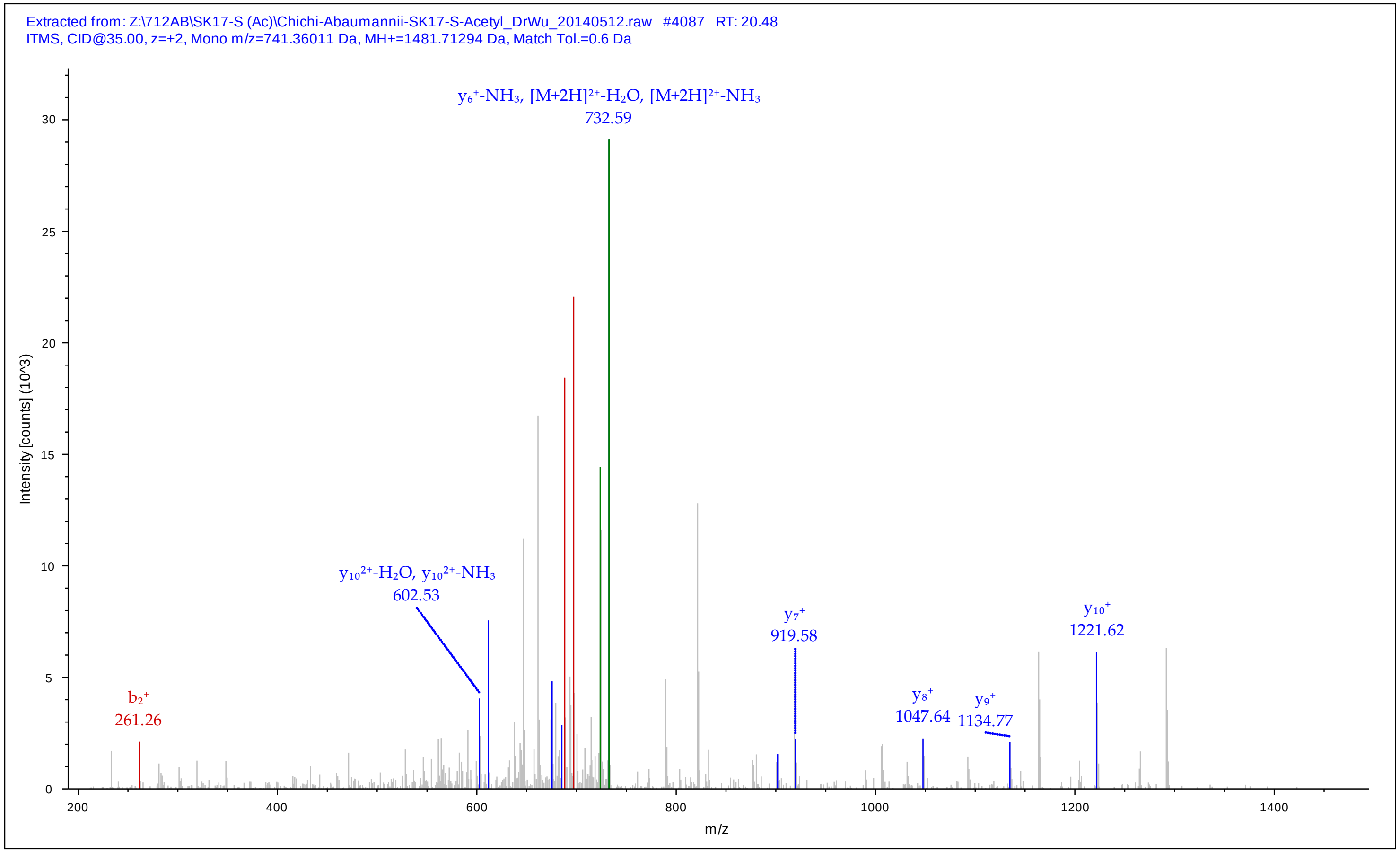

peg.1976   13587

#1	b⁺	b²⁺	Seq.	y⁺	y²⁺	#2
1	171.11281	86.06004	K-Acetyl			10
2	228.13428	114.57078	G	986.56297	493.78512	9
3	356.22925	178.61826	K	929.54150	465.27439	8
4	469.31332	235.16030	L	801.44653	401.22690	7
5	583.35625	292.18176	N	688.36246	344.68487	6
6	698.38320	349.69524	D	574.31953	287.66340	5
7	811.46727	406.23727	L	459.29258	230.14993	4
8	939.52585	470.26656	Q	346.20851	173.60789	3
9	1010.56297	505.78512	A	218.14993	109.57860	2
10			K	147.11281	74.06004	1

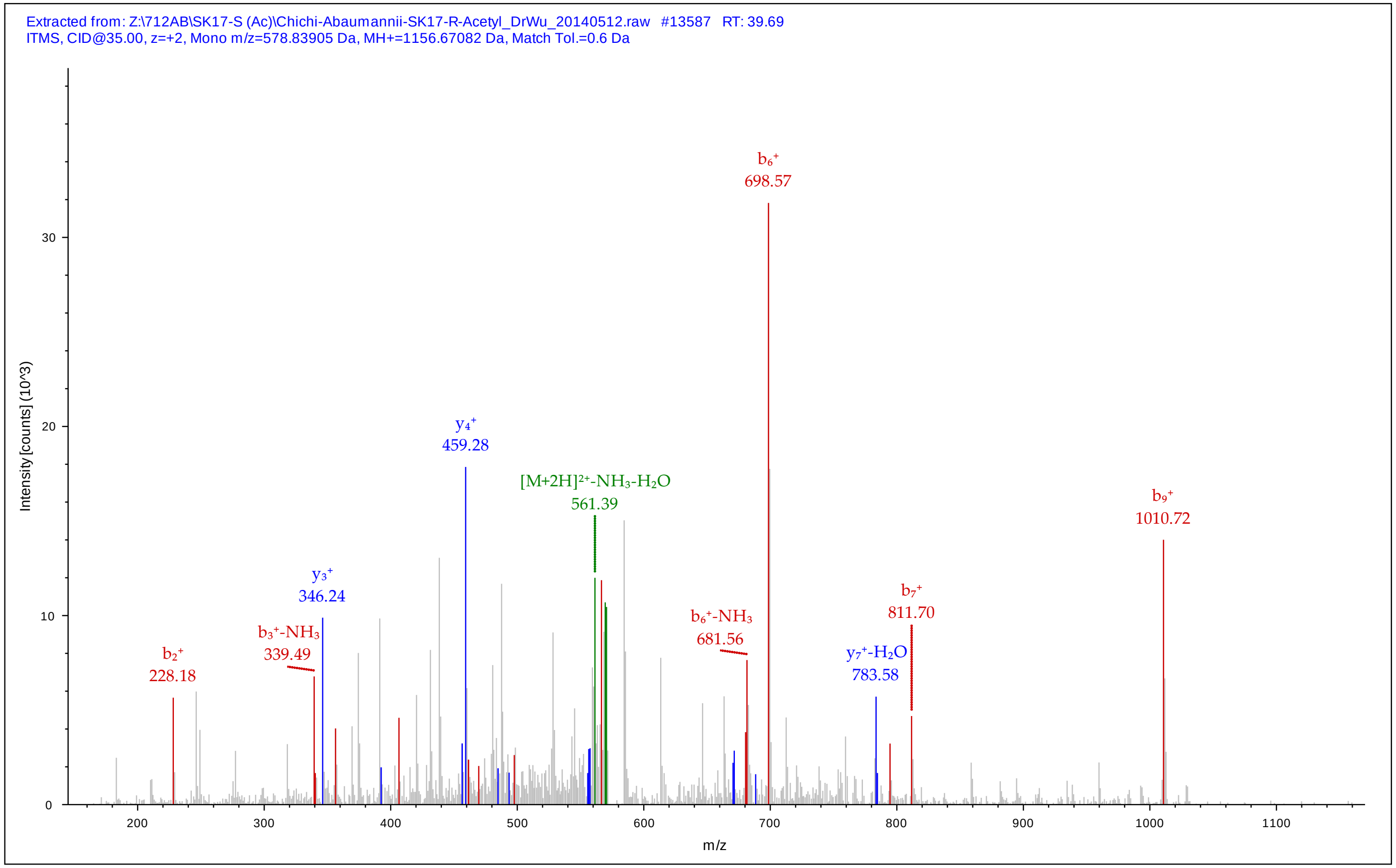

peg.1976   12005

#1	b⁺	b²⁺	Seq.	y⁺	y²⁺	#2
1	171.11281	86.06004	K-Acetyl			10
2	228.13428	114.57078	G	986.56297	493.78512	9
3	356.22925	178.61826	K	929.54150	465.27439	8
4	469.31332	235.16030	L	801.44653	401.22690	7
5	583.35625	292.18176	N	688.36246	344.68487	6
6	698.38320	349.69524	D	574.31953	287.66340	5
7	811.46727	406.23727	L	459.29258	230.14993	4
8	939.52585	470.26656	Q	346.20851	173.60789	3
9	1010.56297	505.78512	A	218.14993	109.57860	2
10			K	147.11281	74.06004	1

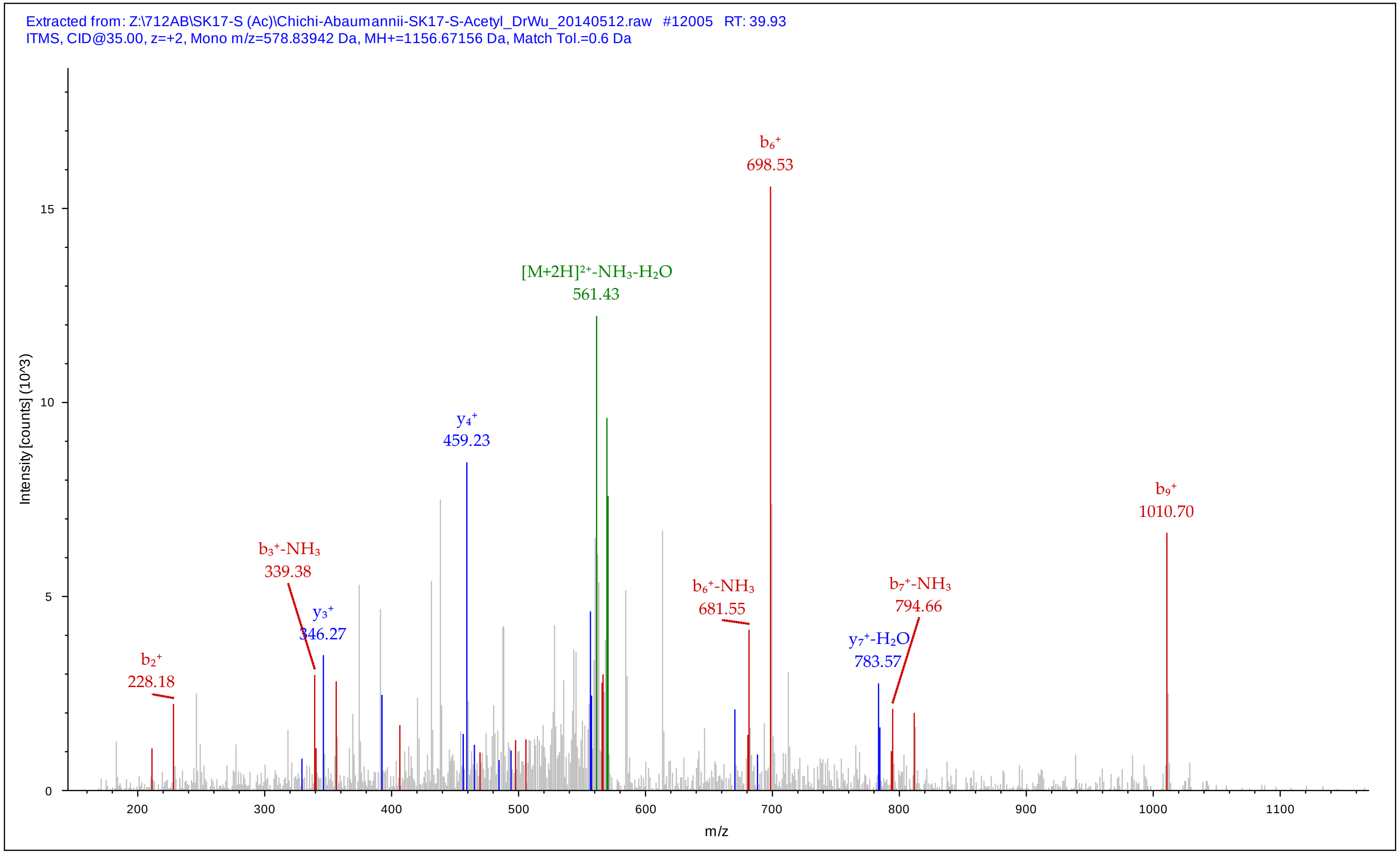

peg.1982 20002

#1	b^{+}	b^{2+}	b^{3+}	Seq.	y^{+}	y^{2+}	y^{3+}	#2
1	129.06586	65.03657	43.69347	Q				16
2	228.13428	114.57078	76.71628	V	1797.99978	899.50353	600.00478	15
3	357.17688	179.09208	119.73048	E	1698.93136	849.96932	566.98197	14
4	485.27185	243.13956	162.42880	K	1569.88876	785.44802	523.96777	13
5	582.32462	291.66595	194.77972	P	1441.79379	721.40053	481.26945	12
6	752.43015	376.71871	251.48157	K-Acetyl	1344.74102	672.87415	448.91852	11
7	865.51422	433.26075	289.17626	L	1174.63548	587.82138	392.21668	10
8	962.56699	481.78713	321.52718	P	1061.55141	531.27934	354.52199	9
9	1091.60959	546.30843	364.54138	E	964.49864	482.75296	322.17106	8
10	1204.69366	602.85047	402.23607	I	835.45604	418.23166	279.15686	7
11	1319.72061	660.36394	440.57839	D	722.37197	361.68962	241.46217	6
12	1390.75773	695.88250	464.25743	A	607.34502	304.17615	203.11986	5
13	1519.80033	760.40380	507.27163	E	536.30790	268.65759	179.44082	4
14	1666.86875	833.93801	556.29443	F	407.26530	204.13629	136.42662	3
15	1779.95282	890.48005	593.98912	L	260.19688	130.60208	87.40381	2
16				K	147.11281	74.06004	49.70912	1

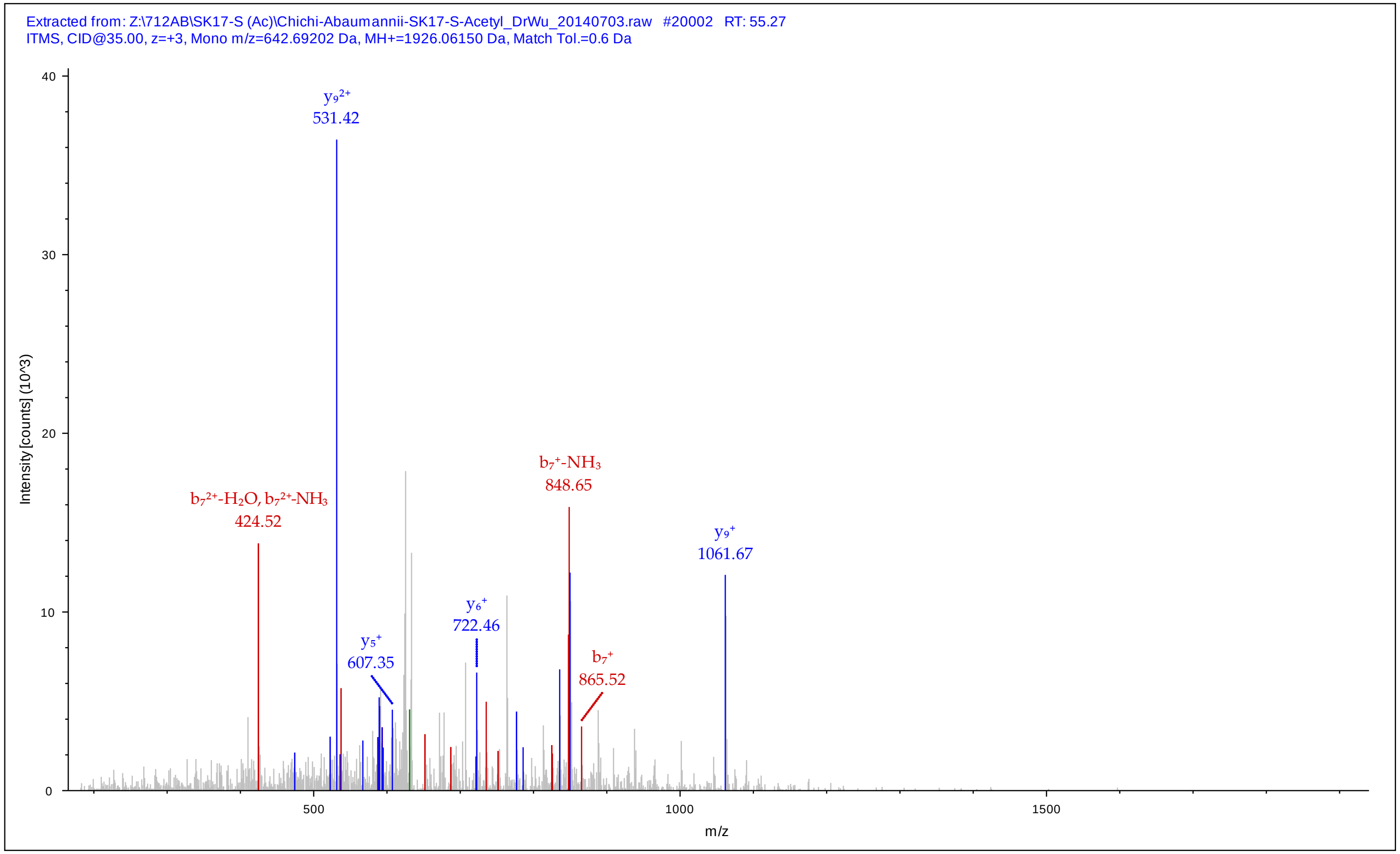

peg.2009   23240

#1	b⁺	b²⁺	Seq.	y⁺	y²⁺	#2
1	132.04778	66.52753	M			15
2	245.13185	123.06956	L	1829.06058	915.03393	14
3	360.15880	180.58304	D	1715.97651	858.49189	13
4	530.26433	265.63580	K-Acetyl	1600.94956	800.97842	12
5	643.34840	322.17784	L	1430.84402	715.92565	11
6	756.43247	378.71987	L	1317.75995	659.38361	10
7	869.51654	435.26191	L	1204.67588	602.84158	9
8	982.60061	491.80394	L	1091.59181	546.29954	8
9	1110.65919	555.83323	Q	978.50774	489.75751	7
10	1207.71196	604.35962	P	850.44916	425.72822	6
11	1367.74262	684.37495	C-Carbam…	753.39639	377.20183	5
12	1480.82669	740.91698	L	593.36574	297.18651	4
13	1643.89001	822.44864	Y	480.28167	240.64447	3
14	1813.99554	907.50141	K-Acetyl	317.21835	159.11281	2
15			K	147.11281	74.06004	1

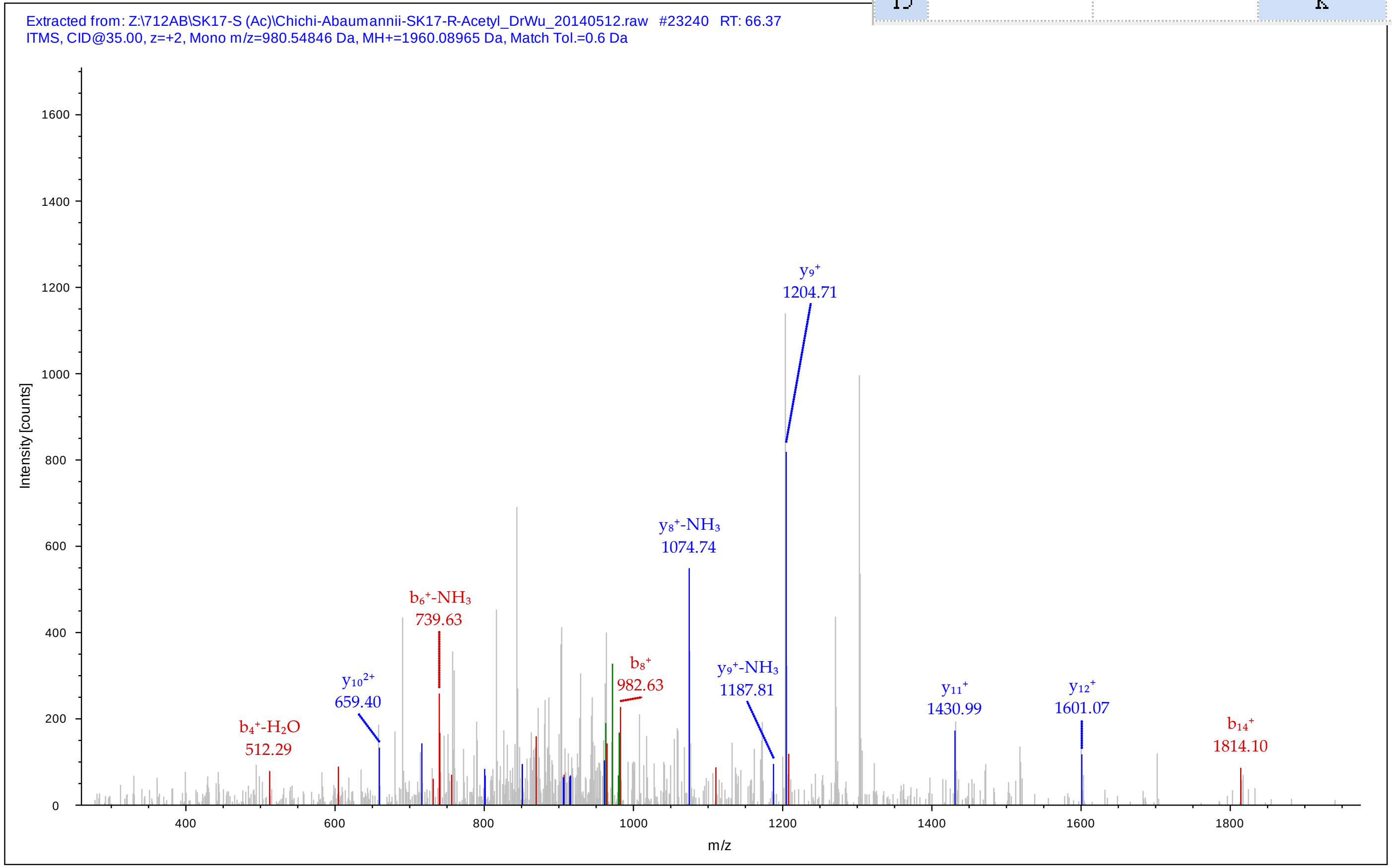

peg.2053 25315

#1	b^+	b^{2+}	b^{3+}	Seq.	y^+	y^{2+}	y^{3+}	#2
1	116.03423	58.52075	39.34959	D				22
2	173.05570	87.03149	58.35675	G	2342.17318	1171.59023	781.39591	21
3	333.08635	167.04681	111.70030	C-Carbam···	2285.15171	1143.07949	762.38875	20
4	404.12347	202.56537	135.37934	A	2125.12105	1063.06416	709.04520	19
5	501.17624	251.09176	167.73026	P	2054.08393	1027.54560	685.36616	18
6	630.21884	315.61306	210.74446	E	1957.03116	979.01922	653.01524	17
7	745.24579	373.12653	249.08678	D	1827.98856	914.49792	610.00104	16
8	844.31421	422.66074	282.10959	V	1712.96161	856.98444	571.65872	15
9	915.35133	458.17930	305.78863	A	1613.89319	807.45023	538.63591	14
10	1044.39393	522.70060	348.80283	E	1542.85607	771.93167	514.95687	13
11	1157.47800	579.24264	386.49752	L	1413.81347	707.41037	471.94267	12
12	1256.54642	628.77685	419.52032	V	1300.72940	650.86834	434.24798	11
13	1327.58354	664.29541	443.19936	A	1201.66098	601.33413	401.22518	10
14	1455.64212	728.32470	485.88556	Q	1130.62386	565.81557	377.54614	9
15	1602.67754	801.84241	534.89736	M-Oxidation	1002.56528	501.78628	334.85994	8
16	1689.70957	845.35842	563.90804	S	855.52987	428.26857	285.84814	7
17	1817.76815	909.38771	606.59423	Q	768.49784	384.75256	256.83746	6
18	1930.85222	965.92975	644.28892	L	640.43926	320.72327	214.15127	5
19	2027.90499	1014.45613	676.63985	P	527.35519	264.18123	176.45658	4
20	2198.01052	1099.50890	733.34169	K-Acetyl	430.30242	215.65485	144.10566	3
21	2311.09459	1156.05093	771.03638	I	260.19688	130.60208	87.40381	2
22				K	147.11281	74.06004	49.70912	1

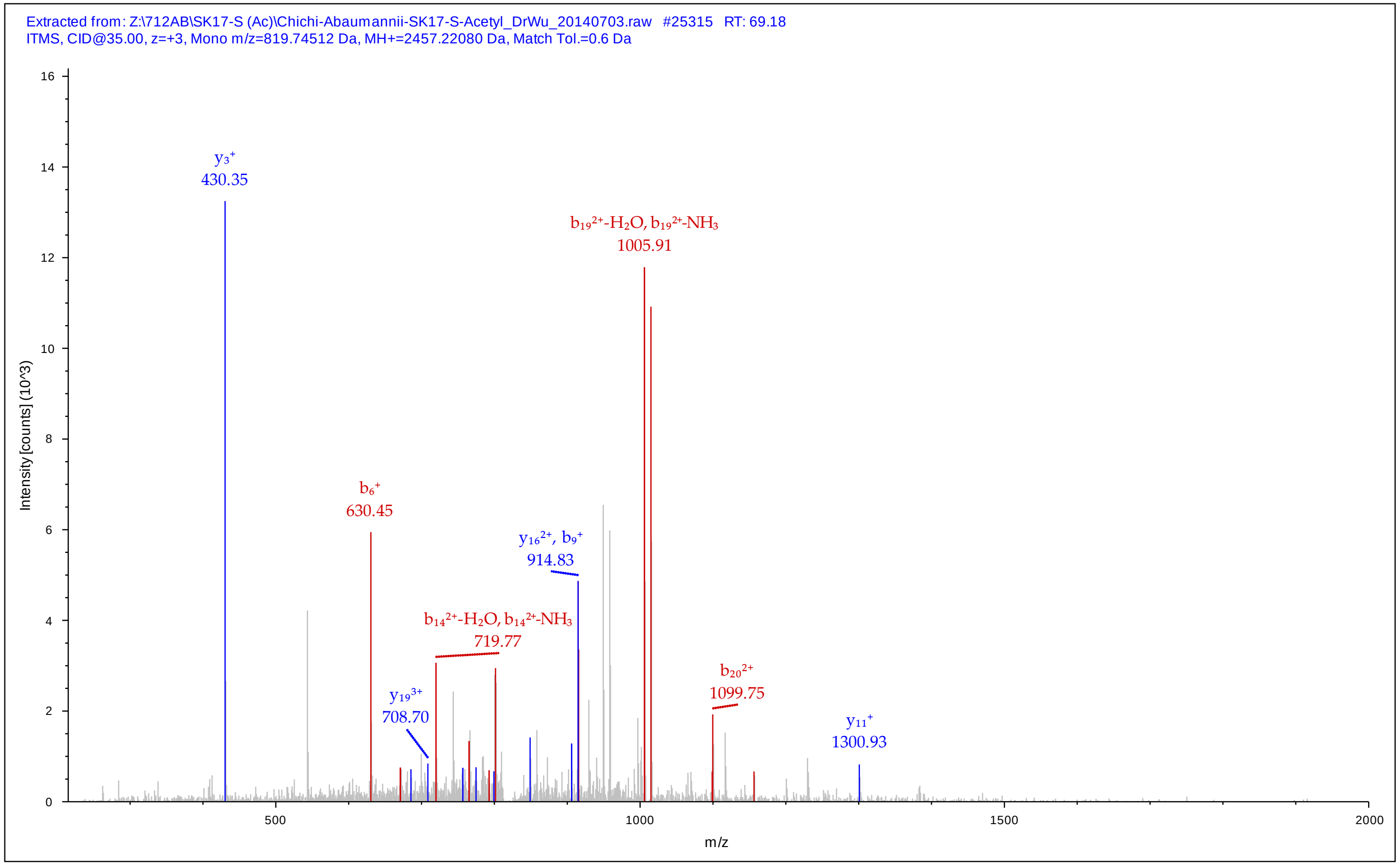

peg.2065 24992

#1	b⁺	b^{2+}	b^{3+}	Seq.	y^+	y^{2+}	y^{3+}	#2
1	206.08116	103.54422	69.36524	Y-Acetyl				19
2	353.11658	177.06193	118.37704	M-Oxidation	1929.01850	965.01289	643.67768	18
3	452.18500	226.59614	151.39985	V	1781.98308	891.49518	594.66588	17
4	551.25342	276.13035	184.42266	V	1682.91466	841.96097	561.64307	16
5	622.29054	311.64891	208.10170	A	1583.84624	792.42676	528.62026	15
6	736.33347	368.67037	246.11601	N	1512.80912	756.90820	504.94122	14
7	837.38115	419.19421	279.79857	T	1398.76619	699.88673	466.92691	13
8	934.43392	467.72060	312.14949	P	1297.71851	649.36289	433.24435	12
9	1047.51799	524.26263	349.84418	L	1200.66574	600.83651	400.89343	11
10	1118.55511	559.78119	373.52322	A	1087.58167	544.29447	363.19874	10
11	1217.62353	609.31540	406.54603	V	1016.54455	508.77591	339.51970	9
12	1387.72906	694.36817	463.24787	K-Acetyl	917.47613	459.24170	306.49689	8
13	1458.76618	729.88673	486.92691	A	747.37060	374.18894	249.79505	7
14	1515.78765	758.39746	505.93407	G	676.33348	338.67038	226.11601	6
15	1618.79684	809.90206	540.27046	C	619.31201	310.15964	207.10885	5
16	1733.82379	867.41553	578.61278	D	516.30282	258.65505	172.77246	4
17	1832.89221	916.94974	611.63559	V	401.27587	201.14157	134.43014	3
18	1945.97628	973.49178	649.33028	L	302.20745	151.60736	101.40733	2
19				K-Acetyl	189.12338	95.06533	63.71264	1

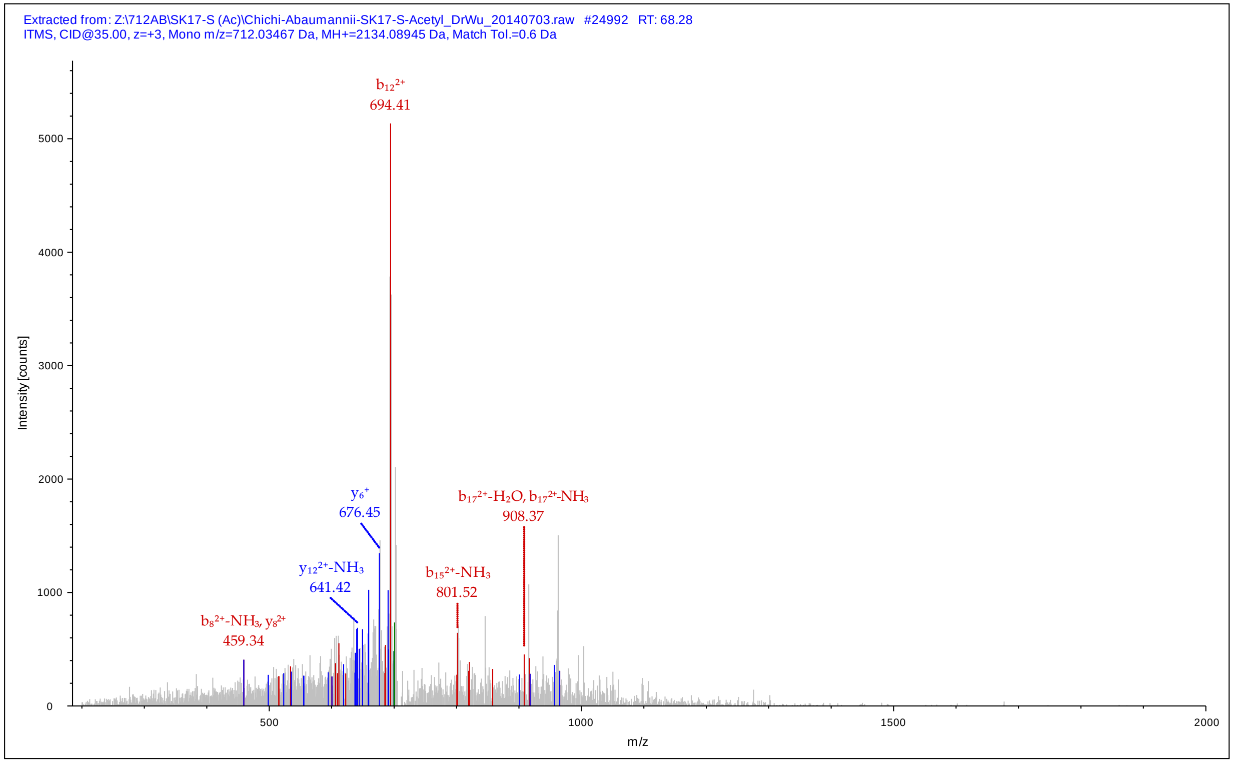

peg.2101 4222

#1	b+	b2+	Seq.	y+	y2+	#2
1	174.05834	87.53281	M-Acetyl			10
2	231.07981	116.04354	G	1111.68343	556.34535	9
3	359.13839	180.07283	Q	1054.66196	527.83462	8
4	472.22246	236.61487	L	926.60338	463.80533	7
5	642.32800	321.66764	K-Acetyl	813.51931	407.26329	6
6	755.41207	378.20967	I	643.41377	322.21052	5
7	868.49614	434.75171	L	530.32970	265.66849	4
8	997.53874	499.27301	E	417.24563	209.12645	3
9	1110.62281	555.81504	L	288.20303	144.60515	2
10			R	175.11896	88.06312	1

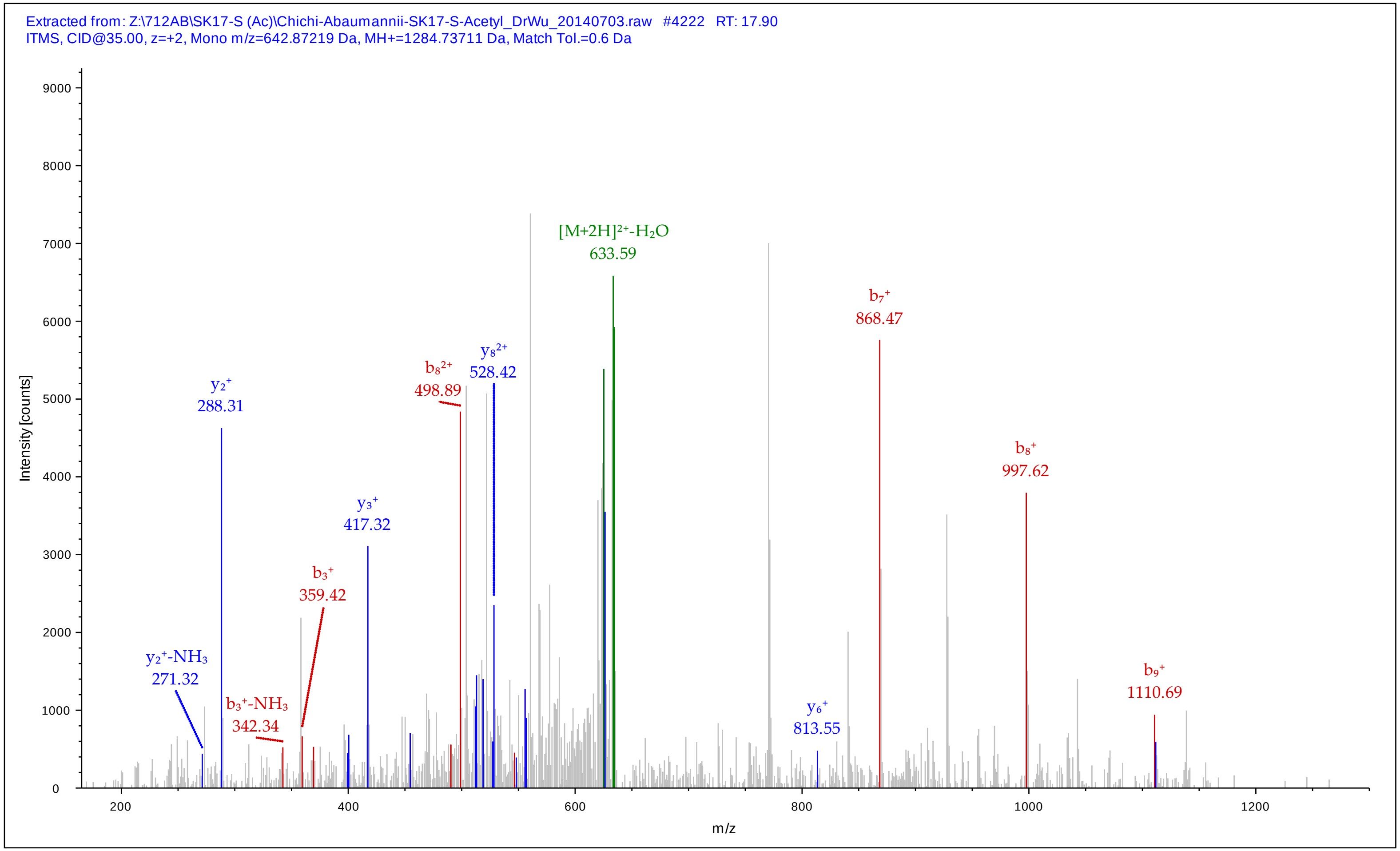

peg.2109   21062

#1	b^+	b^{2+}	Seq.	y^+	y^{2+}	#2
1	146.02703	73.51715	C-Acetyl			20
2	247.07471	124.04099	T	1931.00898	966.00813	19
3	417.18025	209.09376	K-Acetyl	1829.96130	915.48429	18
4	488.21737	244.61232	A	1659.85577	830.43152	17
5	559.25449	280.13088	A	1588.81865	794.91296	16
6	660.30217	330.65472	T	1517.78153	759.39440	15
7	731.33929	366.17328	A	1416.73385	708.87056	14
8	891.36994	446.18861	C-Carbam···	1345.69673	673.35200	13
9	948.39141	474.69934	G	1185.66607	593.33667	12
10	1005.41288	503.21008	G	1128.64460	564.82594	11
11	1108.42207	554.71467	C	1071.62313	536.31520	10
12	1179.45919	590.23323	A	968.61394	484.81061	9
13	1276.51196	638.75962	P	897.57682	449.29205	8
14	1389.59603	695.30165	L	800.52405	400.76566	7
15	1488.66445	744.83586	V	687.43998	344.22363	6
16	1589.71213	795.35970	T	588.37156	294.68942	5
17	1717.77071	859.38899	Q	487.32388	244.16558	4
18	1816.83913	908.92320	V	359.26530	180.13629	3
19	1929.92320	965.46524	L	260.19688	130.60208	2
20			K	147.11281	74.06004	1

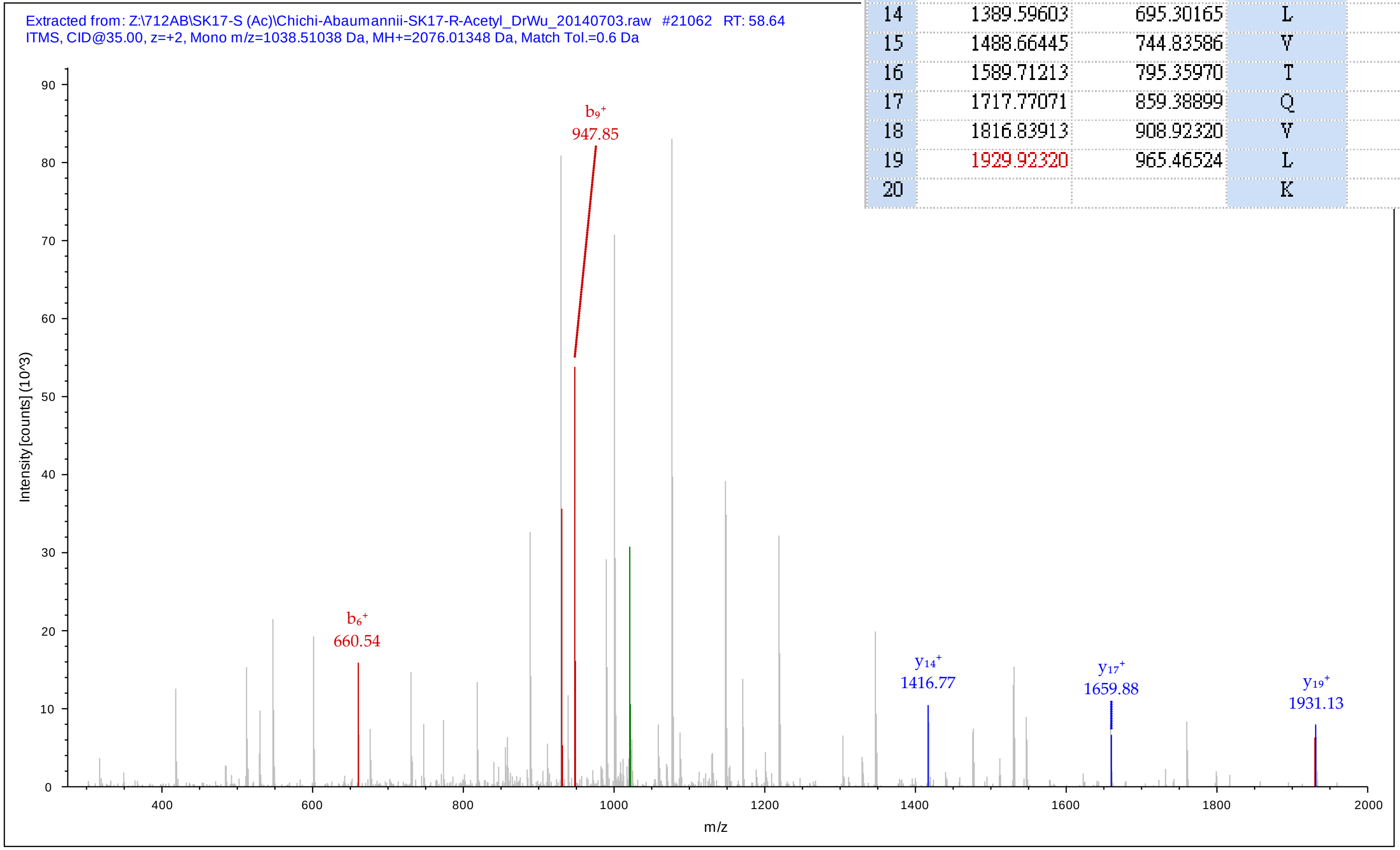

peg.2119 8658

#1	b+	b2+	b3+	Seq.	y+	y2+	y3+	#2
1	115.05021	58.02874	39.02159	N				15
2	228.13428	114.57078	76.71628	L	1733.91815	867.46271	578.64423	14
3	327.20270	164.10499	109.73908	V	1620.83408	810.92068	540.94954	13
4	456.24530	228.62629	152.75328	E	1521.76566	761.38647	507.92674	12
5	593.30421	297.15574	198.43959	H	1392.72306	696.86517	464.91254	11
6	722.34681	361.67704	241.45379	E	1255.66415	628.33571	419.22623	10
7	836.38974	418.69851	279.46810	N	1126.62155	563.81441	376.21203	9
8	965.43234	483.21981	322.48230	E	1012.57862	506.79295	338.19772	8
9	1062.48511	531.74619	354.83322	P	883.53602	442.27165	295.18352	7
10	1175.56918	588.28823	392.52791	L	786.48325	393.74526	262.83260	6
11	1345.67471	673.34099	449.22975	K-Acetyl	673.39918	337.20323	225.13791	5
12	1458.75878	729.88303	486.92444	L	503.29364	252.15046	168.43606	4
13	1545.79081	773.39904	515.93512	S	390.20957	195.60842	130.74137	3
14	1673.84939	837.42833	558.62131	Q	303.17754	152.09241	101.73070	2
15				R	175.11896	88.06312	59.04450	1

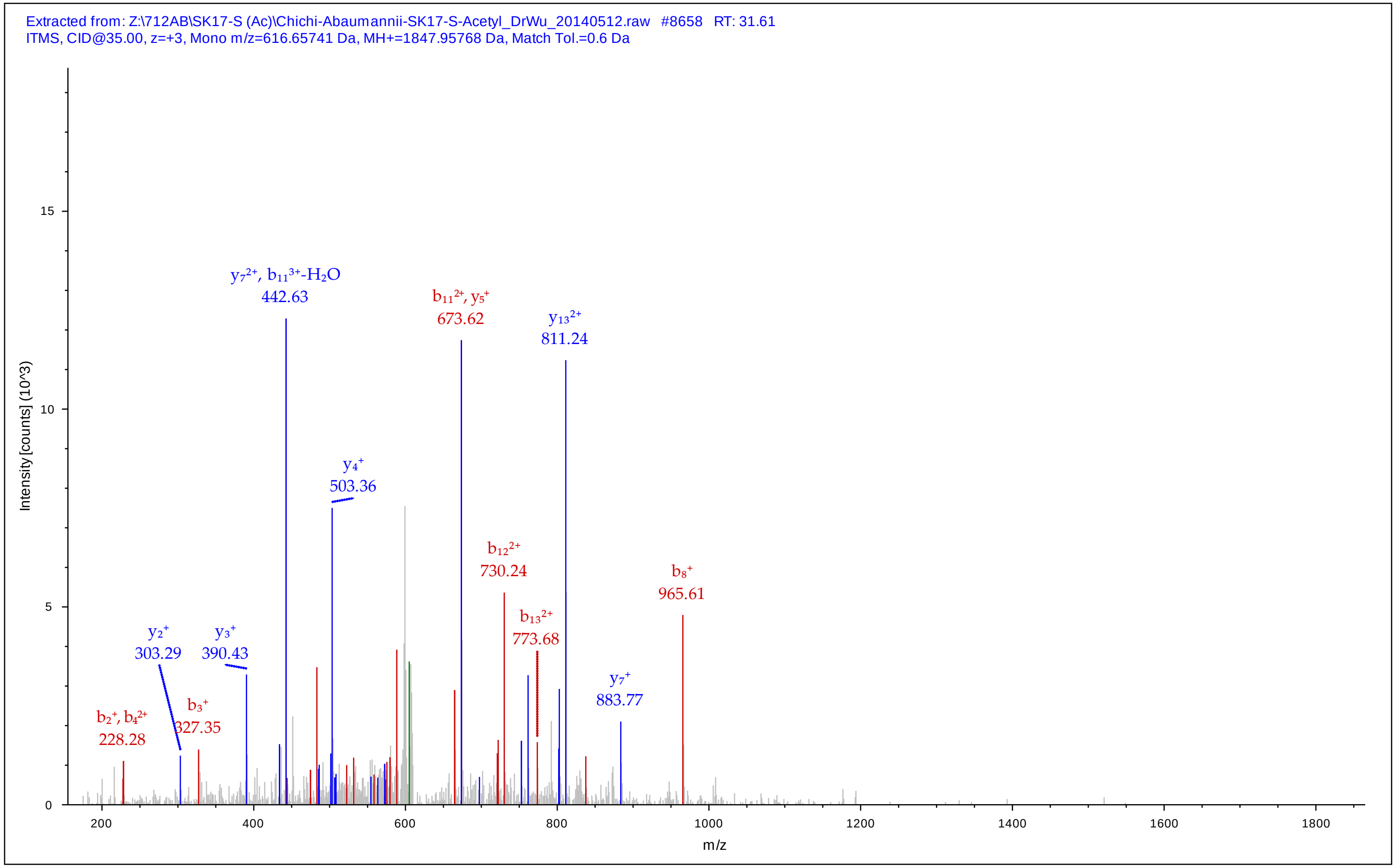

peg.2158   23501

#1	b^+	b^{2+}	Seq.	y^+	y^{2+}	#2
1	100.03931	50.52329	G-Acetyl			10
2	263.10263	132.05495	Y	1140.60483	570.80605	9
3	376.18670	188.59699	L	977.54151	489.27439	8
4	523.25512	262.13120	F	864.45744	432.73236	7
5	636.33919	318.67323	I	717.38902	359.19815	6
6	751.36614	376.18671	D	604.30495	302.65611	5
7	838.39817	419.70272	S	489.27800	245.14264	4
8	1008.50371	504.75549	K-Acetyl	402.24597	201.62662	3
9	1065.52518	533.26623	G	232.14043	116.57385	2
10			R	175.11896	88.06312	1

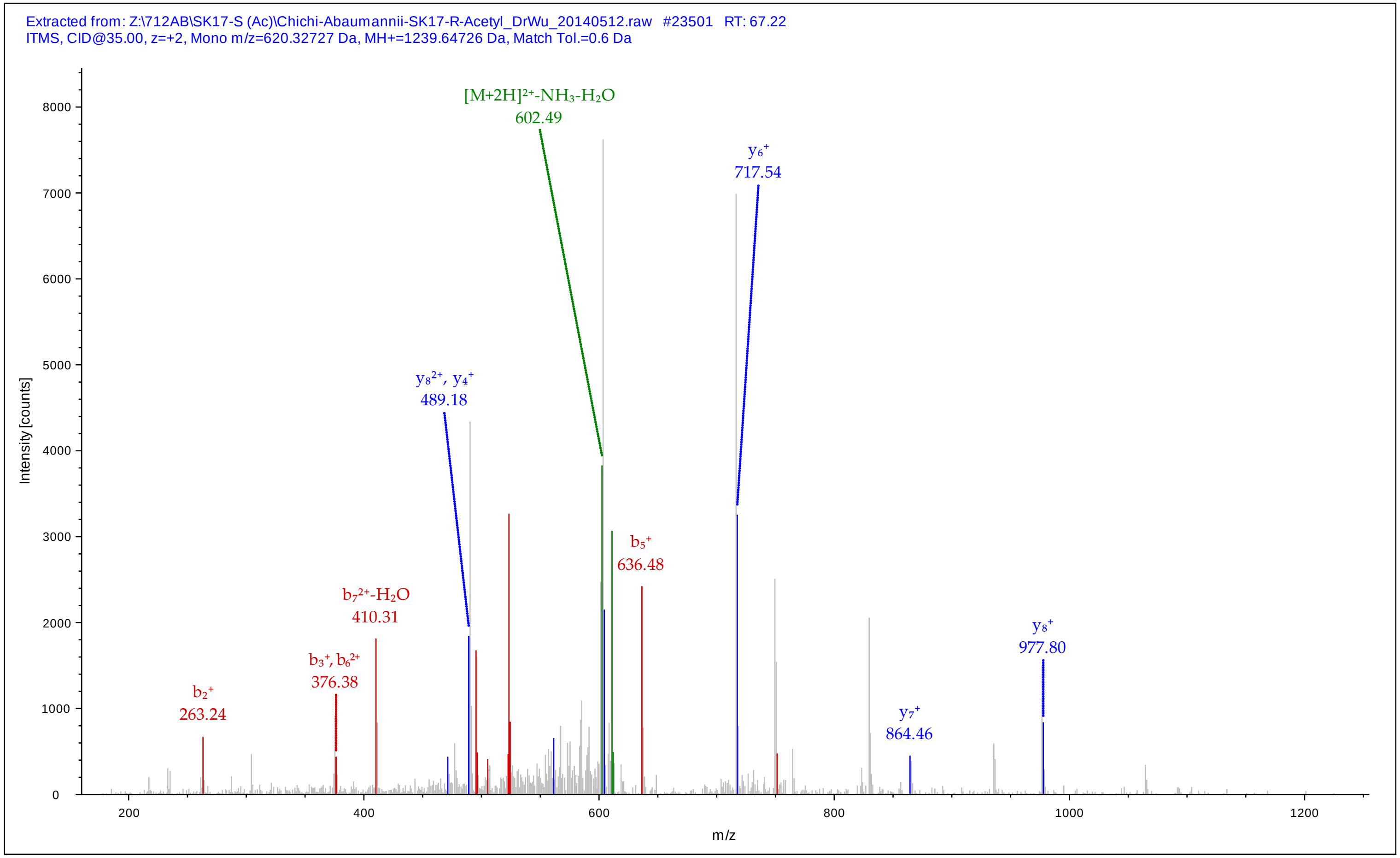

peg.2177 19089

#1	b⁺	b²⁺	b³⁺	Seq.	y⁺	y²⁺	y³⁺	#2
1	100.03931	50.52329	34.01795	G-Acetyl				24
2	228.09789	114.55258	76.70415	Q	2849.46155	1425.23441	950.49203	23
3	356.15647	178.58187	119.39034	Q	2721.40297	1361.20512	907.80584	22
4	469.24054	235.12391	157.08503	L	2593.34439	1297.17583	865.11965	21
5	583.28347	292.14537	195.09934	N	2480.26032	1240.63380	827.42496	20
6	711.34205	356.17466	237.78553	Q	2366.21739	1183.61233	789.41065	19
7	798.37408	399.69068	266.79621	S	2238.15881	1119.58304	746.72445	18
8	855.39555	428.20141	285.80337	G	2151.12678	1076.06703	717.71378	17
9	983.45413	492.23070	328.48956	Q	2094.10531	1047.55629	698.70662	16
10	1082.52255	541.76491	361.51237	V	1966.04673	983.52700	656.02043	15
11	1197.54950	599.27839	399.85468	D	1866.97831	933.99279	622.99762	14
12	1325.60808	663.30768	442.54088	Q	1751.95136	876.47932	584.65530	13
13	1424.67650	712.84189	475.56368	V	1623.89278	812.45003	541.96911	12
14	1552.77147	776.88937	518.26201	K	1524.82436	762.91582	508.94630	11
15	1699.83989	850.42358	567.28481	F	1396.72939	698.86833	466.24798	10
16	1800.88757	900.94742	600.96737	T	1249.66097	625.33412	417.22517	9
17	1928.94615	964.97671	643.65357	Q	1148.61329	574.81028	383.54261	8
18	2088.97681	1044.99204	696.99712	C-Carbam···	1020.55471	510.78099	340.85642	7
19	2202.06088	1101.53408	734.69181	L	860.52405	430.76566	287.51287	6
20	2330.11946	1165.56337	777.37800	Q	747.43998	374.22263	249.81818	5
21	2477.18788	1239.09758	826.40081	F	619.38140	310.19434	207.13198	4
22	2590.27195	1295.63961	864.09550	I	472.31298	236.66013	158.10918	3
23	2760.37748	1380.69238	920.79734	K-Acetyl	359.22891	180.11809	120.41449	2
24				K-Acetyl	189.12338	95.06533	63.71264	1

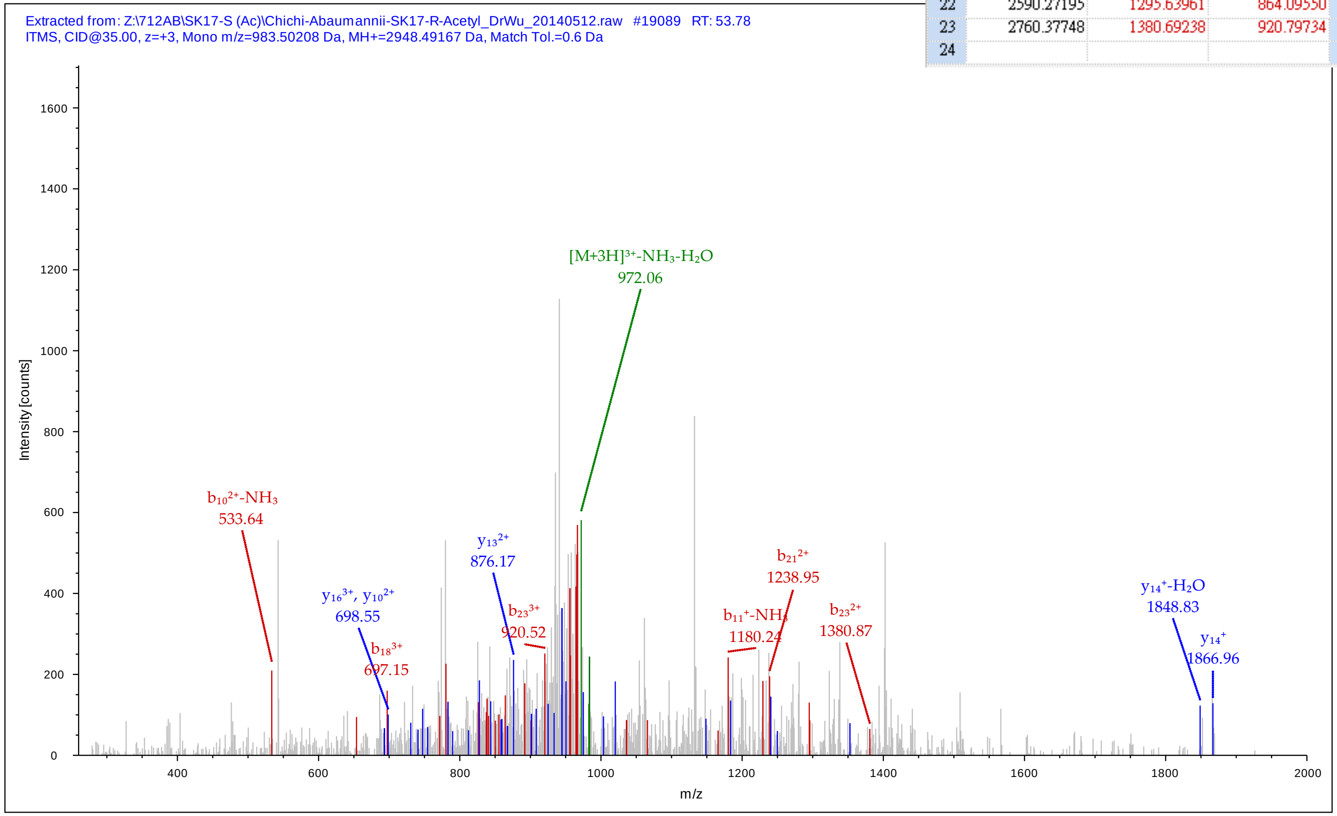

peg.218 19219

#1	b+	b2+	b3+	Seq.	y+	y2+	y3+	#2
1	100.07570	50.54149	34.03008	V				25
2	171.11282	86.06005	57.70912	A	2912.34039	1456.67383	971.45165	24
3	284.19689	142.60208	95.40381	L	2841.30327	1421.15527	947.77261	23
4	412.25547	206.63137	138.09001	Q	2728.21920	1364.61324	910.07792	22
5	541.29807	271.15267	181.10421	E	2600.16062	1300.58395	867.39172	21
6	598.31954	299.66341	200.11136	G	2471.11802	1236.06265	824.37752	20
7	727.36214	364.18471	243.12556	E	2414.09655	1207.55191	805.37037	19
8	840.44621	420.72674	280.82025	I	2285.05395	1143.03061	762.35617	18
9	953.53028	477.26878	318.51494	I	2171.96988	1086.48858	724.66148	17
10	1068.55723	534.78225	356.85726	D	2058.88581	1029.94654	686.96679	16
11	1155.58926	578.29827	385.86794	S	1943.85886	972.43307	648.62447	15
12	1302.62467	651.81597	434.87974	M-Oxidation	1856.82683	928.91705	619.61379	14
13	1449.69309	725.35018	483.90255	F	1709.79142	855.39935	570.60199	13
14	1596.72851	798.86789	532.91435	M-Oxidation	1562.72300	781.86514	521.57918	12
15	1683.76054	842.38391	561.92503	S	1415.68758	708.34743	472.56738	11
16	1853.86607	927.43667	618.62687	K-Acetyl	1328.65555	664.83141	443.55670	10
17	2023.97161	1012.48944	675.32872	K-Acetyl	1158.55002	579.77865	386.85486	9
18	2095.00873	1048.00800	699.00776	A	988.44448	494.72588	330.15301	8
19	2208.09280	1104.55004	736.70245	L	917.40736	459.20732	306.47397	7
20	2311.10199	1156.05463	771.03885	C	804.32329	402.66528	268.77928	6
21	2426.12894	1213.56811	809.38116	D	701.31410	351.16069	234.44288	5
22	2573.19736	1287.10232	858.40397	F	586.28715	293.64721	196.10057	4
23	2736.26068	1368.63398	912.75841	Y	439.21873	220.11300	147.07776	3
24	2865.30328	1433.15528	955.77261	E	276.15541	138.58134	92.72332	2
25				K	147.11281	74.06004	49.70912	1

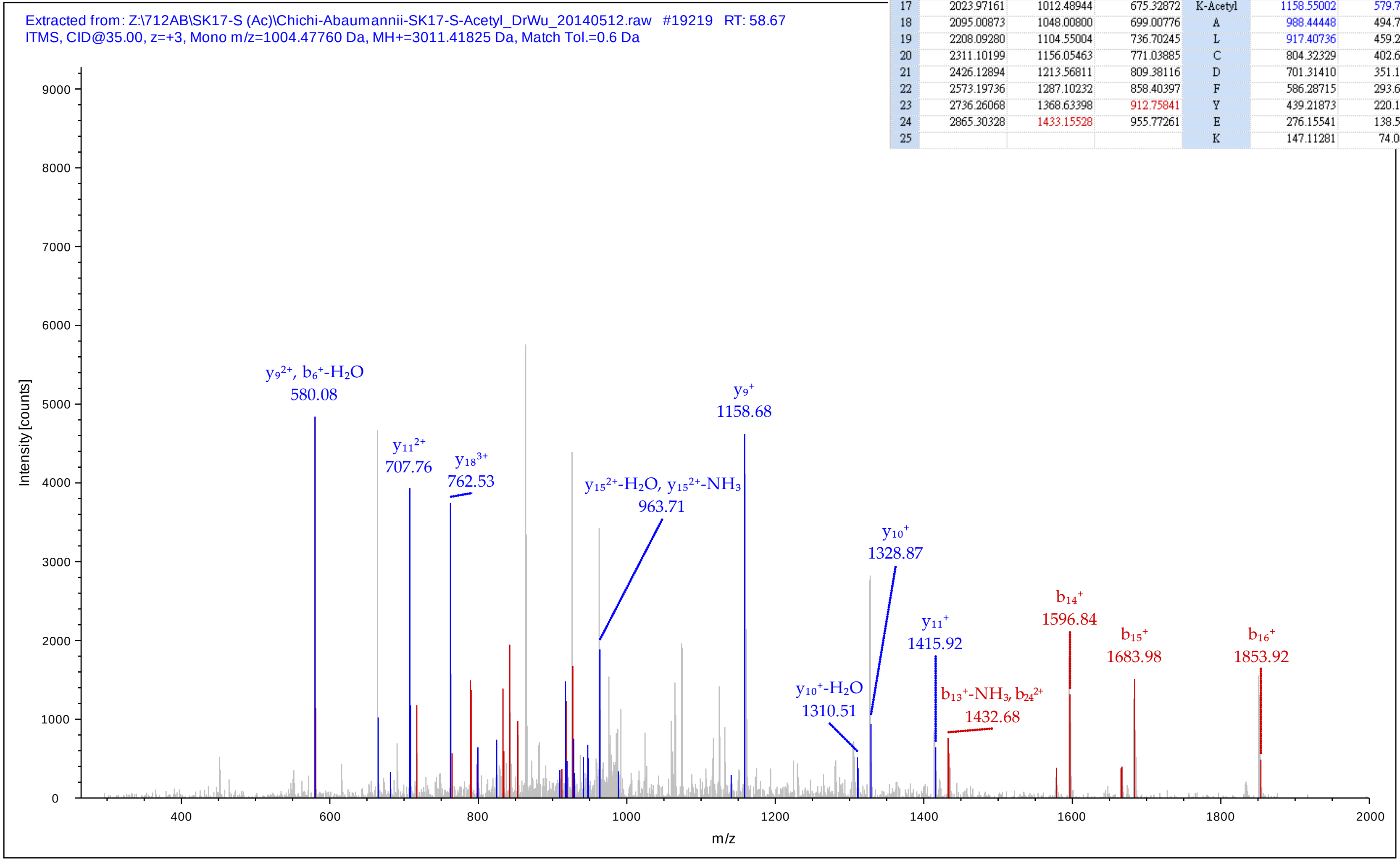

peg.2229   9120

#1	b⁺	b²⁺	b³⁺	Seq.	y⁺	y²⁺	y³⁺	#2
1	129.06586	65.03657	43.69347	Q				10
2	258.10846	129.55787	86.70767	E	1099.68343	550.34535	367.23266	9
3	428.21399	214.61063	143.40951	K-Acetyl	970.64083	485.82405	324.21846	8
4	499.25111	250.12919	167.08855	A	800.53529	400.77128	267.51661	7
5	586.28314	293.64521	196.09923	S	729.49817	365.25272	243.83757	6
6	742.38426	371.69577	248.13294	R	642.46614	321.73671	214.82690	5
7	855.46833	428.23780	285.82763	L	486.36502	243.68615	162.79319	4
8	968.55240	484.77984	323.52232	L	373.28095	187.14411	125.09850	3
9	1081.63647	541.32187	361.21701	L	260.19688	130.60208	87.40381	2
10				K	147.11281	74.06004	49.70912	1

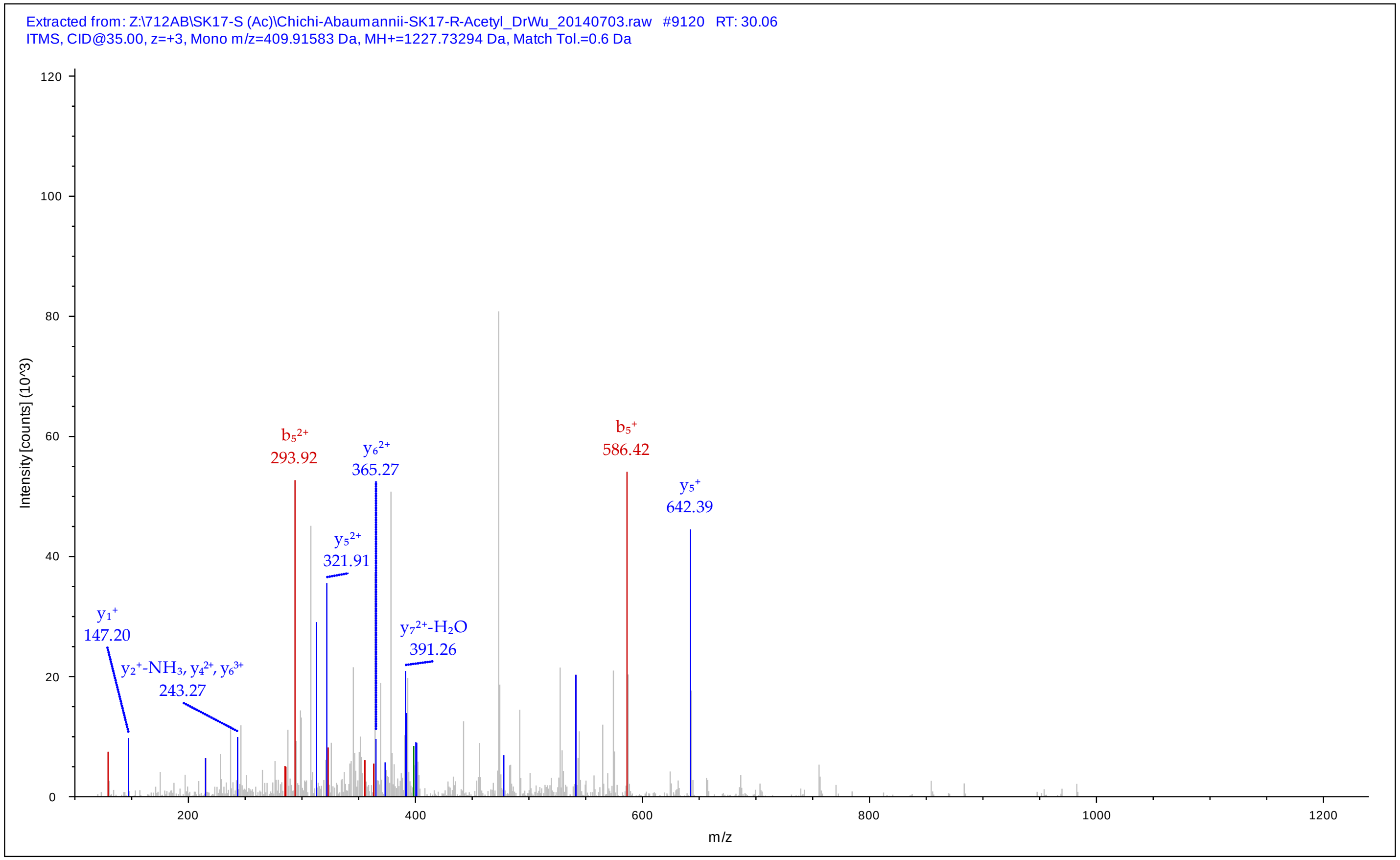

peg.2230 16501

#1	b^+	b^{2+}	Seq.	y^+	y^{2+}	#2
1	171.11281	86.06004	K-Acetyl			15
2	228.13428	114.57078	G	1529.72652	765.36690	14
3	315.16631	158.08679	S	1472.70505	736.85616	13
4	386.20343	193.60535	A	1385.67302	693.34015	12
5	500.24636	250.62682	N	1314.63590	657.82159	11
6	629.28896	315.14812	E	1200.59297	600.80012	10
7	743.33189	372.16958	N	1071.55037	536.27882	9
8	871.39047	436.19887	Q	957.50744	479.25736	8
9	970.45889	485.73308	V	829.44886	415.22807	7
10	1083.54296	542.27512	L	730.38044	365.69386	6
11	1230.57838	615.79283	M-Oxidation	617.29637	309.15182	5
12	1327.63115	664.31921	P	470.26095	235.63411	4
13	1456.67375	728.84051	E	373.20818	187.10773	3
14	1553.72652	777.36690	P	244.16558	122.58643	2
15			K	147.11281	74.06004	1

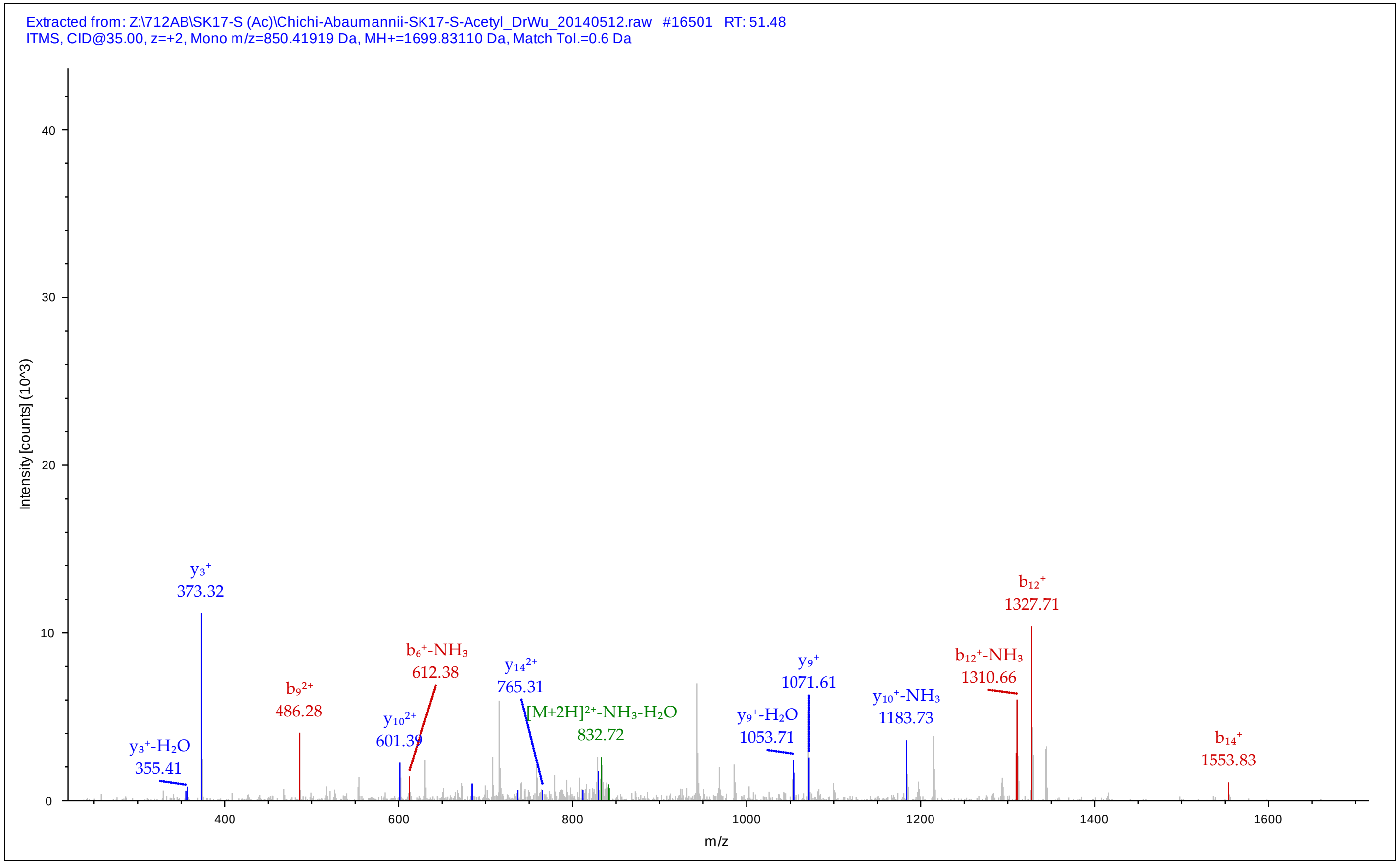

peg.2241 7323

#1	b^+	b^{2+}	Seq.	y^+	y^{2+}	#2
1	172.06044	86.53386	E-Acetyl			13
2	287.08739	144.04733	D	1372.73325	686.87026	12
3	415.14597	208.07662	Q	1257.70630	629.35679	11
4	472.16744	236.58736	G	1129.64772	565.32750	10
5	569.22021	285.11374	P	1072.62625	536.81676	9
6	739.32575	370.16651	K-Acetyl	975.57348	488.29038	8
7	810.36287	405.68507	A	805.46794	403.23761	7
8	957.43129	479.21928	F	734.43082	367.71905	6
9	1014.45276	507.73002	G	587.36240	294.18484	5
10	1142.54773	571.77750	K	530.34093	265.67410	4
11	1255.63180	628.31954	L	402.24596	201.62662	3
12	1369.67473	685.34100	N	289.16189	145.08458	2
13			R	175.11896	88.06312	1

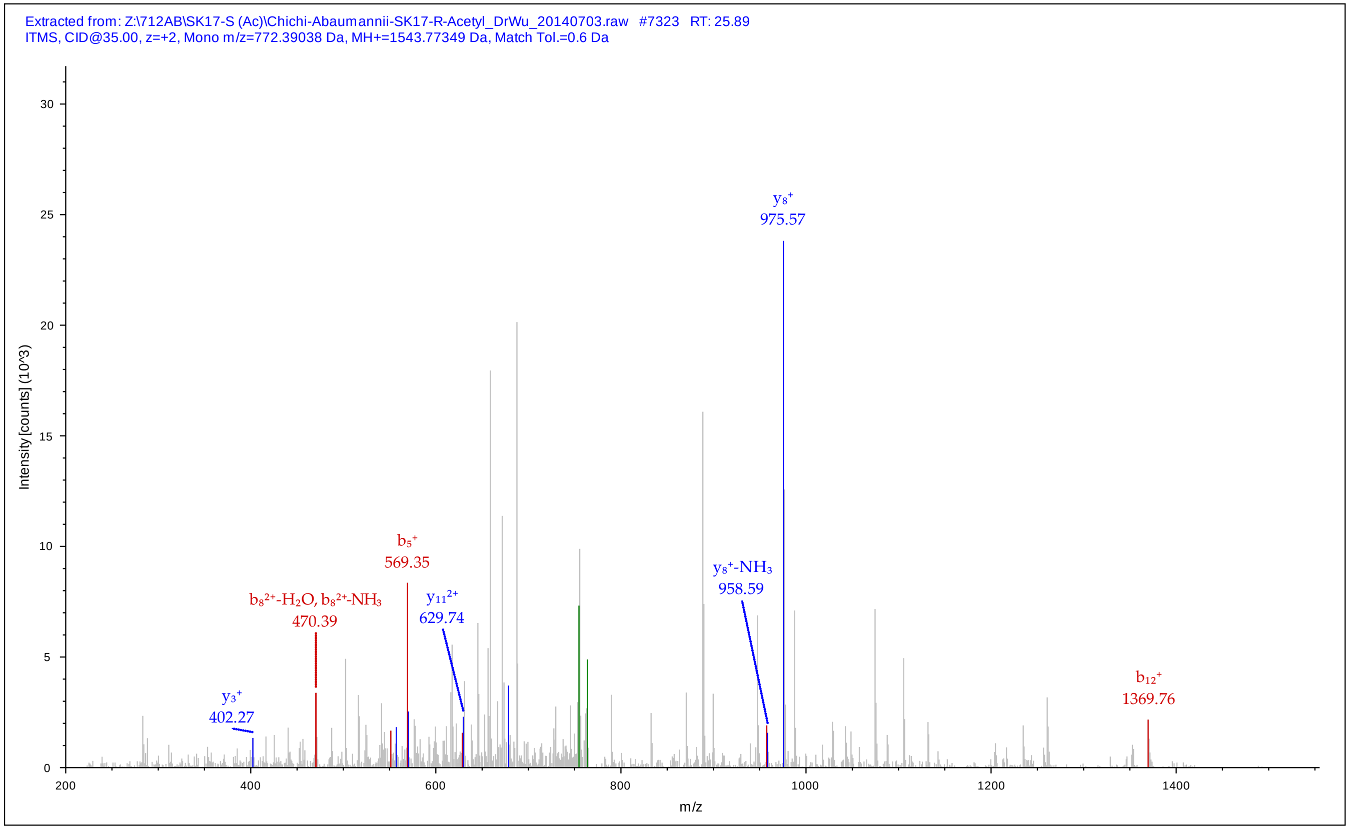

peg.2241   8765

#1	b⁺	b²⁺	Seq.	y⁺	y²⁺	#2
1	172.06044	86.53386	E-Acetyl			13
2	287.08739	144.04733	D	1372.73325	686.87026	12
3	415.14597	208.07662	Q	1257.70630	629.35679	11
4	472.16744	236.58736	G	1129.64772	565.32750	10
5	569.22021	285.11374	P	1072.62625	536.81676	9
6	739.32575	370.16651	K-Acetyl	975.57348	488.29038	8
7	810.36287	405.68507	A	805.46794	403.23761	7
8	957.43129	479.21928	F	734.43082	367.71905	6
9	1014.45276	507.73002	G	587.36240	294.18484	5
10	1142.54773	571.77750	K	530.34093	265.67410	4
11	1255.63180	628.31954	L	402.24596	201.62662	3
12	1369.67473	685.34100	N	289.16189	145.08458	2
13			R	175.11896	88.06312	1

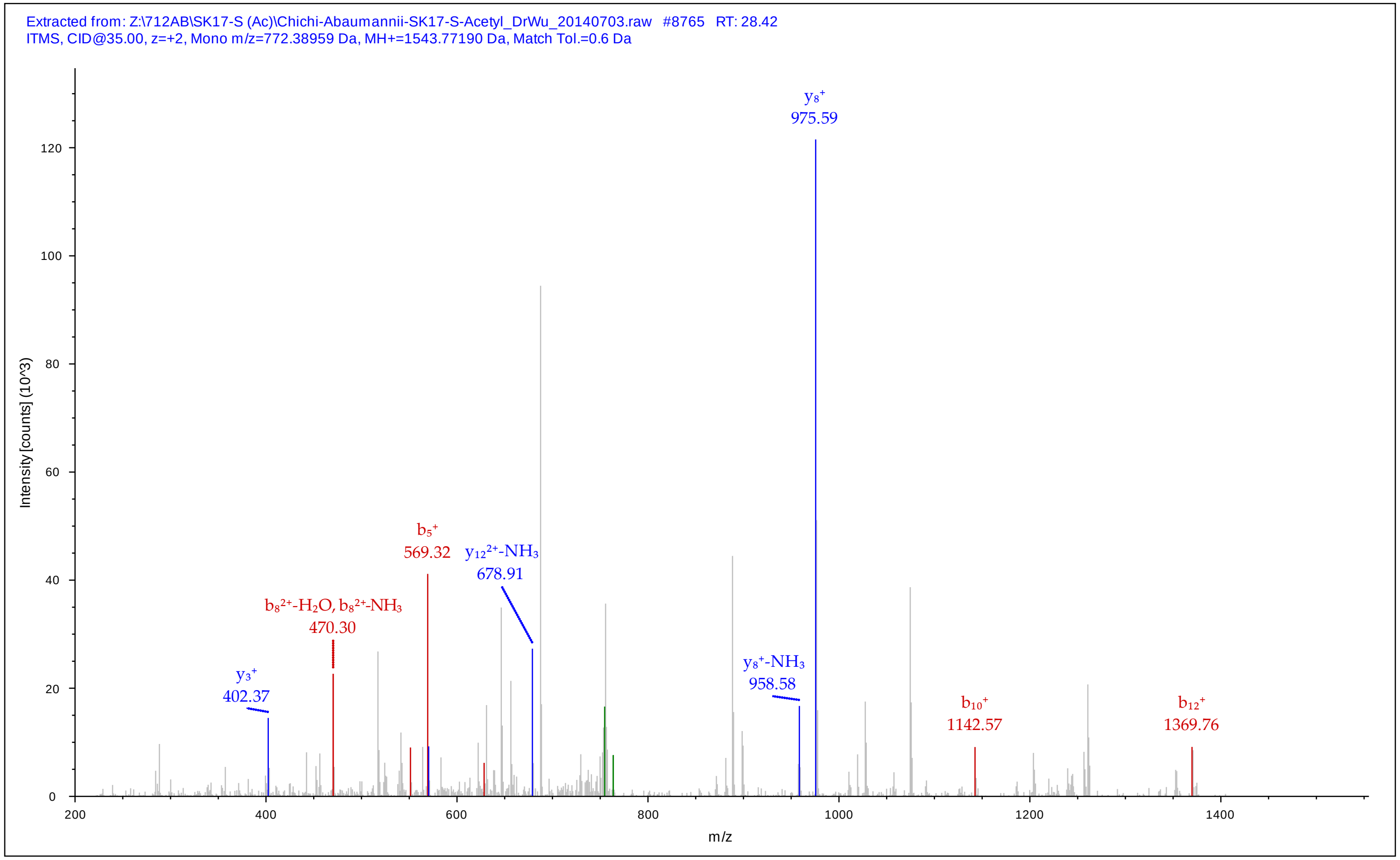

peg.2241 7590

#1	b⁺	b²⁺	Seq.	y⁺	y²⁺	#2
1	172.06044	86.53386	E-Acetyl			13
2	287.08739	144.04733	D	1372.73325	686.87026	12
3	415.14597	208.07662	Q	1257.70630	629.35679	11
4	472.16744	236.58736	G	1129.64772	565.32750	10
5	569.22021	285.11374	P	1072.62625	536.81676	9
6	697.31518	349.16123	K	975.57348	488.29038	8
7	768.35230	384.67979	A	847.47851	424.24289	7
8	915.42072	458.21400	F	776.44139	388.72433	6
9	972.44219	486.72473	G	629.37297	315.19012	5
10	1142.54773	571.77750	K-Acetyl	572.35150	286.67939	4
11	1255.63180	628.31954	L	402.24596	201.62662	3
12	1369.67473	685.34100	N	289.16189	145.08458	2
13			R	175.11896	88.06312	1

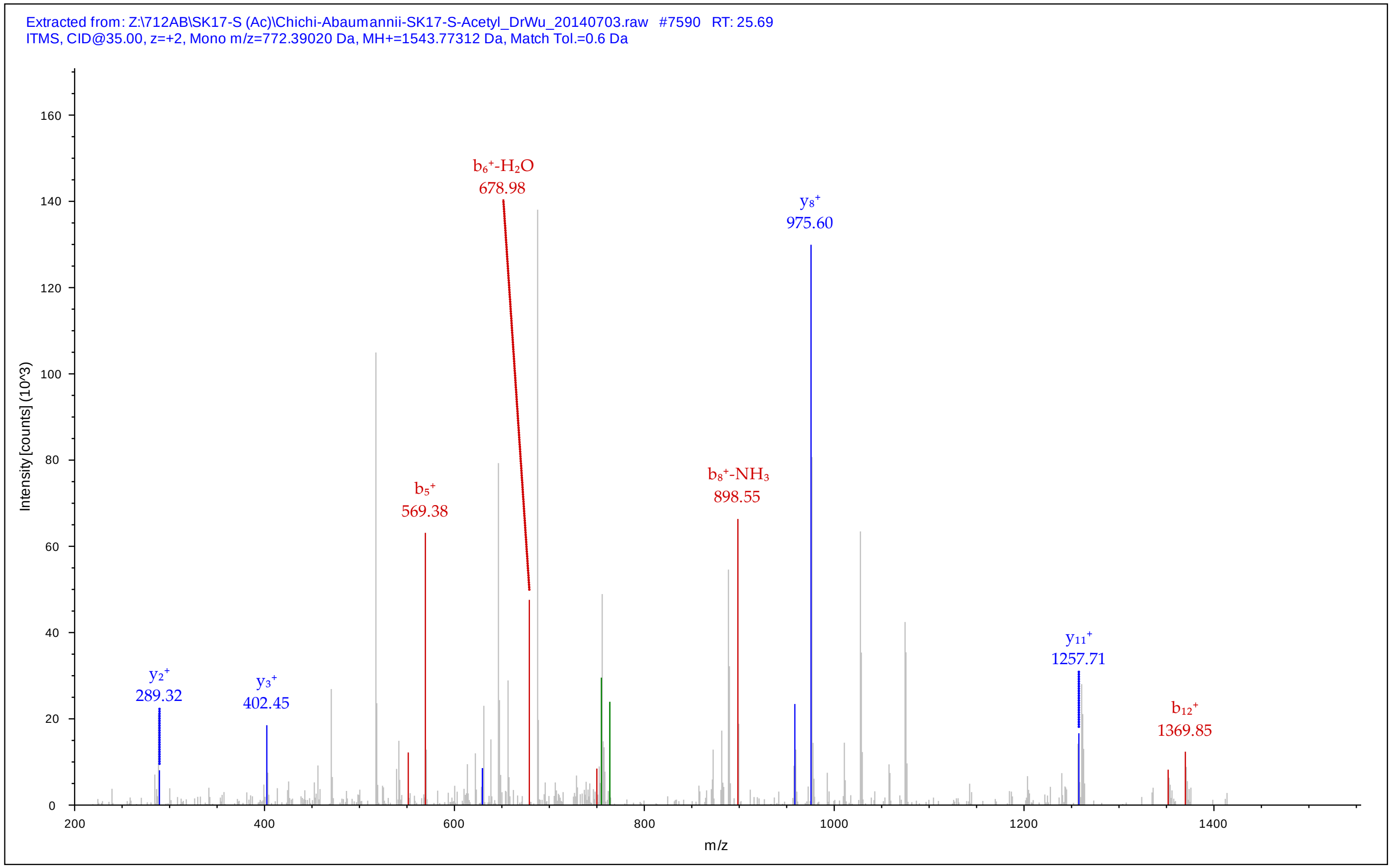

peg.227 14212

#1	b+	b2+	Seq.	y+	y2+	#2
1	157.06077	79.03402	N-Acetyl			12
2	271.10370	136.05549	N	1349.70600	675.35664	11
3	399.16228	200.08478	Q	1235.66307	618.33517	10
4	562.22560	281.61644	Y	1107.60449	554.30588	9
5	649.25763	325.13245	S	944.54117	472.77422	8
6	763.30056	382.15392	N	857.50914	429.25821	7
7	876.38463	438.69595	L	743.46621	372.23674	6
8	1046.49017	523.74872	K-Acetyl	630.38214	315.69471	5
9	1117.52729	559.26728	A	460.27660	230.64194	4
10	1230.61136	615.80932	L	389.23948	195.12338	3
11	1359.65396	680.33062	E	276.15541	138.58134	2
12			K	147.11281	74.06004	1

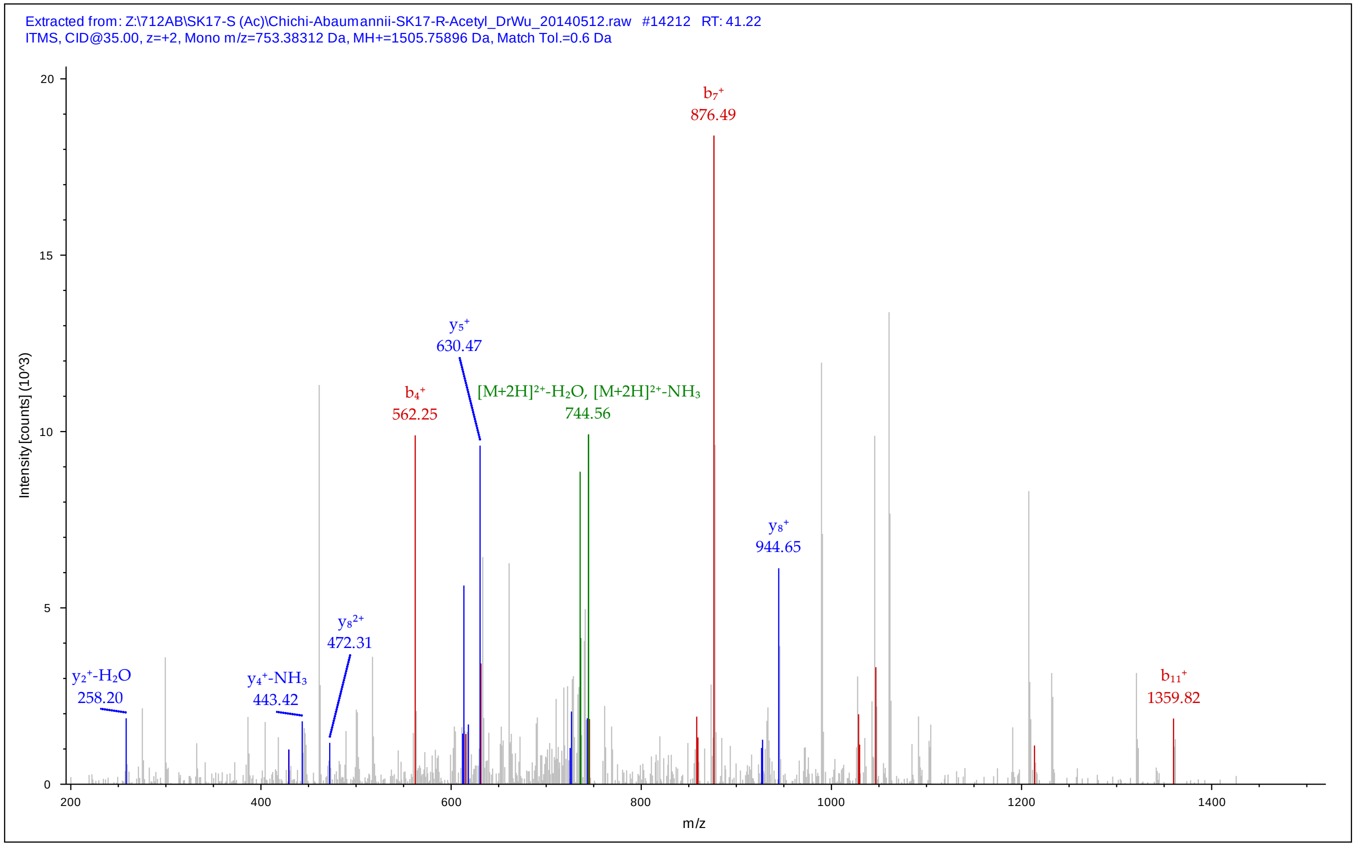

peg.227 12534

#1	b+	b2+	Seq.	y+	y2+	#2
1	157.06077	79.03402	N-Acetyl			12
2	271.10370	136.05549	N	1349.70600	675.35664	11
3	399.16228	200.08478	Q	1235.66307	618.33517	10
4	562.22560	281.61644	Y	1107.60449	554.30588	9
5	649.25763	325.13245	S	944.54117	472.77422	8
6	763.30056	382.15392	N	857.50914	429.25821	7
7	876.38463	438.69595	L	743.46621	372.23674	6
8	1046.49017	523.74872	K-Acetyl	630.38214	315.69471	5
9	1117.52729	559.26728	A	460.27660	230.64194	4
10	1230.61136	615.80932	L	389.23948	195.12338	3
11	1359.65396	680.33062	E	276.15541	138.58134	2
12			K	147.11281	74.06004	1

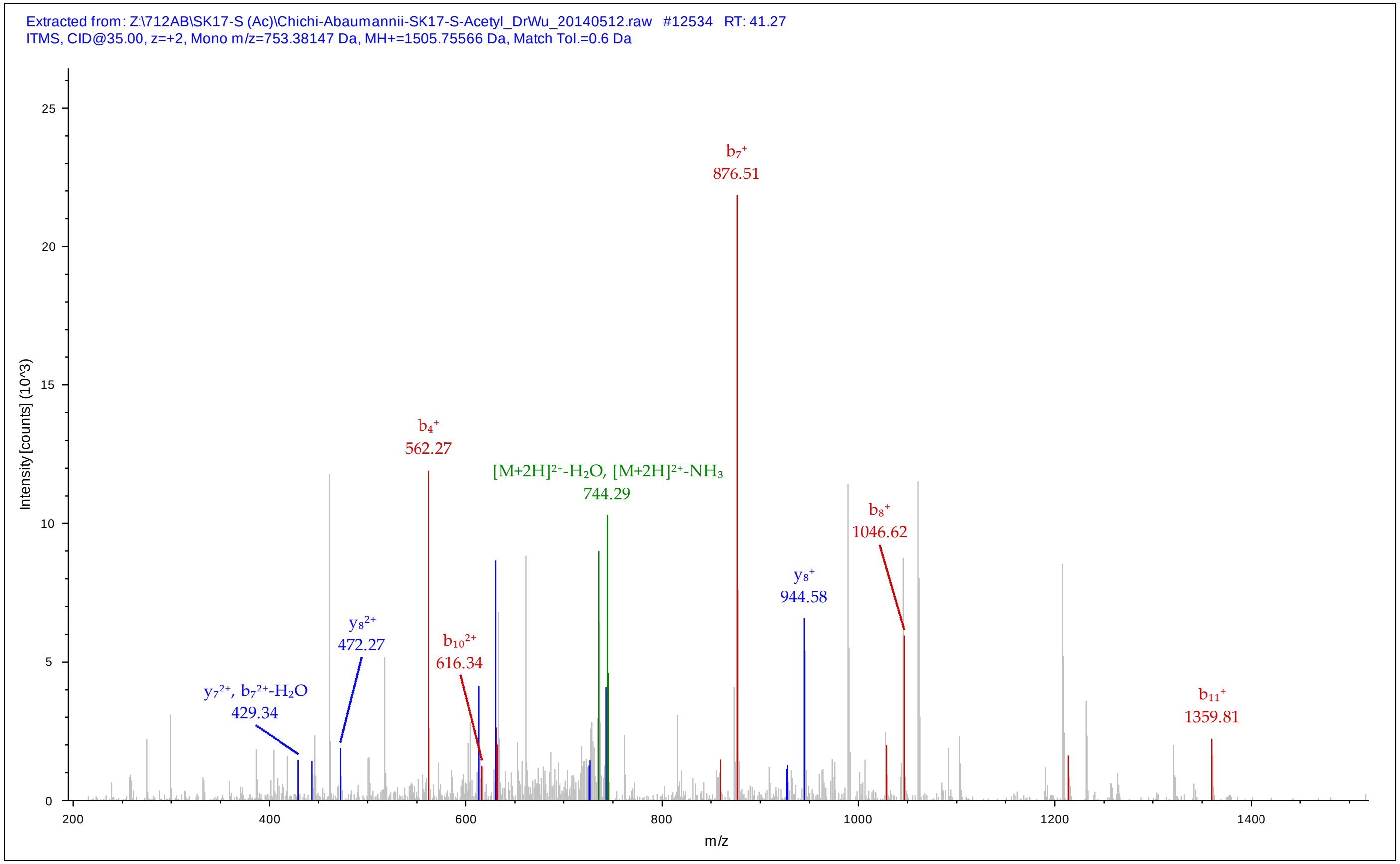

peg.2279 9452

#1	b⁺	b²⁺	Seq.	y⁺	y²⁺	#2
1	171.11281	86.06004	K-Acetyl			10
2	327.21393	164.11060	R	987.55822	494.28275	9
3	384.23540	192.62134	G	831.45710	416.23219	8
4	471.26743	236.13735	S	774.43563	387.72145	7
5	570.33585	285.67156	V	687.40360	344.20544	6
6	657.36788	329.18758	S	588.33518	294.67123	5
7	786.41048	393.70888	E	501.30315	251.15521	4
8	914.50545	457.75636	K	372.26055	186.63391	3
9	1011.55822	506.28275	P	244.16558	122.58643	2
10			K	147.11281	74.06004	1

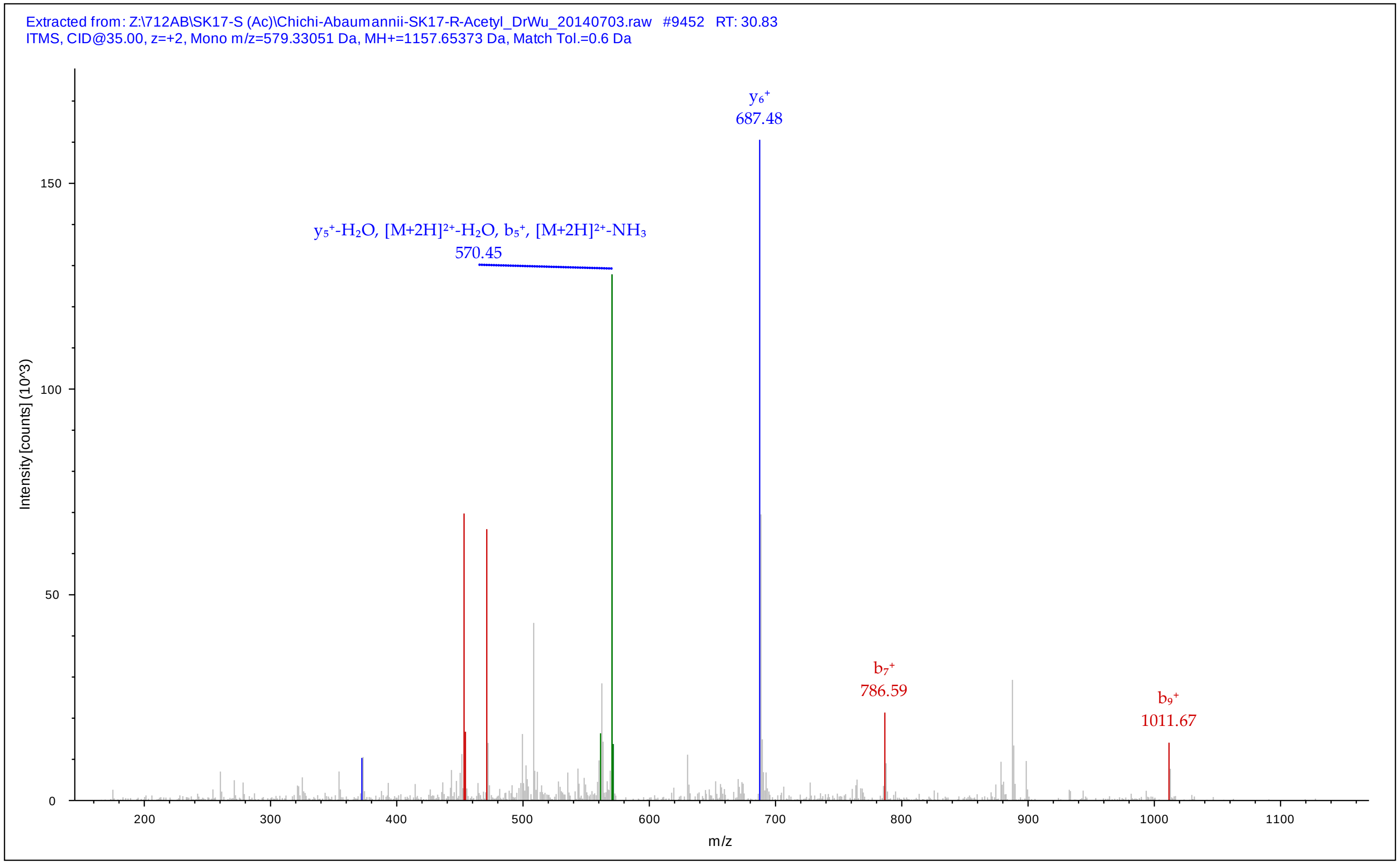

peg.2279   8667

#1	b⁺	b²⁺	Seq.	y⁺	y²⁺	#2
1	171.11281	86.06004	K-Acetyl			10
2	327.21393	164.11060	R	987.55822	494.28275	9
3	384.23540	192.62134	G	831.45710	416.23219	8
4	471.26743	236.13735	S	774.43563	387.72145	7
5	570.33585	285.67156	V	687.40360	344.20544	6
6	657.36788	329.18758	S	588.33518	294.67123	5
7	786.41048	393.70888	E	501.30315	251.15521	4
8	914.50545	457.75636	K	372.26055	186.63391	3
9	1011.55822	506.28275	P	244.16558	122.58643	2
10			K	147.11281	74.06004	1

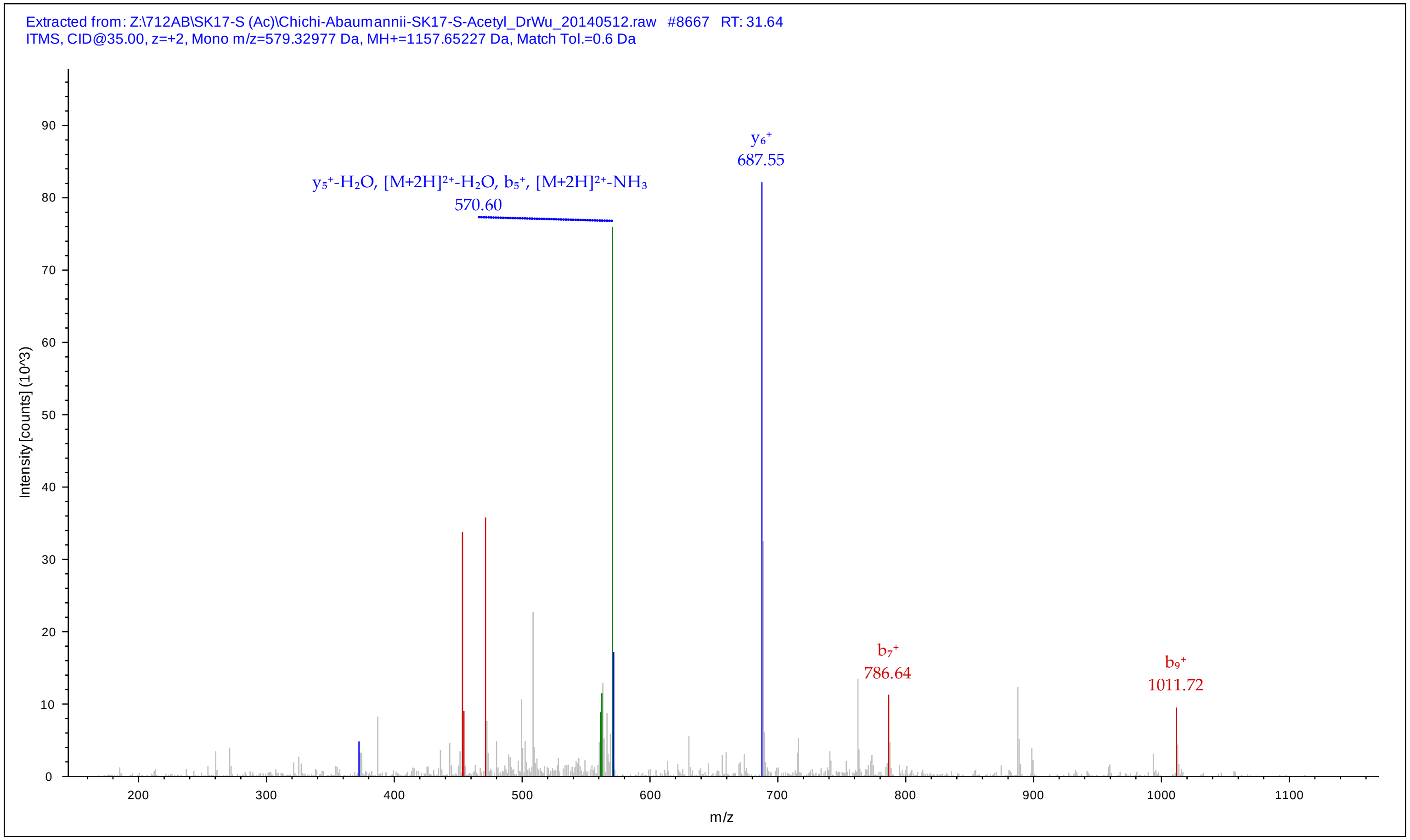

peg.229 13074

#1	b⁺	b²⁺	Seq.	y⁺	y²⁺	#2
1	164.07060	82.53894	Y			8
2	292.12918	146.56823	Q	843.50472	422.25600	7
3	420.18776	210.59752	Q	715.44614	358.22671	6
4	491.22488	246.11608	A	587.38756	294.19742	5
5	619.31985	310.16356	K	516.35044	258.67886	4
6	789.42538	395.21633	K-Acetyl	388.25547	194.63137	3
7	860.46250	430.73489	A	218.14993	109.57860	2
8			K	147.11281	74.06004	1

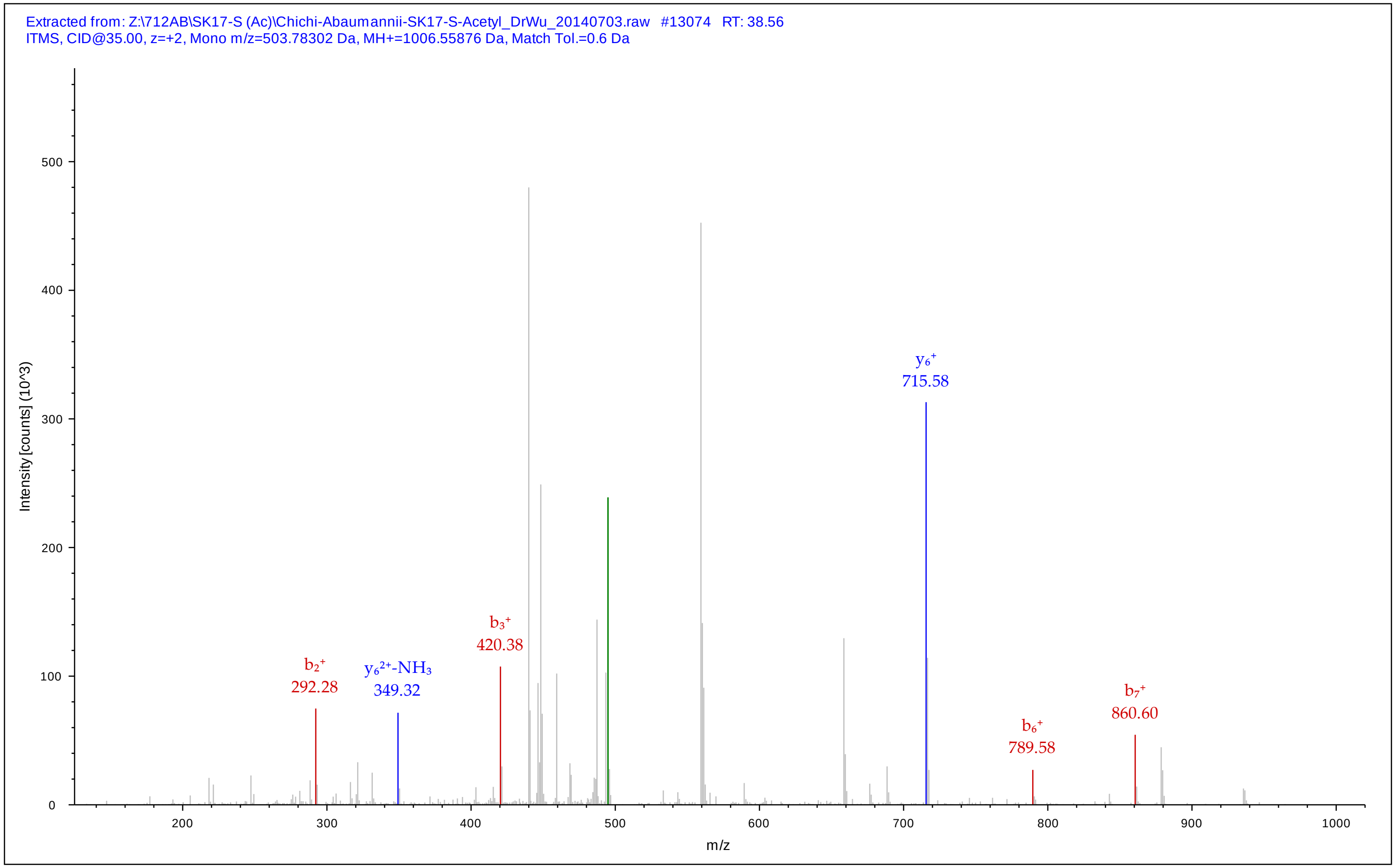

peg.229   26428

#1	b⁺	b²⁺	b³⁺	Seq.	y⁺	y²⁺	y³⁺	#2
1	114.09135	57.54931	38.70197	I				28
2	229.11830	115.06279	77.04428	D	3295.49975	1648.25351	1099.17143	27
3	316.15033	158.57880	106.05496	S	3180.47280	1590.74004	1060.82912	26
4	417.19801	209.10264	139.73752	T	3093.44077	1547.22402	1031.81844	25
5	603.27733	302.14230	201.76396	W	2992.39309	1496.70018	998.13588	24
6	766.34065	383.67396	256.11840	Y	2806.31377	1403.66052	936.10944	23
7	913.37606	457.19167	305.13020	M-Oxidation	2643.25045	1322.12886	881.75500	22
8	1026.46013	513.73370	342.82489	I	2496.21503	1248.61115	832.74319	21
9	1123.51290	562.26009	375.17582	P	2383.13096	1192.06912	795.04850	20
10	1252.55550	626.78139	418.19002	E	2286.07819	1143.54273	762.69758	19
11	1339.58753	670.29740	447.20069	S	2157.03559	1079.02143	719.68338	18
12	1426.61956	713.81342	476.21137	S	2070.00356	1035.50542	690.67270	17
13	1497.65668	749.33198	499.89041	A	1982.97153	991.98940	661.66203	16
14	1611.69961	806.35344	537.90472	N	1911.93441	956.47084	637.98299	15
15	1781.80515	891.40621	594.60657	K-Acetyl	1797.89148	899.44938	599.96868	14
16	1882.85283	941.93005	628.28913	T	1627.78595	814.39661	543.26683	13
17	1995.93690	998.47209	665.98382	I	1526.73827	763.87277	509.58427	12
18	2124.97950	1062.99339	708.99802	E	1413.65420	707.33074	471.88958	11
19	2238.06357	1119.53542	746.69271	L	1284.61160	642.80944	428.87538	10
20	2424.14289	1212.57508	808.71915	W	1171.52753	586.26740	391.18069	9
21	2552.23786	1276.62257	851.41747	K	985.44821	493.22774	329.15425	8
22	2655.24705	1328.12716	885.75387	C	857.35324	429.18026	286.45593	7
23	2756.29473	1378.65100	919.43643	T	754.34405	377.67566	252.11953	6
24	2871.32168	1436.16448	957.77874	D	653.29637	327.15182	218.43697	5
25	3018.39010	1509.69869	1006.80155	F	538.26942	269.63835	180.09466	4
26	3115.44287	1558.22507	1039.15247	P	391.20100	196.10414	131.07185	3
27	3262.47828	1631.74278	1088.16428	M-Oxidation	294.14823	147.57775	98.72093	2
28				K	147.11281	74.06004	49.70912	1

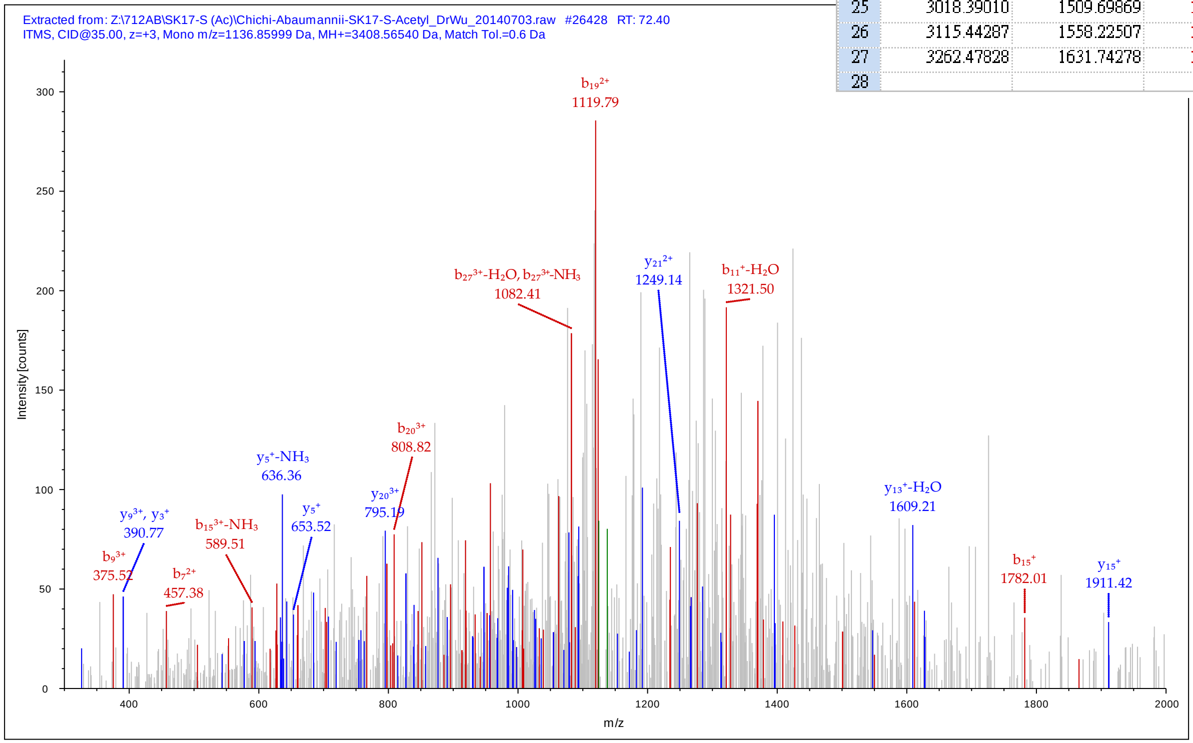

peg.2332 19099

#1	b^+	b^{2+}	Seq.	y^+	y^{2+}	#2
1	157.10840	79.05784	R			14
2	228.14552	114.57640	A	1440.78060	720.89394	13
3	343.17247	172.08987	D	1369.74348	685.37538	12
4	457.21540	229.11134	N	1254.71653	627.86190	11
5	572.24235	286.62481	D	1140.67360	570.84044	10
6	685.32642	343.16685	I	1025.64665	513.32696	9
7	756.36354	378.68541	A	912.56258	456.78493	8
8	869.44761	435.22744	I	841.52546	421.26637	7
9	940.48473	470.74600	A	728.44139	364.72433	6
10	1110.59026	555.79877	K-Acetyl	657.40427	329.20577	5
11	1238.64884	619.82806	Q	487.29873	244.15300	4
12	1309.68596	655.34662	A	359.24015	180.12371	3
13	1422.77003	711.88865	L	288.20303	144.60515	2
14			R	175.11896	88.06312	1

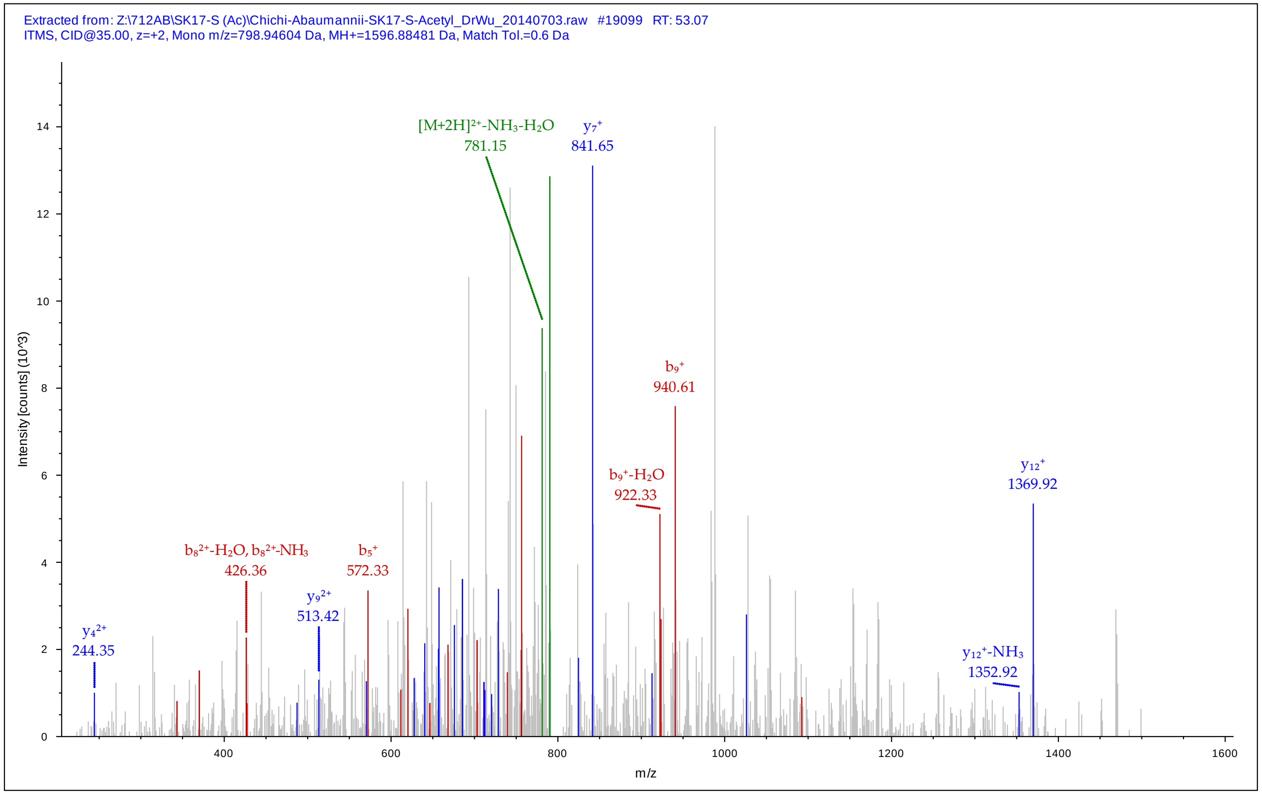

peg.2396   17900

#1	b^+	b^{2+}	Seq.	y^+	y^{2+}	#2
1	88.03931	44.52329	S			15
2	217.08191	109.04459	E	1471.80034	736.40381	14
3	330.16598	165.58663	L	1342.75774	671.88251	13
4	443.25005	222.12866	I	1229.67367	615.34047	12
5	558.27700	279.64214	D	1116.58960	558.79844	11
6	629.31412	315.16070	A	1001.56265	501.28496	10
7	742.39819	371.70273	I	930.52553	465.76640	9
8	813.43531	407.22129	A	817.44146	409.22437	8
9	942.47791	471.74259	E	746.40434	373.70581	7
10	1112.58344	556.79536	K-Acetyl	617.36174	309.18451	6
11	1169.60491	585.30609	G	447.25620	224.13174	5
12	1226.62638	613.81683	G	390.23473	195.62100	4
13	1325.69480	663.35104	V	333.21326	167.11027	3
14	1412.72683	706.86705	S	234.14484	117.57606	2
15			K	147.11281	74.06004	1

Peptide Summary

Sequence: SELIDAIAEKGGVSK, K10-Acetyl (42.01057 Da)
Charge: +2, Monoisotopic m/z: 779.91846 Da (-1.37 mmu/-1.75 ppm), MH+: 1558.82964 Da, RT: 50.54 min,
Identified with: Mascot (v1.30); IonScore:75, Exp Value:1.6E-007, Ions matched by search engine: 17/148
Fragment match tolerance used for search: 0.6 Da

Extracted from: Z:\712AB\SK17-S (Ac)\Chichi-Abaumannii-SK17-R-Acetyl_DrWu_20140512.raw #17900 RT: 50.54
ITMS, CID@35.00, z=+2, Mono m/z=779.91846 Da, MH+=1558.82964 Da, Match Tol.=0.6 Da

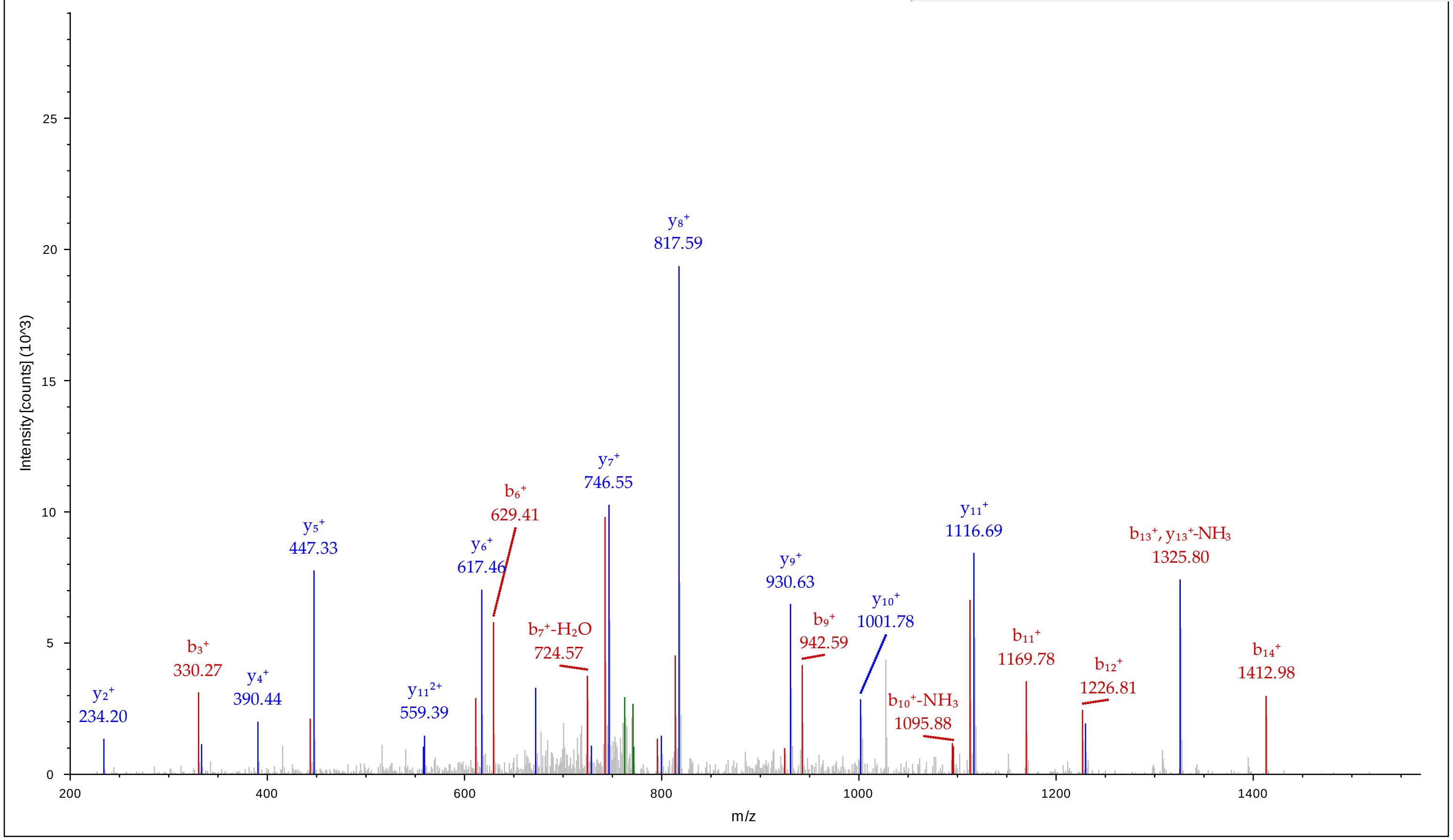

peg.2396 15971

Peptide Summary

Sequence: SELIDAIAEKGGVSK, K10-Acetyl (42.01057 Da)
Charge: +2, Monoisotopic m/z: 779.91833 Da (-1.49 mmu/-1.91 ppm), MH+: 1558.82939 Da, RT: 50.11 min,
Identified with: Mascot (v1.30); IonScore:86, Exp Value:5.5E-008, Ions matched by search engine: 21/148
Fragment match tolerance used for search: 0.6 Da

#1	b⁺	b²⁺	Seq.	y⁺	y²⁺	#2
1	88.03931	44.52329	S			15
2	217.08191	109.04459	E	1471.80034	736.40381	14
3	330.16598	165.58663	L	1342.75774	671.88251	13
4	443.25005	222.12866	I	1229.67367	615.34047	12
5	558.27700	279.64214	D	1116.58960	558.79844	11
6	629.31412	315.16070	A	1001.56265	501.28496	10
7	742.39819	371.70273	I	930.52553	465.76640	9
8	813.43531	407.22129	A	817.44146	409.22437	8
9	942.47791	471.74259	E	746.40434	373.70581	7
10	1112.58344	556.79536	K-Acetyl	617.36174	309.18451	6
11	1169.60491	585.30609	G	447.25620	224.13174	5
12	1226.62638	613.81683	G	390.23473	195.62100	4
13	1325.69480	663.35104	V	333.21326	167.11027	3
14	1412.72683	706.86705	S	234.14484	117.57606	2
15			K	147.11281	74.06004	1

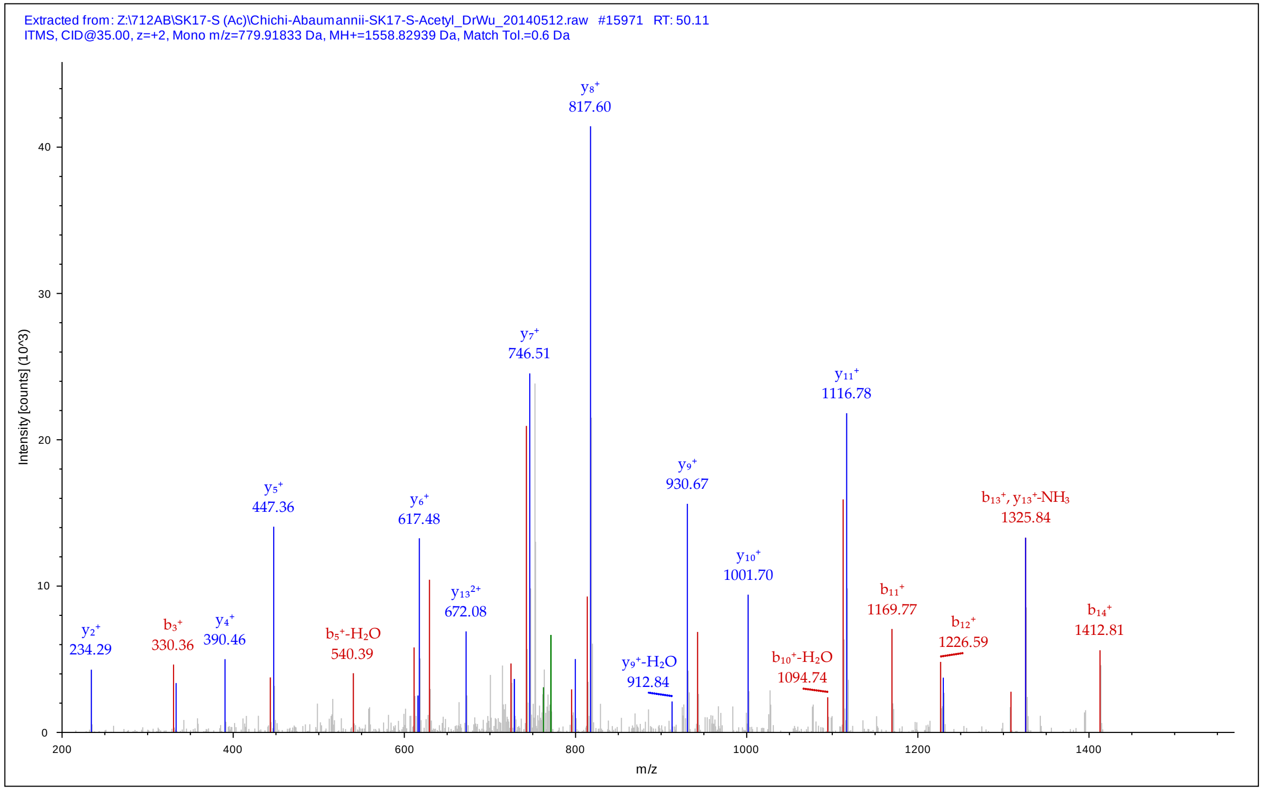

peg.2396   18037

Peptide Summary

Sequence: SELIDAIAEKGGVSK, K10-Acetyl (42.01057 Da)
Charge: +2, Monoisotopic m/z: 779.92090 Da (+1.08 mmu/+1.38 ppm), MH+: 1558.83452 Da, RT: 50.48 min,
Identified with: Sequest HT (v1.3); XCorr:3.00, Ions matched by search engine: 0/0
Fragment match tolerance used for search: 0.6 Da

#1	b^+	b^{2+}	Seq.	y^+	y^{2+}	#2
1	88.03931	44.52329	S			15
2	217.08191	109.04459	E	1471.80034	736.40381	14
3	330.16598	165.58663	L	1342.75774	671.88251	13
4	443.25005	222.12866	I	1229.67367	615.34047	12
5	558.27700	279.64214	D	1116.58960	558.79844	11
6	629.31412	315.16070	A	1001.56265	501.28496	10
7	742.39819	371.70273	I	930.52553	465.76640	9
8	813.43531	407.22129	A	817.44146	409.22437	8
9	942.47791	471.74259	E	746.40434	373.70581	7
10	1112.58344	556.79536	K-Acetyl	617.36174	309.18451	6
11	1169.60491	585.30609	G	447.25620	224.13174	5
12	1226.62638	613.81683	G	390.23473	195.62100	4
13	1325.69480	663.35104	V	333.21326	167.11027	3
14	1412.72683	706.86705	S	234.14484	117.57606	2
15			K	147.11281	74.06004	1

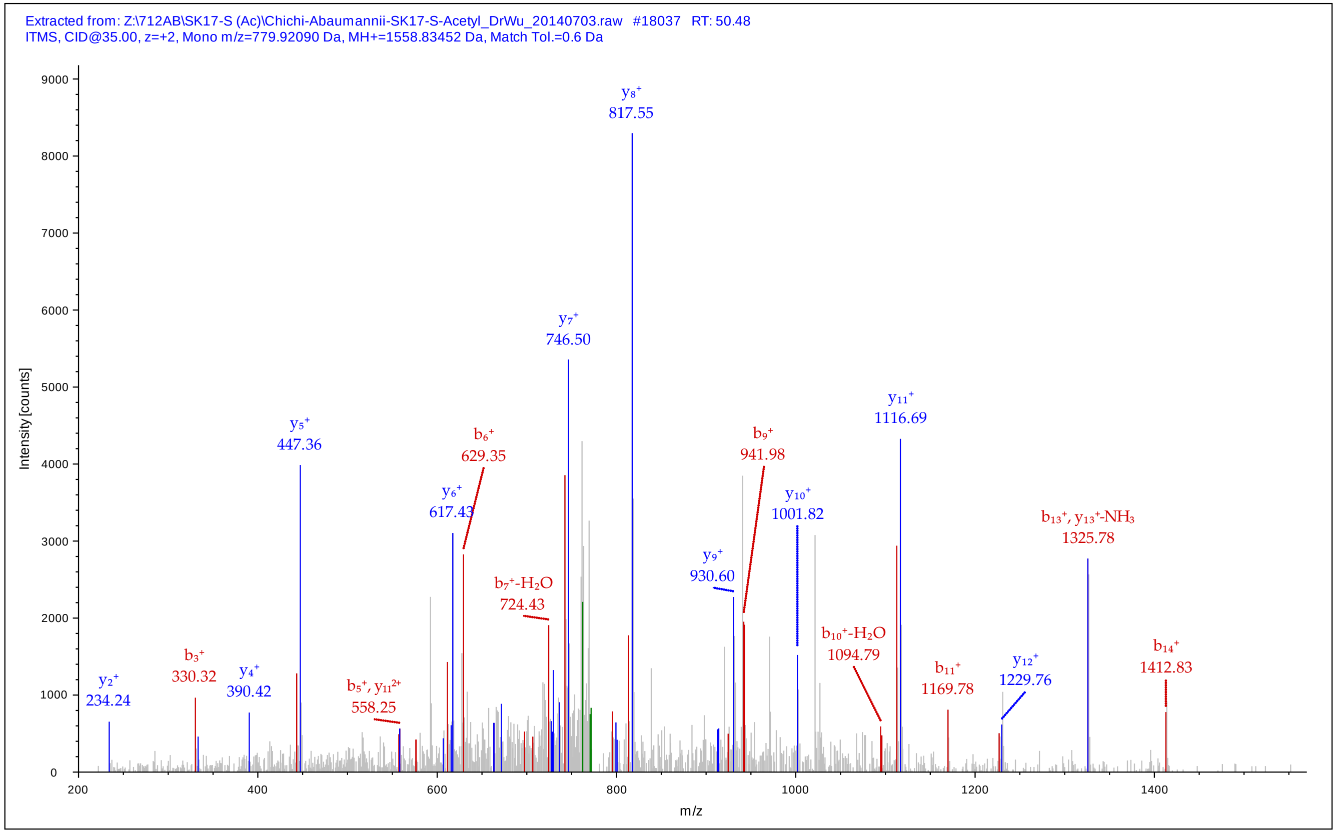

peg.2410 19636

#1	b⁺	b²⁺	b³⁺	Seq.	y⁺	y²⁺	y³⁺	#2
1	138.06619	69.53673	46.69358	H				29
2	301.12951	151.06839	101.04802	Y	3238.68565	1619.84646	1080.23340	28
3	414.21358	207.61043	138.74271	I	3075.62233	1538.31480	1025.87896	27
4	485.25070	243.12899	162.42175	A	2962.53826	1481.77277	988.18427	26
5	598.33477	299.67102	200.11644	L	2891.50114	1446.25421	964.50523	25
6	697.40319	349.20523	233.13925	V	2778.41707	1389.71217	926.81054	24
7	825.46177	413.23452	275.82544	Q	2679.34865	1340.17796	893.78773	23
8	882.48324	441.74526	294.83260	G	2551.29007	1276.14867	851.10154	22
9	1010.54182	505.77455	337.51879	Q	2494.26860	1247.63794	832.09438	21
10	1109.61024	555.30876	370.54160	V	2366.21002	1183.60865	789.40819	20
11	1279.71577	640.36152	427.24344	K-Acetyl	2267.14160	1134.07444	756.38538	19
12	1407.77435	704.39081	469.92963	Q	2097.03606	1049.02167	699.68354	18
13	1536.81695	768.91211	512.94383	E	1968.97748	984.99238	656.99734	17
14	1593.83842	797.42285	531.95099	G	1839.93488	920.47108	613.98314	16
15	1680.87045	840.93886	560.96167	S	1782.91341	891.96034	594.97599	15
16	1779.93887	890.47307	593.98447	V	1695.88138	848.44433	565.96531	14
17	1908.98147	954.99437	636.99867	E	1596.81296	798.91012	532.94250	13
18	1980.01859	990.51293	660.67771	A	1467.77036	734.38882	489.92830	12
19	2077.07136	1039.03932	693.02864	P	1396.73324	698.87026	466.24926	11
20	2190.15543	1095.58135	730.72333	L	1299.68047	650.34387	433.89834	10
21	2303.23950	1152.12339	768.41802	I	1186.59640	593.80184	396.20365	9
22	2404.28718	1202.64723	802.10058	T	1073.51233	537.25980	358.50896	8
23	2519.31413	1260.16070	840.44289	D	972.46465	486.73596	324.82640	7
24	2705.39345	1353.20036	902.46933	W	857.43770	429.22249	286.48408	6
25	2834.43605	1417.72166	945.48353	E	671.35838	336.18283	224.45764	5
26	2948.47898	1474.74313	983.49784	N	542.31578	271.66153	181.44344	4
27	3104.58010	1552.79369	1035.53155	R	428.27285	214.64006	143.42913	3
28	3201.63287	1601.32007	1067.88247	P	272.17173	136.58950	91.39543	2
29				R	175.11896	88.06312	59.04450	1

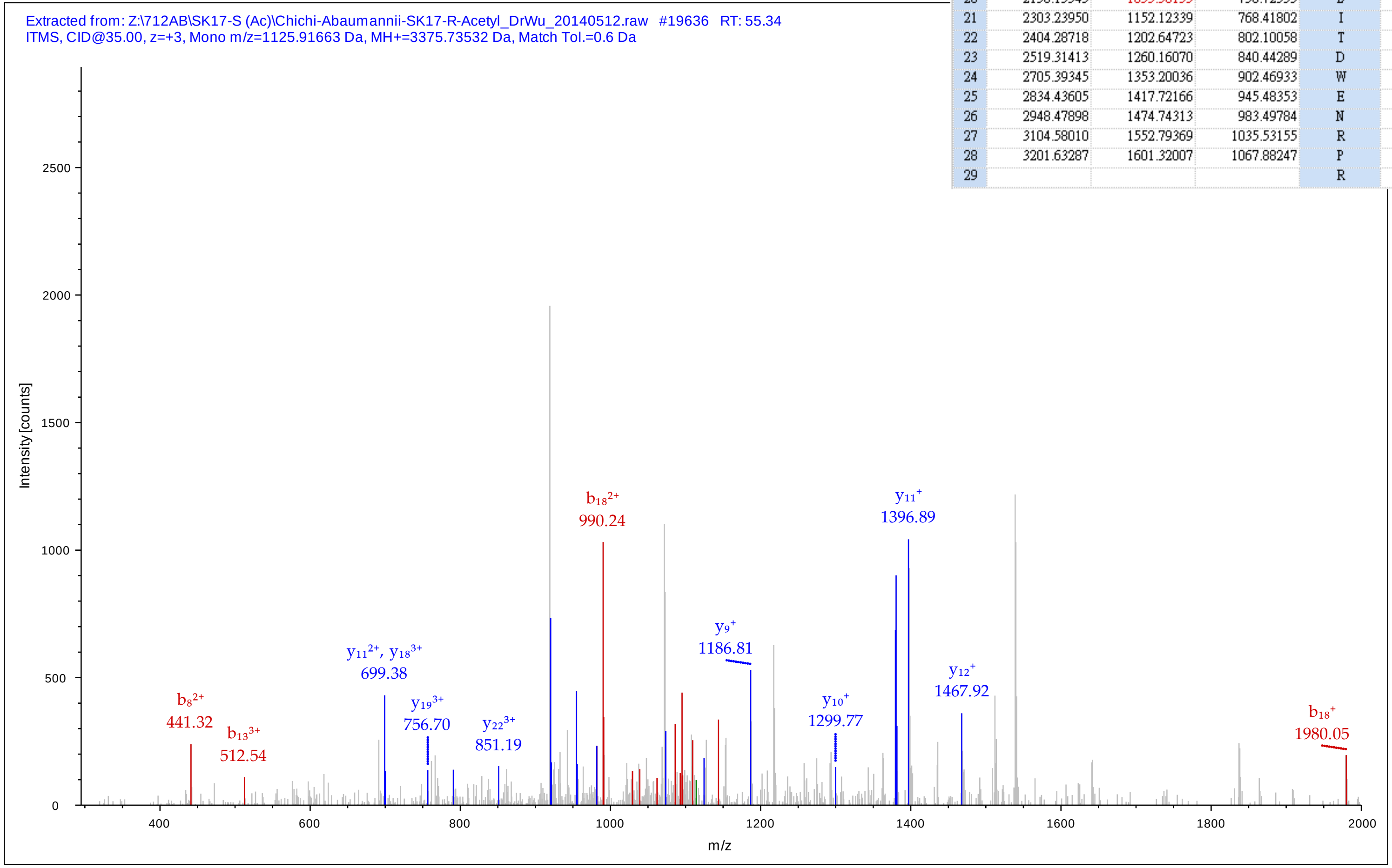

peg.2423 25397

#1	b^+	b^{2+}	b^{3+}	Seq.	y^+	y^{2+}	y^{3+}	#2
1	115.05021	58.02874	39.02159	N				38
2	228.13428	114.57078	76.71628	I	3777.95247	1889.47987	1259.98901	37
3	327.20270	164.10499	109.73908	V	3664.86840	1832.93784	1222.29432	36
4	464.26161	232.63444	155.42539	H	3565.79998	1783.40363	1189.27151	35
5	567.27080	284.13904	189.76178	C	3428.74107	1714.87417	1143.58521	34
6	624.29227	312.64977	208.76894	G	3325.73188	1663.36958	1109.24881	33
7	739.31922	370.16325	247.11126	D	3268.71041	1634.85884	1090.24165	32
8	838.38764	419.69746	280.13406	V	3153.68346	1577.34537	1051.89934	31
9	895.40911	448.20819	299.14122	G	3054.61504	1527.81116	1018.87653	30
10	966.44623	483.72675	322.82026	A	2997.59357	1499.30042	999.86937	29
11	1023.46770	512.23749	341.82742	G	2926.55645	1463.78186	976.19033	28
12	1151.52628	576.26678	384.51361	Q	2869.53498	1435.27113	957.18318	27
13	1264.61035	632.80881	422.20830	I	2741.47640	1371.24184	914.49698	26
14	1335.64747	668.32737	445.88734	A	2628.39233	1314.69980	876.80229	25
15	1505.75300	753.38014	502.58918	K-Acetyl	2557.35521	1279.18124	853.12325	24
16	1618.83707	809.92217	540.28387	I	2387.24967	1194.12847	796.42141	23
17	1778.86773	889.93750	593.62743	C-Carbam···	2274.16560	1137.58644	758.72672	22
18	1892.91066	946.95897	631.64174	N	2114.13495	1057.57111	705.38317	21
19	2006.95359	1003.98043	669.65605	N	2000.09202	1000.54965	667.36886	20
20	2120.03766	1060.52247	707.35074	L	1886.04909	943.52818	629.35455	19
21	2233.12173	1117.06450	745.04543	I	1772.96502	886.98615	591.65986	18
22	2346.20580	1173.60654	782.74012	L	1659.88095	830.44411	553.96517	17
23	2403.22727	1202.11727	801.74727	G	1546.79688	773.90208	516.27048	16
24	2516.31134	1258.65931	839.44196	I	1489.77541	745.39134	497.26332	15
25	2603.34337	1302.17532	868.45264	S	1376.69134	688.84931	459.56863	14
26	2734.38387	1367.69557	912.13281	M	1289.65931	645.33329	430.55795	13
27	2805.42099	1403.21413	935.81185	A	1158.61881	579.81304	386.87779	12
28	2876.45811	1438.73269	959.49089	A	1087.58169	544.29448	363.19875	11
29	2975.52653	1488.26690	992.51369	V	1016.54457	508.77592	339.51971	10
30	3046.56365	1523.78546	1016.19273	A	917.47615	459.24171	306.49690	9
31	3175.60625	1588.30676	1059.20693	E	846.43903	423.72315	282.81786	8
32	3232.62772	1616.81750	1078.21409	G	717.39643	359.20185	239.80366	7
33	3363.66822	1682.33775	1121.89426	M	660.37496	330.69112	220.79650	6
34	3434.70534	1717.85631	1145.57330	A	529.33446	265.17087	177.11634	5
35	3547.78941	1774.39834	1183.26799	L	458.29734	229.65231	153.43730	4
36	3604.81088	1802.90908	1202.27514	G	345.21327	173.11027	115.74261	3
37	3703.87930	1852.44329	1235.29795	V	288.19180	144.59954	96.73545	2
38				K-Acetyl	189.12338	95.06533	63.71264	1

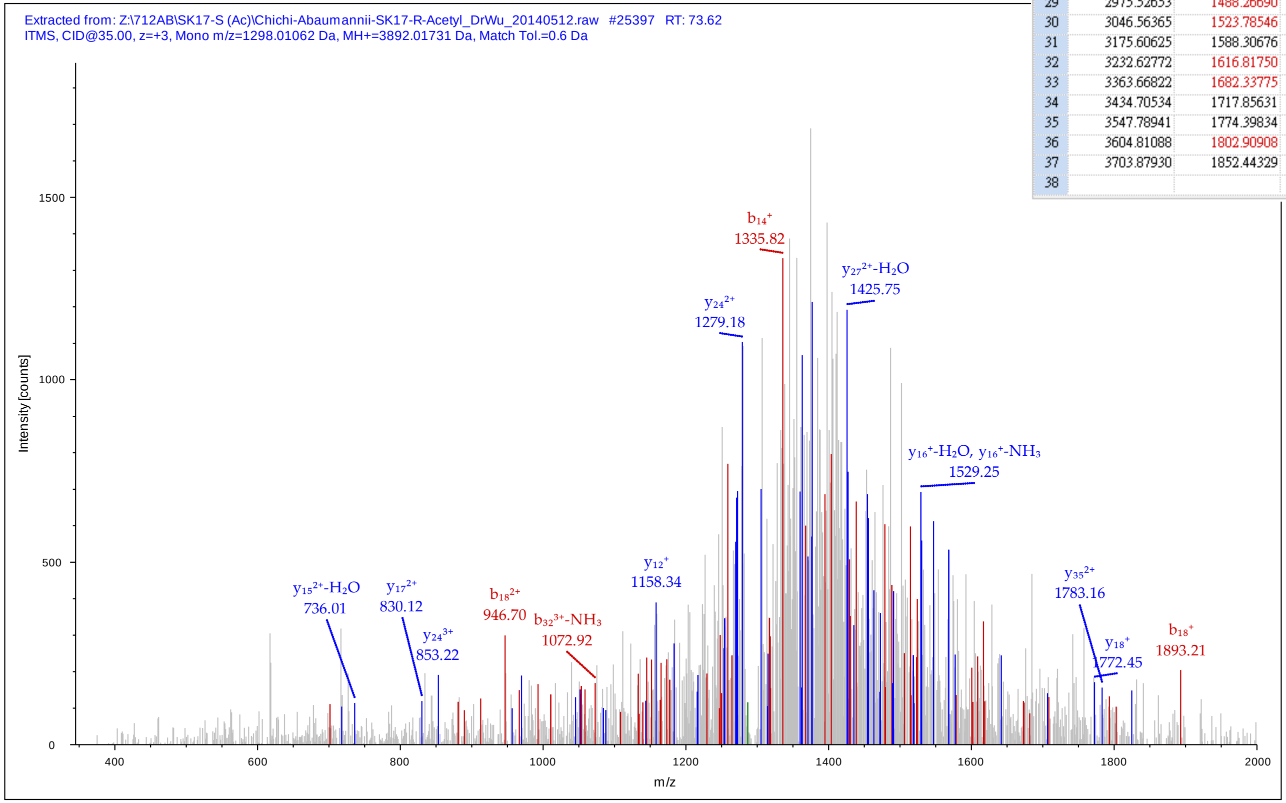

peg.2484 27598

#1	b^+	b^{2+}	b^{3+}	Seq.	y^+	y^{2+}	y^{3+}	#2
1	156.10191	78.55459	52.70549	I-Acetyl				23
2	316.13257	158.56992	106.04904	C-Carbam···	2466.23684	1233.62206	822.75046	22
3	387.16969	194.08848	129.72808	A	2306.20618	1153.60673	769.40691	21
4	486.23811	243.62269	162.75089	V	2235.16906	1118.08817	745.72787	20
5	543.25958	272.13343	181.75804	G	2136.10064	1068.55396	712.70506	19
6	671.31816	336.16272	224.44424	Q	2079.07917	1040.04322	693.69791	18
7	742.35528	371.68128	248.12328	A	1951.02059	976.01393	651.01171	17
8	889.42370	445.21549	297.14608	F	1879.98347	940.49537	627.33267	16
9	976.45573	488.73150	326.15676	S	1732.91505	866.96116	578.30987	15
10	1089.53980	545.27354	363.85145	L	1645.88302	823.44515	549.29919	14
11	1204.56675	602.78701	402.19377	D	1532.79895	766.90311	511.60450	13
12	1303.63517	652.32122	435.21657	V	1417.77200	709.38964	473.26218	12
13	1450.67058	725.83893	484.22838	M-Oxidation	1318.70358	659.85543	440.23938	11
14	1551.71826	776.36277	517.91094	T	1171.66817	586.33772	391.22757	10
15	1721.82380	861.41554	574.61278	K-Acetyl	1070.62049	535.81388	357.54501	9
16	1834.90787	917.95757	612.30747	L	900.51495	450.76111	300.84317	8
17	1947.99194	974.49961	650.00216	L	787.43088	394.21908	263.14848	7
18	2062.03487	1031.52107	688.01647	N	674.34681	337.67704	225.45379	6
19	2175.11894	1088.06311	725.71116	I	560.30388	280.65558	187.43948	5
20	2289.16187	1145.08457	763.72547	N	447.21981	224.11354	149.74479	4
21	2360.19899	1180.60313	787.40451	A	333.17688	167.09208	111.73048	3
22	2475.22594	1238.11661	825.74683	D	262.13976	131.57352	88.05144	2
23				K	147.11281	74.06004	49.70912	1

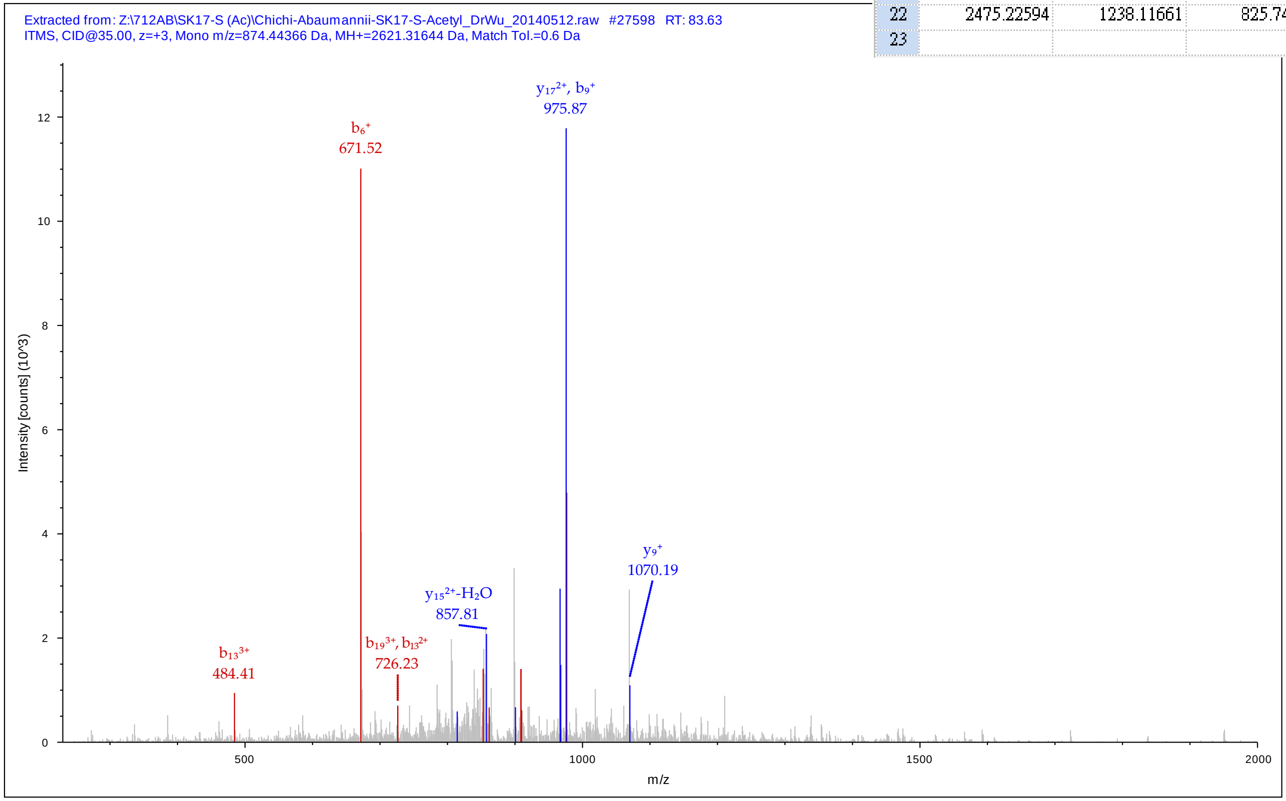

peg.25 28615

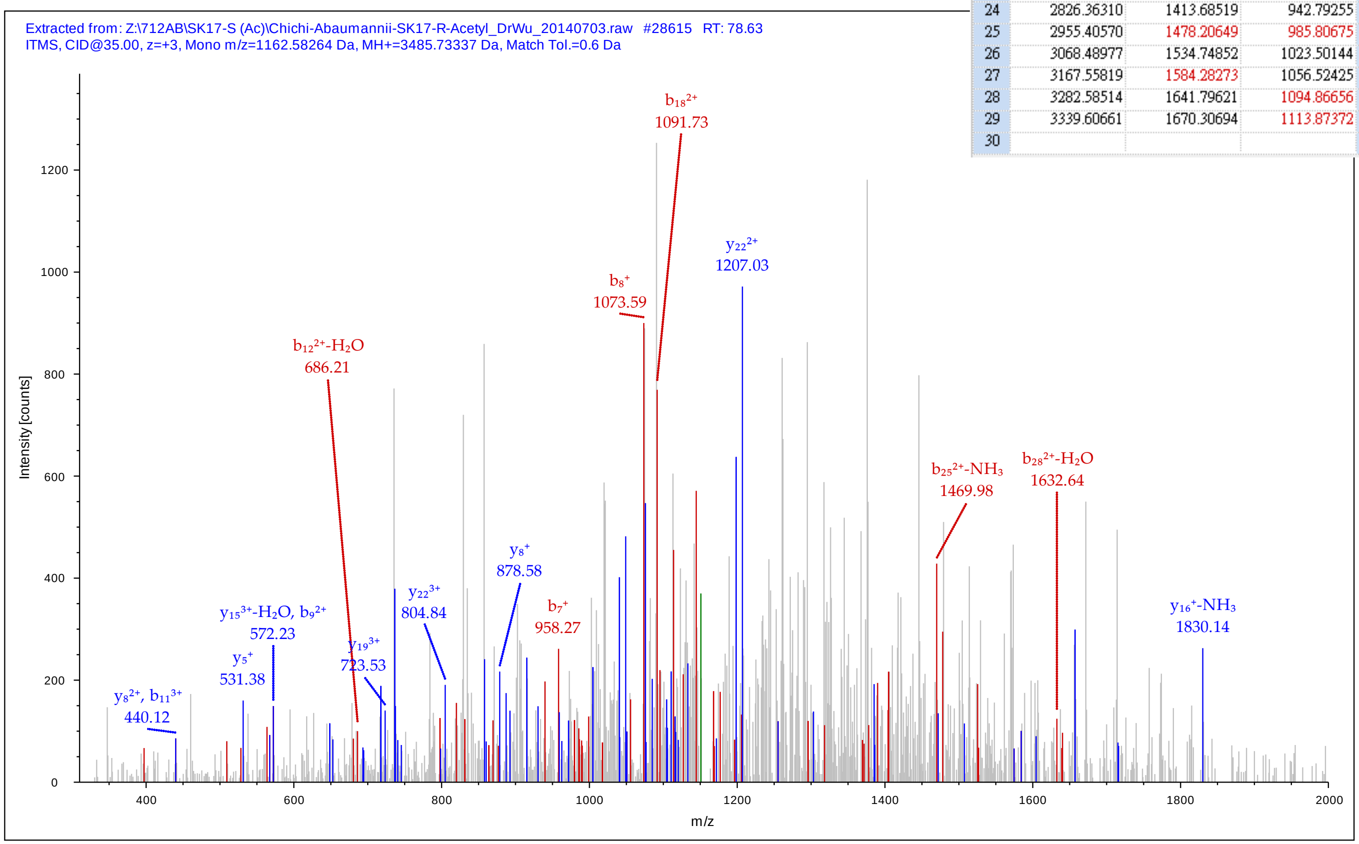

#1	b^+	b^{2+}	b^{3+}	Seq.	y^+	y^{2+}	y^{3+}	#2
1	156.10191	78.55459	52.70549	L-Acetyl				30
2	319.16523	160.08625	107.05993	Y	3330.61751	1665.81239	1110.87735	29
3	456.22414	228.61571	152.74623	H	3167.55419	1584.28073	1056.52291	28
4	585.26674	293.13701	195.76043	E	3030.49528	1515.75128	1010.83661	27
5	698.35081	349.67904	233.45512	L	2901.45268	1451.22998	967.82241	26
6	811.43488	406.22108	271.14981	L	2788.36861	1394.68794	930.12772	25
7	958.47030	479.73879	320.16162	M-Oxidation	2675.28454	1338.14591	892.43303	24
8	1073.49725	537.25226	358.50393	D	2528.24913	1264.62820	843.42123	23
9	1144.53437	572.77082	382.18297	A	2413.22218	1207.11473	805.07891	22
10	1247.54356	624.27542	416.51937	C	2342.18506	1171.59617	781.39987	21
11	1318.58068	659.79398	440.19841	A	2239.17587	1120.09157	747.06347	20
12	1389.61780	695.31254	463.87745	A	2168.13875	1084.57301	723.38443	19
13	1526.67671	763.84199	509.56375	H	2097.10163	1049.05445	699.70539	18
14	1639.76078	820.38403	547.25844	L	1960.04272	980.52500	654.01909	17
15	1752.84485	876.92606	584.95313	I	1846.95865	923.98296	616.32440	16
16	1881.88745	941.44736	627.96733	E	1733.87458	867.44093	578.62971	15
17	1996.91440	998.96084	666.30965	D	1604.83198	802.91963	535.61551	14
18	2182.99372	1092.00050	728.33609	W	1489.80503	745.40615	497.27319	13
19	2353.09925	1177.05326	785.03793	K-Acetyl	1303.72571	652.36649	435.24675	12
20	2410.12072	1205.56400	804.04509	G	1133.62017	567.31372	378.54491	11
21	2509.18914	1255.09821	837.06790	V	1076.59870	538.80299	359.53775	10
22	2608.25756	1304.63242	870.09070	V	977.53028	489.26878	326.51494	9
23	2755.32598	1378.16663	919.11351	F	878.46186	439.73457	293.49214	8
24	2826.36310	1413.68519	942.79255	A	731.39344	366.20036	244.46933	7
25	2955.40570	1478.20649	985.80675	E	660.35632	330.68180	220.79029	6
26	3068.48977	1534.74852	1023.50144	I	531.31372	266.16050	177.77609	5
27	3167.55819	1584.28273	1056.52425	V	418.22965	209.61846	140.08140	4
28	3282.58514	1641.79621	1094.86656	D	319.16123	160.08425	107.05859	3
29	3339.60661	1670.30694	1113.87372	G	204.13428	102.57078	68.71628	2
30				K	147.11281	74.06004	49.70912	1

peg.252   18050

#1	b⁺	b²⁺	b³⁺	Seq.	y⁺	y²⁺	y³⁺	#2
1	114.05496	57.53112	38.68984	A-Acetyl				14
2	213.12338	107.06533	71.71264	V	1556.88297	778.94512	519.63251	13
3	369.22450	185.11589	123.74635	R	1457.81455	729.41091	486.60970	12
4	484.25145	242.62936	162.08867	D	1301.71343	651.36035	434.57599	11
5	615.29195	308.14961	205.76883	M	1186.68648	593.84688	396.23368	10
6	686.32907	343.66817	229.44787	A	1055.64598	528.32663	352.55351	9
7	799.41314	400.21021	267.14256	L	984.60886	492.80807	328.87447	8
8	870.45026	435.72877	290.82160	A	871.52479	436.26603	291.17978	7
9	999.49286	500.25007	333.83580	E	800.48767	400.74747	267.50074	6
10	1169.59840	585.30284	390.53765	K-Acetyl	671.44507	336.22617	224.48654	5
11	1282.68247	641.84487	428.23234	L	501.33953	251.17340	167.78469	4
12	1395.76654	698.38691	465.92703	I	388.25546	194.63137	130.09000	3
13	1523.82512	762.41620	508.61322	Q	275.17139	138.08933	92.39531	2
14				K	147.11281	74.06004	49.70912	1

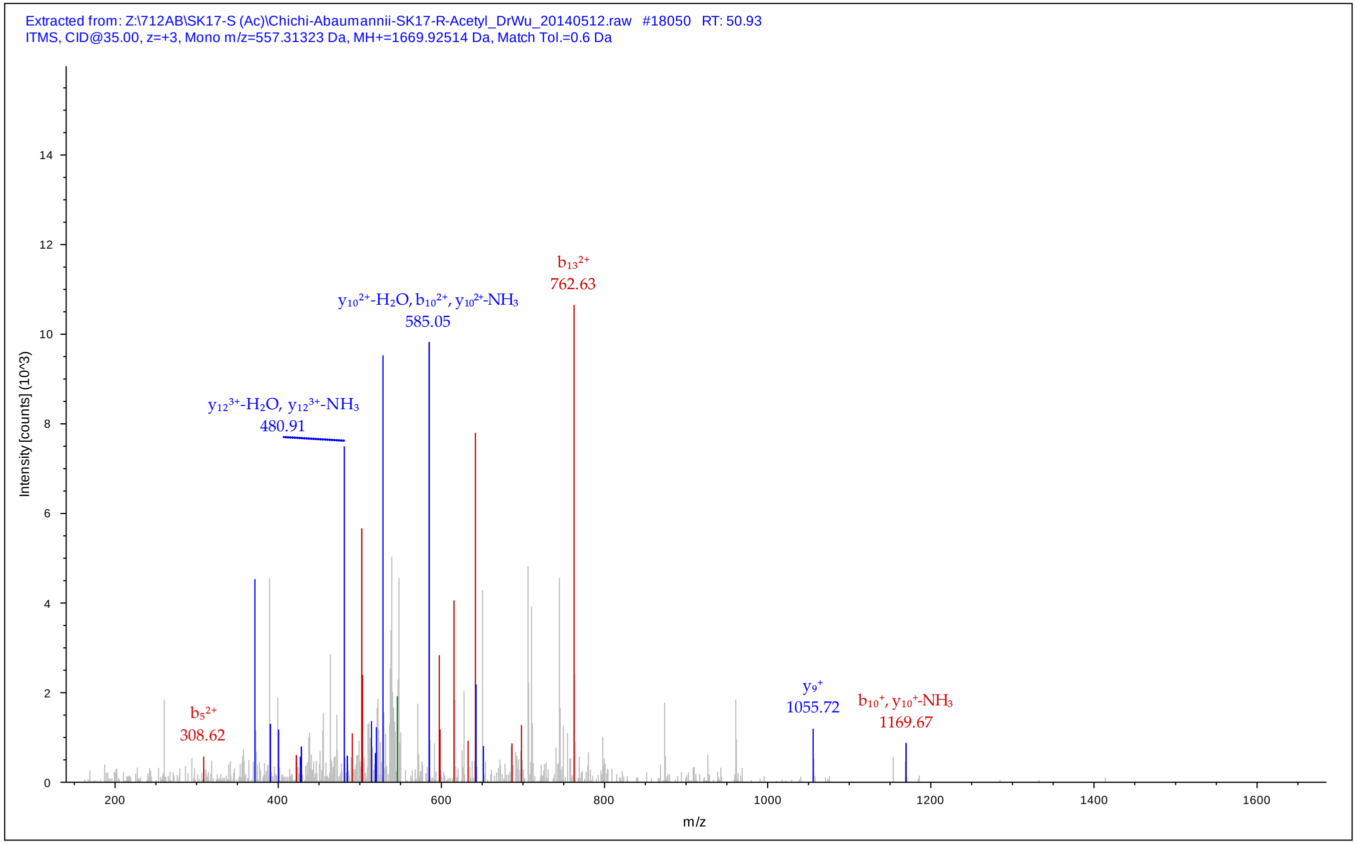

peg.252 16111

#1	b⁺	b²⁺	b³⁺	Seq.	y⁺	y²⁺	y³⁺	#2
1	114.05496	57.53112	38.68984	A-Acetyl				14
2	213.12338	107.06533	71.71264	V	1556.88297	778.94512	519.63251	13
3	369.22450	185.11589	123.74635	R	1457.81455	729.41091	486.60970	12
4	484.25145	242.62936	162.08867	D	1301.71343	651.36035	434.57599	11
5	615.29195	308.14961	205.76883	M	1186.68648	593.84688	396.23368	10
6	686.32907	343.66817	229.44787	A	1055.64598	528.32663	352.55351	9
7	799.41314	400.21021	267.14256	L	984.60886	492.80807	328.87447	8
8	870.45026	435.72877	290.82160	A	871.52479	436.26603	291.17978	7
9	999.49286	500.25007	333.83580	E	800.48767	400.74747	267.50074	6
10	1169.59840	585.30284	390.53765	K-Acetyl	671.44507	336.22617	224.48654	5
11	1282.68247	641.84487	428.23234	L	501.33953	251.17340	167.78469	4
12	1395.76654	698.38691	465.92703	I	388.25546	194.63137	130.09000	3
13	1523.82512	762.41620	508.61322	Q	275.17139	138.08933	92.39531	2
14				K	147.11281	74.06004	49.70912	1

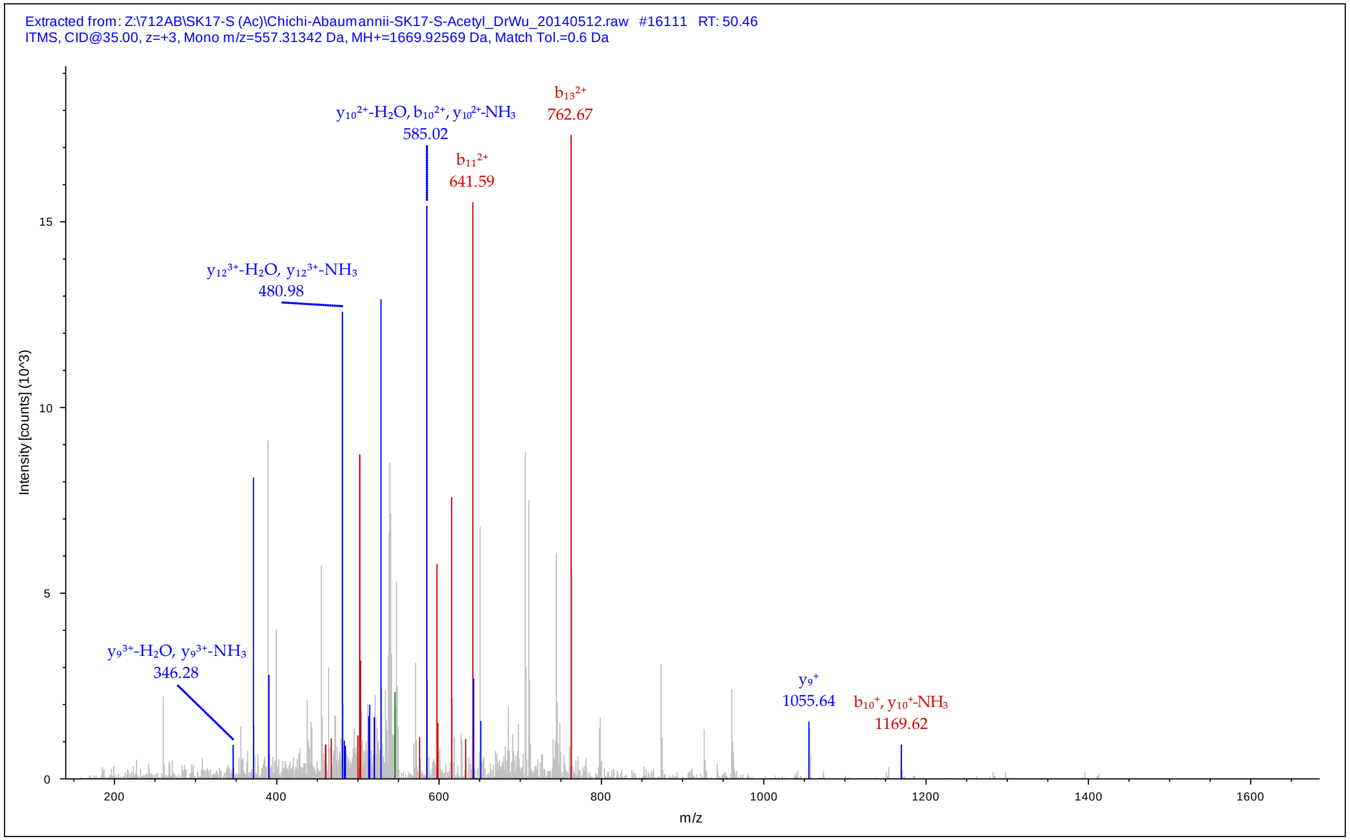

peg.2546 7127

#1	b+	b2+	Seq.	y+	y2+	#2
1	174.05834	87.53281	M-Acetyl			11
2	245.09546	123.05137	A	1171.66817	586.33772	10
3	373.15404	187.08066	Q	1100.63105	550.81916	9
4	444.19116	222.59922	A	972.57247	486.78987	8
5	614.29670	307.65199	K-Acetyl	901.53535	451.27131	7
6	784.40223	392.70475	K-Acetyl	731.42982	366.21855	6
7	871.43426	436.22077	S	561.32428	281.16578	5
8	970.50268	485.75498	V	474.29225	237.64976	4
9	1085.52963	543.26845	D	375.22383	188.11555	3
10	1198.61370	599.81049	I	260.19688	130.60208	2
11			K	147.11281	74.06004	1

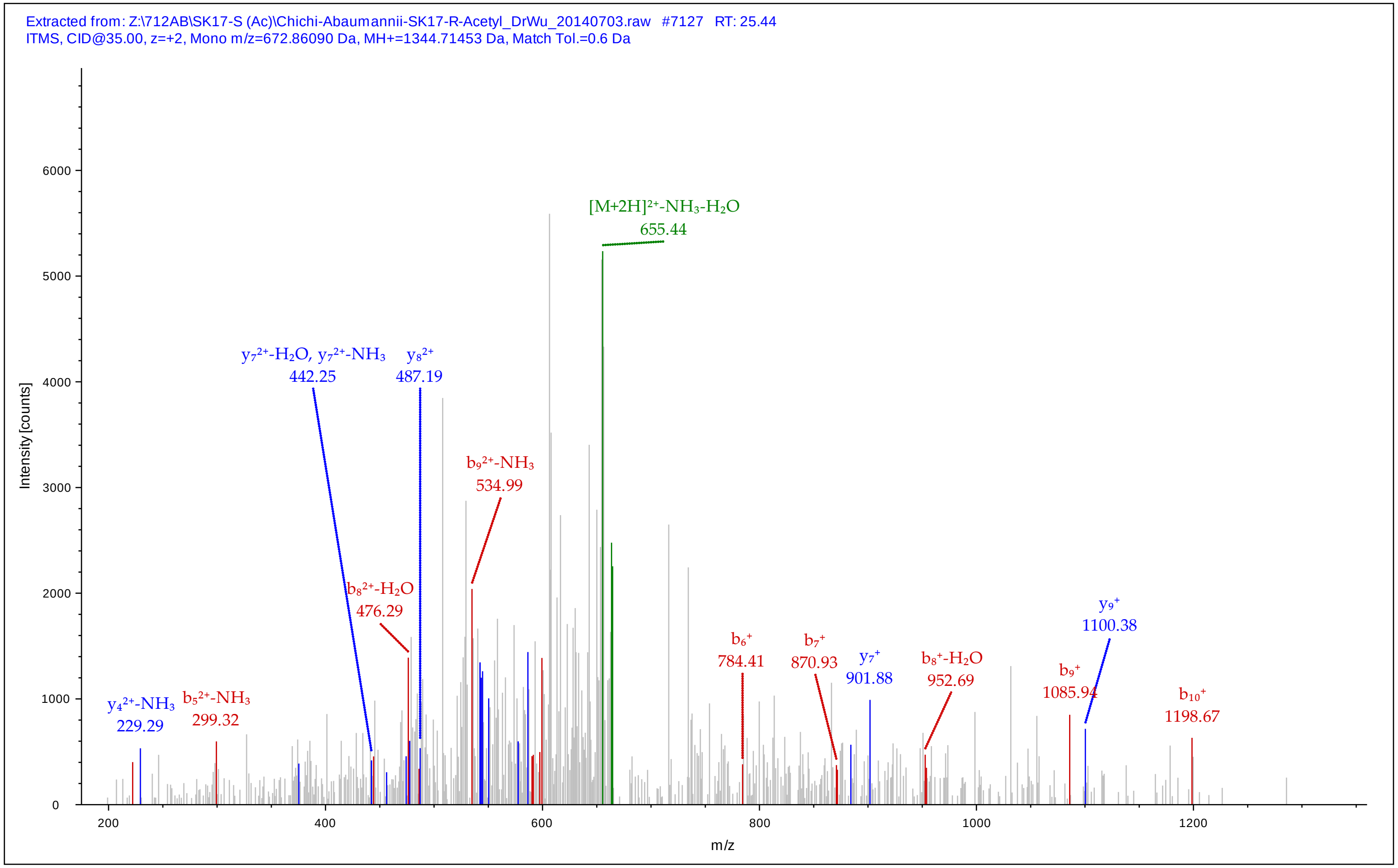

peg.2561 13080

#1	b+	b2+	Seq.	y+	y2+	#2
1	138.06619	69.53673	H			13
2	235.11896	118.06312	P	1442.73087	721.86907	12
3	349.16189	175.08458	N	1345.67810	673.34269	11
4	462.24596	231.62662	I	1231.63517	616.32122	10
5	577.27291	289.14009	D	1118.55110	559.77919	9
6	648.31003	324.65865	A	1003.52415	502.26571	8
7	776.36861	388.68794	Q	932.48703	466.74715	7
8	875.43703	438.22215	V	804.42845	402.71786	6
9	1045.54256	523.27492	K-Acetyl	705.36003	353.18365	5
10	1174.58516	587.79622	E	535.25449	268.13088	4
11	1302.64374	651.82551	Q	406.21189	203.60958	3
12	1433.68424	717.34576	M	278.15331	139.58029	2
13			K	147.11281	74.06004	1

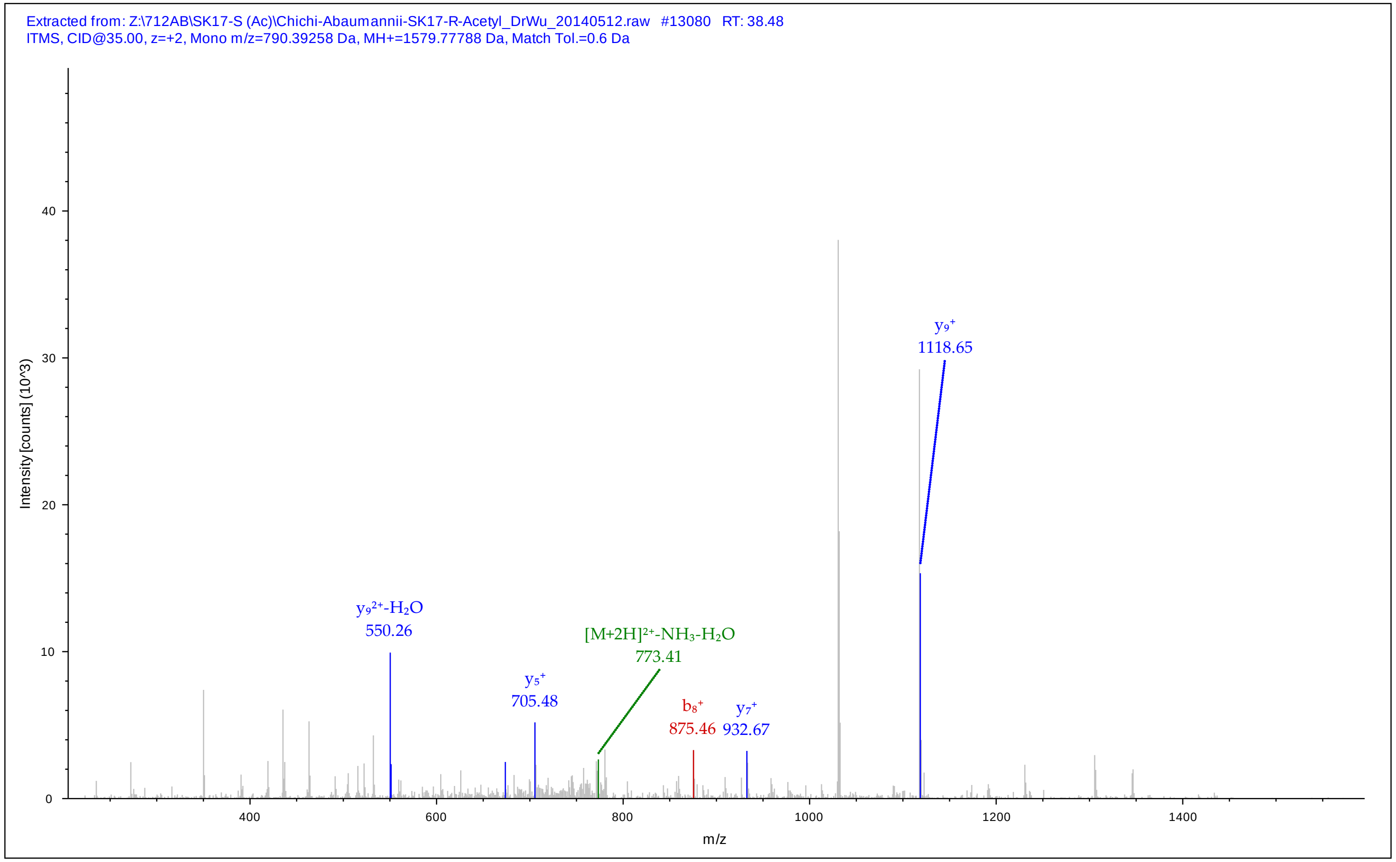

peg.2561 13081

#1	b⁺	b²⁺	Seq.	y⁺	y²⁺	#2
1	138.06619	69.53673	H			13
2	235.11896	118.06312	P	1442.73087	721.86907	12
3	349.16189	175.08458	N	1345.67810	673.34269	11
4	462.24596	231.62662	I	1231.63517	616.32122	10
5	577.27291	289.14009	D	1118.55110	559.77919	9
6	648.31003	324.65865	A	1003.52415	502.26571	8
7	776.36861	388.68794	Q	932.48703	466.74715	7
8	875.43703	438.22215	V	804.42845	402.71786	6
9	1045.54256	523.27492	K-Acetyl	705.36003	353.18365	5
10	1174.58516	587.79622	E	535.25449	268.13088	4
11	1302.64374	651.82551	Q	406.21189	203.60958	3
12	1433.68424	717.34576	M	278.15331	139.58029	2
13			K	147.11281	74.06004	1

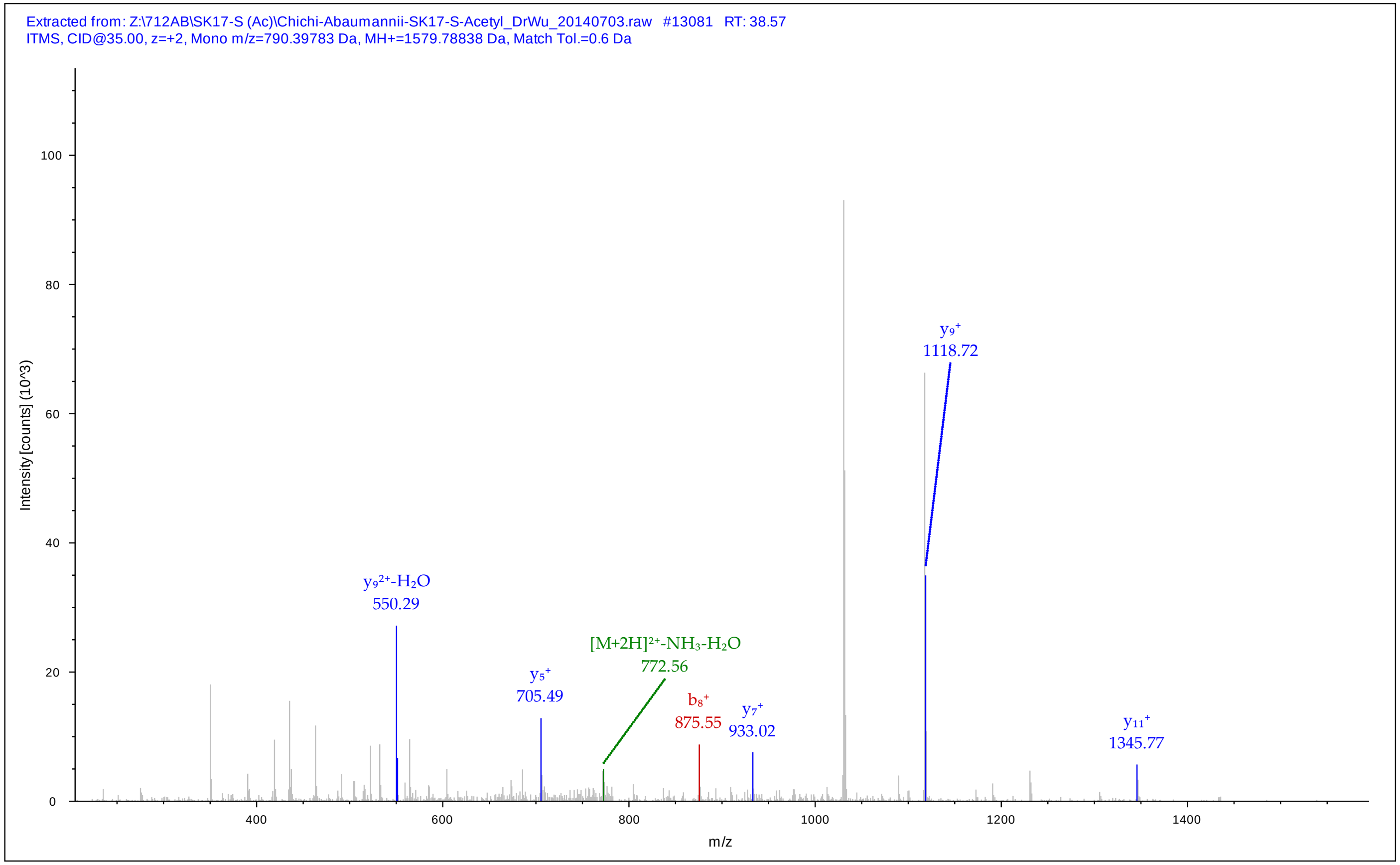

peg.2596 8965 (K8)

#1	b^+	b^{2+}	b^{3+}	Seq.	y^+	y^{2+}	y^{3+}	#2
1	72.04440	36.52584	24.68632	A				15
2	200.13937	100.57332	67.38464	K	1643.82360	822.41544	548.61272	14
3	315.16632	158.08680	105.72696	D	1515.72863	758.36795	505.91439	13
4	444.20892	222.60810	148.74116	E	1400.70168	700.85448	467.57208	12
5	515.24604	258.12666	172.42020	A	1271.65908	636.33318	424.55788	11
6	644.28864	322.64796	215.43440	E	1200.62196	600.81462	400.87884	10
7	773.33124	387.16926	258.44860	E	1071.57936	536.29332	357.86464	9
8	943.43677	472.22202	315.15044	K-Acetyl	942.53676	471.77202	314.85044	8
9	1072.47937	536.74332	358.16464	E	772.43122	386.71925	258.14859	7
10	1143.51649	572.26188	381.84368	A	643.38862	322.19795	215.13439	6
11	1242.58491	621.79609	414.86649	V	572.35150	286.67939	191.45535	5
12	1313.62203	657.31465	438.54553	A	473.28308	237.14518	158.43254	4
13	1427.66496	714.33612	476.55984	N	402.24596	201.62662	134.75350	3
14	1540.74903	770.87815	514.25453	L	288.20303	144.60515	96.73919	2
15				R	175.11896	88.06312	59.04450	1

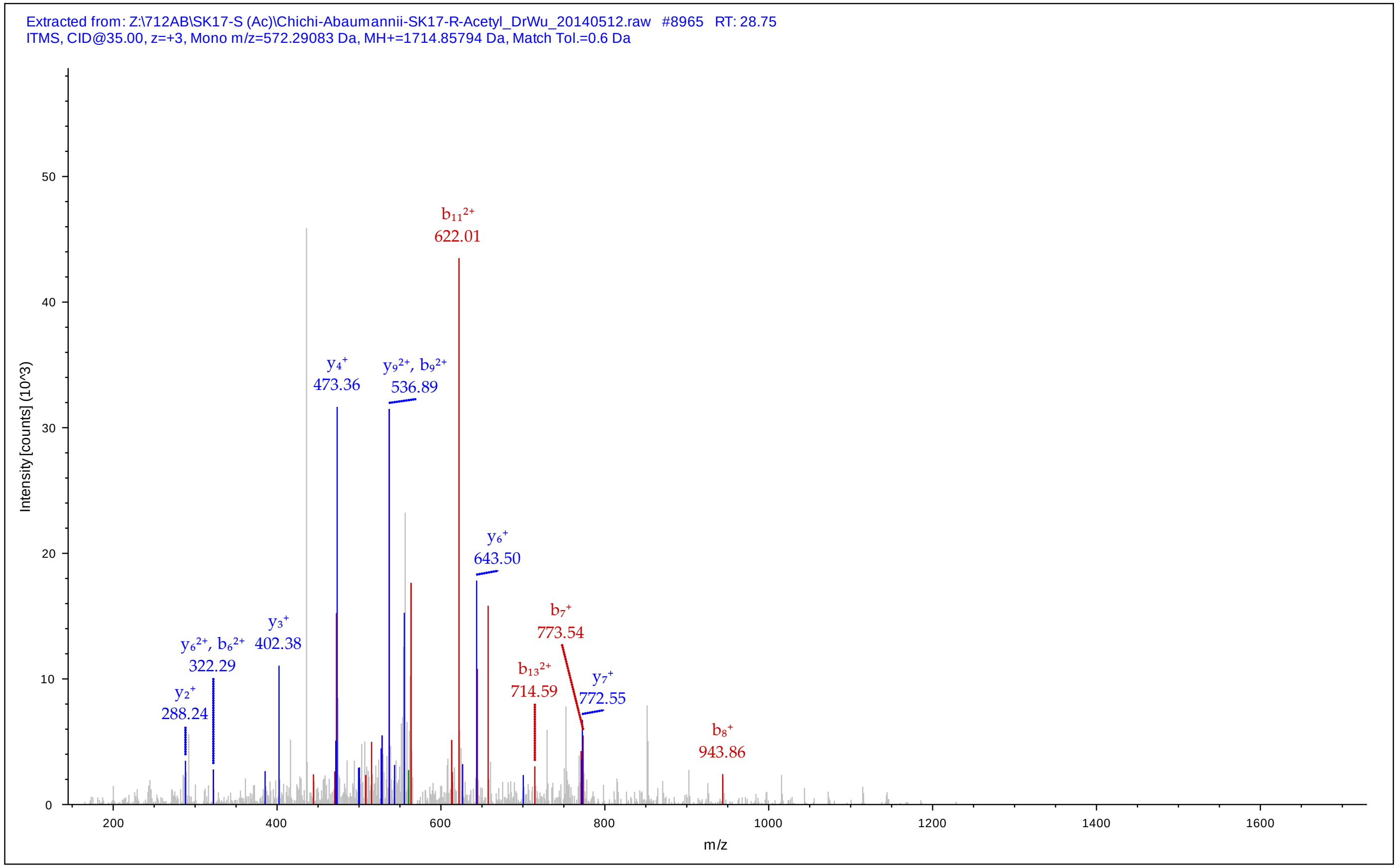

peg.2596 16841

#1	b+	b2+	Seq.	y+	y2+	#2
1	114.09135	57.54931	L			16
2	185.12847	93.06787	A	1687.84981	844.42854	15
3	300.15542	150.58135	D	1616.81269	808.90998	14
4	429.19802	215.10265	E	1501.78574	751.39651	13
5	528.26644	264.63686	V	1372.74314	686.87521	12
6	657.30904	329.15816	E	1273.67472	637.34100	11
7	728.34616	364.67672	A	1144.63212	572.81970	10
8	829.39384	415.20056	T	1073.59500	537.30114	9
9	942.47791	471.74259	L	972.54732	486.77730	8
10	1112.58344	556.79536	K-Acetyl	859.46325	430.23526	7
11	1183.62056	592.31392	A	689.35771	345.18249	6
12	1270.65259	635.82993	S	618.32059	309.66393	5
13	1399.69519	700.35123	E	531.28856	266.14792	4
14	1512.77926	756.89327	I	402.24596	201.62662	3
15	1626.82219	813.91473	N	289.16189	145.08458	2
16			R	175.11896	88.06312	1

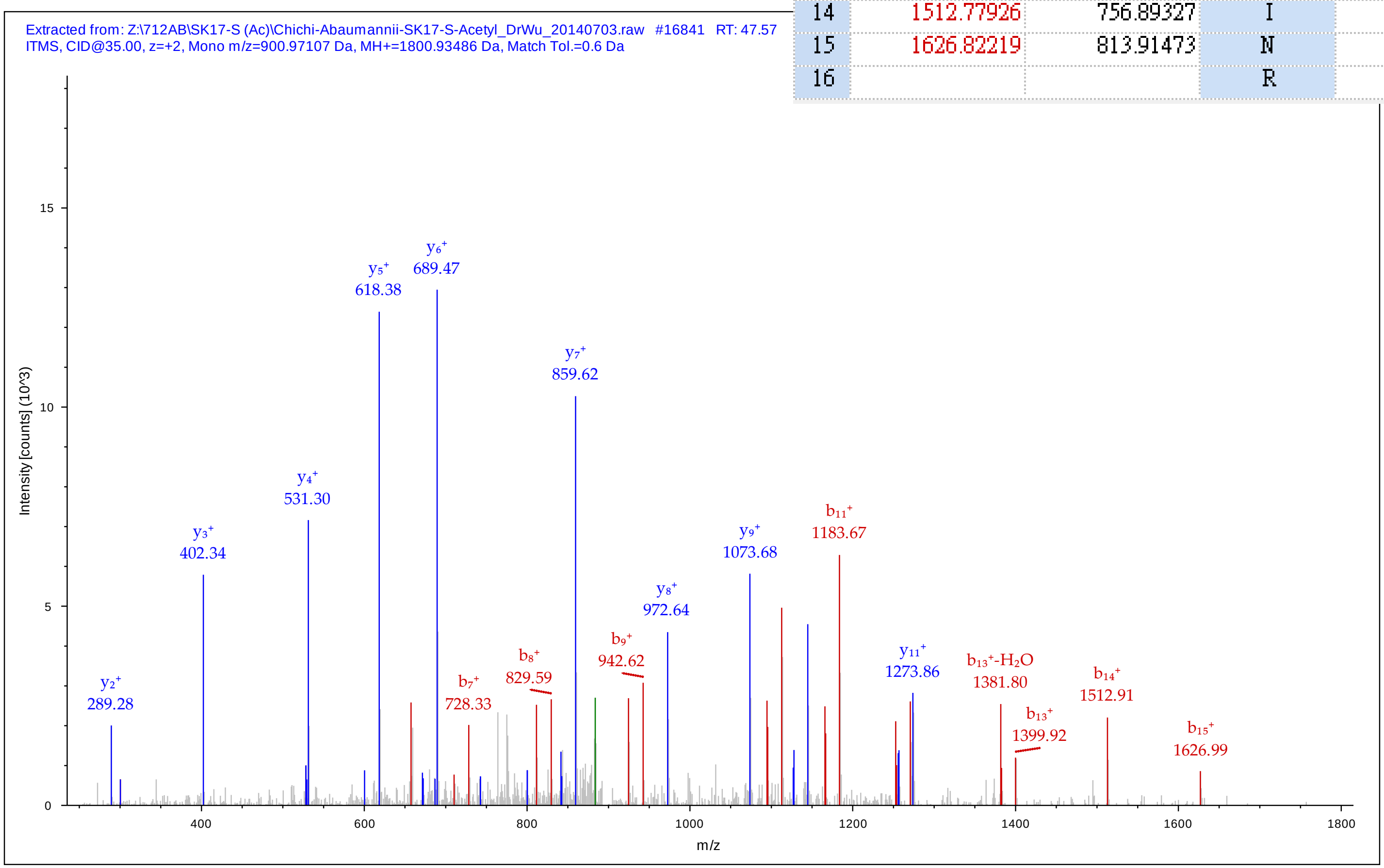

peg.2616   10965

#1	b⁺	b²⁺	Seq.	y⁺	y²⁺	#2
1	158.04479	79.52603	D-Acetyl			11
2	344.12411	172.56569	W	1372.67438	686.84083	10
3	431.15614	216.08171	S	1186.59506	593.80117	9
4	560.19874	280.60301	E	1099.56303	550.28515	8
5	657.25151	329.12939	P	970.52043	485.76385	7
6	827.35705	414.18216	K-Acetyl	873.46766	437.23747	6
7	955.41563	478.21145	Q	703.36213	352.18470	5
8	1084.45823	542.73275	E	575.30355	288.15541	4
9	1254.56376	627.78552	K-Acetyl	446.26095	223.63411	3
10	1383.60636	692.30682	E	276.15541	138.58134	2
11			K	147.11281	74.06004	1

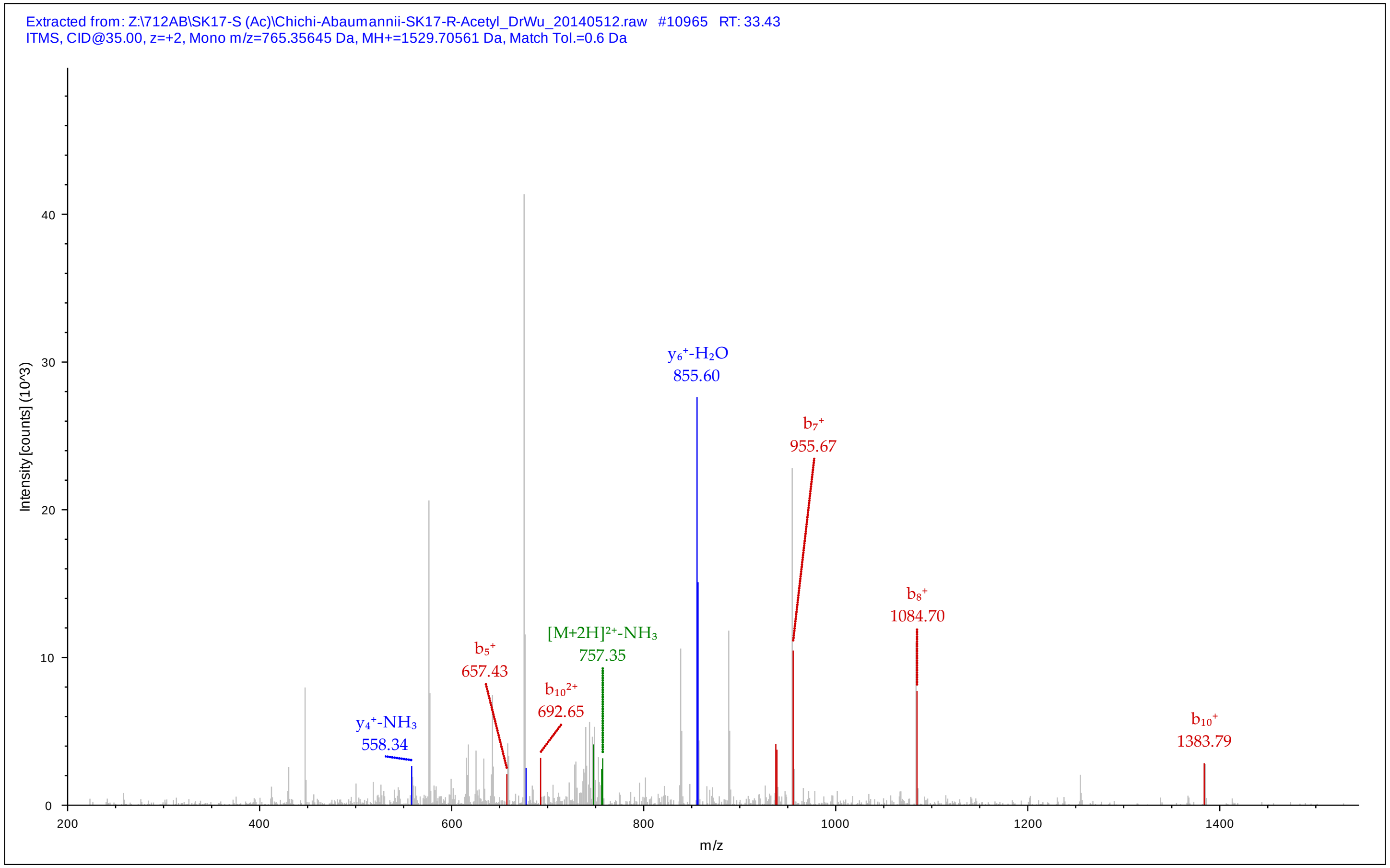

peg.2616   10869

#1	b⁺	b²⁺	Seq.	y⁺	y²⁺	#2
1	158.04479	79.52603	D-Acetyl			11
2	344.12411	172.56569	W	1372.67438	686.84083	10
3	431.15614	216.08171	S	1186.59506	593.80117	9
4	560.19874	280.60301	E	1099.56303	550.28515	8
5	657.25151	329.12939	P	970.52043	485.76385	7
6	827.35705	414.18216	K-Acetyl	873.46766	437.23747	6
7	955.41563	478.21145	Q	703.36213	352.18470	5
8	1084.45823	542.73275	E	575.30355	288.15541	4
9	1254.56376	627.78552	K-Acetyl	446.26095	223.63411	3
10	1383.60636	692.30682	E	276.15541	138.58134	2
11			K	147.11281	74.06004	1

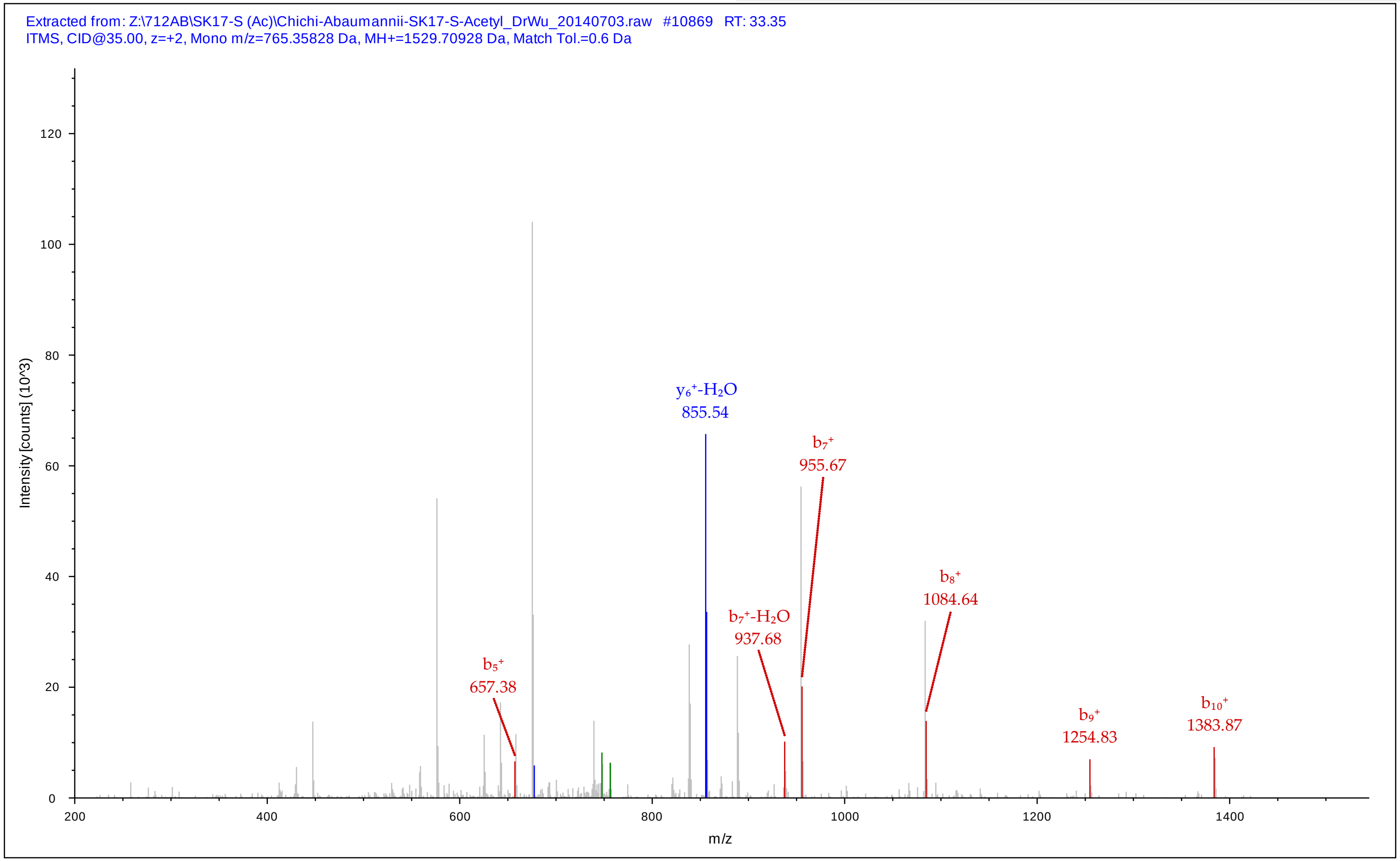

peg.2662 19127

#1	b+	b2+	b3+	Seq.	y+	y2+	y3+	#2
1	114.05496	57.53112	38.68984	A-Acetyl				20
2	243.09756	122.05242	81.70404	E	2367.13268	1184.06998	789.71574	19
3	429.17688	215.09208	143.73048	W	2238.09008	1119.54868	746.70154	18
4	576.21230	288.60979	192.74228	M-Oxidation	2052.01076	1026.50902	684.67510	17
5	713.27121	357.13924	238.42859	H	1904.97534	952.99131	635.66330	16
6	860.33963	430.67345	287.45139	F	1767.91643	884.46185	589.97699	15
7	973.42370	487.21549	325.14608	I	1620.84801	810.92764	540.95419	14
8	1060.45573	530.73150	354.15676	S	1507.76394	754.38561	503.25950	13
9	1131.49285	566.25006	377.83580	A	1420.73191	710.86959	474.24882	12
10	1246.51980	623.76354	416.17812	D	1349.69479	675.35103	450.56978	11
11	1374.61477	687.81102	458.87644	K	1234.66784	617.83756	412.22746	10
12	1445.65189	723.32958	482.55548	A	1106.57287	553.79007	369.52914	9
13	1592.72031	796.86379	531.57829	F	1035.53575	518.27151	345.85010	8
14	1762.82584	881.91656	588.28013	K-Acetyl	888.46733	444.73730	296.82729	7
15	1875.90991	938.45859	625.97482	L	718.36180	359.68454	240.12545	6
16	2004.95251	1002.97989	668.98902	E	605.27773	303.14250	202.43076	5
17	2075.98963	1038.49845	692.66806	A	476.23513	238.62120	159.41656	4
18	2205.03223	1103.01975	735.68226	E	405.19801	203.10264	135.73752	3
19	2292.06426	1146.53577	764.69294	S	276.15541	138.58134	92.72332	2
20				K-Acetyl	189.12338	95.06533	63.71264	1

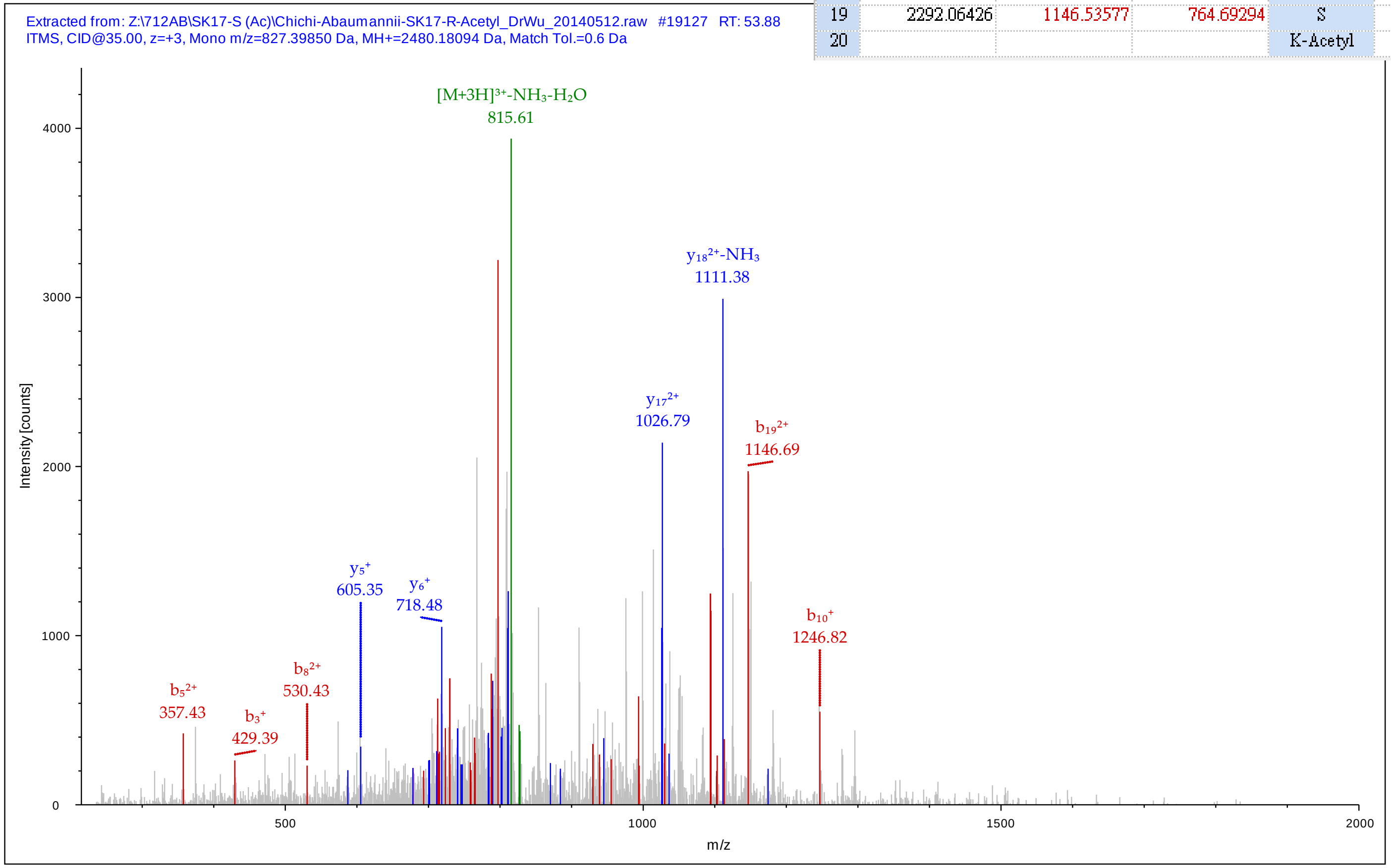

peg.2692 21160

#1	b⁺	b²⁺	Seq.	y⁺	y²⁺	#2
1	100.07570	50.54149	V			11
2	228.13428	114.57078	Q	1172.61581	586.81154	10
3	285.15575	143.08151	G	1044.55723	522.78225	9
4	414.19835	207.60281	E	987.53576	494.27152	8
5	584.30388	292.65558	K-Acetyl	858.49316	429.75022	7
6	655.34100	328.17414	A	688.38762	344.69745	6
7	768.42507	384.71617	L	617.35050	309.17889	5
8	897.46767	449.23747	E	504.26643	252.63685	4
9	1012.49462	506.75095	D	375.22383	188.11555	3
10	1125.57869	563.29298	L	260.19688	130.60208	2
11			K	147.11281	74.06004	1

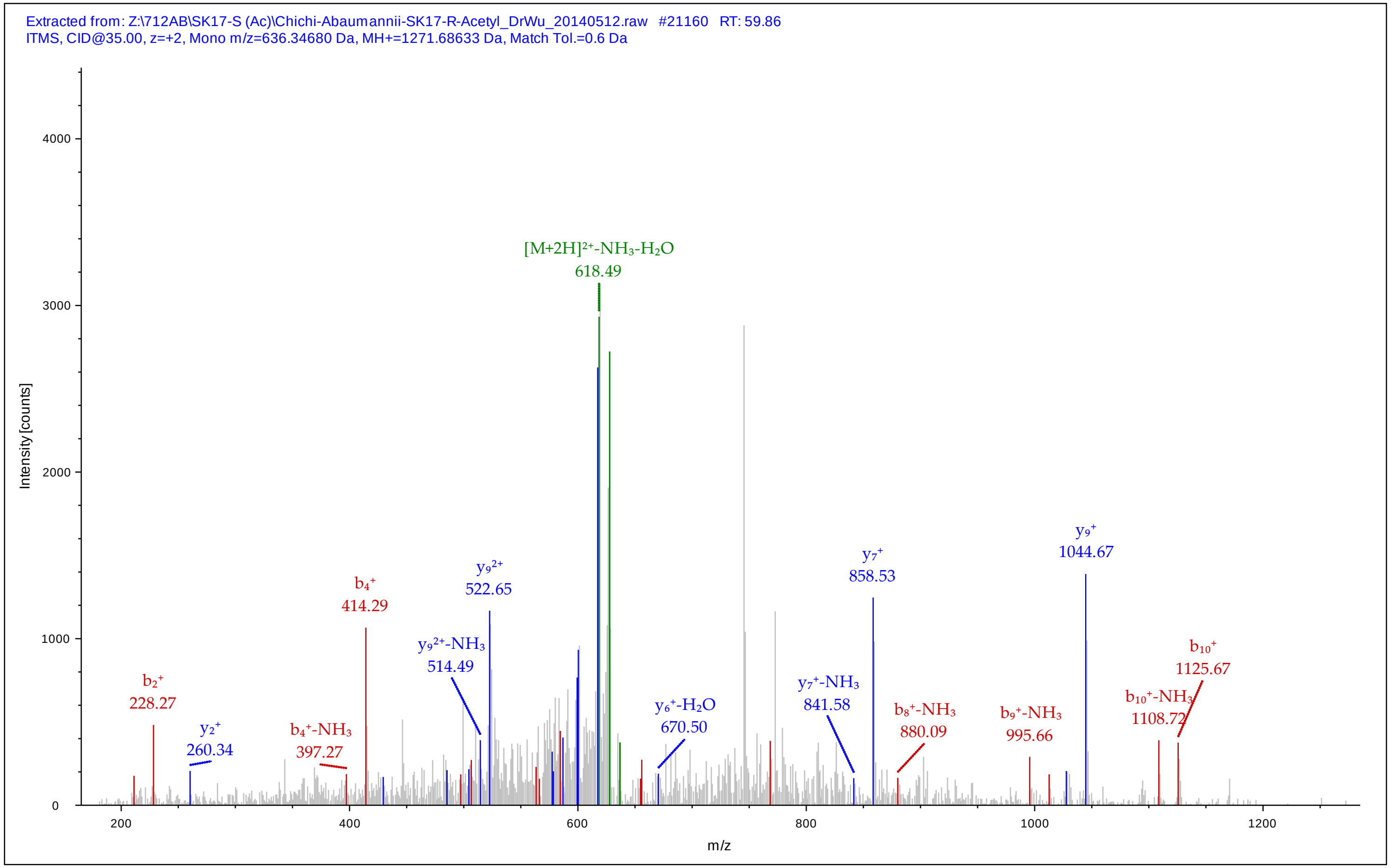

peg.2692   19363

#1	b+	b2+	Seq.	y+	y2+	#2
1	100.07570	50.54149	V			11
2	228.13428	114.57078	Q	1172.61581	586.81154	10
3	285.15575	143.08151	G	1044.55723	522.78225	9
4	414.19835	207.60281	E	987.53576	494.27152	8
5	584.30388	292.65558	K-Acetyl	858.49316	429.75022	7
6	655.34100	328.17414	A	688.38762	344.69745	6
7	768.42507	384.71617	L	617.35050	309.17889	5
8	897.46767	449.23747	E	504.26643	252.63685	4
9	1012.49462	506.75095	D	375.22383	188.11555	3
10	1125.57869	563.29298	L	260.19688	130.60208	2
11			K	147.11281	74.06004	1

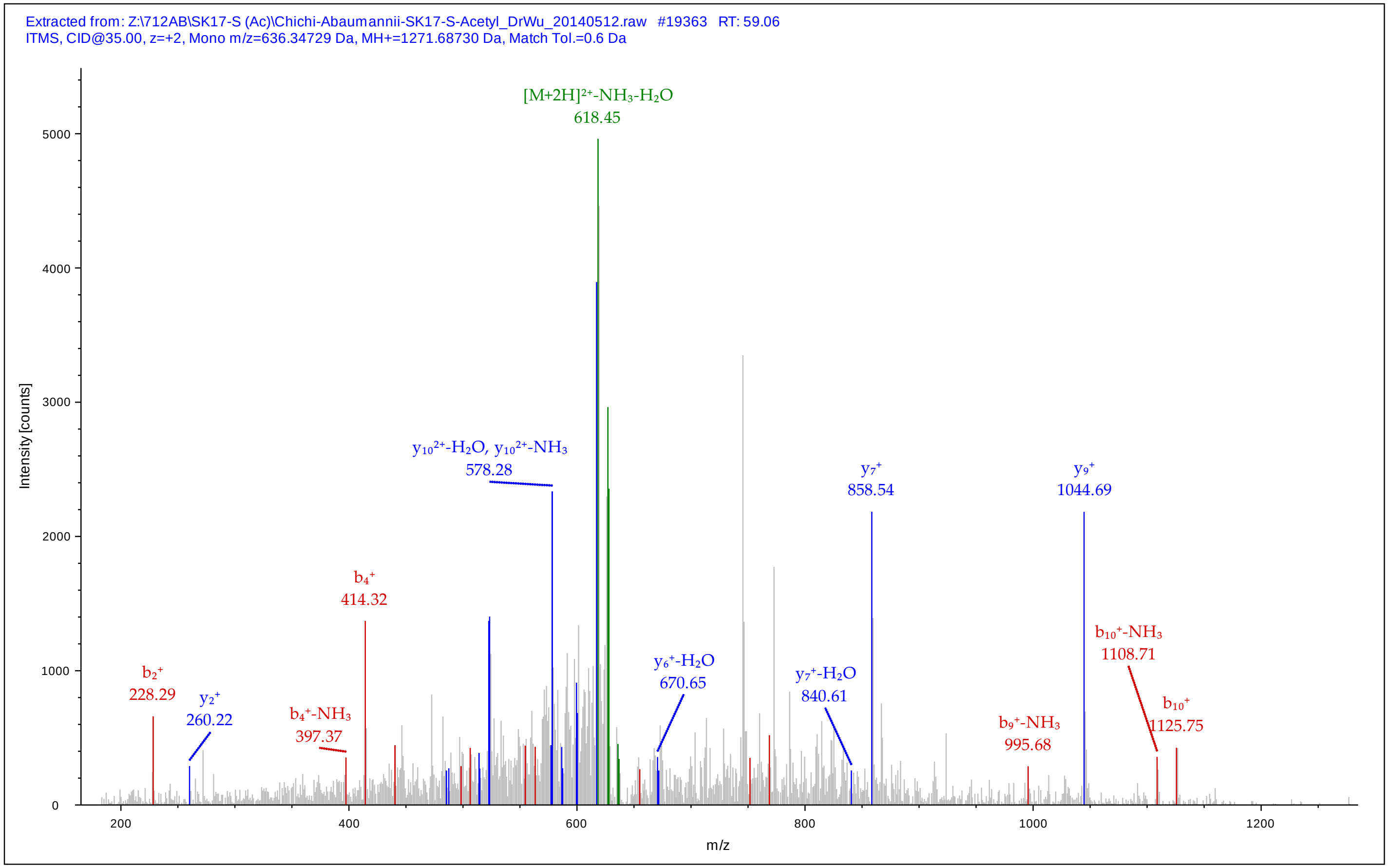

peg.2693   22599

#1	b^{+}	b^{2+}	b^{3+}	Seq.	y^{+}	y^{2+}	y^{3+}	#2
1	130.04988	65.52858	44.02148	E				25
2	201.08700	101.04714	67.70052	A	2703.39245	1352.19986	901.80233	24
3	258.10847	129.55787	86.70767	G	2632.35533	1316.68130	878.12329	23
4	372.15140	186.57934	124.72198	N	2575.33386	1288.17057	859.11614	22
5	485.23547	243.12137	162.41667	I	2461.29093	1231.14910	821.10183	21
6	556.27259	278.63993	186.09571	A	2348.20686	1174.60707	783.40714	20
7	655.34101	328.17414	219.11852	V	2277.16974	1139.08851	759.72810	19
8	811.44213	406.22470	271.15223	R	2178.10132	1089.55430	726.70529	18
9	908.49490	454.75109	303.50315	P	2022.00020	1011.50374	674.67158	17
10	1007.56332	504.28530	336.52596	V	1924.94743	962.97735	642.32066	16
11	1108.61100	554.80914	370.20852	T	1825.87901	913.44314	609.29785	15
12	1236.66958	618.83843	412.89471	Q	1724.83133	862.91930	575.61529	14
13	1349.75365	675.38046	450.58940	L	1596.77275	798.89001	532.92910	13
14	1496.82207	748.91467	499.61221	F	1483.68868	742.34798	495.23441	12
15	1611.84902	806.42815	537.95452	D	1336.62026	668.81377	446.21160	11
16	1708.90179	854.95453	570.30545	P	1221.59331	611.30029	407.86929	10
17	1837.94439	919.47583	613.31965	E	1124.54054	562.77391	375.51836	9
18	2008.04992	1004.52860	670.02149	K-Acetyl	995.49794	498.25261	332.50416	8
19	2065.07139	1033.03933	689.02865	G	825.39240	413.19984	275.80232	7
20	2196.11189	1098.55958	732.70881	M	768.37093	384.68910	256.79516	6
21	2343.18031	1172.09379	781.73162	F	637.33043	319.16885	213.11499	5
22	2456.26438	1228.63583	819.42631	L	490.26201	245.63464	164.09219	4
23	2543.29641	1272.15184	848.43699	S	377.17794	189.09261	126.39750	3
24	2658.32336	1329.66532	886.77930	D	290.14591	145.57659	97.38682	2
25				R	175.11896	88.06312	59.04450	1

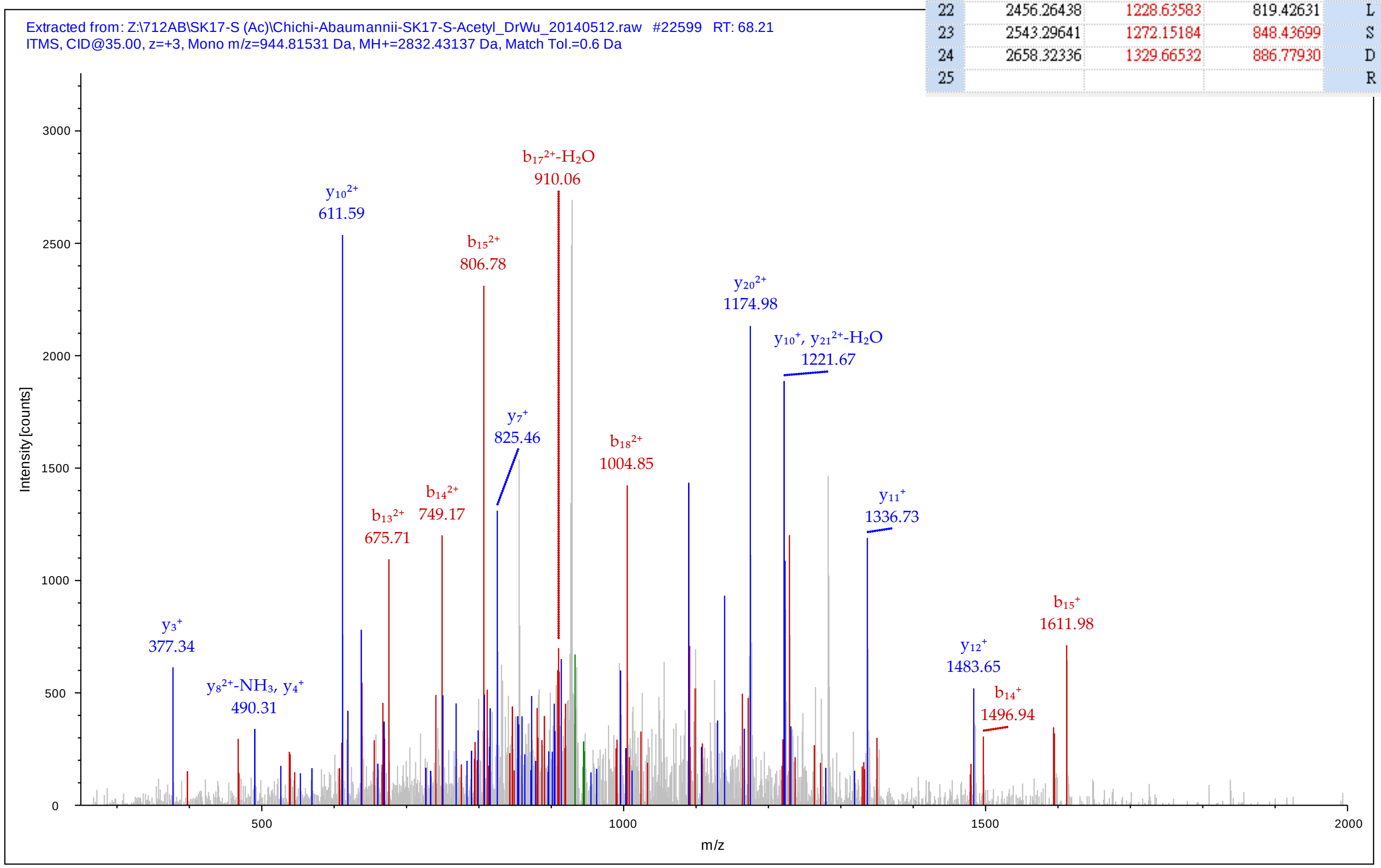

peg.2730 6501

#1	b⁺	b²⁺	b³⁺	Seq.	y⁺	y²⁺	y³⁺	#2
1	130.04988	65.52858	44.02148	E				15
2	229.11830	115.06279	77.04428	V	1711.87966	856.44347	571.29807	14
3	330.16598	165.58663	110.72684	T	1612.81124	806.90926	538.27526	13
4	387.18745	194.09736	129.73400	G	1511.76356	756.38542	504.59270	12
5	500.27152	250.63940	167.42869	L	1454.74209	727.87468	485.58555	11
6	670.37705	335.69216	224.13053	K-Acetyl	1341.65802	671.33265	447.89086	10
7	785.40400	393.20564	262.47285	D	1171.55249	586.27988	391.18901	9
8	898.48807	449.74767	300.16754	L	1056.52554	528.76641	352.84670	8
9	1045.52349	523.26538	349.17935	M-Oxidation	943.44147	472.22437	315.15201	7
10	1173.58207	587.29467	391.86554	Q	796.40605	398.70666	266.14020	6
11	1301.64065	651.32396	434.55173	Q	668.34747	334.67737	223.45401	5
12	1438.69956	719.85342	480.23804	H	540.28889	270.64808	180.76781	4
13	1567.74216	784.37472	523.25224	E	403.22998	202.11863	135.08151	3
14	1666.81058	833.90893	556.27504	V	274.18738	137.59733	92.06731	2
15				R	175.11896	88.06312	59.04450	1

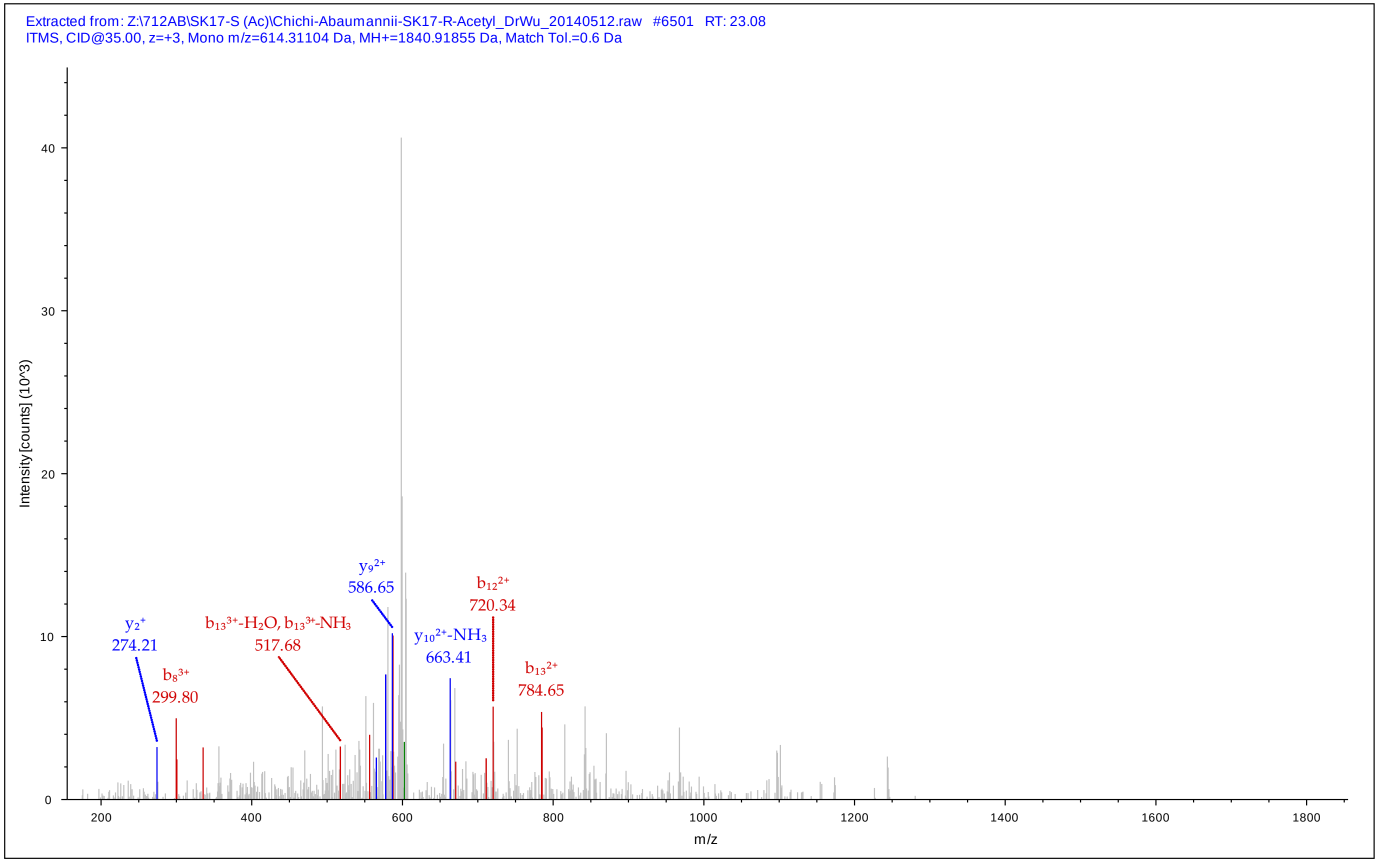

peg.2792 23943

#1	b⁺	b²⁺	b³⁺	Seq.	y⁺	y²⁺	y³⁺	#2
1	148.04269	74.52498	50.01908	M-Oxidation				20
2	263.06964	132.03846	88.36140	D	2136.17725	1068.59226	712.73060	19
3	377.11257	189.05992	126.37571	N	2021.15030	1011.07879	674.38828	18
4	464.14460	232.57594	155.38638	S	1907.10737	954.05732	636.37397	17
5	634.25014	317.62871	212.08823	K-Acetyl	1820.07534	910.54131	607.36330	16
6	747.33421	374.17074	249.78292	L	1649.96980	825.48854	550.66145	15
7	844.38698	422.69713	282.13384	P	1536.88573	768.94650	512.96676	14
8	957.47105	479.23916	319.82853	I	1439.83296	720.42012	480.61584	13
9	1071.51398	536.26063	357.84284	N	1326.74889	663.87808	442.92115	12
10	1199.57256	600.28992	400.52904	Q	1212.70596	606.85662	404.90684	11
11	1312.65663	656.83195	438.22373	I	1084.64738	542.82733	362.22064	10
12	1425.74070	713.37399	475.91842	I	971.56331	486.28529	324.52595	9
13	1496.77782	748.89255	499.59746	A	858.47924	429.74326	286.83126	8
14	1652.87894	826.94311	551.63116	R	787.44212	394.22470	263.15222	7
15	1765.96301	883.48514	589.32585	I	631.34100	316.17414	211.11852	6
16	1880.00594	940.50661	627.34016	N	518.25693	259.63210	173.42383	5
17	1995.03289	998.02008	665.68248	D	404.21400	202.61064	135.40952	4
18	2066.07001	1033.53864	689.36152	A	289.18705	145.09716	97.06720	3
19	2137.10713	1069.05720	713.04056	A	218.14993	109.57860	73.38816	2
20				K	147.11281	74.06004	49.70912	1

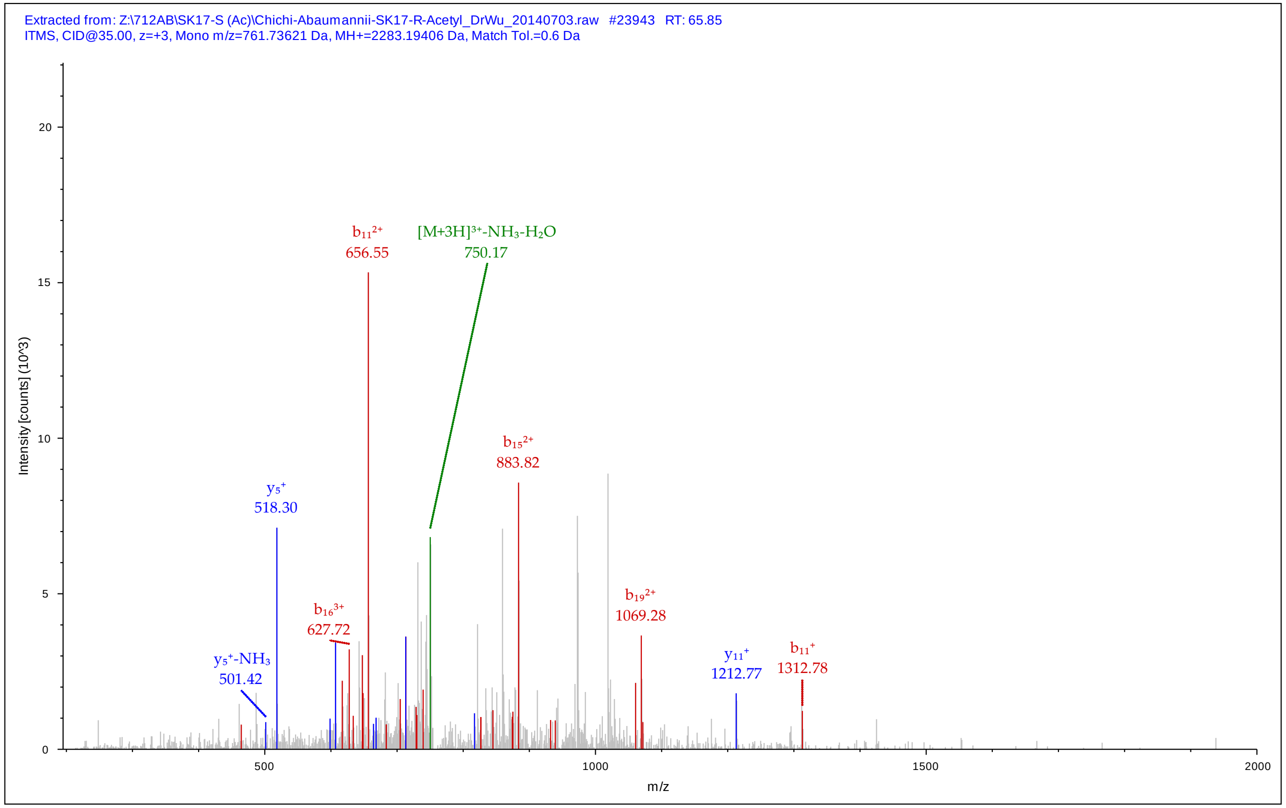

peg.2802 15219

#1	b+	b2+	b3+	Seq.	y+	y2+	y3+	#2
1	114.05496	57.53112	38.68984	A-Acetyl				13
2	243.09756	122.05242	81.70404	E	1586.80748	793.90738	529.60734	12
3	356.18163	178.59445	119.39873	L	1457.76488	729.38608	486.59314	11
4	542.26095	271.63411	181.42517	W	1344.68081	672.84404	448.89845	10
5	629.29298	315.15013	210.43584	S	1158.60149	579.80438	386.87201	9
6	815.37230	408.18979	272.46228	W	1071.56946	536.28837	357.86134	8
7	971.47342	486.24035	324.49599	R	885.49014	443.24871	295.83490	7
8	1141.57896	571.29312	381.19784	K-Acetyl	729.38902	365.19815	243.80119	6
9	1242.62664	621.81696	414.88040	T	559.28348	280.14538	187.09934	5
10	1339.67941	670.34334	447.23132	P	458.23580	229.62154	153.41678	4
11	1468.72201	734.86464	490.24552	E	361.18303	181.09515	121.06586	3
12	1525.74348	763.37538	509.25268	G	232.14043	116.57385	78.05166	2
13				R	175.11896	88.06312	59.04450	1

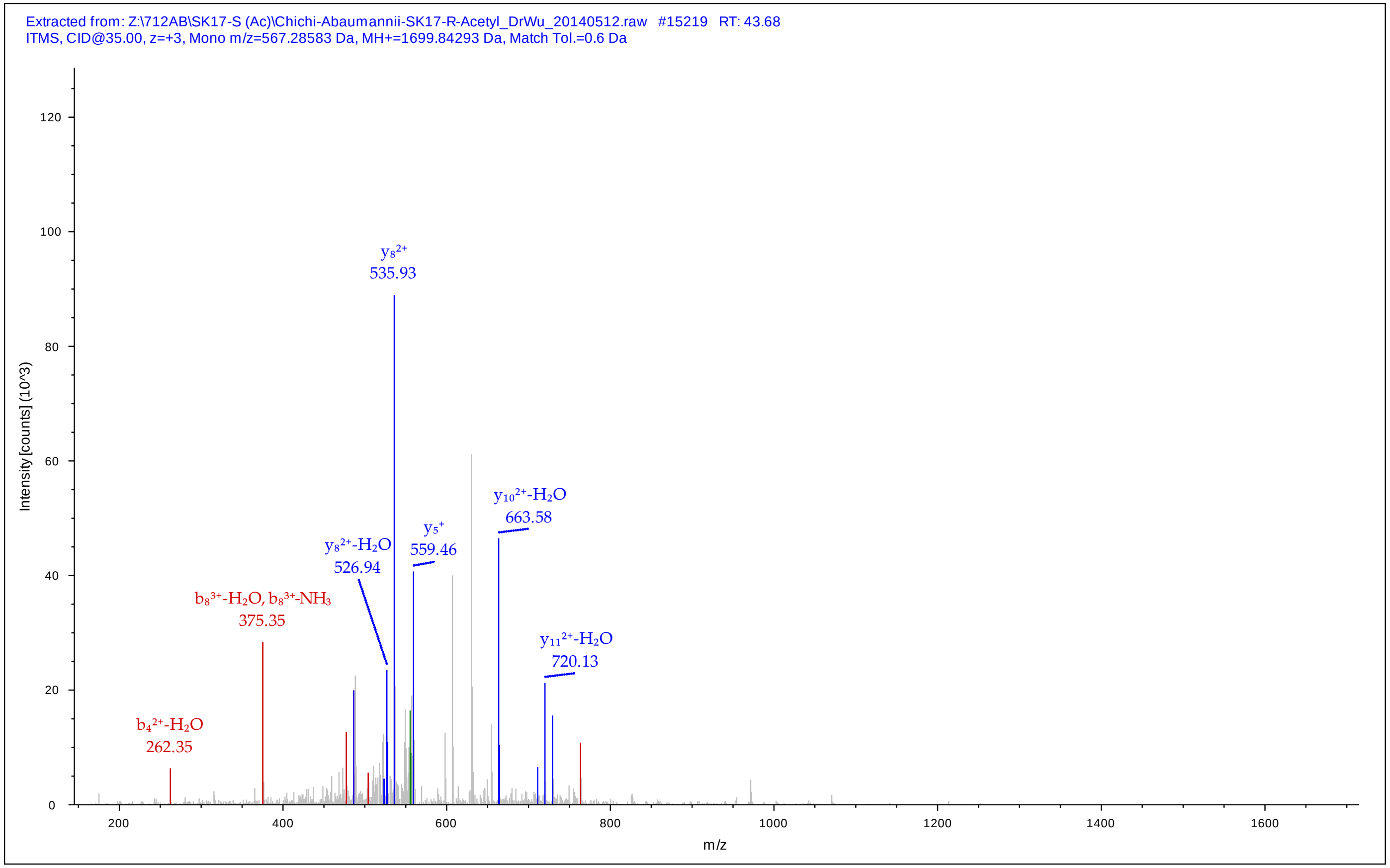

peg.2807   26169

#1	b⁺	b²⁺	b³⁺	Seq.	y⁺	y²⁺	y³⁺	#2
1	129.06586	65.03657	43.69347	Q				21
2	258.10846	129.55787	86.70767	E	2211.08976	1106.04852	737.70144	20
3	371.19253	186.09990	124.40236	L	2082.04716	1041.52722	694.68724	19
4	486.21948	243.61338	162.74468	D	1968.96309	984.98518	656.99255	18
5	599.30355	300.15541	200.43937	I	1853.93614	927.47171	618.65023	17
6	728.34615	364.67671	243.45357	E	1740.85207	870.92967	580.95554	16
7	898.45168	449.72948	300.15541	K-Acetyl	1611.80947	806.40837	537.94134	15
8	1001.46087	501.23407	334.49181	C	1441.70394	721.35561	481.23950	14
9	1058.48234	529.74481	353.49896	G	1338.69475	669.85101	446.90310	13
10	1157.55076	579.27902	386.52177	V	1281.67328	641.34028	427.89594	12
11	1244.58279	622.79503	415.53245	S	1182.60486	591.80607	394.87314	11
12	1331.61482	666.31105	444.54312	S	1095.57283	548.29005	365.86246	10
13	1444.69889	722.85308	482.23781	L	1008.54080	504.77404	336.85178	9
14	1604.72955	802.86841	535.58137	C-Carbam···	895.45673	448.23200	299.15709	8
15	1703.79797	852.40262	568.60417	V	735.42607	368.21667	245.81354	7
16	1840.85688	920.93208	614.29048	H	636.35765	318.68246	212.79073	6
17	1937.90965	969.45846	646.64140	P	499.29874	250.15301	167.10443	5
18	1994.93112	997.96920	665.64856	G	402.24597	201.62662	134.75351	4
19	2051.95259	1026.47993	684.65571	G	345.22450	173.11589	115.74635	3
20	2165.03666	1083.02197	722.35040	I	288.20303	144.60515	96.73919	2
21				R	175.11896	88.06312	59.04450	1

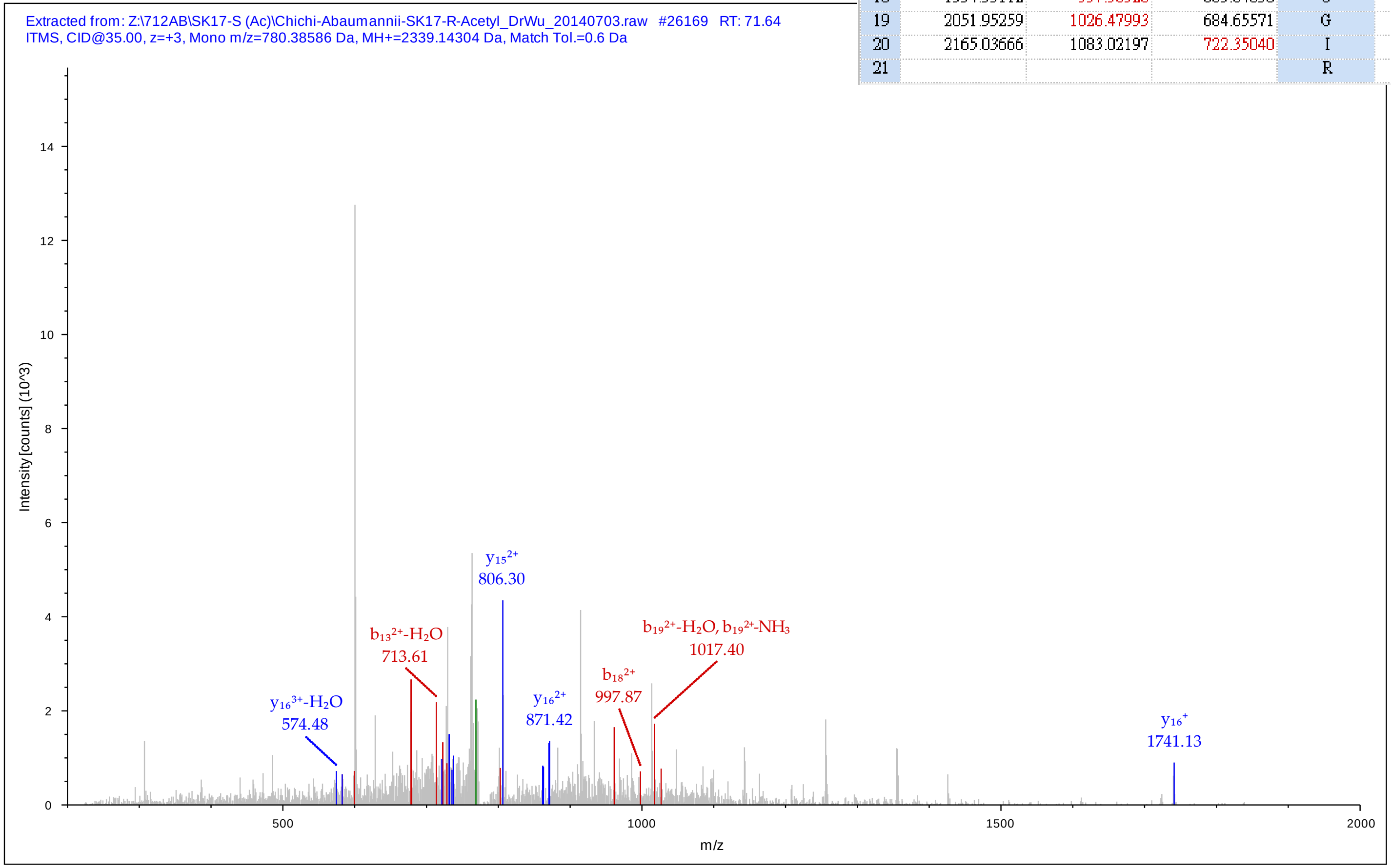

peg.2836 16373

#1	b+	b2+	Seq.	y+	y2+	#2
1	148.07570	74.54149	F			11
2	279.11620	140.06174	M	1217.61951	609.31339	10
3	376.16897	188.58812	P	1086.57901	543.79314	9
4	447.20609	224.10668	A	989.52624	495.26676	8
5	617.31162	309.15945	K-Acetyl	918.48912	459.74820	7
6	718.35930	359.68329	T	748.38359	374.69543	6
7	888.46484	444.73606	K-Acetyl	647.33591	324.17159	5
8	1017.50744	509.25736	E	477.23037	239.11882	4
9	1131.55037	566.27882	N	348.18777	174.59752	3
10	1218.58240	609.79484	S	234.14484	117.57606	2
11			K	147.11281	74.06004	1

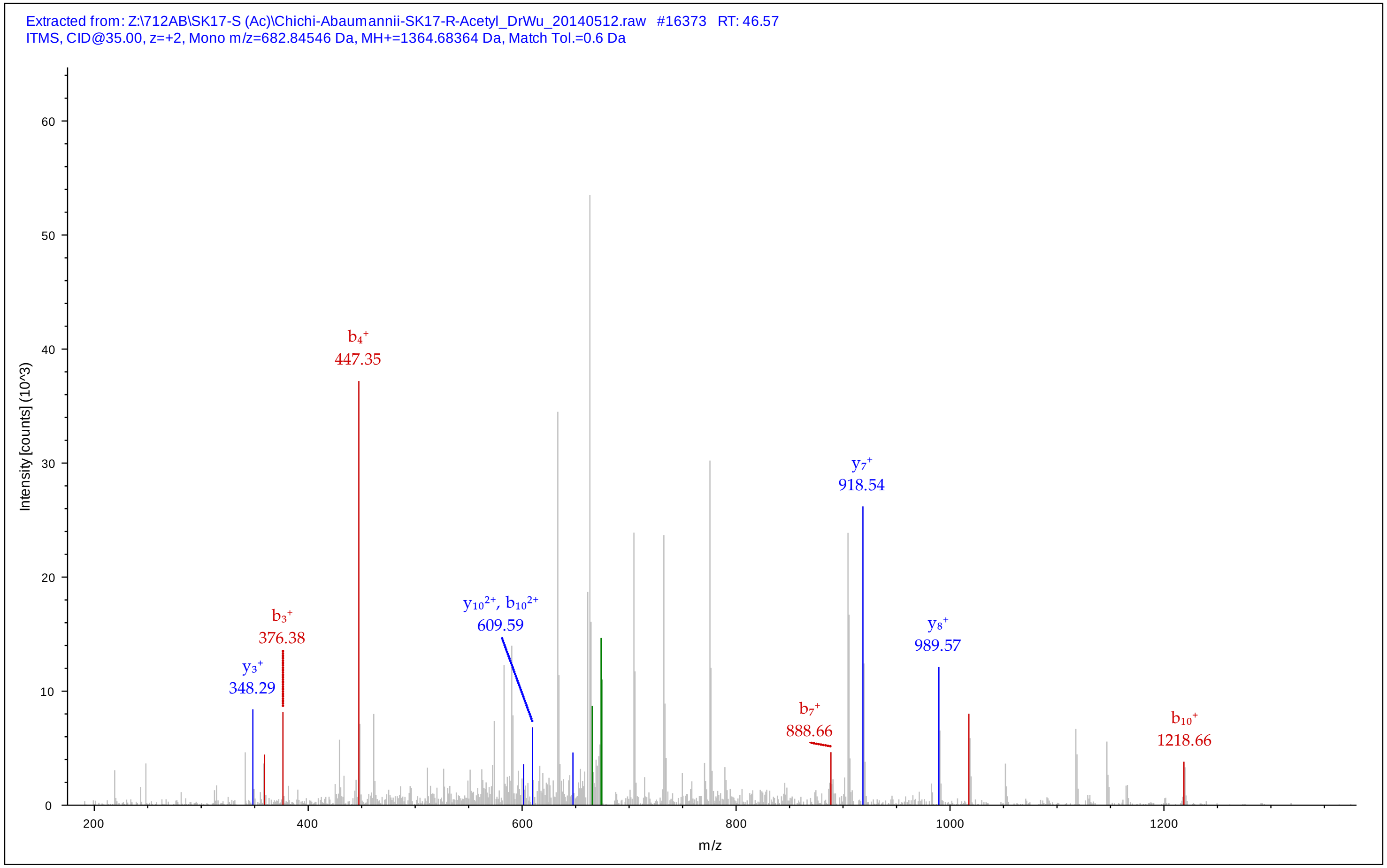

peg.2836 14570

#1	b⁺	b²⁺	Seq.	y⁺	y²⁺	#2
1	148.07570	74.54149	F			11
2	279.11620	140.06174	M	1217.61951	609.31339	10
3	376.16897	188.58812	P	1086.57901	543.79314	9
4	447.20609	224.10668	A	989.52624	495.26676	8
5	617.31162	309.15945	K-Acetyl	918.48912	459.74820	7
6	718.35930	359.68329	T	748.38359	374.69543	6
7	888.46484	444.73606	K-Acetyl	647.33591	324.17159	5
8	1017.50744	509.25736	E	477.23037	239.11882	4
9	1131.55037	566.27882	N	348.18777	174.59752	3
10	1218.58240	609.79484	S	234.14484	117.57606	2
11			K	147.11281	74.06004	1

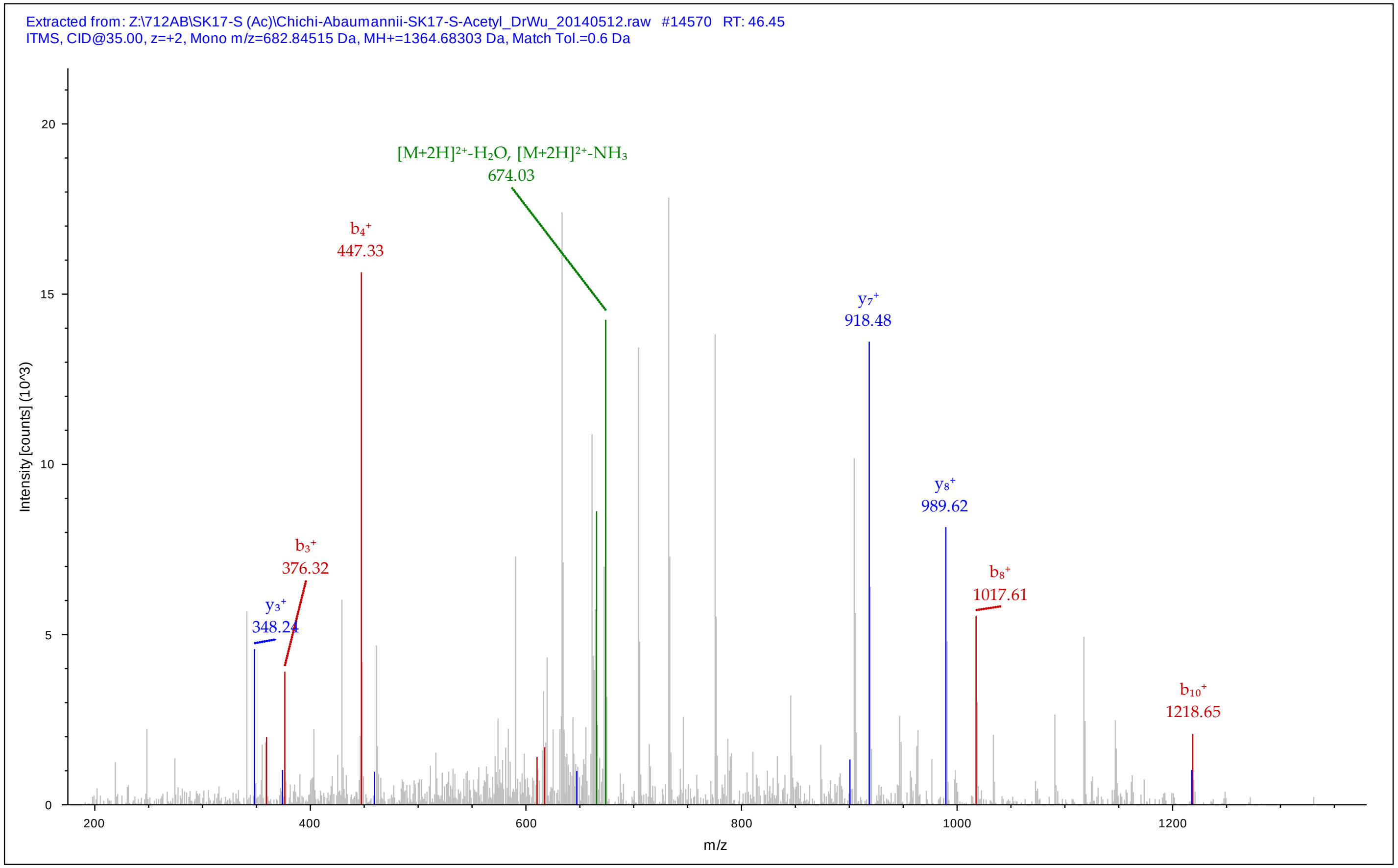

peg.2836   9875

#1	b^+	b^{2+}	b^{3+}	Seq.	y^+	y^{2+}	y^{3+}	#2
1	142.08626	71.54677	48.03360	V-Acetyl				26
2	245.09545	123.05136	82.37000	C	2954.30533	1477.65630	985.43996	25
3	374.13805	187.57266	125.38420	E	2851.29614	1426.15171	951.10356	24
4	502.19663	251.60195	168.07039	Q	2722.25354	1361.63041	908.08936	23
5	559.21810	280.11269	187.07755	G	2594.19496	1297.60112	865.40317	22
6	722.28142	361.64435	241.43199	Y	2537.17349	1269.09038	846.39601	21
7	851.32402	426.16565	284.44619	E	2374.11017	1187.55872	792.04157	20
8	1007.42514	504.21621	336.47990	R	2245.06757	1123.03742	749.02737	19
9	1104.47791	552.74259	368.83082	P	2088.96645	1044.98686	696.99367	18
10	1219.50486	610.25607	407.17314	D	1991.91368	996.46048	664.64274	17
11	1332.58893	666.79810	444.86783	I	1876.88673	938.94700	626.30043	16
12	1479.62435	740.31581	493.87963	M-Oxidation	1763.80266	882.40497	588.60574	15
13	1626.69277	813.85002	542.90244	F	1616.76725	808.88726	539.59393	14
14	1713.72480	857.36604	571.91312	S	1469.69883	735.35305	490.57113	13
15	1850.78371	925.89549	617.59942	H	1382.66680	691.83704	461.56045	12
16	2020.88924	1010.94826	674.30126	K-Acetyl	1245.60789	623.30758	415.87415	11
17	2157.94815	1079.47771	719.98757	H	1075.50235	538.25481	359.17230	10
18	2271.99108	1136.49918	758.00188	N	938.44344	469.72536	313.48600	9
19	2329.01255	1165.00991	777.00903	G	824.40051	412.70389	275.47169	8
20	2430.06023	1215.53375	810.69159	T	767.37904	384.19316	256.46453	7
21	2543.14430	1272.07579	848.38628	I	666.33136	333.66932	222.78197	6
22	2646.15349	1323.58038	882.72268	C	553.24729	277.12728	185.08728	5
23	2733.18552	1367.09640	911.73336	S	450.23810	225.62269	150.75088	4
24	2846.26959	1423.63843	949.42805	L	363.20607	182.10667	121.74021	3
25	2949.27878	1475.14303	983.76444	C	250.12200	125.56464	84.04552	2
26				K	147.11281	74.06004	49.70912	1

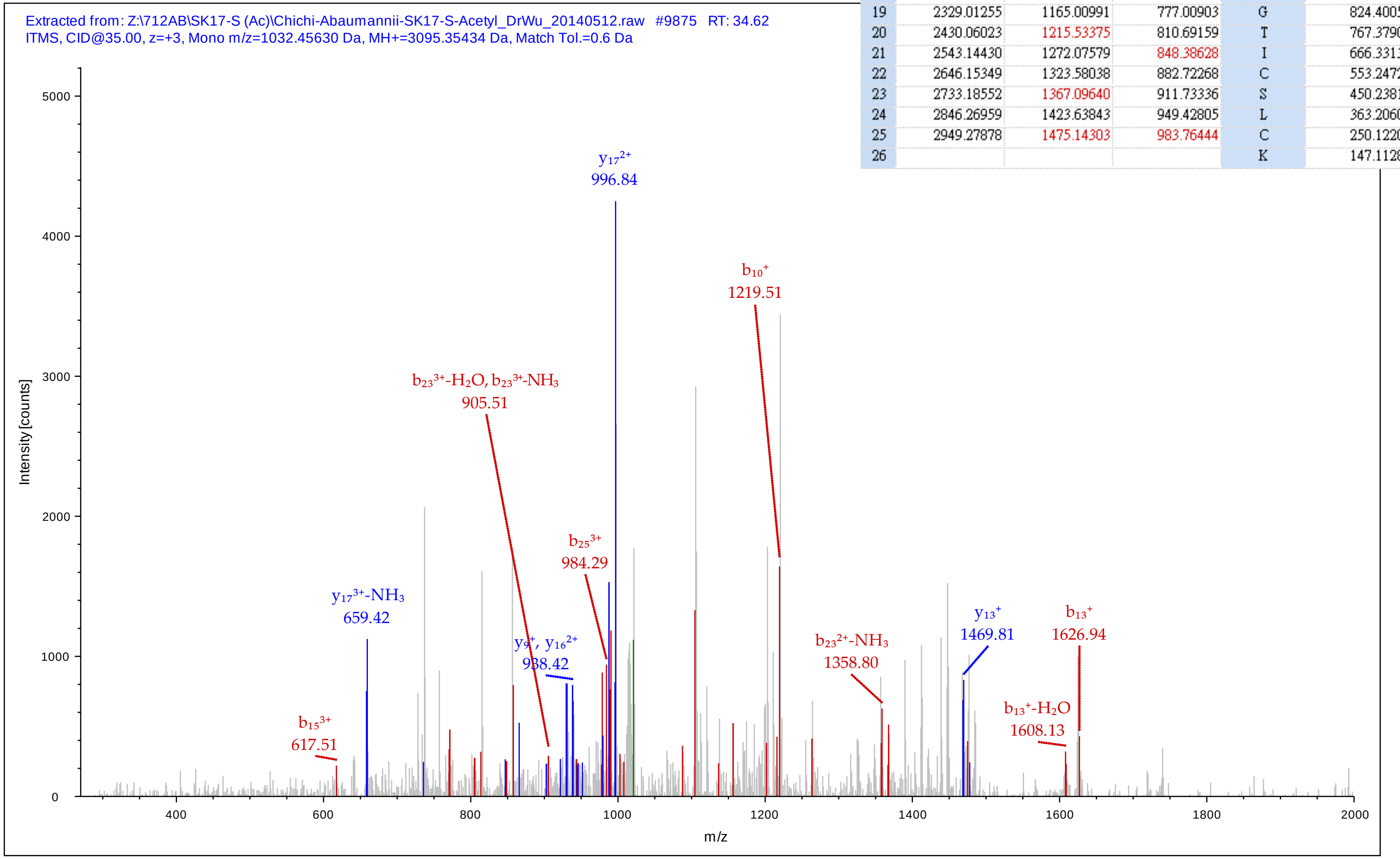

peg.2845 17815

#1	b+	b2+	Seq.	y+	y2+	#2
1	146.02703	73.51715	C-Acetyl			15
2	316.13257	158.56992	K-Acetyl	1645.87652	823.44190	14
3	429.21664	215.11196	L	1475.77098	738.38913	13
4	576.25205	288.62966	M-Oxidation	1362.68691	681.84709	12
5	677.29973	339.15350	T	1215.65150	608.32939	11
6	748.33685	374.67206	A	1114.60382	557.80555	10
7	805.35832	403.18280	G	1043.56670	522.28699	9
8	975.46386	488.23557	K-Acetyl	986.54523	493.77625	8
9	1074.53228	537.76978	V	816.43969	408.72348	7
10	1131.55375	566.28051	G	717.37127	359.18927	6
11	1262.59425	631.80076	M	660.34980	330.67854	5
12	1391.63685	696.32206	E	529.30930	265.15829	4
13	1547.73797	774.37262	R	400.26670	200.63699	3
14	1644.79074	822.89901	P	244.16558	122.58643	2
15			K	147.11281	74.06004	1

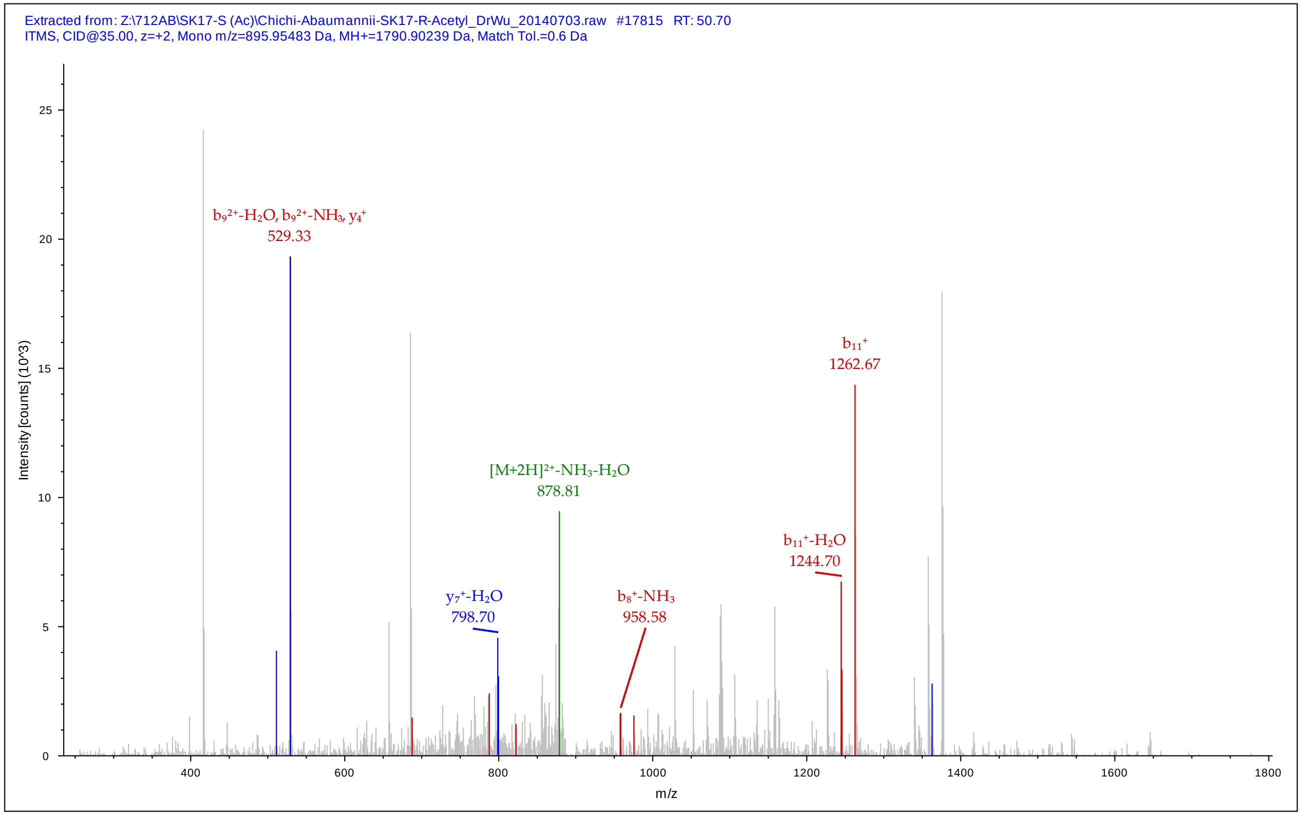

peg.2851 17524

#1	b+	b2+	Seq.	y+	y2+	#2
1	146.02703	73.51715	C-Acetyl			15
2	316.13257	158.56992	K-Acetyl	1645.87652	823.44190	14
3	429.21664	215.11196	L	1475.77098	738.38913	13
4	576.25205	288.62966	M-Oxidation	1362.68691	681.84709	12
5	677.29973	339.15350	T	1215.65150	608.32939	11
6	748.33685	374.67206	A	1114.60382	557.80555	10
7	805.35832	403.18280	G	1043.56670	522.28699	9
8	975.46386	488.23557	K-Acetyl	986.54523	493.77625	8
9	1074.53228	537.76978	V	816.43969	408.72348	7
10	1131.55375	566.28051	G	717.37127	359.18927	6
11	1262.59425	631.80076	M	660.34980	330.67854	5
12	1391.63685	696.32206	E	529.30930	265.15829	4
13	1547.73797	774.37262	R	400.26670	200.63699	3
14	1644.79074	822.89901	P	244.16558	122.58643	2
15			K	147.11281	74.06004	1

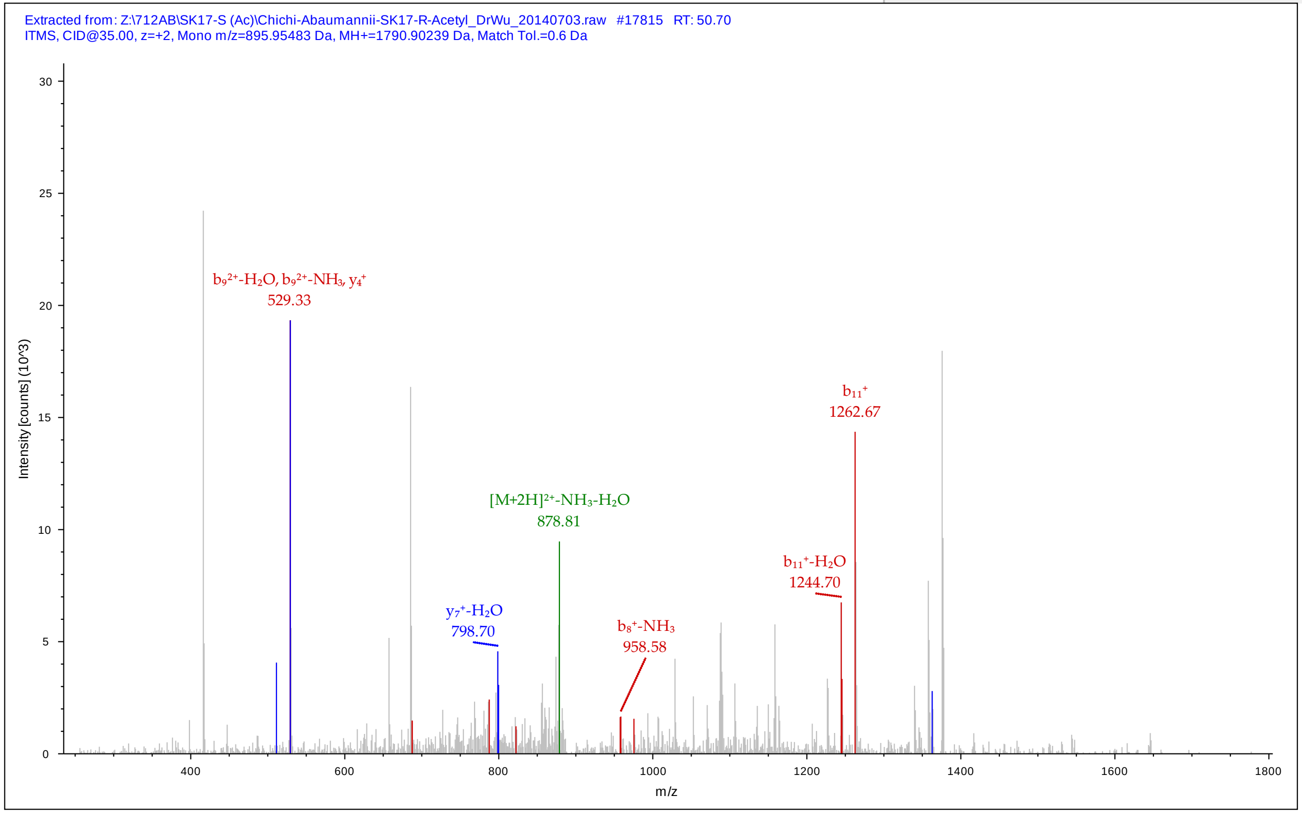

peg.2868   15652

#1	b⁺	b²⁺	Seq.	y⁺	y²⁺	#2
1	114.05496	57.53112	A-Acetyl			7
2	284.16050	142.58389	K-Acetyl	770.52473	385.76600	6
3	397.24457	199.12592	L	600.41919	300.71323	5
4	525.33954	263.17341	K	487.33512	244.17120	4
5	596.37666	298.69197	A	359.24015	180.12371	3
6	709.46073	355.23400	L	288.20303	144.60515	2
7			R	175.11896	88.06312	1

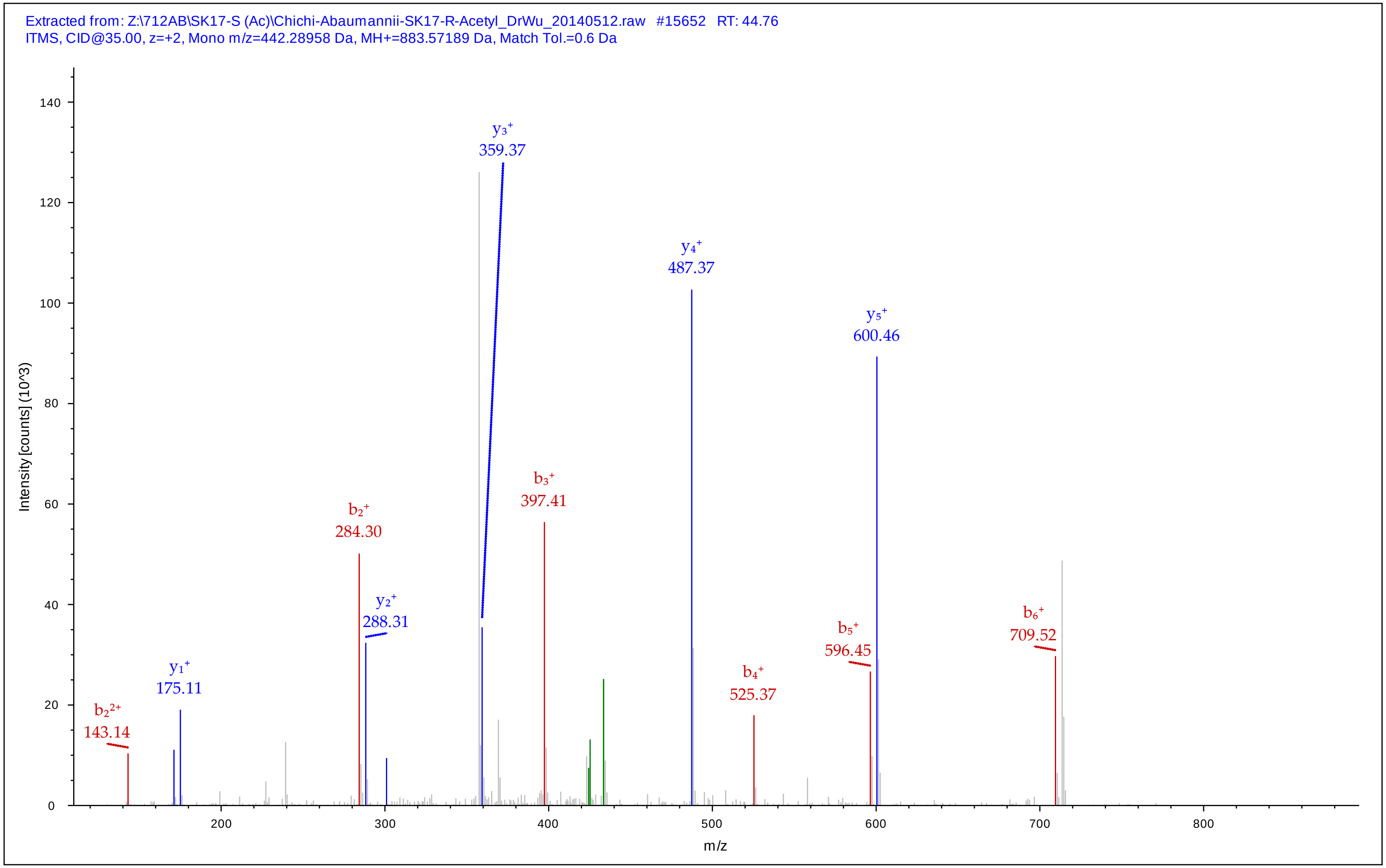

peg.2868   13921

#1	b⁺	b²⁺	Seq.	y⁺	y²⁺	#2
1	114.05496	57.53112	A-Acetyl			7
2	284.16050	142.58389	K-Acetyl	770.52473	385.76600	6
3	397.24457	199.12592	L	600.41919	300.71323	5
4	525.33954	263.17341	K	487.33512	244.17120	4
5	596.37666	298.69197	A	359.24015	180.12371	3
6	709.46073	355.23400	L	288.20303	144.60515	2
7			R	175.11896	88.06312	1

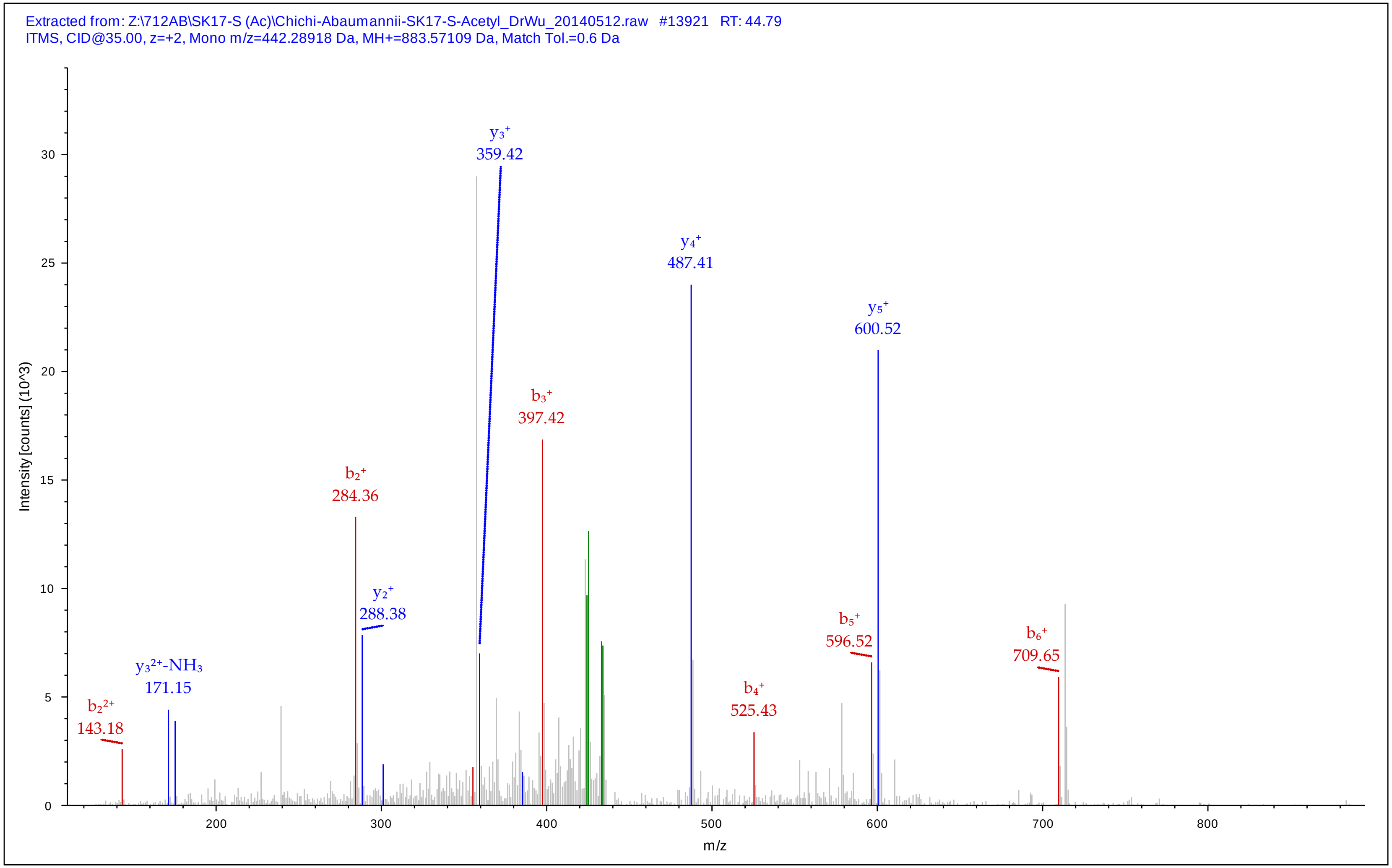

peg.2897 9754

#1	b+	b2+	b3+	Seq.	y+	y2+	y3+	#2
1	132.04778	66.52753	44.68744	M				10
2	245.13185	123.06956	82.38213	L	1096.68376	548.84552	366.23277	9
3	373.19043	187.09885	125.06833	Q	983.59969	492.30348	328.53808	8
4	543.29596	272.15162	181.77017	K-Acetyl	855.54111	428.27419	285.85189	7
5	642.36438	321.68583	214.79298	V	685.43557	343.22142	229.15004	6
6	755.44845	378.22786	252.48767	L	586.36715	293.68721	196.12723	5
7	868.53252	434.76990	290.18236	I	473.28308	237.14518	158.43254	4
8	939.56964	470.28846	313.86140	A	360.19901	180.60314	120.73785	3
9	1053.61257	527.30992	351.87571	N	289.16189	145.08458	97.05881	2
10				R	175.11896	88.06312	59.04450	1

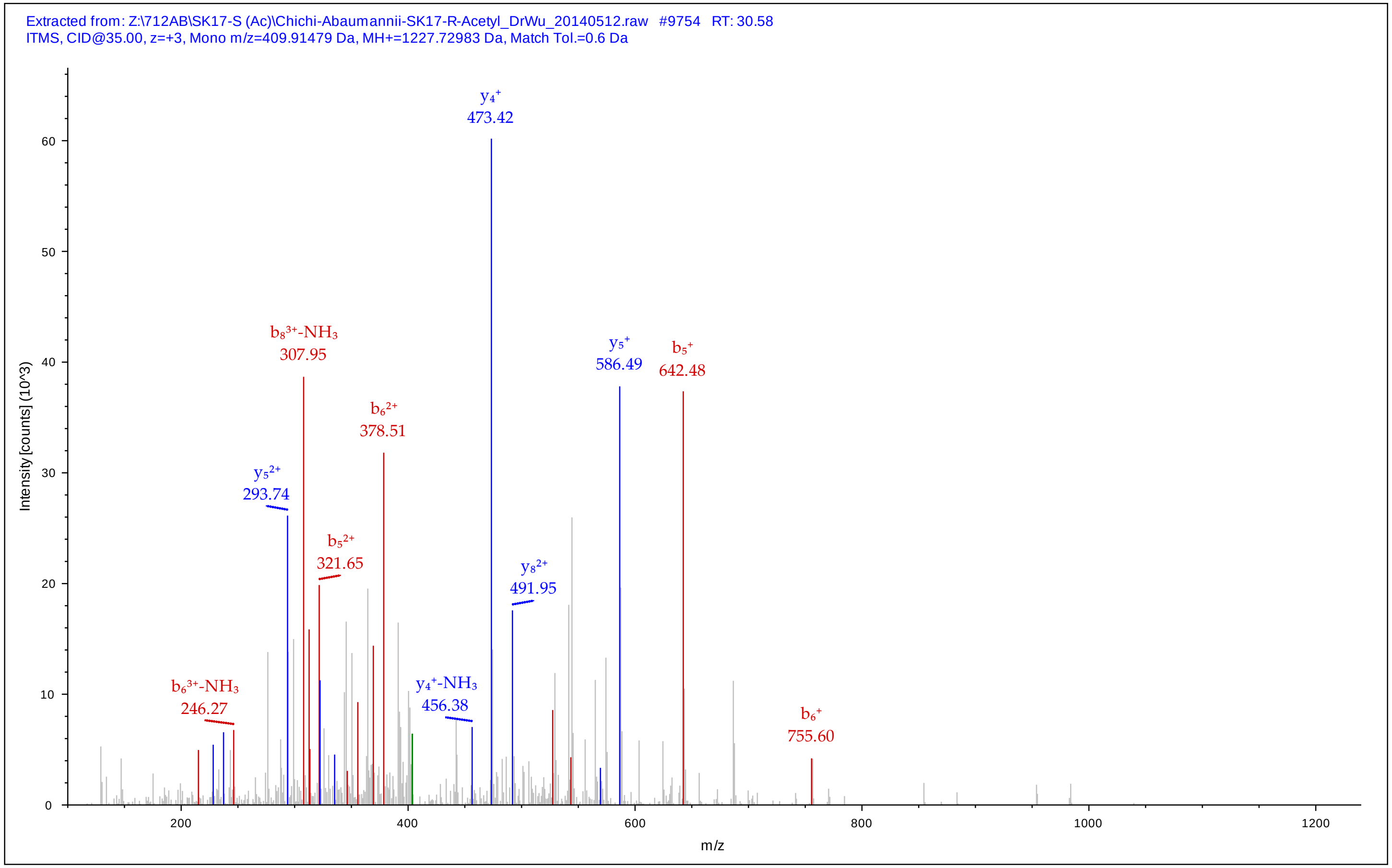

peg.2904 7656

#1	b⁺	b²⁺	Seq.	y⁺	y²⁺	#2
1	171.11281	86.06004	K-Acetyl			7
2	242.14993	121.57860	A	604.33010	302.66869	6
3	299.17140	150.08934	G	533.29298	267.15013	5
4	412.25547	206.63137	L	476.27151	238.63939	4
5	527.28242	264.14485	X-D	363.18744	182.09736	3
6	628.33010	314.66869	T	248.16049	124.58388	2
7			K	147.11281	74.06004	1

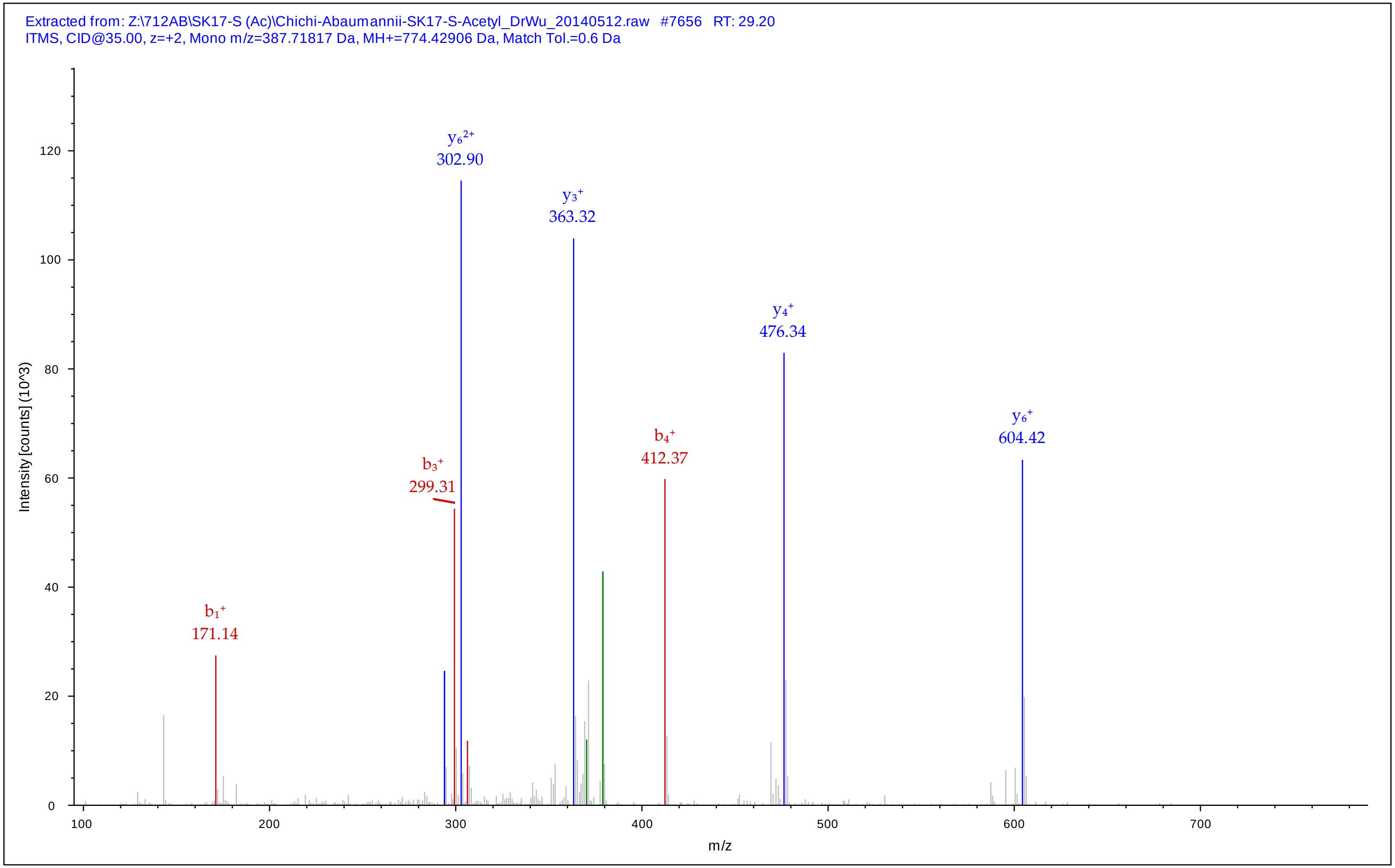

peg.2920 27586

#1	b⁺	b²⁺	Seq.	y⁺	y²⁺	#2
1	174.05834	87.53281	M-Acetyl			19
2	231.07981	116.04354	G	1990.02410	995.51569	18
3	378.14823	189.57775	F	1933.00263	967.00495	17
4	492.19116	246.59922	N	1785.93421	893.47074	16
5	606.23409	303.62068	N	1671.89128	836.44928	15
6	721.26104	361.13416	D	1557.84835	779.42781	14
7	778.28251	389.64489	G	1442.82140	721.91434	13
8	877.35093	439.17910	V	1385.79993	693.40360	12
9	992.37788	496.69258	D	1286.73151	643.86939	11
10	1162.48342	581.74535	K-Acetyl	1171.70456	586.35592	10
11	1275.56749	638.28738	L	1001.59902	501.30315	9
12	1388.65156	694.82942	I	888.51495	444.76111	8
13	1517.69416	759.35072	E	775.43088	388.21908	7
14	1631.73709	816.37218	N	646.38828	323.69778	6
15	1730.80551	865.90639	V	532.34535	266.67631	5
16	1858.90048	929.95388	K	433.27693	217.14210	4
17	1929.93760	965.47244	A	305.18196	153.09462	3
18	2016.96963	1008.98845	S	234.14484	117.57606	2
19			K	147.11281	74.06004	1

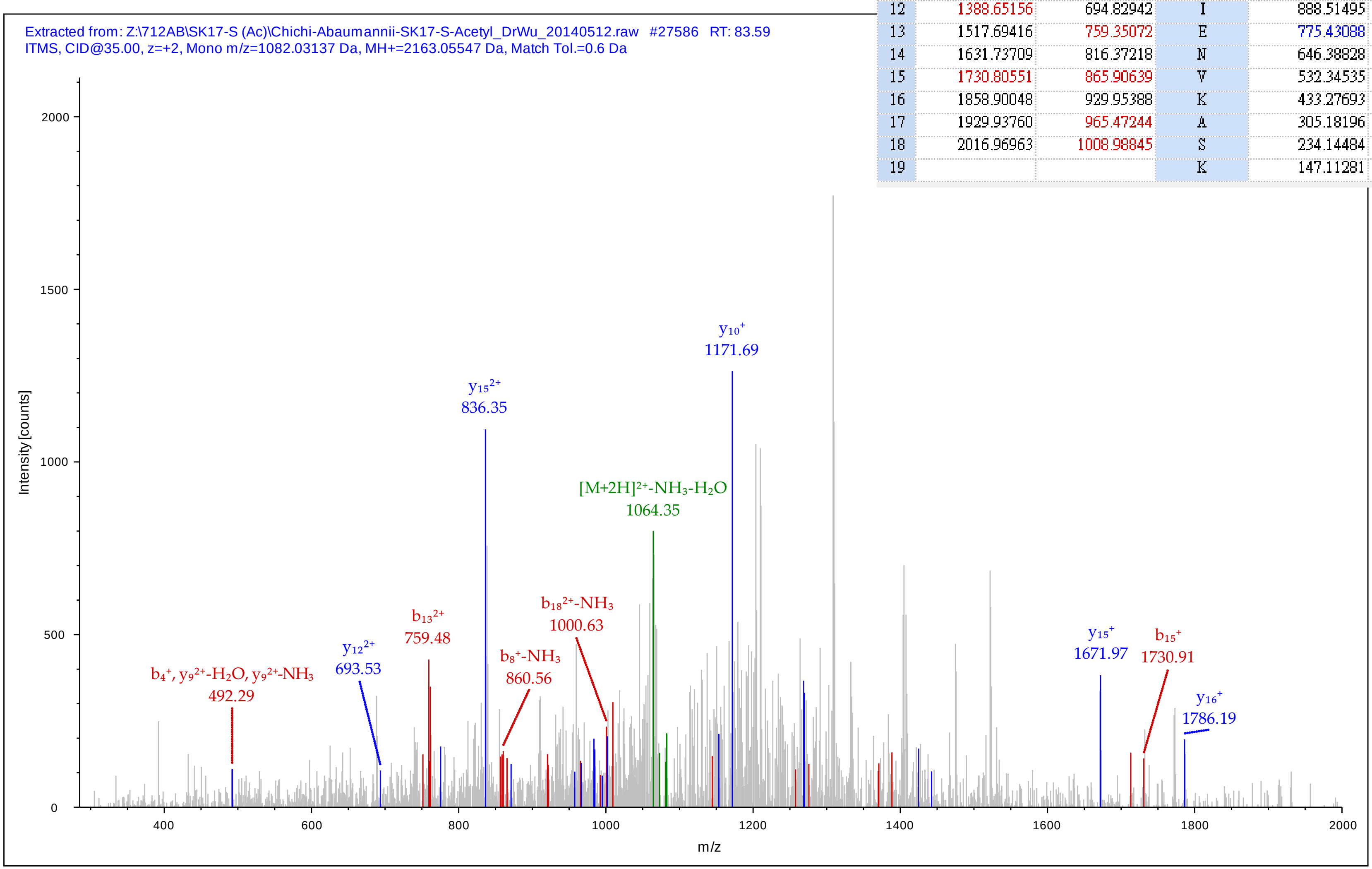

peg.2933 28735

#1	b+	b2+	Seq.	y+	y2+	#2
1	129.06586	65.03657	Q			12
2	228.13428	114.57078	V	1186.66784	593.83756	11
3	343.16123	172.08425	D	1087.59942	544.30335	10
4	456.24530	228.62629	L	972.57247	486.78987	9
5	527.28242	264.14485	A	859.48840	430.24784	8
6	640.36649	320.68688	L	788.45128	394.72928	7
7	711.40361	356.20544	A	675.36721	338.18724	6
8	812.45129	406.72928	T	604.33009	302.66868	5
9	869.47276	435.24002	G	503.28241	252.14484	4
10	1039.57829	520.29278	K-Acetyl	446.26094	223.63411	3
11	1126.61032	563.80880	S	276.15541	138.58134	2
12			K-Acetyl	189.12338	95.06533	1

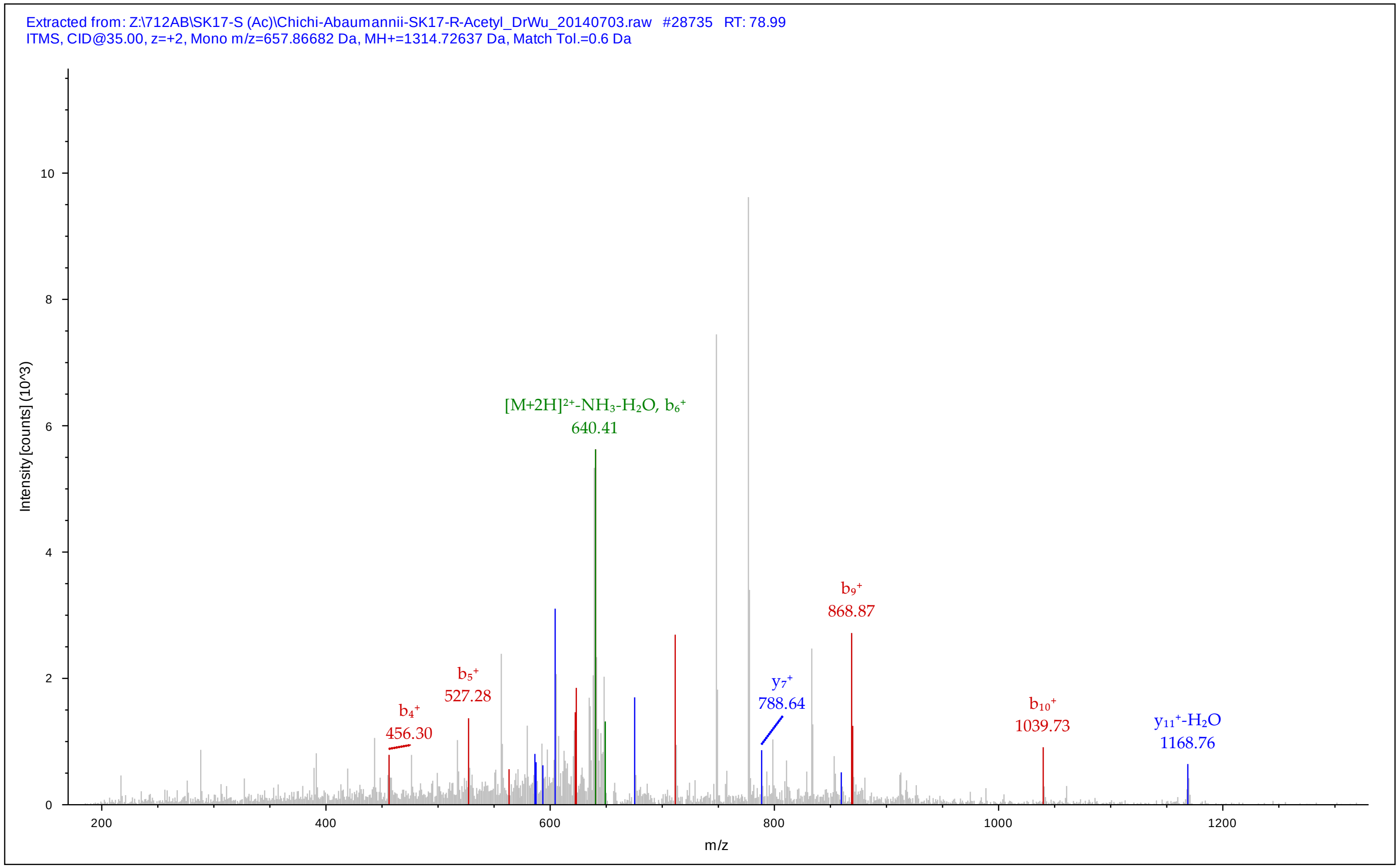

peg.2988 11806

#1	b⁺	b²⁺	b³⁺	Seq.	y⁺	y²⁺	y³⁺	#2
1	115.05021	58.02874	39.02159	N				18
2	243.14518	122.07623	81.71991	K	2106.04244	1053.52486	702.68566	17
3	344.19286	172.60007	115.40247	T	1977.94747	989.47737	659.98734	16
4	514.29839	257.65283	172.10431	K-Acetyl	1876.89979	938.95353	626.30478	15
5	627.38246	314.19487	209.79900	L	1706.79426	853.90077	569.60294	14
6	740.46653	370.73690	247.49369	I	1593.71019	797.35873	531.90825	13
7	837.51930	419.26329	279.84462	P	1480.62612	740.81670	494.21356	12
8	1000.58262	500.79495	334.19906	Y	1383.57335	692.29031	461.86263	11
9	1115.60957	558.30842	372.54137	D	1220.51003	610.75865	407.50819	10
10	1243.66815	622.33771	415.22757	Q	1105.48308	553.24518	369.16588	9
11	1390.70357	695.85542	464.23937	M-Oxidation	977.42450	489.21589	326.47968	8
12	1505.73052	753.36890	502.58169	D	830.38908	415.69818	277.46788	7
13	1604.79894	802.90311	535.60450	V	715.36213	358.18470	239.12556	6
14	1732.89391	866.95059	578.30282	K	616.29371	308.65049	206.10275	5
15	1829.94668	915.47698	610.65374	P	488.19874	244.60301	163.40443	4
16	1958.98928	979.99828	653.66794	E	391.14597	196.07662	131.05351	3
17	2087.04786	1044.02757	696.35414	Q	262.10337	131.55532	88.03931	2
18				D	134.04479	67.52603	45.35311	1

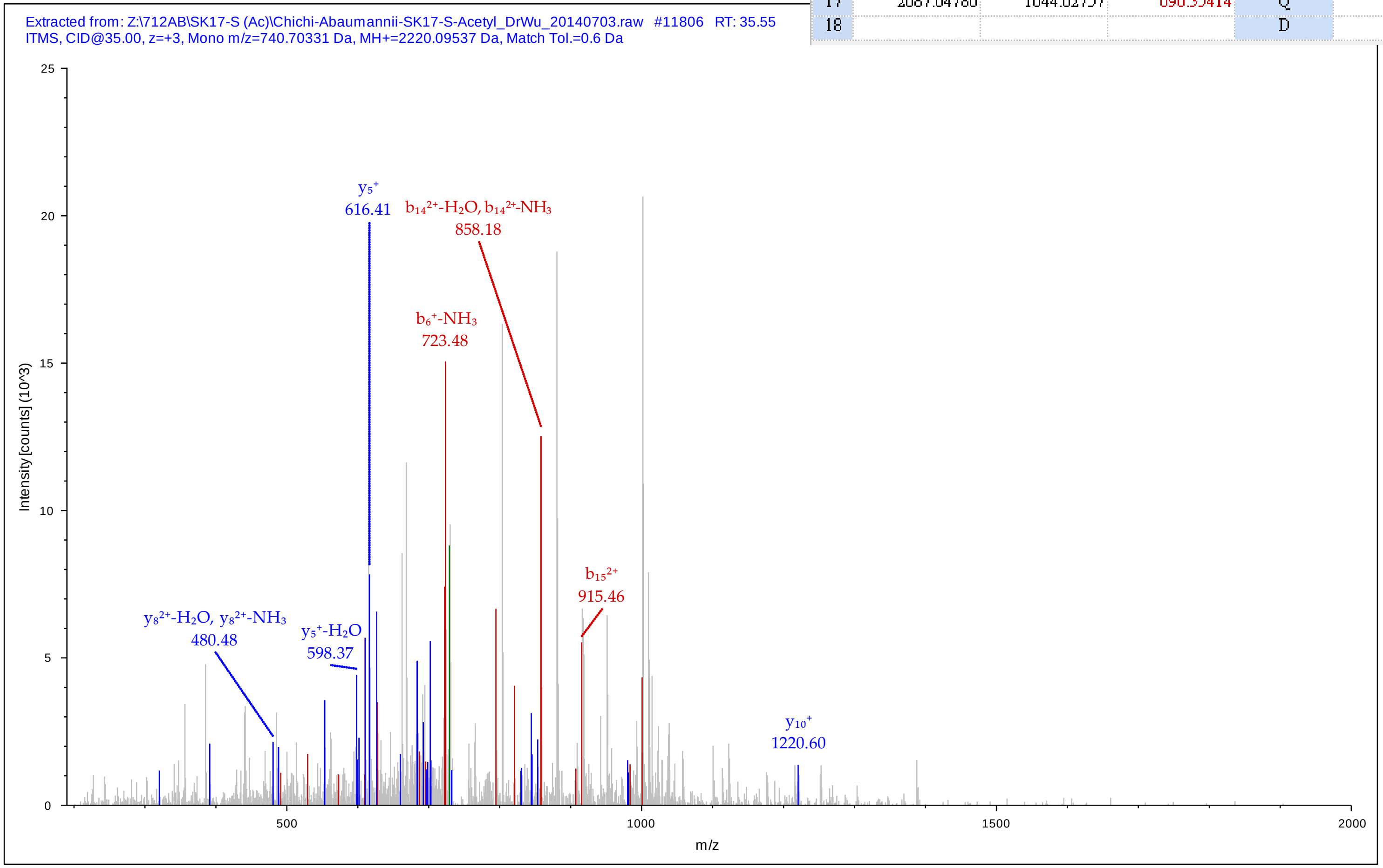

peg.2989 24304

#1	b+	b2+	b3+	Seq.	y+	y2+	y3+	#2
1	100.03931	50.52329	34.01795	G-Acetyl				35
2	171.07643	86.04185	57.69699	A	3472.90204	1736.95466	1158.30553	34
3	272.12411	136.56569	91.37955	T	3401.86492	1701.43610	1134.62649	33
4	371.19253	186.09990	124.40236	V	3300.81724	1650.91226	1100.94393	32
5	428.21400	214.61064	143.40952	G	3201.74882	1601.37805	1067.92112	31
6	515.24603	258.12665	172.42019	S	3144.72735	1572.86731	1048.91397	30
7	614.31445	307.66086	205.44300	V	3057.69532	1529.35130	1019.90329	29
8	727.39852	364.20290	243.13769	I	2958.62690	1479.81709	986.88048	28
9	824.45129	412.72928	275.48861	P	2845.54283	1423.27505	949.18579	27
10	925.49897	463.25312	309.17117	T	2748.49006	1374.74867	916.83487	26
11	1095.60451	548.30589	365.87302	K-Acetyl	2647.44238	1324.22483	883.15231	25
12	1232.66342	616.83535	411.55932	H	2477.33684	1239.17206	826.45046	24
13	1303.70054	652.35391	435.23836	A	2340.27793	1170.64260	780.76416	23
14	1416.78461	708.89594	472.93305	I	2269.24081	1135.12404	757.08512	22
15	1529.86868	765.43798	510.62774	I	2156.15674	1078.58201	719.39043	21
16	1626.92145	813.96436	542.97867	P	2043.07267	1022.03997	681.69574	20
17	1697.95857	849.48292	566.65771	A	1946.01990	973.51359	649.34482	19
18	1768.99569	885.00148	590.33675	A	1874.98278	937.99503	625.66578	18
19	1868.06411	934.53569	623.35955	V	1803.94566	902.47647	601.98674	17
20	1925.08558	963.04643	642.36671	G	1704.87724	852.94226	568.96393	16
21	2024.15400	1012.58064	675.38952	V	1647.85577	824.43152	549.95677	15
22	2139.18095	1070.09411	713.73183	D	1548.78735	774.89731	516.93397	14
23	2252.26502	1126.63615	751.42652	I	1433.76040	717.38384	478.59165	13
24	2309.28649	1155.14688	770.43368	G	1320.67633	660.84180	440.89696	12
25	2412.29568	1206.65148	804.77008	C	1263.65486	632.33107	421.88980	11
26	2469.31715	1235.16221	823.77723	G	1160.64567	580.82647	387.55341	10
27	2616.35256	1308.67992	872.78904	M-Oxidation	1103.62420	552.31574	368.54625	9
28	2730.39549	1365.70138	910.80335	N	956.58879	478.79803	319.53445	8
29	2801.43261	1401.21994	934.48239	A	842.54586	421.77657	281.52014	7
30	2914.51668	1457.76198	972.17708	I	771.50874	386.25801	257.84110	6
31	3070.61780	1535.81254	1024.21078	R	658.42467	329.71597	220.14641	5
32	3183.70187	1592.35457	1061.90547	L	502.32355	251.66541	168.11270	4
33	3270.73390	1635.87059	1090.91615	S	389.23948	195.12338	130.41801	3
34	3383.81797	1692.41262	1128.61084	L	302.20745	151.60736	101.40733	2
35				K-Acetyl	189.12338	95.06533	63.71264	1

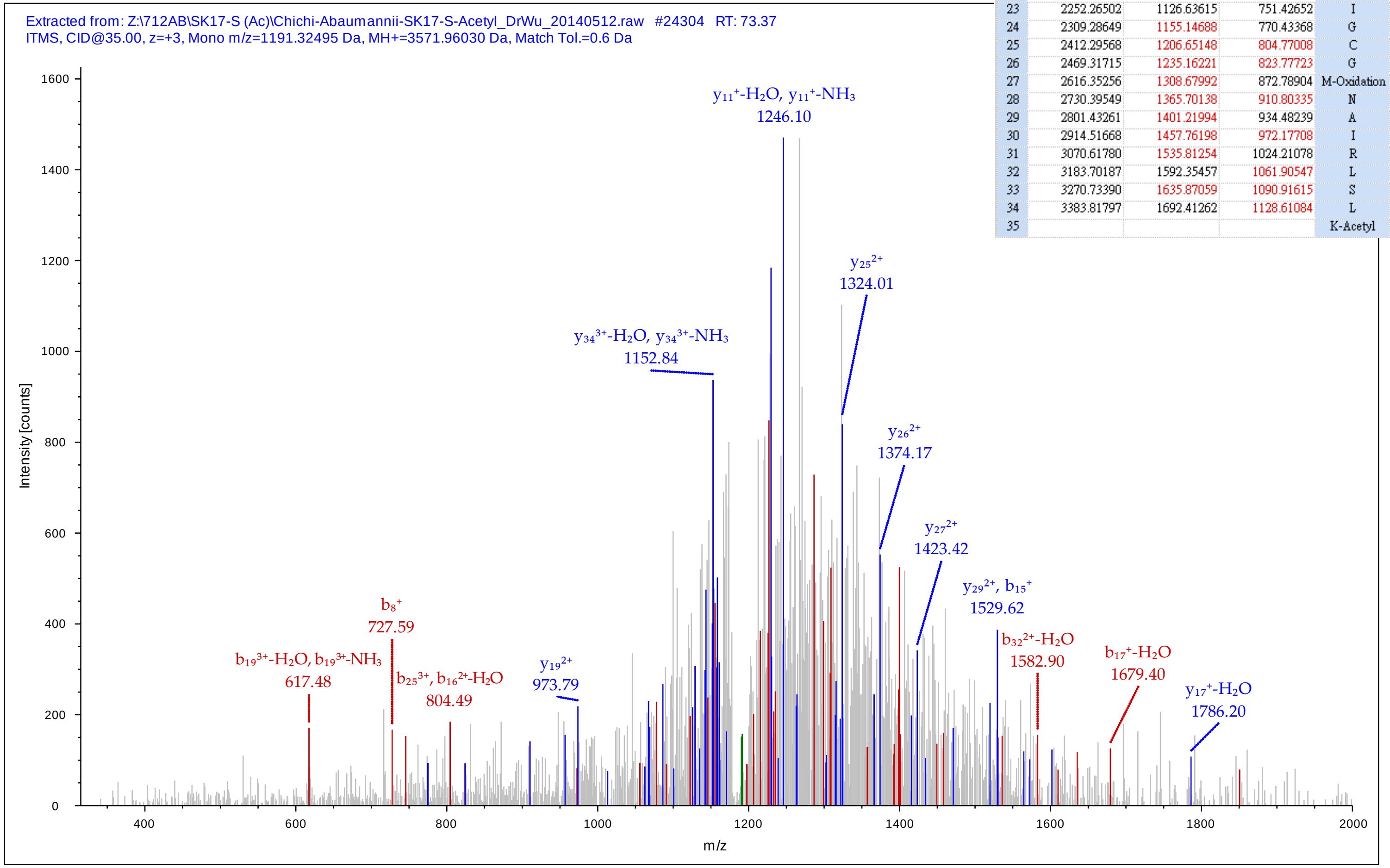

peg.2991 7318 (K4)

#1	b+	b2+	Seq.	y+	y2+	#2
1	100.07570	50.54149	V			14
2	171.11282	86.06005	A	1491.74386	746.37557	13
3	300.15542	150.58135	E	1420.70674	710.85701	12
4	470.26095	235.63411	K-Acetyl	1291.66414	646.33571	11
5	583.34502	292.17615	L	1121.55860	561.28294	10
6	670.37705	335.69216	S	1008.47453	504.74090	9
7	799.41965	400.21346	E	921.44250	461.22489	8
8	962.48297	481.74512	Y	792.39990	396.70359	7
9	1059.53574	530.27151	P	629.33658	315.17193	6
10	1173.57867	587.29297	N	532.28381	266.64554	5
11	1244.61579	622.81153	A	418.24088	209.62408	4
12	1345.66347	673.33537	T	347.20376	174.10552	3
13	1416.70059	708.85393	A	246.15608	123.58168	2
14			R	175.11896	88.06312	1

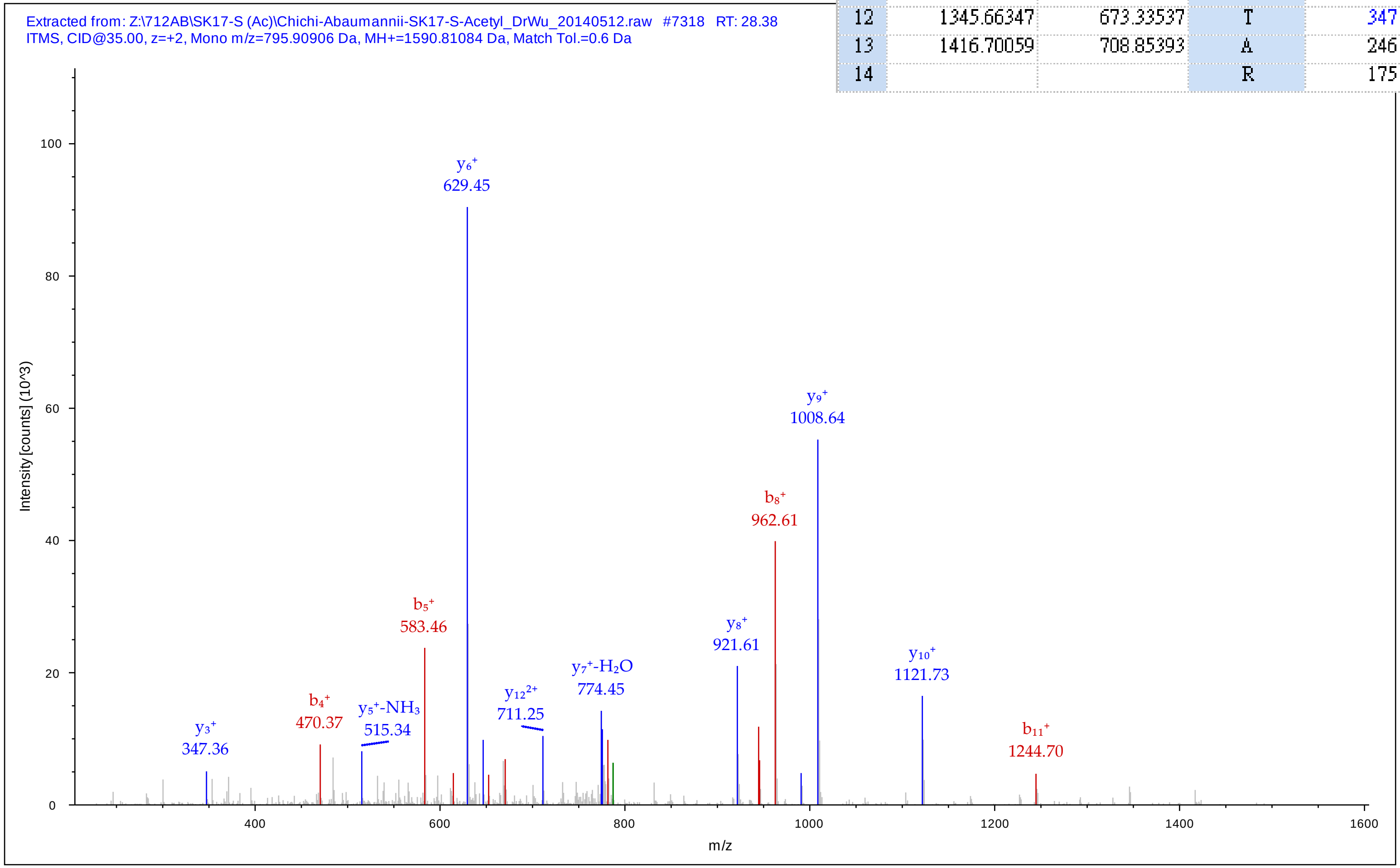

peg.2991 8522 (K4)

#1	b⁺	b²⁺	Seq.	y⁺	y²⁺	#2
1	100.07570	50.54149	V			14
2	171.11282	86.06005	A	1491.74386	746.37557	13
3	300.15542	150.58135	E	1420.70674	710.85701	12
4	470.26095	235.63411	K-Acetyl	1291.66414	646.33571	11
5	583.34502	292.17615	L	1121.55860	561.28294	10
6	670.37705	335.69216	S	1008.47453	504.74090	9
7	799.41965	400.21346	E	921.44250	461.22489	8
8	962.48297	481.74512	Y	792.39990	396.70359	7
9	1059.53574	530.27151	P	629.33658	315.17193	6
10	1173.57867	587.29297	N	532.28381	266.64554	5
11	1244.61579	622.81153	A	418.24088	209.62408	4
12	1345.66347	673.33537	T	347.20376	174.10552	3
13	1416.70059	708.85393	A	246.15608	123.58168	2
14			R	175.11896	88.06312	1

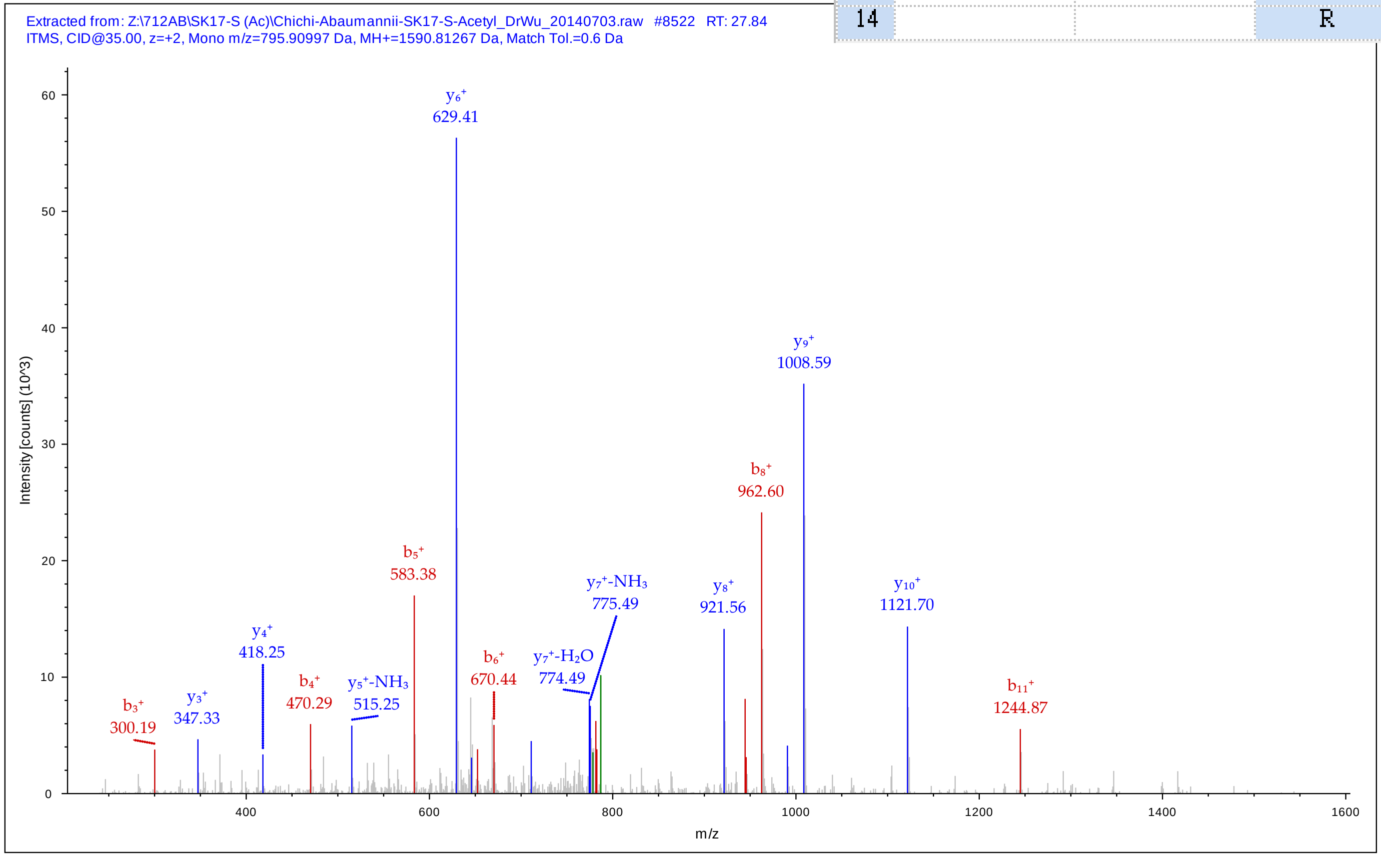

peg.2991   17916   (K16)

#1	b⁺	b²⁺	Seq.	y⁺	y²⁺	#2
1	114.09135	57.54931	L			18
2	201.12338	101.06533	S	1948.94002	974.97365	17
3	302.17106	151.58917	T	1861.90799	931.45763	16
4	430.22964	215.61846	Q	1760.86031	880.93379	15
5	487.25111	244.12919	G	1632.80173	816.90450	14
6	634.31953	317.66340	F	1575.78026	788.39377	13
7	705.35665	353.18196	A	1428.71184	714.85956	12
8	891.43597	446.22162	W	1357.67472	679.34100	11
9	1006.46292	503.73510	D	1171.59540	586.30134	10
10	1134.52150	567.76439	Q	1056.56845	528.78786	9
11	1231.57427	616.29077	P	928.50987	464.75857	8
12	1344.65834	672.83281	I	831.45710	416.23219	7
13	1415.69546	708.35137	A	718.37303	359.69015	6
14	1530.72241	765.86484	D	647.33591	324.17159	5
15	1644.76534	822.88631	N	532.30896	266.65812	4
16	1814.87087	907.93907	K-Acetyl	418.26603	209.63665	3
17	1915.91855	958.46291	T	248.16049	124.58388	2
18			K	147.11281	74.06004	1

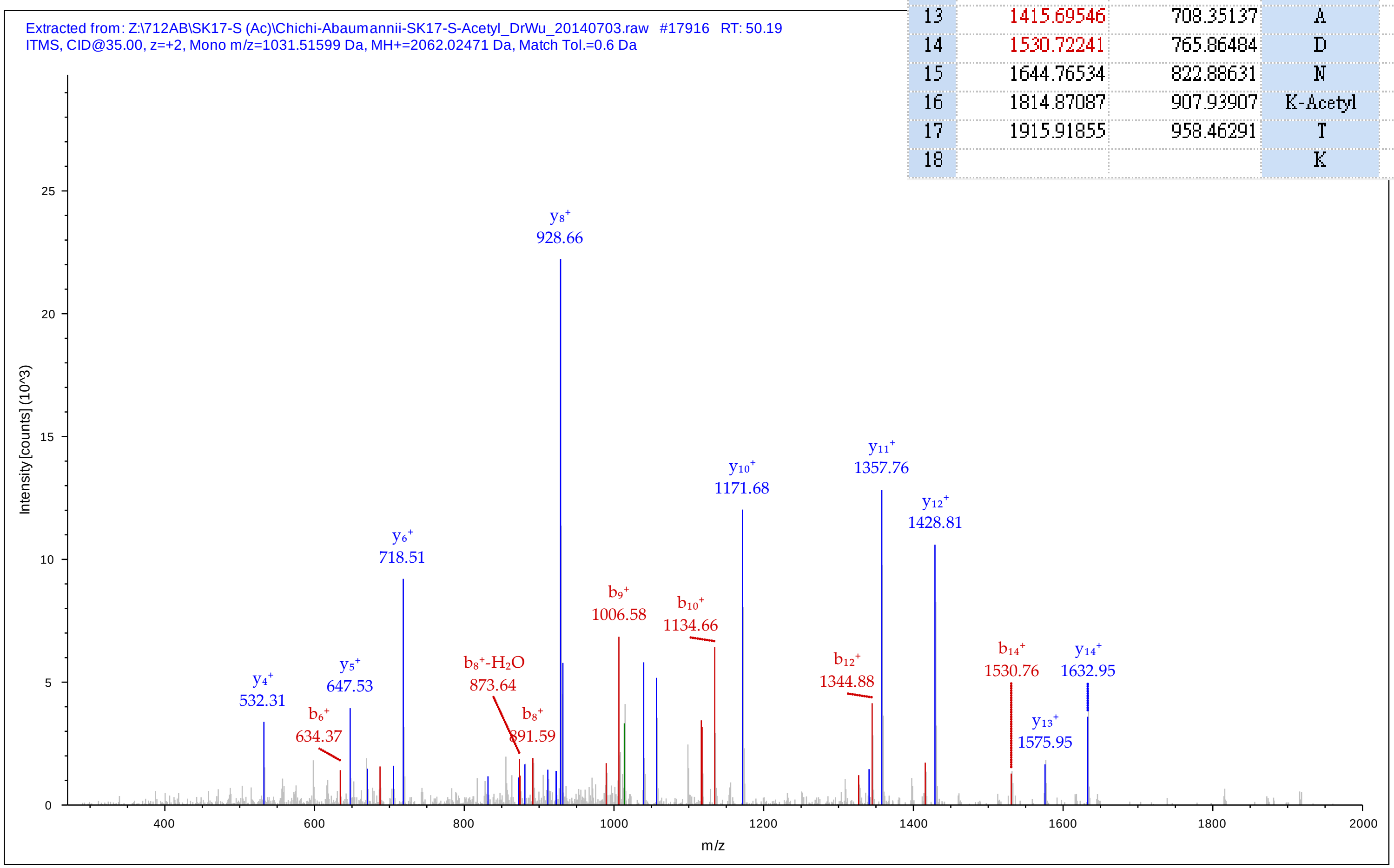

peg.2991 18587 (K16)

#1	b+	b2+	Seq.	y+	y2+	#2
1	114.09135	57.54931	L			18
2	201.12338	101.06533	S	1948.94002	974.97365	17
3	302.17106	151.58917	T	1861.90799	931.45763	16
4	430.22964	215.61846	Q	1760.86031	880.93379	15
5	487.25111	244.12919	G	1632.80173	816.90450	14
6	634.31953	317.66340	F	1575.78026	788.39377	13
7	705.35665	353.18196	A	1428.71184	714.85956	12
8	891.43597	446.22162	W	1357.67472	679.34100	11
9	1006.46292	503.73510	D	1171.59540	586.30134	10
10	1134.52150	567.76439	Q	1056.56845	528.78786	9
11	1231.57427	616.29077	P	928.50987	464.75857	8
12	1344.65834	672.83281	I	831.45710	416.23219	7
13	1415.69546	708.35137	A	718.37303	359.69015	6
14	1530.72241	765.86484	D	647.33591	324.17159	5
15	1644.76534	822.88631	N	532.30896	266.65812	4
16	1814.87087	907.93907	K-Acetyl	418.26603	209.63665	3
17	1915.91855	958.46291	T	248.16049	124.58388	2
18			K	147.11281	74.06004	1

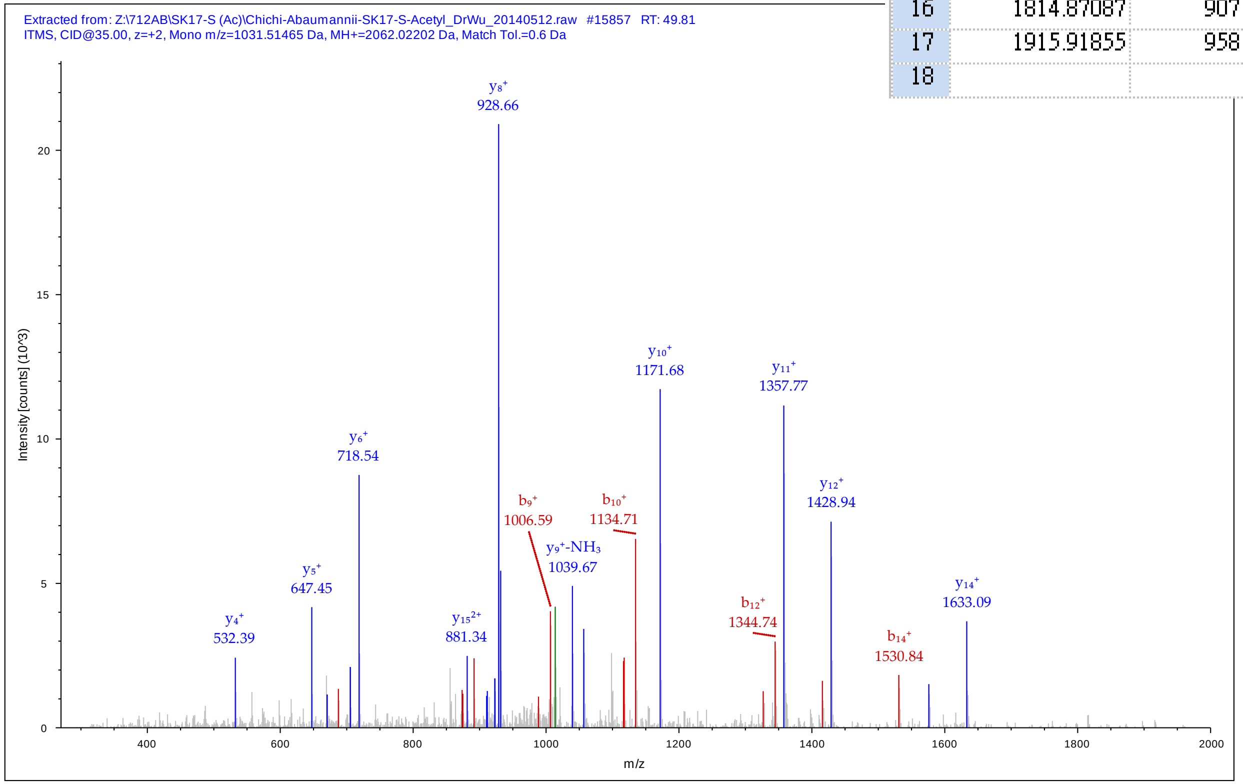

peg.2992 9463

#1	b⁺	b²⁺	Seq.	y⁺	y²⁺	#2
1	174.05834	87.53281	M-Acetyl			11
2	245.09546	123.05137	A	1156.63212	578.81970	10
3	332.12749	166.56738	S	1085.59500	543.30114	9
4	431.19591	216.10159	V	998.56297	499.78512	8
5	560.23851	280.62289	E	899.49455	450.25091	7
6	673.32258	337.16493	L	770.45195	385.72961	6
7	843.42812	422.21770	K-Acetyl	657.36788	329.18758	5
8	957.47105	479.23916	N	487.26234	244.13481	4
9	1058.51873	529.76300	T	373.21941	187.11334	3
10	1155.57150	578.28939	P	272.17173	136.58950	2
11			R	175.11896	88.06312	1

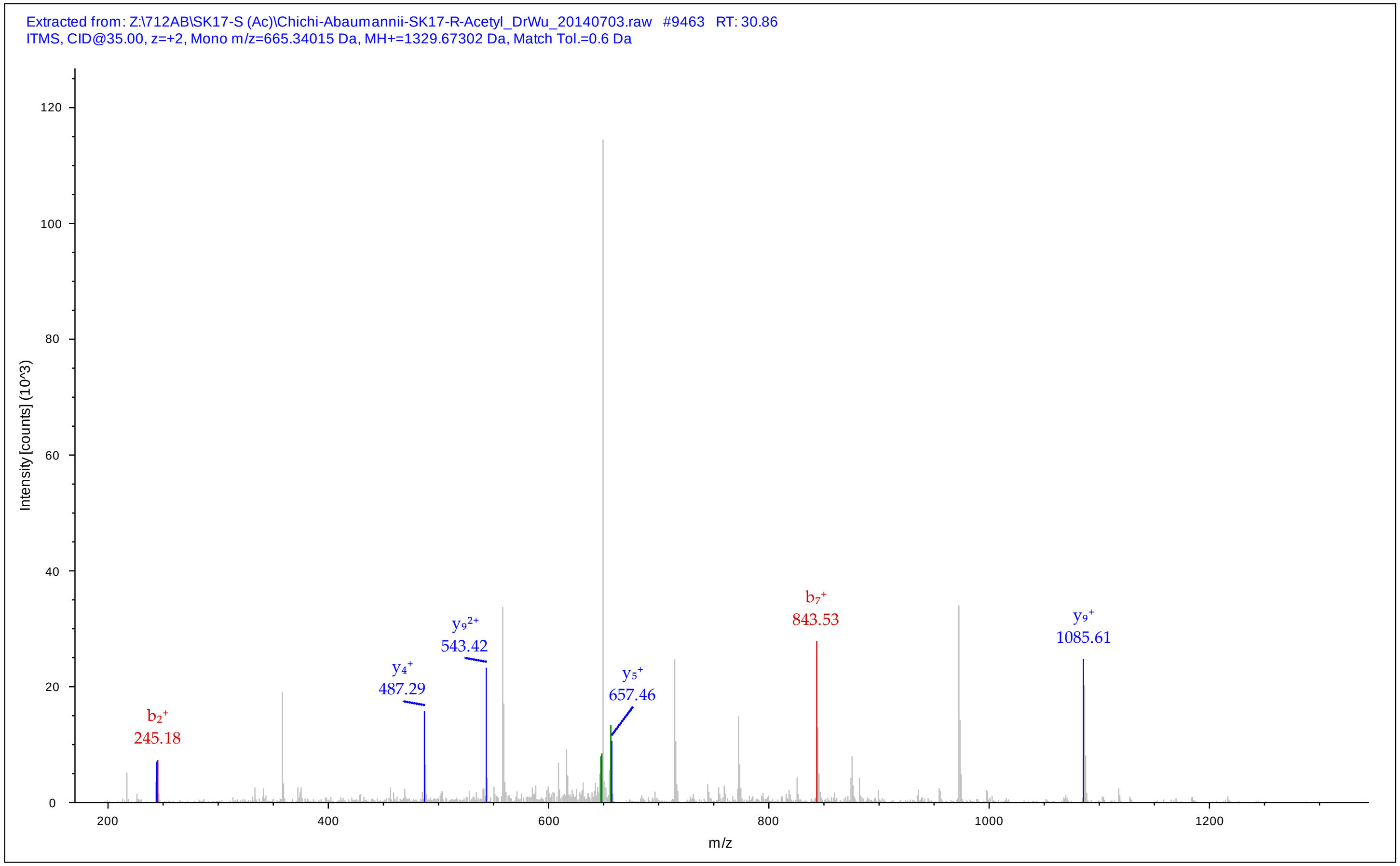

peg.300 9150

#1	b^+	b^{2+}	Seq.	y^+	y^{2+}	#2
1	115.05021	58.02874	N			11
2	230.07716	115.54222	D	1291.61001	646.30864	10
3	416.15648	208.58188	W	1176.58306	588.79517	9
4	529.24055	265.12391	L	990.50374	495.75551	8
5	689.27120	345.13924	C-Carbam···	877.41967	439.21347	7
6	776.30323	388.65525	S	717.38902	359.19815	6
7	891.33018	446.16873	D	630.35699	315.68213	5
8	1061.43572	531.22150	K-Acetyl	515.33004	258.16866	4
9	1174.51979	587.76353	I	345.22450	173.11589	3
10	1231.54126	616.27427	G	232.14043	116.57385	2
11			R	175.11896	88.06312	1

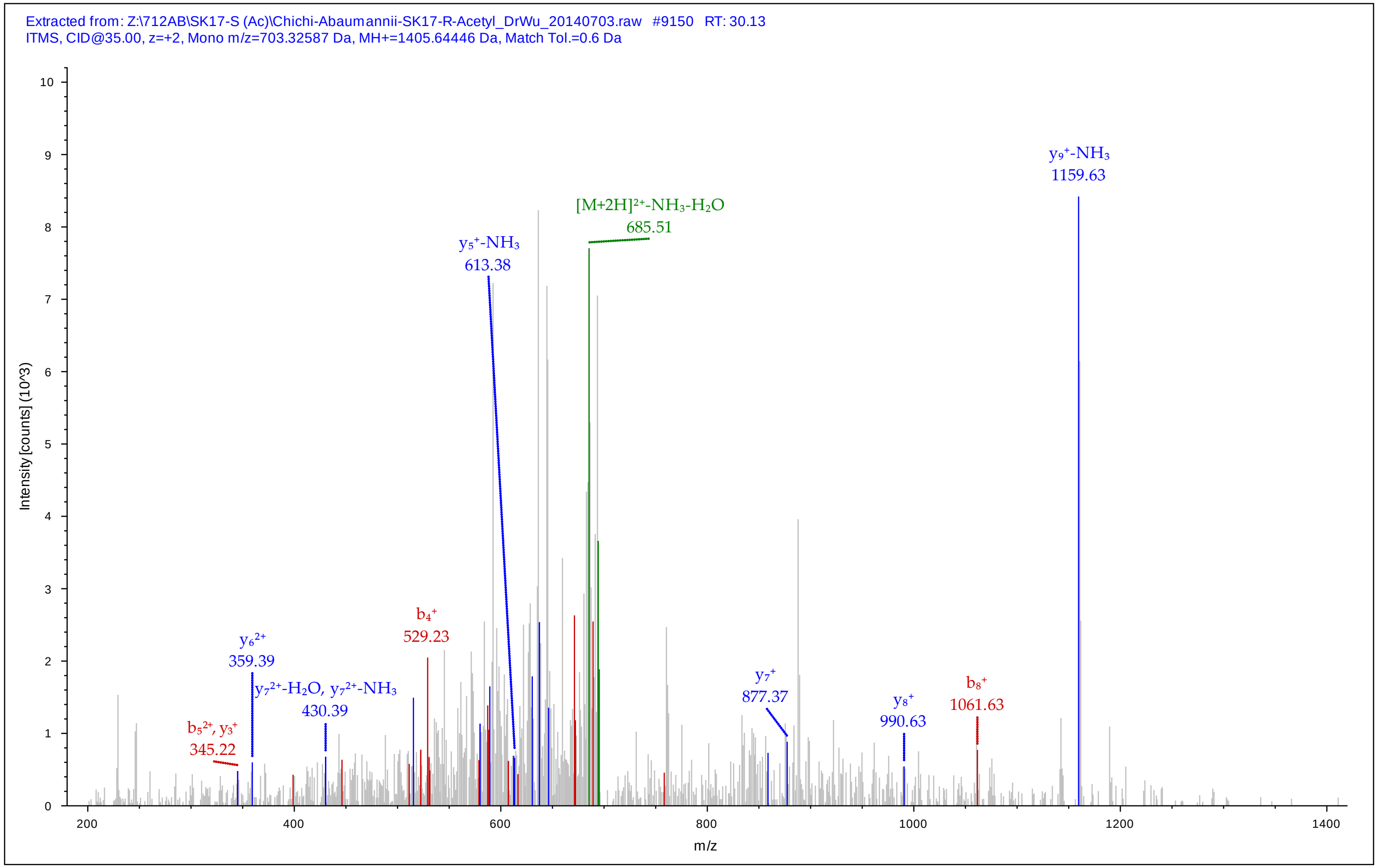

peg.300 9826

#1	b+	b2+	Seq.	y+	y2+	#2
1	115.05021	58.02874	N			11
2	230.07716	115.54222	D	1291.61001	646.30864	10
3	416.15648	208.58188	W	1176.58306	588.79517	9
4	529.24055	265.12391	L	990.50374	495.75551	8
5	689.27120	345.13924	C-Carbam…	877.41967	439.21347	7
6	776.30323	388.65525	S	717.38902	359.19815	6
7	891.33018	446.16873	D	630.35699	315.68213	5
8	1061.43572	531.22150	K-Acetyl	515.33004	258.16866	4
9	1174.51979	587.76353	I	345.22450	173.11589	3
10	1231.54126	616.27427	G	232.14043	116.57385	2
11			R	175.11896	88.06312	1

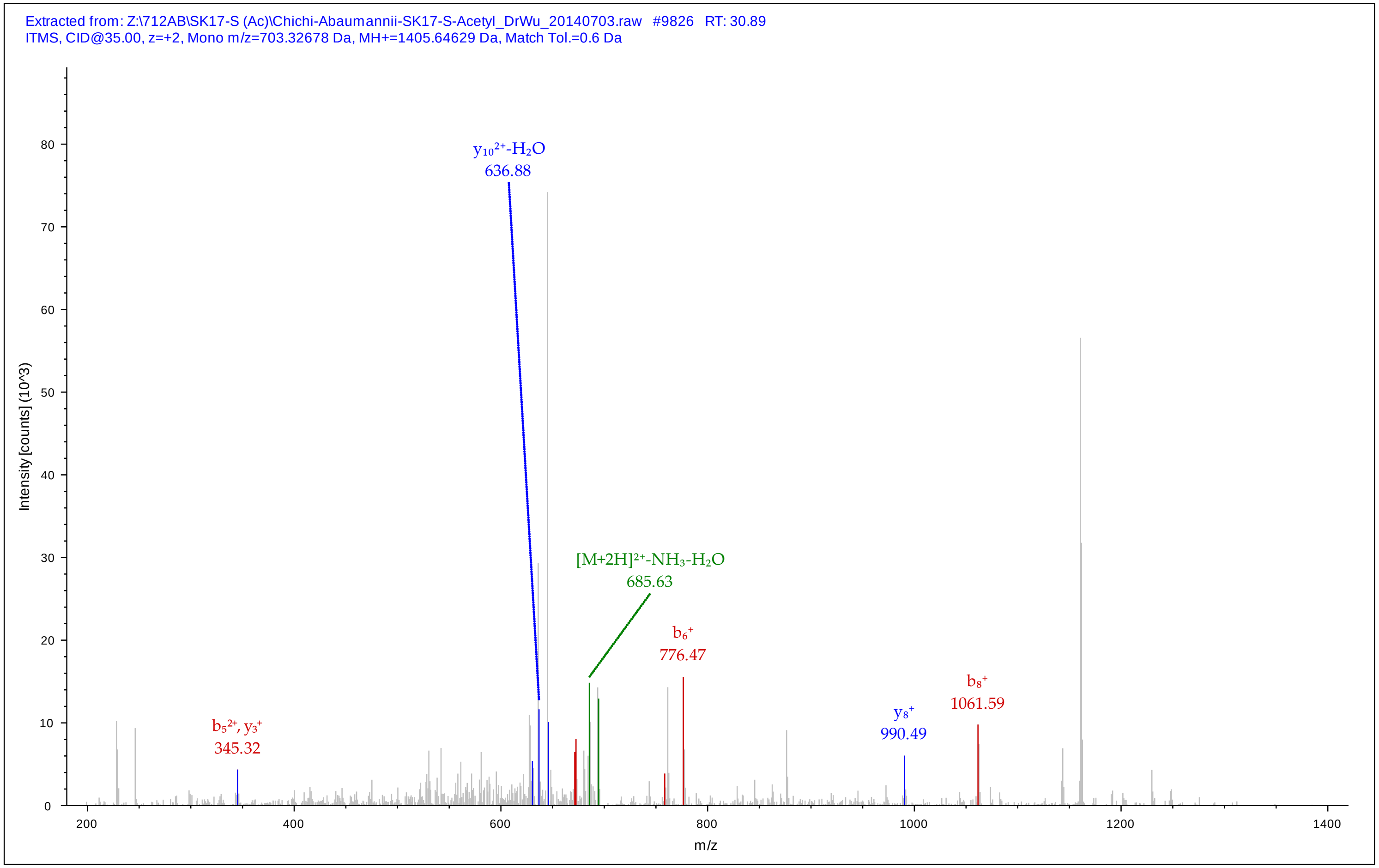

peg.3015 4727

#1	b⁺	b²⁺	Seq.	y⁺	y²⁺	#2
1	157.06077	79.03402	N-Acetyl			10
2	244.09280	122.55004	S	1074.57900	537.79314	9
3	357.17687	179.09207	I	987.54697	494.27712	8
4	444.20890	222.60809	S	874.46290	437.73509	7
5	558.25183	279.62955	N	787.43087	394.21907	6
6	728.35737	364.68232	K-Acetyl	673.38794	337.19761	5
7	815.38940	408.19834	S	503.28241	252.14484	4
8	928.47347	464.74037	I	416.25038	208.62883	3
9	1042.51640	521.76184	N	303.16631	152.08679	2
10			K-Acetyl	189.12338	95.06533	1

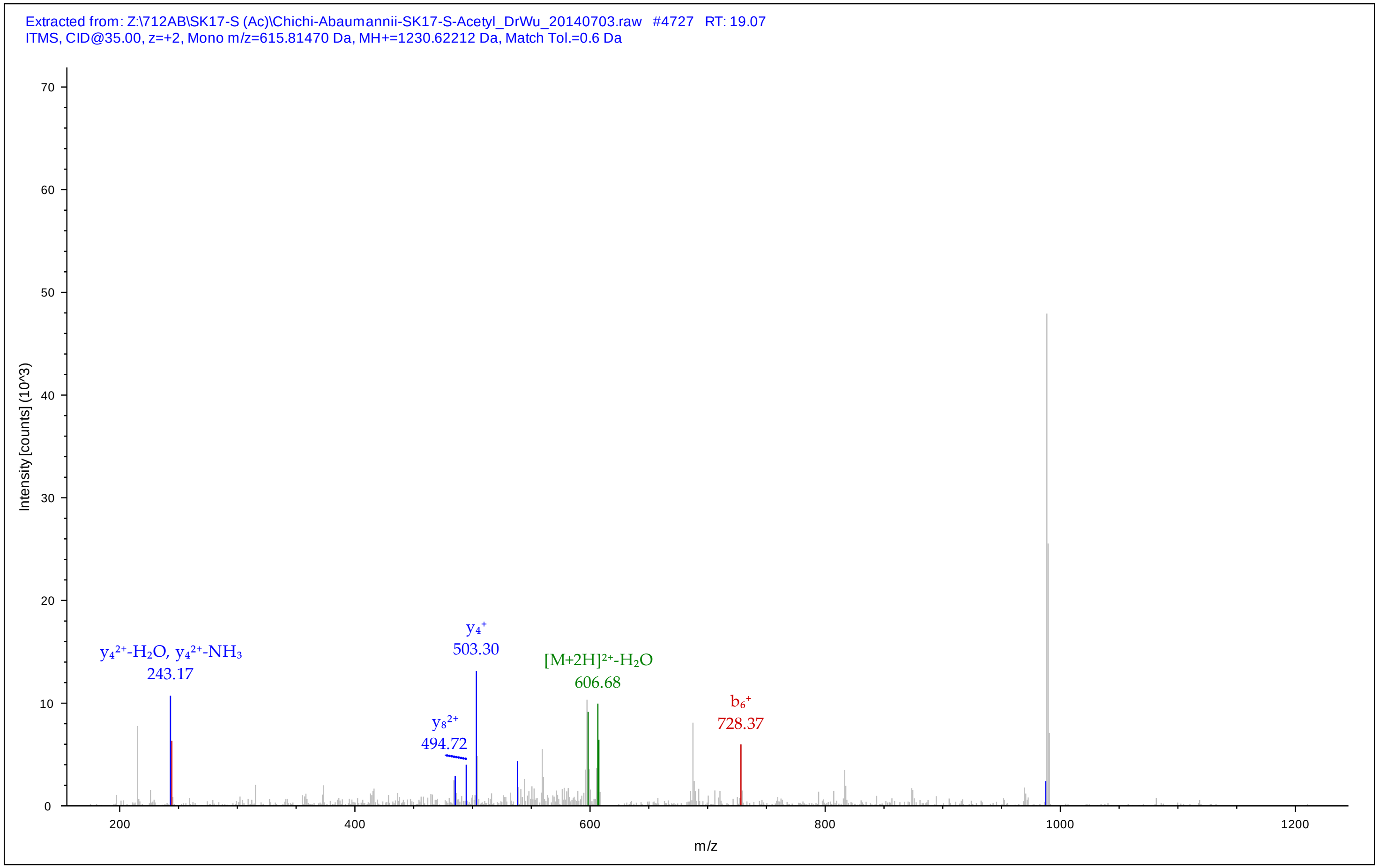

peg.3021 18838

#1	b^+	b^{2+}	b^{3+}	Seq.	y^+	y^{2+}	y^{3+}	#2
1	129.10225	65.05476	43.70560	K				18
2	226.15502	113.58115	76.05652	P	1929.02634	965.01681	643.68030	17
3	339.23909	170.12318	113.75121	I	1831.97357	916.49042	611.32937	16
4	426.27112	213.63920	142.76189	S	1718.88950	859.94839	573.63468	15
5	523.32389	262.16558	175.11281	P	1631.85747	816.43237	544.62401	14
6	652.36649	326.68688	218.12701	E	1534.80470	767.90599	512.27308	13
7	815.42981	408.21854	272.48145	Y	1405.76210	703.38469	469.25888	12
8	946.47031	473.73879	316.16162	M	1242.69878	621.85303	414.90444	11
9	1017.50743	509.25735	339.84066	A	1111.65828	556.33278	371.22428	10
10	1145.56601	573.28664	382.52685	Q	1040.62116	520.81422	347.54524	9
11	1216.60313	608.80520	406.20589	A	912.56258	456.78493	304.85904	8
12	1329.68720	665.34724	443.90058	I	841.52546	421.26637	281.18000	7
13	1428.75562	714.88145	476.92339	V	728.44139	364.72433	243.48531	6
14	1598.86115	799.93421	533.62523	K-Acetyl	629.37297	315.19012	210.46251	5
15	1655.88262	828.44495	552.63239	G	459.26743	230.13735	153.76066	4
16	1754.95104	877.97916	585.65520	V	402.24596	201.62662	134.75350	3
17	1883.00962	942.00845	628.34139	Q	303.17754	152.09241	101.73070	2
18				R	175.11896	88.06312	59.04450	1

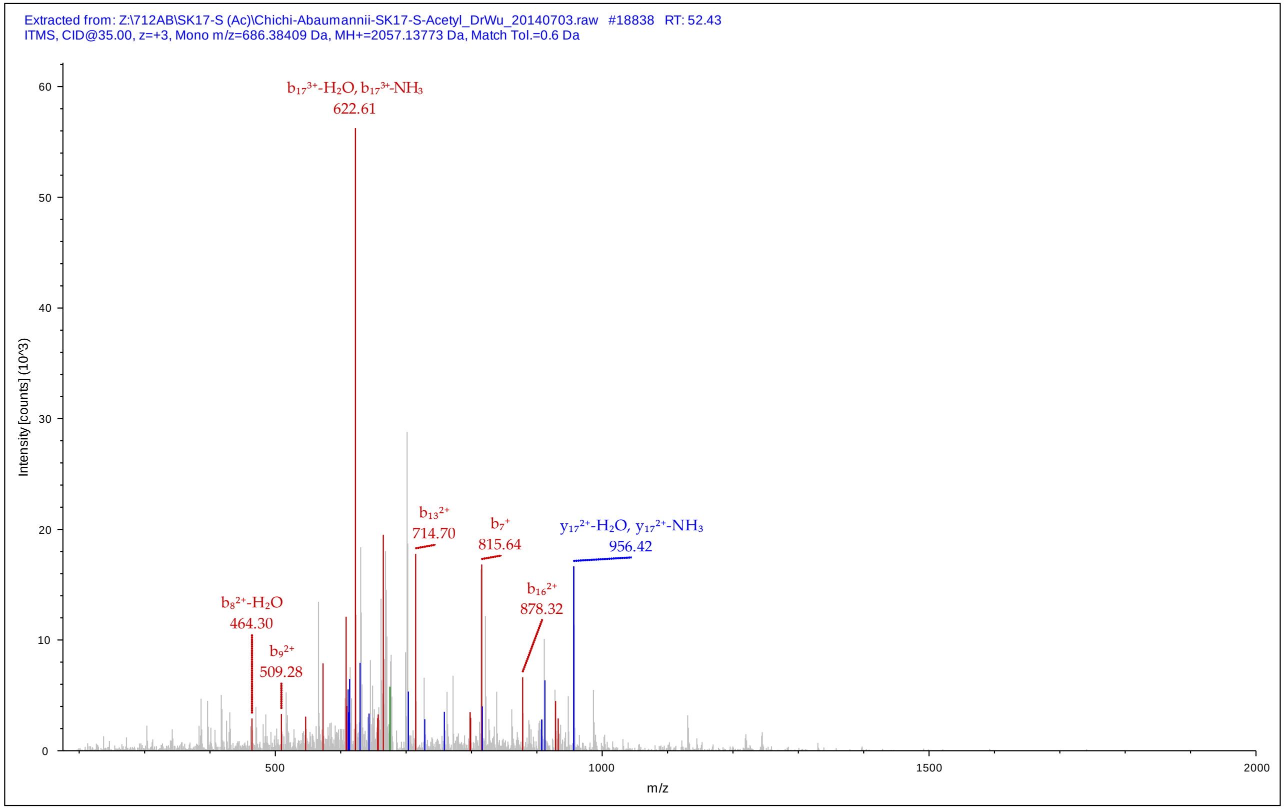

peg.3067 27946

#1	b+	b2+	Seq.	y+	y2+	#2
1	114.09135	57.54931	I			13
2	213.15977	107.08352	V	1299.75192	650.37960	12
3	328.18672	164.59700	D	1200.68350	600.84539	11
4	443.21367	222.11047	D	1085.65655	543.33191	10
5	556.29774	278.65251	L	970.62960	485.81844	9
6	726.40327	363.70527	K-Acetyl	857.54553	429.27640	8
7	825.47169	413.23948	V	687.43999	344.22363	7
8	912.50372	456.75550	S	588.37157	294.68942	6
9	1025.58779	513.29753	L	501.33954	251.17341	5
10	1082.60926	541.80827	G	388.25547	194.63137	4
11	1153.64638	577.32683	A	331.23400	166.12064	3
12	1266.73045	633.86886	L	260.19688	130.60208	2
13			K	147.11281	74.06004	1

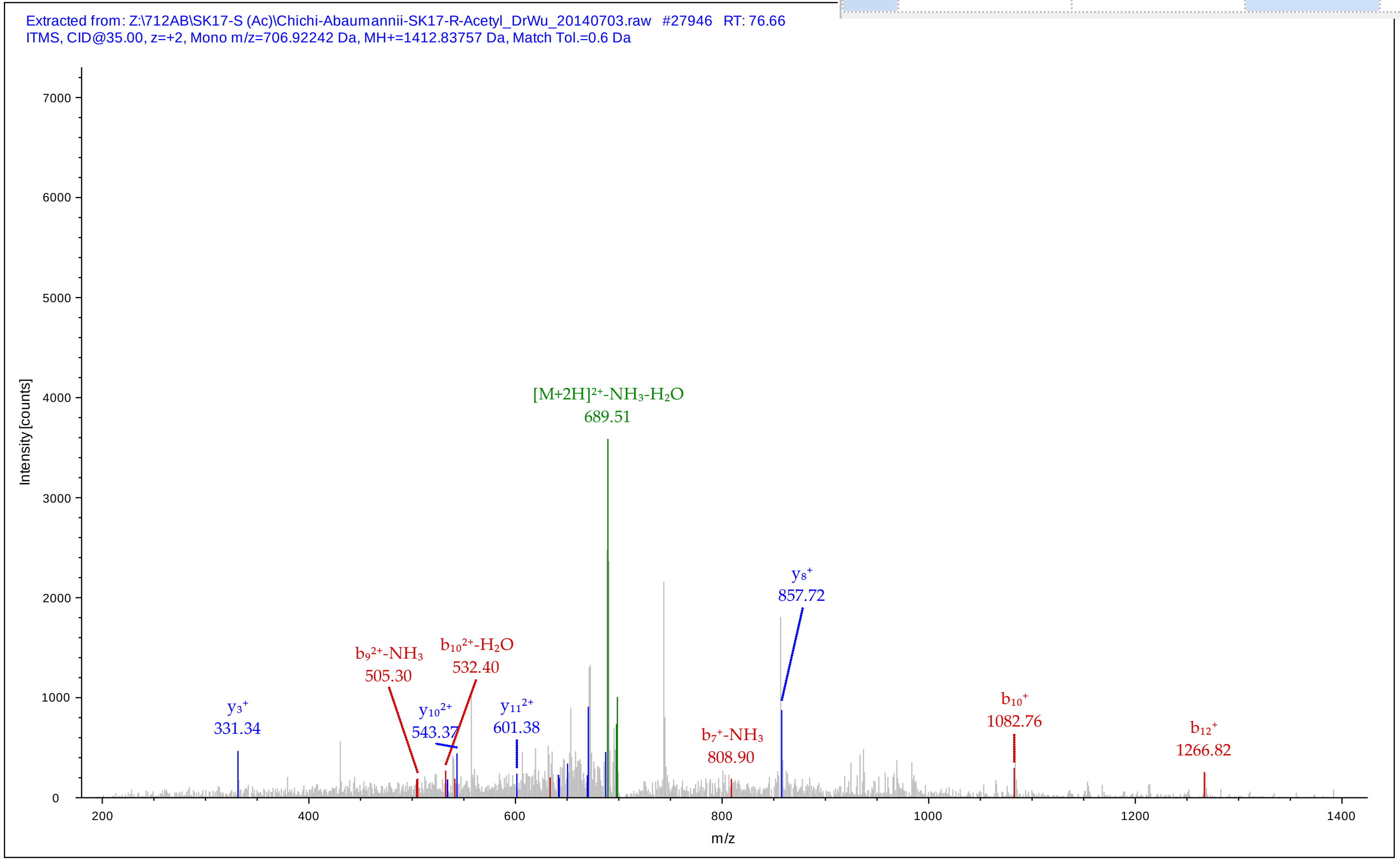

peg.3078 16051

#1	b+	b2+	Seq.	y+	y2+	#2
1	190.08626	95.54677	F-Acetyl			12
2	247.10773	124.05750	G	1345.72234	673.36481	11
3	403.20885	202.10806	R	1288.70087	644.85407	10
4	500.26162	250.63445	P	1132.59975	566.80351	9
5	670.36716	335.68722	K-Acetyl	1035.54698	518.27713	8
6	785.39411	393.20069	D	865.44144	433.22436	7
7	900.42106	450.71417	D	750.41449	375.71088	6
8	1063.48438	532.24583	Y	635.38754	318.19741	5
9	1176.56845	588.78786	L	472.32422	236.66575	4
10	1289.65252	645.32990	I	359.24015	180.12371	3
11	1360.68964	680.84846	A	246.15608	123.58168	2
12			R	175.11896	88.06312	1

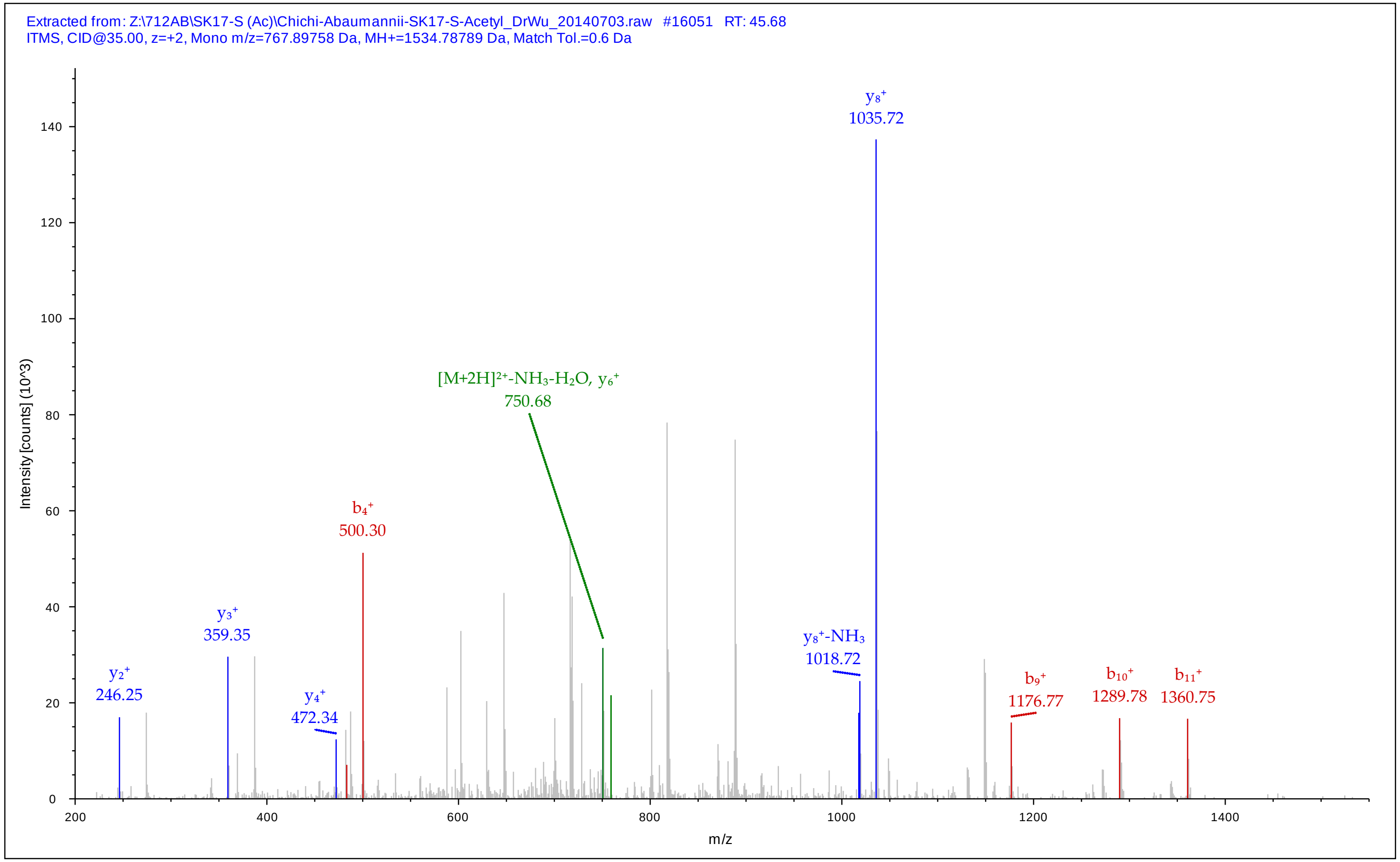

peg.312 29828

#1	b⁺	b²⁺	Seq.	y⁺	y²⁺	#2
1	114.09135	57.54931	L			23
2	227.17542	114.09135	I	2555.53648	1278.27188	22
3	374.24384	187.62556	F	2442.45241	1221.72984	21
4	487.32791	244.16759	L	2295.38399	1148.19563	20
5	558.36503	279.68615	A	2182.29992	1091.65360	19
6	659.41271	330.20999	T	2111.26280	1056.13504	18
7	772.49678	386.75203	I	2010.21512	1005.61120	17
8	829.51825	415.26276	G	1897.13105	949.06916	16
9	928.58667	464.79697	V	1840.10958	920.55843	15
10	1041.67074	521.33901	I	1741.04116	871.02422	14
11	1188.73916	594.87322	F	1627.95709	814.48218	13
12	1301.82323	651.41525	L	1480.88867	740.94797	12
13	1414.90730	707.95729	I	1367.80460	684.40594	11
14	1515.95498	758.48113	T	1254.72053	627.86390	10
15	1586.99210	793.99969	A	1153.67285	577.34006	9
16	1750.05542	875.53135	Y	1082.63573	541.82150	8
17	1837.08745	919.04736	S	919.57241	460.28984	7
18	1993.18857	997.09792	R	832.54038	416.77383	6
19	2106.27264	1053.63996	L	676.43926	338.72327	5
20	2276.37817	1138.69272	K-Acetyl	563.35519	282.18123	4
21	2375.44659	1188.22693	V	393.24965	197.12846	3
22	2522.51501	1261.76114	F	294.18123	147.59425	2
23			K	147.11281	74.06004	1

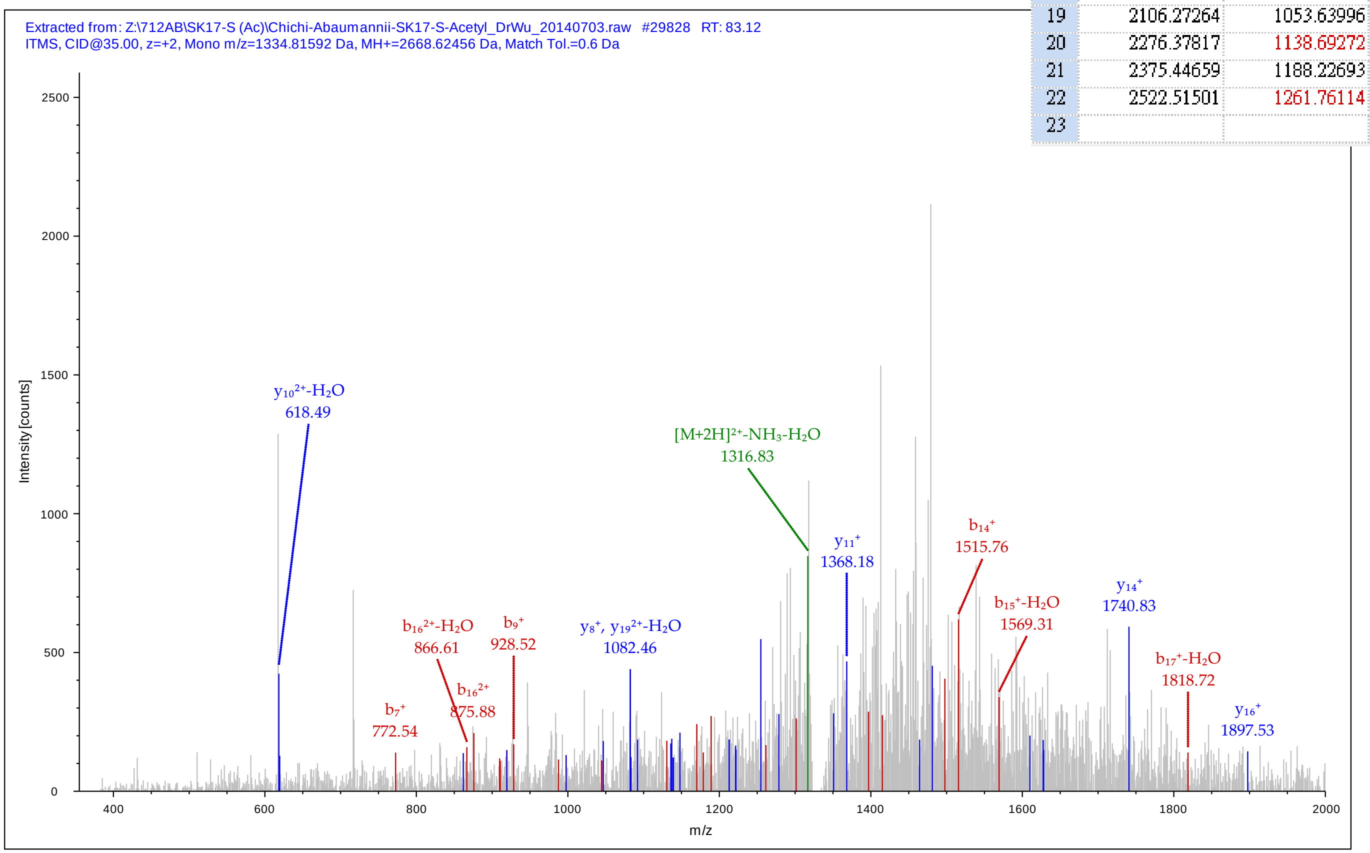

peg.369 28738

#1	b+	b2+	Seq.	y+	y2+	#2
1	158.04479	79.52603	D-Acetyl			13
2	245.07682	123.04205	S	1430.82138	715.91433	12
3	332.10885	166.55806	S	1343.78935	672.39831	11
4	445.19292	223.10010	L	1256.75732	628.88230	10
5	532.22495	266.61611	S	1143.67325	572.34026	9
6	660.31992	330.66360	K	1056.64122	528.82425	8
7	789.36252	395.18490	E	928.54625	464.77676	7
8	917.42110	459.21419	Q	799.50365	400.25546	6
9	1045.47968	523.24348	Q	671.44507	336.22617	5
10	1215.58522	608.29625	K-Acetyl	543.38649	272.19688	4
11	1328.66929	664.83828	L	373.28095	187.14411	3
12	1441.75336	721.38032	L	260.19688	130.60208	2
13			K	147.11281	74.06004	1

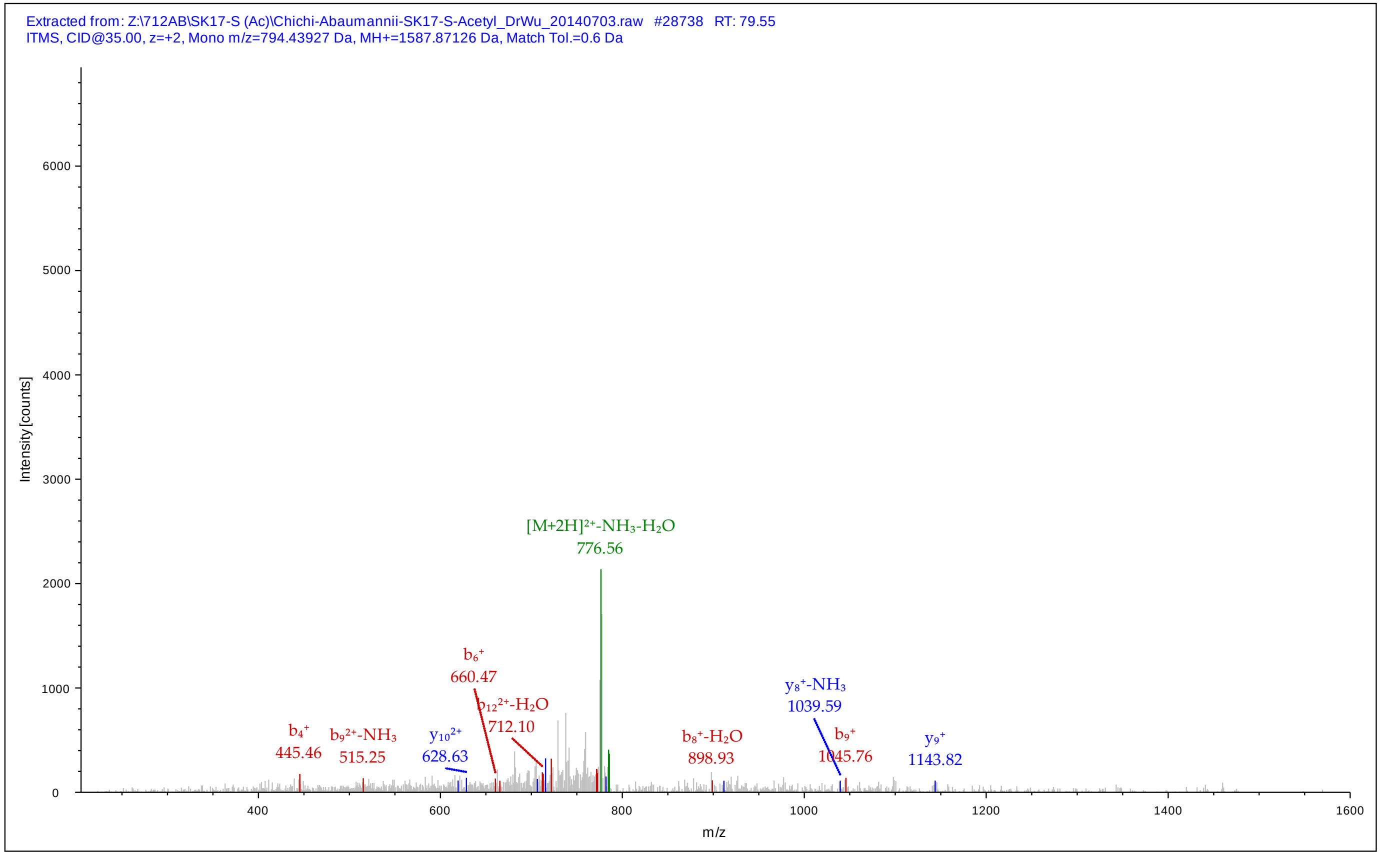

peg.376 22207

#1	b^+	b^{2+}	b^{3+}	Seq.	y^+	y^{2+}	y^{3+}	#2
1	129.06586	65.03657	43.69347	Q				18
2	258.10846	129.55787	86.70767	E	1898.92774	949.96751	633.64743	17
3	329.14558	165.07643	110.38671	A	1769.88514	885.44621	590.63323	16
4	443.18851	222.09789	148.40102	N	1698.84802	849.92765	566.95419	15
5	530.22054	265.61391	177.41170	S	1584.80509	792.90618	528.93988	14
6	633.22973	317.11850	211.74809	C	1497.77306	749.39017	499.92920	13
7	720.26176	360.63452	240.75877	S	1394.76387	697.88557	465.59281	12
8	890.36729	445.68728	297.46061	K-Acetyl	1307.73184	654.36956	436.58213	11
9	987.42006	494.21367	329.81154	P	1137.62631	569.31679	379.88029	10
10	1086.48848	543.74788	362.83434	V	1040.57354	520.79041	347.52936	9
11	1185.55690	593.28209	395.85715	V	941.50512	471.25620	314.50656	8
12	1314.59950	657.80339	438.87135	E	842.43670	421.72199	281.48375	7
13	1428.64243	714.82485	476.88566	N	713.39410	357.20069	238.46955	6
14	1598.74797	799.87762	533.58751	K-Acetyl	599.35117	300.17922	200.45524	5
15	1712.79090	856.89909	571.60182	N	429.24563	215.12645	143.75339	4
16	1783.82802	892.41765	595.28086	A	315.20270	158.10499	105.73908	3
17	1880.88079	940.94403	627.63178	P	244.16558	122.58643	82.06004	2
18				K	147.11281	74.06004	49.70912	1

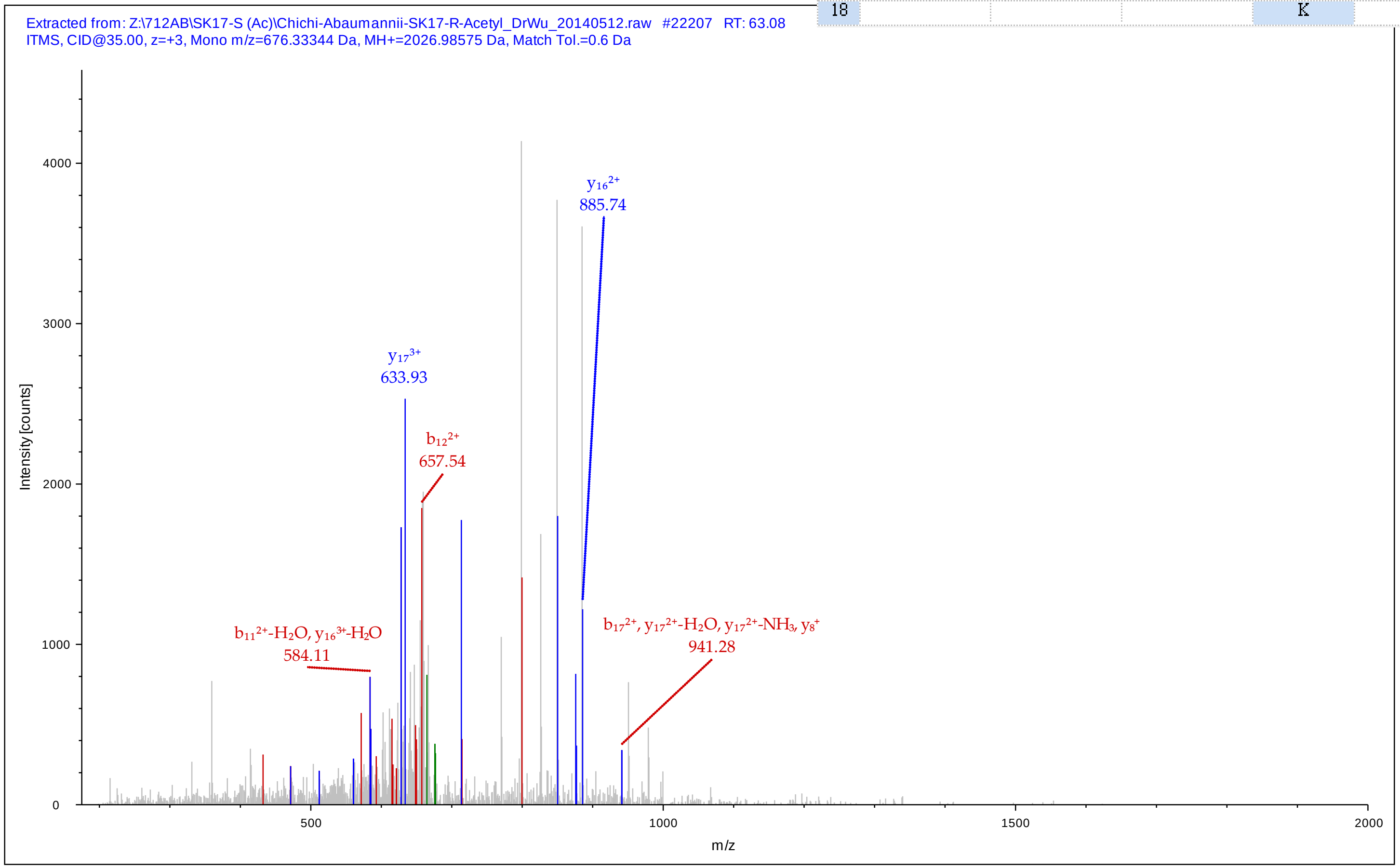

peg.383 13297

#1	b^+	b^{2+}	b^{3+}	b^{4+}	Seq.	y^+	y^{2+}	y^{3+}	y^{4+}	#2
1	130.04988	65.52858	44.02148	33.26793	E					23
2	231.09756	116.05242	77.70404	58.52985	T	2744.39038	1372.69883	915.46831	686.85305	22
3	344.18163	172.59445	115.39873	86.80086	L	2643.34270	1322.17499	881.78575	661.59113	21
4	473.22423	237.11575	158.41293	119.06151	E	2530.25863	1265.63295	844.09106	633.32011	20
5	636.28755	318.64741	212.76737	159.82734	Y	2401.21603	1201.11165	801.07686	601.05946	19
6	773.34646	387.17687	258.45367	194.09207	H	2238.15271	1119.57999	746.72242	560.29363	18
7	910.40537	455.70632	304.13997	228.35680	H	2101.09380	1051.05054	701.03612	526.02891	17
8	1025.43232	513.21980	342.48229	257.11354	D	1964.03489	982.52108	655.34981	491.76418	16
9	1195.53785	598.27256	399.18413	299.63992	K-Acetyl	1849.00794	925.00761	617.00750	463.00744	15
10	1332.59676	666.80202	444.87044	333.90465	H	1678.90240	839.95484	560.30565	420.48106	14
11	1469.65567	735.33147	490.55674	368.16938	H	1541.84349	771.42538	514.61935	386.21633	13
12	1583.69860	792.35294	528.57105	396.68011	N	1404.78458	702.89593	468.93304	351.95160	12
13	1684.74628	842.87678	562.25361	421.94203	T	1290.74165	645.87446	430.91873	323.44087	11
14	1847.80960	924.40844	616.60805	462.70786	Y	1189.69397	595.35062	397.23617	298.17895	10
15	1946.87802	973.94265	649.63086	487.47496	V	1026.63065	513.81896	342.88173	257.41312	9
16	2045.94644	1023.47686	682.65366	512.24207	V	927.56223	464.28475	309.85893	232.64602	8
17	2159.98937	1080.49832	720.66797	540.75280	N	828.49381	414.75054	276.83612	207.87891	7
18	2273.07344	1137.04036	758.36266	569.02382	L	714.45088	357.72908	238.82181	179.36818	6
19	2387.11637	1194.06182	796.37697	597.53455	N	601.36681	301.18704	201.12712	151.09716	5
20	2501.15930	1251.08329	834.39128	626.04528	N	487.32388	244.16558	163.11281	122.58643	4
21	2614.24337	1307.62532	872.08597	654.31630	L	373.28095	187.14411	125.09850	94.07570	3
22	2727.32744	1364.16736	909.78066	682.58732	I	260.19688	130.60208	87.40381	65.80468	2
23					K	147.11281	74.06004	49.70912	37.53366	1

Extracted from: Z:\712AB\SK17-S (Ac)\Chichi-Abaumannii-SK17-S-Acetyl_DrWu_20140512.raw #13297 RT: 43.20
ITMS, CID@35.00, z=+4, Mono m/z=719.11230 Da, MH+=2873.42739 Da, Match Tol.=0.6 Da

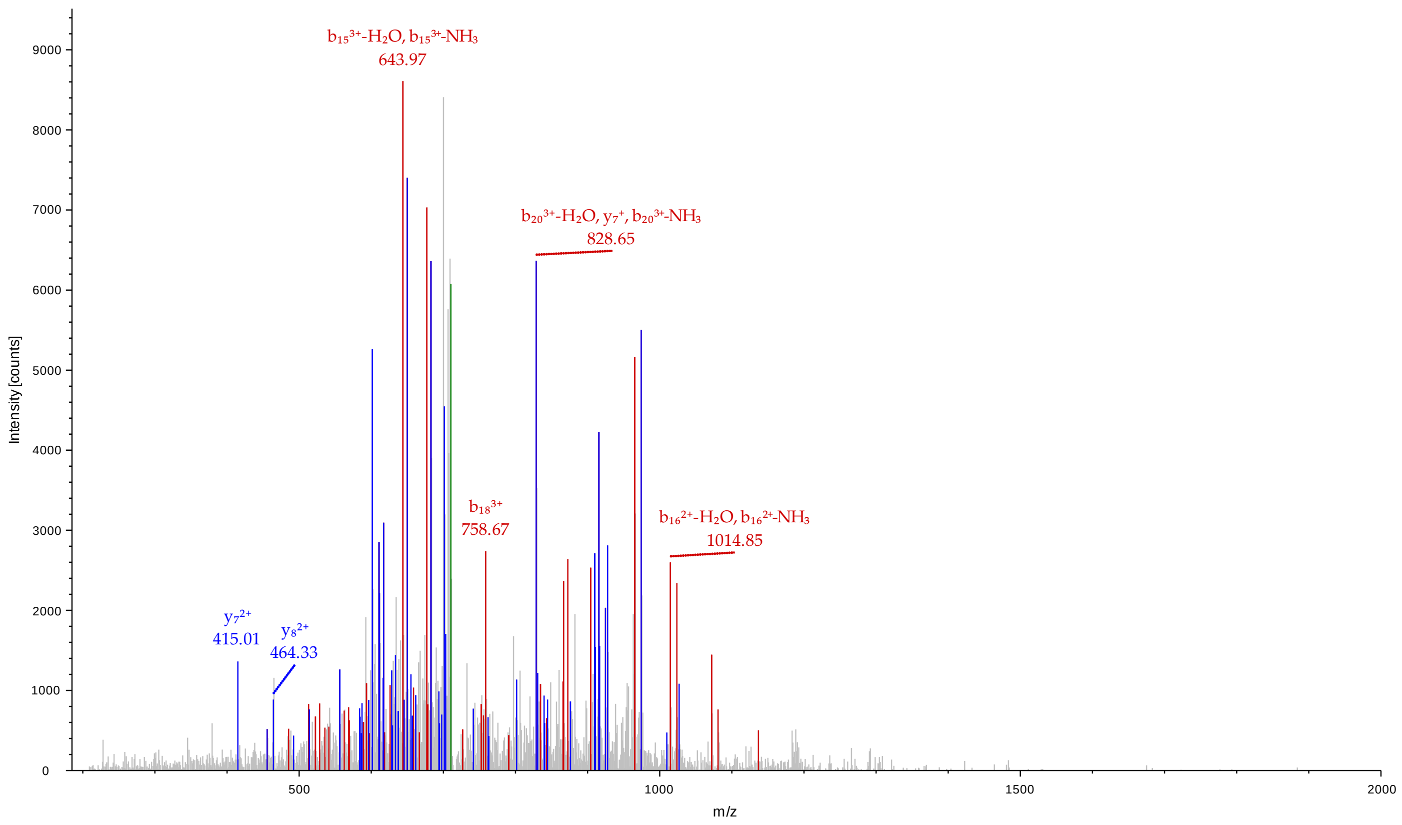

peg.412 14215

#1	b^+	b^{2+}	Seq.	y^+	y^{2+}	#2
1	130.04988	65.52858	E			13
2	277.11830	139.06279	F	1408.72202	704.86465	12
3	424.18672	212.59700	F	1261.65360	631.33044	11
4	481.20819	241.10773	G	1114.58518	557.79623	10
5	552.24531	276.62629	A	1057.56371	529.28549	9
6	651.31373	326.16050	V	986.52659	493.76693	8
7	766.34068	383.67398	D	887.45817	444.23272	7
8	936.44621	468.72674	K-Acetyl	772.43122	386.71925	6
9	993.46768	497.23748	G	602.32568	301.66648	5
10	1106.55175	553.77951	I	545.30421	273.15574	4
11	1234.61033	617.80880	Q	432.22014	216.61371	3
12	1363.65293	682.33010	E	304.16156	152.58442	2
13			R	175.11896	88.06312	1

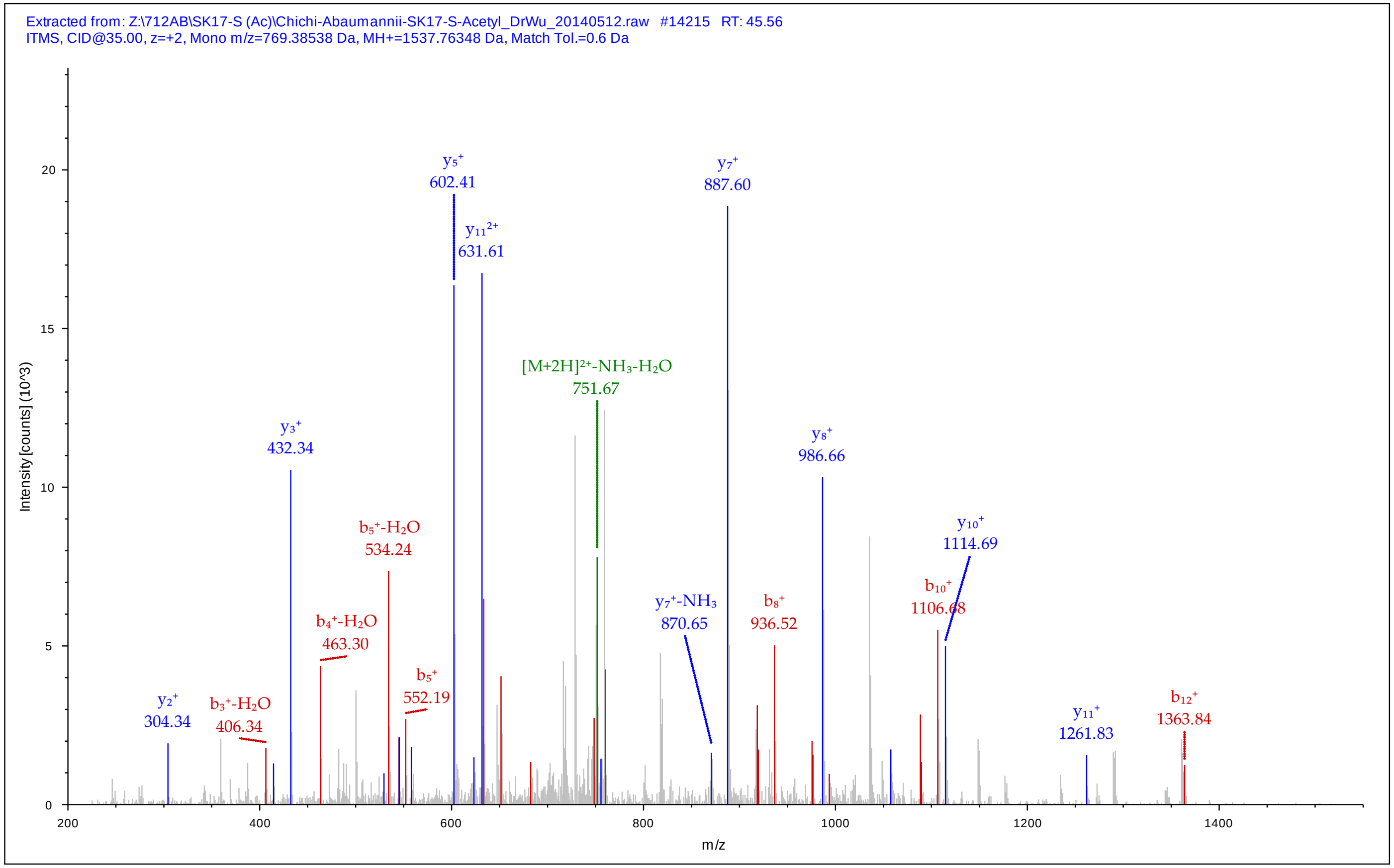

peg.451 24886

#1	b⁺	b²⁺	Seq.	y⁺	y²⁺	#2
1	100.07570	50.54149	V			15
2	199.14412	100.07570	V	1716.94325	858.97526	14
3	346.21254	173.60991	F	1617.87483	809.44105	13
4	417.24966	209.12847	A	1470.80641	735.90684	12
5	530.33373	265.67050	L	1399.76929	700.38828	11
6	645.36068	323.18398	D	1286.68522	643.84625	10
7	746.40836	373.70782	T	1171.65827	586.33277	9
8	916.51389	458.76058	K-Acetyl	1070.61059	535.80893	8
9	973.53536	487.27132	G	900.50505	450.75616	7
10	1101.59394	551.30061	Q	843.48358	422.24543	6
11	1229.65252	615.32990	Q	715.42500	358.21614	5
12	1415.73184	708.36956	W	587.36642	294.18685	4
13	1528.81591	764.91159	L	401.28710	201.14719	3
14	1641.89998	821.45363	L	288.20303	144.60515	2
15			R	175.11896	88.06312	1

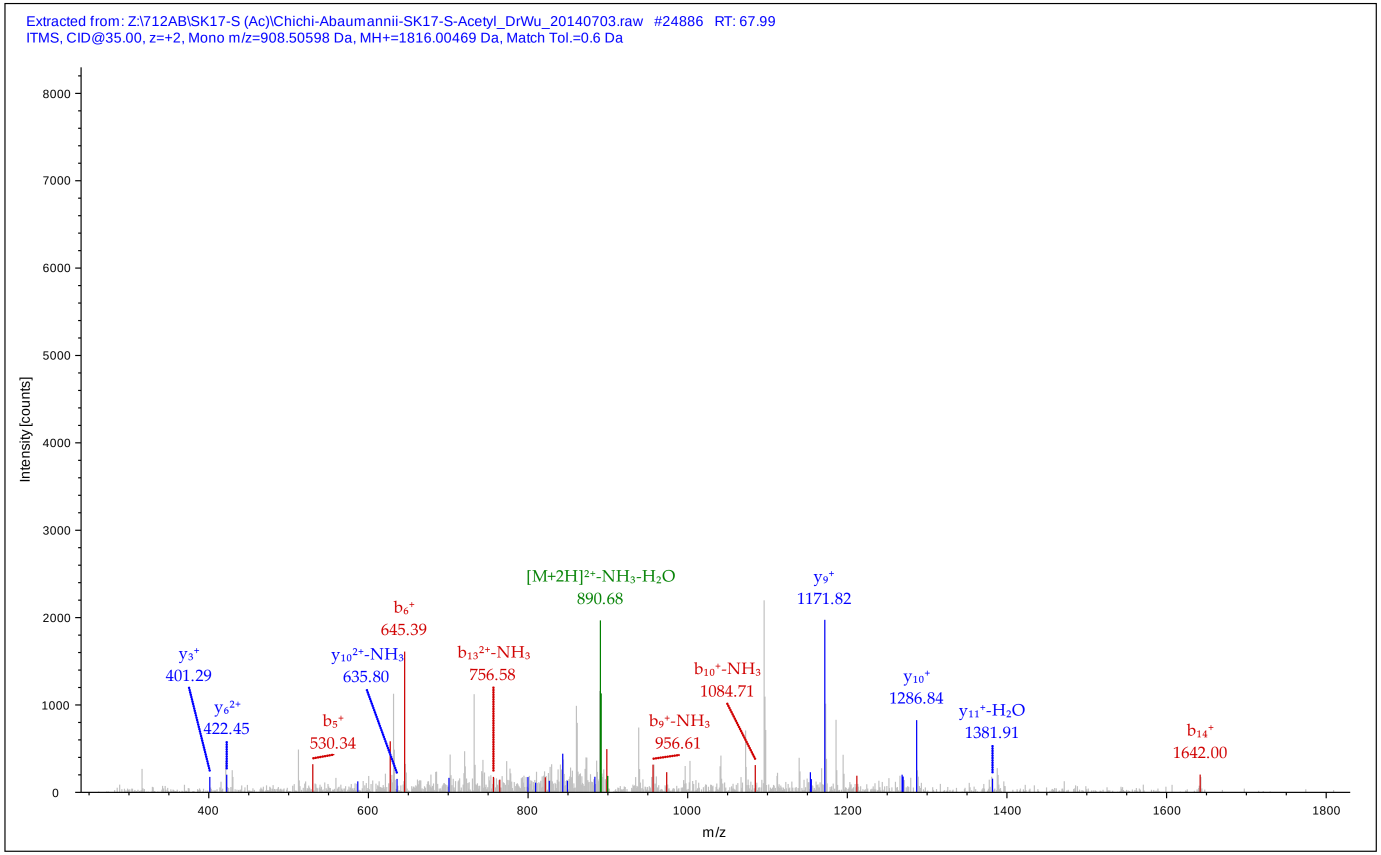

peg.452 27944

#1	b⁺	b²⁺	b³⁺	Seq.	y⁺	y²⁺	y³⁺	#2
1	102.05496	51.53112	34.68984	T				28
2	159.07643	80.04185	53.69699	G	2674.36186	1337.68457	892.12547	27
3	258.14485	129.57606	86.71980	V	2617.34039	1309.17383	873.11831	26
4	371.22892	186.11810	124.41449	L	2518.27197	1259.63962	840.09551	25
5	442.26604	221.63666	148.09353	A	2405.18790	1203.09759	802.40082	24
6	556.30897	278.65812	186.10784	N	2334.15078	1167.57903	778.72178	23
7	693.36788	347.18758	231.79414	H	2220.10785	1110.55756	740.70747	22
8	824.40838	412.70783	275.47431	M	2083.04894	1042.02811	695.02116	21
9	953.45098	477.22913	318.48851	E	1952.00844	976.50786	651.34100	20
10	1010.47245	505.73986	337.49567	G	1822.96584	911.98656	608.32680	19
11	1180.57798	590.79263	394.19751	K-Acetyl	1765.94437	883.47582	589.31964	18
12	1267.61001	634.30864	423.20819	S	1595.83883	798.42305	532.61779	17
13	1324.63148	662.81938	442.21534	G	1508.80680	754.90704	503.60712	16
14	1437.71555	719.36141	479.91003	L	1451.78533	726.39630	484.59996	15
15	1494.73702	747.87215	498.91719	G	1338.70126	669.85427	446.90527	14
16	1581.76905	791.38816	527.92787	S	1281.67979	641.34353	427.89811	13
17	1694.85312	847.93020	565.62256	L	1194.64776	597.82752	398.88744	12
18	1781.88515	891.44621	594.63323	S	1081.56369	541.28548	361.19275	11
19	1868.91718	934.96223	623.64391	S	994.53166	497.76947	332.18207	10
20	1955.94921	978.47824	652.65459	S	907.49963	454.25345	303.17139	9
21	2055.01763	1028.01245	685.67739	V	820.46760	410.73744	274.16072	8
22	2168.10170	1084.55449	723.37208	L	721.39918	361.20323	241.13791	7
23	2267.17012	1134.08870	756.39489	V	608.31511	304.66119	203.44322	6
24	2395.22870	1198.11799	799.08108	Q	509.24669	255.12698	170.42041	5
25	2532.28761	1266.64744	844.76739	H	381.18811	191.09769	127.73422	4
26	2603.32473	1302.16600	868.44643	A	244.12920	122.56824	82.04792	3
27	2700.37750	1350.69239	900.79735	P	173.09208	87.04968	58.36888	2
28				G	76.03931	38.52329	26.01795	1

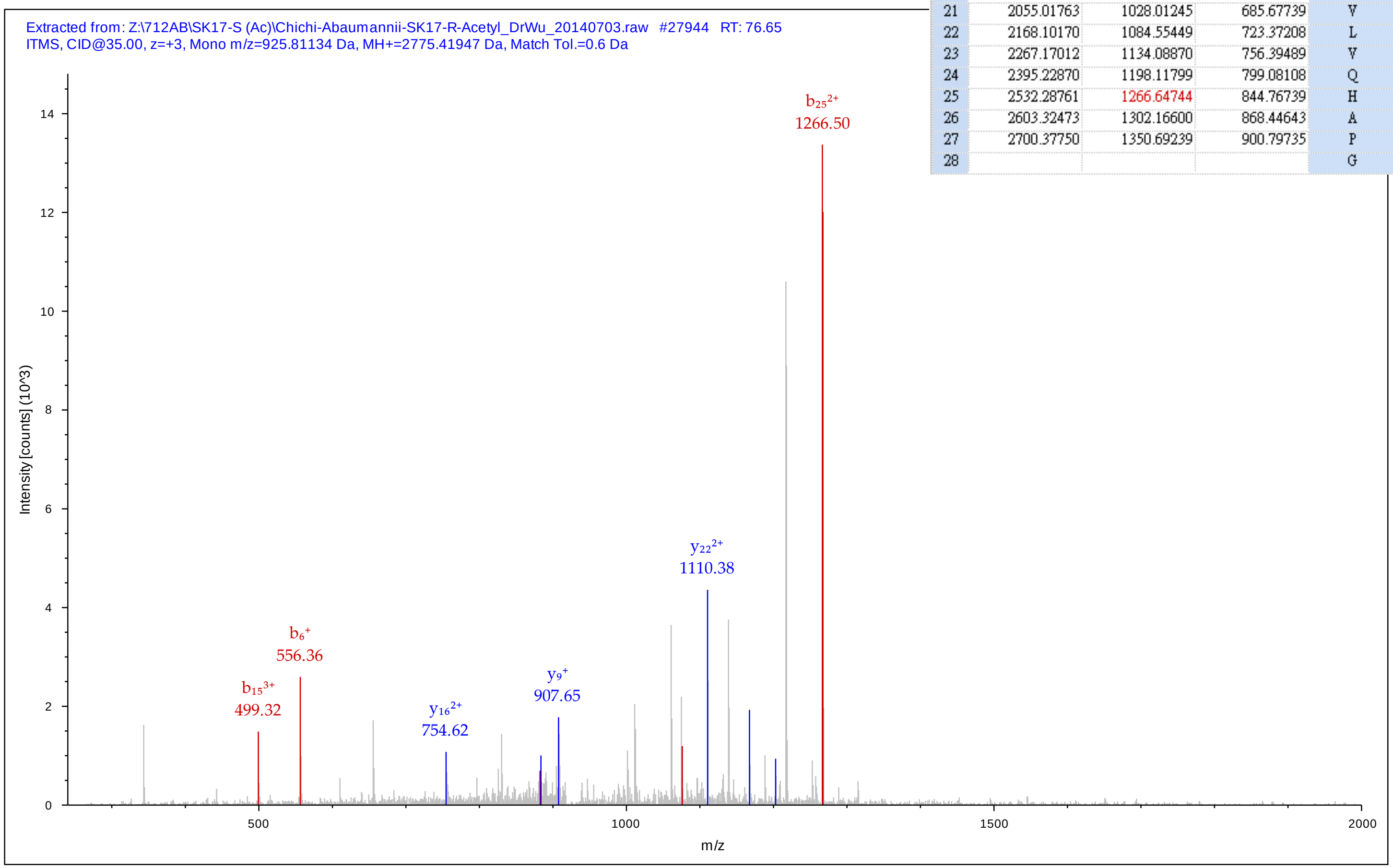

peg.519 14189

#1	b^+	b^{2+}	Seq.	y^+	y^{2+}	#2
1	114.05496	57.53112	A-Acetyl			13
2	217.06415	109.03571	C	1322.65222	661.82975	12
3	346.10675	173.55701	E	1219.64303	610.32515	11
4	417.14387	209.07557	A	1090.60043	545.80385	10
5	530.22794	265.61761	L	1019.56331	510.28529	9
6	617.25997	309.13362	S	906.47924	453.74326	8
7	731.30290	366.15509	N	819.44721	410.22724	7
8	878.37132	439.68930	F	705.40428	353.20578	6
9	935.39279	468.20003	G	558.33586	279.67157	5
10	992.41426	496.71077	G	501.31439	251.16083	4
11	1091.48268	546.24498	V	444.29292	222.65010	3
12	1261.58822	631.29775	K-Acetyl	345.22450	173.11589	2
13			R	175.11896	88.06312	1

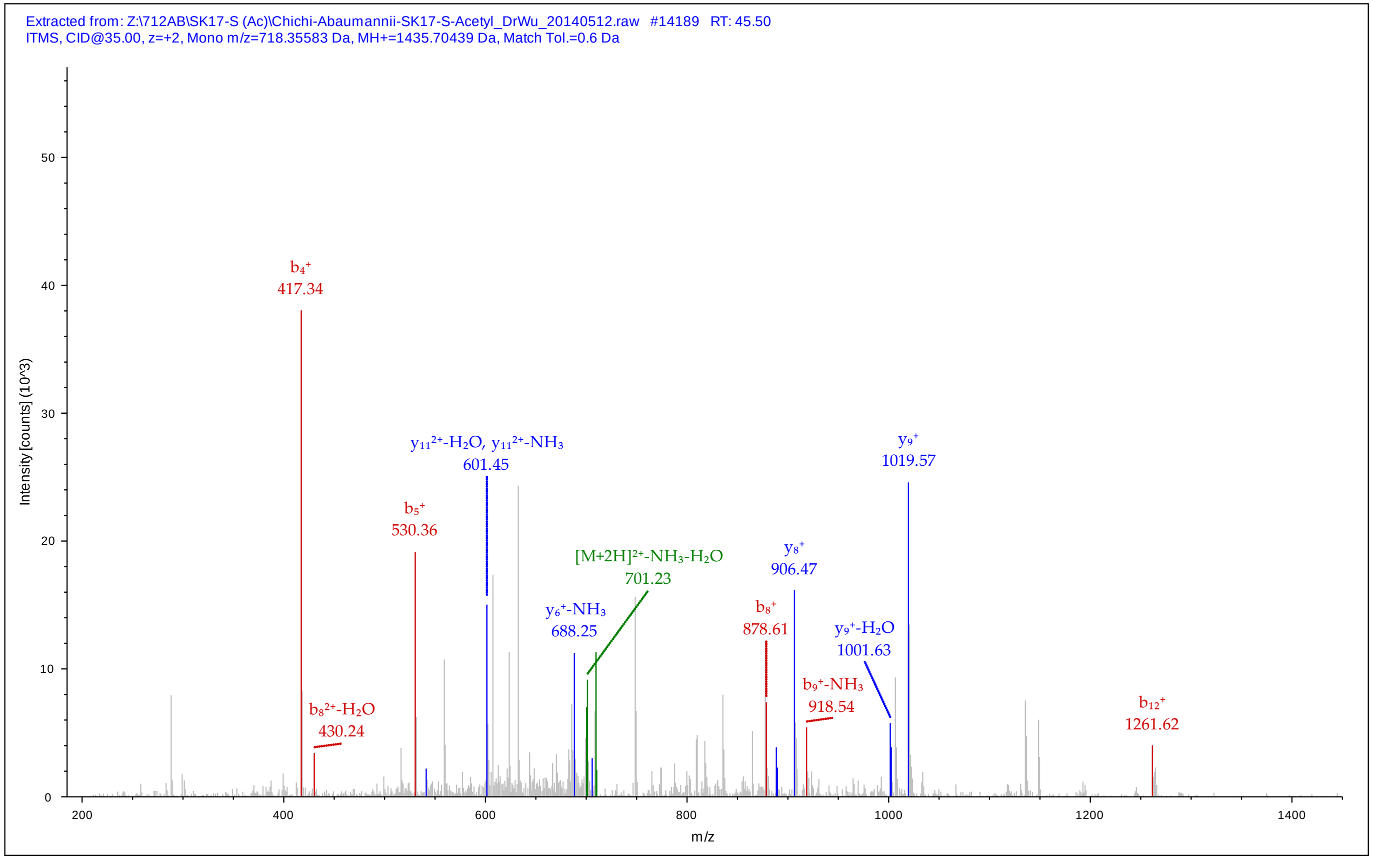

peg.522 29450

#1	b+	b2+	b3+	Seq.	y+	y2+	y3+	#2
1	148.07570	74.54149	50.03008	F				18
2	261.15977	131.08352	87.72477	L	2042.10168	1021.55448	681.37208	17
3	431.26530	216.13629	144.42662	K-Acetyl	1929.01761	965.01244	643.67739	16
4	488.28677	244.64702	163.43377	G	1758.91207	879.95967	586.97554	15
5	616.34535	308.67631	206.11997	Q	1701.89060	851.44894	567.96838	14
6	745.38795	373.19761	249.13417	E	1573.83202	787.41965	525.28219	13
7	874.43055	437.71891	292.14837	E	1444.78942	722.89835	482.26799	12
8	1003.47315	502.24021	335.16257	E	1315.74682	658.37705	439.25379	11
9	1104.52083	552.76405	368.84513	T	1186.70422	593.85575	396.23959	10
10	1218.56376	609.78552	406.85944	N	1085.65654	543.33191	362.55703	9
11	1331.64783	666.32755	444.55413	I	971.61361	486.31044	324.54272	8
12	1460.69043	730.84885	487.56833	E	858.52954	429.76841	286.84803	7
13	1575.71738	788.36233	525.91064	D	729.48694	365.24711	243.83383	6
14	1688.80145	844.90436	563.60533	I	614.45999	307.73363	205.49151	5
15	1816.89642	908.95185	606.30366	K	501.37592	251.19160	167.79682	4
16	1929.98049	965.49388	643.99835	L	373.28095	187.14411	125.09850	3
17	2043.06456	1022.03592	681.69304	L	260.19688	130.60208	87.40381	2
18				K	147.11281	74.06004	49.70912	1

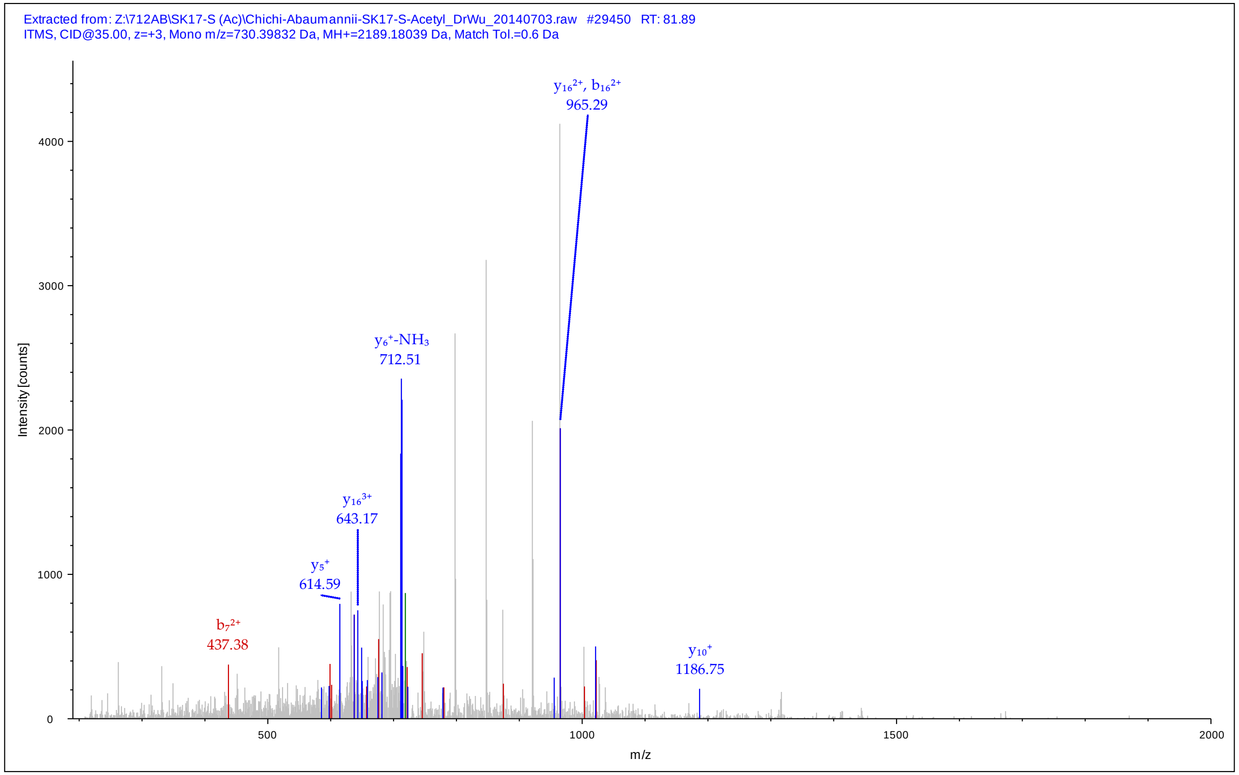

peg.553 26277

#1	b⁺	b^{2+}	b^{3+}	Seq.	y⁺	y^{2+}	y^{3+}	#2
1	88.03931	44.52329	30.01795	S				23
2	203.06626	102.03677	68.36027	D	2575.35159	1288.17943	859.12205	22
3	316.15033	158.57880	106.05496	L	2460.32464	1230.66596	820.77973	21
4	445.19293	223.10010	149.06916	E	2347.24057	1174.12392	783.08504	20
5	558.27700	279.64214	186.76385	I	2218.19797	1109.60262	740.07084	19
6	657.34542	329.17635	219.78666	V	2105.11390	1053.06059	702.37615	18
7	785.40400	393.20564	262.47285	Q	2006.04548	1003.52638	669.35334	17
8	842.42547	421.71637	281.48001	G	1877.98690	939.49709	626.66715	16
9	971.46807	486.23767	324.49421	E	1820.96543	910.98635	607.65999	15
10	1085.51100	543.25914	362.50852	N	1691.92283	846.46505	564.64579	14
11	1198.59507	599.80117	400.20321	L	1577.87990	789.44359	526.63148	13
12	1311.67914	656.34321	437.89790	L	1464.79583	732.90155	488.93679	12
13	1439.73772	720.37250	480.58409	Q	1351.71176	676.35952	451.24210	11
14	1510.77484	755.89106	504.26313	A	1223.65318	612.33023	408.55591	10
15	1673.83816	837.42272	558.61757	Y	1152.61606	576.81167	384.87687	9
16	1820.90658	910.95693	607.64038	F	989.55274	495.28001	330.52243	8
17	1891.94370	946.47549	631.31942	A	842.48432	421.74580	281.49962	7
18	1978.97573	989.99150	660.33009	S	771.44720	386.22724	257.82058	6
19	2076.02850	1038.51789	692.68102	P	684.41517	342.71122	228.80991	5
20	2190.07143	1095.53935	730.69533	N	587.36240	294.18484	196.45898	4
21	2360.17696	1180.59212	787.39717	K-Acetyl	473.31947	237.16337	158.44467	3
22	2488.27193	1244.63960	830.09549	K	303.21393	152.11060	101.74283	2
23				R	175.11896	88.06312	59.04450	1

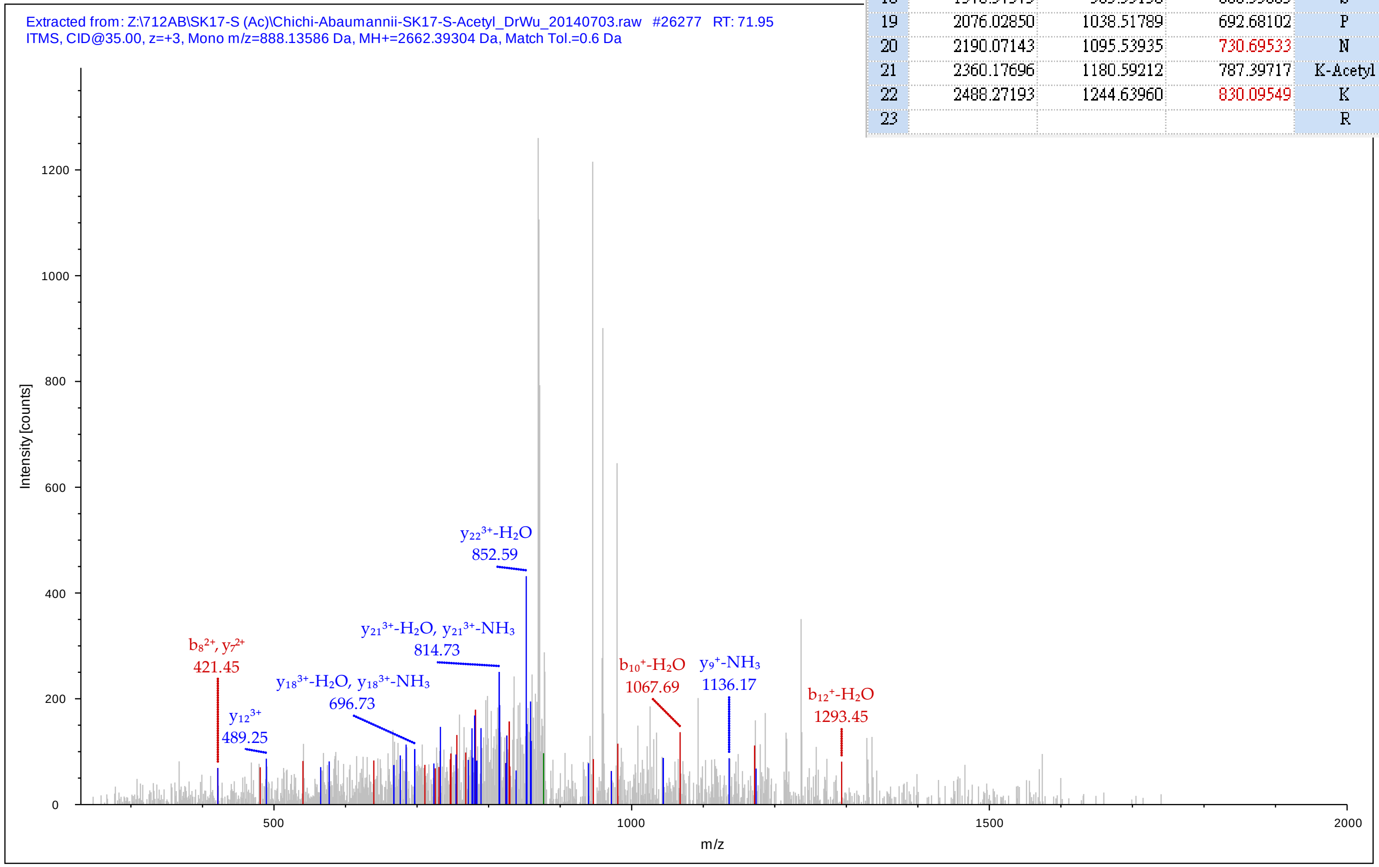

peg.555 4982

#1	b⁺	b²⁺	Seq.	y⁺	y²⁺	#2
1	100.07570	50.54149	V			6
2	229.11830	115.06279	E	630.38214	315.69471	5
3	399.22383	200.11555	K-Acetyl	501.33954	251.17341	4
4	470.26095	235.63411	A	331.23400	166.12064	3
5	583.34502	292.17615	L	260.19688	130.60208	2
6			K	147.11281	74.06004	1

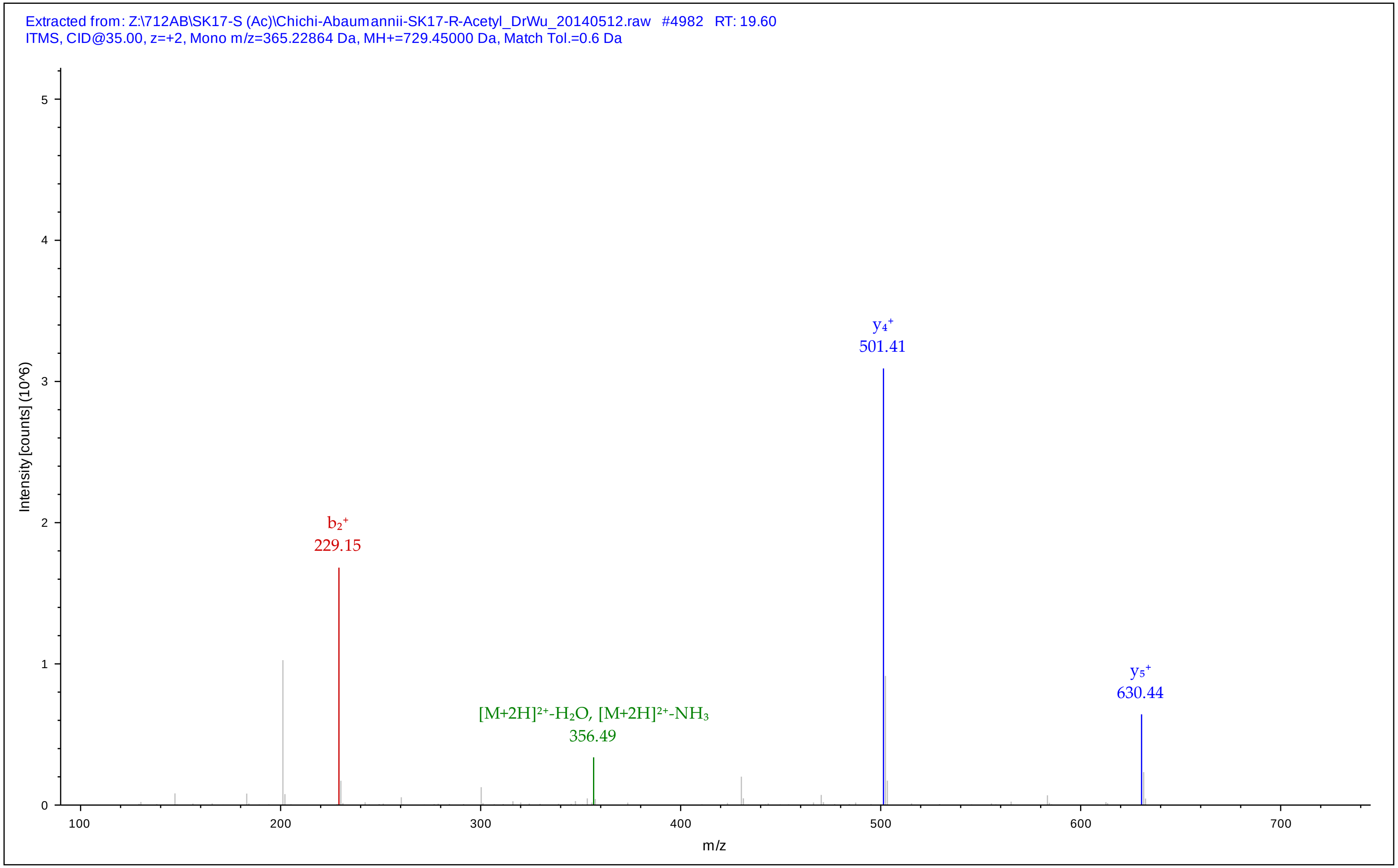

peg.564 20560

#1	b^+	b^{2+}	b^{3+}	Seq.	y^+	y^{2+}	y^{3+}	#2
1	148.07570	74.54149	50.03008	F				18
2	249.12338	125.06533	83.71264	T	2053.03498	1027.02113	685.01651	17
3	362.20745	181.60736	121.40733	L	1951.98730	976.49729	651.33395	16
4	532.31298	266.66013	178.10918	K-Acetyl	1838.90323	919.95525	613.63926	15
5	646.35591	323.68159	216.12349	N	1668.79769	834.90248	556.93741	14
6	717.39303	359.20015	239.80253	A	1554.75476	777.88102	518.92310	13
7	880.45635	440.73181	294.15697	Y	1483.71764	742.36246	495.24406	12
8	993.54042	497.27385	331.85166	I	1320.65432	660.83080	440.88962	11
9	1107.58335	554.29531	369.86597	N	1207.57025	604.28876	403.19493	10
10	1263.68447	632.34587	421.89967	R	1093.52732	547.26730	365.18062	9
11	1378.71142	689.85935	460.24199	D	937.42620	469.21674	313.14692	8
12	1525.77984	763.39356	509.26480	F	822.39925	411.70326	274.80460	7
13	1640.80679	820.90703	547.60711	D	675.33083	338.16905	225.78179	6
14	1754.84972	877.92850	585.62142	N	560.30388	280.65558	187.43948	5
15	1869.87667	935.44197	623.96374	D	446.26095	223.63411	149.42517	4
16	1940.91379	970.96053	647.64278	A	331.23400	166.12064	111.08285	3
17	2053.99786	1027.50257	685.33747	L	260.19688	130.60208	87.40381	2
18				K	147.11281	74.06004	49.70912	1

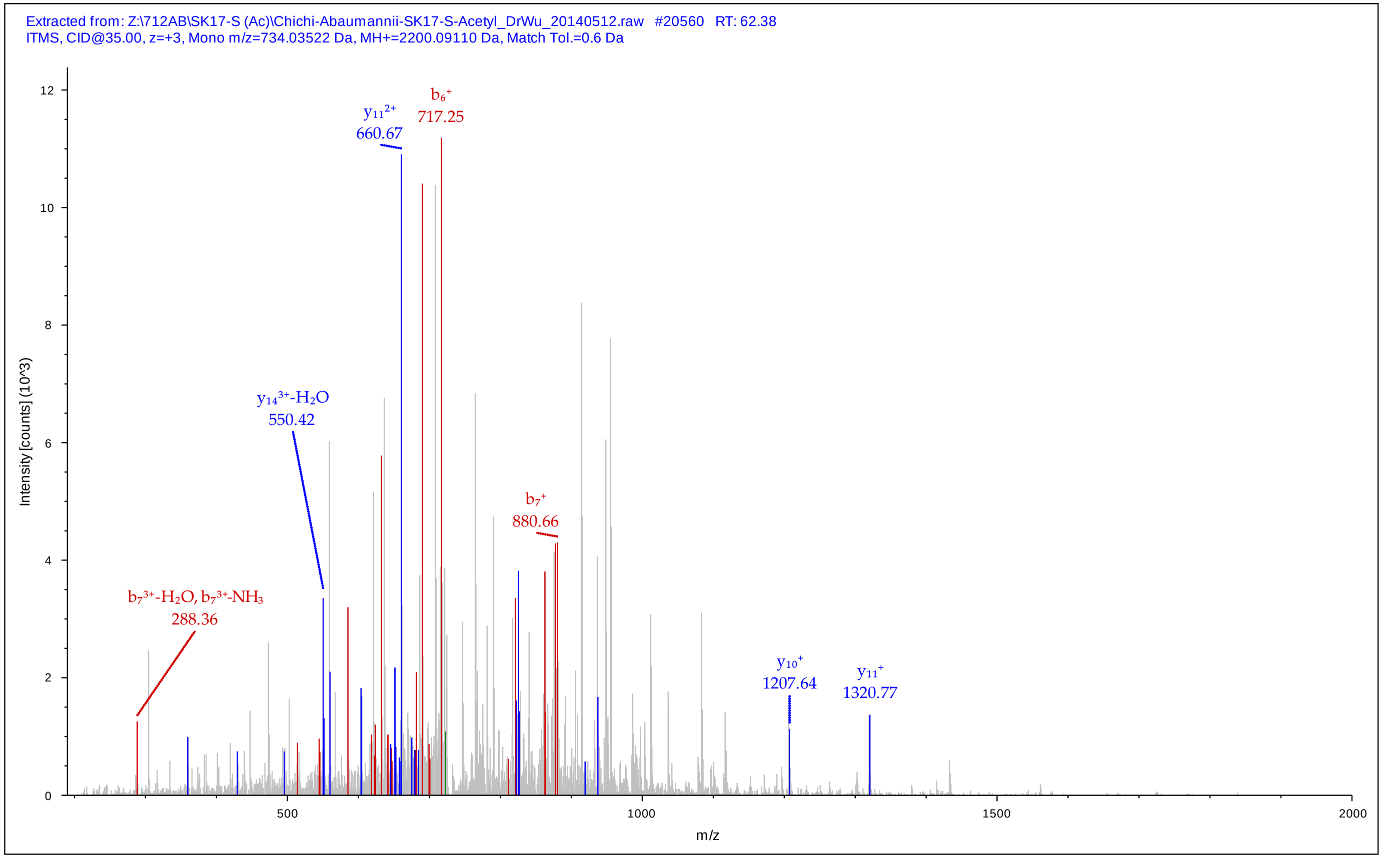

peg.569 25954

#1	b^+	b^{2+}	b^{3+}	Seq.	y^+	y^{2+}	y^{3+}	#2
1	129.10225	65.05476	43.70560	K				33
2	186.12372	93.56550	62.71276	G	3665.78275	1833.39501	1222.59910	32
3	299.20779	150.10753	100.40745	L	3608.76128	1804.88428	1203.59194	31
4	398.27621	199.64174	133.43025	V	3495.67721	1748.34224	1165.89725	30
5	513.30316	257.15522	171.77257	D	3396.60879	1698.80803	1132.87445	29
6	673.33381	337.17054	225.11612	C-Carbam···	3281.58184	1641.29456	1094.53213	28
7	774.38149	387.69438	258.79868	T	3121.55118	1561.27923	1041.18858	27
8	887.46556	444.23642	296.49337	L	3020.50350	1510.75539	1007.50602	26
9	1016.50816	508.75772	339.50757	E	2907.41943	1454.21335	969.81133	25
10	1113.56093	557.28410	371.85849	P	2778.37683	1389.69205	926.79713	24
11	1250.61984	625.81356	417.54480	H	2681.32406	1341.16567	894.44620	23
12	1365.64679	683.32703	455.88711	D	2544.26515	1272.63621	848.75990	22
13	1512.71521	756.86124	504.90992	F	2429.23820	1215.12274	810.41758	21
14	1599.74724	800.37726	533.92060	S	2282.16978	1141.58853	761.39478	20
15	1696.80001	848.90364	566.27152	P	2195.13775	1098.07251	732.38410	19
16	1783.83204	892.41966	595.28220	S	2098.08498	1049.54613	700.03318	18
17	1880.88481	940.94604	627.63312	P	2011.05295	1006.03011	671.02250	17
18	1979.95323	990.48025	660.65593	V	1914.00018	957.50373	638.67158	16
19	2108.01181	1054.50954	703.34212	Q	1814.93176	907.96952	605.64877	15
20	2221.09588	1111.05158	741.03681	L	1686.87318	843.94023	562.96258	14
21	2292.13300	1146.57014	764.71585	A	1573.78911	787.39819	525.26789	13
22	2420.19158	1210.59943	807.40204	Q	1502.75199	751.87963	501.58885	12
23	2567.22700	1284.11714	856.41385	M-Oxidation	1374.69341	687.85034	458.90265	11
24	2664.27977	1332.64352	888.76477	P	1227.65800	614.33264	409.89085	10
25	2777.36384	1389.18556	926.45946	L	1130.60523	565.80625	377.53993	9
26	2878.41152	1439.70940	960.14202	T	1017.52116	509.26422	339.84524	8
27	2991.49559	1496.25143	997.83671	L	916.47348	458.74038	306.16268	7
28	3105.53852	1553.27290	1035.85102	N	803.38941	402.19834	268.46799	6
29	3234.58112	1617.79420	1078.86522	E	689.34648	345.17688	230.45368	5
30	3349.60807	1675.30767	1117.20754	D	560.30388	280.65558	187.43948	4
31	3477.66665	1739.33696	1159.89373	Q	445.27693	223.14210	149.09716	3
32	3647.77218	1824.38973	1216.59558	K-Acetyl	317.21835	159.11281	106.41097	2
33				K	147.11281	74.06004	49.70912	1

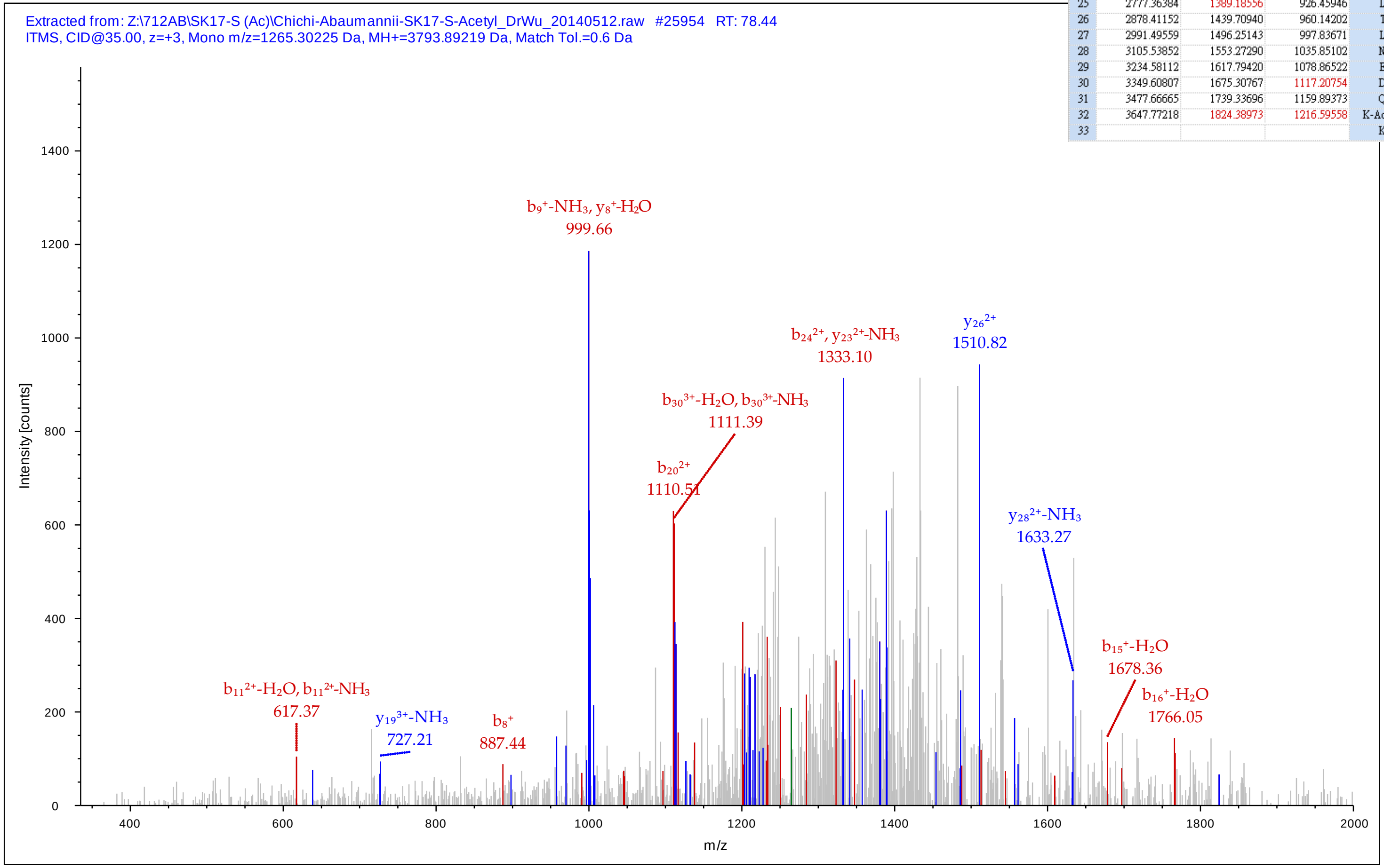

peg.629   19590

#1	b⁺	b^{2+}	Seq.	y⁺	y^{2+}	#2
1	156.10191	78.55459	L-Acetyl			17
2	227.13903	114.07315	A	1815.98115	908.49421	16
3	356.18163	178.59445	E	1744.94403	872.97565	15
4	512.28275	256.64501	R	1615.90143	808.45435	14
5	599.31478	300.16103	S	1459.80031	730.40379	13
6	670.35190	335.67959	A	1372.76828	686.88778	12
7	757.38393	379.19560	S	1301.73116	651.36922	11
8	828.42105	414.71416	A	1214.69913	607.85320	10
9	929.46873	465.23800	T	1143.66201	572.33464	9
10	1099.57427	550.29077	K-Acetyl	1042.61433	521.81080	8
11	1227.63285	614.32006	Q	872.50880	436.75804	7
12	1340.71692	670.86210	I	744.45022	372.72875	6
13	1469.75952	735.38340	E	631.36615	316.18671	5
14	1570.80720	785.90724	T	502.32355	251.66541	4
15	1683.89127	842.44927	L	401.27587	201.14157	3
16	1782.95969	891.98348	Y	288.19180	144.59954	2
17			K-Acetyl	189.12338	95.06533	1

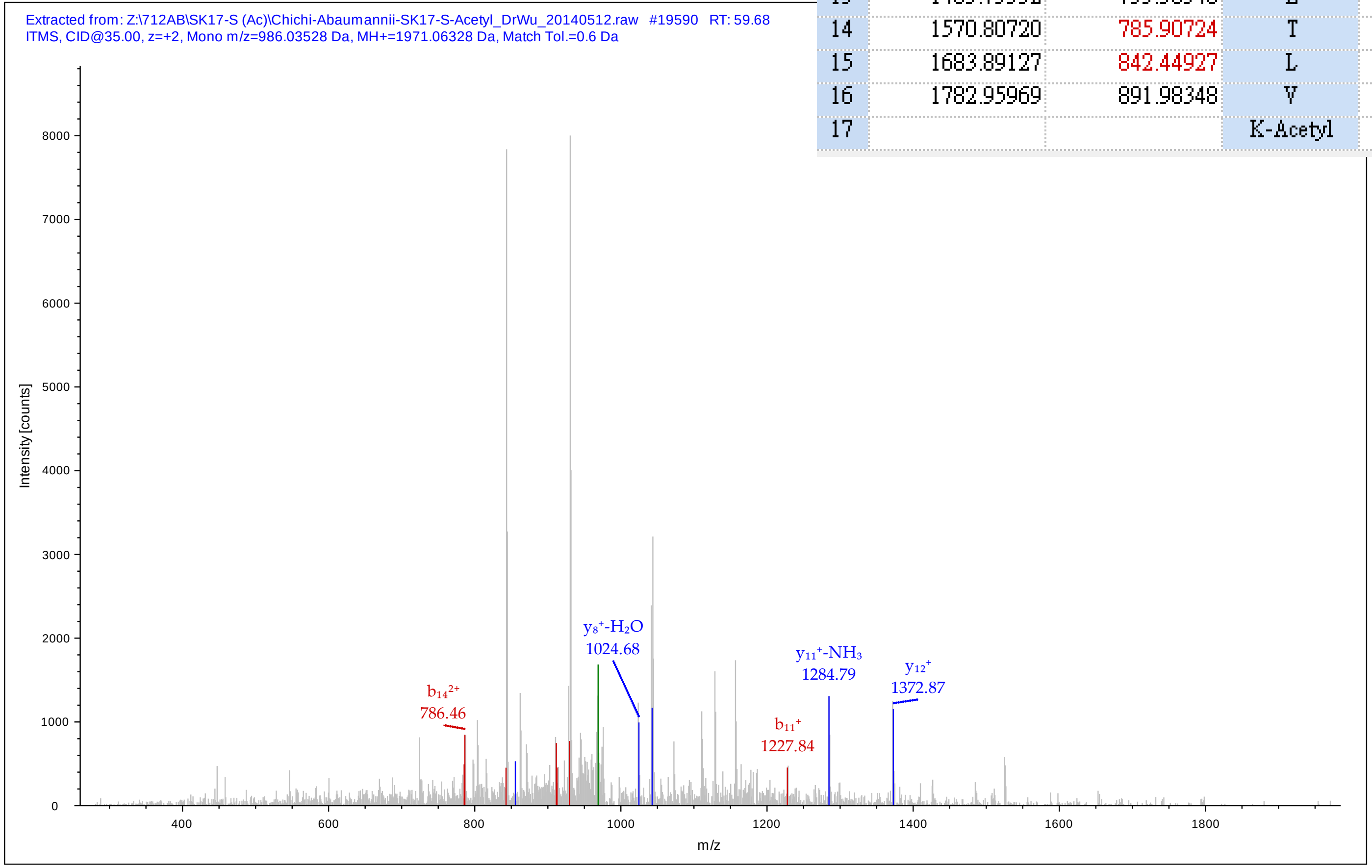

peg.635   13882

#1	b^+	b^{2+}	b^{3+}	Seq.	y^+	y^{2+}	y^{3+}	#2
1	157.06077	79.03402	53.02511	N-Acetyl				20
2	272.08772	136.54750	91.36742	D	2212.12320	1106.56524	738.04592	19
3	359.11975	180.06351	120.37810	S	2097.09625	1049.05176	699.70360	18
4	472.20382	236.60555	158.07279	L	2010.06422	1005.53575	670.69292	17
5	601.24642	301.12685	201.08699	E	1896.98015	948.99371	632.99823	16
6	730.28902	365.64815	244.10119	E	1767.93755	884.47241	589.98403	15
7	817.32105	409.16416	273.11187	S	1638.89495	819.95111	546.96983	14
8	914.37382	457.69055	305.46279	P	1551.86292	776.43510	517.95916	13
9	1011.42659	506.21693	337.81371	P	1454.81015	727.90871	485.60823	12
10	1140.46919	570.73823	380.82791	E	1357.75738	679.38233	453.25731	11
11	1310.57473	655.79100	437.52976	K-Acetyl	1228.71478	614.86103	410.24311	10
12	1397.60676	699.30702	466.54044	S	1058.60925	529.80826	353.54127	9
13	1484.63879	742.82303	495.55111	S	971.57722	486.29225	324.53059	8
14	1597.72286	799.36507	533.24580	L	884.54519	442.77623	295.51991	7
15	1725.78144	863.39436	575.93200	Q	771.46112	386.23420	257.82522	6
16	1824.84986	912.92857	608.95480	V	643.40254	322.20491	215.13903	5
17	1937.93393	969.47060	646.64949	I	544.33412	272.67070	182.11622	4
18	2051.01800	1026.01264	684.34418	I	431.25005	216.12866	144.42153	3
19	2180.06060	1090.53394	727.35838	E	318.16598	159.58663	106.72684	2
20				K-Acetyl	189.12338	95.06533	63.71264	1

Extracted from: Z:\712AB\SK17-S (Ac)\Chichi-Abaumannii-SK17-S-Acetyl_DrWu_20140703.raw #13882 RT: 40.47
ITMS, CID@35.00, z=+3, Mono m/z=790.05933 Da, MH+=2368.16343 Da, Match Tol.=0.6 Da

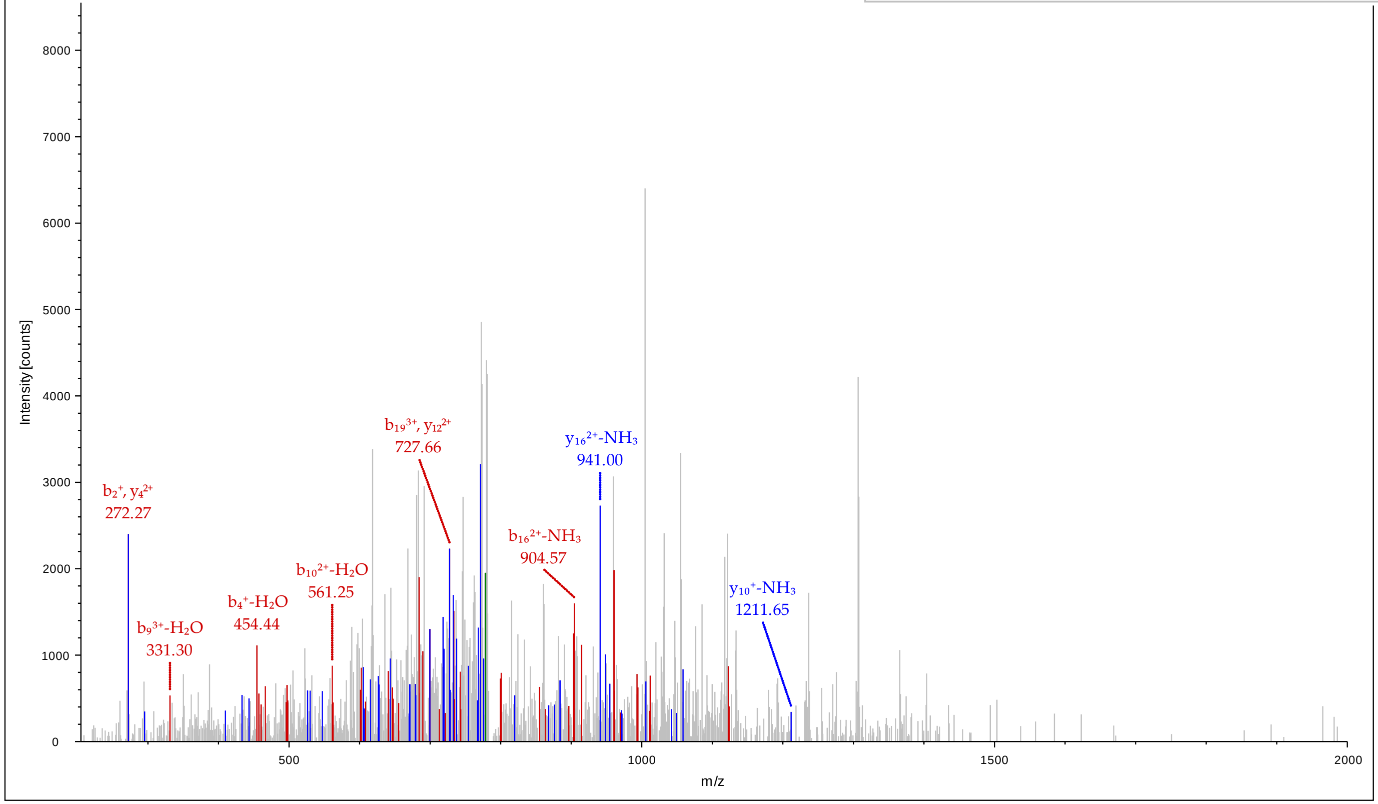

peg.657 7226

#1	b+	b2+	b3+	Seq.	y+	y2+	y3+	#2
1	164.07060	82.53894	55.36172	Y				19
2	327.13392	164.07060	109.71616	Y	2022.96155	1011.98441	674.99203	18
3	414.16595	207.58661	138.72683	S	1859.89823	930.45275	620.63759	17
4	471.18742	236.09735	157.73399	G	1772.86620	886.93674	591.62692	16
5	542.22454	271.61591	181.41303	A	1715.84473	858.42600	572.61976	15
6	657.25149	329.12938	219.75535	D	1644.80761	822.90744	548.94072	14
7	714.27296	357.64012	238.76250	G	1529.78066	765.39397	510.59840	13
8	815.32064	408.16396	272.44506	T	1472.75919	736.88323	491.59125	12
9	985.42617	493.21672	329.14691	K-Acetyl	1371.71151	686.35939	457.90869	11
10	1098.51024	549.75876	366.84160	L	1201.60597	601.30662	401.20684	10
11	1211.59431	606.30079	404.53629	L	1088.52190	544.76459	363.51215	9
12	1310.66273	655.83500	437.55909	V	975.43783	488.22255	325.81746	8
13	1423.74680	712.37704	475.25378	L	876.36941	438.68834	292.79465	7
14	1538.77375	769.89051	513.59610	D	763.28534	382.14631	255.09996	6
15	1653.80070	827.40399	551.93842	D	648.25839	324.63283	216.75765	5
16	1768.82765	884.91746	590.28073	D	533.23144	267.11936	178.41533	4
17	1897.87025	949.43876	633.29493	E	418.20449	209.60588	140.07301	3
18	2011.91318	1006.46023	671.30924	N	289.16189	145.08458	97.05881	2
19				R	175.11896	88.06312	59.04450	1

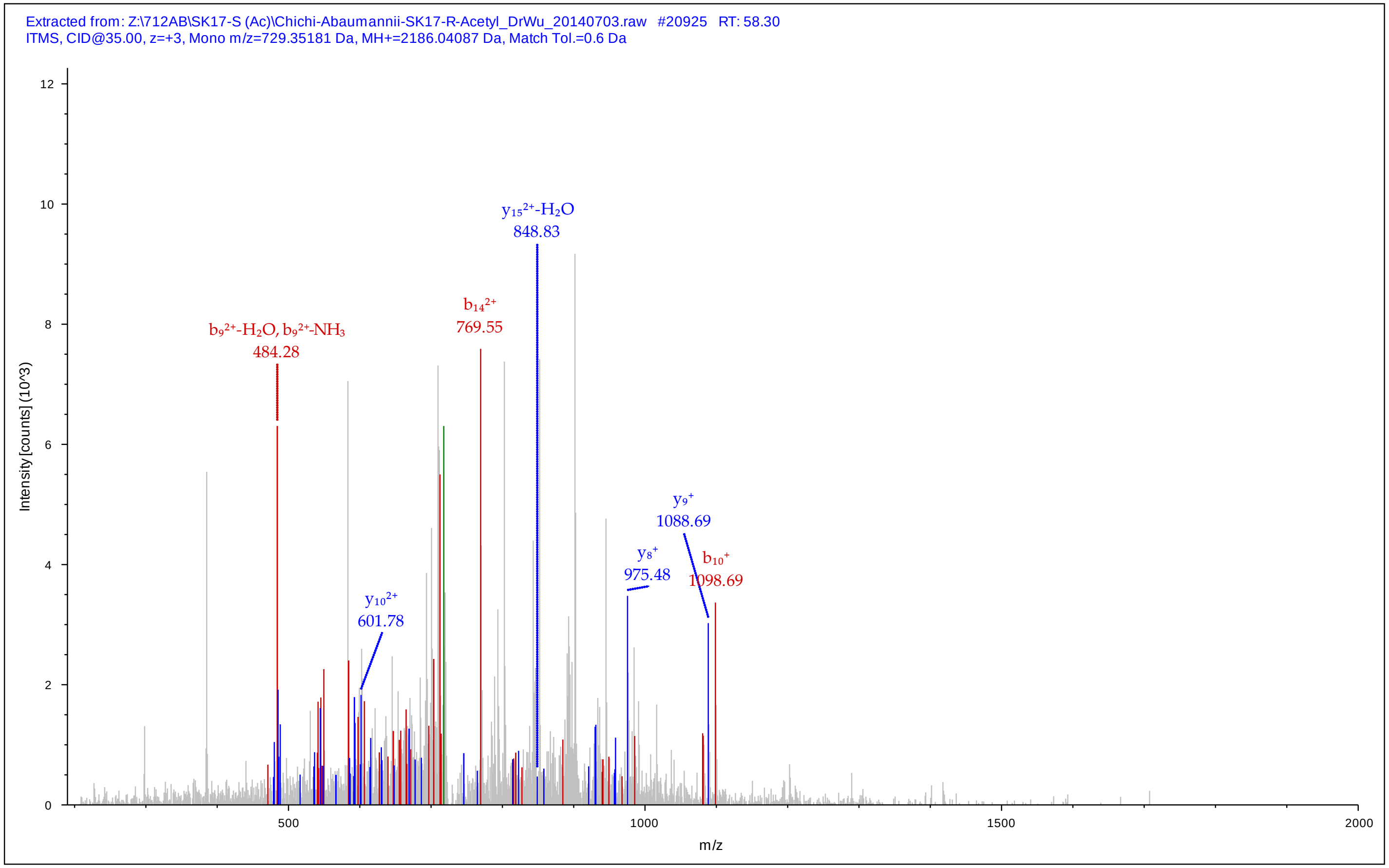

peg.711 3928

#1	b+	b2+	Seq.	y+	y2+	#2
1	102.05496	51.53112	T			14
2	215.13903	108.07315	L	1337.75366	669.38047	13
3	312.19180	156.59954	P	1224.66959	612.83843	12
4	441.23440	221.12084	E	1127.61682	564.31205	11
5	512.27152	256.63940	A	998.57422	499.79075	10
6	583.30864	292.15796	A	927.53710	464.27219	9
7	654.34576	327.67652	A	856.49998	428.75363	8
8	767.42983	384.21855	L	785.46286	393.23507	7
9	838.46695	419.73711	A	672.37879	336.69303	6
10	909.50407	455.25567	A	601.34167	301.17447	5
11	980.54119	490.77423	A	530.30455	265.65591	4
12	1094.58412	547.79570	N	459.26743	230.13735	3
13	1264.68965	632.84846	K-Acetyl	345.22450	173.11589	2
14			R	175.11896	88.06312	1

Extracted from: Z:\712AB\SK17-S (Ac)\Chichi-Abaumannii-SK17-S-Acetyl_DrWu_20140512.raw #3928 RT: 20.07
ITMS, CID@35.00, z=+2, Mono m/z=719.90942 Da, MH+=1438.81157 Da, Match Tol.=0.6 Da

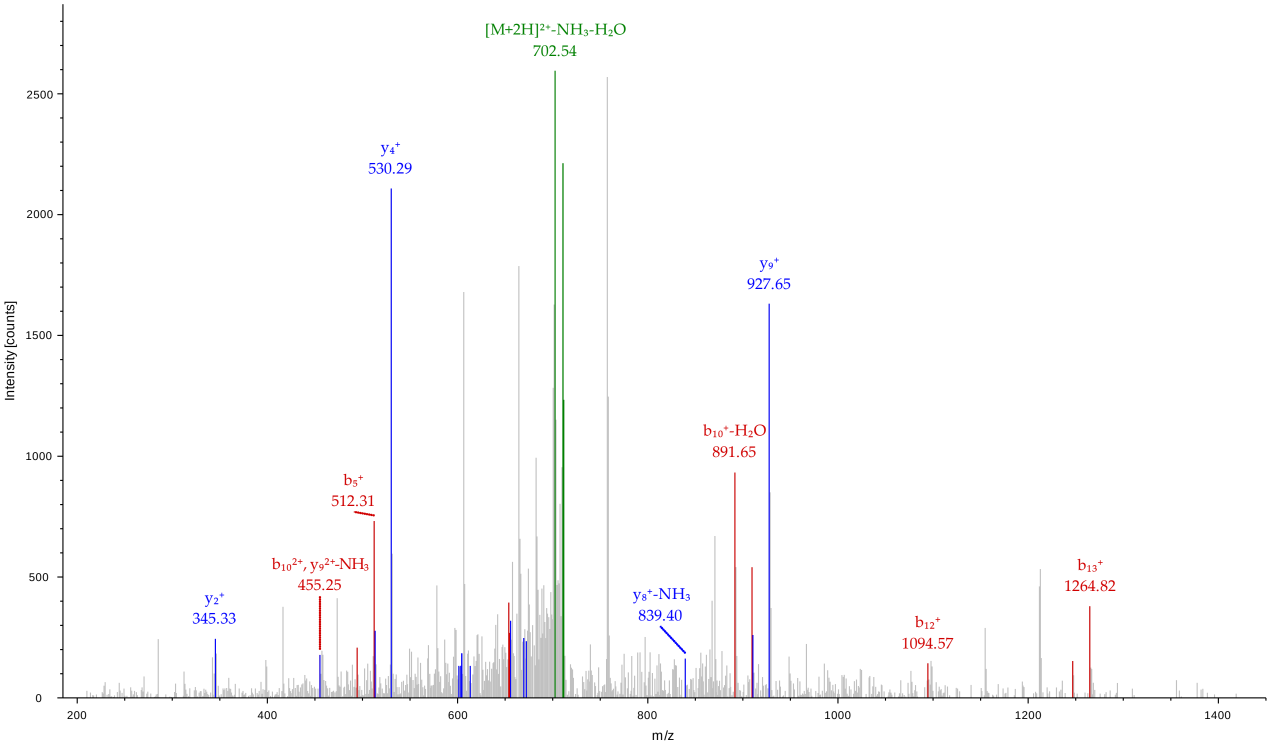

peg.723 21672

#1	b⁺	b²⁺	Seq.	y⁺	y²⁺	#2
1	199.11896	100.06312	R-Acetyl			11
2	328.16156	164.58442	E	1206.61138	603.80933	10
3	442.20449	221.60588	N	1077.56878	539.28803	9
4	570.26307	285.63517	Q	963.52585	482.26656	8
5	684.30600	342.65664	N	835.46727	418.23727	7
6	741.32747	371.16737	G	721.42434	361.21581	6
7	911.43301	456.22014	K-Acetyl	664.40287	332.70507	5
8	1058.50143	529.75435	F	494.29733	247.65230	4
9	1171.58550	586.29639	L	347.22891	174.11809	3
10	1258.61753	629.81240	S	234.14484	117.57606	2
11			K	147.11281	74.06004	1

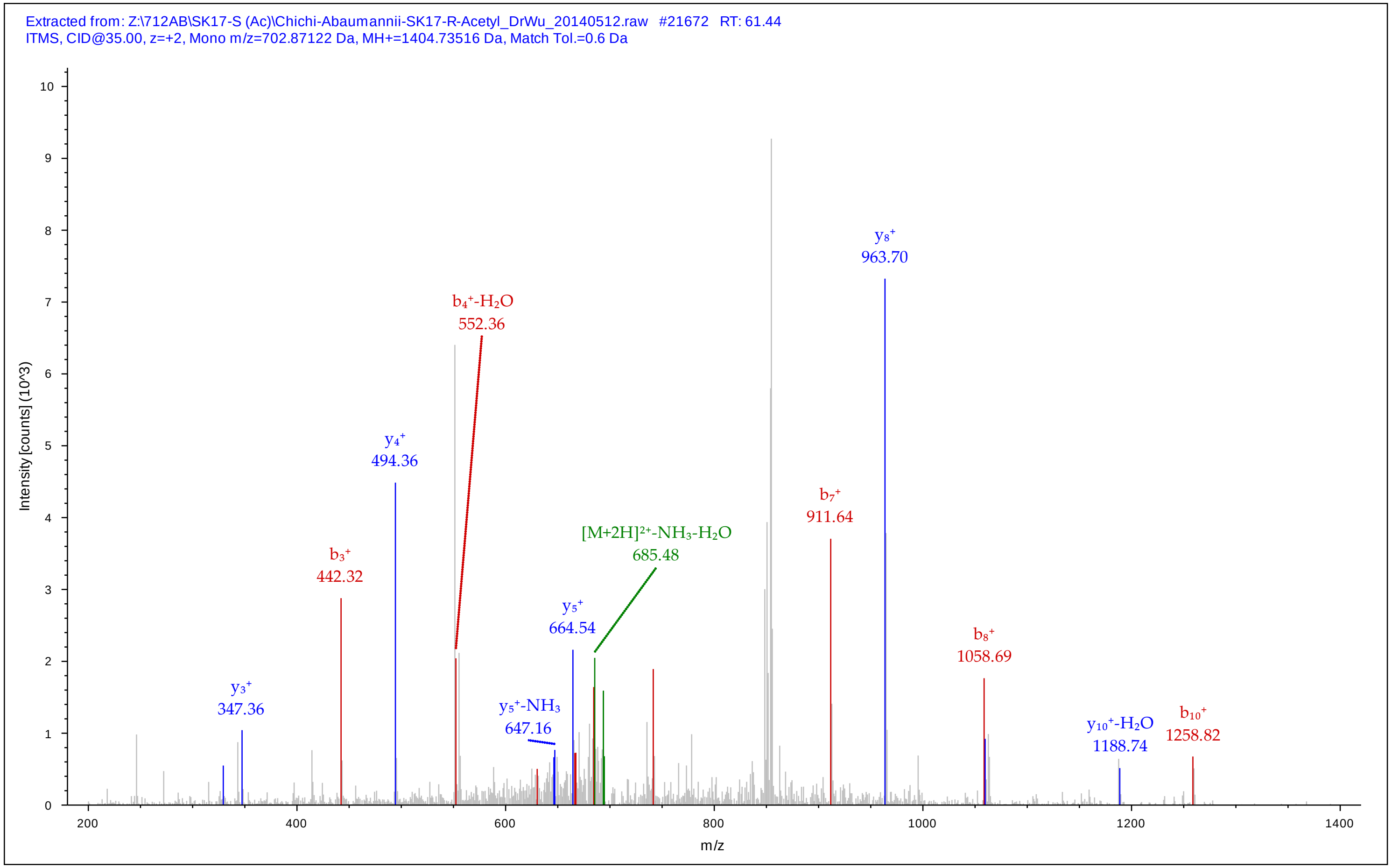

peg.723 19925

#1	b+	b2+	Seq.	y+	y2+	#2
1	199.11896	100.06312	R-Acetyl			11
2	328.16156	164.58442	E	1206.61138	603.80933	10
3	442.20449	221.60588	N	1077.56878	539.28803	9
4	570.26307	285.63517	Q	963.52585	482.26656	8
5	684.30600	342.65664	N	835.46727	418.23727	7
6	741.32747	371.16737	G	721.42434	361.21581	6
7	911.43301	456.22014	K-Acetyl	664.40287	332.70507	5
8	1058.50143	529.75435	F	494.29733	247.65230	4
9	1171.58550	586.29639	L	347.22891	174.11809	3
10	1258.61753	629.81240	S	234.14484	117.57606	2
11			K	147.11281	74.06004	1

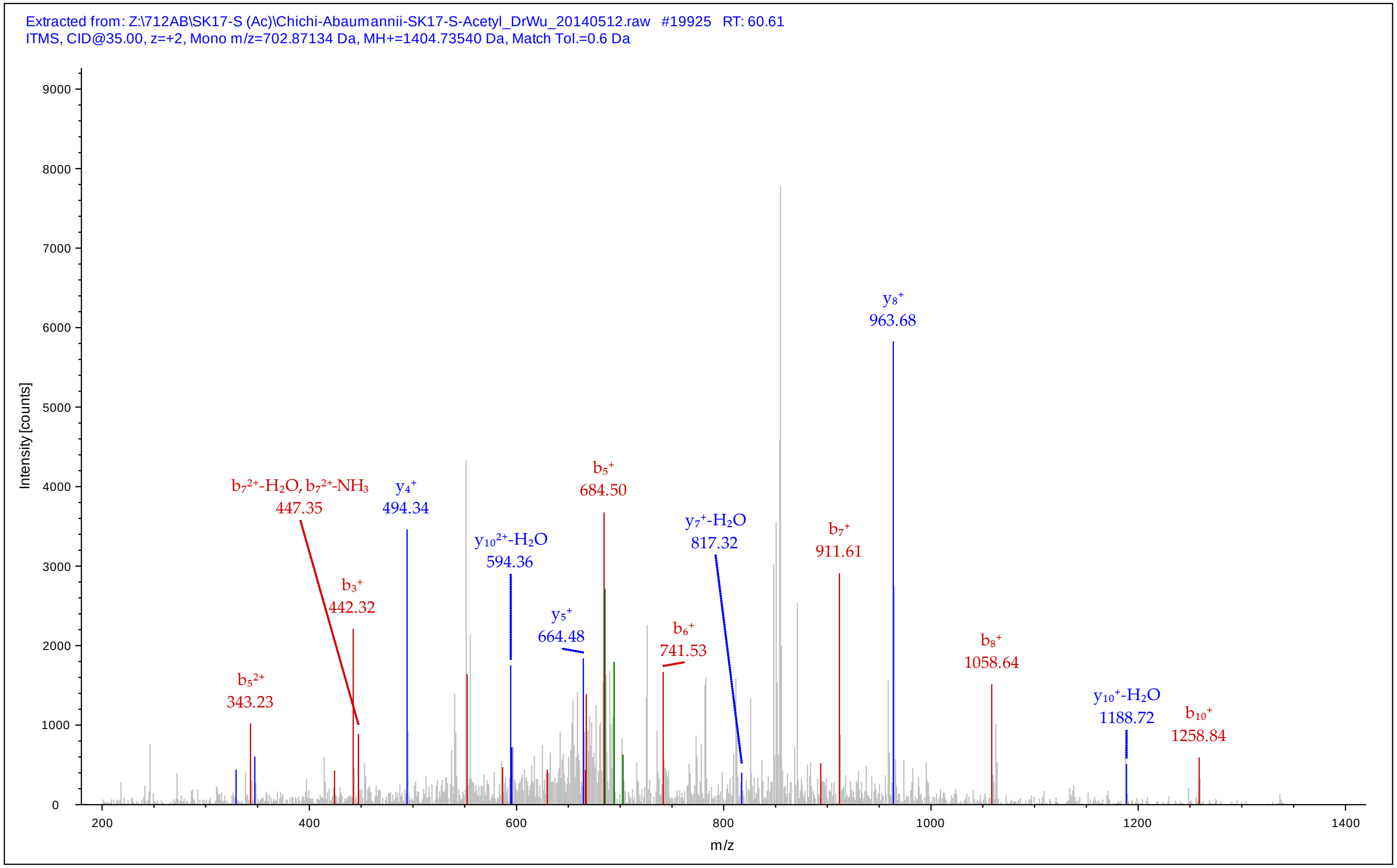

peg.764 6578

#1	b+	b2+	Seq.	y+	y2+	#2
1	72.04440	36.52584	A			8
2	186.08733	93.54730	N	914.49421	457.75074	7
3	299.17140	150.08934	I	800.45128	400.72928	6
4	428.21400	214.61064	E	687.36721	344.18724	5
5	598.31953	299.66340	K-Acetyl	558.32461	279.66594	4
6	669.35665	335.18196	A	388.21908	194.61318	3
7	797.41523	399.21125	Q	317.18196	159.09462	2
8			K-Acetyl	189.12338	95.06533	1

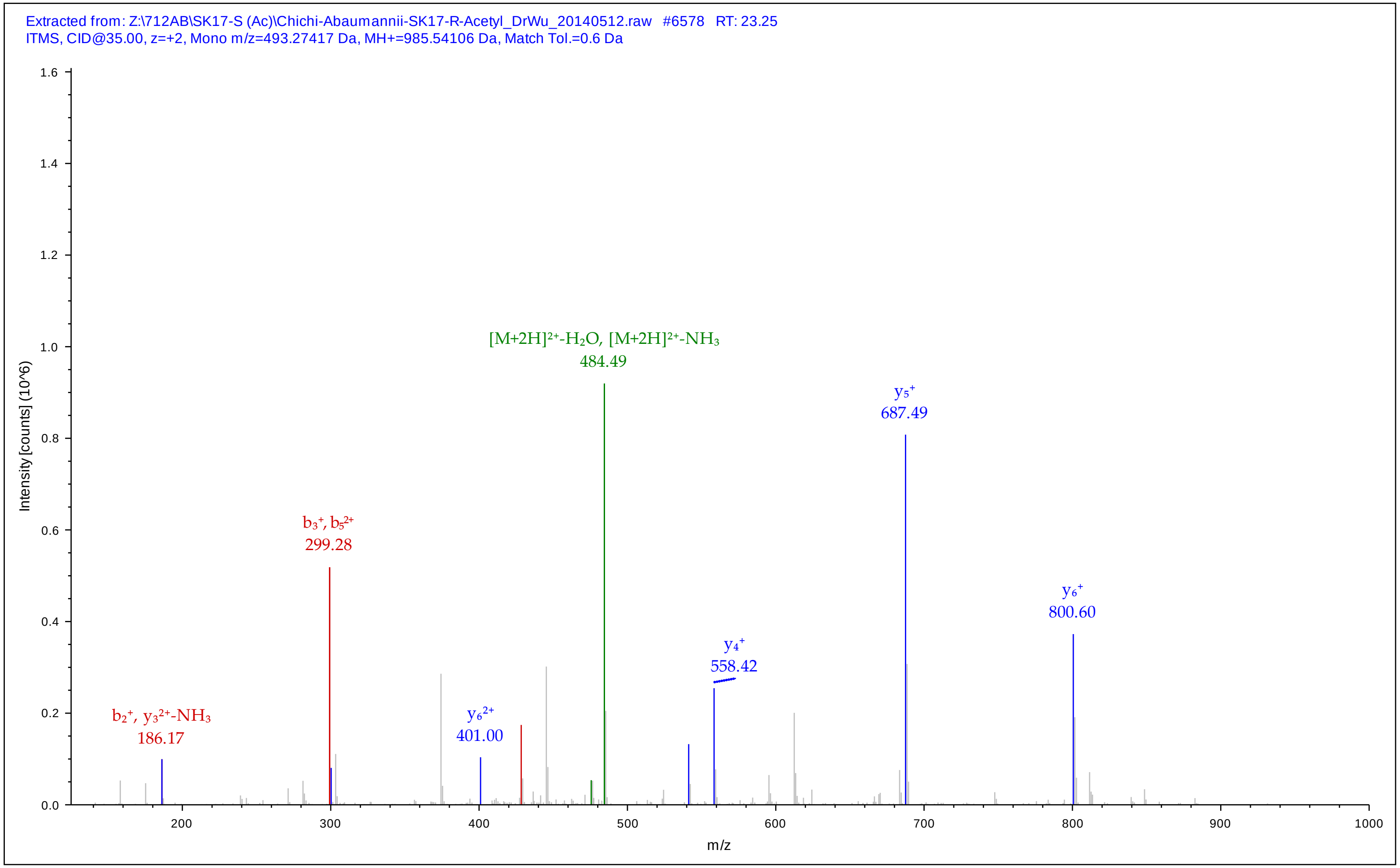

peg.843   8167

#1	b⁺	b²⁺	Seq.	y⁺	y²⁺	#2
1	171.11281	86.06004	K-Acetyl			14
2	357.19213	179.09970	W	1514.77849	757.89288	13
3	504.22755	252.61741	M-Oxidation	1328.69917	664.85322	12
4	603.29597	302.15162	V	1181.66375	591.33551	11
5	731.35455	366.18091	Q	1082.59533	541.80130	10
6	830.42297	415.71512	V	954.53675	477.77201	9
7	901.46009	451.23368	A	855.46833	428.23780	8
8	1014.54416	507.77572	L	784.43121	392.71924	7
9	1085.58128	543.29428	A	671.34714	336.17721	6
10	1182.63405	591.82066	P	600.31002	300.65865	5
11	1296.67698	648.84213	N	503.25725	252.13226	4
12	1424.73556	712.87142	Q	389.21432	195.11080	3
13	1538.77849	769.89288	N	261.15574	131.08151	2
14			K	147.11281	74.06004	1

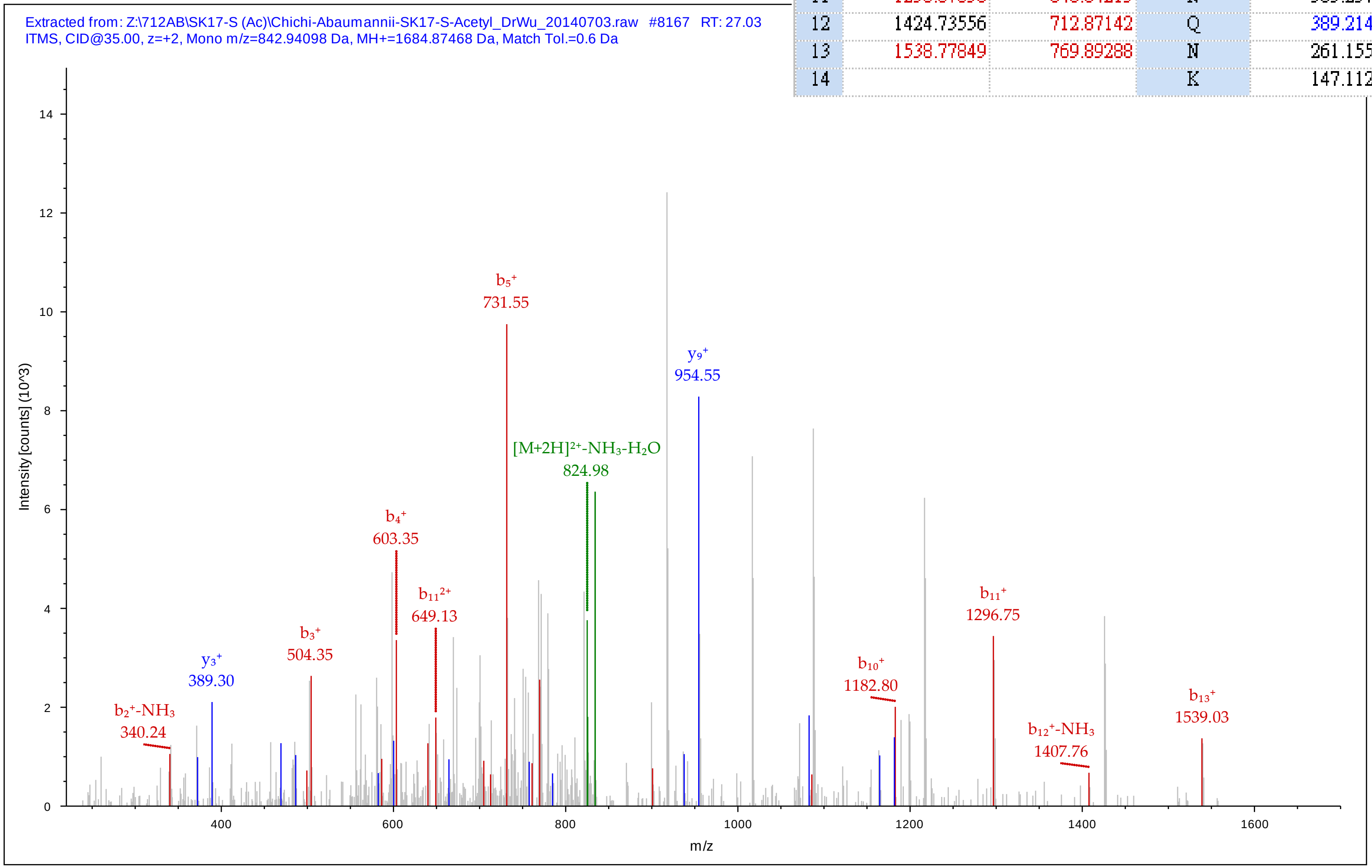

peg.852 17246

#1	b⁺	b²⁺	Seq.	y⁺	y²⁺	#2
1	156.10191	78.55459	L-Acetyl			12
2	271.12886	136.06807	D	1428.68622	714.84675	11
3	418.16428	209.58578	M-Oxidation	1313.65927	657.33327	10
4	505.19631	253.10179	S	1166.62385	583.81556	9
5	618.28038	309.64383	L	1079.59182	540.29955	8
6	765.31579	383.16153	M-Oxidation	966.50775	483.75751	7
7	862.36856	431.68792	P	819.47234	410.23981	6
8	1032.47410	516.74069	K-Acetyl	722.41957	361.71342	5
9	1195.53742	598.27235	Y	552.31403	276.66065	4
10	1323.63239	662.31983	K	389.25071	195.12899	3
11	1437.67532	719.34130	N	261.15574	131.08151	2
12			K	147.11281	74.06004	1

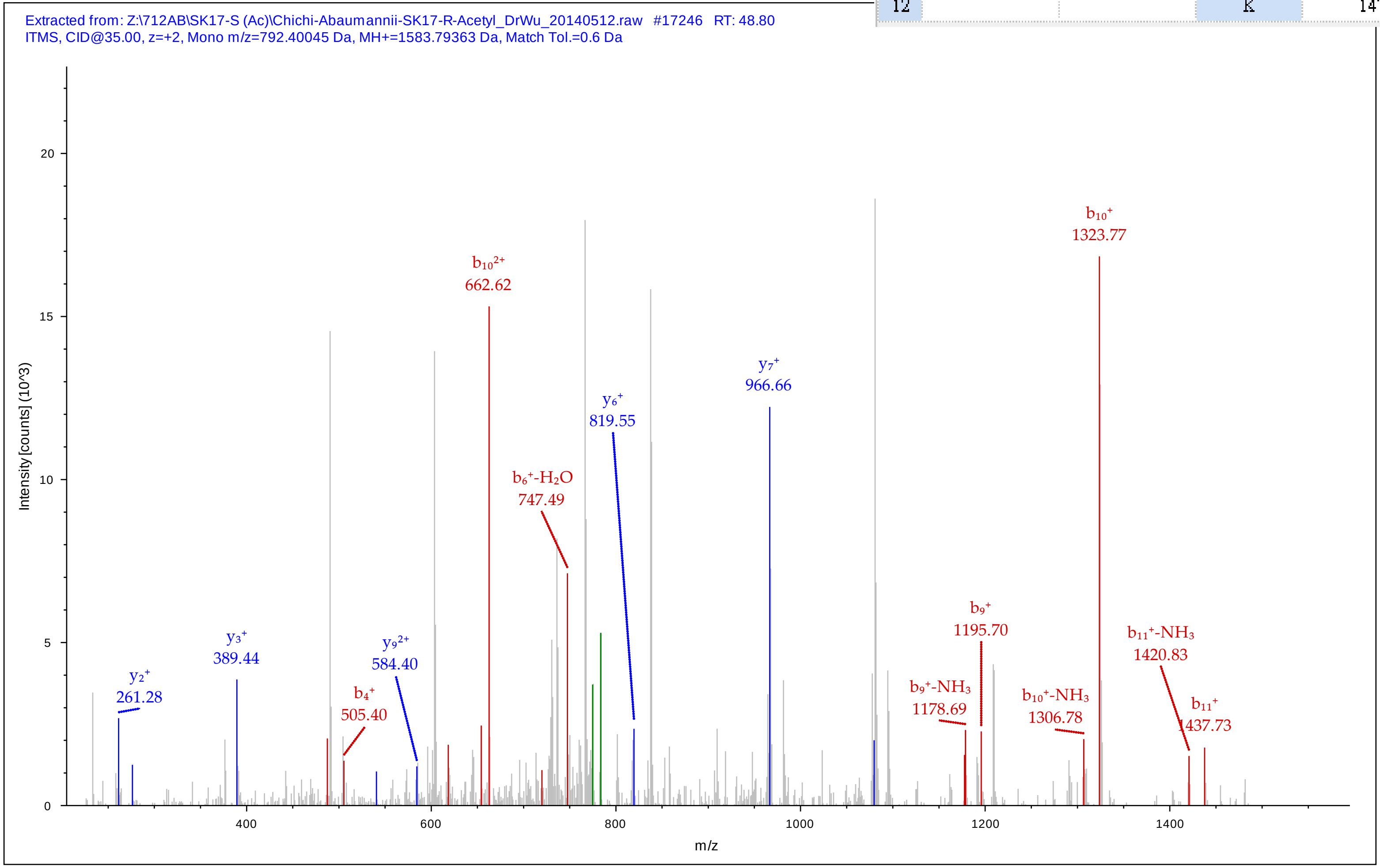

peg.852 17304

#1	b⁺	b²⁺	Seq.	y⁺	y²⁺	#2
1	156.10191	78.55459	L-Acetyl			12
2	271.12886	136.06807	D	1428.68622	714.84675	11
3	418.16428	209.58578	M-Oxidation	1313.65927	657.33327	10
4	505.19631	253.10179	S	1166.62385	583.81556	9
5	618.28038	309.64383	L	1079.59182	540.29955	8
6	765.31579	383.16153	M-Oxidation	966.50775	483.75751	7
7	862.36856	431.68792	P	819.47234	410.23981	6
8	1032.47410	516.74069	K-Acetyl	722.41957	361.71342	5
9	1195.53742	598.27235	Y	552.31403	276.66065	4
10	1323.63239	662.31983	K	389.25071	195.12899	3
11	1437.67532	719.34130	N	261.15574	131.08151	2
12			K	147.11281	74.06004	1

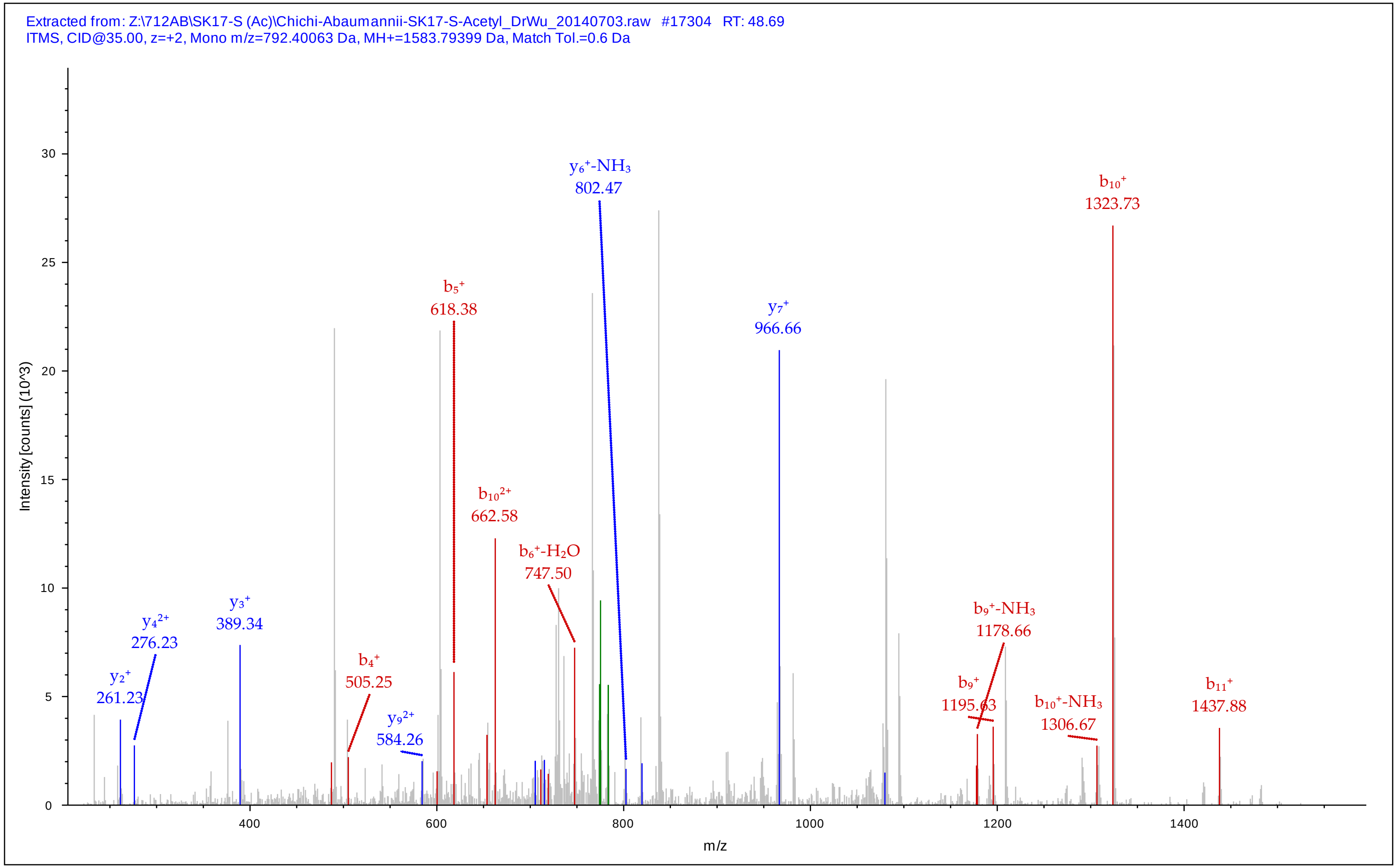

peg.884 26307

#1	b^+	b^{2+}	b^{3+}	Seq.	y^+	y^{2+}	y^{3+}	#2
1	156.10191	78.55459	52.70549	L-Acetyl				31
2	227.13903	114.07315	76.38453	A	3383.66899	1692.33813	1128.56118	30
3	340.22310	170.61519	114.07922	I	3312.63187	1656.81957	1104.88214	29
4	503.28642	252.14685	168.43366	Y	3199.54780	1600.27754	1067.18745	28
5	618.31337	309.66032	206.77597	D	3036.48448	1518.74588	1012.83301	27
6	689.35049	345.17888	230.45501	A	2921.45753	1461.23240	974.49069	26
7	760.38761	380.69744	254.13405	A	2850.42041	1425.71384	950.81165	25
8	907.42303	454.21515	303.14586	M-Oxidation	2779.38329	1390.19528	927.13261	24
9	1063.52415	532.26571	355.17957	R	2632.34788	1316.67758	878.12081	23
10	1226.58747	613.79737	409.53401	Y	2476.24676	1238.62702	826.08710	22
11	1354.64605	677.82666	452.22020	Q	2313.18344	1157.09536	771.73266	21
12	1482.70463	741.85595	494.90639	Q	2185.12486	1093.06607	729.04647	20
13	1611.74723	806.37725	537.92059	E	2057.06628	1029.03678	686.36028	19
14	1748.80614	874.90671	583.60690	H	1928.02368	964.51548	643.34608	18
15	1849.85382	925.43055	617.28946	T	1790.96477	895.98602	597.65977	17
16	1946.90659	973.95693	649.64038	P	1689.91709	845.46218	563.97721	16
17	2059.99066	1030.49897	687.33507	L	1592.86432	796.93580	531.62629	15
18	2159.05908	1080.03318	720.35788	V	1479.78025	740.39376	493.93160	14
19	2272.14315	1136.57521	758.05257	I	1380.71183	690.85955	460.90879	13
20	2385.22722	1193.11725	795.74726	I	1267.62776	634.31752	423.21410	12
21	2456.26434	1228.63581	819.42630	A	1154.54369	577.77548	385.51941	11
22	2513.28581	1257.14654	838.43345	G	1083.50657	542.25692	361.84037	10
23	2683.39134	1342.19931	895.13530	K-Acetyl	1026.48510	513.74619	342.83322	9
24	2812.43394	1406.72061	938.14950	E	856.37956	428.69342	286.13137	8
25	2975.49726	1488.25227	992.50394	Y	727.33696	364.17212	243.11717	7
26	3032.51873	1516.76300	1011.51109	G	564.27364	282.64046	188.76273	6
27	3133.56641	1567.28684	1045.19365	T	507.25217	254.12972	169.75557	5
28	3190.58788	1595.79758	1064.20081	G	406.20449	203.60588	136.07301	4
29	3277.61991	1639.31359	1093.21149	S	349.18302	175.09515	117.06586	3
30	3364.65194	1682.82961	1122.22216	S	262.15099	131.57913	88.05518	2
31				R	175.11896	88.06312	59.04450	1

Extracted from: Z:\712AB\SK17-S (Ac)\Chichi-Abaumannii-SK17-R-Acetyl_DrWu_20140703.raw #26307 RT: 72.04
ITMS, CID@35.00, z=+3, Mono m/z=1180.27026 Da, MH+=3538.79624 Da, Match Tol.=0.6 Da

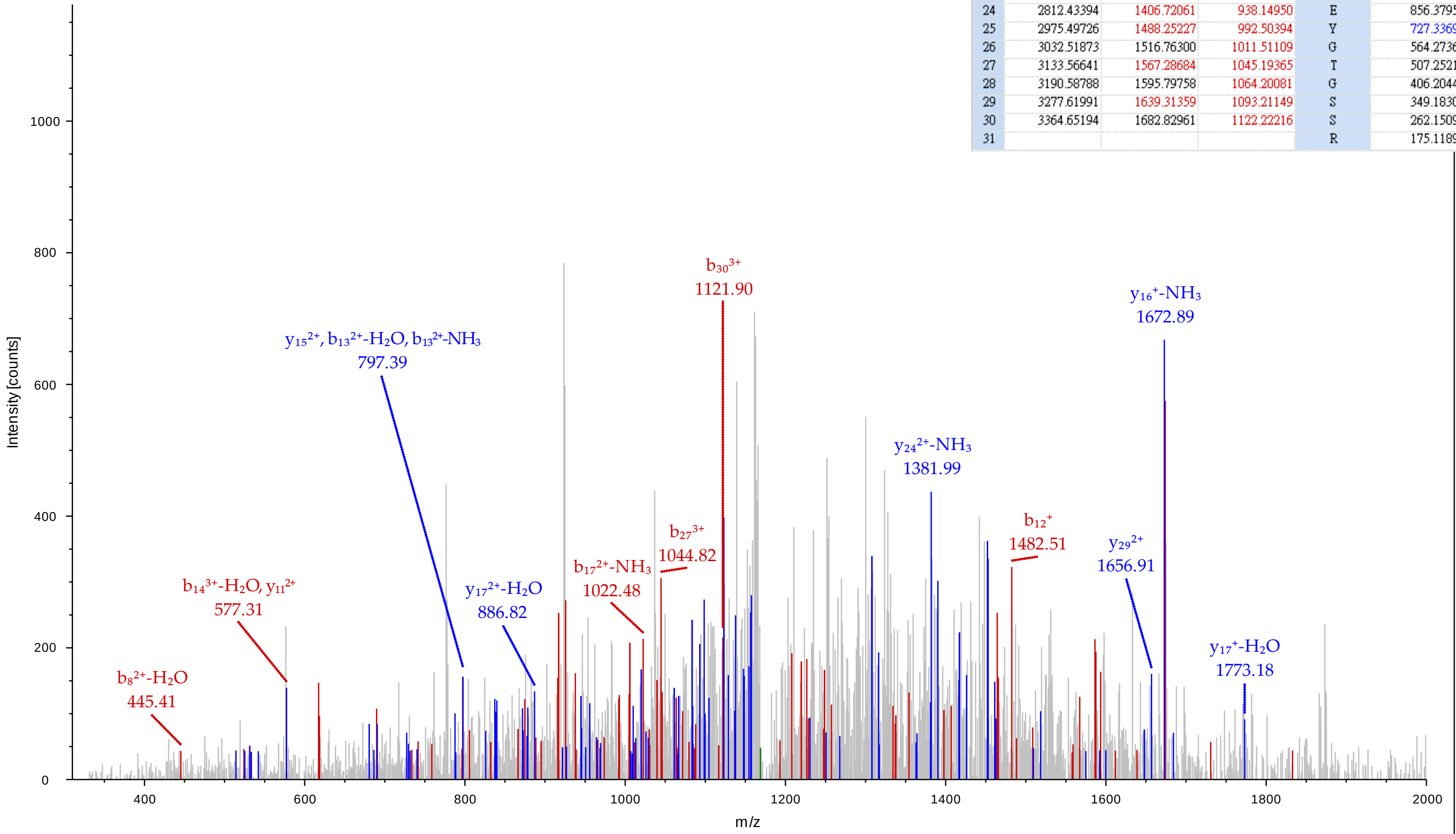

peg.893 29988

#1	b^+	b^{2+}	b^{3+}	Seq.	y^+	y^{2+}	y^{3+}	#2
1	190.08626	95.54677	64.03360	F-Acetyl				36
2	305.11321	153.06024	102.37592	D	3971.96629	1986.48678	1324.66028	35
3	433.17179	217.08953	145.06211	Q	3856.93934	1928.97331	1286.31796	34
4	490.19326	245.60027	164.06927	G	3728.88076	1864.94402	1243.63177	33
5	591.24094	296.12411	197.75183	T	3671.85929	1836.43328	1224.62461	32
6	738.30936	369.65832	246.77464	F	3570.81161	1785.90944	1190.94205	31
7	809.34648	405.17688	270.45368	A	3423.74319	1712.37523	1141.91925	30
8	946.40539	473.70633	316.13998	H	3352.70607	1676.85667	1118.24021	29
9	1047.45307	524.23017	349.82254	T	3215.64716	1608.32722	1072.55390	28
10	1160.53714	580.77221	387.51723	L	3114.59948	1557.80338	1038.87134	27
11	1247.56917	624.28822	416.52791	S	3001.51541	1501.26134	1001.17665	26
12	1360.65324	680.83026	454.22260	L	2914.48338	1457.74533	972.16598	25
13	1507.72166	754.36447	503.24540	F	2801.39931	1401.20329	934.47129	24
14	1636.76426	818.88577	546.25960	E	2654.33089	1327.66908	885.44848	23
15	1749.84833	875.42780	583.95429	I	2525.28829	1263.14778	842.43428	22
16	1919.95387	960.48057	640.65614	K-Acetyl	2412.20422	1206.60575	804.73959	21
17	2090.05940	1045.53334	697.35798	K-Acetyl	2242.09869	1121.55298	748.03775	20
18	2187.11217	1094.05972	729.70891	P	2071.99315	1036.50021	691.33590	19
19	2274.14420	1137.57574	758.71958	S	1974.94038	987.97383	658.98498	18
20	2345.18132	1173.09430	782.39862	A	1887.90835	944.45781	629.97430	17
21	2508.24464	1254.62596	836.75306	Y	1816.87123	908.93925	606.29526	16
22	2609.29232	1305.14980	870.43562	T	1653.80791	827.40759	551.94082	15
23	2724.31927	1362.66327	908.77794	D	1552.76023	776.88375	518.25826	14
24	2837.40334	1419.20531	946.47263	L	1437.73328	719.37028	479.91594	13
25	2936.47176	1468.73952	979.49544	V	1324.64921	662.82824	442.22125	12
26	3037.51944	1519.26336	1013.17800	T	1225.58079	613.29403	409.19845	11
27	3151.56237	1576.28482	1051.19231	N	1124.53311	562.77019	375.51589	10
28	3264.64644	1632.82686	1088.88700	I	1010.49018	505.74873	337.50158	9
29	3427.70976	1714.35852	1143.24144	Y	897.40611	449.20669	299.80689	8
30	3528.75744	1764.88236	1176.92400	T	734.34279	367.67503	245.45245	7
31	3615.78947	1808.39837	1205.93467	S	633.29511	317.15119	211.76989	6
32	3672.81094	1836.90911	1224.94183	G	546.26308	273.63518	182.75921	5
33	3729.83241	1865.41984	1243.94899	G	489.24161	245.12444	163.75205	4
34	3858.87501	1929.94114	1286.96319	E	432.22014	216.61371	144.74490	3
35	3986.93359	1993.97043	1329.64938	Q	303.17754	152.09241	101.73070	2
36				R	175.11896	88.06312	59.04450	1

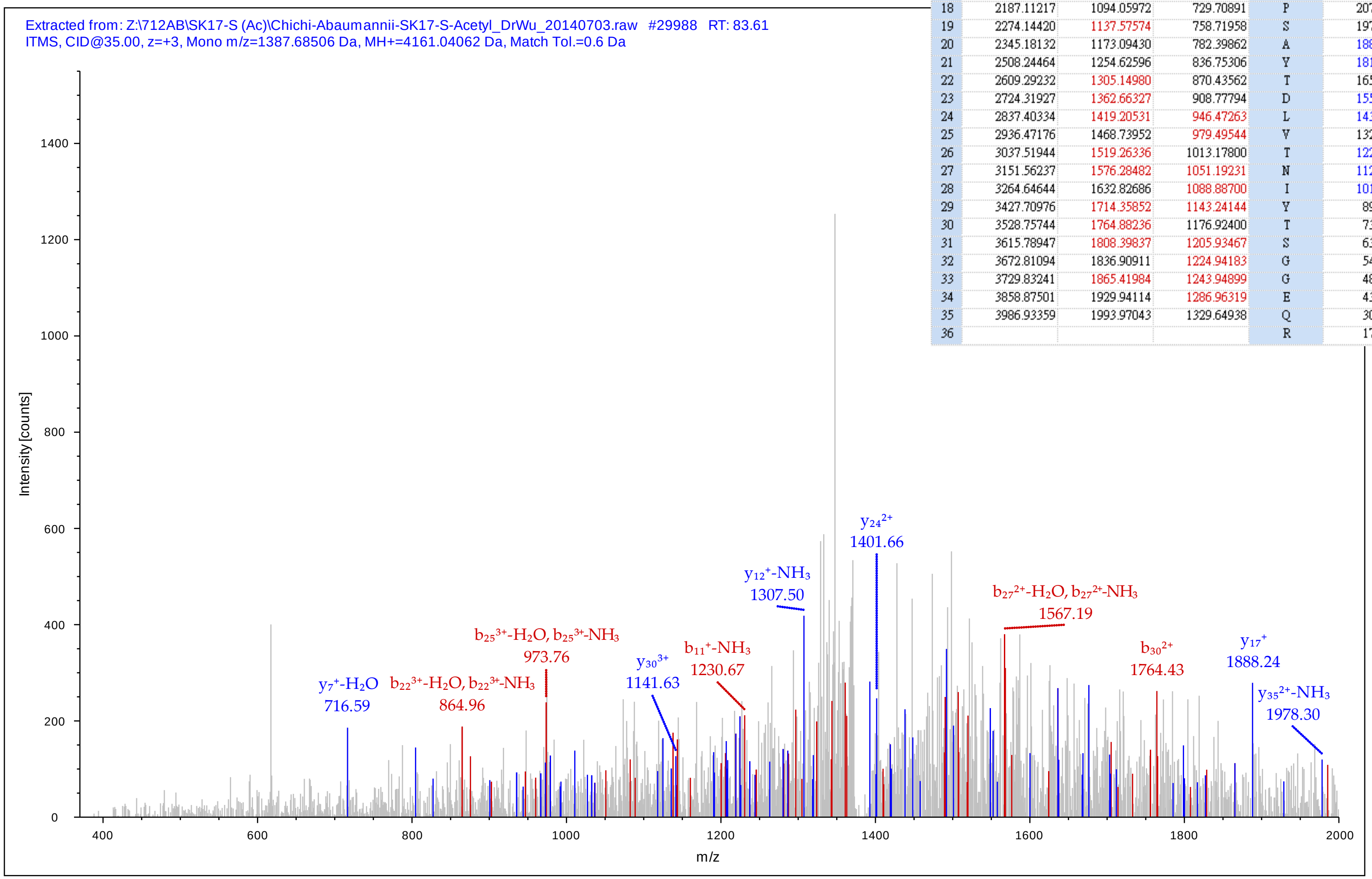

peg.905 21846

#1	b⁺	b²⁺	b³⁺	Seq.	y⁺	y²⁺	y³⁺	#2
1	100.03931	50.52329	34.01795	G-Acetyl				23
2	213.12338	107.06533	71.71264	L	2731.38011	1366.19369	911.13155	22
3	376.18670	188.59699	126.06708	Y	2618.29604	1309.65166	873.43686	21
4	539.25002	270.12865	180.42152	Y	2455.23272	1228.12000	819.08242	20
5	640.29770	320.65249	214.10408	T	2292.16940	1146.58834	764.72798	19
6	796.39882	398.70305	266.13779	R	2191.12172	1096.06450	731.04542	18
7	895.46724	448.23726	299.16060	V	2035.02060	1018.01394	679.01172	17
8	982.49927	491.75327	328.17127	S	1935.95218	968.47973	645.98891	16
9	1111.54187	556.27457	371.18547	E	1848.92015	924.96371	616.97823	15
10	1242.58237	621.79482	414.86564	M	1719.87755	860.44241	573.96403	14
11	1355.66644	678.33686	452.56033	L	1588.83705	794.92216	530.28387	13
12	1412.68791	706.84759	471.56749	G	1475.75298	738.38013	492.58918	12
13	1541.73051	771.36889	514.58169	E	1418.73151	709.86939	473.58202	11
14	1711.83605	856.42166	571.28353	K-Acetyl	1289.68891	645.34809	430.56782	10
15	1858.90447	929.95587	620.30634	F	1119.58337	560.29532	373.86597	9
16	1995.96338	998.48533	665.99264	H	972.51495	486.76111	324.84317	8
17	2109.04745	1055.02736	703.68733	I	835.45604	418.23166	279.15686	7
18	2224.07440	1112.54084	742.02965	D	722.37197	361.68962	241.46217	6
19	2353.11700	1177.06214	785.04385	E	607.34502	304.17615	203.11986	5
20	2424.15412	1212.58070	808.72289	A	478.30242	239.65485	160.10566	4
21	2571.22254	1286.11491	857.74570	F	407.26530	204.13629	136.42662	3
22	2684.30661	1342.65694	895.44039	L	260.19688	130.60208	87.40381	2
23				K	147.11281	74.06004	49.70912	1

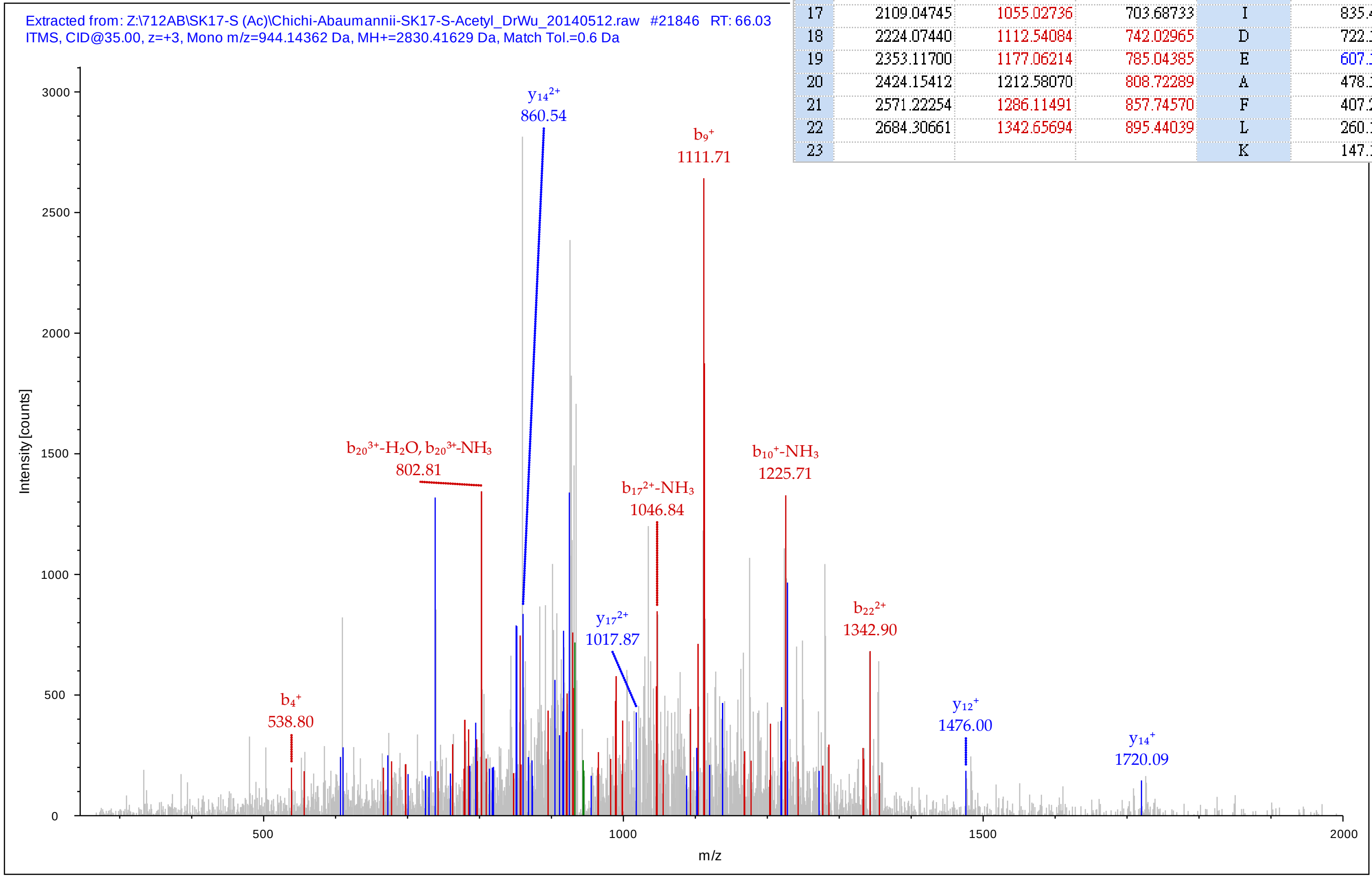

peg.964 28708

#1	b+	b2+	b3+	Seq.	y+	y2+	y3+	#2
1	100.03931	50.52329	34.01795	G-Acetyl				37
2	213.12338	107.06533	71.71264	I	3979.24950	1990.12839	1327.08802	36
3	314.17106	157.58917	105.39520	T	3866.16543	1933.58635	1289.39333	35
4	411.22383	206.11555	137.74613	P	3765.11775	1883.06251	1255.71077	34
5	526.25078	263.62903	176.08844	D	3668.06498	1834.53613	1223.35984	33
6	639.33485	320.17106	213.78313	L	3553.03803	1777.02265	1185.01753	32
7	776.39376	388.70052	259.46944	H	3439.95396	1720.48062	1147.32284	31
8	890.43669	445.72198	297.48375	N	3302.89505	1651.95116	1101.63653	30
9	947.45816	474.23272	316.49090	G	3188.85212	1594.92970	1063.62222	29
10	1034.49019	517.74873	345.50158	S	3131.83065	1566.41896	1044.61507	28
11	1147.57426	574.29077	383.19627	L	3044.79862	1522.90295	1015.60439	27
12	1294.64268	647.82498	432.21908	F	2931.71455	1466.36091	977.90970	26
13	1407.72675	704.36701	469.91377	I	2784.64613	1392.82670	928.88689	25
14	1520.81082	760.90905	507.60846	L	2671.56206	1336.28467	891.19220	24
15	1607.84285	804.42506	536.61913	S	2558.47799	1279.74263	853.49751	23
16	1720.92692	860.96710	574.31382	L	2471.44596	1236.22662	824.48684	22
17	1834.01099	917.50913	612.00851	L	2358.36189	1179.68458	786.79215	21
18	1965.05149	983.02938	655.68868	M	2245.27782	1123.14255	749.09746	20
19	2112.11991	1056.56359	704.71149	F	2114.23732	1057.62230	705.41729	19
20	2183.15703	1092.08215	728.39053	A	1967.16890	984.08809	656.39448	18
21	2240.17850	1120.59289	747.39768	G	1896.13178	948.56953	632.71544	17
22	2396.27962	1198.64345	799.43139	R	1839.11031	920.05879	613.70829	16
23	2509.36369	1255.18548	837.12608	L	1683.00919	842.00823	561.67458	15
24	2566.38516	1283.69622	856.13324	G	1569.92512	785.46620	523.97989	14
25	2663.43793	1332.22260	888.48416	P	1512.90365	756.95546	504.97273	13
26	2776.52200	1388.76464	926.17885	L	1415.85088	708.42908	472.62181	12
27	2877.56968	1439.28848	959.86141	T	1302.76681	651.88704	434.92712	11
28	2990.65375	1495.83051	997.55610	L	1201.71913	601.36320	401.24456	10
29	3061.69087	1531.34907	1021.23514	A	1088.63506	544.82117	363.54987	9
30	3224.75419	1612.88073	1075.58958	Y	1017.59794	509.30261	339.87083	8
31	3337.83826	1669.42277	1113.28427	L	854.53462	427.77095	285.51639	7
32	3450.92233	1725.96480	1150.97896	I	741.45055	371.22891	247.82170	6
33	3521.95945	1761.48336	1174.65800	A	628.36648	314.68688	210.12701	5
34	3623.00713	1812.00720	1208.34056	T	557.32936	279.16832	186.44797	4
35	3720.05990	1860.53359	1240.69148	P	456.28168	228.64448	152.76541	3
36	3890.16544	1945.58636	1297.39333	K-Acetyl	359.22891	180.11809	120.41449	2
37				K-Acetyl	189.12338	95.06533	63.71264	1

Extracted from: Z:\712AB\SK17-S (Ac)\Chichi-Abaumannii-SK17-R-Acetyl_DrWu_20140703.raw #28708 RT: 78.91
ITMS, CID@35.00, z=+3, Mono m/z=1360.09888 Da, MH+=4078.28208 Da, Match Tol.=0.6 Da

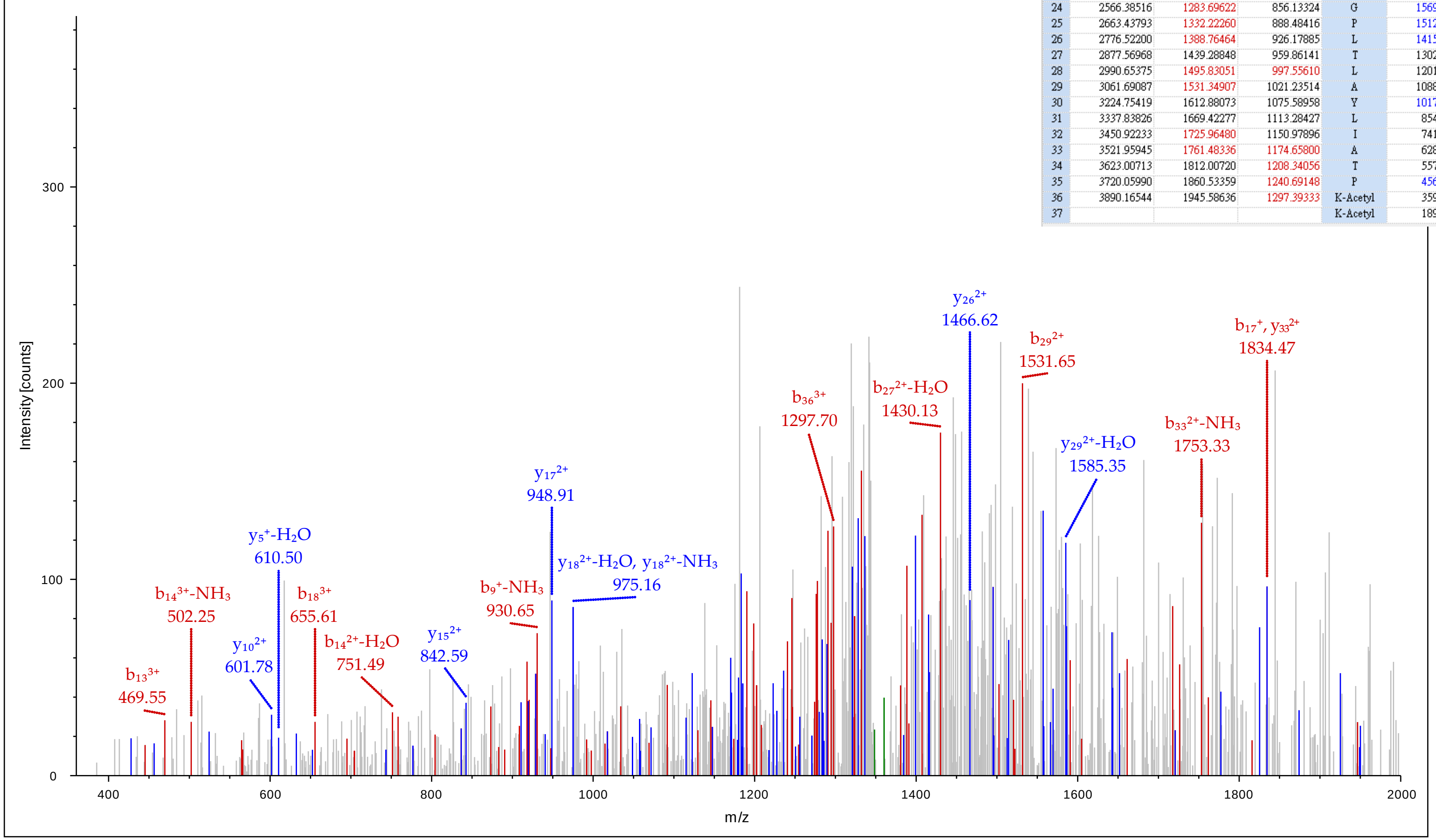

Supplement: Supplementary file 1 [file DataSheet1.pdf]
